# DDX39 as a predictor of clinical prognosis and immune checkpoint therapy efficacy in patients with clear cell renal cell carcinoma

**DOI:** 10.7150/ijbs.62553

**Published:** 2021-07-25

**Authors:** Yewei Bao, Aimin Jiang, Kai Dong, Xinxin Gan, Wenliang Gong, Zhenjie Wu, Bing Liu, Yi Bao, Jie Wang, Linhui Wang

**Affiliations:** 1 Department of Urology, Changhai Hospital, Naval Medical University (Second Military Medical University), Shanghai, China; 2 Department of Urology, Changzheng Hospital, Naval Medical University (Second Military Medical University), Shanghai, China.

**Keywords:** ccRCC, DEAD Box protein 39, prognosis, cancer progression, immune microenvironment, immune checkpoint therapy

## Abstract

DEAD-box protein 39 (DDX39) has been demonstrated to be a tumorigenic gene in multiple tumor types, but its role in the progression and immune microenvironment of clear cell renal cell cancer (ccRCC) remains unclear. The aim of the present study was to investigate the role of DDX39 in the ccRCC tumor progression, immune microenvironment and efficacy of immune checkpoint therapy.

The DDX39 expression level was first detected in tumors in the public data and then verified in ccRCC samples from Changzheng Hospital. The prognostic value of DDX39 expression was assessed in the Cancer Genome Atlas (TCGA) and ccRCC patients from Changhai Hospital. The role of DDX39 in promoting ccRCC was analyzed by bioinformatic analysis and in vitro experiments. The association between DDX39 expression and immune cell infiltration and immune inhibitory markers was analyzed, and its value in predicting the immune checkpoint therapy efficacy in ccRCC were evaluated in the public database. DDX39 expression was elevated in Oncomine, GEO and TCGA ccRCC databases, as well as in Changzheng ccRCC samples. In TCGA ccRCC patients, increased DDX39 expression predicted worse overall survival (OS) (*p*<0.0001) and progression-free interval (PFI) (*p*<0.0001), and was shown as an independent predictive factor for OS (*p*=0.002). These findings were consistent with those from Changhai ccRCC patients. In addition, GO and GSEA analysis identified DDX39 as a pro-ccRCC gene. In vitro experiments confirmed the role of DDX39 in promoting ccRCC cell. Finally, DDX39 was found to be positively correlated with a variety of immune inhibitory markers, and could predict the adverse efficacy of immune checkpoint therapy in TIDE analysis. In conclusion, Increased DDX39 in ccRCC patients predicted worse clinical prognosis, promoted ccRCC cell proliferation, migration and invasion, and also predicted adverse efficacy of immune checkpoint therapy.

## Introduction

Renal cell carcinoma (RCC) is one of the most common tumors of the urinary system and one of the 10 most common cancers in the world. In recent years, the morbidity and mortality of this disease have been increasing gradually. According to the latest statistics, there are about 431,288 new cases of renal cancer worldwide every year, and about 179,368 cases of death 1. Clear cell renal carcinoma (ccRCC) is the most common pathological subtype of renal cancer, accounting for 70~85% of all RCC cases 2. Early diagnosis of renal cancer is relatively difficult due to insipid onset and the absence of specific clinical symptoms in the early stage. About 20-30% RCC patients have already had metastasis at the time of diagnosis, when the therapeutic outcome is usually poor 3. Renal cancer is not sensitive to conventional chemoradiotherapy. Before the advent of targeted drugs such as Sunitinib, Sorafenib and Everolimus, the 2-year survival rate of renal cancer was less than 20%. At the same time, some patients will have primary and secondary drug resistance, which brings great challenges to the treatment of renal cancer 4. With advances in oncoimmunoclinical trials, the American Food and Drug Administration (FDA) has recommended Nivolumab, Cabozantinib, Pembrolizumab and other monotherapy or combination therapy as the first-line and second-line treatment for advanced renal cancer. However, the kidney cancer immunobenefit population is limited and there is currently a lack of characteristic studies on benefit-based patients 5, 6. Therefore, it is an urgent task to study the occurrence and progression of renal cancer from other molecular perspectives, explore new therapeutic targets and find new markers to predict the immune efficacy and prognosis of renal cancer.

The molecular family of dead-box proteins, which exist in many species from viruses to prokaryotes to eukaryotic mammals, is a class of ATP-dependent RNA helicases. So far 40 family members have been identified, and all of them are characterized by 9 highly conserved sequences and named after the sequence II D-E-A-D (Asp-Glu-Ala-Asp) 7. Dead-box protein plays an important role in the recognition of innate immune function, regulation of intracellular signaling pathways, RNA transcription and splicing, and regulation of nuclear epigenetics by regulating gene expression and RNA metabolism in organisms 8, 9. Studies have shown that dead-box protein is closely related to the development and progression of tumors, and that different family members can play a role in either inhibiting or promoting a variety of tumors 10.

DEAD-box protein 39 (DDX39) may play a role in promoting tumor cell proliferation and metastasis in the process of tumor development. Studies have shown that DDX39 is highly expressed in lung squamous cell carcinoma, malignant pleural stromal and gastric stromal tumors, and high expression of DDX39 is closely related to the prognosis of patients 11-13. At the same time, DDX39 may be involved in drug resistance of tumors. It was found that DDX39 expression in gemcitabine-induced pancreatic cancer-acquired drug resistance cell lines was significantly higher than that in sensitive cell lines 12. However, the role of DDX39A in ccRCC is still unclear. In the present study, we analyzed the public databases and corresponding external validation experiments, founding that the high expression of DDX39A in ccRCC was closely associated with multiple clinical characteristics of ccRCC patients and predicted adverse clinical outcomes. In addition, DDX39A may affect a variety of immune cells and induce tumor immune escape in the ccRCC immune microenvironment. Finally, increased DDX39 may compromise the immune checkpoint therapy efficacy in ccRCC patients. All these findings may provide a new direction for the diagnosis and treatment of ccRCC.

## Materials and methods

### Patient and clinical samples

Twenty-nine ccRCC and their adjacent tissue samples were randomly selected from the Biobank of the Urology Department of Changzheng Hospital (Shanghai, China) for real-time quantitative PCR (RT-qPCR) and Western blotting. They were obtained from patients who underwent radical nephrectomy or partial nephrectomy from June 2011 to September 2016. Changhai ccRCC Tissue microarray was made from samples of 186 ccRCC cases from January 2007 to April 2014 in the said Hospital. The characteristics of these samples, including gender, age, tumor size, TNM stage and Feynman (Fuhrman) grade, were collected and their pathological features of ccRCC were evaluated by two pathologists who specialized in urinary system tumors. This study was approved by the ethics committee of the Naval Medical University (Shanghai, China). All the included patients signed the informed consent.

### DDX39 is highly expressed in Oncomine, TCGA, GEO and Changzheng ccRCC patients

Analysis of DDX39 expression in multiple tumors was performed in Oncomine database (https://www.oncomine.org/resource/login.html), using |Foldchange|>1.5 and p<0.01 as the screening condition. Transcriptome profiling data were obtained from TCGA (The Cancer Genome Atlas) database and processed using R scripts. Differential expressions of DDX39 in paired and unpaired samples of TCGA cohort were analyzed and drawn using R package ggplot2 and ggpubr. GSE16449 (including transcriptome data of 52 renal cancer tissues and 18 normal kidney tissues) and GSE40435 (including transcriptome data of 110 paired renal cancer and adjacent normal tissues) were downloaded from GEO database. External validation of TCGA cohort analysis was done using ggplot2 and ggpubr packages in GES16449 and GES40435. The methylation status of DDX39 promoter in ccRCC was examined in UALCAN database (http://ualcan.path.uab.edu/index.html). Representative immunohistochemical images of DDX39 expression in ccRCC patients were retrieved from HPA database.

RT-qPCR was performed to identify the DDX39 expression in 29 paired ccRCC and adjacent normal tissues. The RNA of samples and cells was extracted using Trizol lysate (RNAiso Plus, T9108, TaKaRa) according to the instructions. According to the manufacturer's instructions of reverse transcription kit (PrimeScriptTM RT Master Mix, RR036A, Takara), the reaction system was configured for reverse transcriptase reaction. Primer sequences for RT-qPCR are shown in **Table S1**. High-throughput and fast real-time fluorescence quantitative PCR (Applied Biosystems 7900HT) was used in the experiment, using SYBR® Premix Ex TaqTM (Tli RNaseH Plus, RR820A, TaKaRa) as the reaction reagent. The mean value of internal reference was calculated according to the 2^-ΔΔCT^ formula, and finally the difference between the target gene and the control group was obtained. Western blot was conducted to detect DDX39A expression in four paired ccRCC and adjacent normal tissues. The protein of the sample was obtained by RIPA lysis buffer (Pierce RAPI Buffer, 89900, ThermoFisher Scientific). After denaturation, the protein samples were separated by sodium dodecylsulfate-polyacrylamide gel electrophoresis (SDS-PAGE) and transferred onto the PVDF membrane. DDX39 (DDX39A Polyclonal Antibody, 11723-1-AP, Proteintech) and β-actin antibodies (β-actin Rabbit mAb, 4870, CST) were diluted to 1:1000, and the second antibody (IRDye®800CW Goat anti-Rabbit IgG, bs-40295G-IRDye8, Bioss) was incubated and then detected using an Odyssey CLx scanner (Odessey CLx Studio 3.1, Li-Cor Biosciences Inc.).

### Higher DDX39 expression predicts worse prognosis in TCGA and Changhai ccRCC cohort

Prognostic data were obtained from TCGA (The Cancer Genome Atlas) database and processed using R scripts, and p<0.05 was considered clinically significant. We grouped KIRC patients with the median value of DDX39A expression and divided them into a DDX39 high expression group and a DDX39 low expression group, and used log-rank test for hypothesis testing. At the same time, patients with an OS less than 30 days were excluded. Survival and survminer package were used to draw overall survival (OS) and progression-free interval (PFI) survival curves. Univariate cox and multivariate cox regression analyses on DDX39 expression and other clinical indicators associated with OS were performed through Survival package and the results of multivariate analysis was visualized as a forest plot using Forestplot package. Time dependent Roc curve on DDX39 in TCGA ccRCC patients was drawn by the R package ggplot2.

Immunohistochemical (IHC) stanning was performed on Changhai ccRCC tissue microarray using DDX39 antibody (DDX39A Polyclonal Antibody, 11723-1-AP, Proteintech, diluted at 1:50). The IHC procedure referred to the previous protocol 14. The IHC results were analyzed by Aperio ImageScope software. The clinical data together with the IHC data were analyzed by SPSS 21.0 software, and the continuous variables were represented by the mean ± standard deviation (SD). Pearson's Chi-square test was used to analyze the clinical data of RCC patients. The relationship between DDX39 expression and survival of RCC patients was analyzed by Kaplan-Meier test. Cox proportional hazard regression analysis for OS and disease-free survival (DFS) was used to analyze the independent factors of survival and prognosis in RCC patients. Two-sided P-values less than 0.05 indicated statistical significance.

### GO, KEGG and GSEA analysis

Spearman's correlations between DDX39 and 19712 genes retrieved from TCGA transcriptome data were analyzed. Sorting by the level of association between other genes and DDX39, genes most related to DDX39 expression were selected for enrichment analysis according to the guilt-of-association method. Our annotation method relies on large samples of TCGA, knowing that it can annotate genes and any gene related to DDX39 in ccRCC can be annotated. The method adopted was Pearson's correlation, and significance was defined at p<0.05 and |cor|>0.5. ClusterProfiler package was used to perform functional annotation analysis (GO), KEGG and gene set enrichment analysis (GSEA) on related genes.

### Validation of DDX39 function in ccRCC progression in vitro

Human normal renal tubular epithelial cell line HK-2, human RCC cell lines A498, ACHN, 786-O, 769-P, Caki-1, Caki-2, OS-RC-2 and KETR-3 were purchased from ATCC cell bank of the United States. Cell lines were cultured according to the instructions. Small interfering RNA (siRNA) and its negative control reagents were purchased from GenePharma Company. The plasmid pCMV-Myc-DDX39 in this experiment was gifted by Xiao Yuzhong Research Group, College of Life Sciences, Hunan Normal University. pCMV-Myc-DDX39 was transfected into cells according to the instruction manual of Lipofectamine3000 (Lipofectamine^TM^ 3000 Transfection Reagent, L3000015, Invitrogen). The transfection efficiency was detected by RT-PCR and Western blot assay. Sequences of the primers used for siRNAs and plasmid structures are shown in **Table S2** and **Figure S1.A**.

CCK-8 (Cell Counting Kit-8, DJDB4000X, Dojindo Molecular Technologies) reagent was used to detect cell proliferation. Cells were seeded into a 96-well plate at a density of 2×10^3^ cells per well before transfection. The number of RCC cells was assessed from at least three replicates in three independent experiments after transfection. In clone formation assay, 600 cells were added into each well of a 6-well plate and cultured for 14 days. Then, the cells were fixed with 4% formaldehyde and stained with crystal violet. Cell clones were counted and analyzed. The migration and invasion abilities of ccRCC cells were measured by the number of cells passing through the Matrigel-coated Tranwell inserts (Corning® BioCoat^TM^ Matrigel® Invasion Chambers, 354480, Corning Life Sciences) after 24 h. 500ul medium containing 10% serum was added to the 24-well plate, and then cells diluted in FBS-free medium (1×10^5^/ml) were seeded into the transwell sert. After 24 hours, the chamber was taken out, cleaned, fixed with paraformaldehyde and stained with crystal violet.

### Heatmap and ssGSEA analysis

Based on the transcriptome data retrieved from TCGA ccRCC cohort, 500 genes with mRNA expression related to DDX39 were extracted by Pearson's correlation analysis. R Pheatmap package was used to draw correlation heatmaps of the 500 genes' expression, using |cor|>0.5 and *p*<0.05 as the screening condition. R Estimate package was used to calculate ImmuneScore, estimate score and stromalscore. ssGSEA (single sample Gene Set Enrichment Analysis) algorithm was used to calculate enrichment scores of immunity-related pathways and predictive pathways for immunotherapy efficacy.

### Analysis of the association between DDX39 expression and immune cell infiltration and genes

Association between DDX39 expression and immune cell infiltration was analyzed through Timer 2.0 online database (http://timer.comp-genomics.org/). We collected 28 immune cell markers based on literature research 15 (**Table S3**). ccRCC patients from TCGA cohort were divided into a DDX39 high expression group and a DDX39 low expression group based on the median expression of DDX39, and the infiltration of the 28 immune cells in different groups and the association of DDX39 expression between them were analyzed using the GSVA package. The normalized enrichment score (NES) was calculated using immune infiltration ssGSEA algorithm, and the correlation between DDX39 and NES was analyzed by Pearson's correlation. Visualization was done by ggplot package, and at the same time the correlation between DDX39 expression and the expression of 1989 immunity-related genes was analyzed by Pearson's correlation (**Table S4**). Under the screening condition of |cor|>0.5 and *p*<0.05, 83 genes that were highly correlated with DDX39 were selected to perform GO (Gene Ontology) analysis solely based on 60 positively associated genes again.

### Analysis of the correlation of DDX39 expression with immune-inhibitory markers and TIDE analysis

Recent studies have revealed two different mechanisms of tumor immune evasion: some tumors are often in a state of dysfunction despite high infiltration of cytotoxic T cells, while in other tumors immunosuppressive factors can remove T cells that infiltrate into the tumor tissue. Therefore, Peng et al (16) designed a new computing framework known as Tumor Immune Dysfunction and Exclusion (TIDE) score to integrate these two tumor immune escape mechanisms. In the present study, we uploaded the transcriptome data of 531 ccRCC samples to TIDE database (http://tide.dfci.harvard.edu) and calculated the correlation of DDX39 with the dysfunction and TIDE scores. The response to immune checkpoint therapy groups was calculated and compared between DDX39 high expression group and low expression group using Chi-square test.

### Statistical methods

The experimental results were analyzed by SPSS 21.0 software. Continuous variables were represented by the mean ± standard deviation (SD), and categorical variables were represented by frequencies and percentages. Comparison between groups was done by Student t-test and Mann-Whitney U test. The grey values of western blots were analyzed with Image Lab software. The clone and cell numbers in clone formation and transwell assays were detected by ImageJ software. P value less than 0.05 indicated statistical significance.

## Results

### DDX39 is highly expressed in Oncomine, TCGA, GEO and Changzheng ccRCC patients

**Figure 1:**
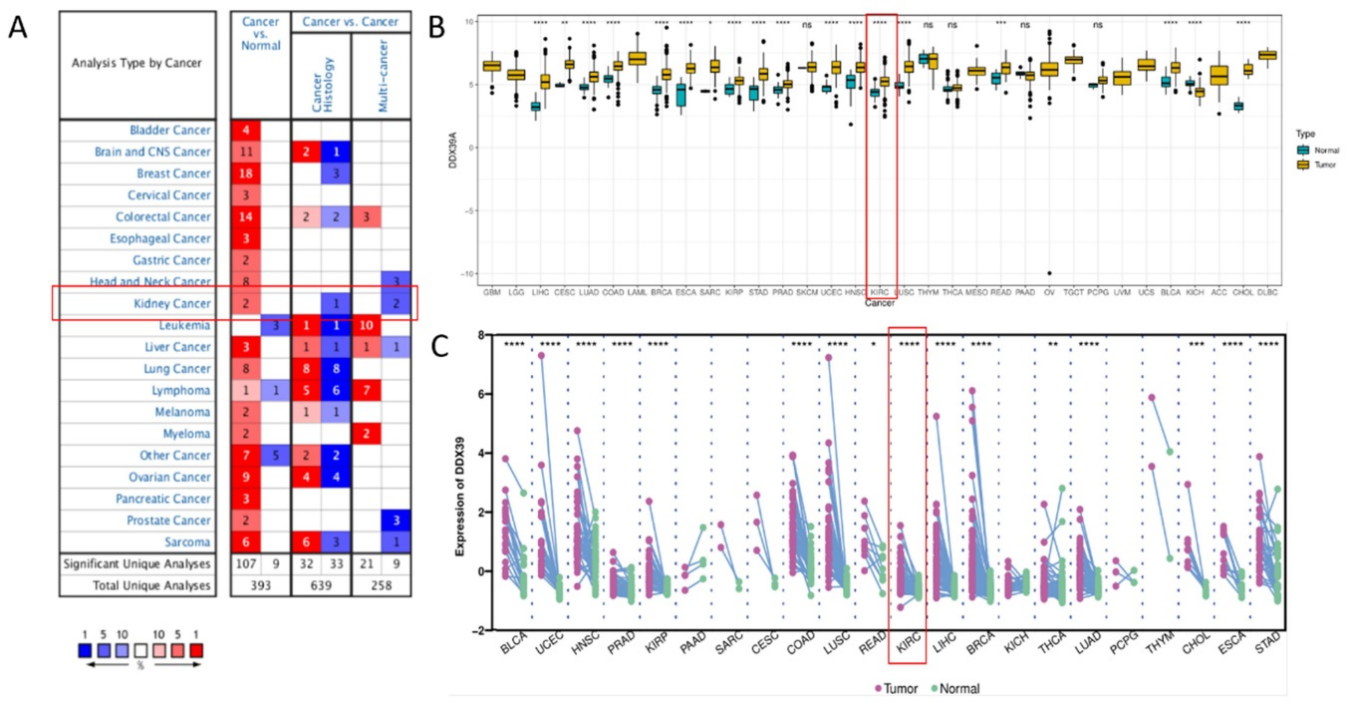
** Pan-cancer analysis of DDX39 expression. A.** Expression levels of DDX39 in 20 different cancer types, derived from Oncomine database. **B.** Differential expression levels of DDX39 in 33 cancer types and corresponding normal tissues from TCGA database, *p<0.05, **p<0.01, ***p<0.001, ****p<0.0001. **C.** Differential expression levels of DDX39 in paired cancer and adjacent normal tissues of 22 cancer types from TCGA database, *p<0.05, **p<0.01, ***p<0.001, ****p<0.0001.

Oncomine DDX39 expression meta-analysis in cancer* vs.* normal tissues showed that DDX39 was highly expressed in kidney, bladder, brain, cervical, colorectal, esophageal, gastric cancer, head and neck, liver cancer, lung, ovarian, pancreatic and prostate cancers, as well as in lymphoma, melanoma, myeloma and sarcoma (**Figure 1A, Table S5**). In TCGA pan-cancer cohort, DDX39 was highly expressed in cancer* vs.* normal tissues of 18 cancer types (**Figure 1B**), as well as in cancer* vs.* paired adjacent tissues of 16 cancer types (**Figure 1C**).

**Figure 2:**
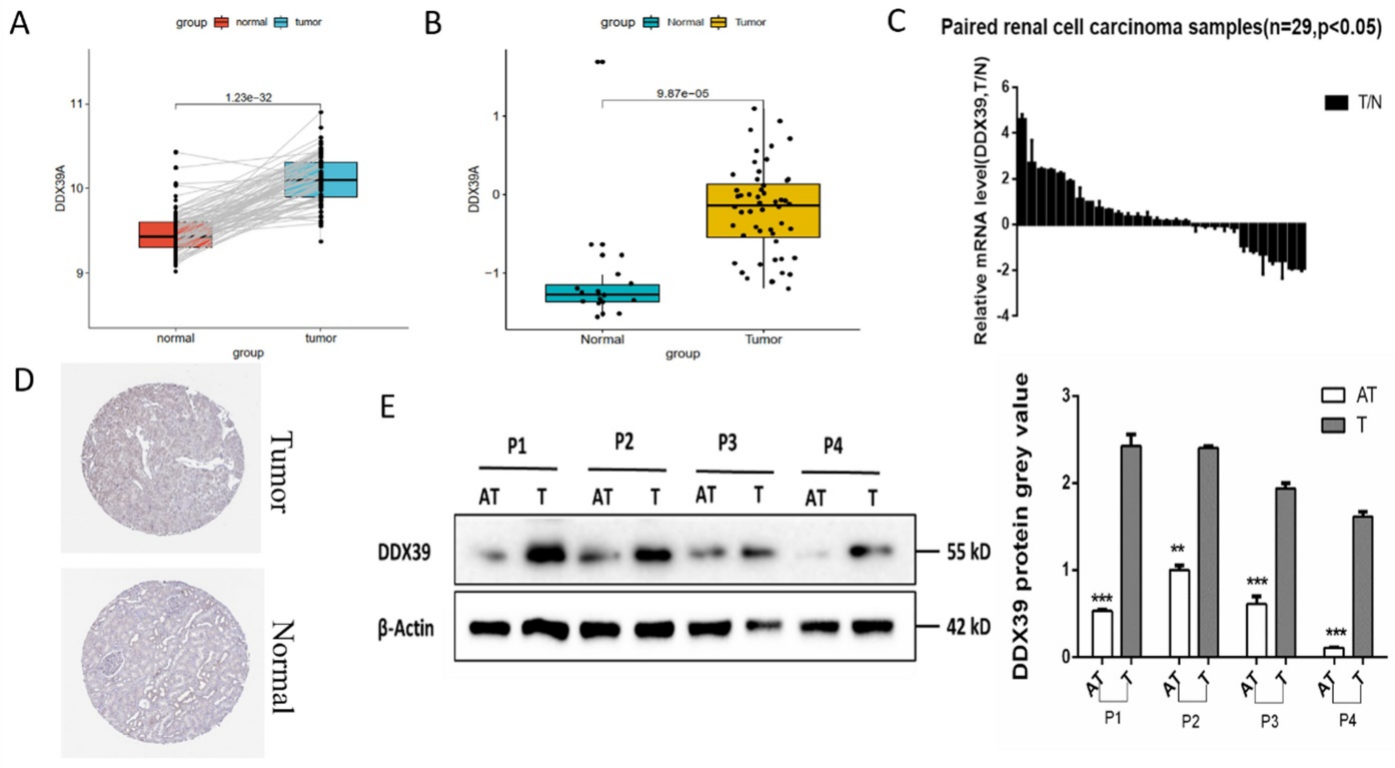
** DDX39 is highly expressed in renal clear cell carcinoma. A.** DDX39 expression levels in GEO GSE40435 dataset, *p*<0.0001. **B.** DDX39 expression levels in GEO GSE16449 dataset,* p*<0.0001. **C.** RT-PCR results of DDX39 expressions of samples from Changzheng cohort, n=29, T: tumor, N: normal tissue, *p*<0.05. **D.** Representative IHC images of DDX39 expression retrieved from HPA database. **E.** Western blot analysis of DDX39 protein expression between the adjacent tissue (AT) and tumor (T) from Changzheng cohort. The gray value shows the difference between two groups, **p<0.01, ***p<0.001.

GEO GSE40435 dataset (110 paired ccRCC and adjacent normal tissues) showed that the DDX39 expression in ccRCC tissue was significantly higher than that in the adjacent normal tissues (**Figure 2A**). Reverse transcription polymerase chain reaction (RT-PCR) showed that the mRNA expression of DDX39 in ccRCC tissue was also significantly higher than that from Changzheng hospital (n=29) (**Figure 2B**). GEO GSE16449 dataset (52 ccRCC and 18 normal tissues) showed that DDX39 was highly expressed in ccRCC as compared with that in the normal tissue (**Figure 2C**). IHC images retrieved from HPA database demonstrated higher expressions of DDX39 in ccRCC tissue (**Figure 2D**). Higher levels of DDX39 protein expression were detected in four pairs of ccRCC and adjacent normal tissues from Changzheng hospital (**Figure 2E**).

### Higher DDX39 expression predicts worse prognosis of TCGA and Changhai ccRCC cohort

**Figure 3:**
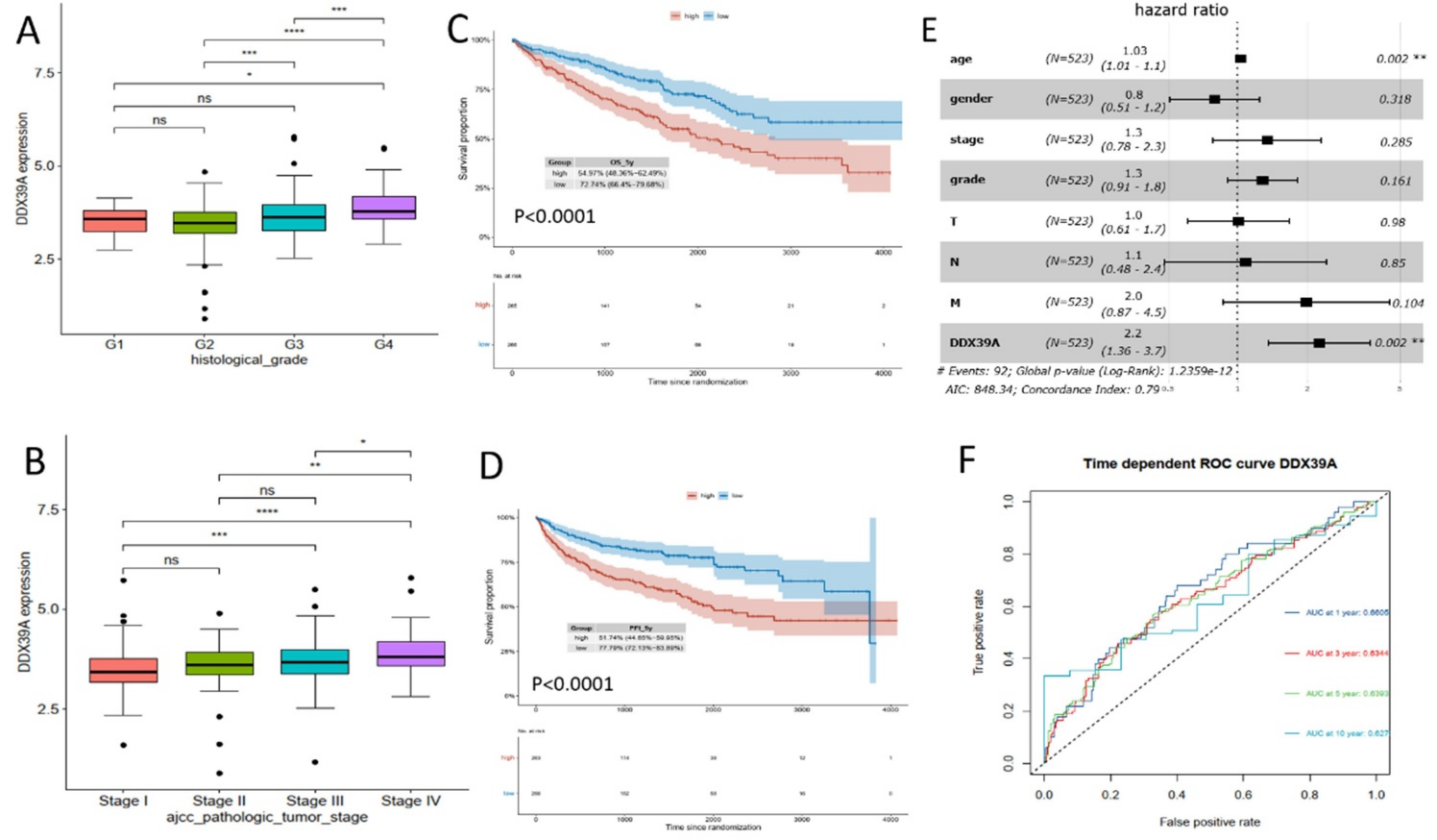
** DDX39 expression is related to clinical prognosis. A.** DDX39 expression in TCGA ccRCC patients with different histological grade, *p<0.05, ***p<0.001, ****p<0.0001. **B.** DDX39 expression in TCGA ccRCC patients with different AJCC clinical stage, *p<0.05, **p<0.01, ***p<0.001, ****p<0.0001. **C.** Kaplan-Meier analysis of OS of TCGA ccRCC patients with high or low DDX39 expression, p<0.0001. **D.** Kaplan-Meier analysis of PFI of TCGA ccRCC patients with high or low DDX39 expression, p<0.0001. **E.** Multivariate COX analysis on DDX39 expression and other clinical factor associated with OS in TCGA ccRCC patients, **p<0.001. **F.** Time dependent ROC curve on DDX39 in TCGA ccRCC patients.

Transcriptome data and prognosis data of 531 ccRCC patients were downloaded from TCGA database. It was found that DDX39 expression in ccRCC patients with a high histological grade was higher than that in patients with a low histological grade (**Figure 3A**). At the same time, DDX39 expression in patients with a high AJCC clinical stage was higher than that in patients with a low AJCC clinical stage (**Figure 3B**). Prognosis analysis showed higher OS and PFI in ccRCC patients with high expression of DDX39 (both p<0.0001) (**Figure 3C, D**). Subsequent univariate COX analysis showed that DDX39 expression was a predictive factor for OS of ccRCC patients (*p*<0.0001) and remained as an independent factor in multivariate COX analysis (*p*=0.002) (**Figure 3, Table 1**). Consistently, the area under the ROC curve (AUC) based on 1-, 3-, 5- and 10-year survival was 0.6605, 0.6344, 0.6393, 0.627 respectively (**Figure 3F**).

**Figure 4:**
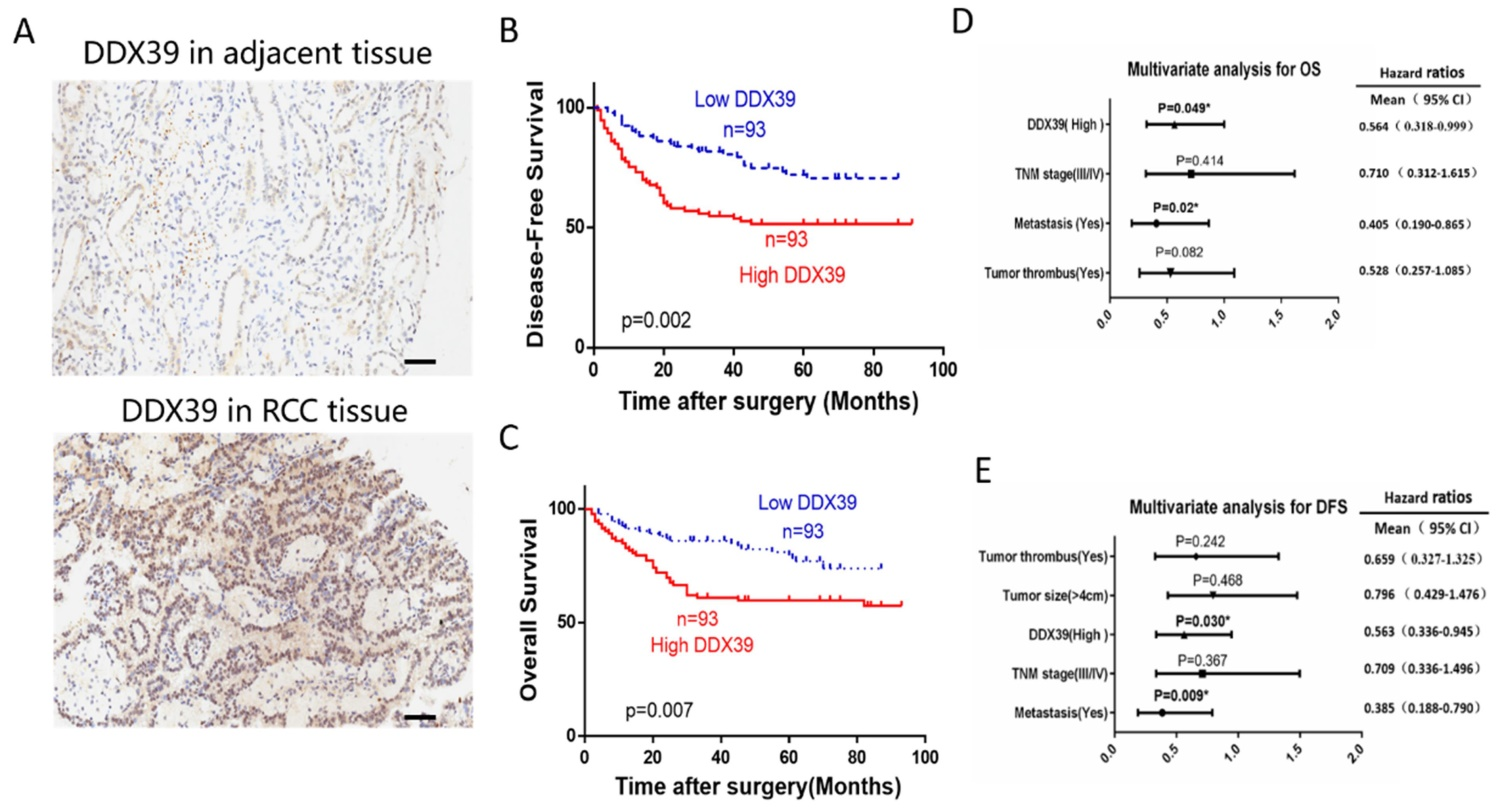
** DDX39 expression is related to clinical prognosis in RCC patients of Changhai cohort. A.** Representative immunohistochemical images of DDX39 expression in ccRCC and adjacent tissues, scale bar = 50um. **B.** Kaplan-Meier analysis of the disease-free survival of ccRCC patients with high or low DDX39 expression. **C.** Kaplan-Meier analysis of the overall survival of ccRCC patients with high or low DDX39 expression. **D.** Multivariate analyses of factors associated with overall survival in ccRCC patients, n = 186, *p<0.05. **E.** Multivariate analyses of factors associated with disease-free survival in ccRCC patients, n = 186, *p<0.05.

IHC representative images are shown in** Figure 4A**. In Changhai ccRCC cohort (n=186), patients were divided into a DDX39 high expression group (n=93) and a DDX39 low expression group (n=93) according to the median expression of DDX39. Pearson's Chi square test showed a positive correlation of high DDX39 expression with tumor size (*p*=0.007), TNM stage (*p*=0.011) and metastasis (*p*=0.001) (**Table 2**), while higher DDX39 expression, larger tumor size, more advanced TNM stage, more tumor emboli and metastases were predictive factors for PFS (**Table 2**). In addition, Kaplan-Meier analysis showed that patients in DDX39 low expression group exhibited better DFS (*p*=0.002) (**Figure 4D**) and OS (*p*=0.007) (**Figure 4E**). Univariate COX analysis showed that higher DDX39 expression (*p*=0.007), more advanced TNM stage (*p*<0.001), the presence of cancer embolus (*p*<0.001) and metastasis (*p*<0.001) were predictive factors for OS. Multivariate COX analysis identified higher DDX39 (*p*=0.049) and existence of metastasis (*p*=0.020) as independent predictive factors for OS (*p*= 0.049) and PFS (*p*=0.020) (**Table 3, 4, Figure 4D, E**). Plus, the baseline information comparison between TCGA cohort and Changhai chohort was shown in **Table S6**.

### DDX39 functioning as RNA processing protein activates proto-oncogene MYC and accelerates cell cycle progression in GO and GSEA analysis

**Figure 5:**
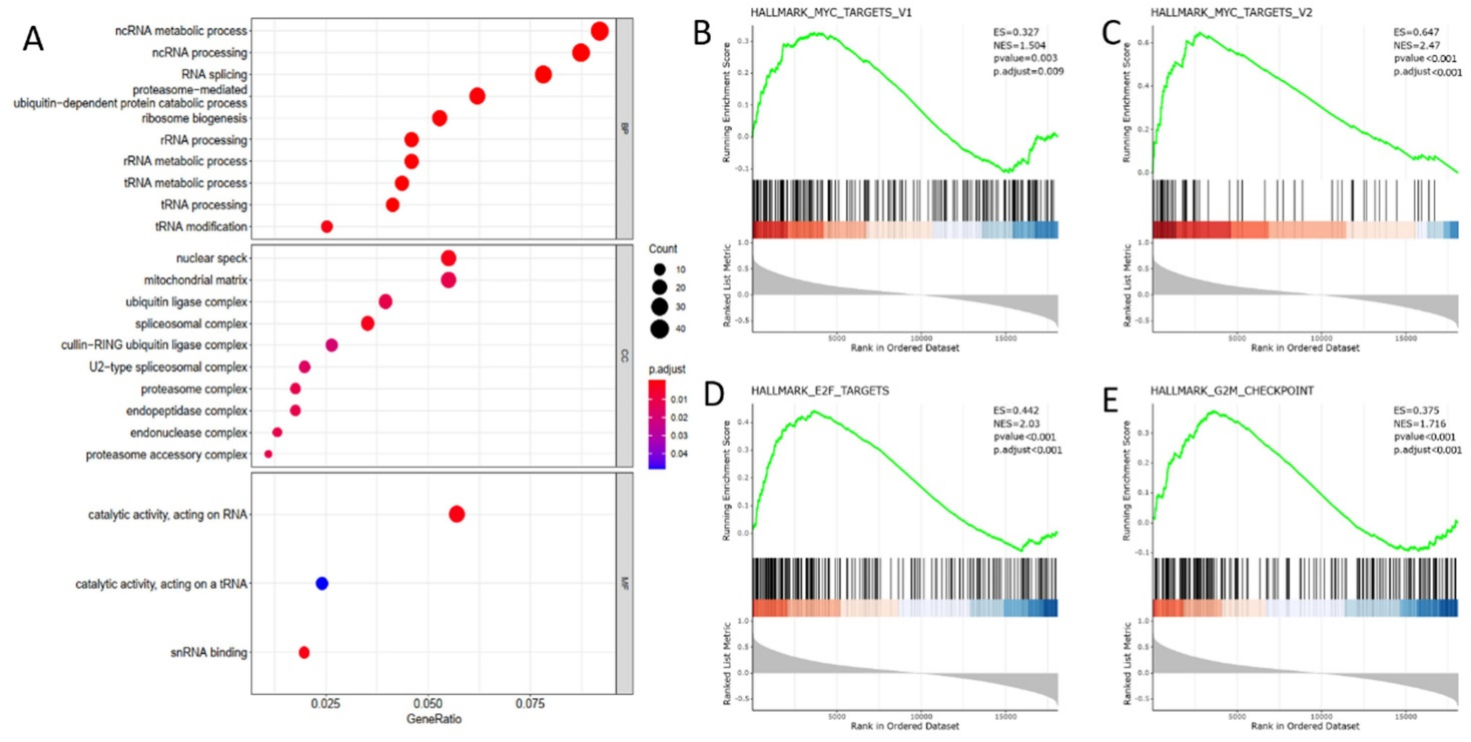
** DDX39 functions as RNA processing protein and associates with MYC and cell cycle acceleration hallmarks in GO and GSEA analysis. A.** DDX39 is associated with RNA processing functions in BP module of GO analysis. **B.** DDX39 is associated with MYC_targets_V1 hallmark in GSEA analysis, p.adjust=0.009. **C.** DDX39 is associated with MYC_targets_V2 hallmark in GSEA analysis, p.adjust<0.001. **D.** DDX39 is associated with E2F hallmark in GSEA analysis, p.adjust<0.001. **E.** DDX39 is associated with G2M hallmark in GSEA analysis, p.adjust<0.001.

To elaborate the biological function of DDX39 in ccRCC progression, we selected 500 genes whose expressions were most correlated with DDX39 expression from 19,712 genes retrieved from TCGA transcriptome data (**Table S7**). GO analysis using the biological process module showed that DDX39 was mainly involved in ncRNA metabolic, ncRNA processing and RNA splicing processes, and the working environment of DDX39 mainly involved nuclear speck, mitochondrial matrix and ubiquitin ligase complex in the cell compartment module (**Figure 5A**).

GSEA analysis revealed that a series of classical hallmarks participated in cancer initialization (such as proto-oncogene MYC hallmarks) and cancer cell cycle acceleration (such as G2M checkpoint hallmark and cell cycle transcription factor E2F hallmark) (**Figure 5B**). The results showed that DDX39 expression was closely related to hallmark_MYC_targets_V1 (*p*. adjust=0.009), hallmark_MYC_targets_V2 (*p*.adjust < 0.0001), hallmark_E2F_targets (*p*<0.0001) and hallmark_G2M_checkpoint (*p*<0.0001) (**Figure 5C**).

### DDX39 promotes the proliferation, migration and invasion of renal cancer in vitro

**Figure 6:**
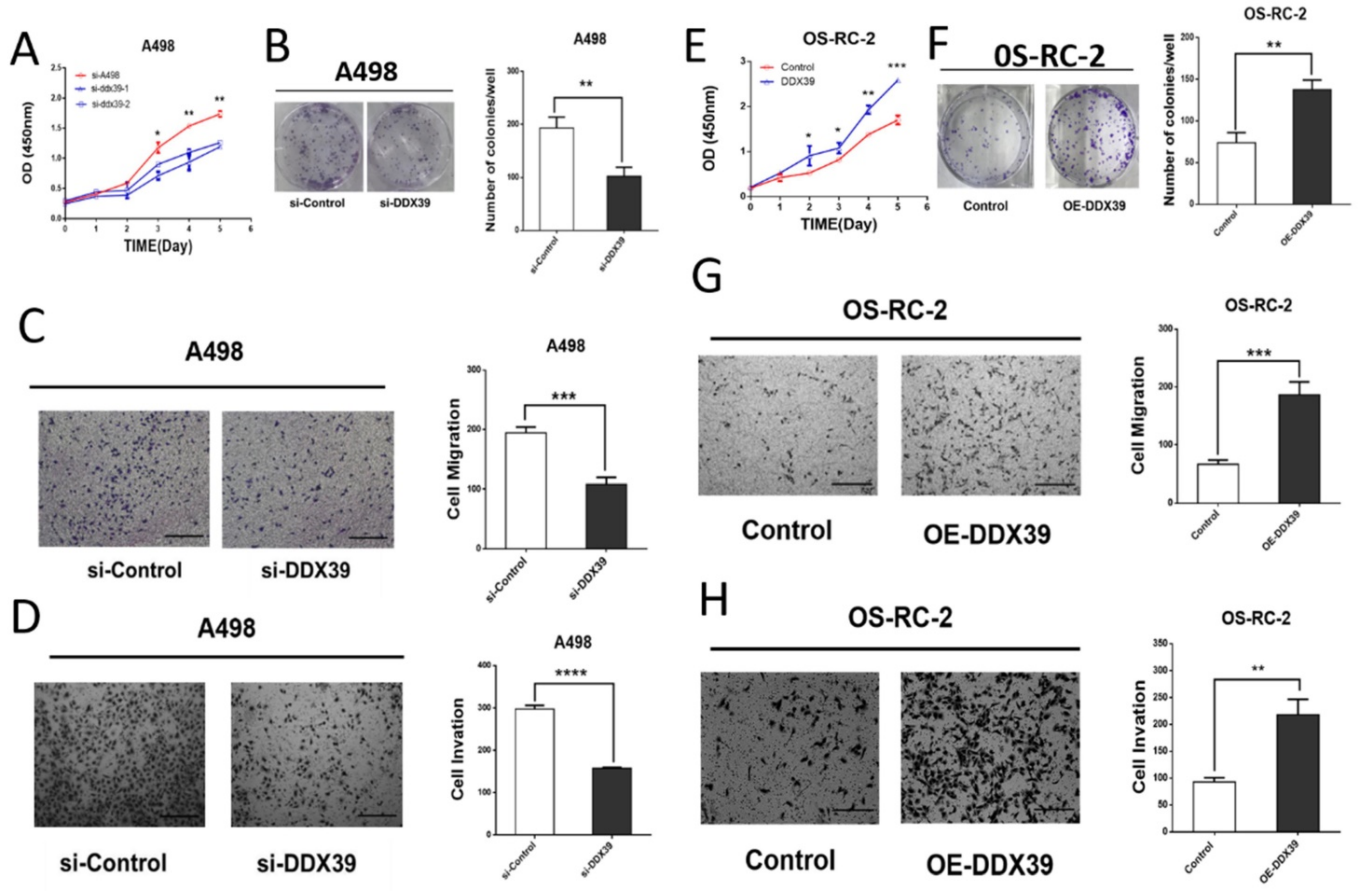
** DDX39 promotes renal cancer cell proliferation, migration and invasion in vitro. A.** Cell proliferation of A498. Si-A498: negative control, si-DDX39: treated with si-RNA, bars indicated SD. *<0.05, **P<0.01. **B.** Cell colonies of A498 after treated with si-DDX39, **P<0.01. **C.** Cell migration of A498 after treated with si-DDX39, ***P<0.001, scale bar = 200um. **D.** Cell invasion of A498 after treated with si-DDX39, ****P<0.0001, scale bar = 200um. **E.** Cell proliferation of OS-RC-2. Control: EV, DDX39: treated with DDX39 overexpression plasmid, bars indicated SD. *P<0.05, **P<0.01, ***P<0.001. **F.** Cell colonies of OS-RC-2 after treated with DDX39 overexpression plasmid, **P<0.01. **G.** Cell migration of OS-RC-2 after treated with DDX39 overexpression plasmid, ***P<0.001, scale bar = 200um.

Small interfering RNAs (siRNA) targeting DDX39 was transfected into RCC cell lines. According to the DDX39 expression in different cell lines, OS-RC-2 and ACHN were detected by RT-PCR for further validation (**Figure S1B**). Kock-down and overexpression efficiency was confirmed by RT-PCR and western blotting (**Figure S1C, D, Figure S2A, B**). The results of cell proliferation and colony formation experiments showed that the proliferation ability of A498 and ACHN decreased after knocking down the DDX39 expression (**Figure 6A, B, Fig. S2C**). Similarly, the migration and invasion of A498 decreased after knocking down DDX39 expression (**Figure 6 C, D**). CCK-8 and colony formation experiments showed that the ability of cell proliferation was enhanced after overexpression of DDX39 in OS-RC-2 and ACHN as expected (**Figure 6E, F, Figure S2C, D**). Also, the migration and invasion ability of OS-RC-2 cells was enhanced after overexpression of DDX39 (**Figure 6 G, H**).

### DDX39 is related to ImmuneScore and immunity-related functions in heatmap and ssGSEA analysis

**Figure 7:**
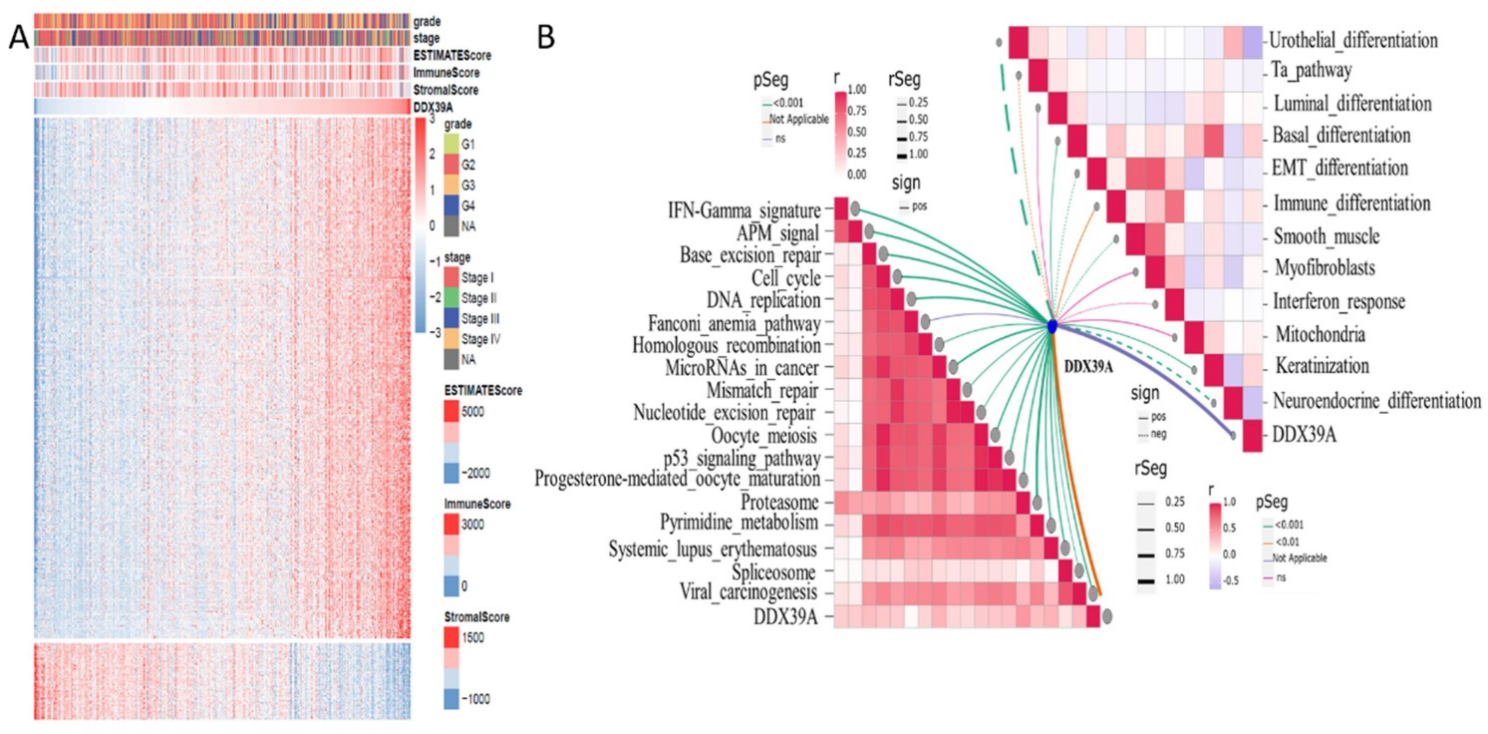
** Increased DDX39 is related to higher Estimatescores and immunotherapy predicting pathways in heatmap and ssGESA analysis. A.** Heatmap of associations between Estimatescores and DDX39 in TCGA ccRCC patients. **B.** ssGSEA analysis of associations between immunotherapy predicting pathways and DDX39 in TCGA ccRCC patients.

Based on transcriptome data retrieved from TCGA ccRCC cohort, 500 genes whose mRNA expression was mostly related to DDX39 were extracted by Spearman's correlation analysis. R Pheatmap package was used to draw correlation heatmaps of expression the 500 genes under the screening condition of |cor|>0.5 and *p*<0.05. R Estimate package was used to calculate ImmuneScore, estimate score and stromalscore. Higher ImmuneScore was exhibited in clusters with higher DDX39 expression (**Figure 7A**). Moreover, ssGSEA (single sample Gene Set Enrichment Analysis) algorithm was used to calculate enrichment scores of immunity-related pathways and predictive pathways for immunotherapy efficacy. The association between DDX39 and immunotherapy-predicting pathways was exhibited by R ggcor package. It was found that higher DDX39 expression was positively associated with immunotherapy-predicting pathways (**Figure 7B**).

### DDX39 is related to immune infiltration and activates the antigen presentation process

**Figure 8:**
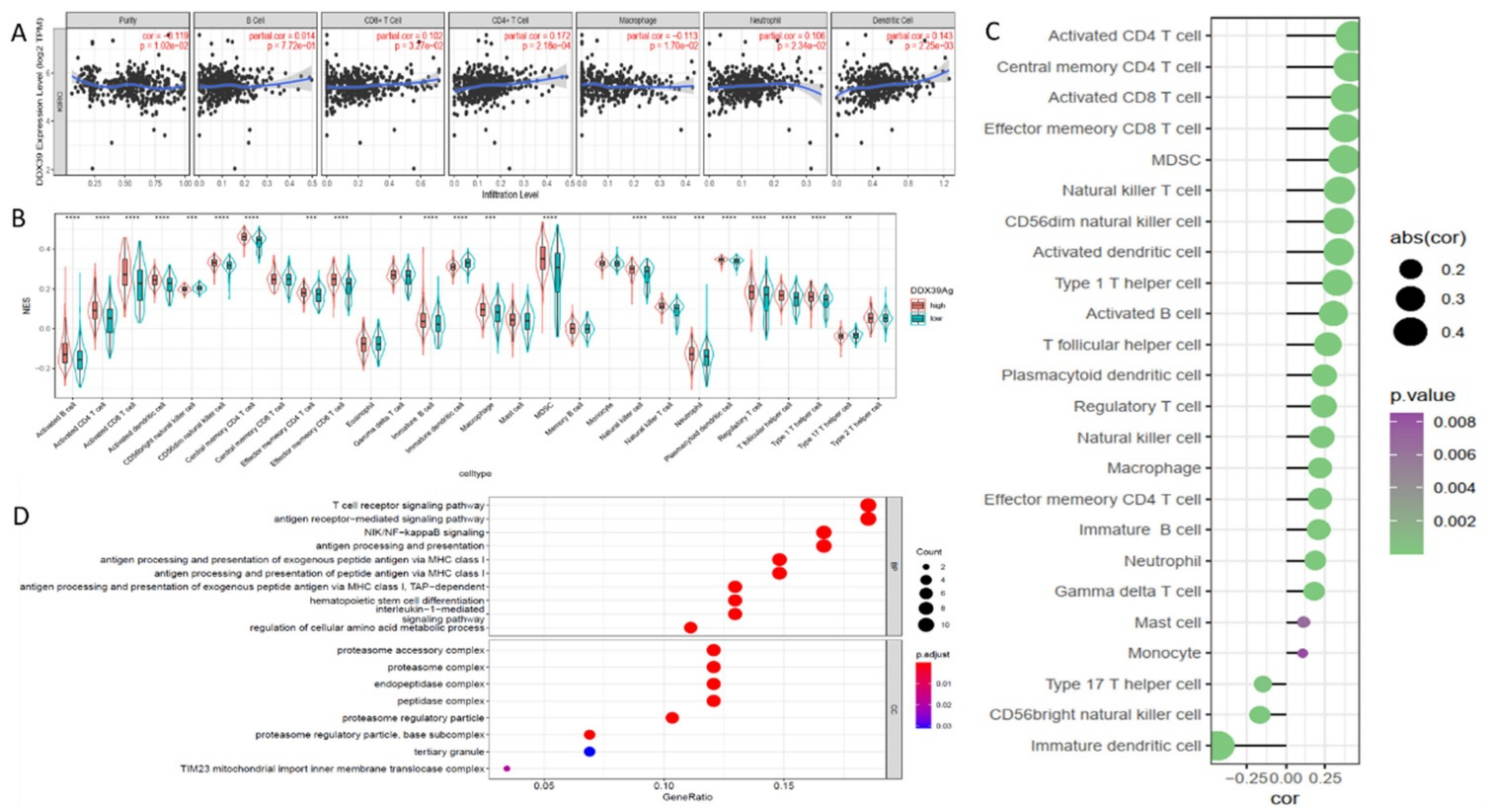
** DDX39 expression is related to immune infiltration and associates with the antigen presentation process in ccRCC patients. A.** DDX39 is associated with multiple immune cell infiltration in ccRCC in TIMER2.0 database. **B.** Different DDX39 expression in 28 immune cells in ccRCC patients from TCGA database, *p<0.05, **p<0.01, ***p<0.001, ****p<0.0001. **C.** Associations between DDX39 expression and NES of 28 immune cells in ccRCC from TCGA database. **D.** DDX39 is related to a variety of immune processes in ccRCC from TCGA database in GO analysis.

Knowng that immune cell infiltration in the tumor microenvironment (TME) may affect the immunotherapy efficacy and the subsequent outcome of ccRCC patients, we examined DDX39 expression and found a positive correlation with B, CD4^+^T cell and CD8^+^T cell, macrophage, neutrophil and dendritic cell infiltration in the data downloaded from TIMER database (**Figure 8A**). ssGSEA analysis iedentified multiple types of immune cell infiltration in TCGA ccRCC samples, among which central memory CD4^+^T, activated CD4^+^T and effector memory CD8^+^T cells found to be positively correlated with DDX39 expression, while immature dendritic, CD56^+^(bright) nature killer and type 17 T helper cells were found to be negatively correlated with DDX39 expression (**Figure 8B, C**). Eighty-three genes highly correlated with DDX39 were selected to perform GO analysis (**Table S8, Figure S3**). It was found that gene function annotations of top 20 genes from the 83 genes mainly pointed to antigen presentation and immune metabolism (**Table S9**). GO analysis of 60 positively associated genes showed that DXX39 had a particular association with the antigen-presentation function (**Figure 8D**). In all, DXX39 was closely related to immune cell infiltration and the antigen presentation function.

### Increased DDX39 is related to higher immune dysfunction score and worse immune check-point therapy efficacy in ccRCC patients

**Figure 9:**
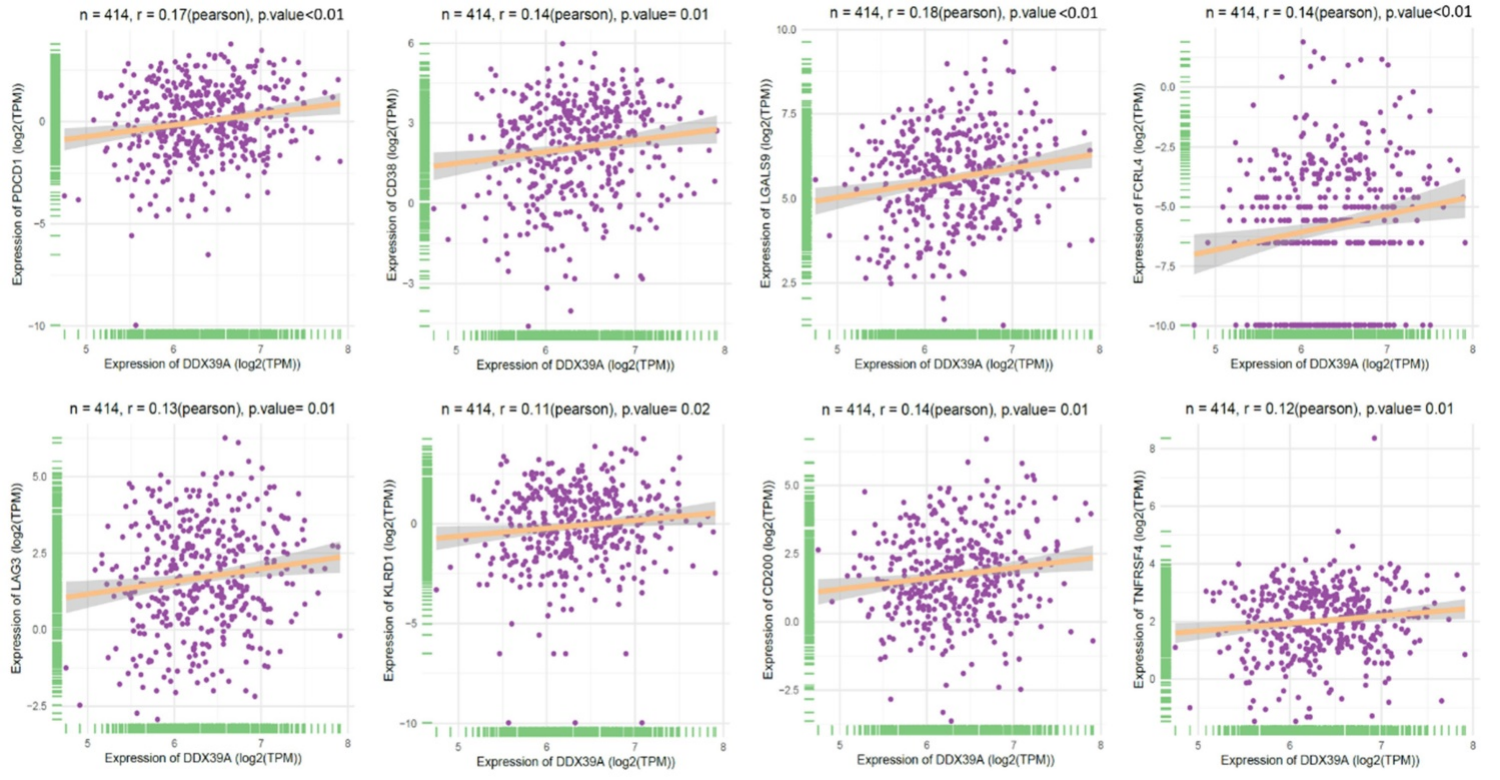
Increased DDX39 expression associates with higher inhibitory immune genes (PD-1, CD38, LGALS9, FCRL4, LAG3, KLRD1, CD200 and TNFRSF4) in ccRCC patients.

To further explore the role of DDX39 as a predictive factor for the patient outcome of immune check-point therapy, we conducted Spearman's correlation analysis and TIDE analysis in 531 ccRCC TCGA cohort. It was found that a higher DDX39 expression was positively associated with inhibitory or coinhibitory immune genes PDCD1 (PD-1), CD38, LGALS9, LAG3, KLRD1 and CD200 (**Figure 9**), suggesting that high DDX39 expression may predict worse immune function in ccRCC patients.

**Figure 10:**
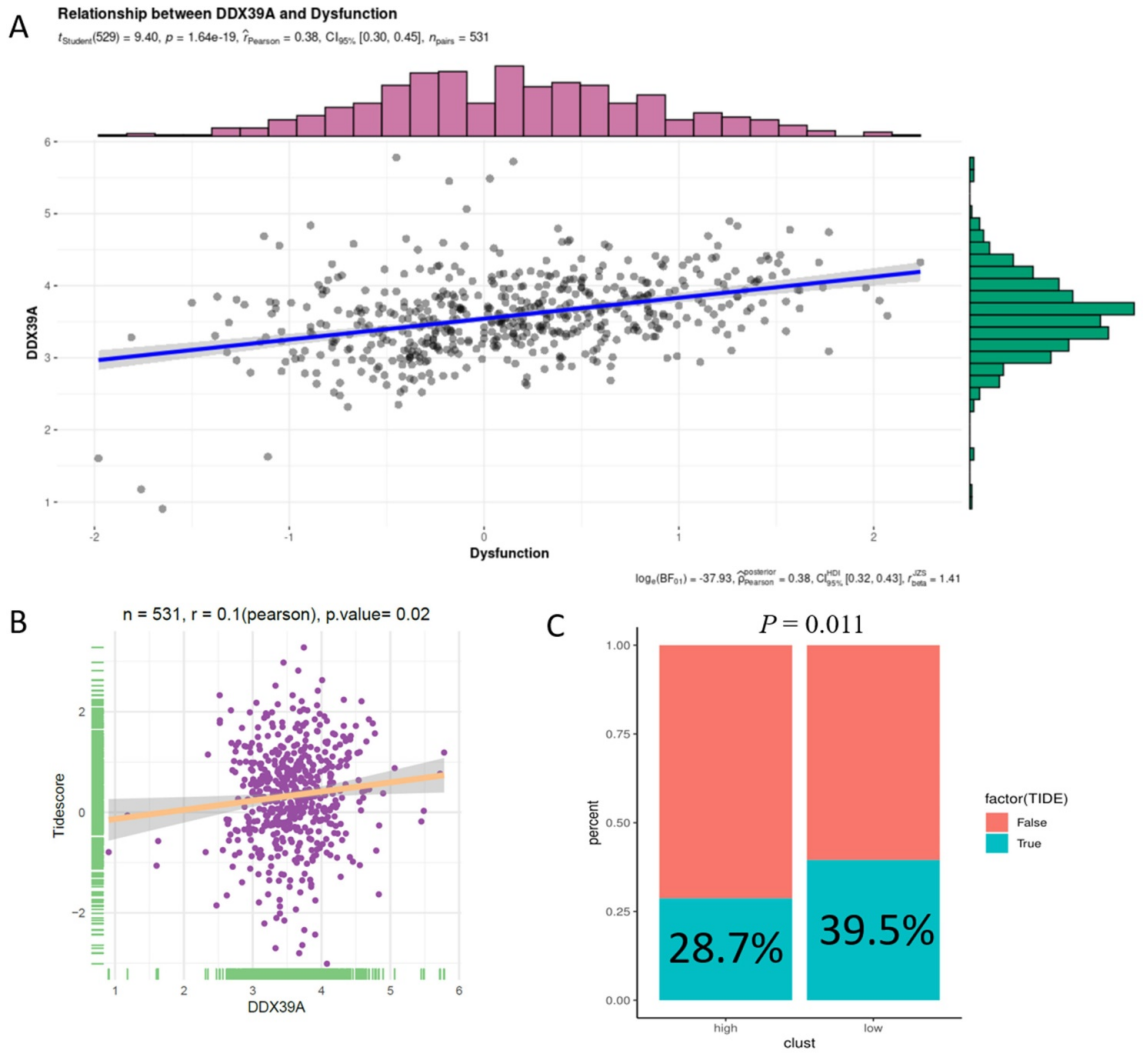
** Increased DDX39 is related to higher immune dysfunction scores and worse immune check-point therapy efficacy in ccRCC patients. A.** Pearson's correlation analysis between DDX39 and immune dysfunction score in TCGA ccRCC patients, n=531, r_Pearson_=0.38, *p*<0.0001. **B.** Pearson's correlation analysis between DDX39 and Tidescore in TCGA ccRCC patients, n=531, r_Pearson_=0.1, *p*=0.02. **C.** Immune checkpoint therapy response rates in TCGA ccRCC patients with high and low DDX39 expression in TIDE analysis, *p*=0.011.

In addition, increased DDX39 expression was related to a higher immune dysfunction score (r=0.38,* p*<0.0001) (**Figure 10A**) and TIDE score (r=0.1, *p*=0.02) (**Figure 10B**). The results of all 14 scores were uploaded in**Table S10**. In consistency with that, high DDX39 expression predicted an adverse response of ccRCC patients to immune check-point therapy (28.7% *vs.* 39.5%, *p*=0.011) (**Figure 10C**).

## Discussion

The DEAD-box protein family contains broad categories of RNA helicases. DDX39 located in the cell nucleus has been revealed to be highly expressed in tumors and correlated with the patient outcome. However, the role of DDX39 in ccRCC remains to be investigated. It was found in this study that high DDX39 expression predicted an adverse outcome of ccRCC patients and played a role in promoting ccRCC cell proliferation, migration and invasion in vitro. GO analysis showed that DDX39 was involved in splicing and metabolic processes of ncRNA, rRNA and tRNA in ccRCC progression. Meanwhile, DDX39 was found to be correlated with hallmarks (such as MYC, G2M, E2F, TGF-β and TNF-α) that participate in cancer activation and cycle acceleration. Ample research has demonstrated that proteins from the DEAD-box protein family can enhance heterotypic hyperplasia and vicious transformation of cancer cells 16. Abnormal or enhanced translation is deemed to be the molecular foundation of neoplastic transformation. Sugiura et al (13) reported that the complex formed from the combination of DDX39 and CIP29 (a proliferation and apoptosis regulating protein) could regulate cell cycle by binding with RNA-binding protein FUS/TLS. DDX39 may promote the transformation from normal cells to tumor cells by increasing gene translation through RNA splicing stimulation 17. DDX39 may play an oncogenic role by interacting with other splicing factors such as SF2/ASF 18. Wnt/β-catenin pathway was identified as a critical mechanism stimulated by DDX39 in promoting cancer growth and metastasis 19. Further studies remain to be done to clarify the detailed mechanism of DDX39 in promoting ccRCC progression and metastasis.

Interestingly, DDX39 was also found to be related to Estimate scores and immunotherapy-predicting pathways in our study. ccRCC is characterized by a high level of immune infiltration 20. The TME infiltrated by different types of adaptive and innate immune cells shapes a critical ecology for all sides of tumor progression 21. To be noted, T cells consist of more than half immune cell types in the ccRCC TME, of which about 25% are double-positive for CD4^+^ and CD8^+^ 22, 23. It was found in our study that DDX39 expression was correlated with different categories of T cells including activated CD4^+^T, central memory CD4^+^ T, activated CD8^+^T, effector memory CD8^+^ T, nature killer T, type 1 Th, T follicular helper and regulatory T cells. Besides, DXX39 was also related to myeloid derived suppressor cells (MDSCs), activated B cells, macrophages and monocytes. These results elucidate that DDX39 was highly associated with most kinds of T cells, especially with CD4^+^T cells. Besides, we only enriched major MHC I-related pathways usually related to antigen presentation. GSEA analysis based on the most associated 60 immunity-related genes showed that the most enriched pathways were T cell receptor signaling, antigen receptor-mediated pathway, antigen processing and presentation pathways. Taken together, we assume that DDX39 mainly functions as a regulatory factor involved in antigen processing and the presentation course in ccRCC TME, but we still need more evidence to affirm its role in the anti-tumor process.

Although ccRCC is usually deemed as a immunogenic tumor, it is also notorious for its immune dysfunction through regulatory T cells and MDSCs by upregulating a variety of inhibitory makers 24. To further understand the role of DDX39 in ccRCC tumor immunity, we performed Pearson's correlation analysis to determine the correlation of DDX39 expression with a series of immune cell markers, and found that DXX39 was associated with a variety of coinhibitory immune receptor markers in ccRCC patients, including PD-1, CD38, LAG3, LGALS9, KLRD1, TNFRSF4, FCRL4 and CD200. Among them, PD-1 is the receptor expressing on T cell surface in PD-1 check-point pathway and is encoded by human PDCD1 gene 25, 26. As a transmembrane receptor, its intracellular part is sited in inhibitory motif which directly receives negative TCR signals that suppress T cell activities, and therefore CD8^+^ T cell exhaustion comes with overexpression of PD-1 27, 28. Tumor cells can escape T cell-mediated tumor-specific immune attacks by expressing PDL-1/2 ligands binding PD-1 receptor. In depleted immune cells, the expression level of PD-1 along with other inhibitory receptors induced during T cell activation continue to increase, causing severe loss of function in the response of T cells to activation signals 29. Importantly, T cell population with high PD-1 expression is usually accompanied with elevated coinhibitory markers or activation markers, including CD38 which has been identified as a T cell exhaustion marker in ccRCC 23. In addition, Lymphocyte activation gene 3 protein (LAG3/CD223) is a T cell inhibitory receptor which can transfer suppressive signals cooperatively with PDCD1/PD-1 30. Besides, LAG3 functions as a negative signal receiver for both Tregs and dendritic cells (DCs). Next, Galectin 9 (LGALS9) in stromal cells induced by IFN-γ or TNF-α released from peripheral blood mononuclear cells (PBMCs) can exert its suppressive function on T cells 31. LGALS9 also functions as an immunosuppressor through many other ways. By binding CD44 receptor on regulatory T cells (Tregs), LGALS9 promotes the immunosuppressive function of Tregs through SMAD3-FOXP3 pathway 32. KLRD1 in complex with its paralogs acts as a critical suppressor on nature killer cells (NKs) and tumor-specific T cells 33, 34. KLRD1 expressing on CD8^+^ γδ Tregs could impose restrictions on the cytotoxicity of CD8^+^ αβ T cells 35. In certain context, KLRD1 as an inhibitory check-point could even dedicate to continuous effector immune cell exhaustion by recruiting INPP/SHIP-1 tyrosine phosphatases to its inhibition motifs, and finally suppress activation signals 36, 37. But TNFRSF4 could also function as a costimulatory receptor for CD4^+^T and CD8^+^T cells 38. What is discussed above illustrates that DDX39 is T cell exhaustion-related, and related to other immune cell inhibition as well. Numerous studies have shown that CD200(OX20)-CD200R1 axis can inhibit pro-inflammatory downstream pathways of myeloid cells including macrophages in a variety of tissues 39, 40, and Fc receptor such as FCRL4/CD307d may block B-cell signaling 41. In a word, DDX39 plays a suppressive role in anti-tumor immunity in ccRCC.

PD-1, programmed cell death receptor ligand-1 (PD-L1), and cytotoxic T lymphocyte-associated molecule-4(CTLA-4) are the most widely used immune check point blockades. However, it is usually difficult to evaluate the response rate to these blockades 42. Recent analyses of TCGA and PRECOG databases 43, 44 reported the impact of tumor infiltration level of different immune cell types on OS of ccRCC patients. To predict tumor responses to immunosuppressive therapy, it is necessary to understand how tumors evade the immune system. Therefore, analysis of the published tumor genetic molecular map may still become a valuable method to find prognostic indicators of immunosuppressive therapy even in the absence of exact data concerning the immunosuppressive therapy. Some recent studies have revealed two different mechanisms of tumor immune evasion: some tumors are often in a state of dysfunction, although cytotoxic T cells have a high degree of infiltration, while in other tumors, immunosuppressive factors can remove T cells that have infiltrated into the tumor tissue. Therefore, Peng et al (16) designed a new computing framework using the TIDE score to integrate these two tumor immune escape mechanisms. Their TIDE analysis included 189 human tumor studies involving 33197 samples. The results that they obtained are believed to be able to replace a single biomarker to effectively predict the effect of immunosuppressive therapy. It was found in our study that DDX39 overexpression was positively correlated with the immune dysfunction score and TIDE score, and the response rate to immune checkpoint therapy was compromised by 10.8% in DDX39 high expression group. This probably indicates that ccRCC patients with DDX39 high expression can benefit less from the existing immune blockade therapies. Other than the existing immune check point blockade drugs, some other check points including inhibitory checkpoint LAG3 and TNFRSF4/OX40 related to DDX39 are under investigation 42. It is our hope that we could find new blockades in ccRCC patients with high expression of DDX39 in our future study.

In conclusion, we demonstrated that high expression of DDX39 predicted an adverse outcome of ccRCC patients both in TCGA and Changhai cohorts. In addition, DDX39 was found to be positively correlated with proto-oncogene MYC activation and cancer cell cycle acceleration in bioinformatic analysis, and with immune cell infiltration and the antigen presentation process as well. DDX39 was also proved to promote ccRCC proliferation, migration and invasion in vitro. Besides, DXX39 was proved to be associated with Estimatescores and immunotherapy predicting pathways in ccRCC. Finally, we revealed a positive correlation between DDX39 and inhibitory immune genes, and its value in predicting the efficacy of immune check-point therapy.

## Supplementary Material

Supplementary figures and tables.

## Figures and Tables

**Table 1:** Univariate and multivariate analysis of the correlation between DDX39 expression and overall survival in TCGA ccRCC patients

Parameter	Univariate analysis			Multivariate anlalysis		
	HR	95%CI	*P* value	HR	95%CI	*P* value
**age**	1.03	1.016-1.042	<0.0001*	1.03	1.01-1.05	0.001947
**gender**	1.1	0.78-1.5	0.69	0.8	0.51-1.25	0.318082
**stage**	1.9	1.7-2.2	<0.0001*	1.34	0.78-2.30	0.285213
**grade**	2.3	1.9-2.8	<0.0001*	1.28	0.91-1.81	0.161255
**T**	1.9	1.6-2.3	<0.0001*	1.01	0.61-1.67	0.979953
**N**	3.5	1.9-6.6	<0.0001*	1.08	0.48-2.41	0.850012
**M**	4.3	3.1-5.8	<0.0001*	1.98	0.87-4.451	0.104414
**DDX39**	2.4	1.8-3.2	<0.0001*	2.25	1.36-3.37	0.00165*

*P value <0.05 is considered statistically significant.

**Table 2:** Correlation between DDX39 and clinicopathological features in Changhai ccRCC cohort

Variables	Low DDX39 (n=93)	High DDX39 (n=93)	*P* value
**Gender**			0.351
male	59	65	
female	34	28	
**Age**			0.358
≤ 60	57	63	
>60	36	30	
**Tumor size, cm**			0.007*
≤ 4	38	21	
>4	55	72	
**Fuhrman grade**			0.399
I/II	72	67	
III/IV	21	26	
**TNM stage**			0.011*
I/II	72	56	
III/IV	21	37	
**Cancer embolus**			0.137
yes	6	12	
no	87	81	
**Metastasis**			0.001*
yes	7	23	
no	86	70	

*P value <0.05 is considered statistically significant.

**Table 3:** Univariate and multivariate analysis for overall survival of ccRCC patients in Changhai cohort

Variable	Overall Survival			
	Univariate	Multivariate		
	*P* value	HR	95% CI	*P* value
DDX39 expression				
**low vs. high**	0.007*	0.564	0.318-0.999	0.049*
Gender				
**Male vs. female**	0.626	0.711	0.410-1.234	0.225
Age				
**≤60 vs. >60 y.o.**	0.080	0.610	0.354-1.052	0.076
Maximum tumor size				
**≤4 vs. >4*cm***	0.052	0.846	0.425-1.686	0.635
Fuhrman grading				
**I/II vs. III/IV**	0.194	1.063	0.594-1.900	0.837
TNM staging				
**I/II vs. III/IV**	<0.001*	0.710	0.312-1.615	0.414
Cancer embolus				
**no vs. yes**	<0.001*	0.528	0.257-1.085	0.082
Metastasis				
**no vs. yes**	<0.001*	0.405	0.190-0.865	0.02*

*P value <0.05 is considered statistically significant.

**Table 4:** Univariate and multivariate analysis for disease-free survival of ccRCC patients in Changhai cohort

Variable	Disease-free Survival			
	Univariate	Multivariate		
	*P* value	HR	95% CI	*P* value
DDX39 expression				
**low vs. high**	0.002*	0.563	0.336-0.945	0.030*
Gender				
**Male vs. female**	0.878	0.797	0.481-1.322	0.380
Age				
**≤60 vs. >60 y.o.**	0.225	0.745	0.450-1.234	0.253
Maximum tumor size				
**≤4 vs. >4*cm***	0.028*	0.796	0.429-1.476	0.468
Fuhrman grading				
**I/II vs. III/IV**	0.760	1.373	0.781-2.414	0.271
TNM staging				
**I/II vs. III/IV**	<0.001*	0.709	0.336-1.496	0.367
Cancer embolus				
**no vs. yes**	<0.001*	0.659	0.327-1.325	0.242
Metastasis				
**no vs. yes**	<0.001	0.385	0.188-0.790	0.009*

*P value <0.05 is considered statistically significant.
