# Supplementary material for: DDX39 as a predictor of clinical prognosis and immune checkpoint therapy efficacy in patients with clear cell renal cell carcinoma
Source: Int J Biol Sci. 2021 Jul 25;17(12):3158–72. doi: 10.7150/ijbs.62553 (PMC8375229; doi:10.7150/ijbs.62553)
Supplement: Supplementary file 1 — Supplementary figures and tables. [file ijbsv17p3158s1.pdf]

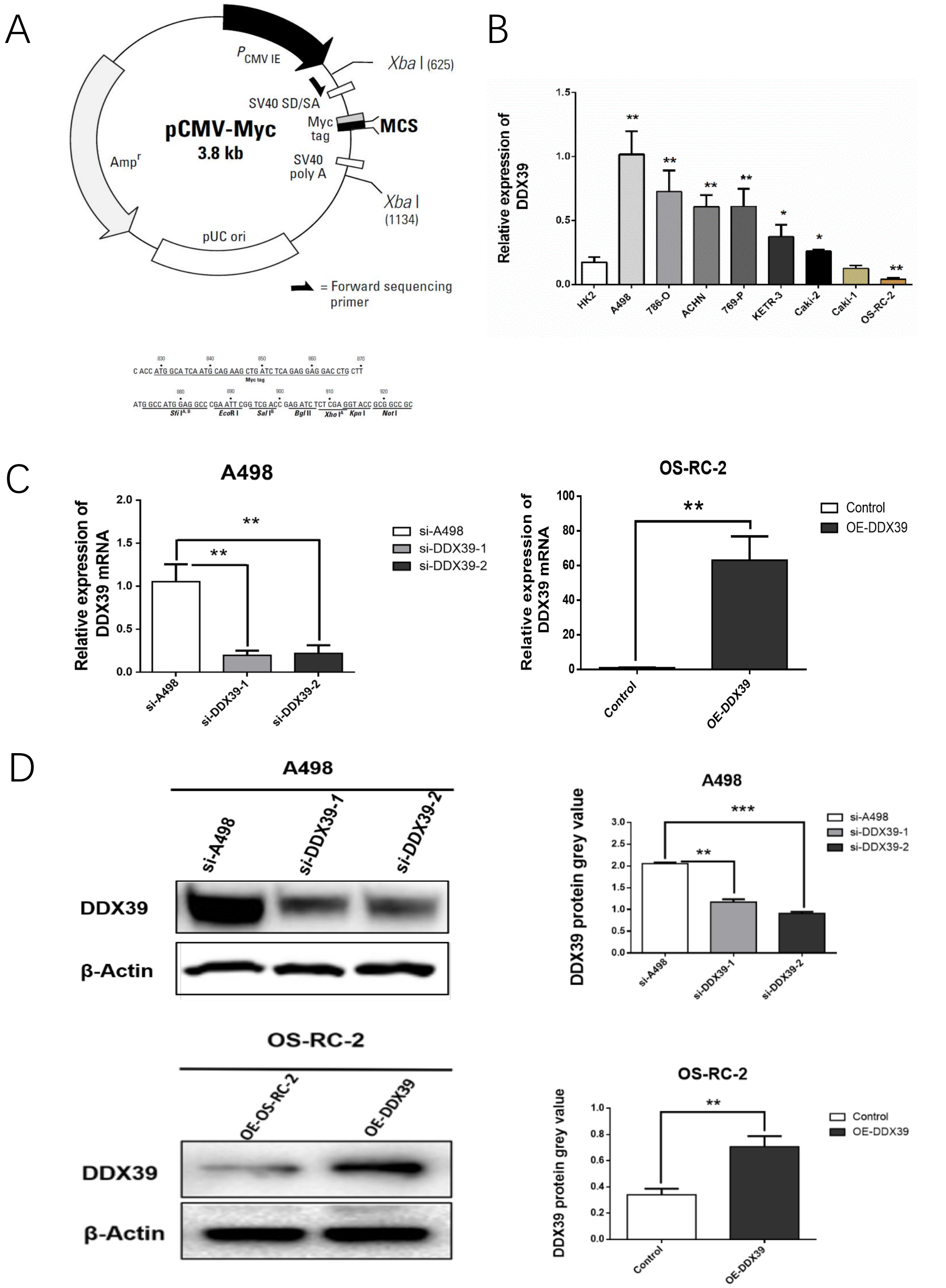

Figure S1. (A) Structure of Myc-DDX39 plasmid vector, **(B)** Comparison of DDX39 mRNA expression in normal renal epithelial cell line (HK-2) and RCC cell lines, $*p < 0.05$, $**p<0.01$, **(C)** DDX39 mRNA expression after transfection in A498 and OS-RC-2, $**p < 0.01$, **(D)** Western blot analysis of DDX39 protein expression in A498 and OS-RC-2 cell line after transfection. Left: WB blot; Right: the gray value of the blot, $**p < 0.01$, $***P<0.001$.

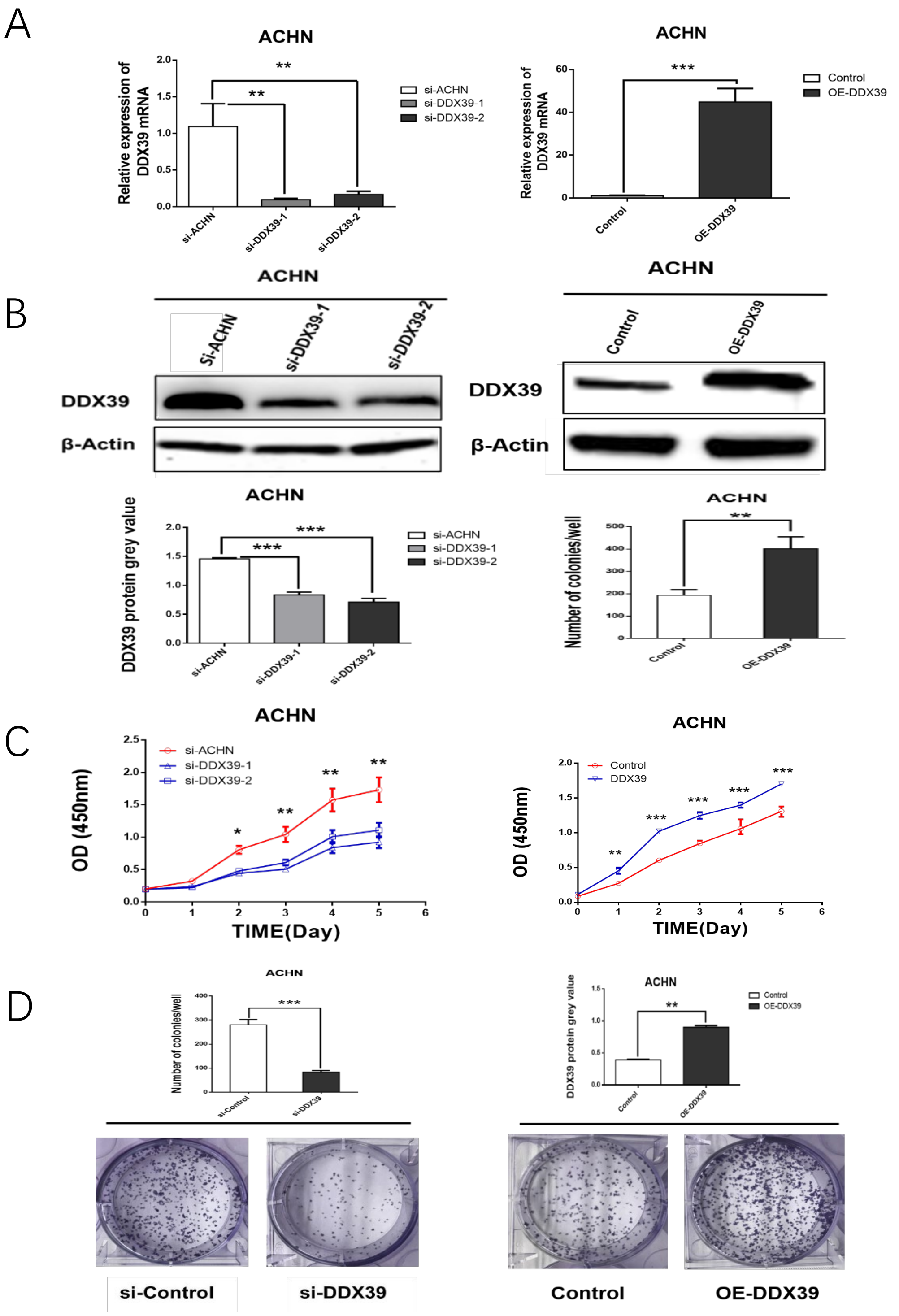

Figure S2. (A) DDX39 mRNA expression after transfection in ACHN cell line, $**p < 0.01$, $***p < 0.001$, **(B)** Western blot analysis of DDX39 protein expression in ACHN cell line after transfection. Up: WB blot; Low: the gray value of the blot, $**p<0.01$, $***p < 0.001$, **(C)** Cell proliferation of ACHN after knock-down and overexpression of DDX39, bars indicated SD. $**p<0.01$, $***p<0.001$.**(D)** Cell colonies of ACHN after transfection, $**p<0.01$, $***P<0.001$.

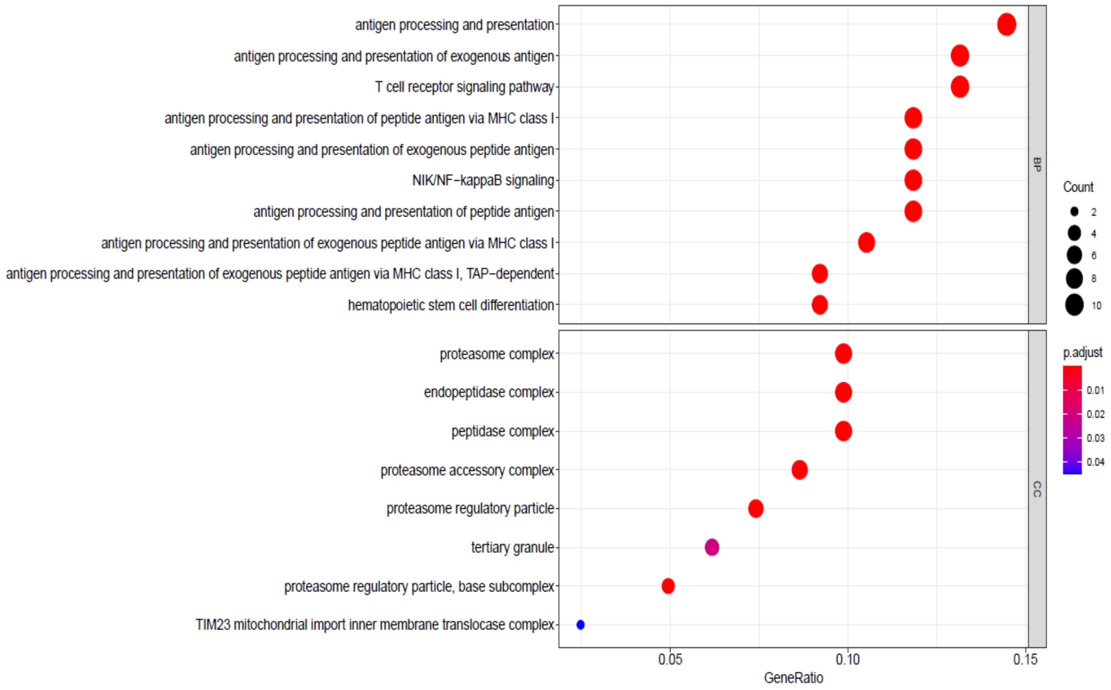

Figure S3. Gene Ontology (GO) analysis of 83 most related immunity genes in ccRCC patients

Table S1. Sequences of primers used for RT-PCR in this study

Gene	Sequence
DDX39	Forward： 5′- CTTCCGCCTGACACCACA-3′
	Reverse： 5′- TCGTCCACAAACACCTCCA -3′
GAPDH	Forward, 5′-GGTCTCCTCTGACTTCAACA-3′
	Reverse, 5′- GTGAGGGTCTCTCTCTTCCT -3′

Table S2. Sequences of primers used for siRNAs used in this study

Gene	Sequence
si-DDX39-1	Forward： 5'-GCAGCAGUACUACGUCAAATT-3'
	Reverse： 5'-UUUGACGUAGUACUGCUGCTT-3'
si-DDX39-2	Forward： 5'-GCGAGUCAACAUCGUCUUUTT-3'
	Reverse： 5'-AAAGACGAUGUUGACUCGCTT-3'
Negative control	Forward： 5'-UUCUCCGAACGUGUCACGUTT-3'
	Reverse： 5'-ACGUGACACGUUCGGAGAATT-3'

Table S3: Markers of 28 immune cell types

Metagene	Cell type	Immunity
ADAM28	Activated B cell	Adaptive
CD180	Activated B cell	Adaptive
CD79B	Activated B cell	Adaptive
BLK	Activated B cell	Adaptive
CD19	Activated B cell	Adaptive
MS4A1	Activated B cell	Adaptive
TNFRSF17	Activated B cell	Adaptive
IGHM	Activated B cell	Adaptive
GNG7	Activated B cell	Adaptive
MICAL3	Activated B cell	Adaptive
SPIB	Activated B cell	Adaptive
HLA-DOB	Activated B cell	Adaptive
IGKC	Activated B cell	Adaptive
PNOC	Activated B cell	Adaptive
FCRL2	Activated B cell	Adaptive
BACH2	Activated B cell	Adaptive
CR2	Activated B cell	Adaptive
TCL1A	Activated B cell	Adaptive
AKNA	Activated B cell	Adaptive
ARHGAP25	Activated B cell	Adaptive
CCL21	Activated B cell	Adaptive
CD27	Activated B cell	Adaptive
CD38	Activated B cell	Adaptive
CLEC17A	Activated B cell	Adaptive
CLEC9A	Activated B cell	Adaptive
CLECL1	Activated B cell	Adaptive
AIM2	Activated CD4 T cell	Adaptive
BIRC3	Activated CD4 T cell	Adaptive
BRIP1	Activated CD4 T cell	Adaptive
CCL20	Activated CD4 T cell	Adaptive
CCL4	Activated CD4 T cell	Adaptive
CCL5	Activated CD4 T cell	Adaptive
CCNB1	Activated CD4 T cell	Adaptive
CCR7	Activated CD4 T cell	Adaptive
DUSP2	Activated CD4 T cell	Adaptive
ESCO2	Activated CD4 T cell	Adaptive
ETS1	Activated CD4 T cell	Adaptive
EXO1	Activated CD4 T cell	Adaptive
EXOC6	Activated CD4 T cell	Adaptive
IARS	Activated CD4 T cell	Adaptive
ITK	Activated CD4 T cell	Adaptive
KIF11	Activated CD4 T cell	Adaptive
KNTC1	Activated CD4 T cell	Adaptive
NUF2	Activated CD4 T cell	Adaptive
PRC1	Activated CD4 T cell	Adaptive
PSAT1	Activated CD4 T cell	Adaptive
RGS1	Activated CD4 T cell	Adaptive
RTKN2	Activated CD4 T cell	Adaptive
SAMSN1	Activated CD4 T cell	Adaptive
SELL	Activated CD4 T cell	Adaptive
TRAT1	Activated CD4 T cell	Adaptive
ADRM1	Activated CD8 T cell	Adaptive
AHSA1	Activated CD8 T cell	Adaptive
C1GALT1C1	Activated CD8 T cell	Adaptive
CCT6B	Activated CD8 T cell	Adaptive
CD37	Activated CD8 T cell	Adaptive

CD3D	Activated CD8 T cell	Adaptive
CD3E	Activated CD8 T cell	Adaptive
CD3G	Activated CD8 T cell	Adaptive
CD69	Activated CD8 T cell	Adaptive
CD8A	Activated CD8 T cell	Adaptive
CETN3	Activated CD8 T cell	Adaptive
CSE1L	Activated CD8 T cell	Adaptive
GEMIN6	Activated CD8 T cell	Adaptive
GNLY	Activated CD8 T cell	Adaptive
GPT2	Activated CD8 T cell	Adaptive
GZMA	Activated CD8 T cell	Adaptive
GZMH	Activated CD8 T cell	Adaptive
GZMK	Activated CD8 T cell	Adaptive
IL2RB	Activated CD8 T cell	Adaptive
LCK	Activated CD8 T cell	Adaptive
MPZL1	Activated CD8 T cell	Adaptive
NKG7	Activated CD8 T cell	Adaptive
PIK3IP1	Activated CD8 T cell	Adaptive
PTRH2	Activated CD8 T cell	Adaptive
TIMM13	Activated CD8 T cell	Adaptive
ZAP70	Activated CD8 T cell	Adaptive
ABHD3	Central memory CD4 T cell	Adaptive
AHNAK	Central memory CD4 T cell	Adaptive
ANXA2P2	Central memory CD4 T cell	Adaptive
AQP3	Central memory CD4 T cell	Adaptive
ATHL1	Central memory CD4 T cell	Adaptive
BMI1	Central memory CD4 T cell	Adaptive
BZW2	Central memory CD4 T cell	Adaptive
CD63	Central memory CD4 T cell	Adaptive
COL4A1	Central memory CD4 T cell	Adaptive
CYLD	Central memory CD4 T cell	Adaptive
ELMO2	Central memory CD4 T cell	Adaptive
FYN	Central memory CD4 T cell	Adaptive
GLIPR1	Central memory CD4 T cell	Adaptive
GSS	Central memory CD4 T cell	Adaptive
IFITM2	Central memory CD4 T cell	Adaptive
ITGB1	Central memory CD4 T cell	Adaptive
ITGB2	Central memory CD4 T cell	Adaptive
KLF5	Central memory CD4 T cell	Adaptive
LSP1	Central memory CD4 T cell	Adaptive
NDUFB9	Central memory CD4 T cell	Adaptive
PKM2	Central memory CD4 T cell	Adaptive
SFXN3	Central memory CD4 T cell	Adaptive
SIRPG	Central memory CD4 T cell	Adaptive
SMAD4	Central memory CD4 T cell	Adaptive
STX4	Central memory CD4 T cell	Adaptive
TRADD	Central memory CD4 T cell	Adaptive
VIM	Central memory CD4 T cell	Adaptive
XRCC6	Central memory CD4 T cell	Adaptive
ACTN4	Central memory CD8 T cell	Adaptive
ADAM12	Central memory CD8 T cell	Adaptive
ADCY9	Central memory CD8 T cell	Adaptive
F13A1	Central memory CD8 T cell	Adaptive
FCER1G	Central memory CD8 T cell	Adaptive
FCGR3B	Central memory CD8 T cell	Adaptive
FGF7	Central memory CD8 T cell	Adaptive
FKBP4	Central memory CD8 T cell	Adaptive
GLUD1	Central memory CD8 T cell	Adaptive

GM2A	Central memory CD8 T cell	Adaptive
GUSB	Central memory CD8 T cell	Adaptive
IL1RN	Central memory CD8 T cell	Adaptive
NOL11	Central memory CD8 T cell	Adaptive
NTRK1	Central memory CD8 T cell	Adaptive
RARA	Central memory CD8 T cell	Adaptive
RNF128	Central memory CD8 T cell	Adaptive
SIGLEC1	Central memory CD8 T cell	Adaptive
TNFRSF11A	Central memory CD8 T cell	Adaptive
TOX4	Central memory CD8 T cell	Adaptive
UBA52	Central memory CD8 T cell	Adaptive
ULBP1	Central memory CD8 T cell	Adaptive
ATM	Effector memeory CD4 T cell	Adaptive
CASP3	Effector memeory CD4 T cell	Adaptive
CASQ1	Effector memeory CD4 T cell	Adaptive
CD300E	Effector memeory CD4 T cell	Adaptive
DARS	Effector memeory CD4 T cell	Adaptive
DOCK9	Effector memeory CD4 T cell	Adaptive
EXOSC9	Effector memeory CD4 T cell	Adaptive
EZH2	Effector memeory CD4 T cell	Adaptive
GDE1	Effector memeory CD4 T cell	Adaptive
IL34	Effector memeory CD4 T cell	Adaptive
NCOA4	Effector memeory CD4 T cell	Adaptive
NEFL	Effector memeory CD4 T cell	Adaptive
PDGFRL	Effector memeory CD4 T cell	Adaptive
PTGS1	Effector memeory CD4 T cell	Adaptive
REPS1	Effector memeory CD4 T cell	Adaptive
SCG2	Effector memeory CD4 T cell	Adaptive
SDPR	Effector memeory CD4 T cell	Adaptive
SIGLEC14	Effector memeory CD4 T cell	Adaptive
SIGLEC6	Effector memeory CD4 T cell	Adaptive
TAL1	Effector memeory CD4 T cell	Adaptive
TFEC	Effector memeory CD4 T cell	Adaptive
TIPIN	Effector memeory CD4 T cell	Adaptive
TPK1	Effector memeory CD4 T cell	Adaptive
UQCRB	Effector memeory CD4 T cell	Adaptive
USP9Y	Effector memeory CD4 T cell	Adaptive
WIPF1	Effector memeory CD4 T cell	Adaptive
ZCRB1	Effector memeory CD4 T cell	Adaptive
ACAP1	Effector memeory CD8 T cell	Adaptive
APOL3	Effector memeory CD8 T cell	Adaptive
ARHGAP10	Effector memeory CD8 T cell	Adaptive
ATP10D	Effector memeory CD8 T cell	Adaptive
C3AR1	Effector memeory CD8 T cell	Adaptive
CCR5	Effector memeory CD8 T cell	Adaptive
CD160	Effector memeory CD8 T cell	Adaptive
CD55	Effector memeory CD8 T cell	Adaptive
CFLAR	Effector memeory CD8 T cell	Adaptive
CMKLR1	Effector memeory CD8 T cell	Adaptive
DAPP1	Effector memeory CD8 T cell	Adaptive
FCRL6	Effector memeory CD8 T cell	Adaptive
FLT3LG	Effector memeory CD8 T cell	Adaptive
GZMM	Effector memeory CD8 T cell	Adaptive
HAPLN3	Effector memeory CD8 T cell	Adaptive
HLA-DMB	Effector memeory CD8 T cell	Adaptive
HLA-DPA1	Effector memeory CD8 T cell	Adaptive
HLA-DPB1	Effector memeory CD8 T cell	Adaptive
IFI16	Effector memeory CD8 T cell	Adaptive

LIME1	Effector memeory CD8 T cell	Adaptive
LTK	Effector memeory CD8 T cell	Adaptive
NFKBIA	Effector memeory CD8 T cell	Adaptive
SETD7	Effector memeory CD8 T cell	Adaptive
SIK1	Effector memeory CD8 T cell	Adaptive
TRIB2	Effector memeory CD8 T cell	Adaptive
ACP5	Gamma delta T cell	Adaptive
AQP9	Gamma delta T cell	Adaptive
BTN3A2	Gamma delta T cell	Adaptive
C1orf54	Gamma delta T cell	Adaptive
CARD8	Gamma delta T cell	Adaptive
CCL18	Gamma delta T cell	Adaptive
CD209	Gamma delta T cell	Adaptive
CD33	Gamma delta T cell	Adaptive
CD36	Gamma delta T cell	Adaptive
CDK5	Gamma delta T cell	Adaptive
IL10RB	Gamma delta T cell	Adaptive
KLRF1	Gamma delta T cell	Adaptive
LGALS1	Gamma delta T cell	Adaptive
MAPK7	Gamma delta T cell	Adaptive
KLHL7	Gamma delta T cell	Adaptive
KRT80	Gamma delta T cell	Adaptive
LAMC1	Gamma delta T cell	Adaptive
LCORL	Gamma delta T cell	Adaptive
LMNB1	Gamma delta T cell	Adaptive
MEIS3P1	Gamma delta T cell	Adaptive
MPL	Gamma delta T cell	Adaptive
FABP1	Gamma delta T cell	Adaptive
FABP5	Gamma delta T cell	Adaptive
FADD	Gamma delta T cell	Adaptive
MFAP3L	Gamma delta T cell	Adaptive
MINPP1	Gamma delta T cell	Adaptive
RPS24	Gamma delta T cell	Adaptive
RPS7	Gamma delta T cell	Adaptive
RPS9	Gamma delta T cell	Adaptive
DBNL	Gamma delta T cell	Adaptive
CCL13	Gamma delta T cell	Adaptive
CD22	Immature B cell	Adaptive
CYBB	Immature B cell	Adaptive
FAM129C	Immature B cell	Adaptive
FCRL1	Immature B cell	Adaptive
FCRL3	Immature B cell	Adaptive
FCRL5	Immature B cell	Adaptive
FCRLA	Immature B cell	Adaptive
HDAC9	Immature B cell	Adaptive
HLA-DQA1	Immature B cell	Adaptive
HVCN1	Immature B cell	Adaptive
KIAA0226	Immature B cell	Adaptive
NCF1	Immature B cell	Adaptive
NCF1B	Immature B cell	Adaptive
P2RY10	Immature B cell	Adaptive
SP100	Immature B cell	Adaptive
TXNIP	Immature B cell	Adaptive
STAP1	Immature B cell	Adaptive
TAGAP	Immature B cell	Adaptive
ZCCHC2	Immature B cell	Adaptive
AICDA	Memory B cell	Adaptive
CCNA2	Memory B cell	Adaptive

CDKN3	Memory B cell	Adaptive
CLCN5	Memory B cell	Adaptive
ENPP1	Memory B cell	Adaptive
FCER1A	Memory B cell	Adaptive
FCRL4	Memory B cell	Adaptive
MYC	Memory B cell	Adaptive
RUNX2	Memory B cell	Adaptive
SORL1	Memory B cell	Adaptive
SOX5	Memory B cell	Adaptive
STAT5A	Memory B cell	Adaptive
STAT5B	Memory B cell	Adaptive
TLR9	Memory B cell	Adaptive
CCL3L1	Regulatory T cell	Adaptive
CD72	Regulatory T cell	Adaptive
CLEC5A	Regulatory T cell	Adaptive
FOXP3	Regulatory T cell	Adaptive
ITGA4	Regulatory T cell	Adaptive
L1CAM	Regulatory T cell	Adaptive
LIPA	Regulatory T cell	Adaptive
LRP1	Regulatory T cell	Adaptive
LRRC42	Regulatory T cell	Adaptive
MARCO	Regulatory T cell	Adaptive
MMP12	Regulatory T cell	Adaptive
MNDA	Regulatory T cell	Adaptive
MRC1	Regulatory T cell	Adaptive
MS4A6A	Regulatory T cell	Adaptive
PELO	Regulatory T cell	Adaptive
PLEK	Regulatory T cell	Adaptive
PRSS23	Regulatory T cell	Adaptive
PTGIR	Regulatory T cell	Adaptive
ST8SIA4	Regulatory T cell	Adaptive
STAB1	Regulatory T cell	Adaptive
B3GAT1	T follicular helper cell	Adaptive
CDK5R1	T follicular helper cell	Adaptive
PDCD1	T follicular helper cell	Adaptive
BCL6	T follicular helper cell	Adaptive
CD200	T follicular helper cell	Adaptive
CD83	T follicular helper cell	Adaptive
CD84	T follicular helper cell	Adaptive
FGF2	T follicular helper cell	Adaptive
GPR18	T follicular helper cell	Adaptive
CEBPA	T follicular helper cell	Adaptive
CECR1	T follicular helper cell	Adaptive
CLEC10A	T follicular helper cell	Adaptive
CLEC4A	T follicular helper cell	Adaptive
CSF1R	T follicular helper cell	Adaptive
CTSS	T follicular helper cell	Adaptive
DMN	T follicular helper cell	Adaptive
DPP4	T follicular helper cell	Adaptive
LRRC32	T follicular helper cell	Adaptive
MC5R	T follicular helper cell	Adaptive
MICA	T follicular helper cell	Adaptive
NCAM1	T follicular helper cell	Adaptive
NCR2	T follicular helper cell	Adaptive
NRP1	T follicular helper cell	Adaptive
PDCD1LG2	T follicular helper cell	Adaptive
PDCD6	T follicular helper cell	Adaptive
PRDX1	T follicular helper cell	Adaptive

RAE1	T follicular helper cell	Adaptive
RAET1E	T follicular helper cell	Adaptive
SIGLEC7	T follicular helper cell	Adaptive
SIGLEC9	T follicular helper cell	Adaptive
TYRO3	T follicular helper cell	Adaptive
CHST12	T follicular helper cell	Adaptive
CLIC3	T follicular helper cell	Adaptive
IVNS1ABP	T follicular helper cell	Adaptive
KIR2DL2	T follicular helper cell	Adaptive
LGMN	T follicular helper cell	Adaptive
CD70	Type 1 T helper cell	Adaptive
TBX21	Type 1 T helper cell	Adaptive
ADAM8	Type 1 T helper cell	Adaptive
AHCYL2	Type 1 T helper cell	Adaptive
ALCAM	Type 1 T helper cell	Adaptive
B3GALNT1	Type 1 T helper cell	Adaptive
BBS12	Type 1 T helper cell	Adaptive
BST1	Type 1 T helper cell	Adaptive
CD151	Type 1 T helper cell	Adaptive
CD47	Type 1 T helper cell	Adaptive
CD48	Type 1 T helper cell	Adaptive
CD52	Type 1 T helper cell	Adaptive
CD53	Type 1 T helper cell	Adaptive
CD59	Type 1 T helper cell	Adaptive
CD6	Type 1 T helper cell	Adaptive
CD68	Type 1 T helper cell	Adaptive
CD7	Type 1 T helper cell	Adaptive
CD96	Type 1 T helper cell	Adaptive
CFHR3	Type 1 T helper cell	Adaptive
CHRM3	Type 1 T helper cell	Adaptive
CLEC7A	Type 1 T helper cell	Adaptive
COL23A1	Type 1 T helper cell	Adaptive
COL4A4	Type 1 T helper cell	Adaptive
COL5A3	Type 1 T helper cell	Adaptive
DAB1	Type 1 T helper cell	Adaptive
DLEU7	Type 1 T helper cell	Adaptive
DOC2B	Type 1 T helper cell	Adaptive
EMP1	Type 1 T helper cell	Adaptive
F12	Type 1 T helper cell	Adaptive
FURIN	Type 1 T helper cell	Adaptive
GAB3	Type 1 T helper cell	Adaptive
GATM	Type 1 T helper cell	Adaptive
GFPT2	Type 1 T helper cell	Adaptive
GPR25	Type 1 T helper cell	Adaptive
GREM2	Type 1 T helper cell	Adaptive
HAVCR1	Type 1 T helper cell	Adaptive
HSD11B1	Type 1 T helper cell	Adaptive
HUNK	Type 1 T helper cell	Adaptive
IGF2	Type 1 T helper cell	Adaptive
RCSD1	Type 1 T helper cell	Adaptive
RYR1	Type 1 T helper cell	Adaptive
SAV1	Type 1 T helper cell	Adaptive
SELE	Type 1 T helper cell	Adaptive
SELP	Type 1 T helper cell	Adaptive
SH3KBP1	Type 1 T helper cell	Adaptive
SIT1	Type 1 T helper cell	Adaptive
SLC35B3	Type 1 T helper cell	Adaptive
SIGLEC10	Type 1 T helper cell	Adaptive

SKAP1	Type 1 T helper cell	Adaptive
THUMPD2	Type 1 T helper cell	Adaptive
TIGIT	Type 1 T helper cell	Adaptive
ZEB2	Type 1 T helper cell	Adaptive
ENC1	Type 1 T helper cell	Adaptive
FAM134B	Type 1 T helper cell	Adaptive
FBXO30	Type 1 T helper cell	Adaptive
FCGR2C	Type 1 T helper cell	Adaptive
STAC	Type 1 T helper cell	Adaptive
LTC4S	Type 1 T helper cell	Adaptive
MAN1B1	Type 1 T helper cell	Adaptive
MDH1	Type 1 T helper cell	Adaptive
MMD	Type 1 T helper cell	Adaptive
RGS16	Type 1 T helper cell	Adaptive
IL12A	Type 1 T helper cell	Adaptive
P2RX5	Type 1 T helper cell	Adaptive
CD97	Type 1 T helper cell	Adaptive
ITGB4	Type 1 T helper cell	Adaptive
ICAM3	Type 1 T helper cell	Adaptive
METRNL	Type 1 T helper cell	Adaptive
TNFRSF1A	Type 1 T helper cell	Adaptive
IRF1	Type 1 T helper cell	Adaptive
HTR2B	Type 1 T helper cell	Adaptive
CALD1	Type 1 T helper cell	Adaptive
MOCOS	Type 1 T helper cell	Adaptive
TRAF3IP2	Type 1 T helper cell	Adaptive
TLR8	Type 1 T helper cell	Adaptive
TRAF1	Type 1 T helper cell	Adaptive
DUSP14	Type 1 T helper cell	Adaptive
IL17A	Type 17 T helper cell	Adaptive
IL17RA	Type 17 T helper cell	Adaptive
C2CD4A	Type 17 T helper cell	Adaptive
C2CD4B	Type 17 T helper cell	Adaptive
CA2	Type 17 T helper cell	Adaptive
CCDC65	Type 17 T helper cell	Adaptive
CEACAM3	Type 17 T helper cell	Adaptive
IL17C	Type 17 T helper cell	Adaptive
IL17F	Type 17 T helper cell	Adaptive
IL17RC	Type 17 T helper cell	Adaptive
IL17RE	Type 17 T helper cell	Adaptive
IL23A	Type 17 T helper cell	Adaptive
ILDR1	Type 17 T helper cell	Adaptive
LONRF3	Type 17 T helper cell	Adaptive
SH2D6	Type 17 T helper cell	Adaptive
TNIP2	Type 17 T helper cell	Adaptive
ABCA1	Type 17 T helper cell	Adaptive
ABCB1	Type 17 T helper cell	Adaptive
ADAMTS12	Type 17 T helper cell	Adaptive
ANK1	Type 17 T helper cell	Adaptive
ANKRD22	Type 17 T helper cell	Adaptive
B3GALT2	Type 17 T helper cell	Adaptive
CAMTA1	Type 17 T helper cell	Adaptive
CCR9	Type 17 T helper cell	Adaptive
CD40	Type 17 T helper cell	Adaptive
GPR44	Type 17 T helper cell	Adaptive
IFT80	Type 17 T helper cell	Adaptive
ASB2	Type 2 T helper cell	Adaptive
CSRP2	Type 2 T helper cell	Adaptive

DAPK1	Type 2 T helper cell	Adaptive
DLC1	Type 2 T helper cell	Adaptive
DNAJC12	Type 2 T helper cell	Adaptive
DUSP6	Type 2 T helper cell	Adaptive
GNAI1	Type 2 T helper cell	Adaptive
LAMP3	Type 2 T helper cell	Adaptive
NRP2	Type 2 T helper cell	Adaptive
OSBPL1A	Type 2 T helper cell	Adaptive
PDE4B	Type 2 T helper cell	Adaptive
PHLDA1	Type 2 T helper cell	Adaptive
PLA2G4A	Type 2 T helper cell	Adaptive
RAB27B	Type 2 T helper cell	Adaptive
RBMS3	Type 2 T helper cell	Adaptive
RNF125	Type 2 T helper cell	Adaptive
TMPRSS3	Type 2 T helper cell	Adaptive
GATA3	Type 2 T helper cell	Adaptive
BIRC5	Type 2 T helper cell	Adaptive
CDC25C	Type 2 T helper cell	Adaptive
CDC7	Type 2 T helper cell	Adaptive
CENPF	Type 2 T helper cell	Adaptive
CXCR6	Type 2 T helper cell	Adaptive
DHFR	Type 2 T helper cell	Adaptive
EVI5	Type 2 T helper cell	Adaptive
GSTA4	Type 2 T helper cell	Adaptive
HELLS	Type 2 T helper cell	Adaptive
IL26	Type 2 T helper cell	Adaptive
LAIR2	Type 2 T helper cell	Adaptive
ABCD1	Activated dendritic cell	Innate
C1QC	Activated dendritic cell	Innate
CAPG	Activated dendritic cell	Innate
CCL3L3	Activated dendritic cell	Innate
CD207	Activated dendritic cell	Innate
CD302	Activated dendritic cell	Innate
ATP5B	Activated dendritic cell	Innate
ATP5L	Activated dendritic cell	Innate
ATP6V1A	Activated dendritic cell	Innate
BCL2L1	Activated dendritic cell	Innate
C1QB	Activated dendritic cell	Innate
SNURF	Activated dendritic cell	Innate
SPCS3	Activated dendritic cell	Innate
CCNA1	Activated dendritic cell	Innate
CEACAM8	Activated dendritic cell	Innate
NOS2	Activated dendritic cell	Innate
SRA1	Activated dendritic cell	Innate
TNFRSF6B	Activated dendritic cell	Innate
TREM1	Activated dendritic cell	Innate
TREML1	Activated dendritic cell	Innate
RHOA	Activated dendritic cell	Innate
SLC25A37	Activated dendritic cell	Innate
TNFSF14	Activated dendritic cell	Innate
TREML4	Activated dendritic cell	Innate
VNN2	Activated dendritic cell	Innate
XPO6	Activated dendritic cell	Innate
CLEC4C	Activated dendritic cell	Innate
TNFAIP2	Activated dendritic cell	Innate
UBD	Activated dendritic cell	Innate
ACTR3	Activated dendritic cell	Innate
RAB1A	Activated dendritic cell	Innate

SLA	Activated dendritic cell	Innate
HLA-DQA2	Activated dendritic cell	Innate
SIGLEC5	Activated dendritic cell	Innate
SLAMF9	Activated dendritic cell	Innate
ABAT	CD56bright natural killer cell	Innate
C11orf75	CD56bright natural killer cell	Innate
C5orf15	CD56bright natural killer cell	Innate
CDHR1	CD56bright natural killer cell	Innate
DCAF12	CD56bright natural killer cell	Innate
DYNLL1	CD56bright natural killer cell	Innate
GPR137B	CD56bright natural killer cell	Innate
HCP5	CD56bright natural killer cell	Innate
HDGFRP2	CD56bright natural killer cell	Innate
KRT86	CD56bright natural killer cell	Innate
MLST8	CD56bright natural killer cell	Innate
ELMOD3	CD56bright natural killer cell	Innate
ENTPD5	CD56bright natural killer cell	Innate
FAM119A	CD56bright natural killer cell	Innate
FAM179A	CD56bright natural killer cell	Innate
CLIC2	CD56bright natural killer cell	Innate
COX7A2L	CD56bright natural killer cell	Innate
CREB3L4	CD56bright natural killer cell	Innate
CSF1	CD56bright natural killer cell	Innate
CSNK2A2	CD56bright natural killer cell	Innate
CSTA	CD56bright natural killer cell	Innate
CSTB	CD56bright natural killer cell	Innate
CTPS	CD56bright natural killer cell	Innate
CTSD	CD56bright natural killer cell	Innate
FST	CD56bright natural killer cell	Innate
GATA2	CD56bright natural killer cell	Innate
GMPR	CD56bright natural killer cell	Innate
HDC	CD56bright natural killer cell	Innate
HEY1	CD56bright natural killer cell	Innate
HOXA1	CD56bright natural killer cell	Innate
HS2ST1	CD56bright natural killer cell	Innate
HS3ST1	CD56bright natural killer cell	Innate
BCL11B	CD56bright natural killer cell	Innate
CDH3	CD56bright natural killer cell	Innate
MYL6B	CD56bright natural killer cell	Innate
NAA16	CD56bright natural killer cell	Innate
CIQA	CD56bright natural killer cell	Innate
CIQB	CD56bright natural killer cell	Innate
CYP27B1	CD56bright natural killer cell	Innate
EIF3M	CD56bright natural killer cell	Innate
CYP27A1	CD56dim natural killer cell	Innate
DDX55	CD56dim natural killer cell	Innate
DYRK2	CD56dim natural killer cell	Innate
RPL37A	CD56dim natural killer cell	Innate
NOTCH3	CD56dim natural killer cell	Innate
AKR7A3	CD56dim natural killer cell	Innate
GPRC5C	CD56dim natural killer cell	Innate
GRIN1	CD56dim natural killer cell	Innate
HLA-E	CD56dim natural killer cell	Innate
PORCN	CD56dim natural killer cell	Innate
PSMC4	CD56dim natural killer cell	Innate
UPP1	CD56dim natural killer cell	Innate
IL21R	CD56dim natural killer cell	Innate
KIR2DS1	CD56dim natural killer cell	Innate

KIR2DS2	CD56dim natural killer cell	Innate
KIR2DS5	CD56dim natural killer cell	Innate
GIPR	Eosinophil	Innate
KRT18P50	Eosinophil	Innate
LRMP	Eosinophil	Innate
FOSB	Eosinophil	Innate
RRP12	Eosinophil	Innate
GPR183	Eosinophil	Innate
NR4A3	Eosinophil	Innate
ST3GAL6	Eosinophil	Innate
DEPDC5	Eosinophil	Innate
PDE6C	Eosinophil	Innate
PKD2L2	Eosinophil	Innate
GPR65	Eosinophil	Innate
IL5RA	Eosinophil	Innate
P2RY14	Eosinophil	Innate
DACH1	Eosinophil	Innate
DAPK2	Eosinophil	Innate
EMR3	Eosinophil	Innate
ACADM	Immature dendritic cell	Innate
AHCYL1	Immature dendritic cell	Innate
ALDH1A2	Immature dendritic cell	Innate
ALDH3A2	Immature dendritic cell	Innate
ALDH9A1	Immature dendritic cell	Innate
ALOX15	Immature dendritic cell	Innate
AMT	Immature dendritic cell	Innate
ARL1	Immature dendritic cell	Innate
ATIC	Immature dendritic cell	Innate
ATP5A1	Immature dendritic cell	Innate
CAPZA1	Immature dendritic cell	Innate
LILRA5	Immature dendritic cell	Innate
RDX	Immature dendritic cell	Innate
RRAGD	Immature dendritic cell	Innate
TACSTD2	Immature dendritic cell	Innate
INPP5F	Immature dendritic cell	Innate
RAB38	Immature dendritic cell	Innate
PLAU	Immature dendritic cell	Innate
CSF3R	Immature dendritic cell	Innate
SLC18A2	Immature dendritic cell	Innate
AMPD2	Immature dendritic cell	Innate
CLTB	Immature dendritic cell	Innate
C1orf162	Immature dendritic cell	Innate
AIF1	Macrophage	Innate
CCL1	Macrophage	Innate
CCL14	Macrophage	Innate
CCL23	Macrophage	Innate
CCL26	Macrophage	Innate
CD300LB	Macrophage	Innate
CNR1	Macrophage	Innate
CNR2	Macrophage	Innate
EIF1	Macrophage	Innate
EIF4A1	Macrophage	Innate
FPR1	Macrophage	Innate
FPR2	Macrophage	Innate
FRAT2	Macrophage	Innate
GPR27	Macrophage	Innate
GPR77	Macrophage	Innate
RNASE2	Macrophage	Innate

MS4A2	Macrophage	Innate
BASP1	Macrophage	Innate
IGSF6	Macrophage	Innate
HK3	Macrophage	Innate
VNN1	Macrophage	Innate
FES	Macrophage	Innate
NPL	Macrophage	Innate
FZD2	Macrophage	Innate
FAM198B	Macrophage	Innate
HNMT	Macrophage	Innate
SLC15A3	Macrophage	Innate
CD4	Macrophage	Innate
TXNDC3	Macrophage	Innate
FRMD4A	Macrophage	Innate
CRYBB1	Macrophage	Innate
HRH1	Macrophage	Innate
WNT5B	Macrophage	Innate
ADAMTS3	Mast cell	Innate
CPA3	Mast cell	Innate
CMA1	Mast cell	Innate
CTSG	Mast cell	Innate
ARHGAP15	Mast cell	Innate
CPM	Mast cell	Innate
FCN1	Mast cell	Innate
FTL	Mast cell	Innate
HSPA6	Mast cell	Innate
ITGA9	Mast cell	Innate
RNASE3	Mast cell	Innate
S100A4	Mast cell	Innate
SIGLEC8	Mast cell	Innate
SLC6A4	Mast cell	Innate
PTGS2	Mast cell	Innate
EGR3	Mast cell	Innate
PILRA	Mast cell	Innate
CCR2	MDSC	Innate
CD14	MDSC	Innate
CD2	MDSC	Innate
CD86	MDSC	Innate
CXCR4	MDSC	Innate
FCGR2A	MDSC	Innate
FCGR2B	MDSC	Innate
FCGR3A	MDSC	Innate
FERMT3	MDSC	Innate
GPSM3	MDSC	Innate
IL18BP	MDSC	Innate
IL4R	MDSC	Innate
ITGAL	MDSC	Innate
ITGAM	MDSC	Innate
PARVG	MDSC	Innate
PSAP	MDSC	Innate
PTGER2	MDSC	Innate
PTGES2	MDSC	Innate
S100A8	MDSC	Innate
S100A9	MDSC	Innate
ASGR2	Monocyte	Innate
CFP	Monocyte	Innate
ASGR1	Monocyte	Innate
CD1D	Monocyte	Innate

UPK3A	Monocyte	Innate
ACTG1	Monocyte	Innate
ANXA5	Monocyte	Innate
ATP6V1B2	Monocyte	Innate
CFL1	Monocyte	Innate
DAZAP2	Monocyte	Innate
CTBS	Monocyte	Innate
EMR4P	Monocyte	Innate
HIVEP2	Monocyte	Innate
MARCKSL1	Monocyte	Innate
MBP	Monocyte	Innate
MMP15	Monocyte	Innate
PNPLA6	Monocyte	Innate
TMBIM6	Monocyte	Innate
PQBP1	Monocyte	Innate
TEX264	Monocyte	Innate
IKZF1	Monocyte	Innate
AKT3	Natural killer cell	Innate
AXL	Natural killer cell	Innate
BST2	Natural killer cell	Innate
CDH2	Natural killer cell	Innate
CRTAM	Natural killer cell	Innate
CSF2RA	Natural killer cell	Innate
CTSZ	Natural killer cell	Innate
CXCL1	Natural killer cell	Innate
CYTH1	Natural killer cell	Innate
DAXX	Natural killer cell	Innate
DGKH	Natural killer cell	Innate
DLL4	Natural killer cell	Innate
DPYD	Natural killer cell	Innate
ERBB3	Natural killer cell	Innate
F11R	Natural killer cell	Innate
FAM27A	Natural killer cell	Innate
FAM49A	Natural killer cell	Innate
FASLG	Natural killer cell	Innate
FCGR1A	Natural killer cell	Innate
FN1	Natural killer cell	Innate
FSTL1	Natural killer cell	Innate
FUCA1	Natural killer cell	Innate
GBP3	Natural killer cell	Innate
GLS2	Natural killer cell	Innate
GRB2	Natural killer cell	Innate
LST1	Natural killer cell	Innate
BCL2	Natural killer cell	Innate
CDC5L	Natural killer cell	Innate
FGF18	Natural killer cell	Innate
FUT5	Natural killer cell	Innate
FZR1	Natural killer cell	Innate
GAGE2	Natural killer cell	Innate
IGFBP5	Natural killer cell	Innate
KANK2	Natural killer cell	Innate
LDB3	Natural killer cell	Innate
BTN2A2	Natural killer T cell	Innate
CD101	Natural killer T cell	Innate
CD109	Natural killer T cell	Innate
CNPY3	Natural killer T cell	Innate
CNPY4	Natural killer T cell	Innate
CREB1	Natural killer T cell	Innate

CRTC2	Natural killer T cell	Innate
CRTC3	Natural killer T cell	Innate
CSF2	Natural killer T cell	Innate
KLRC1	Natural killer T cell	Innate
FUT4	Natural killer T cell	Innate
ICAM2	Natural killer T cell	Innate
IL32	Natural killer T cell	Innate
LAMP2	Natural killer T cell	Innate
LILRB5	Natural killer T cell	Innate
KLRG1	Natural killer T cell	Innate
HSPA4	Natural killer T cell	Innate
HSPB6	Natural killer T cell	Innate
ISM2	Natural killer T cell	Innate
ITIH2	Natural killer T cell	Innate
KDM4C	Natural killer T cell	Innate
KIR2DS4	Natural killer T cell	Innate
KIRREL3	Natural killer T cell	Innate
SDCBP	Natural killer T cell	Innate
NFATC2IP	Natural killer T cell	Innate
MICB	Natural killer T cell	Innate
KIR2DL1	Natural killer T cell	Innate
KIR2DL3	Natural killer T cell	Innate
KIR3DL1	Natural killer T cell	Innate
KIR3DL2	Natural killer T cell	Innate
NCR1	Natural killer T cell	Innate
FOSL1	Natural killer T cell	Innate
TSLP	Natural killer T cell	Innate
SLC7A7	Natural killer T cell	Innate
SPP1	Natural killer T cell	Innate
TREM2	Natural killer T cell	Innate
UBASH3A	Natural killer T cell	Innate
YBX2	Natural killer T cell	Innate
CCDC88A	Natural killer T cell	Innate
CLEC1A	Natural killer T cell	Innate
THBD	Natural killer T cell	Innate
PDPN	Natural killer T cell	Innate
VCAM1	Natural killer T cell	Innate
EMR1	Natural killer T cell	Innate
CREB5	Neutrophil	Innate
CDA	Neutrophil	Innate
CHST15	Neutrophil	Innate
S100A12	Neutrophil	Innate
APOBEC3A	Neutrophil	Innate
CASP5	Neutrophil	Innate
MMP25	Neutrophil	Innate
HAL	Neutrophil	Innate
C1orf183	Neutrophil	Innate
FFAR2	Neutrophil	Innate
MAK	Neutrophil	Innate
CXCR1	Neutrophil	Innate
STEAP4	Neutrophil	Innate
MGAM	Neutrophil	Innate
BTNL8	Neutrophil	Innate
CXCR2	Neutrophil	Innate
TNFRSF10C	Neutrophil	Innate
VNN3	Neutrophil	Innate
CBX6	Plasmacytoid dendritic cell	Innate
DAB2	Plasmacytoid dendritic cell	Innate

DDX17	Plasmacytoid dendritic cell	Innate
HIGD1A	Plasmacytoid dendritic cell	Innate
IDH3A	Plasmacytoid dendritic cell	Innate
IL3RA	Plasmacytoid dendritic cell	Innate
MAGED1	Plasmacytoid dendritic cell	Innate
NUCB2	Plasmacytoid dendritic cell	Innate
OFD1	Plasmacytoid dendritic cell	Innate
OGT	Plasmacytoid dendritic cell	Innate
PDIA4	Plasmacytoid dendritic cell	Innate
SERTAD2	Plasmacytoid dendritic cell	Innate
SIRPA	Plasmacytoid dendritic cell	Innate
TMED2	Plasmacytoid dendritic cell	Innate
ENG	Plasmacytoid dendritic cell	Innate
FCAR	Plasmacytoid dendritic cell	Innate
IGF1	Plasmacytoid dendritic cell	Innate
ITGA2B	Plasmacytoid dendritic cell	Innate
GABARAP	Plasmacytoid dendritic cell	Innate
GPX1	Plasmacytoid dendritic cell	Innate
KRT23	Plasmacytoid dendritic cell	Innate
PROK2	Plasmacytoid dendritic cell	Innate
RALB	Plasmacytoid dendritic cell	Innate
RETNLB	Plasmacytoid dendritic cell	Innate
RNF141	Plasmacytoid dendritic cell	Innate
SEC14L1	Plasmacytoid dendritic cell	Innate
SEPX1	Plasmacytoid dendritic cell	Innate
EMP3	Plasmacytoid dendritic cell	Innate
CD300LF	Plasmacytoid dendritic cell	Innate
ABTB1	Plasmacytoid dendritic cell	Innate
KLHL21	Plasmacytoid dendritic cell	Innate
PHRF1	Plasmacytoid dendritic cell	Innate

Table S4. 1989 immune genes.

Gene Symbol
A2M
ABCA6
ABCA8
ABCA9
ABCB1
ABCC9
ABCD2
ABI3
ABI3BP
ACAP1
ACE
ACHE
ACOXL
ACP5
ACSL5
ACSM5
ACSS3
ACTA2
ACTN1
ACVR1
ACVR1B
ACVR2A
ACVR2B
ACVRL1
ADAM12
ADAM28
ADAM6
ADAM8
ADAMDEC1
ADAMTS10
ADAMTS12
ADAMTS14
ADAMTS16
ADAMTS2
ADAMTS4
ADAMTS5
ADAMTS9
ADAMTSL2
ADAP2
ADAT1
ADCY4
ADCYAP1
ADORA2A
ADORA3
ADPRH
ADRA2A
ADRM1
AEBP1
AFF3
AGAP2
AGTR1
AHCYL2
AIF1
CRYBG2
AIM2
AKAP12

AKAP2
AKAP5
AKNA
ALDH1A1
ALDH3B1
ALOX5
ALOX5AP
ALOXE3
ALPK2
AMH
AMHR2
JAML
AMIGO3
AMPD1
AMPH
ANGPTL1
ANGPTL2
ANK2
ANKRD17
ANKRD22
ANKRD36BP1
ANKRD44
ANKRD55
SOWAHD
ANO6
ANTXR1
ANTXR2
ANXA6
AOAH
AOC3
AP1S2
AP3B1
APBB1IP
APBB2
APLNR
APOBR
APOBEC3A
APOBEC3D
APOBEC3G
APOBEC3H
APOC1
APOC2
APOE
APOL3
APOL6
AQP10
AQP1
AQP9
AREG
ARHGAP15
ARHGAP18
ARHGAP22
ARHGAP25
ARHGAP30
ARHGAP31
ARHGAP4
ARHGAP6
ARHGAP9

ARHGDIB
ARHGEF15
ARHGEF37
ARHGEF6
ARID5A
ARL6IP5
ARRB1
ARRB2
ARRDC5
ARSB
ART4
ASAH1
CLMP
ASGR2
ASPN
ASRGL1
ASXL2
ASXL3
ATE1
ATP10A
ATP2A3
ATP8A1
ATP8B4
AVPR1A
AZGP1
B2M
B3GAT1
BANK1
BATF
BATF2
BATF3
BCL2A1
BCL2L14
BCL6B
BDKRB2
BEND5
BEX5
BFSP2
BGN
BHLHA15
BHLHE22
BHLHE41
BICC1
BIN2
BIRC6
BLK
BMP2
BMP2K
BMP7
BMPR1A
BMPR1B
BMPR2
BNC2
BST1
BST2
BTK
BTLA
BTN2A2

BTN3A1
BTN3A2
BTN3A3
C10orf128
VSIR
VSTM4
C10orf99
C11orf21
RUBCNL
MEDAG
CEP128
SLIRP
RTRAF
C14orf2
C15orf48
C15orf53
C16orf54
MILR1
SCIMP
LDLRAD4
PEAK3
C19orf38
TRIR
MCEMP1
C19orf66
C1orf116
C1orf127
GCSAML
C1orf162
PIK3CD-AS1
C1orf228
THEMIS2
C1orf54
C1QA
C1QB
C1QC
C1QTNF7
LAMP5
C2
RTP5
TRABD2A
C3
C3AR1
C4A
NDNF
C5AR1
DCANP1
CREBRF
C5orf56
ADTRP
UQCC2
C6orf132
CCDC170
C7
CPED1
NUGGC
ERCC6L2
C9orf139

CYSRT1
CACNA1C
CACNA2D2
CACNA2D4
CALB2
CALD1
CALR
CAMK1
CAMK4
CANX
CARD11
CARD8
CARD9
CASP5
CASS4
CAV1
CCDC102B
CCDC141
CCDC69
CCDC80
CCL11
CCL13
CCL14
CCL15
CCL1
CCL16
CCL17
CCL18
CCL19
CCL20
CCL21
CCL22
CCL23
CCL24
CCL25
CCL26
CCL2
CCL28
CCL3
CCL3L1
CCL3L3
CCL4
CCL4L1
CCL5
CCL7
CCL8
CCNT1
CCR10
CCR1
CCR2
CCR3
CCR4
CCR5
CCR6
CCR7
CCR8
CCR9
CCRL2

CD14
CD160
CD163
CD163L1
CD180
CD19
CD1A
CD1B
CD1C
CD1D
CD1E
CD200
CD200R1
CD207
CD209
CD226
CD22
CD244
CD247
CD248
CD274
CD27
CD28
CD2
CD300A
CD300C
CD300E
CD300LB
CD300LF
CD302
CD33
CD34
CD36
CD37
CD3D
CD3E
CD3G
CD40
CD40LG
CD48
CD4
CD52
CD53
CD58
CD5
CD68
CD6
CD69
CD70
CD72
CD74
CD7
CD79A
CD79B
CD80
CD84
CD86
CD8A

CD8B
CD93
CD96
ADGRE5
CDC42SE2
CDH11
CDH20
CDH3
CDH5
CDH6
CDK15
CDKL5
CDSN
CEACAM19
CEACAM21
CEACAM4
ADA2
CELF2
CERKL
CETP
CFP
CHAC1
CHIT1
CHN1
CHRD
CHRDL1
CHRNA6
CHST13
CHST2
CIITA
CILP
CISH
CLCF1
CLEC10A
CLEC11A
CLEC12A
CLEC14A
CLEC1A
CLEC3B
CLEC4A
CLEC4D
CLEC4E
CLEC4G
CLEC4M
CLEC5A
CLEC6A
CLEC9A
CLECL1
CLIC2
CLIC3
CLIC5
CLIP3
CLNK
CLOCK
CLTB
CMA1
CMAHP
CMKLR1

CMPK2
CNFN
CNR2
CNRIP1
CNTF
CNTFR
COL10A1
COL11A1
COL12A1
COL14A1
COL15A1
COL18A1
COL1A1
COL1A2
COL6A5
COL3A1
COL4A1
COL4A2
COL5A1
COL5A2
COL5A3
COL6A1
COL6A2
COL6A3
COL6A6
COL8A1
COLEC12
CORIN
CORO1A
COTL1
CPA3
CPNE5
CPVL
CPXM1
CPZ
CR1
CR1L
CR2
CRCT1
CREB1
CREB3L1
CREBL2
CRISPLD2
CRLF2
CRTAM
CRYBB1
CSF1
CSF1R
CSF2
CSF2RA
CSF2RB
CSF3
CSF3R
CSGALNACT2
CSMD2
CST7
CTF1
CTGF

CTHRC1
CTLA4
CTSB
CTSE
CTSG
CTSK
CTSL
CTSO
CTSS
CTSW
CTSZ
CTTNBP2
CX3CL1
CX3CR1
CXCL10
CXCL11
CXCL12
CXCL1
CXCL13
CXCL16
CXCL2
CXCL3
CXCL5
CXCL6
CXCL9
CXCR1
CXCR2
CXCR2P1
CXCR3
CXCR4
CXCR5
CXCR6
ACKR3
CXorf21
CXorf36
CXorf65
CYBA
CYBB
CYFIP2
CYP1B1
CYP27A1
CYSLTR1
CYSLTR2
CYTH4
CYTIP
DAAM2
DAB2
DACT1
DACT3
ACKR1
DBH
DCBLD1
DCHS1
DCN
DDI2
DDR2
DDX58
DDX60

DENND1C
DENND2A
DERL3
DHRS1
DHRS9
DIXDC1
DKK2
DLC1
DLL4
DMKN
DMXL2
DNAH8
DNAJC5B
DOCK10
DOCK11
DOCK2
DOCK4
DOCK8
DOK1
DOK2
DOK3
DOK5
DOK6
DPEP1
DPEP2
DPP8
DPT
DSC1
DSG1
DSP
DUOXA1
DUSP16
DUSP4
DYSF
E2F5
EBF1
EBF2
EBI3
ECM2
ECSCR
EDA
EDA2R
EDAR
EDNRA
EDNRB
EFEMP2
EFTUD2
EGF
EGFR
EHD2
ADGRL4
EMCN
EMILIN1
EMILIN2
ADGRE2
ADGRE4P
ENG
ENO3

ENOX1
ENPEP
ENPP2
ENPP3
ENPP4
ENTPD1
EOMES
EP300
EPCAM
EPO
EPOR
EPS8
EPS8L1
EPSTI1
ERAP1
ERAP2
ERN1
ERP27
ESAM
ETS1
ETV3
ETV7
EVI2A
EVI2B
EVPL
F13A1
F2R
F5
FABP3
FCMR
FAM105A
FAM107A
PCED1B
DENND6B
FAM129C
FAM13C
FAM155A
FAM168A
FAM171B
FAM177B
TVP23A
CCSER1
FAM198B
FAM19A5
FAM25A
FAM25BP
CALHM5
CALHM6
PIEZO2
STRIP2
FAM46B
FAM46C
FAM49A
NXPE4
MINDY2
RIPOR2
FAM78A
FAM83A

FAM92B
FAP
FAS
FASLG
FAT4
FBLN2
FBLN5
FBN1
FBP1
FBXL7
FBXO6
FCAR
FCER1A
FCER1G
FCER2
FCGBP
FCGR1A
FCGR1B
FCGR1CP
FCGR2A
FCGR2B
FCGR2C
FCGR3A
FCGR3B
FCGRT
FCN1
FCRL1
FCRL2
FCRL3
FCRL4
FCRL5
FCRL6
FCRLA
FERMT2
FERMT3
FGD2
FGD3
FGD5
FGF14
FGF7
FGL2
FGR
FHL5
FIBIN
FICD
VEGFD
FILIP1L
FKBP11
FKBP7
FLI1
ANKRD36BP2
FLT1
FLT3
FLT3LG
FLT4
FLVCR2
FMNL1
FMNL3

FMOD
FN1
FNBP1
FNDC1
FNIP2
FOLR2
FOXP3
FPR1
FPR2
FPR3
FRZB
FSCN1
FSTL1
FSTL3
FUCA1
FUT7
FYB1
FYN
FZD4
GAB3
GALM
GALNT15
GAPT
GAS7
GATA1
GATA2
GATA3
GATM
GBGT1
GBP1
GBP2
GBP4
GBP5
GCSAM
GDF5
GFI1
GFRA3
GGT1
GGT5
GGTA1P
GHR
GHRL
GIMAP1
GIMAP2
GIMAP4
GIMAP5
GIMAP6
GIMAP7
GIMAP8
GIPC3
GIT2
GJA4
GJA5
GJB2
GJB3
GJB5
GJD3
GLIPR2

GLIS3
GLRX
COLGALT2
GLT8D2
GMFG
GMIP
GMPR
GNA15
GNAI2
GNG11
GNG2
GNG7
GNGT2
GNLY
GNS
GPBAR1
GPC5
GPC6
GPIHBP1
ADGRG5
ADGRF4
ADGRF5
ADGRA2
GPR132
ADGRD1
GPR137B
GPR141
GPR15
GPR157
GPR171
GPR174
GPR18
GPR183
GPR25
GPR34
GPR35
GPR4
GPR55
GPR65
GPR78
GPR82
GPR84
GPRIN3
GPSM3
GRAP
GRAP2
GREM1
GRIN3A
GSDMA
GTF2A1
GUCY1A2
GUCY1A3
GVINP1
GYPC
GZMA
GZMB
GZMH
GZMK

GZMM
HAMP
HAPLN3
HAVCR1
HAVCR2
HCG26
HCK
HCLS1
HCP5
HCST
HDC
HECW2
HEPH
HEPHL1
HERC6
HERPUD1
HEYL
HFE
HGF
HIC1
HIPK3
HIST1H2AE
HIST1H2AG
HIST1H2AM
HIST1H3H
HK3
HLA-A
HLA-B
HLA-C
HLA-DMA
HLA-DMB
HLA-DOA
HLA-DOB
HLA-DPA1
HLA-DPB1
HLA-DPB2
HLA-DQA1
HLA-DQA2
HLA-DQB1
HLA-DQB2
HLA-DRA
HLA-DRB1
HLA-DRB5
HLA-DRB6
HLA-E
HLA-F
HLA-G
HLA-H
HLX
HMCN1
ARHGAP45
HMSD
HNMT
HPGD
HPGDS
HRH2
HS3ST1
HS3ST2

HSD11B1
HSD17B14
HSH2D
HSP90AA1
HSP90AB1
HSPA12B
HSPA1A
HSPA1B
HSPA1L
HSPA2
HSPA4
HSPA5
HSPA6
HSPA8
HTR2A
HTRA3
HTRA4
HVCN1
HYDIN
ICAM1
ICAM2
ICAM3
ICK
ICOS
ICOSLG
IDO1
IDO2
IFFO1
IFI27
IFI30
IFI35
IFI44
IFI44L
IFI6
IFIH1
IFIT2
IFIT3
IFIT5
IFITM1
IFITM3
IFNA13
IFNA21
IFNAR1
IFNAR2
IFNB1
IFNE
IFNG
IFNGR1
IFNGR2
IFNK
IFNW1
IGDCC4
IGF1
JCHAIN
IGLL1
IGSF10
IGSF21
IGSF6

IKZF1
IKZF3
IL10
IL10RA
IL10RB
IL11
IL11RA
IL12A
IL12B
IL12RB1
IL12RB2
IL13
IL13RA1
IL15
IL15RA
IL16
IL17A
IL17B
IL17RA
IL17RB
IL18
IL18BP
IL18R1
IL18RAP
IL19
IL1A
IL1B
IL36G
IL1R1
IL1R2
IL1RAP
IL1RL1
IL1RN
IL20
IL20RA
IL20RB
IL21
IL21R
IL22
IL22RA1
IL22RA2
IL2
IL23A
IL23R
IL24
IL27
IFNL2
IFNLR1
IFNL1
IL2RA
IL2RB
IL2RG
IL32
IL3RA
IL4
IL4I1
IL4R
IL5

IL5RA
IL6
IL6R
IL6ST
IL7
IL7R
CXCL8
IL9
IL9R
INHBA
INHBB
INHBC
INHBE
INMT
INPP5D
IPCEF1
IQGAP2
IRF1
IRF4
IRF7
IRF8
IRF9
ISG15
ISG20
ISLR
ITGA11
ITGA1
ITGA2B
ITGA4
ITGA5
ITGA8
ITGA9
ITGAD
ITGAL
ITGAM
ITGAX
ITGB1
ITGB2
ITGB3
ITGB7
ITGBL1
ITK
ITM2A
IVL
JAK2
JAK3
JAKMIP1
JAM2
JAM3
JMY
JSRP1
JUP
KCNA3
KCNAB2
KCND2
KCNE4
KCNH2
KCNJ10

KCNJ8
KCNK13
KCNK6
KCNMB1
KCNN3
KCNN4
KCNT2
KCTD12
KDR
FAM30A
KIAA0368
TESPA1
KIAA0754
JCAD
KIAA1549
TLDC1
SHISAL1
KIAA1755
KIF21B
KIR2DL1
KIR2DL3
KIR2DL4
KIR2DS4
KIR3DL1
KIR3DL2
KIR3DL3
KIRREL1
KIT
KITLG
KL
KLHDC10
KLHL11
KLHL23
KLHL6
KLK7
KLK9
KLRB1
KLRC1
KLRC2
KLRC3
KLRC4
KLRD1
KLRG1
KLRK1
KMO
KRT1
KRT14
KRT16
KRT6A
KRT6B
KRT6C
KRT78
LAD1
LAG3
LAIR1
LAIR2
LAMA2
LAMA4

LAMC2
LAP3
LAPTM5
LAT
LAT2
LATS1
LATS2
LAX1
LCE3D
LCK
LCN10
LCOR
LCP1
LCP2
LDB2
LEP
LEPR
LGALS2
LGALS9
LGI2
LGMN
LHFPL6
LHFPL2
LIF
LIFR
LIG3
LILRA1
LILRA2
LILRA3
LILRA4
LILRA5
LILRA6
LILRB1
LILRB2
LILRB3
LILRB4
LILRB5
LILRP2
LIMD2
LIME1
LIMS1
LIPA
LMOD1
LMTK2
UNQ6494
FAM83A-AS1
LINC00426
PCED1B-AS1
TNFRSF14-AS1
LINC00654
LINC00926
SMIM1
LGALS17A
MIR31HG
LOC606724
LOC653786
LOC730101
BMS1P20

LOXL2
LOXL3
LPAR4
LPL
PLPPR4
LPXN
LRCH2
LRMP
LRP6
LRRC15
LRRC17
LRRC25
LRRC32
NRROS
LSAMP
LSP1
LST1
LTA
LTB
LTBP2
LTBR
LTC4S
LUM
LY86
LY9
LY96
LYL1
LYN
LYPD3
LYPD5
LYVE1
LYZ
MAGEL2
MAN1A1
MAN1A2
MAN1C1
MAOB
MAP1LC3C
MAP3K2
MAP4K1
MAP7D1
43160
43167
MARCO
MBNL3
SLC25A53
MCOLN2
MED13
MED13L
MEF2B
MEF2C
MEI1
MEOX2
MET
MFAP3
MFAP4
MFNG
MFRP

MFSD7
MGAT4A
MGAT5
MZB1
MGP
NA
MICAL2
MICB
MIR155HG
MITF
MLPH
MMP12
MMP1
MMP14
MMP16
MMP2
MMP25
MMP3
MMP9
MMRN1
MMRN2
MNDA
MPEG1
MPL
MPP1
MR1
MRC1
MRGPRF
MRO
MRPL27
MRPL55
MRPS12
MRPS21
MRVI1
MS4A14
MS4A1
MS4A2
MS4A4A
MS4A6A
MS4A7
MSR1
MSRB3
MVP
MX1
MXD1
MXRA8
MYCT1
MYEF2
MYO1F
MYO1G
MYO7A
MYO9A
N4BP2
N4BP2L1
NAALADL1
NAIP
NAP1L3
NAPSB

NBEA
NBEAL1
NCCRP1
NCF1
NCF1B
NCF1C
NCF2
NCF4
NCKAP1L
NCOA2
NCR1
NCR3
NEGR1
NFAM1
NFATC2
NFKB2
NFKBID
NFKBIE
NFYA
NFYB
NFYC
NHLRC2
NHSL2
NID2
NIPAL4
NKG7
NLRC3
NLRC4
NLRC5
NLRP12
NLRP3
NMI
NOD2
NOTCH4
NOVA2
NOX4
NR1H3
NR5A2
NRP1
NRXN3
NT5E
NTM
NTNG2
NTRK1
OAS1
OAS2
OAS3
OASL
TENM3
OGFRL1
OGN
OLFML1
OLFML2B
OLFML3
OLR1
OMD
OSCAR
OSM

OSMR
OTOA
OVOL1
P2RX1
P2RX4
P2RX5
P2RX7
P2RY10
P2RY11
P2RY12
P2RY13
P2RY14
P2RY8
P4HA3
PABPC5
PADI2
PAFAH1B2
PAG1
PAK5
PALM2-AKAP2
PARM1
PARP12
PARP14
PARP15
PARP9
PARVG
PATL2
PATZ1
PBX4
PCDH12
PCDH17
PCDH18
PCDHGA12
PCOLCE
PCYOX1L
PDCD1
PDCD1LG2
PDE1A
PDE1B
PDE3A
PDE3B
PDE4B
PDE6G
PDGFA
PDGFB
PDGFC
PDGFRA
PDGFRB
PDGFRL
PDIA2
PDIA3
PDZK1IP1
PDZRN3
PECAM1
PEG3
PFDN2
PGLYRP4
PGM5

PHACTR1
PIK3AP1
PIK3CG
PIK3R5
PIK3R6
PILRA
PIM2
PIP4K2A
PKD2L1
PKHD1L1
PKIB
PKP3
PLA1A
PLA2G2D
PLA2G4E
PLA2G7
PLAC9
PLCB2
PLCB4
PLCL1
PLCL2
PLD4
PLEK2
PLEK
PLEKHM3
PLEKHN1
PLEKHO1
PLEKHO2
PLIN3
PLVAP
PLXDC1
PLXNA4
PLXNC1
PLXND1
PML
PMP22
PNLIPRP3
PNMA2
PNOC
PODN
POSTN
POU2AF1
POU2F2
PLPP3
PLPP4
PLPP7
PPBP
PPFIA2
PPL
PPM1H
PPM1M
PPP1R13L
PPP1R16B
PPP1R9A
PRAM1
PRELP
PREX1
PREX2

PRF1
PRG2
PRKAR2A
PRKAR2B
PRKCB
PRKCQ
PRKG1
PRL
PRLR
PROCR
PROM1
PRRX1
PRSS27
PRTG
PSAP
PSMB10
PSMB8
PSMB9
PSMC1
PSMC2
PSMC3
PSMC4
PSMC5
PSMC6
PSMD10
PSMD11
PSMD13
PSMD14
PSMD1
PSMD2
PSMD3
PSMD4
PSMD5
PSMD6
PSMD7
PSMD8
PSME1
PSME2
PSME3
PSTPIP1
PTAFR
PTCRA
PTGDR
PTGDS
PTGER2
PTGFR
PTGIR
PTGIS
PTH1R
PTPN22
PTPN6
PTPN7
PTPRB
PTPRC
PTPRCAP
PTPRJ
PTPRM
PTPRO

CAVIN1
PUS10
PVR
PVRIG
NECTIN2
NECTIN3
PYHIN1
QPRT
RAB20
RAB33A
RAB37
RAB39A
RAB39B
RAB42
RAB8B
RAD23B
RAD54L2
RAET1E
RAET1G
RAET1L
RAI2
RAMP3
RAPGEF2
RAPGEF6
RARRES2
RARRES3
RASAL3
RASGRF2
RASGRP2
RASGRP3
RASGRP4
RASL12
RASSF2
RASSF3
RASSF4
RASSF5
RASSF6
RBM38
RBP5
RC3H2
RCAN2
RCN3
RCSD1
RDH12
RECK
REL
RELB
RELN
RENBP
REST
RFTN1
RFX5
RFXANK
RFXAP
RGL1
RGL4
RGPD1
RGS13

RGS1
RGS18
RGS5
RHOD
RHOH
RHOJ
RIF1
RIMKLA
RIN1
RIN3
CARMIL2
RNASE1
RNASE2
RNASE6
RNASE7
RNF125
RNF166
RNF180
RNF222
LAMTOR2
ROBO4
ROCK2
ROR1
RPS6KA4
RRN3P2
RSAD2
RTKN2
RTN1
RTP4
RUFY4
NA
RUNX1T1
RUNX3
S100A12
S100A16
S100A2
S100A7
S100A7A
S100A8
S100A9
S100B
S1PR1
S1PR4
SALL2
SAMD14
SAMD3
SAMD9
SAMD9L
SAMHD1
SAMSN1
SARDH
SASH3
SBNO1
SBSN
SCARF1
SCARF2
SCEL
SCML4

SCN7A
SCUBE3
SDC2
SDCBP2
SDR9C7
SDS
SDSL
SEC24A
SEC24D
SECISBP2L
SECTM1
SELENBP1
SELL
SELP
SELPLG
43344
43349
SERINC5
SERPINA1
SERPINE1
SERPINF1
SERPING1
SFMBT2
SFN
SFRP2
SFTPB
SGCD
SGIP1
POMK
PEAK1
SH2B3
SH2D1A
SH2D2A
SH2D3C
SH2D5
SHE
SEM1
SIGLEC10
SIGLEC11
SIGLEC12
SIGLEC14
SIGLEC1
SIGLEC5
SIGLEC6
SIGLEC7
SIGLEC8
SIGLEC9
SIGLEC17P
SIRPB1
SIRPB2
SIRPG
SIT1
SKAP1
SLA2
SLA
SLAMF1
SLAMF6
SLAMF7

SLAMF8
SLC10A2
SLC11A1
SLC12A3
SLC15A3
SLC17A9
SLC18A2
SLC1A7
SLC24A4
SLC25A45
SLC29A3
SLC2A5
SLC34A2
SLC39A2
SLC45A3
SLC6A12
SLC7A7
SLC8A1
SLCO2B1
SLCO5A1
NA
SLIT2
SLIT3
SLURP1
SMAP2
SMPDL3B
SNAI3
SNED1
SNRPF
SNTB1
SNX20
SOD3
SON
SOX17
SOX5
SP100
SP110
SP140
SPAG4
SPARC
SPARCL1
SPATA13
SPI1
SPIB
SPN
SPNS3
SPOCK2
SPON1
SPRR1A
SPRR1B
SPRR2D
SPRR2E
SPRR2G
SPRY1
SRGN
SSC5D
SSTR3
ST3GAL2

ST3GAL5
ST3GAL6
ST6GAL1
ST6GALNAC3
ST8SIA4
STAB1
STAC3
STAP1
STARD13
STARD8
STAT1
STAT4
STAT5A
STK17B
STK33
STRN
STXBP6
SUCNR1
SULF1
SULT1C2
SULT1C4
SULT2B1
SUSD3
SVOPL
SYNE1
SYT11
SYTL3
TAGAP
TAOK1
TAP1
TAP2
TAPBP
TAPBPL
TARP
TBC1D10C
TBCEL
TBX21
TBXA2R
TBXAS1
TCEAL7
TCIRG1
TCL1A
TCN2
TEK
TESC
TFEC
TGFB1
TGFB2
TGFB3
TGFBI
TGFBR1
TGFBR2
TGFBRAP1
TGM1
TGM2
THBS1
THBS2
THEMIS

THPO
THSD7A
THY1
TIE1
TIFAB
TIGIT
TIMD4
PAM16
TIMM50
TIMP2
TIMP3
TLR10
TLR1
TLR4
TLR5
TLR7
TLR8
TLR9
TM4SF18
TM6SF1
DCSTAMP
TMC8
TMEM106A
TMEM119
TMEM140
IGFLR1
TMEM150B
TMEM156
TMEM170B
TMEM176A
TMEM176B
TMEM200A
TMEM204
TMEM229B
TMEM233
TMEM26
TMEM47
TMEM79
SYNDIG1
TMIGD2
TNF
TNFAIP6
TNFAIP8L2
TNFRSF10A
TNFRSF10B
TNFRSF10C
TNFRSF10D
TNFRSF11A
TNFRSF11B
TNFRSF12A
TNFRSF13B
TNFRSF13C
TNFRSF14
TNFRSF17
TNFRSF18
TNFRSF1A
TNFRSF1B
TNFRSF25

TNFRSF4
TNFRSF6B
TNFRSF8
TNFRSF9
TNFSF10
TNFSF11
TNFSF12
TNFSF12-TNFSF13
TNFSF13
TNFSF13B
TNFSF14
TNFSF15
TNFSF18
TNFSF4
TNFSF8
TNFSF9
TNIK
TNIP3
TNN
TNNT2
TNS3
TOX
TPK1
TPSAB1
TPSB2
TPSD1
TPSG1
TRAF1
TRAF3IP3
TRANK1
TRAT1
TREM1
TREM2
TREML1
TRIM21
TRIM22
TRIM61
TRPC4AP
TRPV2
TRPV3
TSHR
TSHZ3
TSLP
TSPAN11
TSPAN32
TSPAN4
TTBK2
TTC16
TTC21B
TTC24
TTC37
TUBA4A
TUBB6
TXK
NME8
TYMP
TYROBP
UBA7

UBASH3A
UBD
UBE2L6
UBR1
UBXN11
UBXN1
UCP2
UHMK1
ULBP1
ULBP2
ULBP3
UNC13D
UNC5C
UNC93B1
USHBP1
USP12
USP51
UTS2
VAMP5
VASH1
VAV1
VCAM1
VCAN
VEGFA
VEGFB
VEGFC
VENTX
VGLL3
VIM
VMO1
VNN2
VPREB3
VPS37D
VSIG4
VWF
WARS
WAS
WDFY4
WFDC12
WIPF1
WISP1
WNT2
XAF1
XBP1
XCL1
XCL2
XCR1
XKR8
XPNPEP2
ZAP70
ZBP1
ZBTB10
ZBTB32
ZC3H12D
ZCCHC24
ZDHHC20
ZEB1
ZEB2

ZFPM2
ZKSCAN1
ZMYND15
ZNF185
ZKSCAN8
ZNF215
ZNF366
ZNF423
ZNF469
ZNF521
ZNF620
ZNF660
ZNF671
ZNF683
ZNF804A
ZNF80
ZNF827
ZNF831
ZNF835

Table S5. DDX39 Expression Differences in Oncomine Database

Cohort	Pathological type	N (tumor/normal)	P value
1.Beroukhim	Non-Hereditary ccRCC	27/11	3.03E-9*
	Hereditary ccRCC	32/11	8.08E-7*
2.Yusenko	ccRCC	26/5	0.007*
3.Gumz	ccRCC	10/10	5.62E-4*
4.Jones	ccRCC	23/23	3.96E-5*
5.Lenburg	ccRCC	9/9	0.018

*P values <0.05 were considered statistically significant.

Table S6. Baseline Information Comparison between TCGA and Changhai cohorts

	Changhai<br>*N=186*	TCGA<br>*N=531*	*P.* Value
Age	56.3 (12.4)	60.6 (12.1)	<0.001*
Gender:			0.742
FEMALE	62 (33.3%)	186 (35.0%)	
MALE	124 (66.7%)	345 (65.0%)	
T4:			<0.001*
0	128 (68.8%)	520 (97.9%)	
1	58 (31.2%)	11 (2.07%)	
OS:			0.901
0	127 (68.3%)	358 (67.4%)	
1	59 (31.7%)	173 (32.6%)	
OS.time	55.0 (28.3)	45.0 (32.8)	<0.001*

*P values <0.05 were considered statistically significant.

Table S7. Correlations between DDX39 and 19712 genes in TCGA ccRCC.

No.	Gene symbol	correlation	pvalue
1	MT-CO3	0.239292218	2.10E-08
2	MT-CO1	0.165548425	0.000119688
3	MT-ND4	0.234055122	4.32E-08
4	MT-CO2	0.254296707	2.42E-09
5	MT-ATP6	0.223692217	1.71E-07
6	MT-ND2	0.226450685	1.19E-07
7	MT-CYB	0.24535191	8.93E-09
8	MT-ND1	0.173024283	5.74E-05
9	MT-ND3	0.216520943	4.27E-07
10	MT-ATP8	0.181700216	2.36E-05
11	MT-ND4L	0.165669373	0.000118304
12	FTL	0.263512498	6.00E-10
13	MT-ND6	0.035921567	0.406995476
14	MT-ND5	0.018355731	0.671848864
15	TMSB10	0.523630811	5.33E-39
16	GAPDH	0.289321306	8.93E-12
17	ACTB	0.458685689	3.42E-29
18	EEF1A1	-0.334196511	2.00E-15
19	CD74	0.288074942	1.11E-11
20	B2M	-0.143901527	0.000843668
21	HLA-B	0.185594375	1.56E-05
22	HLA-DRA	0.023672247	0.584834628
23	RPS11	0.405763038	1.27E-22
24	HLA-A	0.313885655	1.07E-13
25	ACTG1	0.291607604	6.02E-12
26	TMSB4X	0.378603254	1.12E-19
27	PSAP	-0.194618076	5.78E-06
28	RPS12	0.005845113	0.892704556
29	IGFBP7	-0.024616136	0.569948576
30	ENO1	0.066765059	0.122978947
31	GPX3	-0.130339047	0.002522791
32	EEF2	-0.069629687	0.107676424
33	RPS18	0.287754906	1.17E-11
34	HLA-C	0.201559223	2.61E-06
35	SPP1	-0.252119843	3.34E-09
36	RPS27	0.153990093	0.000350476
37	RPL13A	0.385364457	2.19E-20
38	RPS6	0.219629488	2.88E-07
39	RPL8	0.396045919	1.55E-21
40	TPT1	0.124871097	0.003817615
41	HLA-DRB1	0.186587825	1.40E-05
42	VIM	0.24708307	6.97E-09
43	HLA-E	0.062853504	0.146545786
44	RPL3	0.190000165	9.65E-06
45	SPARC	-0.112962864	0.00892007
46	CTSD	-0.065191927	0.132075115
47	CD24	-0.311048177	1.82E-13
48	RPL7A	0.309256006	2.55E-13
49	IGFBP4	0.291272245	6.38E-12
50	RPL19	0.475166676	1.74E-31
51	DUSP1	-0.047526652	0.272491218
52	CALR	0.273894172	1.16E-10
53	S100A11	0.507265206	2.51E-36
54	RPS4X	0.166660184	0.00010752
55	S100A6	0.336176477	1.33E-15
56	HSP90AB1	-0.236224677	3.21E-08

57	IFITM3	0.444465942	2.60E-27
58	TPI1	0.069242412	0.109652706
59	ATP1B1	-0.257597393	1.48E-09
60	RPL11	0.300517703	1.25E-12
61	IGFBP3	0.090654464	0.036059371
62	RPS25	0.243681694	1.13E-08
63	RPL10A	0.003680064	0.932323937
64	RPS8	0.320393432	3.09E-14
65	PEBP1	0.008117222	0.851413013
66	BGN	0.184838169	1.69E-05
67	ALDOA	0.236704961	3.01E-08
68	RPLP0	0.269032943	2.53E-10
69	UBB	0.048861273	0.259237559
70	RPL5	0.107587203	0.012777563
71	TXNIP	-0.325887933	1.06E-14
72	CD63	0.373673955	3.58E-19
73	RHOB	-0.082602856	0.056209886
74	FTH1	0.05432688	0.209634212
75	ATP5B	-0.303596287	7.19E-13
76	RPS2	0.469026023	1.29E-30
77	SLC25A6	0.238075896	2.49E-08
78	APP	-0.405829348	1.25E-22
79	RPLP1	0.54954986	1.52E-43
80	RPL10	0.223783365	1.69E-07
81	HSPB1	0.595787164	1.04E-52
82	RPLP2	0.527110283	1.38E-39
83	PLVAP	-0.012733081	0.768880111
84	RPS21	0.417164674	6.11E-24
85	GNB2L1	0.234396686	4.12E-08
86	CRYAB	0.136753319	0.001521125
87	NDUFA4L2	0.218555585	3.30E-07
88	LDHA	-0.059405446	0.170047864
89	RPS20	0.429665557	1.91E-25
90	PKM	0.084336614	0.051221022
91	UBC	0.255709327	1.96E-09
92	LAPTM4A	-0.246364875	7.73E-09
93	PFN1	0.650566027	1.07E-65
94	A2M	-0.206254542	1.50E-06
95	TMBIM6	-0.437273798	2.15E-26
96	TMEM176B	0.131600725	0.002287733
97	RPL4	0.248407587	5.75E-09
98	PRDX5	0.349339968	8.43E-17
99	COX8A	0.248694987	5.52E-09
100	RPL27	0.359183559	9.79E-18
101	MYL12B	0.13820846	0.001352099
102	RGS5	-0.242106859	1.42E-08
103	RPL12	0.468194985	1.68E-30
104	RPS16	0.453703772	1.60E-28
105	CLU	-0.008365581	0.846921962
106	HSP90B1	-0.12882768	0.002833263
107	RPL35	0.530163256	4.15E-40
108	S100A10	0.415149026	1.05E-23
109	NDUFS5	0.364183634	3.19E-18
110	BSG	0.237450356	2.71E-08
111	NDRG1	-0.101301618	0.019095341
112	CANX	-0.511700137	4.89E-37
113	PDZK1IP1	0.206851414	1.40E-06
114	TIMP1	0.421466254	1.88E-24

115	EZR	-0.179277276	3.03E-05
116	RPL41	0.064474134	0.136394474
117	RPL36AL	0.094190854	0.029377018
118	CLIC1	0.342806233	3.38E-16
119	PTMA	0.331934698	3.16E-15
120	LGALS1	0.531116475	2.85E-40
121	SERPINA1	0.033862784	0.434425785
122	RPL7	-0.091453224	0.034446574
123	EIF1	0.147678345	0.000611129
124	TAGLN2	0.336326696	1.29E-15
125	RPL29	0.409594993	4.65E-23
126	RPL24	0.349662501	7.86E-17
127	P4HB	0.335333563	1.59E-15
128	FAU	0.489123637	1.58E-33
129	PTTG1IP	-0.03939349	0.363141189
130	HSPA8	-0.37182155	5.52E-19
131	YBX1	0.105125073	0.014990588
132	PGK1	-0.384462994	2.73E-20
133	RHOA	-0.324844304	1.30E-14
134	AQP1	-0.126766201	0.003312867
135	PTMS	0.540315294	7.04E-42
136	RPS3A	-0.111343121	0.009955573
137	RPS14	0.239847263	1.94E-08
138	PPDPF	0.361637638	5.66E-18
139	RPS19	0.572962207	5.29E-48
140	ITM2B	-0.235405665	3.59E-08
141	CXCL14	0.025892392	0.550112092
142	IGFBP5	-0.158516142	0.000232127
143	LDHB	-0.25116634	3.85E-09
144	EDF1	0.591578999	8.17E-52
145	SARAF	-0.313247569	1.21E-13
146	ANGPTL4	0.302417897	8.90E-13
147	CNBP	-0.191666467	8.03E-06
148	APOE	0.325556986	1.13E-14
149	NPM1	-0.167269444	0.000101355
150	RPS24	0.306249329	4.43E-13
151	MYL6	0.32743343	7.78E-15
152	HSPA5	-0.056655754	0.190724991
153	RPS13	0.138868581	0.001281271
154	ALDH1A1	-0.150263511	0.000487933
155	CTSB	0.040482735	0.350014382
156	MYH9	-0.081850275	0.058497683
157	PABPC1	0.100706831	0.019814969
158	ZFP36	0.187203535	1.31E-05
159	HLA-DRB5	0.047557387	0.27218089
160	RPS27A	0.223677666	1.71E-07
161	PRDX1	0.145305556	0.000749031
162	BTF3	-0.009762934	0.821750165
163	RPL9	0.126282611	0.003435556
164	ARF1	0.33022256	4.46E-15
165	COX6B1	0.436861136	2.43E-26
166	CTSZ	0.183041163	2.05E-05
167	RPL23A	0.313854379	1.08E-13
168	PGRMC1	-0.275374726	9.16E-11
169	RPL13	0.44401581	2.97E-27
170	RPS3	0.289406192	8.80E-12
171	SERPING1	0.057485187	0.184302254
172	TUBB	0.247044902	7.01E-09

173	YWHAE	-0.058548082	0.176306955
174	CHCHD2	0.121015464	0.005065222
175	EEF1B2	0.174277137	5.06E-05
176	GPX1	0.404403655	1.81E-22
177	RPS9	0.417259371	5.95E-24
178	NNMT	0.236898883	2.93E-08
179	ATP6V1F	0.277376299	6.61E-11
180	RPS23	0.060210574	0.164322748
181	OST4	0.457551703	4.87E-29
182	NAT8	0.011839728	0.784682
183	RPL36	0.457169349	5.48E-29
184	MSN	-0.14467444	0.000790284
185	SLC25A5	-0.235407126	3.59E-08
186	RPS17	0.32766579	7.43E-15
187	ANXA5	-0.004419982	0.918759971
188	HLA-DPB1	0.154297531	0.000340924
189	GSTP1	0.175367852	4.53E-05
190	SDC4	-0.113117066	0.008826675
191	RPL32	0.307284176	3.66E-13
192	RPL35A	0.262720713	6.77E-10
193	STOM	-0.270590977	1.98E-10
194	RPS5	0.236990089	2.89E-08
195	RPL6	0.259579036	1.10E-09
196	RPL18	0.491360766	7.29E-34
197	COX7C	0.060569076	0.161820594
198	CCND1	-0.206225302	1.50E-06
199	DAD1	0.035432095	0.413421903
200	HINT1	0.003543071	0.934837537
201	LGALS3BP	0.267624953	3.16E-10
202	COL4A1	-0.148849535	0.000552117
203	RPL34	0.230231944	7.23E-08
204	CLIC4	-0.342612991	3.51E-16
205	CCNI	-0.610167751	7.11E-56
206	SAT1	0.219294048	3.01E-07
207	PDIA3	-0.039989798	0.355917294
208	PLIN2	0.057844872	0.181567205
209	RPL30	0.316443182	6.59E-14
210	C1QA	0.27186115	1.61E-10
211	GPX4	0.514933036	1.46E-37
212	NDUFB7	0.486241511	4.25E-33
213	NDUFA1	0.324407416	1.41E-14
214	TMEM176A	0.172375407	6.13E-05
215	HSP90AA1	-0.237770854	2.59E-08
216	ATP6V0E1	0.025348815	0.558519191
217	VAMP8	0.180046625	2.80E-05
218	ATP1A1	-0.362707995	4.45E-18
219	RPL26	0.138671873	0.001302011
220	PCBP1	0.002769407	0.949044382
221	SPARCL1	-0.2704909	2.01E-10
222	RBP5	0.151183105	0.00044999
223	COL4A2	0.002686258	0.950572302
224	KRT18	0.307398621	3.59E-13
225	APLP2	-0.369290824	9.92E-19
226	SERINC1	-0.567349207	6.69E-47
227	NUCB1	0.285022964	1.86E-11
228	RPN2	-0.189699765	9.98E-06
229	CD59	-0.404286568	1.87E-22
230	HNRNPA1	-0.032373346	0.454917424

231	HTRA1	0.123955869	0.004085505
232	SRP14	0.050628542	0.242382534
233	RPS15	0.568640072	3.75E-47
234	PSME1	0.343325283	3.03E-16
235	MRFAP1	-0.275491738	8.99E-11
236	C1QC	0.182401888	2.19E-05
237	C1QB	0.213503079	6.22E-07
238	ARHGDIB	0.230388841	7.08E-08
239	C3	0.011004685	0.799533074
240	PPIB	0.451246447	3.38E-28
241	HNRNPK	-0.250514574	4.23E-09
242	BST2	0.136157185	0.001595812
243	CNN3	-0.35541553	2.25E-17
244	CAPNS1	0.46525963	4.31E-30
245	ACTA2	0.064787528	0.134495425
246	MGP	0.023044567	0.594832464
247	RPL31	0.269437442	2.37E-10
248	SRGN	-0.03451729	0.425592017
249	DDIT4	0.218109889	3.50E-07
250	RPL23	0.323260117	1.77E-14
251	GRN	0.142697097	0.000933539
252	EPAS1	-0.263443766	6.06E-10
253	RAC1	0.103259825	0.016884648
254	CALM3	0.016291725	0.706937515
255	SNX3	-0.255944255	1.90E-09
256	PTRF	0.172096785	6.30E-05
257	TXN	0.368953302	1.07E-18
258	SOD1	0.194869966	5.61E-06
259	CD151	0.392622024	3.66E-21
260	JUNB	0.238627607	2.30E-08
261	ECHS1	0.136373326	0.001568352
262	MCL1	-0.054097011	0.211571553
263	RAB7A	-0.073513882	0.089375166
264	GRINA	0.381229094	5.97E-20
265	CSDE1	-0.494473206	2.46E-34
266	EGR1	-0.147960057	0.000596423
267	PRDX6	0.030262664	0.484864415
268	EID1	-0.415160615	1.05E-23
269	HMGN3	0.344889078	2.18E-16
270	VDAC1	-0.23295053	5.02E-08
271	EIF5A	0.66668656	4.76E-70
272	ENG	0.063900736	0.139922387
273	GANAB	-0.252246428	3.28E-09
274	GSTA1	0.006269855	0.884960061
275	ACTN4	0.094482274	0.028877118
276	NFE2L1	-0.140439453	0.001126237
277	RPS28	0.432740722	7.95E-26
278	PEA15	0.108031234	0.012410702
279	ATF4	0.530366289	3.83E-40
280	EIF3I	0.371444824	6.03E-19
281	RNASE1	0.003071073	0.943502693
282	BCAP31	0.340793642	5.14E-16
283	RPL21	-0.090678854	0.03600919
284	PFDN5	0.378088962	1.26E-19
285	DSTN	-0.373951661	3.36E-19
286	CD81	0.12996812	0.002595973
287	CA12	-0.211459836	7.99E-07
288	OAZ1	0.360166876	7.86E-18

289	C10orf10	0.098139674	0.023198464
290	CYR61	0.05511558	0.203084441
291	GHITM	-0.466815674	2.62E-30
292	RPL15	-0.166317288	0.000111143
293	CTSL	-0.00721044	0.867850008
294	TCEB2	0.571523637	1.02E-47
295	MT2A	0.343727302	2.78E-16
296	JUN	0.12957886	0.002674848
297	VAT1	0.13024317	0.002541525
298	CFL1	0.486978234	3.30E-33
299	LMAN2	0.302537426	8.71E-13
300	EIF4G2	-0.476111255	1.27E-31
301	BRK1	-0.039433277	0.362656362
302	KDELR1	0.179482005	2.97E-05
303	CAP1	-0.128431275	0.00292026
304	FLNA	0.107556997	0.012802863
305	FKBP1A	0.040866199	0.345465516
306	C1orf43	-0.111275062	0.010001331
307	RARRES2	0.231597101	6.02E-08
308	YWHAH	-0.049297828	0.255000571
309	TRAM1	-0.366590821	1.84E-18
310	HMOX1	0.114228567	0.008178855
311	IFITM2	0.361897465	5.34E-18
312	PFKP	0.061931485	0.152572907
313	NOP10	0.434516028	4.77E-26
314	DEFB1	-0.141588831	0.001023968
315	HLA-DPA1	0.004121807	0.924223416
316	YWHAQ	-0.34459	2.32E-16
317	HADHA	-0.161736916	0.000171963
318	UBA52	0.437040536	2.30E-26
319	PDK4	-0.199636381	3.26E-06
320	FKBP8	0.559675043	1.99E-45
321	ECH1	0.402726581	2.80E-22
322	FOS	-0.039718386	0.359194007
323	RPL37A	0.396916259	1.24E-21
324	DDOST	0.089064181	0.039462055
325	CHMP4B	0.268810206	2.62E-10
326	HIGD2A	0.340107549	5.93E-16
327	ST13	-0.502139734	1.61E-35
328	MAGED2	-0.004814041	0.911545532
329	ACADVL	0.453246276	1.84E-28
330	IFI6	0.137957654	0.001379941
331	RAB1B	0.139138494	0.001253307
332	RPSA	0.292840853	4.86E-12
333	TMED9	0.301297692	1.09E-12
334	MYL9	0.259221004	1.16E-09
335	RPL27A	0.46312601	8.48E-30
336	BHLHE40	0.062060547	0.151718097
337	COX5B	0.376548872	1.82E-19
338	EIF4B	-0.469755577	1.02E-30
339	KRT8	0.251958229	3.43E-09
340	TOMM20	-0.464599345	5.31E-30
341	PSMB4	0.606839896	3.97E-55
342	TOMM7	0.114133887	0.008232336
343	TUBB4B	0.277058144	6.96E-11
344	YWHAB	-0.44202939	5.35E-27
345	CIB1	0.539821055	8.61E-42
346	NGFRAP1	-0.079286851	0.066876003

347	VEGFB	0.303782264	6.95E-13
348	UGT2B7	-0.042993196	0.320920262
349	ATP6AP2	-0.415098127	1.07E-23
350	SF3B5	0.328435894	6.38E-15
351	TUFM	0.30108236	1.13E-12
352	EIF4H	-0.354860522	2.54E-17
353	ATP6V1G1	-0.055137464	0.202904844
354	GNAS	0.323909175	1.56E-14
355	VEGFA	0.071216277	0.099872057
356	HSPD1	-0.076273777	0.077957926
357	ATP5C1	0.012780132	0.768050456
358	CNDP2	-0.072222308	0.095161118
359	SLC9A3R1	0.115992139	0.007237776
360	TPP1	-0.243431596	1.17E-08
361	SH3BGRL3	0.607831247	2.38E-55
362	RPS7	0.416820506	6.70E-24
363	HSPA9	-0.266974372	3.50E-10
364	FAM127A	0.439046289	1.28E-26
365	XRCC6	-0.125312198	0.003694276
366	TMEM14C	0.056983446	0.18816814
367	RPL38	0.453508998	1.69E-28
368	GLUD1	-0.325721748	1.09E-14
369	AP2M1	0.431516765	1.13E-25
370	RPN1	0.107438584	0.012902473
371	HIST1H1C	0.338609659	8.09E-16
372	MYL12A	0.057152579	0.186858366
373	ACLY	-0.12175067	0.004802185
374	CYB5R3	0.131274742	0.002346469
375	PDLIM1	0.223687168	1.71E-07
376	PTGES3	-0.34354458	2.89E-16
377	ATP5I	0.443493447	3.47E-27
378	LY6E	0.224595823	1.52E-07
379	PECAM1	-0.224453054	1.55E-07
380	CST3	0.352246081	4.50E-17
381	PRDX2	-0.028987228	0.503464288
382	15-Sep	-0.261999123	7.57E-10
383	H3F3B	0.094498824	0.028848948
384	RRAGA	0.089637332	0.038205621
385	ZFP36L1	-0.011231842	0.795485721
386	NPC2	0.170101055	7.68E-05
387	SEC61A1	0.214907845	5.22E-07
388	FN1	-0.117573791	0.006477483
389	TAPBP	0.338416338	8.42E-16
390	PHB2	0.280168647	4.17E-11
391	RPS10	0.216363442	4.35E-07
392	LAPTM5	0.165666002	0.000118342
393	HNRNPA2B1	0.329178526	5.50E-15
394	NDUFB10	0.421718288	1.76E-24
395	COX6A1	0.299108656	1.61E-12
396	SH3BGRL	-0.389346077	8.26E-21
397	LGALS3	-0.003642148	0.933019571
398	PPIA	0.450832692	3.83E-28
399	AHNAK	-0.335787912	1.45E-15
400	CTGF	-0.084653849	0.050349423
401	NCOA4	-0.555580644	1.17E-44
402	ARL6IP5	-0.286179431	1.53E-11
403	TMEM123	-0.445344732	2.00E-27
404	RPL18A	0.43699845	2.33E-26

405	RNF5	0.015322633	0.723634686
406	HDGF	0.2722955	1.50E-10
407	SNRPB	0.538528633	1.46E-41
408	IFITM1	0.28578838	1.63E-11
409	MORF4L2	0.061778545	0.15359059
410	VWF	-0.254417865	2.38E-09
411	TMED2	-0.36860628	1.16E-18
412	DDX5	0.195576841	5.18E-06
413	ITGB1	-0.397081915	1.19E-21
414	EHD2	0.146949963	0.000650727
415	PPAP2A	-0.186213007	1.46E-05
416	SOD2	-0.072299456	0.094807313
417	ARHGDIA	0.491002125	8.25E-34
418	RPL37	0.441161017	6.91E-27
419	TNIP1	0.103106322	0.017049469
420	NFKBIA	0.216508664	4.28E-07
421	HCFC1R1	0.497900313	7.34E-35
422	FLOT1	0.421141977	2.06E-24
423	MLEC	-0.32593936	1.05E-14
424	RPL28	0.562822636	4.99E-46
425	TMEM37	-0.024849773	0.566291911
426	YWHAZ	-0.082791206	0.055649087
427	TGOLN2	-0.492146561	5.54E-34
428	PLXNB2	0.173931761	5.24E-05
429	MAOB	-0.091179615	0.034991963
430	CXCR4	0.218458091	3.34E-07
431	COL3A1	-0.016612047	0.701448776
432	SRP9	-0.343657864	2.82E-16
433	TNFSF10	-0.093125494	0.031268204
434	OS9	-0.186139925	1.47E-05
435	TRMT112	0.60246521	3.69E-54
436	GNB1	-0.245462891	8.79E-09
437	ATP5G2	0.473278532	3.23E-31
438	CDC42	-0.366099309	2.06E-18
439	VCAM1	-0.021502002	0.619727308
440	TYROBP	0.379326942	9.42E-20
441	NACA	0.226727717	1.15E-07
442	PARK7	0.40689429	9.47E-23
443	GDI2	-0.335302107	1.60E-15
444	JUND	0.378573062	1.13E-19
445	GPI	0.135109411	0.001735292
446	RAB1A	-0.182858548	2.09E-05
447	PSMB7	0.275281098	9.30E-11
448	ANP32B	0.13077736	0.002438743
449	RGCC	-0.124670176	0.003875023
450	SURF4	0.018244054	0.673730095
451	CRYL1	-0.112149934	0.009427135
452	EIF6	0.5300926	4.27E-40
453	ANPEP	0.126128105	0.003475616
454	PRKCSH	0.446106859	1.59E-27
455	GNB2	0.373093979	4.10E-19
456	IDH2	0.040090384	0.354707723
457	HSPB8	0.085579139	0.047877725
458	APEX1	0.036775665	0.395925132
459	ACTR2	-0.324357973	1.43E-14
460	NCL	0.009005105	0.835380661
461	UQCRQ	0.265730687	4.25E-10
462	CREG1	-0.273336441	1.27E-10

463	REEP5	-0.346549973	1.53E-16
464	C5orf15	-0.171226546	6.87E-05
465	CDH16	-0.134750803	0.001785541
466	TMEM109	-0.103263711	0.016880494
467	RTN4	-0.116558187	0.006956959
468	TMED10	-0.47204686	4.83E-31
469	ANXA4	-0.147946301	0.000597133
470	C19orf43	0.619527451	5.05E-58
471	FBXL5	-0.420248513	2.63E-24
472	COL6A2	0.329341016	5.32E-15
473	KLF6	-0.128473726	0.002910829
474	GNS	-0.522561686	8.05E-39
475	CD9	-0.188433404	1.15E-05
476	SBDS	-0.22875972	8.80E-08
477	PSMB3	0.640554212	4.00E-63
478	GSTO1	0.309391425	2.48E-13
479	PSMB8	0.388234636	1.09E-20
480	SNRPD2	0.549419379	1.61E-43
481	MTCH1	0.022088957	0.610201237
482	LYZ	-0.090408922	0.036567874
483	CAPZB	0.345135825	2.07E-16
484	PPT1	-0.162261502	0.000163674
485	PDIA4	0.140349392	0.001134635
486	CPE	-0.039458679	0.362347038
487	CDKN1A	0.006375676	0.883032263
488	CA9	0.247739458	6.34E-09
489	AES	0.457541265	4.88E-29
490	PSMA7	0.305115016	5.45E-13
491	ID2	0.049388542	0.254126205
492	KDELR2	-0.035246177	0.415878484
493	CMBL	-0.090737554	0.035888664
494	DAZAP2	-0.46989944	9.71E-31
495	CERS2	-0.074009228	0.087232516
496	C4orf3	-0.440636838	8.06E-27
497	UQCR10	0.296890328	2.39E-12
498	RARRES3	0.211061549	8.39E-07
499	YWHAG	-0.156276358	0.000285022
500	TGFBR2	-0.423862517	9.70E-25
501	USMG5	0.212271058	7.24E-07
502	TRAPPC1	0.57016856	1.88E-47
503	RBM3	-0.039939318	0.356525296
504	ESM1	-0.1538408	0.000355205
505	IL32	0.458718664	3.38E-29
506	TMEM205	0.316801063	6.16E-14
507	JTB	0.45720276	5.42E-29
508	CALM1	-0.223556785	1.74E-07
509	RNF181	0.49661436	1.16E-34
510	CCNG1	-0.538469142	1.49E-41
511	CHMP2A	0.555952304	9.97E-45
512	GLTSCR2	0.313505738	1.15E-13
513	CTNNA1	-0.302694118	8.46E-13
514	SET	-0.175235123	4.60E-05
515	ARPC3	0.407877972	7.31E-23
516	TSC22D3	0.053068572	0.220397341
517	DNAJA1	-0.198931019	3.54E-06
518	HILPDA	0.084497445	0.050777579
519	COX4I1	0.313614903	1.13E-13
520	KIAA1191	-0.495917571	1.48E-34

521	TGFBI	-0.008222637	0.849506208
522	S100A16	0.477650767	7.66E-32
523	POLDIP2	0.281169168	3.54E-11
524	ASAH1	-0.37473332	2.80E-19
525	BNIP3L	-0.193481777	6.56E-06
526	EIF4A2	-0.187489172	1.27E-05
527	SLC6A8	0.06472021	0.134901616
528	VAMP5	0.440747972	7.80E-27
529	TM9SF2	-0.569199578	2.91E-47
530	SYPL1	-0.554698742	1.71E-44
531	SERPINF2	0.167746564	9.68E-05
532	APOL1	0.182634064	2.14E-05
533	AEBP1	0.061632747	0.154565532
534	2-Sep	-0.191519882	8.16E-06
535	TMEM27	-0.115616119	0.007429884
536	BHMT2	-0.032527749	0.452768249
537	CYC1	0.230544018	6.94E-08
538	SCD	-0.01354304	0.754635438
539	SCARB2	-0.558000989	4.11E-45
540	EIF2S3	-0.282220561	2.97E-11
541	RPL22	-0.184134247	1.82E-05
542	DUSP23	0.368873665	1.09E-18
543	IVNS1ABP	-0.117368217	0.006572073
544	ATRAID	0.268416532	2.79E-10
545	TCN2	-0.064824881	0.134270445
546	RPS15A	0.20617752	1.51E-06
547	FAM96B	0.546521762	5.43E-43
548	PSMB1	0.258431299	1.30E-09
549	S100A4	0.352044162	4.70E-17
550	LAMB2	-0.080255547	0.063600888
551	ADM	0.174628568	4.89E-05
552	ARF5	0.548040193	2.88E-43
553	DAB2	-0.10782914	0.012576504
554	EIF3K	0.313131406	1.23E-13
555	C19orf53	0.620645326	2.77E-58
556	CD164	-0.418963585	3.74E-24
557	PLP2	0.346397987	1.58E-16
558	AKR1B1	-0.090695226	0.035975541
559	NONO	-0.122147777	0.004665297
560	RPS26	0.260999127	8.82E-10
561	TLN1	-0.10558314	0.014555021
562	CYSTM1	0.081029658	0.061079656
563	PNRC1	-0.128441394	0.002918009
564	RHOC	0.448137947	8.66E-28
565	CNPPD1	0.022042769	0.610948484
566	CCT7	0.203584038	2.06E-06
567	PICALM	-0.479153224	4.64E-32
568	ZMAT2	-0.239825439	1.95E-08
569	BTG1	0.038035582	0.379929844
570	ARL6IP1	-0.235037227	3.78E-08
571	HNRNPF	-0.098704072	0.02241448
572	NUCKS1	-0.370363255	7.74E-19
573	MTPN	-0.512160649	4.12E-37
574	MVP	0.308846508	2.75E-13
575	TNS1	-0.275796863	8.55E-11
576	PPAP2B	-0.41685861	6.64E-24
577	HMGN2	-0.091216085	0.034918845
578	LAMP1	-0.122217686	0.004641567

579	GNG5	0.36032727	7.59E-18
580	CAT	-0.451147472	3.48E-28
581	PRDX3	-0.361775473	5.49E-18
582	ILF2	0.178805694	3.19E-05
583	CALM2	-0.159180038	0.000218308
584	ITM2C	0.06221572	0.150695169
585	PSMB6	0.534473289	7.48E-41
586	VCP	-0.009428357	0.827761594
587	IGBP1	-0.124675787	0.00387341
588	CUTA	0.319271974	3.84E-14
589	SLC39A1	0.191561077	8.13E-06
590	APH1A	-0.019408667	0.654213865
591	PPP1CA	0.463385488	7.81E-30
592	RNF187	0.224189456	1.60E-07
593	CALD1	-0.158357324	0.000235551
594	TUBA1B	-0.033096214	0.444905136
595	FLOT2	0.201243881	2.71E-06
596	POMP	0.162258075	0.000163727
597	ARRDC3	-0.10285301	0.017324537
598	THBS1	-0.218158381	3.47E-07
599	SERPINE1	0.168288067	9.18E-05
600	ARF4	-0.156771513	0.00027244
601	SMIM24	-0.060533251	0.162069337
602	NDUFA8	0.229374391	8.11E-08
603	ANXA7	-0.217053938	3.99E-07
604	CTNNB1	-0.40522362	1.47E-22
605	ANXA2	0.257266383	1.55E-09
606	FCGRT	0.213113985	6.52E-07
607	CA2	-0.334599112	1.84E-15
608	PIGT	0.02887297	0.50514866
609	ID3	0.157170922	0.000262672
610	CLSTN1	-0.174137304	5.14E-05
611	CAV1	-0.073924874	0.087594445
612	SEC61B	0.454384203	1.29E-28
613	MMP14	0.214305202	5.63E-07
614	DYNLL1	0.346153107	1.66E-16
615	WBP5	0.005198951	0.904505614
616	FABP3	-0.01850064	0.669410864
617	DYNLRB1	0.443154787	3.83E-27
618	GSN	-0.053752251	0.214501347
619	ARPC1A	0.041261763	0.340812659
620	CAPG	0.167782653	9.64E-05
621	CRIM1	-0.509220823	1.22E-36
622	XRCC5	-0.225627789	1.33E-07
623	CCT4	-0.267343955	3.30E-10
624	MEA1	0.435091013	4.05E-26
625	SLC25A1	0.466437475	2.96E-30
626	OGDH	-0.197637867	4.10E-06
627	TMBIM1	-0.103984967	0.016124758
628	TAGLN	0.106719332	0.01352233
629	PBXIP1	-0.079819066	0.065059877
630	H1F0	-0.106515532	0.01370267
631	CDC37	0.566665634	9.08E-47
632	DEGS1	0.126364271	0.003414554
633	BHMT	-0.094001316	0.029706119
634	CSTB	0.440295275	8.91E-27
635	TGM2	0.104350603	0.015753095
636	COL18A1	0.17605817	4.23E-05

637	SLC3A1	-0.316241803	6.85E-14
638	LAMP2	-0.487053726	3.22E-33
639	PCBP2	-0.038047875	0.379775751
640	SEPHS2	-0.100608873	0.019935726
641	SPINT2	0.004897152	0.910024854
642	CCT3	0.425869041	5.55E-25
643	PPP1CB	-0.491540895	6.84E-34
644	COX5A	0.099843908	0.020900956
645	FUCA1	-0.333601743	2.26E-15
646	CCT8	-0.158398236	0.000234665
647	EIF3D	0.094023822	0.029666877
648	FSTL1	-0.087216682	0.043752623
649	DDX17	0.105367676	0.014758495
650	NDUFB4	0.246215544	7.89E-09
651	ARF3	-0.281478175	3.36E-11
652	APMAP	-0.128710704	0.002858686
653	CRYZ	-0.135181682	0.001725324
654	FIS1	0.223257401	1.81E-07
655	POLR2L	0.450570696	4.15E-28
656	ZFP36L2	-0.08567302	0.047632722
657	UQCRH	0.013959461	0.7473438
658	C6orf62	-0.28843737	1.04E-11
659	HNRNPAB	0.466053847	3.34E-30
660	UBE2L6	0.048587262	0.261921677
661	MYDGF	0.498217845	6.55E-35
662	BCAM	0.067657378	0.118040648
663	JUP	-0.045903422	0.289224426
664	TAF7	0.036777874	0.395896747
665	HADHB	-0.369206302	1.01E-18
666	TSC22D1	-0.193963616	6.22E-06
667	PDIA6	-0.274818428	1.00E-10
668	HNRNPC	0.044259263	0.306863234
669	GLUL	-0.141427552	0.001037782
670	BCL2L1	0.221761984	2.19E-07
671	HERPUD1	-0.263038966	6.45E-10
672	CD99	0.339649752	6.52E-16
673	UBA1	0.188245271	1.17E-05
674	COL1A2	0.015284754	0.724290054
675	DAP	-0.155745121	0.000299123
676	PRKAR1A	-0.510863778	6.67E-37
677	EGLN3	0.005860627	0.892421497
678	HLA-DMA	0.283868926	2.25E-11
679	COL1A1	0.134535609	0.001816331
680	MAF1	0.506221461	3.68E-36
681	RRAS	0.596314259	8.00E-53
682	BTG2	-0.137499421	0.001432175
683	HLA-DQB1	0.151284297	0.000445987
684	MCAM	-0.011021838	0.799227272
685	MAPRE1	-0.230197061	7.26E-08
686	PLOD1	0.229043672	8.47E-08
687	JAK1	-0.395480264	1.79E-21
688	TIMP2	-0.073726568	0.088450059
689	KHDRBS1	-0.303552997	7.24E-13
690	GTF3C6	0.163963534	0.000139289
691	COL6A1	0.311226946	1.76E-13
692	AKR1A1	0.353800086	3.21E-17
693	ROMO1	0.567295388	6.85E-47
694	DARS	-0.207382457	1.31E-06

695	MDH2	-0.086045485	0.046671038
696	HSD17B10	0.51562668	1.13E-37
697	OSTC	0.015291208	0.724178373
698	TCEAL8	-0.314112005	1.03E-13
699	TMEM219	0.485304045	5.85E-33
700	CD14	0.245023805	9.36E-09
701	CLTA	0.31307068	1.25E-13
702	TMEM30A	-0.589196955	2.59E-51
703	STRAP	-0.285846359	1.61E-11
704	VAMP3	-0.466020492	3.38E-30
705	PHPT1	0.608319967	1.85E-55
706	TMED4	-0.206169973	1.51E-06
707	CLDN2	-0.008520091	0.844130467
708	SDC1	-0.011629668	0.788410747
709	METTL7A	-0.396365997	1.43E-21
710	TMEM140	-0.037095993	0.391820443
711	ZYX	0.481383639	2.20E-32
712	ERH	0.209533681	1.01E-06
713	COPG1	0.217084894	3.98E-07
714	ASS1	0.034713763	0.422960787
715	MRPL51	0.333537782	2.29E-15
716	NDUFB11	0.399451881	6.51E-22
717	UFC1	0.184915427	1.68E-05
718	PRDX4	0.05196716	0.230138023
719	MRPS34	0.544456881	1.28E-42
720	SLC2A1	0.012698556	0.769489084
721	MYADM	0.064025172	0.139150892
722	DPP7	0.510224476	8.46E-37
723	MLF2	0.482483507	1.52E-32
724	CTNND1	-0.401129686	4.23E-22
725	ASNA1	0.222081836	2.11E-07
726	PSMC3	0.508954123	1.35E-36
727	SMS	0.051386339	0.235395907
728	SAP18	0.032272475	0.456324549
729	EIF3E	-0.181327523	2.45E-05
730	CIRBP	0.040375477	0.351293495
731	ARCN1	-0.466886258	2.56E-30
732	FBL	0.407785524	7.49E-23
733	TSPAN12	-0.238359908	2.39E-08
734	NENF	0.468088704	1.74E-30
735	SLC40A1	-0.462409229	1.06E-29
736	LMBRD1	-0.516541191	7.99E-38
737	CAMK2N1	-0.214190752	5.71E-07
738	STOML2	0.207950176	1.22E-06
739	RAB10	-0.365216316	2.52E-18
740	ZNF395	-0.040590073	0.34873728
741	SERF2	0.543304821	2.06E-42
742	GNAI2	0.186166996	1.46E-05
743	GLO1	-0.452510494	2.30E-28
744	PCNP	-0.399675904	6.15E-22
745	AGRN	0.12884413	0.002829704
746	PODXL	-0.283354663	2.46E-11
747	ATP6V1E1	-0.122340269	0.004600219
748	SERINC2	0.187733755	1.24E-05
749	CTDSP2	-0.35976514	8.60E-18
750	PRPF6	0.225534459	1.35E-07
751	P4HA1	-0.241550498	1.53E-08
752	C6orf48	0.258634471	1.26E-09

753	HIBADH	-0.373547071	3.69E-19
754	EMC7	0.116234282	0.00711643
755	GSTK1	0.272616241	1.43E-10
756	BNIP3	-0.163103975	0.000151142
757	PSMB5	0.256196943	1.83E-09
758	GJA1	-0.234223181	4.22E-08
759	HPN	0.030855105	0.476352732
760	HNRNPU	-0.252399937	3.21E-09
761	CITED2	-0.346058033	1.70E-16
762	TSPO	0.51001823	9.12E-37
763	HMGN4	-0.316692396	6.29E-14
764	TPM4	0.15194449	0.000420672
765	TRIM28	0.405011247	1.55E-22
766	ACAA2	-0.251367189	3.74E-09
767	TUBA1A	0.171617408	6.61E-05
768	SERINC3	-0.465959805	3.44E-30
769	MAPKAPK2	0.269517381	2.34E-10
770	HNRNPH2	-0.33446793	1.89E-15
771	SLC44A2	-0.144105845	0.000829241
772	UBL5	0.40871478	5.86E-23
773	COPZ1	0.104129662	0.015976769
774	COPA	-0.26033955	9.76E-10
775	ALKBH5	0.117676487	0.006430689
776	NDUFA2	0.395591028	1.74E-21
777	DHRS3	0.045819391	0.290109064
778	EPHX1	-0.001368023	0.97481619
779	MARCKS	-0.140311477	0.001138188
780	CEBPD	0.36978965	8.84E-19
781	BANF1	0.568404599	4.16E-47
782	RPL14	0.31631295	6.76E-14
783	HBB	-0.064198333	0.138082738
784	LMNA	0.375812656	2.17E-19
785	PLD3	0.284351705	2.08E-11
786	MPC2	-0.060716341	0.160801112
787	ATP5A1	-0.285436148	1.73E-11
788	PMVK	0.284595536	1.99E-11
789	OCIAD2	0.161682959	0.000172837
790	TSPAN1	-0.075222578	0.082158464
791	PPP2R1A	0.32315577	1.81E-14
792	ERP29	0.295843287	2.87E-12
793	RPL39	0.234488009	4.07E-08
794	GALNT14	0.094648559	0.028595158
795	EFNA1	0.225910648	1.28E-07
796	SERPINH1	0.363006335	4.16E-18
797	LGMN	-0.23512872	3.73E-08
798	SRPR	0.12794846	0.003029502
799	PSMD8	0.375517452	2.32E-19
800	ADIPOR1	-0.183817238	1.88E-05
801	ESAM	-0.064078316	0.138822397
802	STAT1	-0.045143649	0.297288946
803	HRSP12	0.097347517	0.02433881
804	MAT2A	-0.060579944	0.161745192
805	ENPEP	-0.179311599	3.02E-05
806	GATM	-0.139618797	0.001204931
807	ATP6AP1	0.008354003	0.847131214
808	FKBP2	0.489938693	1.19E-33
809	LASP1	0.030829916	0.476712952
810	PCBD1	0.055814415	0.197406253

811	TRIM8	0.120312046	0.005328971
812	SUMO1	-0.030533973	0.480956313
813	CTDSP1	0.398567239	8.16E-22
814	AHCY	0.224325112	1.58E-07
815	RAMP3	-0.009681352	0.823215027
816	MIF	0.440374649	8.70E-27
817	YIPF3	0.122560027	0.004526926
818	TXN2	0.164173643	0.000136527
819	MARCKSL1	0.173944439	5.24E-05
820	NDUFA6	0.108343683	0.012158161
821	UQCRC1	0.085855438	0.047159674
822	RNF11	-0.617833844	1.25E-57
823	RALB	-0.233022117	4.97E-08
824	STC1	-0.07655703	0.076856391
825	SLC25A3	-0.240134502	1.87E-08
826	PRNP	-0.312808315	1.31E-13
827	SDCBP	-0.212712238	6.85E-07
828	TNFRSF1A	0.397733361	1.01E-21
829	TMEM230	-0.183109085	2.03E-05
830	ENPP2	-0.214156494	5.73E-07
831	PJA2	-0.609855985	8.36E-56
832	ATP5F1	-0.352760649	4.02E-17
833	SLC39A7	0.026240725	0.544757569
834	RAB13	0.38653358	1.65E-20
835	ATPIF1	0.383194488	3.72E-20
836	ERRFI1	-0.054047889	0.211987229
837	MRPS18B	-0.104607074	0.0154969
838	GOT1	-0.169885487	7.85E-05
839	APRT	0.511222285	5.84E-37
840	H2AFZ	0.106174651	0.01400905
841	IL6ST	-0.446604015	1.37E-27
842	C6orf106	-0.18706291	1.33E-05
843	BBOX1	-0.089674481	0.038125368
844	GPR56	-0.17246984	6.07E-05
845	SUMO2	0.096011727	0.026371574
846	RAB11B	0.415606375	9.31E-24
847	TMED7	-0.485833617	4.88E-33
848	SF3B6	0.199555699	3.29E-06
849	CLTC	-0.478449591	5.87E-32
850	SRRM2	0.187913066	1.21E-05
851	B4GALT1	-0.060519499	0.162164893
852	LAMTOR5	0.184491956	1.75E-05
853	PFKFB3	0.028051766	0.51734137
854	STAU1	-0.382353793	4.55E-20
855	GUK1	0.503219666	1.09E-35
856	ACSL1	-0.292442656	5.21E-12
857	ACO2	-0.32520902	1.21E-14
858	SQSTM1	0.046446628	0.283549549
859	LITAF	-0.285077563	1.84E-11
860	CNPY3	0.590105782	1.67E-51
861	WDR1	-0.041413866	0.339034241
862	ITGAV	-0.498349806	6.25E-35
863	GOLPH3	-0.341115311	4.81E-16
864	LMAN1	-0.469990393	9.43E-31
865	CTSA	0.236509348	3.09E-08
866	CDK2AP2	0.591199755	9.83E-52
867	TSPYL1	-0.610193599	7.01E-56
868	GADD45B	0.277285813	6.71E-11

869	LAMC1	-0.244826926	9.63E-09
870	NARS	-0.286606165	1.42E-11
871	KLHDC3	0.278689162	5.33E-11
872	ALDH9A1	-0.226346126	1.21E-07
873	AUP1	0.754943207	9.90E-100
874	SUMO3	-0.168342495	9.13E-05
875	BAG6	0.217716377	3.67E-07
876	NCSTN	-0.006386199	0.882840606
877	FAM129B	0.122448817	0.004563883
878	SRSF1	0.136530528	0.001548654
879	FOSL2	-0.073821704	0.088038751
880	IMPA2	-0.280897806	3.70E-11
881	IFNGR1	-0.147686547	0.000610696
882	ATN1	0.367943778	1.35E-18
883	SYNGR2	0.425695899	5.82E-25
884	TMEM50A	0.104499275	0.015604134
885	RRBP1	0.326746154	8.92E-15
886	CALU	-0.177963584	3.48E-05
887	MET	-0.26748903	3.23E-10
888	UXT	0.389763885	7.45E-21
889	ATP5H	0.274414114	1.07E-10
890	CCT6A	-0.003315631	0.939012083
891	NDUFB9	0.208308697	1.17E-06
892	HDLBP	-0.028222796	0.514789565
893	GNG12	-0.598772528	2.36E-53
894	FAM32A	0.351874439	4.88E-17
895	ETS2	-0.074588821	0.084778137
896	SYNPO	-0.028038531	0.517539112
897	PFDN2	0.701432412	2.01E-80
898	NDUFS4	-0.317917377	4.98E-14
899	RNF167	0.282209866	2.97E-11
900	SDC2	-0.407431116	8.22E-23
901	ENPP3	0.057671863	0.182878998
902	PFKL	0.175052837	4.68E-05
903	PLA2G16	0.019909276	0.645895733
904	AIP	0.458255632	3.91E-29
905	SHMT2	0.166849703	0.000105565
906	ETS1	-0.217068222	3.99E-07
907	LGALS2	0.169090265	8.49E-05
908	ADAR	-0.140577991	0.001113429
909	THY1	0.145849739	0.000715072
910	TMEM59	-0.478436126	5.90E-32
911	ATP5J	-0.04164325	0.336363493
912	TNFRSF12A	0.431160843	1.25E-25
913	RAD23A	0.354149626	2.97E-17
914	LEPROT	-0.426464599	4.70E-25
915	SUMF2	-0.122633493	0.00450266
916	MYO1C	0.103465011	0.016666514
917	CES2	-0.113179387	0.008789178
918	CTDNEP1	0.501816777	1.81E-35
919	IRF2BP2	-0.13138379	0.002326669
920	HNRNPDL	0.050893829	0.239920319
921	EIF4EBP2	-0.622938669	7.98E-59
922	EIF3L	-0.491683243	6.51E-34
923	SIRPA	0.11836781	0.006123499
924	SARS	-0.025323601	0.558910658
925	FAM127B	0.504950753	5.84E-36
926	IER3	0.252771097	3.04E-09

927	PAM	-0.343018317	3.23E-16
928	PCNA	0.079308805	0.066800272
929	PHC2	0.287772432	1.16E-11
930	FCER1G	0.276462614	7.67E-11
931	AOC1	-0.031057525	0.473463357
932	ANXA6	0.054938004	0.204545998
933	PRR13	0.146804655	0.000658906
934	LIPA	-0.337554145	1.01E-15
935	COX7A2	0.098118005	0.023229031
936	ATP5L	0.126763554	0.003313528
937	DIAPH1	-0.260127708	1.01E-09
938	CFI	-0.117451487	0.00653361
939	DDX3X	-0.303498419	7.32E-13
940	H1FX	0.517753044	5.05E-38
941	APOC1	0.292098318	5.53E-12
942	AHCYL1	-0.474691432	2.03E-31
943	ESD	-0.182107662	2.26E-05
944	TM4SF1	0.080101225	0.064113618
945	IFNGR2	0.290266075	7.59E-12
946	SSR2	0.325930872	1.05E-14
947	TMEM141	0.196920789	4.45E-06
948	CMPK1	-0.314049778	1.04E-13
949	STAT6	0.180926367	2.56E-05
950	PKN1	0.47174116	5.34E-31
951	DBI	0.306391775	4.32E-13
952	GNG11	-0.020992209	0.628053137
953	MGAT1	0.204427773	1.86E-06
954	HNRNPA3	-0.020133007	0.64219236
955	SPTBN1	-0.379708077	8.60E-20
956	TGFB1	0.274576725	1.04E-10
957	COA3	0.201659265	2.58E-06
958	GINM1	-0.366264303	1.99E-18
959	AP2B1	-0.385261483	2.25E-20
960	RAN	0.119944442	0.00547167
961	TMA7	0.423459591	1.09E-24
962	WASF2	-0.289262385	9.02E-12
963	PPP2R4	0.336369093	1.28E-15
964	RAB5C	-0.110548974	0.010501248
965	AQP3	0.018703045	0.66601137
966	CUEDC2	0.343448078	2.95E-16
967	UQCRC2	-0.312919845	1.28E-13
968	C1R	0.2171251	3.96E-07
969	TALDO1	0.336137197	1.35E-15
970	HAX1	0.289776501	8.26E-12
971	SNRPC	0.379048793	1.01E-19
972	ODC1	-0.08672878	0.04494916
973	C1S	0.144557614	0.000798147
974	NHP2L1	0.401002537	4.37E-22
975	FHL1	-0.110583094	0.010477268
976	SOCS3	0.191827171	7.89E-06
977	CHMP5	-0.292964568	4.76E-12
978	EIF4G1	0.090336182	0.036719681
979	BLVRB	0.371452554	6.02E-19
980	SDF4	0.36782854	1.39E-18
981	CD93	-0.314133037	1.02E-13
982	NRP1	-0.266522346	3.75E-10
983	TCEAL4	0.186966444	1.34E-05
984	ANXA1	0.05609629	0.195149052

985	CDK18	0.181464976	2.42E-05
986	PSMD4	0.531735596	2.23E-40
987	SMPDL3A	-0.184730644	1.71E-05
988	SSR3	-0.056422089	0.192563695
989	TTYH3	0.340502892	5.46E-16
990	NDUFC1	0.1500747	0.000496082
991	MMADHC	-0.388569038	1.00E-20
992	SLC39A14	-0.051963387	0.230171908
993	CTSO	-0.463919739	6.59E-30
994	RAMP2	0.068625822	0.112858057
995	FXYD2	0.014160214	0.743836547
996	TPM1	-0.087606582	0.042815814
997	SDHD	-0.406529145	1.04E-22
998	CX3CL1	-0.049175962	0.256178479
999	STARD7	-0.361675762	5.61E-18
1000	PIM3	0.385679979	2.03E-20
1001	COPS6	0.491638948	6.61E-34
1002	HIF1A	-0.274441125	1.07E-10
1003	CRTAP	-0.190198543	9.44E-06
1004	AAMP	0.327364457	7.89E-15
1005	HNRNPUL1	0.0639281	0.13975245
1006	DNAJB1	0.107687473	0.012693893
1007	SND1	-0.022562874	0.60255737
1008	NID1	-0.184060316	1.84E-05
1009	YPEL5	-0.365992183	2.11E-18
1010	MORF4L1	-0.401015582	4.36E-22
1011	AKR7A2	0.005040605	0.907400856
1012	KARS	0.056604383	0.19112812
1013	GIMAP4	-0.068629712	0.112837607
1014	TMEM248	-0.252127534	3.34E-09
1015	SNX17	0.230418489	7.05E-08
1016	MRPS35	-0.36382259	3.46E-18
1017	TTC1	-0.201525357	2.62E-06
1018	CENPB	0.342404236	3.67E-16
1019	TCEA3	0.036312283	0.401908569
1020	TPRG1L	-0.277834069	6.13E-11
1021	CDIPT	0.296861743	2.40E-12
1022	CREBL2	-0.598789325	2.34E-53
1023	SHISA5	0.382579085	4.31E-20
1024	GRB2	0.085315066	0.048572542
1025	NPTN	-0.449204247	6.28E-28
1026	TM7SF3	-0.268283827	2.85E-10
1027	UBXN1	0.50833037	1.70E-36
1028	COPE	0.591959589	6.79E-52
1029	PDZK1	-0.080716383	0.06208985
1030	TMX2	-0.257797232	1.44E-09
1031	GUCD1	0.042085768	0.331249432
1032	TMEM256	0.468653712	1.45E-30
1033	PEF1	0.261244184	8.50E-10
1034	NIPSNAP1	-0.125537416	0.003632705
1035	RHBDD2	0.359016439	1.02E-17
1036	PFDN1	0.122726205	0.004472204
1037	SHC1	0.264025537	5.54E-10
1038	SLC2A4RG	0.549017862	1.91E-43
1039	TPM3	-0.003292809	0.939431065
1040	MRPL15	0.017589324	0.684799877
1041	RAB6A	-0.505888411	4.15E-36
1042	PRPF19	0.373630997	3.62E-19

1043	DNASE2	0.149452403	0.000523848
1044	TSR2	0.010121371	0.815321611
1045	LAMTOR1	0.239204456	2.13E-08
1046	SLC16A4	-0.179185324	3.06E-05
1047	GSTA2	-0.128793309	0.002840711
1048	APOA1BP	0.259802337	1.06E-09
1049	CYBA	0.563623171	3.51E-46
1050	TSPAN9	-0.086048236	0.046663997
1051	HSPE1	0.377281682	1.53E-19
1052	AURKAIP1	0.524583104	3.69E-39
1053	CETN2	0.115107173	0.007697165
1054	CD46	-0.495681235	1.61E-34
1055	SF1	0.138113831	0.001362542
1056	TMEM43	-0.060419389	0.162861819
1057	RABAC1	0.508139969	1.82E-36
1058	SERPINB6	0.228391558	9.24E-08
1059	ADH5	-0.382897208	3.99E-20
1060	EPS8L2	0.334070195	2.05E-15
1061	COX14	0.309067609	2.64E-13
1062	DNAJB9	-0.368523394	1.18E-18
1063	FAM134A	-0.032475396	0.453496318
1064	SUPT4H1	0.133476363	0.001975101
1065	ARPC2	0.139051754	0.001262231
1066	LHFP	0.030109671	0.487075767
1067	OPTN	-0.006431824	0.882009675
1068	ERGIC3	0.299959599	1.38E-12
1069	PDAP1	0.456634352	6.47E-29
1070	UBXN6	0.306775306	4.02E-13
1071	GAL3ST1	0.328043972	6.89E-15
1072	NUDC	0.435408089	3.69E-26
1073	BEX4	-0.253957135	2.55E-09
1074	KLF10	-0.169126152	8.46E-05
1075	CXXC5	0.05388523	0.213367848
1076	NDUFA4	-0.04281282	0.322956574
1077	FCGR3A	0.059044092	0.172665334
1078	TM9SF3	-0.599811394	1.40E-53
1079	GAA	0.305055598	5.51E-13
1080	TIMM23	0.241358134	1.57E-08
1081	SF3B1	0.002256655	0.958469275
1082	NDFIP1	-0.498385868	6.17E-35
1083	OSTF1	-0.036311378	0.401920306
1084	RSL24D1	-0.255061881	2.16E-09
1085	TSR3	0.458677113	3.43E-29
1086	ATP1B3	0.140721112	0.001100339
1087	CRIP2	0.312356136	1.43E-13
1088	ITGA6	-0.486456601	3.95E-33
1089	NINJ1	0.446844477	1.28E-27
1090	FKBP10	0.30159488	1.03E-12
1091	FLT1	-0.294258604	3.79E-12
1092	IDH3B	0.235990694	3.32E-08
1093	PRELID1	0.369011307	1.06E-18
1094	COX7B	0.130781564	0.002437949
1095	MCFD2	-0.48029767	3.17E-32
1096	PTBP1	0.510616401	7.31E-37
1097	DNPH1	0.418480463	4.27E-24
1098	CDV3	-0.248074328	6.04E-09
1099	AARS	0.063829855	0.140363315
1100	AP2S1	0.550342899	1.09E-43

1101	PLOD2	-0.023871393	0.581678929
1102	IMPDH2	0.100190989	0.020458089
1103	MRPL24	0.53183599	2.14E-40
1104	NDUFV1	0.190274735	9.37E-06
1105	LSM14A	-0.19642219	4.71E-06
1106	CHMP1A	0.484426103	7.88E-33
1107	FBXW5	0.49457045	2.38E-34
1108	EMP3	0.557936567	4.23E-45
1109	CYB5A	-0.145410023	0.000742398
1110	TOMM22	0.132792646	0.002084226
1111	CHPF	0.299740844	1.44E-12
1112	CHD4	-0.115286953	0.007601784
1113	ZFAND5	-0.127030299	0.003247557
1114	C16orf13	0.568203543	4.56E-47
1115	PCYOX1	-0.600586048	9.51E-54
1116	SLC35A4	0.20777953	1.25E-06
1117	INSR	-0.274103263	1.13E-10
1118	SRSF2	0.520931825	1.51E-38
1119	ARF6	-0.15756807	0.000253284
1120	DGCR6L	0.448435486	7.92E-28
1121	AP1S1	0.197711836	4.07E-06
1122	SLC25A39	0.408773985	5.77E-23
1123	WBP2	0.274487454	1.06E-10
1124	LRRC32	-0.060325955	0.163514294
1125	EFCAB14	-0.533346656	1.17E-40
1126	GTF2F1	0.313761199	1.10E-13
1127	ENO2	0.265430979	4.45E-10
1128	HN1L	-0.071895623	0.096671024
1129	SLC38A2	-0.235564279	3.51E-08
1130	SGCB	-0.322389743	2.10E-14
1131	SLC3A2	0.198162232	3.86E-06
1132	LAMB1	0.075806159	0.079804462
1133	SPR	0.322198999	2.18E-14
1134	SERBP1	-0.372611213	4.59E-19
1135	DNAJC8	0.109517646	0.01124941
1136	ATP5J2	0.488737046	1.80E-33
1137	ADRM1	0.723464429	8.06E-88
1138	CHP1	-0.420164073	2.69E-24
1139	MTCH2	-0.235142102	3.72E-08
1140	CAPZA1	0.069453758	0.108570683
1141	LAP3	-0.150123848	0.000493949
1142	FOLR1	-0.077269885	0.074140091
1143	ITGA3	0.220581703	2.55E-07
1144	EI24	-0.216326541	4.37E-07
1145	IK	0.16987	7.86E-05
1146	SLC12A7	0.081671939	0.059050973
1147	COL23A1	0.123795144	0.004134268
1148	KHK	0.049248715	0.255474822
1149	EIF3H	-0.18756556	1.26E-05
1150	AGT	0.098131339	0.023210218
1151	H2AFV	-0.29805146	1.94E-12
1152	HIST1H2BD	0.229888506	7.57E-08
1153	DUSP3	-0.326026777	1.03E-14
1154	B4GALT5	-0.11131052	0.009977468
1155	UBXN4	-0.278872744	5.17E-11
1156	CCL2	0.278084574	5.88E-11
1157	GOLM1	-0.318577362	4.38E-14
1158	RTN3	-0.402755856	2.78E-22

1159	MRPL54	0.315457312	7.95E-14
1160	GABARAP	0.144157486	0.00082563
1161	YBX3	0.204495987	1.85E-06
1162	TNFRSF21	-0.069467776	0.108499214
1163	ATP5E	0.371847001	5.49E-19
1164	KDR	-0.339411631	6.85E-16
1165	DPP4	-0.312688405	1.34E-13
1166	SRSF6	0.250965526	3.96E-09
1167	CKB	0.030718637	0.478306126
1168	RAD23B	-0.379542573	8.94E-20
1169	PDGFRB	0.005855598	0.892513256
1170	SAT2	0.424750496	7.58E-25
1171	GPR116	-0.38037301	7.33E-20
1172	LPCAT1	0.156173349	0.000287707
1173	SRSF5	0.207623687	1.27E-06
1174	UBE2D2	-0.037005687	0.39297501
1175	TAP1	0.270795586	1.91E-10
1176	ANAPC16	-0.148311468	0.000578538
1177	PA2G4	0.491188794	7.73E-34
1178	C12orf57	0.416408456	7.50E-24
1179	EIF3G	0.509330133	1.18E-36
1180	MRFAP1L1	-0.057598822	0.183434906
1181	UBE2M	0.489212645	1.53E-33
1182	TCP1	-0.252656192	3.09E-09
1183	DRAP1	0.679362868	1.15E-73
1184	GTF3A	0.414023324	1.43E-23
1185	RAB5B	-0.32660629	9.17E-15
1186	GPAA1	0.326372408	9.60E-15
1187	QARS	0.226537106	1.18E-07
1188	DDRGK1	0.158589104	0.000230569
1189	GDI1	0.473262647	3.25E-31
1190	LMF2	0.60801453	2.17E-55
1191	TMEM147	0.41692986	6.51E-24
1192	PPP1R11	-0.104132955	0.015973415
1193	PSMC4	0.604821282	1.11E-54
1194	LBH	-0.061093995	0.15820883
1195	CTSS	-0.053292987	0.218449355
1196	PPP1R15A	0.277032204	6.99E-11
1197	SDHB	-0.142158789	0.000976498
1198	IL13RA1	-0.32802032	6.93E-15
1199	GSS	0.355856562	2.04E-17
1200	LAMTOR4	0.538198293	1.67E-41
1201	MGLL	-0.031013878	0.474085562
1202	PSMD2	0.058903956	0.173688456
1203	TRIP6	0.478197183	6.39E-32
1204	CCDC47	-0.481958185	1.81E-32
1205	CLEC14A	-0.169996972	7.76E-05
1206	ESYT1	-0.187224725	1.31E-05
1207	NDUFS2	0.026679888	0.538043799
1208	SERPINB1	-0.103037585	0.017123729
1209	ACKR3	0.142762098	0.000928471
1210	FUCA2	-0.031993608	0.46022738
1211	CHCHD10	0.168947567	8.61E-05
1212	PGRMC2	-0.577042636	8.11E-49
1213	HPCAL1	0.267407205	3.27E-10
1214	CBX3	0.0466505	0.28143923
1215	ARPC1B	0.486804862	3.50E-33
1216	SLC25A11	0.17320529	5.64E-05

1217	IER2	0.184907094	1.68E-05
1218	RAB14	-0.484621922	7.37E-33
1219	COPB1	-0.389053383	8.88E-21
1220	ATP6V1A	-0.467264084	2.27E-30
1221	ID1	0.169745049	7.96E-05
1222	THRAP3	-0.197247058	4.29E-06
1223	P3H2	-0.055026989	0.20381265
1224	EIF4EBP1	0.555722105	1.10E-44
1225	CREB3	0.259453094	1.12E-09
1226	CBR1	0.014913673	0.730721027
1227	NDUFB3	0.078102247	0.071067977
1228	SEC62	-0.451714149	2.93E-28
1229	TINAGL1	0.085965602	0.046875909
1230	MRPS21	0.501544749	2.00E-35
1231	DDX1	-0.292514937	5.14E-12
1232	KHSRP	0.27322351	1.30E-10
1233	UGT2A3	-0.200525078	2.94E-06
1234	BRD2	0.07729146	0.074059115
1235	PROS1	-0.107545965	0.012812115
1236	SEPP1	-0.563301942	4.04E-46
1237	STAT3	-0.128683405	0.002864649
1238	RBMX	-0.108255667	0.012228838
1239	LAPTM4B	-0.263870469	5.67E-10
1240	PGM1	-0.260168874	1.00E-09
1241	SSNA1	0.635347065	7.98E-62
1242	WDR83OS	0.434590438	4.67E-26
1243	TMEM9	0.226282009	1.22E-07
1244	ATP5D	0.505416764	4.93E-36
1245	GSR	-0.12279094	0.00445105
1246	TOB1	-0.258254996	1.34E-09
1247	ACTR1B	0.219081563	3.09E-07
1248	GABARAPL2	-0.25993303	1.04E-09
1249	CAPN2	-0.180153151	2.77E-05
1250	ISG15	0.551268465	7.37E-44
1251	DPYSL2	-0.161486901	0.00017605
1252	SFPQ	0.292094122	5.53E-12
1253	SEPN1	-0.00583151	0.892952745
1254	RNF10	0.142825763	0.000923532
1255	U2AF2	0.552067745	5.25E-44
1256	UBE2D3	-0.47952931	4.10E-32
1257	MRPS26	0.518704744	3.52E-38
1258	USP22	-0.29317178	4.59E-12
1259	DCTN2	0.331979618	3.13E-15
1260	EIF3A	-0.405514407	1.36E-22
1261	CAPRIN1	-0.468903584	1.34E-30
1262	RHOG	0.565995165	1.22E-46
1263	TRPC4AP	0.196038733	4.92E-06
1264	PNRC2	-0.361287781	6.12E-18
1265	GABARAPL1	-0.353072154	3.76E-17
1266	FH	-0.112950694	0.008927478
1267	DHX9	-0.315942841	7.25E-14
1268	ERLEC1	-0.355212585	2.35E-17
1269	HSD17B11	-0.20373048	2.02E-06
1270	NDUFAB1	-0.002469831	0.954550112
1271	TBCA	0.144446336	0.000805704
1272	KCNJ16	-0.155574535	0.000303788
1273	IARS2	-0.418439796	4.32E-24
1274	SLC28A1	0.099766912	0.021000323

1275	NOTCH3	-0.029998674	0.488683508
1276	CLPTM1	0.169783948	7.93E-05
1277	CTTN	0.314918576	8.81E-14
1278	LARP1	-0.076452829	0.077260136
1279	FKBP9	-0.260325656	9.78E-10
1280	VAMP2	-0.095582504	0.027055075
1281	MPC1	-0.313022359	1.26E-13
1282	GJA4	0.013902456	0.748340648
1283	ELOVL1	0.24365836	1.14E-08
1284	WDR45B	-0.027889875	0.519762817
1285	CTSF	-0.204819334	1.78E-06
1286	SLC38A1	-0.339909662	6.18E-16
1287	FAM120A	-0.355614771	2.16E-17
1288	MAGT1	-0.468999664	1.30E-30
1289	POLR2A	-0.126382466	0.00340989
1290	SZRD1	0.078330624	0.070243512
1291	GPNMB	-0.231349907	6.22E-08
1292	SEC31A	-0.266514869	3.76E-10
1293	WWTR1	-0.264737356	4.96E-10
1294	SLC9A3R2	0.149674203	0.00051379
1295	MRPL14	0.478780179	5.26E-32
1296	ARPP19	-0.603951234	1.74E-54
1297	MRPL18	0.350928451	5.98E-17
1298	SPINT1	-0.107926008	0.012496788
1299	POLR2E	0.385111062	2.33E-20
1300	DDAH1	-0.318707099	4.28E-14
1301	GOT2	-0.160347166	0.000195865
1302	PLEKHB2	-0.448986138	6.71E-28
1303	FURIN	0.110671068	0.010415665
1304	OCIAD1	-0.35408842	3.01E-17
1305	B4GAT1	-0.129196381	0.00275447
1306	LINC00493	0.250401726	4.30E-09
1307	PYGB	0.096784799	0.025178011
1308	RAB11A	-0.491974652	5.89E-34
1309	HLA-F	0.435966801	3.14E-26
1310	RBCK1	0.548141755	2.76E-43
1311	MDH1	-0.133894114	0.001911026
1312	EMD	0.450629112	4.08E-28
1313	CCL5	0.404249483	1.89E-22
1314	TIMM10	0.306365394	4.34E-13
1315	HLA-DQA1	0.081232716	0.060432153
1316	KLF9	-0.324401138	1.42E-14
1317	TMEM204	-0.014200361	0.74313579
1318	HNRNPM	-0.02169234	0.6166311
1319	CYS1	-0.179114951	3.09E-05
1320	MGEA5	-0.141212023	0.001056511
1321	SEPW1	0.310158954	2.15E-13
1322	GDF15	0.124602307	0.003894591
1323	UROD	0.435812139	3.29E-26
1324	CLPTM1L	0.080622844	0.062394134
1325	COX6C	0.261166521	8.60E-10
1326	GLS	-0.037354273	0.388529648
1327	PRPF8	-0.269385495	2.39E-10
1328	ATP6V0B	0.188563705	1.13E-05
1329	PGAM1	-0.28573183	1.65E-11
1330	EIF2AK1	0.012970169	0.764702175
1331	PSMF1	0.042735667	0.323830129
1332	BLVRA	0.207042582	1.36E-06

1333	VPS28	0.599058314	2.04E-53
1334	RTCB	-0.124472153	0.003932367
1335	SUN2	0.051786968	0.231760225
1336	SSR4	0.491529273	6.87E-34
1337	SEC61G	0.261246919	8.50E-10
1338	VWA1	0.105217161	0.014902114
1339	CXCL16	0.060273852	0.163878997
1340	ESYT2	-0.315898214	7.31E-14
1341	RSL1D1	-0.137573361	0.001423625
1342	NTN4	-0.284756407	1.94E-11
1343	ITGB5	-0.034520009	0.425555535
1344	EIF2A	-0.317424102	5.47E-14
1345	ERGIC1	-0.158155658	0.000239968
1346	SRSF3	0.075387472	0.081487707
1347	SERP1	-0.199188404	3.43E-06
1348	HIST1H2AC	0.029207029	0.50023233
1349	HIPK2	-0.395274769	1.88E-21
1350	OXA1L	0.186690032	1.38E-05
1351	PLTP	0.174972126	4.72E-05
1352	VDAC3	-0.120899402	0.005107913
1353	SMARCB1	0.167790079	9.64E-05
1354	ARHGAP1	0.152437789	0.000402637
1355	LRP10	-0.13868714	0.00130039
1356	CCT2	-0.076326743	0.077750979
1357	KPNB1	-0.056567158	0.191420629
1358	HNRNPH1	0.200082816	3.10E-06
1359	PKIG	0.171824213	6.48E-05
1360	ADPRHL2	0.439909511	9.97E-27
1361	BHLHE41	-0.092111867	0.033163312
1362	ARPC4	0.407499108	8.08E-23
1363	SNX10	0.008987212	0.835703113
1364	IQGAP1	-0.458585395	3.53E-29
1365	ATP6V0C	0.047443702	0.273329957
1366	SLC35B2	0.193233626	6.75E-06
1367	PSME2	0.593853365	2.69E-52
1368	TMEM259	0.628594734	3.56E-60
1369	EDNRB	-0.311161077	1.79E-13
1370	FUS	0.349788928	7.65E-17
1371	NOL3	0.3555584	2.18E-17
1372	MZT2B	0.584015823	3.09E-50
1373	ANXA11	0.061068449	0.158383178
1374	CCDC124	0.602704753	3.27E-54
1375	ECE1	0.020699894	0.632848618
1376	GADD45A	-0.141664939	0.001017508
1377	PTP4A2	-0.152546006	0.000398779
1378	MYC	0.180578505	2.65E-05
1379	GPR108	0.183152492	2.02E-05
1380	TP53INP2	-0.268976009	2.55E-10
1381	RTFDC1	0.015685925	0.717359393
1382	VASN	0.139244325	0.001242496
1383	LOX	-0.013078094	0.762802565
1384	CTSH	0.150482942	0.000478618
1385	NDUFS6	0.483264481	1.17E-32
1386	PPP2CA	-0.16934277	8.28E-05
1387	NDUFB6	-0.024076934	0.578430264
1388	C1QBP	0.092365135	0.032680836
1389	UQCR11	0.295643831	2.98E-12
1390	TOP1	-0.214908591	5.22E-07

1391	SCAMP2	0.11933961	0.005713956
1392	SPIN1	-0.337503481	1.02E-15
1393	DLST	-0.235834084	3.39E-08
1394	ARL1	-0.395755467	1.67E-21
1395	PPP4C	0.626555708	1.10E-59
1396	PTP4A1	-0.328541493	6.24E-15
1397	XRN2	-0.465027274	4.64E-30
1398	SLC17A3	0.039598517	0.360647162
1399	TMEM120A	0.402755421	2.78E-22
1400	DHCR24	-0.076762235	0.076066299
1401	ETF1	-0.28931426	8.94E-12
1402	HSPG2	-0.270461016	2.02E-10
1403	TPM2	0.403761669	2.14E-22
1404	ENSA	0.324302207	1.44E-14
1405	F2R	-0.166526572	0.000108918
1406	SF3B4	0.624699553	3.05E-59
1407	BCKDK	0.209285749	1.04E-06
1408	TMEM179B	0.257103772	1.59E-09
1409	TNFAIP1	-0.022179505	0.608737462
1410	GIMAP7	-0.009349309	0.829183367
1411	TMEM14A	-0.122458474	0.004560663
1412	S1PR1	-0.284507797	2.02E-11
1413	PAF1	0.359491553	9.14E-18
1414	NHP2	0.216135142	4.48E-07
1415	OAF	0.27853015	5.47E-11
1416	VMP1	0.446746531	1.32E-27
1417	TEX261	-0.116899246	0.00679251
1418	RAB2A	-0.267904185	3.02E-10
1419	PMP22	0.096701141	0.025304882
1420	CLTB	0.598316558	2.96E-53
1421	S100A9	0.143958703	0.000839607
1422	ANO6	-0.385640539	2.05E-20
1423	SNRPD3	0.307680091	3.41E-13
1424	ATP5O	0.133294374	0.002003625
1425	POLDIP3	-0.111432815	0.009895554
1426	ISCU	-0.066769125	0.122956086
1427	DGCR2	0.055108178	0.20314521
1428	COPS7A	0.17955768	2.95E-05
1429	KIF5B	-0.41114582	3.08E-23
1430	C11orf54	-0.298753076	1.72E-12
1431	MTIF3	0.19294865	6.96E-06
1432	METTL9	-0.326742204	8.92E-15
1433	ABHD14B	0.252414078	3.20E-09
1434	PHB	0.031916768	0.461306035
1435	SRSF4	0.390379689	6.40E-21
1436	SCAMP3	0.405780239	1.27E-22
1437	CDHR5	0.11240143	0.009267591
1438	ACTR1A	0.394123342	2.51E-21
1439	AGPAT2	0.39907014	7.18E-22
1440	RNF13	-0.493459119	3.51E-34
1441	USO1	-0.564171578	2.75E-46
1442	JAG1	-0.178227473	3.38E-05
1443	PDZD11	0.305494869	5.09E-13
1444	PEPD	-0.00513608	0.905655016
1445	ACAT1	-0.345643044	1.86E-16
1446	PPP2CB	-0.268624068	2.70E-10
1447	AIFM1	0.034146954	0.430577481
1448	TSPAN33	0.078330554	0.070243762

1449	VDAC2	-0.277800926	6.17E-11
1450	NBR1	-0.446591265	1.38E-27
1451	SLC35F6	0.132940877	0.00206011
1452	MSRB1	0.326364386	9.62E-15
1453	HSDL2	-0.304681162	5.90E-13
1454	EHHADH	-0.31307762	1.25E-13
1455	PSMC5	0.593893915	2.64E-52
1456	SDHA	-0.079353934	0.066644821
1457	RRAD	0.396932538	1.24E-21
1458	HNRNPD	0.109115191	0.011553933
1459	TMEM127	-0.202184601	2.42E-06
1460	MRPL37	-0.009824546	0.820644304
1461	CPQ	-0.346638697	1.50E-16
1462	MANBAL	0.255233314	2.11E-09
1463	BAG3	0.009053874	0.834501962
1464	VKORC1	0.468449443	1.55E-30
1465	PAIP2	-0.347859624	1.16E-16
1466	SLC37A4	0.195954829	4.97E-06
1467	MIDN	0.300994824	1.15E-12
1468	EFHD2	0.614043848	9.35E-57
1469	MOB1A	-0.31756467	5.32E-14
1470	GGT1	0.128021522	0.003012736
1471	CDKN1B	-0.206384532	1.48E-06
1472	TPD52L2	0.514450987	1.75E-37
1473	SLC47A1	-0.087034804	0.044195489
1474	COLGALT1	0.454438287	1.27E-28
1475	ADAM9	-0.438108825	1.69E-26
1476	MED29	0.084600965	0.050493854
1477	RBM42	0.620749884	2.61E-58
1478	LYPLA2	0.366333645	1.96E-18
1479	CD53	0.09690502	0.024996652
1480	FAM107B	-0.30902642	2.66E-13
1481	RBP7	-0.000807907	0.98512571
1482	STT3B	-0.447243758	1.13E-27
1483	SNRNP70	0.582494694	6.35E-50
1484	EPS8	-0.466278885	3.11E-30
1485	FAM168B	-0.404321618	1.85E-22
1486	IGJ	0.066655164	0.1235981
1487	NR1H2	0.472197928	4.60E-31
1488	9-Sep	0.155417433	0.000308143
1489	ATF6B	0.245748347	8.44E-09
1490	VASP	0.523259635	6.15E-39
1491	PACSIN2	-0.196210582	4.82E-06
1492	HES1	0.205387556	1.66E-06
1493	HEXB	-0.202203163	2.42E-06
1494	GOLGA7	-0.345326238	1.98E-16
1495	AIF1	0.205830549	1.58E-06
1496	AMFR	-0.377802221	1.35E-19
1497	CS	-0.243023963	1.24E-08
1498	SELT	-0.319585161	3.61E-14
1499	GAMT	0.316206539	6.90E-14
1500	HMGB1	-0.002380176	0.956198257
1501	DRG1	0.010627073	0.806272997
1502	TMEM214	0.386073927	1.85E-20
1503	MGAT4B	0.231146595	6.40E-08
1504	TNS3	-0.400347925	5.17E-22
1505	KRT19	0.11453778	0.008006347
1506	CHTF8	-0.355125454	2.40E-17

1507	NELFB	0.30125136	1.10E-12
1508	WARS	0.079448787	0.066319061
1509	SPRY1	-0.166153138	0.000112917
1510	TSPAN3	-0.131146971	0.002369863
1511	IFI27	0.281677443	3.25E-11
1512	ELTD1	-0.276348413	7.82E-11
1513	CASC4	-0.523379171	5.87E-39
1514	CORO1C	0.067950286	0.11645389
1515	PSMC2	0.000296352	0.99454362
1516	PARL	0.175058471	4.68E-05
1517	CDC42EP1	0.294834858	3.43E-12
1518	AK2	0.012795188	0.767785016
1519	CHMP1B	-0.377497831	1.46E-19
1520	TIMM8B	0.35528071	2.32E-17
1521	GLTP	-0.097908618	0.023526202
1522	NELFE	0.515143762	1.35E-37
1523	PERP	-0.250015641	4.55E-09
1524	UBE2Z	0.105858989	0.014298124
1525	NFE2L2	-0.230841789	6.66E-08
1526	IMMT	-0.237524124	2.68E-08
1527	CLSTN3	0.26431339	5.30E-10
1528	HNRNPL	0.375387301	2.40E-19
1529	PVRL2	0.333241891	2.43E-15
1530	GM2A	-0.107151854	0.013146509
1531	C19orf70	0.475958772	1.34E-31
1532	KCTD12	-0.483334148	1.14E-32
1533	PQBP1	0.68216335	1.72E-74
1534	RAB32	-0.005589525	0.897369751
1535	LCP1	0.029437856	0.496850089
1536	MAF	-0.091951876	0.033471217
1537	AK4	-0.128217342	0.002968214
1538	COMMD7	0.390106851	6.84E-21
1539	HSD17B14	0.345512656	1.91E-16
1540	NEU1	0.025499653	0.556180071
1541	S100A13	0.407280903	8.55E-23
1542	TNFSF12	0.379221497	9.66E-20
1543	FAM50A	0.703155526	5.61E-81
1544	HDAC1	0.180118396	2.78E-05
1545	HIST1H2BK	0.101142795	0.019285235
1546	ARL8A	0.326044951	1.02E-14
1547	ATP5G3	-0.019814027	0.647475033
1548	CRK	-0.196616054	4.61E-06
1549	AGPAT1	-0.009814671	0.820821524
1550	RETSAT	-0.199242792	3.41E-06
1551	C19orf33	0.379131913	9.87E-20
1552	WASL	-0.519244505	2.87E-38
1553	APOL2	0.29524919	3.19E-12
1554	EPRS	-0.3892097	8.54E-21
1555	SNRNP200	-0.127049176	0.003242934
1556	UCP2	0.241319032	1.58E-08
1557	SUCLG2	-0.506436409	3.40E-36
1558	NDUFA12	0.127139555	0.003220883
1559	NOL7	0.019657439	0.650074824
1560	MXI1	-0.264684652	5.00E-10
1561	FSTL3	0.371474552	5.99E-19
1562	MAPK3	0.117667166	0.006434924
1563	DYNLT1	0.332029391	3.10E-15
1564	RAD21	-0.312193357	1.47E-13

1565	ZNF358	0.512674715	3.41E-37
1566	TGFA	-0.173630927	5.40E-05
1567	PXDC1	0.361252837	6.17E-18
1568	MAT2B	-0.473810752	2.72E-31
1569	CYCS	-0.086771816	0.044842523
1570	APOLD1	-0.283992853	2.21E-11
1571	POLR2C	-0.14521983	0.000754515
1572	SH3GL1	0.562596067	5.52E-46
1573	HSPA4	-0.207736625	1.26E-06
1574	ZMPSTE24	-0.387256004	1.38E-20
1575	ICAM1	0.153065884	0.000380717
1576	KIF12	0.284450006	2.04E-11
1577	MYOF	-0.217892808	3.59E-07
1578	PNPLA2	0.373059089	4.14E-19
1579	RING1	0.387604406	1.27E-20
1580	FLII	0.363263451	3.92E-18
1581	ABHD12	0.297492204	2.15E-12
1582	CDC42SE2	-0.294617533	3.56E-12
1583	RNF145	-0.256149848	1.84E-09
1584	UBAP1	-0.12270071	0.004480561
1585	UGCG	0.011260619	0.794973383
1586	ALDH3A2	-0.250656422	4.15E-09
1587	MT1E	0.090627408	0.036115106
1588	CAMLG	-0.138053017	0.001369293
1589	NEK6	-0.130816938	0.002431281
1590	PXN	0.197965587	3.95E-06
1591	WFDC2	-0.050117286	0.247177532
1592	PPM1G	0.460906914	1.71E-29
1593	SNRPE	0.201708599	2.56E-06
1594	MRPL17	0.453518234	1.69E-28
1595	FXYD5	0.459517292	2.64E-29
1596	SNX12	-0.264715149	4.98E-10
1597	CLRN3	0.051718341	0.232380174
1598	NAA20	0.01660063	0.701644154
1599	DNAJC3	-0.53614904	3.81E-41
1600	ARFGAP3	-0.247008277	7.04E-09
1601	EIF2S2	0.057416514	0.184827891
1602	LDOC1	0.177853894	3.52E-05
1603	RSU1	-0.007157669	0.868808413
1604	ZFAND3	-0.284867566	1.90E-11
1605	AIF1L	-0.260568424	9.42E-10
1606	MFN2	-0.259543372	1.10E-09
1607	GADD45GIP1	0.621195726	2.05E-58
1608	COTL1	0.34749905	1.25E-16
1609	PRRC2A	0.150123468	0.000493965
1610	C8orf4	-0.134582975	0.001809512
1611	MIOX	0.108989499	0.011650522
1612	ZDHHC5	-0.200904036	2.81E-06
1613	CPNE1	0.526977797	1.45E-39
1614	PPP1R14B	0.561486799	8.99E-46
1615	NCK2	0.07852098	0.069562303
1616	MKNK2	0.277622349	6.35E-11
1617	BCAS2	-0.392265246	4.00E-21
1618	POU3F3	-0.064622371	0.135493656
1619	TCTA	-0.036292912	0.402159869
1620	CD34	-0.184614861	1.73E-05
1621	ZNF768	0.339637402	6.54E-16
1622	HNRNPH3	-0.039793477	0.358285554

1623	MBNL2	-0.412029606	2.43E-23
1624	LAMTOR2	0.656791275	2.40E-67
1625	HTATSF1	-0.3060353	4.61E-13
1626	HK1	-0.095815388	0.026682359
1627	CDH5	-0.160578299	0.000191685
1628	WDR34	0.468275058	1.64E-30
1629	SDPR	-0.285856034	1.61E-11
1630	PLXND1	0.076840391	0.075767124
1631	CCDC28A	-0.294080363	3.91E-12
1632	NAP1L4	0.28806277	1.11E-11
1633	USP5	0.240657391	1.74E-08
1634	BET1L	0.151884029	0.000422933
1635	FZD4	-0.243602538	1.15E-08
1636	POLD2	0.375071945	2.58E-19
1637	SNX2	-0.541935455	3.62E-42
1638	IPO7	-0.524119802	4.41E-39
1639	RPA2	-0.027191471	0.530275497
1640	SNRPN	-0.063301863	0.143681359
1641	PROCR	0.213927326	5.90E-07
1642	UFM1	-0.241625926	1.52E-08
1643	UBE2Q1	0.20279129	2.26E-06
1644	PTPRF	-0.126497642	0.003380501
1645	DDX41	0.348584267	9.91E-17
1646	APLN	-0.081783552	0.058704187
1647	ASB13	0.069912974	0.106248688
1648	SPCS3	-0.385038359	2.38E-20
1649	DRAM1	-0.039185675	0.365680094
1650	USH1C	0.13096113	0.002404271
1651	SLC22A2	-0.138012469	0.001373811
1652	NME3	0.663984837	2.67E-69
1653	MRPL45	-0.248502598	5.67E-09
1654	FLNB	-0.218469546	3.34E-07
1655	SLCO2A1	-0.237888808	2.55E-08
1656	ADD1	-0.211153431	8.30E-07
1657	FAHD1	-0.138820274	0.001286336
1658	PRKCDBP	0.485418749	5.62E-33
1659	HP1BP3	-0.147844772	0.000602401
1660	SLC6A13	0.040143855	0.354065772
1661	SLC16A3	0.312186949	1.47E-13
1662	UBE2B	-0.262586835	6.92E-10
1663	DHRS7	-0.222377805	2.03E-07
1664	AKR1C3	0.142088197	0.000982265
1665	NR2F2	-0.229551814	7.92E-08
1666	OSER1	-0.026954695	0.533863833
1667	DECR1	-0.071283909	0.099549647
1668	UBE2H	-0.283662454	2.33E-11
1669	SCCPDH	-0.265872982	4.16E-10
1670	FBP1	0.069781806	0.106907889
1671	TCTN3	-0.347196155	1.33E-16
1672	SPG21	-0.164409386	0.00013349
1673	RNPEP	0.300181534	1.33E-12
1674	DERA	-0.063117446	0.144854314
1675	IFIT3	-0.132359625	0.002156163
1676	ELOVL5	-0.09400395	0.029701525
1677	SURF1	0.245974638	8.17E-09
1678	TYMP	0.582982043	5.04E-50
1679	DSG2	-0.461000837	1.66E-29
1680	DDAH2	0.468441082	1.55E-30

1681	M6PR	-0.409692219	4.53E-23
1682	DCTD	-0.125354181	0.003682728
1683	IRAK1	0.26460446	5.06E-10
1684	UBE2L3	0.245811517	8.36E-09
1685	ZBTB4	-0.352136522	4.61E-17
1686	ITGA5	0.133618389	0.0019531
1687	MRPL3	-0.10048967	0.020083535
1688	SMIM15	-0.307787673	3.34E-13
1689	UBE2J1	-0.280506041	3.95E-11
1690	PPP1R3C	-0.115508585	0.007485655
1691	POLR2G	0.408275043	6.59E-23
1692	CLEC3B	-0.05245717	0.225767411
1693	ADI1	-0.094545763	0.028769182
1694	MAN1A1	-0.493485874	3.47E-34
1695	ALDH18A1	0.044790749	0.30108527
1696	DEK	-0.338766838	7.83E-16
1697	GUSB	0.23310648	4.91E-08
1698	CFDP1	0.064583527	0.135729262
1699	ARSE	-0.034310906	0.428366197
1700	SKP1	-0.449247096	6.20E-28
1701	ANAPC13	-0.16373507	0.000142351
1702	FGL2	-0.250636052	4.16E-09
1703	KCTD3	-0.026368748	0.542796125
1704	RCC2	0.402656882	2.85E-22
1705	FAM167B	0.129379889	0.002716003
1706	GYS1	0.133783961	0.001927734
1707	EDN1	-0.139877576	0.001179586
1708	C11orf68	0.376144804	2.00E-19
1709	PLBD2	0.040933139	0.344675301
1710	TMEM47	-0.374550533	2.92E-19
1711	SH3GLB1	-0.382554002	4.34E-20
1712	SETD3	-0.215874767	4.63E-07
1713	PTPN1	0.047538455	0.272372012
1714	SLC25A23	-0.170718655	7.23E-05
1715	SLC39A6	-0.290436917	7.37E-12
1716	TMEM203	0.14990631	0.000503457
1717	CAPN1	0.066822361	0.122657065
1718	CAST	-0.3730627	4.13E-19
1719	SNW1	-0.051069658	0.238298113
1720	CYP27A1	0.179264509	3.04E-05
1721	ADD3	-0.400619501	4.83E-22
1722	GLG1	-0.324954898	1.27E-14
1723	LSM2	0.3808681	6.51E-20
1724	MRPL49	-0.282112624	3.02E-11
1725	TMEM167A	-0.416818951	6.71E-24
1726	EDEM2	0.374272952	3.11E-19
1727	MRPL28	0.508623003	1.53E-36
1728	SSRP1	0.029652223	0.493719955
1729	FRZB	-0.018746023	0.665290407
1730	SMG5	0.239978989	1.91E-08
1731	ARL8B	-0.443736823	3.23E-27
1732	PITPNA	-0.206049517	1.54E-06
1733	EGFR	-0.333087141	2.50E-15
1734	TMEM54	0.475800848	1.41E-31
1735	ALKBH7	0.388585814	9.97E-21
1736	PTPRA	-0.3255666	1.13E-14
1737	DPM3	0.572360074	6.96E-48
1738	TERF2IP	-0.012901838	0.765905607

1739	LTBR	0.490200868	1.09E-33
1740	RNF114	-0.034414742	0.426969136
1741	ZNF664	-0.493438281	3.53E-34
1742	HSD3B7	0.473643777	2.87E-31
1743	POLR2K	-0.085008211	0.04939051
1744	MAGED1	0.064531718	0.136043996
1745	CCT5	0.130558016	0.002480478
1746	PKD2	-0.365483444	2.37E-18
1747	PPP1R9B	0.364041019	3.29E-18
1748	GOLIM4	-0.303038215	7.95E-13
1749	C11orf31	0.481424087	2.17E-32
1750	DYNC1H1	-0.239507036	2.04E-08
1751	BOD1	-0.10758985	0.012775348
1752	UBE2R2	-0.053253297	0.218792973
1753	SUCLG1	-0.056702359	0.190359801
1754	STT3A	-0.216797231	4.12E-07
1755	RAB20	0.196957511	4.43E-06
1756	SHMT1	0.007744786	0.858156707
1757	PGD	-0.022440873	0.604521021
1758	KRCC1	-0.135088975	0.00173812
1759	MEPCE	0.063216502	0.144223376
1760	USF2	0.562152421	6.71E-46
1761	FAM162A	0.019665203	0.649945824
1762	CPVL	-0.204165107	1.92E-06
1763	SPTAN1	-0.162292214	0.000163201
1764	FSCN1	0.266399204	3.83E-10
1765	CP	-0.112286547	0.00934017
1766	PTGR1	-0.248598103	5.60E-09
1767	SRP72	-0.465791986	3.63E-30
1768	EGFL7	0.392353854	3.91E-21
1769	DUSP6	-0.050890488	0.239951214
1770	SUSD6	-0.263455638	6.05E-10
1771	DYNC1I2	-0.359600576	8.92E-18
1772	BDH2	-0.156803651	0.000271642
1773	ALDOC	-0.002245401	0.958676206
1774	VPS29	-0.014816479	0.732408573
1775	ENOPH1	-0.26261811	6.88E-10
1776	METTL7B	0.299153898	1.60E-12
1777	OSMR	-0.139255682	0.001241341
1778	SEC23B	-0.342619974	3.51E-16
1779	NFIL3	0.093267437	0.031010367
1780	YKT6	0.31744678	5.44E-14
1781	IDH3G	0.491661635	6.56E-34
1782	TOB2	-0.147304585	0.00063116
1783	KIAA2013	0.307727027	3.38E-13
1784	DPM1	-0.108469135	0.012058048
1785	FAM134B	-0.232106243	5.62E-08
1786	MTDH	-0.390468386	6.26E-21
1787	UQCRB	0.004701326	0.913608412
1788	CD248	0.21336449	6.32E-07
1789	TES	-0.255170576	2.13E-09
1790	SEMA3F	0.034902057	0.420448043
1791	TOMM70A	-0.249528888	4.89E-09
1792	CKAP4	0.193933169	6.24E-06
1793	SRI	-0.204888313	1.76E-06
1794	BAD	0.504496187	6.89E-36
1795	EXOC3L2	-0.008184413	0.850197523
1796	MOAP1	-0.254087837	2.50E-09

1797	RAB11FIP3	0.245875527	8.29E-09
1798	PON2	0.122122062	0.004674054
1799	C12orf75	-0.024367981	0.573844726
1800	SULF2	0.049544596	0.252626918
1801	CSE1L	-0.276110681	8.13E-11
1802	SCARB1	0.152785895	0.000390347
1803	HMGB2	0.295369796	3.12E-12
1804	TNS2	0.125294134	0.003699256
1805	CIAO1	-0.058343781	0.177823384
1806	TMEM106C	-0.129818801	0.002625975
1807	TPMT	-0.126693481	0.003331059
1808	MRPS16	-0.044051394	0.309142845
1809	WSB2	-0.217923742	3.58E-07
1810	PNMA1	-0.044459574	0.304677048
1811	SF3A1	-0.30809362	3.16E-13
1812	GNPTG	0.487192185	3.07E-33
1813	NAP1L1	-0.08040452	0.06310914
1814	FAM213A	-0.280778399	3.77E-11
1815	COA4	0.494040649	2.86E-34
1816	STMN3	0.378832174	1.06E-19
1817	STIP1	0.26572559	4.25E-10
1818	SCYL1	0.510243216	8.40E-37
1819	MPDU1	0.239869236	1.94E-08
1820	MAP1LC3B	-0.312626902	1.36E-13
1821	NSA2	-0.163307831	0.000148248
1822	DGUOK	0.653167459	2.22E-66
1823	SGTA	0.531237589	2.72E-40
1824	RCAN2	-0.335864057	1.42E-15
1825	TMEM51	0.073639379	0.088828367
1826	ZNF622	0.062883767	0.146351078
1827	KIF3B	-0.50710687	2.66E-36
1828	CD2BP2	0.400329905	5.20E-22
1829	SCP2	-0.455984114	7.91E-29
1830	ANP32E	-0.176680211	3.97E-05
1831	LSR	0.221436571	2.29E-07
1832	CAPZA2	-0.473236381	3.28E-31
1833	COPRS	0.185136797	1.64E-05
1834	SON	-0.292029712	5.59E-12
1835	ERBB3	-0.07143936	0.098811736
1836	SAMHD1	-0.048374729	0.26401672
1837	PAK2	-0.332377692	2.89E-15
1838	CUBN	-0.158859936	0.000224872
1839	ACADS	0.088810202	0.040029899
1840	TMEM9B	-0.349600695	7.97E-17
1841	POLE3	-0.157215136	0.000261611
1842	FBXO17	0.222080773	2.11E-07
1843	TM9SF4	-0.102262156	0.017981265
1844	WBP1L	-0.316393374	6.66E-14
1845	DDR1	-0.251907301	3.45E-09
1846	LRRC59	0.142417164	0.000955656
1847	UBA2	-0.158980529	0.000222378
1848	CYP2J2	-0.04288349	0.322157761
1849	TXNDC12	0.091447823	0.034457268
1850	ZBED1	0.015083348	0.727778132
1851	MAPK1	-0.468569853	1.49E-30
1852	PEX11B	-0.149930697	0.000502383
1853	GLYR1	-0.202784425	2.26E-06
1854	CWC15	0.335985032	1.39E-15

1855	HIGD1A	-0.328592112	6.18E-15
1856	LRP2	-0.202090269	2.45E-06
1857	GMPR2	-0.138431244	0.001327802
1858	OTUD5	0.353938034	3.11E-17
1859	COMMD6	0.135929137	0.001625263
1860	PSMD13	0.602521157	3.58E-54
1861	CDC42SE1	0.076601464	0.076684744
1862	MRPL40	0.442249546	5.01E-27
1863	ACY3	0.204845693	1.77E-06
1864	ATP6V1B2	-0.156964025	0.00026769
1865	FKBP4	0.048255638	0.26519568
1866	ATP2A2	-0.282025941	3.07E-11
1867	ADAMTS1	-0.063730073	0.140985822
1868	GNA13	-0.365066283	2.61E-18
1869	PPCS	-0.15155545	0.000435423
1870	MRPL34	0.209506091	1.01E-06
1871	RNH1	0.322389775	2.10E-14
1872	YME1L1	-0.464656899	5.22E-30
1873	FARSA	0.363686793	3.57E-18
1874	G3BP2	-0.585903247	1.26E-50
1875	OSBP	-0.487348328	2.91E-33
1876	SPRYD3	-0.141348437	0.001044621
1877	HYOU1	-0.09193603	0.033501846
1878	PFN2	-0.226604439	1.17E-07
1879	EFNB2	-0.216288959	4.40E-07
1880	MAP4	-0.118804751	0.005936205
1881	PSMD3	0.392212931	4.05E-21
1882	SMDT1	0.146777098	0.000660468
1883	FZD1	-0.163891198	0.000140252
1884	ENDOD1	-0.521811989	1.07E-38
1885	MMRN2	-0.238580122	2.32E-08
1886	LONP1	0.35224581	4.50E-17
1887	SMIM3	-0.000544375	0.989977263
1888	STX12	-0.480837586	2.64E-32
1889	IST1	-0.13566523	0.001659967
1890	CNOT11	0.003149006	0.942071481
1891	FAM20C	0.272079453	1.56E-10
1892	HNF1B	-0.078289	0.070393192
1893	TMEM208	0.506909737	2.86E-36
1894	CPNE3	-0.625589443	1.87E-59
1895	PPIF	-0.006450362	0.881672088
1896	GMFG	0.413223369	1.77E-23
1897	DNAJC1	-0.040905766	0.344998288
1898	RDX	-0.466182874	3.21E-30
1899	CARHSP1	0.288790486	9.78E-12
1900	SNAPIN	0.27041167	2.03E-10
1901	CAV2	-0.185499644	1.57E-05
1902	AKAP12	-0.15948715	0.00021218
1903	RAPGEF1	-0.109077162	0.011583081
1904	G0S2	0.134594579	0.001807845
1905	TIMM13	0.456294096	7.19E-29
1906	ARAF	-0.014831339	0.732150488
1907	HYAL2	0.172106683	6.30E-05
1908	TRAPPC6A	0.31902638	4.02E-14
1909	EIF3B	0.29865472	1.75E-12
1910	GARS	0.158730055	0.000227587
1911	NES	-0.084494406	0.050785929
1912	TBC1D9B	0.010594455	0.806855875

1913	LDB1	-0.015926351	0.713216818
1914	DCAF7	-0.343985159	2.63E-16
1915	PROSC	-0.327926021	7.06E-15
1916	DYNLL2	-0.33443757	1.90E-15
1917	PPP1CC	-0.348863636	9.33E-17
1918	AMOTL2	-0.110369492	0.010628189
1919	MAGEF1	0.081994042	0.05805478
1920	AHSA1	0.316888547	6.06E-14
1921	TMEM115	0.319267902	3.84E-14
1922	RNF7	0.368062724	1.32E-18
1923	PLSCR1	-0.114401596	0.008081919
1924	CYFIP2	-0.389195733	8.57E-21
1925	PLOD3	0.433179991	7.01E-26
1926	IGSF8	0.267531997	3.20E-10
1927	PWP1	-0.203189967	2.15E-06
1928	AP1B1	0.080082011	0.064177692
1929	MRPL44	-0.212104935	7.38E-07
1930	GAS6	-0.005364267	0.90148429
1931	EFEMP1	-0.133744234	0.001933793
1932	PLEC	0.333337044	2.38E-15
1933	SPOCK2	0.087088368	0.044064673
1934	HOXC10	0.018894489	0.662802257
1935	IFNAR1	-0.47515925	1.74E-31
1936	GALM	0.142305743	0.000964593
1937	TTC38	0.203149174	2.17E-06
1938	TRIB2	-0.060017439	0.165682714
1939	AXL	0.040940086	0.344593362
1940	GPR137B	-0.176071442	4.22E-05
1941	KANK2	-0.010589809	0.806938899
1942	PUM2	-0.414724264	1.18E-23
1943	TM4SF18	-0.290956165	6.74E-12
1944	CRAT	0.001268406	0.976649489
1945	RBM39	0.318216031	4.70E-14
1946	MMP7	-0.023504286	0.587502301
1947	LEPROTL1	-0.127638122	0.003101668
1948	CCNY	-0.389816921	7.35E-21
1949	TMEM159	-0.152512177	0.000399981
1950	A4GALT	0.332783216	2.66E-15
1951	ACP1	-0.208338531	1.17E-06
1952	TMCO1	-0.156635356	0.000275847
1953	SP1	-0.210466879	9.02E-07
1954	POP7	0.590484217	1.39E-51
1955	RBPMS	0.17077363	7.19E-05
1956	SRSF7	0.353086397	3.75E-17
1957	API5	-0.58909259	2.73E-51
1958	NUTF2	0.572715333	5.92E-48
1959	JMJD8	0.368162623	1.29E-18
1960	ERI3	0.169436649	8.20E-05
1961	ECHDC3	-0.087607832	0.042812837
1962	IFI35	0.528154482	9.16E-40
1963	MFSD10	0.642506904	1.28E-63
1964	PMEPA1	0.051170527	0.237370985
1965	HIAT1	-0.430569716	1.48E-25
1966	PLIN3	0.170033827	7.73E-05
1967	AFF1	-0.379677347	8.66E-20
1968	HNMT	-0.3161742	6.94E-14
1969	ZNF32	0.349722294	7.76E-17
1970	SLC29A1	-0.118064024	0.006256825

1971	SEC11A	-0.156371379	0.000282566
1972	TMX4	-0.629041892	2.78E-60
1973	GHDC	0.137653216	0.001414444
1974	PLK2	0.094479722	0.028881463
1975	NME4	0.514990366	1.43E-37
1976	CBX1	-0.128809979	0.002837096
1977	MICU1	-0.142756976	0.00092887
1978	NDUFAF3	0.46188067	1.26E-29
1979	AGTRAP	0.458134002	4.06E-29
1980	SORBS3	0.346530995	1.54E-16
1981	FAM174A	-0.290272734	7.58E-12
1982	RARS	-0.229379925	8.10E-08
1983	GNPAT	-0.066567651	0.124092884
1984	SRM	0.662779283	5.72E-69
1985	PSMA3	0.229475706	8.00E-08
1986	RPS29	0.129692092	0.002651681
1987	CLDN4	-0.122169549	0.004657895
1988	CLINT1	-0.456578285	6.58E-29
1989	BICC1	-0.24954666	4.88E-09
1990	CLPP	0.467624017	2.02E-30
1991	RNASET2	0.257528156	1.49E-09
1992	POLR2J	0.602935352	2.91E-54
1993	UBE2E3	-0.114907463	0.007804371
1994	SF3B2	0.089222701	0.039111104
1995	NRAS	-0.353207066	3.65E-17
1996	NUPR1	0.355354954	2.28E-17
1997	MRPL20	0.372930855	4.26E-19
1998	ICMT	-0.120958491	0.005086138
1999	TAF15	0.220444227	2.60E-07
2000	SNRPB2	0.055845378	0.197157374
2001	DDB1	-0.359131303	9.91E-18
2002	ILF3	0.302296217	9.09E-13
2003	VPS25	0.210785059	8.68E-07
2004	RUVBL2	0.596262639	8.21E-53
2005	PTPN12	-0.168034463	9.41E-05
2006	C1orf115	-0.135933911	0.001624642
2007	THOC7	0.093357283	0.030848103
2008	SLMO2	-0.255995202	1.88E-09
2009	ANGPTL2	0.004418285	0.918791052
2010	EGLN1	-0.462777951	9.46E-30
2011	RGS1	0.20131196	2.68E-06
2012	TDP2	-0.298786432	1.71E-12
2013	ALPL	0.042863534	0.322383198
2014	TWF2	0.481984866	1.80E-32
2015	PAFAH1B1	-0.470501016	7.99E-31
2016	FOXJ3	-0.146097667	0.000700078
2017	SLC1A5	0.448775426	7.15E-28
2018	HSPA1A	0.345283852	2.00E-16
2019	B3GNT2	-0.405329672	1.43E-22
2020	GBP2	0.156330752	0.000283614
2021	VAMP7	-0.506206577	3.70E-36
2022	CD40	0.227334777	1.06E-07
2023	ESRRA	0.171190098	6.90E-05
2024	UBL7	0.330026301	4.64E-15
2025	RAB34	0.295549923	3.03E-12
2026	SLC4A4	-0.281333921	3.44E-11
2027	ECI1	0.336682565	1.20E-15
2028	KEAP1	0.377842868	1.34E-19

2029	STX5	0.205082284	1.72E-06
2030	TAX1BP3	0.328205601	6.67E-15
2031	MUL1	-0.179608271	2.93E-05
2032	NET1	-0.092454491	0.032512048
2033	CISD2	-0.176778744	3.93E-05
2034	BLOC1S1	0.508765654	1.45E-36
2035	UBL4A	0.211980106	7.50E-07
2036	INSIG2	-0.278568554	5.44E-11
2037	SLC38A10	0.367508517	1.49E-18
2038	CSRP1	0.054108781	0.211472044
2039	MAPRE2	-0.344617939	2.30E-16
2040	SLC25A28	0.522822167	7.28E-39
2041	MRPL47	0.263736467	5.79E-10
2042	PAX8	0.299422403	1.52E-12
2043	ERAL1	0.374724489	2.80E-19
2044	PRSS23	-0.243636054	1.14E-08
2045	MRPL57	0.440809927	7.66E-27
2046	YIF1A	0.46521528	4.37E-30
2047	PSMB9	0.413476719	1.65E-23
2048	RAB11FIP5	-0.19578042	5.07E-06
2049	EIF5	-0.370510234	7.49E-19
2050	RIT1	-0.128808199	0.002837482
2051	PLS3	-0.257401896	1.52E-09
2052	ARMCX3	-0.412695442	2.04E-23
2053	HMGN1	0.144314001	0.000814777
2054	MKRN1	-0.303441724	7.39E-13
2055	CPT1A	-0.18319408	2.01E-05
2056	TXNDC11	0.127728267	0.003080546
2057	RAB12	-0.358489446	1.14E-17
2058	SHARPIN	0.648439251	3.84E-65
2059	TNFRSF1B	0.265021649	4.74E-10
2060	DDX23	0.013772644	0.750612271
2061	ZC3H15	-0.162335212	0.00016254
2062	PTPN11	-0.492383199	5.11E-34
2063	UBAC2	0.093404778	0.030762621
2064	CTSC	-0.030573527	0.48038799
2065	NOB1	0.079267231	0.06694374
2066	CDK2AP1	0.042457932	0.326987468
2067	EPCAM	-0.200181752	3.06E-06
2068	GBA	-0.050777686	0.240996105
2069	ZCRB1	-0.131112652	0.002376183
2070	REPIN1	0.220920711	2.44E-07
2071	QSOX1	0.084057242	0.051998988
2072	HIPK3	-0.549026179	1.90E-43
2073	MRPL10	0.154165906	0.000344984
2074	PLEKHM2	0.406288528	1.11E-22
2075	FAM210B	-0.403794622	2.13E-22
2076	KCNE3	-0.001810201	0.966680312
2077	GNAQ	-0.469277116	1.19E-30
2078	CUL1	-0.040988255	0.344025535
2079	APOBEC3C	0.170803662	7.17E-05
2080	EFNB1	0.300959004	1.16E-12
2081	TSTD1	0.296216247	2.69E-12
2082	SNX9	-0.248371971	5.78E-09
2083	TFG	0.071265737	0.099636197
2084	SIGMAR1	0.433827208	5.82E-26
2085	CMTM6	-0.295257016	3.19E-12
2086	ATP5G1	0.410532651	3.62E-23

2087	PABPC4	0.156503571	0.000279183
2088	YAP1	-0.417869745	5.04E-24
2089	10-Sep	-0.396643354	1.33E-21
2090	EFEMP2	0.102438564	0.017782949
2091	COQ9	0.075059586	0.082825868
2092	MTX2	-0.075103666	0.082644944
2093	HSPA1B	0.291257275	6.40E-12
2094	PPP1R3B	-0.080447238	0.062968711
2095	YIPF5	-0.501454934	2.06E-35
2096	PDHA1	-0.042612753	0.325224984
2097	DLD	-0.495456297	1.74E-34
2098	SGK1	-0.125092134	0.003755351
2099	11-Sep	-0.51189457	4.55E-37
2100	TNKS1BP1	0.260729497	9.19E-10
2101	CNN2	0.306316639	4.38E-13
2102	VBP1	-0.342035687	3.97E-16
2103	MRPL39	-0.071891932	0.09668819
2104	BRI3	0.280988434	3.64E-11
2105	ACP5	0.18216399	2.24E-05
2106	TSN	-0.304938827	5.63E-13
2107	RNF149	0.1020147	0.018262707
2108	C12orf10	0.525853731	2.25E-39
2109	MAGEH1	-0.241970351	1.44E-08
2110	FAM134C	0.203126482	2.17E-06
2111	HGD	0.053555576	0.216185716
2112	DMTN	-0.053032759	0.220709353
2113	BAX	0.591621982	8.00E-52
2114	MOGS	0.629551081	2.09E-60
2115	PRPS1	-0.141400214	0.00104014
2116	PES1	0.316796824	6.16E-14
2117	DDIT3	0.280977705	3.65E-11
2118	PDCD5	0.590837269	1.17E-51
2119	GBP4	-0.105266427	0.014854971
2120	WDR6	0.10522474	0.014894853
2121	SNX5	-0.189732159	9.94E-06
2122	SPNS2	0.125576783	0.003622039
2123	6-Mar	-0.416031051	8.30E-24
2124	FAT1	-0.279131116	4.95E-11
2125	CFAP36	0.022906196	0.597046902
2126	CDH6	-0.134897543	0.00176482
2127	TMEM184B	0.109709993	0.011106393
2128	SAE1	0.03736965	0.388334262
2129	IMP3	0.275159318	9.48E-11
2130	IRX3	0.158596344	0.000230415
2131	PPP6C	-0.440804644	7.67E-27
2132	PEX19	-0.51976618	2.35E-38
2133	TNFRSF10B	0.303714511	7.03E-13
2134	PLEKHA2	-0.230047639	7.41E-08
2135	TIMMDC1	0.132011097	0.002215704
2136	ZDHHC9	-0.232663624	5.22E-08
2137	CDC42BPB	-0.221784239	2.19E-07
2138	ALDH2	0.048339242	0.26436765
2139	WDR82	-0.219816988	2.81E-07
2140	PCMT1	-0.359268884	9.61E-18
2141	MRPL41	0.523358255	5.92E-39
2142	NCEH1	-0.084491481	0.050793967
2143	CD52	0.379456992	9.13E-20
2144	MOB3A	0.326662496	9.07E-15

2145		7-Sep	-0.264880054	4.85E-10
2146	PER1		0.158959718	0.000222806
2147	C14orf105		-0.071367328	0.099153124
2148	MRPL33		-0.0261359	0.546366197
2149	SMCO4		0.22233377	2.04E-07
2150	NRD1		-0.127506549	0.003132733
2151	SPRY4		-0.074243942	0.086231779
2152	ALYREF		0.494596903	2.35E-34
2153	UGP2		-0.416436701	7.44E-24
2154	MBNL1		-0.211289836	8.16E-07
2155	IL10RB		0.352878761	3.92E-17
2156	PHF1		0.312629664	1.36E-13
2157	CAB39		-0.456646277	6.44E-29
2158	USF1		0.677527434	3.93E-73
2159	SYNCRIP		-0.340556096	5.40E-16
2160	DNAJA2		-0.383528924	3.43E-20
2161	DNM2		0.206922063	1.38E-06
2162	CEBPB		0.376570952	1.81E-19
2163	PTPRM		-0.135984406	0.00161808
2164	CD4		0.082521646	0.056453127
2165	YTHDF2		-0.221994622	2.13E-07
2166	MAP2K1		-0.315890604	7.33E-14
2167	TINF2		-0.039473086	0.362171682
2168	NRBP1		0.371119156	6.50E-19
2169	IDS		-0.410362227	3.79E-23
2170	C6orf89		-0.438750713	1.40E-26
2171	OAZ2		-0.192651905	7.20E-06
2172	NOLC1		-0.1478046	0.000604497
2173	TMEM129		0.160921642	0.00018563
2174	VPS26A		-0.482350886	1.59E-32
2175	TNFRSF14		0.48180839	1.91E-32
2176	CDC34		0.639172305	8.90E-63
2177	NDUFB8		0.372480952	4.74E-19
2178	RASD1		-0.078409554	0.069960393
2179	ATP2B4		-0.274984545	9.76E-11
2180	NKG7		0.400045405	5.59E-22
2181	EAPP		-0.299922706	1.39E-12
2182	IFI16		0.175629778	4.41E-05
2183	TST		0.299073237	1.62E-12
2184	TSG101		-0.270019675	2.16E-10
2185	CD99L2		-0.16413706	0.000137004
2186	MANF		0.243485873	1.17E-08
2187	SYF2		-0.104101583	0.016005394
2188	BTBD6		0.329316575	5.35E-15
2189	MT1X		0.231638502	5.99E-08
2190	COX7A2L		-0.206335174	1.48E-06
2191	GYPC		0.323507904	1.69E-14
2192	MPZL1		0.013636235	0.75300165
2193		2-Mar	-0.187731695	1.24E-05
2194	CARKD		0.164561255	0.000131567
2195	EIF1B		-0.2955544	3.02E-12
2196	EXT2		-0.193246364	6.74E-06
2197	ATXN7L3B		-0.396909216	1.24E-21
2198	CDC16		0.187832516	1.22E-05
2199	SPAG7		0.383169274	3.74E-20
2200	ZMYND11		-0.547257693	3.99E-43
2201	COPB2		-0.229750242	7.71E-08
2202	CMAS		-0.063146264	0.144670538

2203	ECSIT	0.227825391	9.96E-08
2204	PPP1R10	0.221681432	2.22E-07
2205	SNX4	-0.271476324	1.72E-10
2206	HSD11B2	-0.185478201	1.58E-05
2207	PRPF31	0.552441917	4.48E-44
2208	SEL1L	-0.559525594	2.12E-45
2209	ACTR3	-0.197546873	4.14E-06
2210	PARP4	-0.379618513	8.78E-20
2211	GNPDA1	0.088207524	0.041405081
2212	NFIC	0.013743737	0.751118408
2213	SCAND1	0.549505804	1.55E-43
2214	MRPS2	0.295512968	3.04E-12
2215	F11R	-0.074263664	0.086148114
2216	ATP11A	-0.273312911	1.28E-10
2217	PSENEN	0.56439482	2.49E-46
2218	FNBP1L	-0.519145234	2.98E-38
2219	BFAR	-0.205634456	1.61E-06
2220	SNRPA	0.724337385	3.96E-88
2221	PCED1A	0.520132394	2.04E-38
2222	ACADM	-0.461958901	1.23E-29
2223	SFXN3	0.259481966	1.11E-09
2224	PNP	0.044616581	0.302970693
2225	HADH	-0.399256554	6.84E-22
2226	SEL1L3	-0.058377961	0.17756901
2227	DNAJC5	-0.029572281	0.494886025
2228	SLBP	-0.072286894	0.094864849
2229	SEC14L1	-0.260763001	9.15E-10
2230	RELA	0.351434262	5.36E-17
2231	PRMT1	0.585068281	1.88E-50
2232	EIF3J	-0.274764214	1.01E-10
2233	ELK3	-0.195917753	4.99E-06
2234	CDC123	0.190862366	8.78E-06
2235	ARL2	0.500995403	2.43E-35
2236	GORASP2	0.034508227	0.425713614
2237	THAP4	0.327855826	7.16E-15
2238	LSM4	0.478800249	5.22E-32
2239	PPP1R15B	-0.140181674	0.00115043
2240	SKAP2	-0.427041565	3.99E-25
2241	TRIP10	0.564560281	2.32E-46
2242	SQRDL	0.294137575	3.88E-12
2243	ABLIM1	-0.346226869	1.64E-16
2244	LIMA1	-0.387038658	1.46E-20
2245	NRSN2	0.453132925	1.90E-28
2246	FAM65A	0.192233889	7.54E-06
2247	RNF128	-0.082618749	0.056162385
2248	CSF1R	0.05158191	0.23361611
2249	RIOK3	-0.252028746	3.39E-09
2250	LENG8	0.44624954	1.53E-27
2251	NMT1	0.130077093	0.002574275
2252	HPRT1	-0.055507787	0.199883243
2253	BUD31	0.342426576	3.65E-16
2254	CSNK2B	0.565390174	1.60E-46
2255	SMPD1	0.011597893	0.788975206
2256	PSMB2	0.261014925	8.80E-10
2257	ITGB2	0.179548585	2.95E-05
2258	UBQLN1	-0.438844268	1.36E-26
2259	PLEKHO2	0.159861393	0.00020493
2260	EIF1AX	-0.335808905	1.44E-15

2261	PSMA1	0.182973262	2.06E-05
2262	LRPAP1	-0.089165625	0.03923716
2263	UBAP2L	0.205627707	1.62E-06
2264	MRPL9	0.348547427	9.99E-17
2265	PREB	0.511646243	4.99E-37
2266	SEMA5B	0.08712184	0.04398309
2267	STAT5B	-0.204991669	1.74E-06
2268	FDPS	-0.013273465	0.759367469
2269	ATXN10	-0.318571794	4.39E-14
2270	PHLDA1	-0.180873101	2.57E-05
2271	CDH1	-0.355155749	2.38E-17
2272	ANP32A	-0.110833652	0.010302662
2273	SCPEP1	-0.072267955	0.094951651
2274	RDH11	-0.233750505	4.50E-08
2275	RIN2	-0.513781403	2.25E-37
2276	MCM3	0.080973747	0.061258945
2277	SNTA1	0.133167645	0.00202371
2278	SNX7	-0.430663556	1.44E-25
2279	FOPNL	-0.440741776	7.81E-27
2280	LRPPRC	-0.512834454	3.21E-37
2281	KIAA0100	-0.313517229	1.15E-13
2282	INPPL1	0.335977985	1.39E-15
2283	RCN1	0.034101313	0.43119423
2284	GUCY1B3	-0.071156667	0.100156912
2285	CALCOCO2	-0.182262804	2.22E-05
2286	AZIN1	-0.371082316	6.56E-19
2287	TFE3	0.287052488	1.32E-11
2288	CRKL	-0.16338022	0.000147233
2289	TAF11	0.153828795	0.000355587
2290	ERF	0.405918703	1.22E-22
2291	RCAN1	-0.143146613	0.00089901
2292	CDH2	-0.083098596	0.054743836
2293	PDGFD	-0.326551982	9.27E-15
2294	NME1-NME2	0.462114253	1.17E-29
2295	EWSR1	0.390912364	5.60E-21
2296	ETFA	-0.182251646	2.22E-05
2297	SRSF9	0.040635428	0.348198531
2298	SAR1A	-0.320692655	2.92E-14
2299	ITPA	0.63529736	8.21E-62
2300	RASSF4	0.320761676	2.88E-14
2301	CSRNP1	-0.074151928	0.086622986
2302	EIF5B	0.098641617	0.022500089
2303	LRRC8A	-0.030781539	0.477405206
2304	GALNT11	-0.199856754	3.18E-06
2305	CSNK2A1	-0.4251844	6.72E-25
2306	PSMD10	-0.326605411	9.17E-15
2307	TAX1BP1	-0.36641322	1.92E-18
2308	PPP2R5A	-0.423963795	9.44E-25
2309	WDR13	0.499097534	4.79E-35
2310	MAOA	-0.277190678	6.81E-11
2311	RER1	0.258172025	1.36E-09
2312	AK3	-0.331329654	3.57E-15
2313	VCL	-0.230730106	6.76E-08
2314	RALY	0.620276916	3.37E-58
2315	ALDOB	-0.105829433	0.014325457
2316	GBA2	0.218187982	3.46E-07
2317	NDUFS3	0.273742373	1.19E-10
2318	TOMM34	0.165119827	0.000124716

2319	ATP6V1C1	-0.414906513	1.12E-23
2320	UBE2E1	-0.269426068	2.38E-10
2321	MYLIP	-0.327264116	8.05E-15
2322	SKI	-0.058084204	0.17976404
2323	BRE	0.071832093	0.096966858
2324	STAT2	0.307852938	3.30E-13
2325	AKIRIN1	-0.234821497	3.89E-08
2326	ATL3	-0.437018711	2.32E-26
2327	AP3D1	0.112793324	0.00902377
2328	PSMD7	0.025168853	0.561316183
2329	HNRNPA0	-0.177432562	3.67E-05
2330	KIF1C	-0.003312665	0.939066543
2331	SELK	0.295849492	2.87E-12
2332	PHYH	-0.308981269	2.68E-13
2333	DDX56	0.712754251	3.88E-84
2334	PPA1	-0.038226205	0.377544761
2335	GOLGA5	-0.188162496	1.18E-05
2336	SRSF8	-0.297132903	2.29E-12
2337	MRPS36	-0.055740092	0.198004578
2338	UBL3	-0.522910682	7.04E-39
2339	DNAJB2	0.384658534	2.61E-20
2340	BIRC3	0.110783841	0.010337166
2341	SIL1	0.322601921	2.01E-14
2342	DENR	-0.257102183	1.59E-09
2343	FIBP	0.488904472	1.70E-33
2344	OLFML2A	-0.076414581	0.077408765
2345	DDX24	-0.211627	7.83E-07
2346	TMEM173	0.153350451	0.000371156
2347	USP11	0.008212246	0.849694123
2348	EPB41L1	-0.254368163	2.40E-09
2349	SUPT5H	0.388425578	1.04E-20
2350	CDK9	0.646850918	9.90E-65
2351	TSPAN18	-0.114224691	0.008181039
2352	PITHD1	0.108099244	0.012355341
2353	LRRC41	0.09162071	0.034116307
2354	TECR	0.430460727	1.52E-25
2355	PNN	0.213540047	6.19E-07
2356	HIATL1	-0.094631037	0.028624757
2357	DCTN6	-0.24127991	1.59E-08
2358	HLA-DQA2	0.016689533	0.700123363
2359	GIMAP6	-0.25084765	4.03E-09
2360	DAG1	-0.269024148	2.53E-10
2361	BTBD2	0.450570617	4.15E-28
2362	SOD3	0.167651083	9.77E-05
2363	EFHD1	-0.197123703	4.35E-06
2364	SRRT	0.678825182	1.65E-73
2365	TMEM258	0.475894971	1.37E-31
2366	AMD1	-0.360821554	6.79E-18
2367	RBM8A	-0.044869491	0.300235424
2368	NDST1	-0.294638444	3.55E-12
2369	MRPS15	0.50024932	3.18E-35
2370	TBL1XR1	-0.213160391	6.48E-07
2371	ANKRD10	0.32276199	1.95E-14
2372	PPP1R7	0.342062383	3.94E-16
2373	PUF60	0.474521371	2.15E-31
2374	SNX6	-0.402110579	3.29E-22
2375	CXCL10	0.101911932	0.018380715
2376	YTHDF1	0.169908421	7.83E-05

2377	DERL1	-0.107241601	0.013069692
2378	C5orf24	-0.367655285	1.45E-18
2379	WLS	-0.44047047	8.46E-27
2380	SPATS2L	0.083128653	0.054655983
2381	RHEB	0.050757981	0.241178965
2382	RGL1	-0.227656488	1.02E-07
2383	EFR3A	-0.404426878	1.80E-22
2384	DCTPP1	-0.073909889	0.087658868
2385	NAGA	-0.051890018	0.230831513
2386	ACTN1	0.168922981	8.63E-05
2387	METAP2	-0.388526606	1.01E-20
2388	LACTB2	-0.06658994	0.123966719
2389	NDUFB2	0.181009796	2.53E-05
2390	EIF3M	-0.058429307	0.177187389
2391	CNOT8	-0.323053724	1.84E-14
2392	ISOC2	0.371606815	5.80E-19
2393	LARS	-0.287888696	1.14E-11
2394	STX10	0.750292172	7.36E-98
2395	TXNDC15	-0.079773026	0.065215362
2396	ZNF146	-0.252623328	3.11E-09
2397	TOP2B	-0.461408231	1.46E-29
2398	PGLS	0.645494527	2.21E-64
2399	PTP4A3	0.271486584	1.71E-10
2400	POLR2I	0.544433381	1.29E-42
2401	KDELC2	-0.324288803	1.45E-14
2402	WBP11	-0.368770081	1.12E-18
2403	RBM22	-0.087480005	0.043118068
2404	MSRB2	0.309105728	2.62E-13
2405	AMZ2	0.049596359	0.252130967
2406	ISCA1	-0.402975984	2.63E-22
2407	COMT	0.464491232	5.50E-30
2408	DOK4	0.225629424	1.33E-07
2409	UQCRFS1	-0.259504522	1.11E-09
2410	RB1	-0.382174969	4.76E-20
2411	MTA2	0.447260912	1.13E-27
2412	ELF1	-0.431585395	1.11E-25
2413	RNF185	-0.284994073	1.86E-11
2414	MRPL16	0.269119709	2.49E-10
2415	TRA2A	0.363536109	3.69E-18
2416	SMAP2	0.083780523	0.05277926
2417	PQLC3	-0.222251917	2.06E-07
2418	TMEM263	-0.358355214	1.18E-17
2419	TRIM21	0.102110635	0.018153143
2420	ZFR	-0.465325162	4.22E-30
2421	VPS51	-0.016609557	0.701491397
2422	TP53I11	-0.011075334	0.798273716
2423	PPP1R18	0.44728372	1.12E-27
2424	MXD4	0.2224315	2.01E-07
2425	EMP1	-0.184146948	1.82E-05
2426	PLA1A	0.068577339	0.113113185
2427	MAP2K2	0.658949867	6.31E-68
2428	KLHDC10	-0.428894603	2.37E-25
2429	PBX2	0.183592966	1.93E-05
2430	DRAM2	-0.077994912	0.07145819
2431	LTA4H	-0.244780411	9.69E-09
2432	GPBP1	-0.311479763	1.68E-13
2433	NFU1	-0.066629831	0.123741168
2434	PIP4K2B	-0.021014397	0.627689769

2435	COASY	0.541011021	5.29E-42
2436	ACBD3	-0.39077456	5.80E-21
2437	RBM47	-0.495447474	1.75E-34
2438	DDX21	-0.277983379	5.98E-11
2439	BCL7B	0.256457724	1.76E-09
2440	RALBP1	-0.445938511	1.67E-27
2441	ARL4C	0.147944217	0.000597241
2442	F2RL1	-0.25256937	3.13E-09
2443	NDUFB1	0.344639314	2.29E-16
2444	UNG	-0.103376334	0.016760482
2445	ZDHHC7	-0.053917811	0.21309079
2446	UBTF	0.237948152	2.53E-08
2447	RNF14	-0.394769373	2.14E-21
2448	TADA3	0.345952444	1.74E-16
2449	ERP44	-0.111257146	0.010013407
2450	NAGLU	0.205560596	1.63E-06
2451	TXLNA	0.256844954	1.66E-09
2452	EMC4	0.154297373	0.000340929
2453	PUM1	-0.450014972	4.91E-28
2454	CD97	0.357805049	1.33E-17
2455	CIZ1	0.329233614	5.44E-15
2456	SPG20	-0.283855303	2.26E-11
2457	JOSD1	0.030471176	0.481859363
2458	AP3S1	0.031958542	0.460719452
2459	R3HDM4	0.666486708	5.41E-70
2460	EDIL3	-0.104562454	0.015541208
2461	RAP1A	-0.346985064	1.39E-16
2462	MRPS7	0.189599401	1.01E-05
2463	NPTX2	0.051661795	0.232891865
2464	HEYL	-0.046639531	0.281552498
2465	HLA-DOA	-0.017305141	0.68962592
2466	OAT	-0.364657141	2.86E-18
2467	IPO5	-0.393957293	2.62E-21
2468	SMARCA2	-0.382047202	4.90E-20
2469	ICT1	0.447313533	1.11E-27
2470	LANCL1	-0.524792646	3.40E-39
2471	PKP4	-0.202898671	2.23E-06
2472	KLF2	0.013833554	0.749546107
2473	EPDR1	-0.235007524	3.79E-08
2474	CISD1	-0.209769314	9.82E-07
2475	PDGFB	-0.03645254	0.400091813
2476	ATP6V1D	-0.307342868	3.62E-13
2477	2-Mar	0.414347978	1.31E-23
2478	TNFAIP2	0.509538028	1.09E-36
2479	RCBTB2	-0.300289069	1.30E-12
2480	SSR1	-0.408753197	5.81E-23
2481	FAM96A	-0.203470731	2.08E-06
2482	TMEM60	0.209351695	1.03E-06
2483	ECI2	-0.171003796	7.03E-05
2484	NPDC1	0.427652079	3.37E-25
2485	YPEL3	0.504915177	5.91E-36
2486	PSME3	-0.120516093	0.005251217
2487	PSMB10	0.439766391	1.04E-26
2488	MGST1	0.128418362	0.002923134
2489	MRPS10	-0.234544665	4.04E-08
2490	NDRG3	-0.119757877	0.0055454
2491	SLCO4C1	-0.246355974	7.74E-09
2492	ASNSD1	-0.269313961	2.42E-10

2493	HMCES	0.250424856	4.29E-09
2494	SNRPG	0.509062142	1.30E-36
2495	NUB1	-0.124821963	0.003831583
2496	JAGN1	0.183884281	1.87E-05
2497	RBX1	0.490195185	1.09E-33
2498	UCK1	0.293536276	4.30E-12
2499	PTOV1	0.615434029	4.48E-57
2500	MSMO1	-0.31165289	1.63E-13
2501	CREB3L2	-0.375916275	2.12E-19
2502	UHMK1	-0.523788387	5.01E-39
2503	CALCRL	-0.368096857	1.31E-18
2504	SPATA20	0.471075338	6.63E-31
2505	DDX50	-0.253733686	2.64E-09
2506	FAM120AOS	-0.162888967	0.000154252
2507	QDPR	-0.009012282	0.835251339
2508	VTI1B	-0.392872793	3.44E-21
2509	BTBD1	-0.492749161	4.49E-34
2510	HTATIP2	0.077675288	0.072630554
2511	MUC1	-0.025729871	0.552619156
2512	HEXIM1	0.086796201	0.044782195
2513	MFAP1	-0.401372929	3.98E-22
2514	PHF5A	0.259538541	1.10E-09
2515	PSMD1	-0.14173438	0.001011647
2516	NPR3	-0.288153592	1.09E-11
2517	ADNP	-0.365820724	2.20E-18
2518	RPS4Y1	0.008540377	0.843764113
2519	DYSF	-0.148109355	0.000588763
2520	PTPN18	0.416060155	8.24E-24
2521	RNASE6	0.015127119	0.727019595
2522	ERO1L	-0.074824908	0.083794491
2523	HSD17B8	-0.067196404	0.120572055
2524	RIC8A	0.411933374	2.50E-23
2525	PAFAH1B2	-0.399376433	6.64E-22
2526	ATP13A3	-0.199727462	3.23E-06
2527	DPCD	0.150423177	0.000481138
2528	TMEM126A	0.08529565	0.04862396
2529	ARHGAP18	-0.500631447	2.77E-35
2530	TOLLIP	-0.028907228	0.504643331
2531	ATXN2L	0.442836417	4.21E-27
2532	DNPEP	0.372782817	4.41E-19
2533	LRP1	-0.035402838	0.41380791
2534	MAL	-0.148537286	0.00056731
2535	SF3A3	-0.078690342	0.068960777
2536	PDCD4	-0.411390854	2.88E-23
2537	MED4	0.018227349	0.674011671
2538	HUWE1	-0.358597688	1.12E-17
2539	SLC2A3	0.073449595	0.089656317
2540	IGF2R	-0.467678434	1.99E-30
2541	ALG5	-0.159958068	0.000203095
2542	ARRDC2	0.282400966	2.88E-11
2543	C11orf58	-0.460103708	2.20E-29
2544	RERE	-0.158295247	0.000236903
2545	CLK1	0.370866672	6.89E-19
2546	NABP2	0.429567618	1.96E-25
2547	GLRX	0.027765985	0.521619809
2548	IMPAD1	-0.334889525	1.74E-15
2549	COL15A1	-0.203496919	2.08E-06
2550	HLA-DMB	0.040899949	0.34506695

2551	HM13	0.407481674	8.12E-23
2552	ABHD10	-0.188465628	1.14E-05
2553	PPIC	0.02917376	0.500720812
2554	DHPS	0.479640076	3.95E-32
2555	UNC119B	-0.456652251	6.43E-29
2556	NDUFA3	0.478463011	5.85E-32
2557	GRB10	-0.205005367	1.74E-06
2558	CDIP1	-0.004382329	0.919449678
2559	AIG1	-0.174496604	4.95E-05
2560	CCDC50	-0.318667762	4.31E-14
2561	MEAF6	-0.00612455	0.887608269
2562	ZER1	0.06526606	0.131635108
2563	EIF3F	0.101514367	0.018843526
2564	BIRC2	-0.236202991	3.22E-08
2565	TARDBP	0.163861078	0.000140654
2566	APEH	-0.052496895	0.225415693
2567	CYBRD1	-0.214980967	5.18E-07
2568	TUBA1C	0.171467043	6.71E-05
2569	SLC10A3	0.454228391	1.36E-28
2570	CYYR1	-0.295703821	2.94E-12
2571	MAP2K3	0.361607136	5.70E-18
2572	PABPN1	0.627310751	7.25E-60
2573	AAR2	0.019081302	0.659676696
2574	SPTLC1	-0.502456049	1.44E-35
2575	AKIRIN2	-0.018749998	0.665223745
2576	CLUH	0.169233514	8.37E-05
2577	VPS35	-0.54009829	7.69E-42
2578	SMEK2	-0.354168647	2.96E-17
2579	KL	-0.314945826	8.76E-14
2580	HSD17B4	-0.441962886	5.45E-27
2581	MRPL43	0.310275017	2.11E-13
2582	GLYAT	-0.071031806	0.100755672
2583	ADIPOR2	-0.220624111	2.54E-07
2584	PCIF1	0.263463667	6.04E-10
2585	CNIH1	-0.431834849	1.03E-25
2586	ATIC	0.290238732	7.62E-12
2587	LMAN2L	0.006862215	0.874177766
2588	GSDMD	0.640379131	4.43E-63
2589	ZFP91	-0.517155223	6.34E-38
2590	STUB1	0.20045713	2.96E-06
2591	NFKB2	0.661560028	1.23E-68
2592	MCM7	0.381317153	5.85E-20
2593	RNF19B	-0.16196127	0.000168371
2594	POLR3GL	0.055353398	0.201138949
2595	ARHGEF17	-0.084398688	0.051049491
2596	MAN2B1	0.177263447	3.74E-05
2597	DDT	0.259496344	1.11E-09
2598	CGGBP1	-0.348301284	1.05E-16
2599	TBCB	0.657216497	1.85E-67
2600	SH2B3	-0.138192555	0.001353849
2601	PI4KB	0.277858677	6.11E-11
2602	DCTN1	-0.126388551	0.003408331
2603	SDC3	0.150792417	0.000465761
2604	RBBP4	-0.282564704	2.80E-11
2605	NR3C1	-0.411438141	2.85E-23
2606	PIK3IP1	-0.115570614	0.007453439
2607	PBLD	-0.133945278	0.00190331
2608	LXN	-0.089189807	0.03918371

2609	CASC3	-0.105681802	0.014462675
2610	ARFGAP2	0.052375335	0.226493192
2611	SF3A2	0.644072148	5.12E-64
2612	FKBP3	0.13247006	0.002137604
2613	UBE2K	-0.446979966	1.23E-27
2614	GTPBP6	0.43802881	1.73E-26
2615	SNRK	-0.37002058	8.38E-19
2616	RAB31	-0.194784593	5.67E-06
2617	CD320	0.322599619	2.01E-14
2618	GBP1	0.148506745	0.000568817
2619	NUDT21	-0.541832345	3.78E-42
2620	USP10	-0.375952452	2.10E-19
2621	TSPAN4	0.465367352	4.16E-30
2622	ILVBL	0.08731054	0.043525547
2623	CPSF7	0.219305914	3.00E-07
2624	MAPK1IP1L	-0.286349873	1.48E-11
2625	CTCF	-0.316144976	6.98E-14
2626	PBDC1	0.238205964	2.44E-08
2627	DCXR	0.34294295	3.28E-16
2628	RPL36A	0.458760291	3.34E-29
2629	SELM	0.267649207	3.15E-10
2630	GLMP	0.168497523	8.99E-05
2631	CORO1B	0.605821888	6.68E-55
2632	SLC16A12	-0.287318481	1.26E-11
2633	FN3KRP	0.050227644	0.246136937
2634	RFXANK	0.656042361	3.81E-67
2635	NDFIP2	-0.429751232	1.86E-25
2636	UTP3	-0.219759989	2.84E-07
2637	NCLN	0.516049286	9.62E-38
2638	ACMSD	-0.028536018	0.510133177
2639	NT5C3B	0.285328602	1.76E-11
2640	ARPC5	0.188506353	1.14E-05
2641	TRIP12	-0.414350074	1.31E-23
2642	YIPF2	0.260439942	9.61E-10
2643	C10orf54	0.156244473	0.000285851
2644	RASSF7	0.551393687	6.99E-44
2645	ABI1	-0.276236315	7.96E-11
2646	RALA	-0.297975282	1.97E-12
2647	RPP25L	0.268418005	2.79E-10
2648	FBXO7	-0.360872371	6.72E-18
2649	LAMA5	0.290781281	6.94E-12
2650	SCAMP1	-0.630315863	1.36E-60
2651	GPSM3	0.454529461	1.24E-28
2652	CLCN3	-0.505961261	4.04E-36
2653	POMGNT1	0.232048447	5.67E-08
2654	PMPCB	-0.252474444	3.17E-09
2655	ACP2	0.324191274	1.48E-14
2656	TRIM22	-0.116853671	0.006814281
2657	MCCC2	-0.287968465	1.13E-11
2658	DKK3	-0.10532712	0.014797073
2659	DDX49	0.638691608	1.17E-62
2660	BAZ1B	-0.187791629	1.23E-05
2661	ZCCHC17	-0.002382903	0.956148133
2662	VEZF1	-0.45658263	6.57E-29
2663	SLC15A4	0.042025874	0.331938663
2664	NSMCE1	0.331202656	3.66E-15
2665	ZMIZ2	0.568370204	4.23E-47
2666	CSNK1E	0.453284054	1.81E-28

2667	WBSCR16	0.285199149	1.80E-11
2668	LGR4	-0.494530964	2.41E-34
2669	GID8	-0.257256772	1.56E-09
2670	TNFAIP6	-0.010831951	0.802614362
2671	TUBB6	0.403488563	2.30E-22
2672	NUMA1	0.004047455	0.925586334
2673	RGS10	0.37157254	5.85E-19
2674	RPA1	-0.220731529	2.50E-07
2675	GPBP1L1	-0.347527503	1.24E-16
2676	RNF26	-0.173703855	5.36E-05
2677	ID4	-0.116391281	0.007038729
2678	GPRC5C	0.100122458	0.020544881
2679	PAIP1	-0.356516051	1.77E-17
2680	ABCF1	0.06032294	0.163535384
2681	GSK3A	0.543004374	2.33E-42
2682	DAXX	0.442625406	4.48E-27
2683	CCDC115	-0.109458444	0.011293755
2684	PDIA5	0.212174509	7.32E-07
2685	CNOT1	-0.502443313	1.45E-35
2686	RPS19BP1	0.52349227	5.62E-39
2687	MEF2A	-0.460977719	1.67E-29
2688	STAG2	-0.494896097	2.12E-34
2689	PRKAA1	-0.483106518	1.23E-32
2690	TRIM26	0.083282468	0.054208222
2691	MYL6B	0.398483481	8.34E-22
2692	MFSD1	-0.377049733	1.62E-19
2693	SSB	0.009350396	0.829163797
2694	TACC1	-0.423623755	1.04E-24
2695	NR4A1	-0.023654867	0.585110404
2696	YTHDF3	-0.475076261	1.79E-31
2697	B3GAT3	0.607788077	2.44E-55
2698	CECR1	-0.047437568	0.273392047
2699	RNF139	-0.375410185	2.38E-19
2700	ATP2C1	-0.456356653	7.05E-29
2701	PDLIM5	-0.433711644	6.02E-26
2702	VCAN	-0.152879616	0.000387099
2703	C1GALT1C1	-0.312354584	1.43E-13
2704	WTAP	-0.022422118	0.604823144
2705	CYFIP1	-0.345298236	2.00E-16
2706	ACVR1B	-0.227964904	9.77E-08
2707	COPS3	0.037124468	0.391456803
2708	TFPI	-0.08093136	0.061395158
2709	NMB	0.246619278	7.45E-09
2710	SRPRB	0.193333142	6.67E-06
2711	TCF25	0.354731853	2.62E-17
2712	TIMM9	-0.004795451	0.911885717
2713	PSMG2	0.165539073	0.000119796
2714	RAB18	-0.558132836	3.88E-45
2715	PHLDA3	0.372114892	5.16E-19
2716	FASTK	0.6330211	2.98E-61
2717	DDX6	-0.548437563	2.43E-43
2718	MBD2	-0.046114587	0.287009376
2719	STX3	-0.064447407	0.136557379
2720	OGT	0.231567815	6.05E-08
2721	BTN3A2	0.147225503	0.000635476
2722	TMBIM4	-0.094355612	0.029093489
2723	HEBP1	-0.141009555	0.001074388
2724	AFF4	-0.540219328	7.32E-42

2725	MEF2D	-0.056346397	0.193162097
2726	H6PD	-0.155602846	0.000303009
2727	TMEM126B	6.98E-05	0.99871496
2728	FAM84B	-0.401330709	4.02E-22
2729	HMG20B	0.640007363	5.49E-63
2730	SLC1A1	-0.211348522	8.10E-07
2731	ALAS1	-0.206086415	1.53E-06
2732	GSPT1	-0.489345437	1.46E-33
2733	TMEM8A	0.287736191	1.17E-11
2734	TSC22D4	0.445964553	1.66E-27
2735	EPHX2	-0.174652395	4.88E-05
2736	ALDH7A1	-0.0883446	0.041088847
2737	TWF1	-0.345650763	1.85E-16
2738	GALNT1	-0.261384703	8.32E-10
2739	DAP3	0.183402881	1.97E-05
2740	SPCS2	-0.13522476	0.001719407
2741	SLC39A13	0.369854789	8.71E-19
2742	PLBD1	-0.146269619	0.00068985
2743	PARP10	0.556372941	8.31E-45
2744	PRPS2	-0.36862083	1.16E-18
2745	AIMP1	-0.25959747	1.09E-09
2746	RNF19A	-0.041015291	0.343707087
2747	MCRS1	0.501103039	2.34E-35
2748	ABCC3	0.125335526	0.003687856
2749	STC2	0.069532571	0.108169348
2750	HEG1	-0.323143826	1.81E-14
2751	PARP1	-0.122727442	0.004471799
2752	NAA38	0.543652969	1.79E-42
2753	NAA50	-0.372644093	4.56E-19
2754	G3BP1	-0.372841515	4.35E-19
2755	INF2	0.327448938	7.76E-15
2756	BSDC1	-0.107713491	0.012672261
2757	TRADD	0.374718176	2.81E-19
2758	DCTN4	-0.505280787	5.18E-36
2759	PPP2R5D	-0.001003362	0.981527781
2760	TAB2	-0.464834251	4.93E-30
2761	PLAU	0.196886277	4.47E-06
2762	MIEN1	0.51519557	1.33E-37
2763	SPAG4	0.312089789	1.50E-13
2764	HSPBP1	0.588517594	3.60E-51
2765	MAX	0.277590738	6.38E-11
2766	DLL4	0.021921319	0.612915321
2767	ITM2A	-0.112653124	0.009110333
2768	SLC35D2	0.107964851	0.01246495
2769	COX7A1	0.07466841	0.084445498
2770	FUBP1	-0.107086505	0.013202694
2771	RRM2B	-0.521950602	1.02E-38
2772	RHOBTB1	-0.111748219	0.009687044
2773	RAB9A	-0.232875466	5.07E-08
2774	HRAS	0.686396822	9.36E-76
2775	SFXN4	0.076543745	0.076907768
2776	TMUB1	0.660006903	3.26E-68
2777	BRMS1	0.597248996	5.03E-53
2778	KXD1	0.687572849	4.13E-76
2779	ORMDL3	0.214361957	5.59E-07
2780	E2F4	0.58376689	3.48E-50
2781	ABL1	0.05982911	0.167016956
2782	SEPHS1	-0.023324841	0.590358563

2783	INHBB	0.057661429	0.182958335
2784	UBR7	-0.289317902	8.93E-12
2785	ALG2	-0.182457881	2.18E-05
2786	CXCL9	0.154865267	0.00032392
2787	PRKACA	0.316921044	6.02E-14
2788	IVD	-0.168745851	8.78E-05
2789	ARSD	-0.208381614	1.16E-06
2790	PARM1	-0.266955794	3.51E-10
2791	GOLGB1	-0.259879298	1.05E-09
2792	ABRACL	0.211259036	8.19E-07
2793	AHR	-0.185174334	1.63E-05
2794	TUBA4A	0.29126778	6.38E-12
2795	CDK4	-0.049723405	0.250916584
2796	ARHGEF12	-0.58362318	3.72E-50
2797	GLIS2	0.196446718	4.70E-06
2798	ACSL4	-0.410132805	4.03E-23
2799	FAF2	-0.183825558	1.88E-05
2800	CEBPZ	-0.351100846	5.77E-17
2801	PRKAB1	-0.148607166	0.000563877
2802	TMEM87A	-0.495484713	1.72E-34
2803	IER3IP1	-0.199005194	3.51E-06
2804	RHOQ	-0.086371852	0.045841821
2805	NECAP2	0.153088149	0.00037996
2806	GPR4	-0.023957072	0.580323695
2807	SLC16A1	-0.124107916	0.004039854
2808	PAPSS1	-0.29325194	4.52E-12
2809	SGPP2	-0.351550947	5.23E-17
2810	TCEA1	-0.334245059	1.98E-15
2811	TRIAP1	0.123822075	0.004126061
2812	MRPL55	0.626602441	1.07E-59
2813	POFUT1	-0.319537854	3.64E-14
2814	ATF3	-0.015544403	0.719801716
2815	UBALD2	0.532427308	1.69E-40
2816	IGFBP6	0.384103282	2.98E-20
2817	QPRT	0.069072948	0.11052643
2818	NT5C	0.665760446	8.61E-70
2819	KDM3B	-0.405550851	1.35E-22
2820	UBQLN2	-0.465333452	4.21E-30
2821	TAF13	-0.054874924	0.205067023
2822	KBTBD11	-0.152887708	0.000386819
2823	KCTD20	-0.335263204	1.61E-15
2824	MAGOH	0.503969554	8.33E-36
2825	C11orf96	0.228633757	8.95E-08
2826	ISYNA1	0.394188565	2.47E-21
2827	SORT1	-0.438236821	1.63E-26
2828	PIH1D1	0.572326837	7.06E-48
2829	TWSG1	-0.35443398	2.79E-17
2830	DTX3L	-0.187120804	1.32E-05
2831	ALDH4A1	0.082475086	0.05659298
2832	TFDP1	-0.110718156	0.010382824
2833	IRF2	-0.010428383	0.80982518
2834	HAVCR2	0.062829132	0.146702724
2835	SLC44A3	-0.289343186	8.89E-12
2836	GRAMD4	0.14804512	0.000592047
2837	PARP14	0.023674809	0.584793979
2838	NCOR2	0.219578678	2.90E-07
2839	MAP7D1	0.513448525	2.55E-37
2840	MXRA7	-0.00450217	0.917254705

2841	TRAPPC3	0.350513625	6.55E-17
2842	SUCLA2	-0.482107957	1.73E-32
2843	MPZL2	-0.283178277	2.53E-11
2844	HAAO	0.244408229	1.02E-08
2845	SLC50A1	0.483677958	1.02E-32
2846	SCAF1	0.498734019	5.46E-35
2847	MUT	-0.507900663	1.99E-36
2848	RBM10	0.577090626	7.93E-49
2849	GALNT18	-0.047602736	0.271723448
2850	SLC39A9	-0.483948237	9.26E-33
2851	COQ5	-0.014915861	0.730683057
2852	EHD4	-0.197104591	4.36E-06
2853	TMUB2	0.503532721	9.76E-36
2854	TAPBPL	0.088319933	0.041145601
2855	FAM192A	0.255260405	2.10E-09
2856	TMX1	-0.453444894	1.73E-28
2857	CLDN3	0.07536581	0.08157557
2858	CYB5R1	-0.14777872	0.000605851
2859	DDX42	-0.089385228	0.038754029
2860	TGFBR1	-0.358699806	1.09E-17
2861	MBTPS1	-0.391096978	5.35E-21
2862	NEK7	-0.412657019	2.06E-23
2863	RPL26L1	0.333307379	2.40E-15
2864	LOXL2	0.145312719	0.000748575
2865	EMC10	0.160398918	0.000194922
2866	TAF9B	-0.434914028	4.26E-26
2867	MFGE8	0.105685904	0.014458846
2868	EHD1	0.306870377	3.95E-13
2869	PIP4K2C	-0.301080976	1.13E-12
2870	DVL3	0.396498244	1.38E-21
2871	PRCP	-0.416669802	6.98E-24
2872	MGST3	0.151062797	0.000454793
2873	TREM2	0.228938371	8.59E-08
2874	PYCR2	0.53175062	2.22E-40
2875	STK38	0.21482478	5.28E-07
2876	ATF5	0.370907183	6.83E-19
2877	COX15	-0.437118152	2.25E-26
2878	GPR107	-0.382461316	4.44E-20
2879	CHCHD3	-0.246879688	7.17E-09
2880	CHPF2	0.345489492	1.92E-16
2881	CYB5D2	0.056838043	0.189299532
2882	SOX4	-0.110099811	0.010821484
2883	YIPF1	0.09517011	0.027726085
2884	DAB2IP	-0.138165257	0.001356858
2885	G6PC3	0.467023833	2.45E-30
2886	GOLPH3L	-0.457867807	4.41E-29
2887	GBE1	-0.353863506	3.16E-17
2888	SCMH1	0.167073863	0.000103297
2889	VPS11	-0.020911284	0.629379157
2890	TMED5	-0.502928355	1.21E-35
2891	GLB1	-0.24563542	8.58E-09
2892	YY1	-0.186139001	1.47E-05
2893	ACSM2A	0.045547985	0.292978693
2894	DMGDH	-0.280708769	3.82E-11
2895	CXCL12	-0.110226901	0.010730006
2896	UNC50	-0.021334671	0.622454838
2897	PTBP3	-0.452945503	2.01E-28
2898	LRP5	-0.147710716	0.000609422

2899	OTUB1	0.497248285	9.24E-35
2900	YES1	-0.461755981	1.31E-29
2901	PDCD6IP	-0.516203154	9.08E-38
2902	DDC	-0.069725098	0.107193878
2903	CCS	0.481792161	1.92E-32
2904	SRSF11	0.264929026	4.81E-10
2905	CHSY1	-0.179111765	3.09E-05
2906	CD70	0.301972031	9.64E-13
2907	IARS	-0.29394078	4.01E-12
2908	DYNLT3	-0.446225698	1.54E-27
2909	MAPKAPK3	-0.01532409	0.723609472
2910	ERBB2	-0.154889682	0.000323206
2911	CIR1	0.142078898	0.000983027
2912	EPHA2	0.142384999	0.000958228
2913	TMEM245	-0.59086098	1.16E-51
2914	GGA2	-0.146320005	0.00068688
2915	MRPS18A	0.032902267	0.447579079
2916	CHCHD1	0.379940088	8.13E-20
2917	IFI27L2	0.465633287	3.82E-30
2918	TP53I3	0.432726065	7.98E-26
2919	SRF	0.177810308	3.53E-05
2920	CYBB	-0.194286273	6.00E-06
2921	ERAP1	-0.367006528	1.68E-18
2922	ARRDC4	-0.151556651	0.000435376
2923	KIAA0368	-0.308291173	3.04E-13
2924	PATL1	-0.096993602	0.024863746
2925	VPS4B	-0.542748969	2.59E-42
2926	GUCY1A3	-0.346892619	1.42E-16
2927	RGS2	0.23290328	5.05E-08
2928	MMP2	0.056728994	0.190151327
2929	OBFC1	-0.065219784	0.131909641
2930	CD2AP	-0.468704931	1.43E-30
2931	NBN	-0.313912311	1.07E-13
2932	ANKRD13A	0.031834032	0.462469037
2933	TBC1D13	-0.02208654	0.610240329
2934	FAM8A1	-0.478032175	6.75E-32
2935	SUB1	-0.077148088	0.074598568
2936	ST6GAL1	-0.359250499	9.65E-18
2937	EIF4EBP3	0.195481933	5.24E-06
2938	HNRNPUL2	0.129560437	0.002678635
2939	SYVN1	0.302208977	9.24E-13
2940	FABP7	0.118134802	0.006225531
2941	OLFML2B	0.124762542	0.003848536
2942	PPP3R1	-0.18951502	1.02E-05
2943	SEZ6L2	0.125904167	0.00353443
2944	ITPRIP	0.031940224	0.460976617
2945	HSF1	0.546598451	5.26E-43
2946	NDUFB5	-0.282482421	2.84E-11
2947	CKS2	0.357143425	1.54E-17
2948	RAB5A	-0.437942257	1.77E-26
2949	GRHPR	0.097313858	0.024388317
2950	POR	0.292990052	4.73E-12
2951	SLC6A3	0.114113777	0.008243736
2952	COPS8	-0.186279751	1.45E-05
2953	HPS1	0.375756883	2.20E-19
2954	POLR2B	-0.38605794	1.85E-20
2955	EIF2B1	0.384929026	2.44E-20
2956	CUL4B	-0.428713165	2.50E-25

2957	ADRBK1	0.554171194	2.14E-44
2958	SEMA4B	0.244374278	1.03E-08
2959	WSB1	0.342401332	3.67E-16
2960	WDTC1	-0.077376691	0.073739933
2961	WNK1	-0.345262236	2.01E-16
2962	MRPL12	0.566380437	1.03E-46
2963	STX4	0.670128171	5.16E-71
2964	LPHN2	-0.340223757	5.79E-16
2965	PIM1	0.248233696	5.90E-09
2966	UNC5B	-0.141510077	0.001030692
2967	NAB1	-0.229615708	7.85E-08
2968	SHOC2	-0.461627117	1.36E-29
2969	PDXDC1	-0.275771206	8.59E-11
2970	SLC4A2	0.102085659	0.018181612
2971	NSFL1C	0.128170551	0.002978798
2972	NCOA5	0.167212953	0.000101912
2973	TXNRD1	-0.214359799	5.59E-07
2974	CTBP1	0.582643369	5.92E-50
2975	NEDD9	-0.270913213	1.88E-10
2976	RARA	0.39421525	2.46E-21
2977	RTKN	0.45851126	3.61E-29
2978	GBF1	-0.00598576	0.890138907
2979	EMX2	-0.314626215	9.31E-14
2980	IDH1	-0.10277591	0.017409024
2981	ARSA	0.536425299	3.41E-41
2982	CDK16	0.295166092	3.24E-12
2983	HSPA13	-0.450870227	3.79E-28
2984	CLDN1	0.039495202	0.36190258
2985	MRPS9	0.065789191	0.12856225
2986	HGSNAT	-0.027377116	0.527470616
2987	TRAP1	-0.00159717	0.970599611
2988	CD44	0.01696356	0.695443414
2989	CSPG4	-0.009896601	0.81935144
2990	SDF2L1	0.559430543	2.21E-45
2991	MGST2	0.00404066	0.925710894
2992	HCLS1	0.208651549	1.12E-06
2993	SUPT16H	-0.229113493	8.39E-08
2994	NDRG2	-0.21704859	4.00E-07
2995	C10orf32	-0.380085278	7.86E-20
2996	R3HCC1	0.149999427	0.000499367
2997	ELOVL7	-0.35369023	3.29E-17
2998	GEM	0.066317791	0.125514032
2999	CSNK1G2	0.567177033	7.22E-47
3000	TMEM167B	-0.519214575	2.90E-38
3001	PRUNE	-0.168550136	8.95E-05
3002	LIX1L	-0.018794045	0.664485196
3003	POLR2H	0.639779248	6.27E-63
3004	H2AFJ	0.43726967	2.15E-26
3005	ADCK2	0.02782691	0.520706181
3006	ZMIZ1	-0.244505084	1.01E-08
3007	ACVRL1	-0.057922745	0.180979033
3008	SS18	-0.179117853	3.09E-05
3009	LUM	0.023749582	0.583608212
3010	TMEM128	-0.280736348	3.80E-11
3011	SMC3	-0.323957439	1.54E-14
3012	ARAP1	-0.029801119	0.491552032
3013	VPS4A	0.40120777	4.15E-22
3014	PCMTD2	-0.295781638	2.90E-12

3015	ZNF189	-0.162426255	0.000161149
3016	UACA	-0.133442556	0.001980372
3017	SWAP70	-0.410158064	4.00E-23
3018	SMARCD2	0.297017635	2.34E-12
3019	STAP2	0.393683743	2.81E-21
3020	GIT1	0.430280327	1.60E-25
3021	SUPT6H	0.006747572	0.876262784
3022	APLNR	-0.203932028	1.97E-06
3023	WDFY1	-0.338505678	8.27E-16
3024	USP7	-0.323625089	1.65E-14
3025	PHKA2	0.212304452	7.21E-07
3026	REXO2	-0.127241335	0.003196213
3027	GIPC1	0.523900781	4.80E-39
3028	HAVCR1	-0.165651547	0.000118507
3029	BABAM1	0.589822629	1.92E-51
3030	CHID1	0.241486589	1.55E-08
3031	RFTN1	0.040799676	0.346251945
3032	FNDC3A	-0.458878027	3.22E-29
3033	SRP54	-0.315481711	7.92E-14
3034	STX16	0.368276524	1.25E-18
3035	RPL17	-0.037441325	0.387424297
3036	PRCC	0.521040327	1.44E-38
3037	NFKBIE	0.468082485	1.74E-30
3038	UFSP2	-0.429462245	2.02E-25
3039	ZC3H7B	-0.125092072	0.003755368
3040	MXRA8	0.125914263	0.003531759
3041	IMP4	0.527353468	1.25E-39
3042	NMD3	-0.414057101	1.41E-23
3043	SREBF2	-0.260436996	9.61E-10
3044	ATG13	0.005780198	0.893889089
3045	C8orf33	0.011119674	0.797483593
3046	THAP11	0.110642474	0.010435653
3047	SOX9	0.005952247	0.890750152
3048	BPGM	-0.279015343	5.05E-11
3049	SLC35E1	-0.281196171	3.52E-11
3050	TBC1D1	-0.42498091	7.11E-25
3051	UBE2N	-0.19241645	7.39E-06
3052	PAICS	-0.120901924	0.005106982
3053	NCBP2	0.046350227	0.284551114
3054	SMIM14	-0.471686114	5.44E-31
3055	TSPAN15	0.140354477	0.00113416
3056	MED10	0.132060383	0.002207194
3057	WAC	-0.354079369	3.02E-17
3058	SLC30A1	-0.411343429	2.92E-23
3059	KLHL12	-0.279113754	4.97E-11
3060	TBL1X	-0.386135749	1.82E-20
3061	RARS2	-0.304452615	6.15E-13
3062	TP53	0.136329139	0.001573931
3063	MDK	0.260052458	1.02E-09
3064	TMEM88	0.041755887	0.335056991
3065	AGPAT3	-0.100810092	0.019688367
3066	RRAGD	-0.383866078	3.16E-20
3067	SLC17A5	-0.414996176	1.10E-23
3068	NR1D1	0.11037781	0.010622276
3069	TNPO3	-0.133372581	0.001991322
3070	NAMPT	-0.14009396	0.001158771
3071	SAMD4B	0.119760051	0.005544536
3072	NEDD8	0.312062836	1.51E-13

3073	ZNHIT1	0.57936623	2.75E-49
3074	FAM195B	0.505813734	4.27E-36
3075	SPAG9	-0.313274892	1.20E-13
3076	GNA11	-0.211920222	7.55E-07
3077	TKT	0.229722804	7.74E-08
3078	ARL15	-0.382310666	4.60E-20
3079	CDK5RAP3	0.586747714	8.42E-51
3080	EIF4A3	0.146966491	0.000649803
3081	DDX54	0.332775807	2.67E-15
3082	SHKBP1	0.546569913	5.32E-43
3083	HOXD8	-0.227610007	1.02E-07
3084	OLA1	-0.173662229	5.39E-05
3085	ALDH1B1	-0.088442173	0.040864991
3086	RIPK1	-0.045114414	0.297602228
3087	THOC6	0.722702398	1.49E-87
3088	CRY2	-0.304034588	6.64E-13
3089	RNPEPL1	0.575144692	1.95E-48
3090	CHMP3	-0.525576374	2.51E-39
3091	ZHX2	-0.067141804	0.120874676
3092	GLB1L	0.109632239	0.011164011
3093	ACBD5	-0.449284651	6.13E-28
3094	THUMPD1	-0.417541837	5.51E-24
3095	VIMP	0.202025043	2.47E-06
3096	NAPA	0.036233746	0.402928003
3097	HMOX2	0.428627251	2.56E-25
3098	GDE1	-0.247876607	6.21E-09
3099	ACIN1	0.378567768	1.13E-19
3100	RPF1	-0.078526649	0.0695421
3101	ARGLU1	0.334316439	1.95E-15
3102	CDKN2AIPNL	0.163119103	0.000150926
3103	ABLIM3	0.05691274	0.188717686
3104	KIAA0040	-0.263601503	5.91E-10
3105	PYGL	0.060527635	0.162108354
3106	PTPLAD1	-0.494429765	2.50E-34
3107	TSPAN7	-0.332188227	3.00E-15
3108	KIAA0141	-0.085355743	0.048464967
3109	COQ10B	0.002784126	0.948773937
3110	DHX40	-0.328224849	6.65E-15
3111	UAP1	-0.078355722	0.070153384
3112	ACTR10	-0.325072536	1.24E-14
3113	BMPR2	-0.526481494	1.76E-39
3114	NIFK	0.191288127	8.38E-06
3115	DNAJC4	0.527700907	1.09E-39
3116	KLF13	-0.317083575	5.84E-14
3117	PTER	-0.28408611	2.17E-11
3118	MRPL11	0.226934088	1.12E-07
3119	SCRN1	-0.398625307	8.04E-22
3120	ITGA1	-0.351580485	5.20E-17
3121	SSFA2	-0.37609779	2.03E-19
3122	SMYD2	-0.183610571	1.93E-05
3123	NCAPH2	0.479408085	4.27E-32
3124	MFSD5	0.448244589	8.39E-28
3125	ISOC1	-0.299300997	1.56E-12
3126	COG4	0.296550046	2.54E-12
3127	RBL2	-0.489112732	1.59E-33
3128	NUDT16L1	0.207194868	1.34E-06
3129	PRKAG1	-0.247079152	6.97E-09
3130	RAB35	0.096702235	0.025303219

3131	FBXW4	0.242297814	1.38E-08
3132	MAFB	-0.073551346	0.089211652
3133	DVL1	0.483755329	9.89E-33
3134	HSBP1	-0.101033643	0.019416693
3135	SNX1	-0.290440346	7.36E-12
3136	MYO1B	-0.218426776	3.36E-07
3137	CRTC2	0.609708308	9.02E-56
3138	TIPRL	-0.070964416	0.10108001
3139	ANKRD40	-0.319647782	3.57E-14
3140	DHX15	-0.272691834	1.41E-10
3141	SART1	0.593786096	2.78E-52
3142	PPP1R8	-0.144603677	0.000795038
3143	ANG	0.138687951	0.001300304
3144	TNFAIP3	0.046443825	0.283578635
3145	CHMP2B	-0.434405336	4.93E-26
3146	AFG3L2	-0.402092802	3.30E-22
3147	SMIM20	-0.174420991	4.99E-05
3148	EMCN	-0.265717147	4.26E-10
3149	GPS1	0.610494799	5.99E-56
3150	TARS	-0.20792491	1.23E-06
3151	SMG7	-0.110518463	0.010522732
3152	EBP	0.376846397	1.70E-19
3153	MICU2	-0.344744683	2.24E-16
3154	TTC37	-0.42153404	1.85E-24
3155	LRRFIP1	-0.10267327	0.01752206
3156	CCND3	0.141451604	0.001035711
3157	NQO1	0.034726793	0.422786627
3158	POLR1D	0.01583618	0.714769492
3159	ABHD2	-0.546665737	5.11E-43
3160	UBE2G2	0.112868913	0.008977404
3161	ITPKC	0.292167748	5.46E-12
3162	UBD	0.091115921	0.035119977
3163	ARRB2	0.344244304	2.49E-16
3164	MRPL27	0.484235249	8.41E-33
3165	SGCE	-0.106811828	0.013441173
3166	PRMT5	-0.293887601	4.05E-12
3167	PAPOLA	-0.383909294	3.13E-20
3168	IFI44	0.221354336	2.31E-07
3169	SCRN2	0.426038723	5.29E-25
3170	POSTN	-0.035731609	0.409482408
3171	PIGS	-0.248661113	5.55E-09
3172	LYPLA1	-0.336503442	1.25E-15
3173	GBP3	-0.005111782	0.906099281
3174	MYO6	-0.422159832	1.56E-24
3175	ACSM2B	0.019979074	0.644739439
3176	RAB29	-0.059660243	0.168220151
3177	SALL1	-0.180912215	2.56E-05
3178	NELFCD	0.324491618	1.39E-14
3179	SMARCA1	-0.380892858	6.47E-20
3180	TULP3	0.120931031	0.005096247
3181	PHF23	0.25907212	1.18E-09
3182	TMEM139	-0.023165528	0.592899724
3183	GPKOW	0.182133041	2.25E-05
3184	MMGT1	-0.625745052	1.72E-59
3185	CSF1	0.324118644	1.50E-14
3186	TNPO2	0.160177801	0.000198982
3187	PRRC2C	-0.028470173	0.511110213
3188	URM1	0.411337481	2.93E-23

3189	FBXW11	-0.574144186	3.08E-48
3190	LDB2	-0.219654158	2.87E-07
3191	ACO1	-0.283793059	2.28E-11
3192	RUFY1	0.139127044	0.001254481
3193	ITFG3	0.516192214	9.11E-38
3194	RMND1	-0.04701167	0.277726855
3195	BOK	0.041452626	0.338582003
3196	BTN3A1	0.081975873	0.0581106
3197	SLC30A9	-0.610330746	6.53E-56
3198	PRUNE2	-0.229047413	8.47E-08
3199	CNP	0.23775508	2.60E-08
3200	PRMT2	0.106228598	0.013960165
3201	SMARCD1	0.401752549	3.61E-22
3202	TLR3	-0.299005367	1.64E-12
3203	CD276	0.217405734	3.82E-07
3204	FAM101B	-0.180331564	2.72E-05
3205	ARID5B	-0.360503208	7.30E-18
3206	NFIB	-0.187353354	1.29E-05
3207	STK25	0.449349985	6.01E-28
3208	BAP1	0.119037629	0.0058385
3209	C6orf211	-0.423451669	1.09E-24
3210	SLC25A4	-0.370697729	7.17E-19
3211	CLNS1A	-0.170530382	7.36E-05
3212	TMEM101	-0.135172234	0.001726624
3213	WWP1	-0.48400371	9.09E-33
3214	SNRNP27	-0.208909082	1.09E-06
3215	C9orf78	0.149344086	0.000528826
3216	GZMA	0.308621675	2.86E-13
3217	UGDH	-0.147345307	0.000628949
3218	DTD1	0.170173656	7.63E-05
3219	CLDND1	-0.027873082	0.520014341
3220	NOP58	0.39390587	2.65E-21
3221	STARD3NL	-0.260942024	8.90E-10
3222	WDR91	0.286242289	1.51E-11
3223	FOLR2	-0.016635556	0.70104656
3224	USP9X	-0.506230424	3.66E-36
3225	PRRC2B	-0.331526135	3.43E-15
3226	SSU72	0.319563063	3.63E-14
3227	SPTSSA	-0.186408842	1.43E-05
3228	DENND5A	-0.17431128	5.05E-05
3229	CLN6	0.41488148	1.13E-23
3230	SLK	-0.497878264	7.39E-35
3231	CCDC94	0.493370753	3.62E-34
3232	SNX33	0.057741228	0.182352221
3233	SLC16A9	-0.132157852	0.002190452
3234	WDR18	0.548645649	2.23E-43
3235	IFIT1	-0.170507034	7.38E-05
3236	8-Sep	-0.06335582	0.14333955
3237	YTHDC1	-0.214554358	5.46E-07
3238	BROX	-0.405137074	1.50E-22
3239	ZDHHC6	-0.140972454	0.001077694
3240	PPARD	0.16251518	0.000159802
3241	MGRN1	0.306186074	4.48E-13
3242	SIAH2	0.029811795	0.491396785
3243	THEM6	0.064493986	0.136273567
3244	ANGPT2	-0.079023887	0.067788584
3245	PSMA4	0.388201258	1.10E-20
3246	HNRNPR	-0.302573954	8.65E-13

3247	EXOSC5	0.514488554	1.73E-37
3248	NAPRT	0.489166346	1.56E-33
3249	NUDT2	0.195720121	5.10E-06
3250	TSPAN6	-0.334862985	1.75E-15
3251	REEP3	-0.481804885	1.91E-32
3252	TICAM1	0.27558332	8.85E-11
3253	DNTTIP1	0.526562444	1.71E-39
3254	MED16	0.332927887	2.59E-15
3255	CITED4	0.350996483	5.90E-17
3256	HERPUD2	-0.128126149	0.002988873
3257	NDNL2	0.003563783	0.934457454
3258	OLFML3	0.126455949	0.003391113
3259	ADSS	-0.285058144	1.84E-11
3260	RAB3GAP1	-0.399672544	6.15E-22
3261	CMTM3	0.349222868	8.64E-17
3262	FDFT1	-0.243596904	1.15E-08
3263	ALAD	-0.115597103	0.007439719
3264	FNDC3B	-0.270661963	1.95E-10
3265	UFL1	-0.444370598	2.67E-27
3266	SPI1	0.411975751	2.47E-23
3267	ZDHHC4	-0.300878089	1.17E-12
3268	SF3B3	-0.290952681	6.74E-12
3269	MPG	0.505263711	5.21E-36
3270	SMAD7	0.026363692	0.542873513
3271	PVR	0.090796042	0.035768912
3272	UBE2Q2	-0.450873356	3.79E-28
3273	LMO2	-0.103842195	0.016271957
3274	MYD88	0.097517658	0.024089881
3275	SRP68	-3.84E-05	0.999293021
3276	BAZ2A	-0.087237581	0.043701975
3277	RGL2	0.333138093	2.48E-15
3278	MPHOSPH8	-0.12294619	0.004400684
3279	RRP36	0.292379871	5.26E-12
3280	TMEM2	-0.135569953	0.001672662
3281	DEDD	0.355526334	2.20E-17
3282	CSNK1D	0.433164106	7.04E-26
3283	PNPO	-0.381861541	5.13E-20
3284	VMA21	-0.324008152	1.53E-14
3285	CCDC167	0.552714291	3.99E-44
3286	AATF	0.220231026	2.67E-07
3287	UPF1	0.136646759	0.001534235
3288	C7orf50	0.398075027	9.25E-22
3289	C9orf16	0.538828477	1.29E-41
3290	MED11	0.378927556	1.04E-19
3291	LCMT1	0.063806041	0.140511688
3292	RXRB	0.231624053	6.00E-08
3293	SYAP1	-0.217889772	3.59E-07
3294	RBBP9	-0.373025243	4.17E-19
3295	DPY30	0.240669565	1.73E-08
3296	TRPT1	0.570738635	1.45E-47
3297	SPTLC2	-0.499056212	4.86E-35
3298	CCNG2	-0.544364111	1.33E-42
3299	RANBP9	-0.353888438	3.15E-17
3300	SNCG	0.289808373	8.21E-12
3301	STRA13	0.559770373	1.90E-45
3302	SGPL1	-0.178588337	3.26E-05
3303	PNKD	0.402223709	3.19E-22
3304	BCL2	-0.343050262	3.21E-16

3305	RAC2	0.358455353	1.15E-17
3306	ADAM15	0.365176326	2.54E-18
3307	DCAF6	-0.332427157	2.86E-15
3308	MAN1B1	0.355730705	2.10E-17
3309	CD47	-0.14979559	0.000508362
3310	NOP56	0.609237063	1.15E-55
3311	ZNF22	0.022241246	0.607740267
3312	CSK	0.520949358	1.50E-38
3313	PPFIBP1	-0.23892748	2.21E-08
3314	FMO1	-0.05950056	0.169363867
3315	EXOC3	0.079334425	0.066711985
3316	DPM2	0.519566638	2.54E-38
3317	COA6	0.338082263	9.02E-16
3318	PARVA	-0.38801189	1.15E-20
3319	FCHO2	-0.445086168	2.16E-27
3320	KCNJ15	-0.207569771	1.28E-06
3321	SMARCA5	-0.447101475	1.18E-27
3322	PACS1	0.115103708	0.007699013
3323	FUNDC1	-0.062319699	0.15001266
3324	RNF44	0.198167789	3.86E-06
3325	PCGF5	-0.594993359	1.54E-52
3326	GALNT2	0.156438819	0.000280835
3327	CPEB2	-0.108688162	0.011885005
3328	HOXD9	0.128803171	0.002838572
3329	CERK	-0.113982831	0.008318308
3330	CALCOCO1	-0.036854806	0.394908623
3331	DYNC1LI2	-0.437746602	1.88E-26
3332	KIRREL	-0.063541463	0.142168286
3333	CHMP6	0.354225181	2.92E-17
3334	EEF1D	0.296142902	2.73E-12
3335	GRSF1	-0.480278123	3.19E-32
3336	MBOAT7	0.460933908	1.69E-29
3337	EP300	-0.359438786	9.25E-18
3338	XAB2	0.499994659	3.48E-35
3339	TSEN34	0.369181919	1.02E-18
3340	MAP4K4	-0.020705796	0.632751638
3341	NOC2L	0.307964713	3.23E-13
3342	NDUFAF2	0.173827083	5.30E-05
3343	ABCF2	0.158724581	0.000227702
3344	HYAL1	-0.233694684	4.54E-08
3345	PYGO2	0.49500134	2.04E-34
3346	GLA	0.213281475	6.39E-07
3347	CYB561	0.281862294	3.15E-11
3348	ZBTB7B	0.236828003	2.95E-08
3349	FAM213B	0.215658735	4.76E-07
3350	EBPL	0.081299762	0.06021961
3351	C16orf58	-0.054275265	0.210068106
3352	SLU7	-0.447098706	1.18E-27
3353	RERG	-0.278529405	5.47E-11
3354	ZC3H11A	0.019413021	0.654141324
3355	PREX1	-0.04625321	0.285561498
3356	PPID	-0.151259437	0.000446967
3357	HDHD3	0.364047294	3.29E-18
3358	COPS2	-0.451749958	2.90E-28
3359	CRB3	-0.202009556	2.47E-06
3360	CBX6	-0.058190667	0.178966205
3361	IL4R	0.331727969	3.30E-15
3362	TUSC1	-0.046288432	0.285194405

3363	AP1G1	-0.450472944	4.28E-28
3364	NEO1	-0.247806169	6.28E-09
3365	ST6GALNAC6	0.114851943	0.007834411
3366	GIMAP8	-0.16216627	0.00016515
3367	ACOT7	0.228114764	9.58E-08
3368	CYGB	0.194601111	5.79E-06
3369	ZNF428	0.487664487	2.61E-33
3370	RBBP7	0.038847647	0.369833362
3371	CISD3	0.320979452	2.76E-14
3372	FUT11	-0.002407256	0.955700433
3373	IFT52	-0.176334028	4.11E-05
3374	C6orf47	0.020565509	0.635058428
3375	ZFAND6	-0.282807216	2.69E-11
3376	ABHD11	0.230501822	6.97E-08
3377	URI1	-0.431088296	1.27E-25
3378	RFC2	0.387801002	1.21E-20
3379	PRSS8	-0.105678125	0.014466107
3380	APOM	0.112491133	0.00921127
3381	RNF20	-0.450041054	4.87E-28
3382	CHST15	-0.008343262	0.847325356
3383	MFSD12	0.411692233	2.66E-23
3384	FAM3C	-0.425586782	6.00E-25
3385	DCAF11	-0.191443378	8.23E-06
3386	TAF9	0.154661565	0.000329928
3387	TIMM17A	-0.128553726	0.00289313
3388	MLLT1	0.263915078	5.63E-10
3389	KIAA1147	-0.42338992	1.11E-24
3390	KDM2A	0.017436942	0.68738608
3391	UTRN	-0.421173255	2.04E-24
3392	FAM110C	-0.100838481	0.019653683
3393	EBNA1BP2	0.224614828	1.52E-07
3394	LAMA4	-0.204617646	1.82E-06
3395	CBFB	0.023679026	0.584727063
3396	NPLOC4	0.399341226	6.70E-22
3397	GYG1	-0.188187187	1.18E-05
3398	PCGF2	0.386044682	1.86E-20
3399	GLOD4	-0.057359248	0.185267056
3400	OSBPL9	-0.353311157	3.57E-17
3401	CIAPIN1	0.063928745	0.13974845
3402	P4HA2	0.045756116	0.290776385
3403	WWC1	-0.153855248	0.000354744
3404	TRIB1	-0.005080265	0.906675592
3405	VSIG4	0.031527959	0.466785581
3406	HDAC3	0.05749445	0.184231442
3407	HOXB7	0.106403506	0.0138027
3408	PITPNB	-0.203839211	2.00E-06
3409	SLC39A5	0.054942737	0.204506943
3410	C14orf2	0.293406646	4.40E-12
3411	PPP6R1	0.323310409	1.75E-14
3412	ACSS2	0.036664598	0.397354382
3413	PGPEP1	-0.099807368	0.020948062
3414	TNIP2	0.710078901	3.04E-83
3415	NDUFS8	0.476824854	1.01E-31
3416	CD200	-0.025281507	0.559564511
3417	ACE2	-0.155571754	0.000303864
3418	BCL2L2	-0.250555658	4.21E-09
3419	ANKIB1	-0.204821861	1.78E-06
3420	EPS15	-0.392900317	3.41E-21

3421	PIGR	-0.147577225	0.000616489
3422	PHF10	-0.240207742	1.85E-08
3423	TOM1	0.219874803	2.79E-07
3424	HINT3	-0.616097061	3.15E-57
3425	ELP5	0.495598745	1.66E-34
3426	KBTBD2	-0.184837815	1.69E-05
3427	NNT	-0.473823642	2.71E-31
3428	SSBP3	0.107325319	0.012998391
3429	LAMTOR3	-0.475626134	1.50E-31
3430	SCO2	0.743894565	2.37E-95
3431	ING4	0.217342178	3.85E-07
3432	FAM89B	0.573064073	5.05E-48
3433	NUDCD3	-0.093380888	0.030805595
3434	ETHE1	0.289991422	7.96E-12
3435	MPEG1	-0.166475571	0.000109456
3436	FKBP5	-0.016409411	0.704919174
3437	ITPK1	0.175096224	4.66E-05
3438	ZRANB2	0.125917999	0.003530771
3439	CTNNBL1	0.270781128	1.92E-10
3440	VAPA	-0.462467324	1.04E-29
3441	RRN3	-0.219874018	2.79E-07
3442	RAP2A	-0.387022838	1.46E-20
3443	C19orf24	0.621029926	2.25E-58
3444	SAP30	0.057457006	0.184517824
3445	DEPTOR	-0.348403894	1.03E-16
3446	KIAA0196	-0.459381962	2.75E-29
3447	BCL6	0.142321927	0.00096329
3448	RDH14	-0.088206221	0.041408097
3449	CBX5	-0.397438785	1.09E-21
3450	LSM7	0.783464635	3.34E-112
3451	PDGFA	0.282963603	2.62E-11
3452	DHTKD1	-0.148018228	0.000593427
3453	PTPRK	-0.441714163	5.87E-27
3454	RHOT2	0.617136391	1.82E-57
3455	SMYD5	0.263993204	5.57E-10
3456	ARMCX2	-0.214670036	5.38E-07
3457	WRNIP1	0.107921273	0.012500675
3458	ATF2	-0.309452218	2.45E-13
3459	TRIM47	0.621584364	1.66E-58
3460	MINK1	0.184289566	1.79E-05
3461	TM2D2	-0.199567647	3.29E-06
3462	RPL7L1	-0.15935589	0.000214779
3463	TNFRSF11B	-0.258099863	1.37E-09
3464	XPO1	-0.10237543	0.017853703
3465	7-Mar	-0.284961668	1.87E-11
3466	UGT1A9	-0.082685655	0.055962782
3467	PGF	0.15901689	0.000221631
3468	TOMM40	0.629915683	1.71E-60
3469	CPTP	0.384588787	2.65E-20
3470	CPD	-0.486309743	4.15E-33
3471	KDM5C	0.229994017	7.46E-08
3472	TRAF7	0.307298345	3.65E-13
3473	MRPL32	0.101642109	0.018693724
3474	STAB1	0.222993311	1.87E-07
3475	XPO7	-0.341675255	4.28E-16
3476	PREPL	-0.431445328	1.15E-25
3477	ROBO4	-0.053009129	0.220915392
3478	C14orf1	0.066599992	0.123909849

3479	CLDN7	-0.084132211	0.051789262
3480	HECTD3	0.379133689	9.86E-20
3481	CHTOP	0.454839257	1.13E-28
3482	SSBP1	0.291927748	5.69E-12
3483	OCEL1	0.463606689	7.28E-30
3484	LSM10	0.594984731	1.54E-52
3485	COL5A2	-0.019370932	0.654842629
3486	IFIT2	-0.225938846	1.28E-07
3487	ATP6V0D1	0.012381457	0.77508872
3488	BLOC1S2	-0.142361216	0.000960134
3489	C9orf142	0.757265358	1.11E-100
3490	BEX2	-0.194018719	6.18E-06
3491	IKBIP	0.079095248	0.067539931
3492	TNKS2	-0.409195725	5.17E-23
3493	ITFG1	-0.605625856	7.39E-55
3494	PRKD2	0.388266384	1.08E-20
3495	CYB5B	-0.25579741	1.94E-09
3496	DBN1	0.110566086	0.010489216
3497	TJP2	-0.30518463	5.38E-13
3498	FAM168A	-0.468321362	1.62E-30
3499	DOCK1	-0.352620377	4.15E-17
3500	COMMD10	-0.390516895	6.18E-21
3501	FOSB	-0.093248604	0.031044472
3502	SLC35B3	-0.209681929	9.93E-07
3503	ZNF362	0.121091875	0.005037292
3504	RBPJ	-0.317332373	5.57E-14
3505	BCL3	0.61101661	4.57E-56
3506	CSNK1A1	-0.430498938	1.51E-25
3507	THYN1	0.265968928	4.09E-10
3508	PCK2	0.117746244	0.006399077
3509	CANT1	-0.007577194	0.861194686
3510	UNC45A	0.21994021	2.77E-07
3511	PGAP3	0.12842006	0.002922756
3512	NDUFC2	-0.083089235	0.054771224
3513	SEC13	0.299459877	1.51E-12
3514	SEC14L6	0.032737643	0.449855884
3515	GTF3C5	0.466856637	2.59E-30
3516	IFT57	-0.100707154	0.019814573
3517	LETMD1	-0.155765687	0.000298565
3518	NR2F6	0.456626273	6.48E-29
3519	DCAKD	0.366401785	1.92E-18
3520	OGFR	0.623232711	6.80E-59
3521	INTS1	0.257235776	1.56E-09
3522	NFKBIL1	0.312206437	1.47E-13
3523	ZNF330	-0.353675563	3.30E-17
3524	FRMD8	0.276825276	7.23E-11
3525	TYK2	0.583552491	3.85E-50
3526	LRRC47	0.033538385	0.438843011
3527	EMC3	-0.2196987	2.86E-07
3528	S100A1	0.127453791	0.003145268
3529	UBAC1	0.284826319	1.92E-11
3530	NUBP1	0.062702934	0.147517435
3531	C1RL	0.2548425	2.23E-09
3532	RRM1	-0.345542422	1.90E-16
3533	CCDC53	0.126494579	0.00338128
3534	SEC63	-0.427793665	3.23E-25
3535	CSNK2A2	-0.1413306	0.001046168
3536	FBRS	0.503701362	9.18E-36

3537	GOLT1B	-0.133889629	0.001911704
3538	THBS2	-0.002371593	0.956356048
3539	RTCA	-0.093016873	0.031466751
3540	MAL2	-0.263096524	6.39E-10
3541	TMCC1	0.028839147	0.50564785
3542	LTBP3	0.085392668	0.048367487
3543	CDC42EP4	-0.039478961	0.362100178
3544	ERBB2IP	-0.448079404	8.81E-28
3545	TCEAL1	-0.268997794	2.54E-10
3546	LUC7L3	0.437176014	2.21E-26
3547	CTDSPL	-0.460942709	1.69E-29
3548	BZW1	-0.184093157	1.83E-05
3549	GFPT1	-0.402385652	3.06E-22
3550	BCL2L13	-0.381274815	5.91E-20
3551	VPS52	-0.002502847	0.953943225
3552	LYG1	0.097908146	0.023526875
3553	TMEM63B	0.213223868	6.43E-07
3554	PCMTD1	-0.362178046	5.01E-18
3555	MYO1D	-0.321754891	2.37E-14
3556	RNF144B	-0.24920502	5.12E-09
3557	HK2	-0.039840021	0.357723193
3558	PMM1	0.114357847	0.008106331
3559	SNN	-0.234608003	4.01E-08
3560	ENTPD6	0.388609392	9.91E-21
3561	GATAD1	0.030257259	0.484942456
3562	ORAI3	0.403870291	2.08E-22
3563	BCLAF1	-0.365996549	2.11E-18
3564	ATPAF1	-0.31955165	3.64E-14
3565	MYEOV2	0.496631843	1.15E-34
3566	WDR26	-0.229236194	8.26E-08
3567	ULK3	0.563121096	4.38E-46
3568	ACVR1	-0.242674736	1.31E-08
3569	ASL	0.184539957	1.74E-05
3570	SLC25A13	-0.287884586	1.14E-11
3571	SERPINE2	0.145378822	0.000744374
3572	DPYS	0.02016385	0.641682521
3573	MDFIC	-0.224749131	1.49E-07
3574	CBX7	-0.13950825	0.00121591
3575	ABHD4	-0.022067147	0.610554039
3576	MFF	0.020439828	0.637128036
3577	SMEK1	-0.06379191	0.140599795
3578	TP53INP1	-0.109064819	0.011592557
3579	ATMIN	-0.480759318	2.71E-32
3580	HMGCL	-0.063831579	0.140352574
3581	SAFB	0.452597096	2.24E-28
3582	SH3BP4	-0.308785095	2.78E-13
3583	KLHDC8B	0.164918763	0.000127142
3584	SREBF1	0.225349198	1.38E-07
3585	VPS36	-0.358378028	1.17E-17
3586	GOLGA2	0.129225275	0.002748381
3587	GCN1L1	-0.051566338	0.233757476
3588	TPPP3	0.214283527	5.64E-07
3589	IL3RA	0.143644737	0.000862129
3590	RND3	0.007970537	0.854067771
3591	EML4	-0.093721526	0.030197705
3592	MARVELD1	0.093306397	0.030939915
3593	SPCS1	-0.176494865	4.04E-05
3594	AP2A2	0.106484833	0.013730018

3595	PPP6R3	-0.297026363	2.33E-12
3596	CUL7	0.18713396	1.32E-05
3597	HARS2	0.15894476	0.000223115
3598	HN1	0.528581331	7.74E-40
3599	FBXO21	-0.465732221	3.70E-30
3600	IRF3	0.738003231	4.16E-93
3601	DCAF12	-0.295014605	3.32E-12
3602	C1orf186	0.188777577	1.10E-05
3603	PSMD11	0.224221498	1.60E-07
3604	UBE2A	-0.036336544	0.401593957
3605	TNC	-0.026399054	0.542332306
3606	KIAA1522	-0.011367967	0.793062952
3607	C7	-0.078577065	0.069362632
3608	OAS1	0.158377889	0.000235105
3609	DLAT	-0.582480615	6.39E-50
3610	EVA1B	0.453112956	1.91E-28
3611	RTF1	-0.181156868	2.49E-05
3612	PTGES2	0.410414459	3.74E-23
3613	GLE1	-0.036683091	0.39711619
3614	NFKB1	-0.186563998	1.40E-05
3615	RNF135	0.339838195	6.27E-16
3616	ARHGAP35	-0.454676623	1.18E-28
3617	FLYWCH2	0.53330055	1.20E-40
3618	NSMF	0.278454148	5.54E-11
3619	NSDHL	0.081318221	0.060161202
3620	NR1H4	-0.032788713	0.449148859
3621	RAB17	0.062071548	0.151645401
3622	NPR1	0.040992076	0.343980512
3623	WDR46	0.416287419	7.75E-24
3624	CGNL1	-0.381469154	5.64E-20
3625	ANTXR1	-0.147138961	0.00064023
3626	SH3BP2	0.143288593	0.000888352
3627	PIP4K2A	-0.073141424	0.091013937
3628	SELENBP1	0.140524514	0.001118357
3629	FMNL2	0.040866352	0.345463705
3630	TBC1D17	0.491867118	6.11E-34
3631	SMARCC2	-0.105384114	0.014742884
3632	PARN	0.049413215	0.25388875
3633	PIEZO1	0.325717892	1.09E-14
3634	DCTN3	0.042476074	0.326780623
3635	MTIF2	-0.189415788	1.03E-05
3636	ST14	-0.12128892	0.004965907
3637	LIMS1	-0.33015345	4.52E-15
3638	DCAF8	0.286364561	1.48E-11
3639	TRAFD1	0.165179687	0.000124002
3640	SIPA1	0.531033769	2.94E-40
3641	MBD4	0.123218606	0.004313552
3642	KPNA6	-0.410892237	3.29E-23
3643	GRAMD1A	0.618548272	8.54E-58
3644	AUH	-0.334544048	1.86E-15
3645	ETFB	0.349535274	8.08E-17
3646	SEC24B	-0.577204627	7.52E-49
3647	NUDT14	0.399528467	6.38E-22
3648	PRAF2	0.405222549	1.47E-22
3649	HBA2	0.140861413	0.001087645
3650	THRA	-0.077277683	0.074110815
3651	COA5	0.15313094	0.000378511
3652	ASCC2	0.61668185	2.31E-57

3653	TTC3	-0.320232813	3.19E-14
3654	MTFR1L	-0.071994233	0.096213259
3655	MRTO4	0.430258259	1.61E-25
3656	PSIP1	-0.259050355	1.19E-09
3657	C14orf119	0.140102709	0.001157936
3658	REN	0.053620934	0.215624925
3659	CHRAC1	0.200286111	3.02E-06
3660	DNAJB12	0.248348859	5.80E-09
3661	EIF2S1	-0.313855347	1.08E-13
3662	SERPINF1	0.24558052	8.64E-09
3663	KMT2E	-0.232792341	5.13E-08
3664	NFIX	-0.079956454	0.064597703
3665	MORN2	0.171386733	6.76E-05
3666	PAPSS2	-0.115134962	0.007682352
3667	SLC7A7	0.119594362	0.005610755
3668	ZNF24	-0.464577556	5.35E-30
3669	COX17	0.346620942	1.51E-16
3670	LPIN2	-0.233518351	4.65E-08
3671	MRPS27	-0.244972759	9.43E-09
3672	APBB1IP	0.149327965	0.00052957
3673	TBCC	0.193529559	6.53E-06
3674	MLLT6	0.174643788	4.88E-05
3675	MED8	0.387753957	1.22E-20
3676	GPC4	-0.307228065	3.70E-13
3677	MRPS6	0.069163245	0.110060194
3678	FAM127C	0.083917302	0.05239237
3679	AIM1	-0.184804321	1.70E-05
3680	GLRX5	-0.131618114	0.002284638
3681	CCNB1IP1	-0.099955189	0.020758065
3682	NIT1	0.429252121	2.14E-25
3683	MRPL36	0.415115005	1.06E-23
3684	ELMO3	0.168718985	8.80E-05
3685	MSC	0.474113712	2.46E-31
3686	SLC27A2	-0.19054595	9.09E-06
3687	TCIRG1	0.660760339	2.04E-68
3688	C4orf27	-0.109771662	0.011060883
3689	SSBP4	0.691553392	2.53E-77
3690	MS4A7	-0.114206952	0.008191038
3691	TMEM50B	-0.213489056	6.23E-07
3692	MIF4GD	0.303659679	7.10E-13
3693	PCK1	-0.14898999	0.000545407
3694	TIMM17B	0.539688503	9.09E-42
3695	VAV3	-0.387821806	1.20E-20
3696	MME	0.006463308	0.881436353
3697	SLC12A4	0.042897033	0.322004823
3698	ARMCX1	-0.16668794	0.000107231
3699	KIAA1033	-0.351323271	5.50E-17
3700	PTDSS1	-0.083456363	0.053705686
3701	CFAP20	0.05035784	0.244913218
3702	MRPS12	0.445488251	1.92E-27
3703	RFK	-0.311104413	1.80E-13
3704	SLC25A38	-0.001617816	0.970219736
3705	HMGXB3	0.396766721	1.29E-21
3706	PALLD	-0.218797923	3.20E-07
3707	WWC3	0.014260134	0.742092867
3708	KDSR	-0.458184876	4.00E-29
3709	MYLK	-0.265448986	4.44E-10
3710	SERTAD3	0.366692799	1.80E-18

3711	PPP6R2	0.445486695	1.92E-27
3712	PSMD12	-0.148858384	0.000551692
3713	DAPK3	0.55002668	1.25E-43
3714	FRG1	-0.020464096	0.636728183
3715	GBAS	-0.347809924	1.17E-16
3716	SLC2A5	0.05023899	0.246030122
3717	LYRM1	0.027736972	0.522055177
3718	OTUD1	-0.38687173	1.52E-20
3719	TPCN1	0.09052252	0.036331867
3720	RAB4A	-0.417167701	6.10E-24
3721	DEDD2	0.388962631	9.08E-21
3722	PDHB	-0.223970225	1.65E-07
3723	AGMAT	0.009915063	0.819020254
3724	PDXK	0.202094105	2.45E-06
3725	STK40	0.072063613	0.095892221
3726	SERTAD1	0.202360306	2.38E-06
3727	PSMA5	0.264096864	5.48E-10
3728	AKAP1	-0.226749115	1.15E-07
3729	OGDHL	-0.254898218	2.22E-09
3730	NXT1	0.525335306	2.75E-39
3731	UBA7	0.477913095	7.02E-32
3732	SPSB1	0.181781384	2.34E-05
3733	CRCP	-0.16032245	0.000196317
3734	DDX39A	1	0
3735	SIRT2	0.404138397	1.94E-22
3736	BMI1	-0.470397851	8.26E-31
3737	CACYBP	-0.001593826	0.970661147
3738	ADAM10	-0.484714564	7.14E-33
3739	SAFB2	0.432653152	8.15E-26
3740	CDR2	0.202669078	2.29E-06
3741	RBM12	-0.429527282	1.98E-25
3742	CASKIN2	-0.011152528	0.796898271
3743	MS4A6A	0.025378557	0.558057584
3744	RAF1	-0.015759181	0.716096285
3745	SEC16A	-0.055307781	0.201511067
3746	WDR5	0.267128223	3.41E-10
3747	SURF2	0.545285749	9.08E-43
3748	ZC3HC1	0.271188503	1.80E-10
3749	ATAD1	-0.519498435	2.60E-38
3750	ERLIN2	-0.527809012	1.05E-39
3751	NCKIPSD	0.057320035	0.185568216
3752	SUN1	-0.072984361	0.091712177
3753	UBA5	-0.07831815	0.070288341
3754	KIAA0319L	-0.211611572	7.85E-07
3755	VPS39	-0.021342538	0.622326484
3756	AP2A1	0.418811826	3.90E-24
3757	POLG	0.304316481	6.31E-13
3758	XPO6	0.192163646	7.60E-06
3759	CELF1	0.076050803	0.078834037
3760	PLN	-0.21581648	4.66E-07
3761	PELI1	-0.121654578	0.004835849
3762	SIAE	-0.458368687	3.77E-29
3763	STIM1	-0.173125316	5.69E-05
3764	RIPK4	-0.126765569	0.003313025
3765	APOL6	-0.170818152	7.16E-05
3766	SGPP1	-0.202016981	2.47E-06
3767	TMEM243	0.059677729	0.168095259
3768	TSPAN13	-0.054121913	0.21136106

3769	TRIM44	-0.492444518	5.00E-34
3770	MYH11	-0.098892515	0.022157886
3771	GNAI1	-0.359611689	8.90E-18
3772	RMND5A	-0.433775295	5.91E-26
3773	AKAP8	0.331057244	3.77E-15
3774	TIE1	-0.007596474	0.860845083
3775	CD163	-0.127097247	0.003231188
3776	KLF11	-0.46632433	3.07E-30
3777	MTMR12	-0.562906526	4.81E-46
3778	GRPEL1	-0.113850646	0.008394195
3779	METAP1	-0.40271591	2.81E-22
3780	CCDC6	-0.438708811	1.42E-26
3781	ARMC1	-0.313852158	1.08E-13
3782	DLGAP4	0.460243481	2.10E-29
3783	NDUFA5	-0.346562573	1.53E-16
3784	HMGA1	0.259124749	1.17E-09
3785	PARD3	-0.232611668	5.25E-08
3786	FUNDC2	-0.097331254	0.024362719
3787	VOPP1	0.196354555	4.75E-06
3788	COX4I2	0.095105626	0.02783229
3789	VARS	0.351284395	5.54E-17
3790	PPIL1	-0.164627268	0.000130739
3791	ZNF581	0.336461628	1.26E-15
3792	LSM14B	0.180776098	2.60E-05
3793	CTR9	-0.514589805	1.66E-37
3794	RASL11A	-0.013301764	0.758870292
3795	C6orf120	-0.470498685	8.00E-31
3796	SAMD1	0.620347161	3.25E-58
3797	TMEM261	-0.010871548	0.801907746
3798	CHD3	0.047720044	0.270542579
3799	SNF8	0.602862984	3.01E-54
3800	ABCF3	0.352648815	4.12E-17
3801	THBD	-0.104782851	0.015323432
3802	MRPL35	-0.263654402	5.87E-10
3803	CST7	0.346093976	1.69E-16
3804	EMC2	-0.291449614	6.19E-12
3805	KIAA1279	-0.39029418	6.53E-21
3806	PPP1R2	-0.387521643	1.29E-20
3807	TRAK2	-0.454009702	1.45E-28
3808	IL1R1	-0.102963199	0.017204411
3809	CIRH1A	-0.122333727	0.004602417
3810	ACAD9	0.086426166	0.045705031
3811	METTL23	0.434715641	4.51E-26
3812	RUSC2	0.230051357	7.41E-08
3813	SFT2D1	-0.007599517	0.860789914
3814	TIMM10B	-0.436966484	2.35E-26
3815	XPOT	-0.115355938	0.007565467
3816	PPTC7	-0.313549538	1.14E-13
3817	FOXO3	-0.395224302	1.90E-21
3818	SLC17A1	0.128778605	0.002843903
3819	RANGAP1	0.529419028	5.57E-40
3820	MCMBP	-0.266591724	3.71E-10
3821	PRKCD	0.083457151	0.05370342
3822	MPV17	0.48996387	1.18E-33
3823	ABCG1	-0.195186134	5.42E-06
3824	MRPL2	0.377524112	1.45E-19
3825	FBLN5	-0.018278407	0.67315118
3826	FBXL3	-0.552064352	5.26E-44

3827	IPO8	-0.587984428	4.65E-51
3828	KTN1	-0.374142557	3.21E-19
3829	EEFSEC	0.28295466	2.63E-11
3830	ZNFX1	-0.211111837	8.34E-07
3831	USP14	-0.353425145	3.48E-17
3832	ELAVL1	0.116860633	0.006810951
3833	TOR1A	0.067463344	0.119101059
3834	GNA12	-0.041714238	0.33553971
3835	COMMD8	-0.29870142	1.73E-12
3836	FAM136A	0.36673658	1.78E-18
3837	WIPF1	0.056916284	0.188690111
3838	AGPAT5	-0.405337781	1.42E-22
3839	MAZ	0.265621279	4.32E-10
3840	DDX18	-0.339430408	6.83E-16
3841	PTEN	-0.396390478	1.42E-21
3842	PDCL3	0.18337685	1.97E-05
3843	BAG5	-0.34880844	9.45E-17
3844	MRPL1	-0.250577566	4.19E-09
3845	HECTD1	-0.490666763	9.27E-34
3846	IP6K1	-0.154107006	0.000346815
3847	HIP1R	0.236047457	3.29E-08
3848	SECISBP2L	-0.616038508	3.25E-57
3849	TIPARP	-0.102866875	0.017309381
3850	SOX18	0.151897185	0.00042244
3851	RASSF3	-0.118302479	0.006151954
3852	NXF1	0.424758358	7.56E-25
3853	KIFAP3	-0.232660233	5.22E-08
3854	GPRC5B	-0.361428126	5.93E-18
3855	NOTCH4	0.03408369	0.431432504
3856	KLHL9	-0.423946777	9.48E-25
3857	BCAR1	0.38623349	1.78E-20
3858	CORO1A	0.432821079	7.77E-26
3859	DNAJB4	-0.334706662	1.80E-15
3860	USP12	-0.50408677	7.99E-36
3861	HIP1	-0.205106303	1.72E-06
3862	FTSJ2	0.018147365	0.675360491
3863	CPT2	-0.272491766	1.46E-10
3864	TMEM185B	-0.202193017	2.42E-06
3865	SLC35F5	-0.473278562	3.23E-31
3866	HBP1	-0.59356092	3.10E-52
3867	SEC11C	-0.136717498	0.001525521
3868	IWS1	0.173204095	5.64E-05
3869	NUP62	0.454211883	1.37E-28
3870	CXXC1	0.423220065	1.16E-24
3871	WDR45	0.49990369	3.60E-35
3872	SKIV2L2	-0.467118696	2.38E-30
3873	LPAR6	0.101280646	0.019120321
3874	MPND	0.403265457	2.44E-22
3875	STYXL1	0.146268123	0.000689939
3876	TEF	-0.304717385	5.86E-13
3877	TBC1D20	0.116952947	0.006766936
3878	QTRT1	0.682273026	1.59E-74
3879	KLF3	-0.477312994	8.56E-32
3880	TMEM222	0.379156166	9.81E-20
3881	RNF213	-0.033983298	0.43279134
3882	UBLCP1	-0.284813619	1.92E-11
3883	TRIB3	0.298783613	1.71E-12
3884	XRCC1	0.40178128	3.58E-22

3885	TRAPPC2L	0.32062139	2.96E-14
3886	SDF2	0.300990642	1.15E-12
3887	TUBG1	0.55954666	2.10E-45
3888	GSTA4	-0.126628385	0.00334742
3889	CBY1	0.358197942	1.22E-17
3890	BTN2A1	0.123422684	0.004249302
3891	MPRIP	-0.028756867	0.506863272
3892	NACC1	0.473765117	2.76E-31
3893	RNF146	-0.199765172	3.21E-06
3894	MLX	0.137304774	0.001454908
3895	C1orf198	-0.09154885	0.034257676
3896	DUSP5	0.16455785	0.00013161
3897	FAM198B	-0.395398086	1.82E-21
3898	RBP4	-0.052517574	0.225232764
3899	TEX264	0.194617826	5.78E-06
3900	KDM1A	-0.079256198	0.066981856
3901	SOAT1	-0.291057989	6.62E-12
3902	FEM1B	-0.533539004	1.09E-40
3903	TMEM57	-0.284407489	2.06E-11
3904	ATP6V1H	-0.293204491	4.56E-12
3905	FAM20B	-0.38368711	3.30E-20
3906	ITCH	-0.504266305	7.48E-36
3907	RABGGTB	-0.044634804	0.302773061
3908	RBMS2	-0.197286913	4.27E-06
3909	PDDC1	0.38824514	1.08E-20
3910	NAB2	0.434559617	4.72E-26
3911	ASB8	-0.446256762	1.52E-27
3912	ABI3	0.233125138	4.90E-08
3913	BLOC1S6	-0.473714506	2.80E-31
3914	KIAA0195	0.191504147	8.18E-06
3915	BCCIP	0.111584968	0.009794475
3916	LRP11	-0.338970623	7.51E-16
3917	CMTR1	0.027548155	0.524893105
3918	GPN1	-0.135958221	0.00162148
3919	LBR	-0.200428387	2.97E-06
3920	CD36	-0.162722952	0.000156694
3921	NUDT15	-0.243420298	1.18E-08
3922	RBM14	0.340851672	5.08E-16
3923	CDK7	0.161119502	0.000182223
3924	COPS4	-0.526136681	2.02E-39
3925	TSPAN31	-0.351524535	5.26E-17
3926	ARNT	-0.209520754	1.01E-06
3927	CPM	-0.189241116	1.05E-05
3928	TBC1D16	-0.187311903	1.29E-05
3929	TOR1AIP1	-0.502936028	1.21E-35
3930	TMEM91	0.25069842	4.12E-09
3931	TJP1	-0.361753561	5.51E-18
3932	WIPF2	-0.282701594	2.74E-11
3933	AGXT2	0.013267084	0.759479584
3934	KCTD11	0.174180479	5.11E-05
3935	NAT10	0.123456778	0.004238653
3936	C20orf27	0.535335736	5.29E-41
3937	RPS27L	0.04243452	0.327254534
3938	SCOC	-0.522879658	7.12E-39
3939	LYPLAL1	-0.190310839	9.33E-06
3940	UTP18	-0.161947282	0.000168593
3941	POLR3C	-0.032463982	0.453655143
3942	GATAD2A	0.268333139	2.82E-10

3943	FGFRL1	0.052305585	0.227113104
3944	MAVS	-0.081022785	0.061101672
3945	C7orf73	-0.002632549	0.951559338
3946	PEX5	-0.06899109	0.110950433
3947	CRLS1	-0.03367788	0.436940406
3948	ARL5A	-0.584582765	2.36E-50
3949	ATHL1	0.478113118	6.57E-32
3950	MTMR11	0.205509326	1.64E-06
3951	HLA-G	-0.003674674	0.932422826
3952	NDUFA10	-0.016064026	0.710848434
3953	OSBPL8	-0.574968842	2.11E-48
3954	RNF40	-0.087755406	0.042462721
3955	PNMA2	-0.064245058	0.137795593
3956	SYDE1	0.332269993	2.95E-15
3957	SFXN1	-0.199802566	3.20E-06
3958	HDGFRP2	0.581717854	9.15E-50
3959	FUBP3	-0.247070588	6.98E-09
3960	TRIM56	-0.008445251	0.845482355
3961	LPGAT1	-0.215670083	4.75E-07
3962	SYS1	-0.158753473	0.000227095
3963	ATRN	-0.548098467	2.81E-43
3964	SELPLG	0.262293413	7.23E-10
3965	FAM114A1	-0.079047391	0.067706605
3966	PELO	-0.316886051	6.06E-14
3967	NDUFA13	0.494988478	2.05E-34
3968	GTF2B	-0.038256121	0.377171294
3969	PSMG3	0.658205081	1.00E-67
3970	TOX4	-0.358696173	1.09E-17
3971	VAV2	-0.012307869	0.776389896
3972	MT1G	0.024039132	0.579027101
3973	KPNA2	0.243594723	1.15E-08
3974	PTPLB	-0.473826505	2.70E-31
3975	KCTD10	-0.292279244	5.36E-12
3976	MRPL21	0.313086459	1.24E-13
3977	TMEM38B	-0.316749454	6.22E-14
3978	MBD6	0.486174955	4.34E-33
3979	LARP7	-0.099500433	0.021347402
3980	USP33	-0.425271046	6.56E-25
3981	VPS16	0.598654293	2.50E-53
3982	NSUN2	0.228120149	9.58E-08
3983	GALE	0.406261472	1.12E-22
3984	NREP	-0.062206432	0.150756251
3985	B4GALT3	0.301041789	1.14E-12
3986	EVA1A	0.074305076	0.085972651
3987	CHMP7	0.03828092	0.376861865
3988	SPPL3	-0.038661677	0.372130745
3989	ELAC2	0.334127582	2.03E-15
3990	CCDC85B	0.416241247	7.84E-24
3991	CDC73	-0.472199573	4.60E-31
3992	ADPGK	0.281390536	3.41E-11
3993	ATF6	-0.503465242	1.00E-35
3994	ATP5SL	0.18158042	2.39E-05
3995	PITRM1	-0.008203167	0.849858321
3996	UNC13B	-0.178750427	3.21E-05
3997	GNL3	0.319615529	3.59E-14
3998	CSTF2T	-0.396423466	1.41E-21
3999	USE1	0.586744415	8.43E-51
4000	AFTPH	-0.262093226	7.46E-10

4001	PPP1R21	-0.117990021	0.006289696
4002	C7orf49	-0.00793597	0.854693613
4003	JKAMP	-0.390137645	6.79E-21
4004	TUBGCP2	0.246587548	7.48E-09
4005	TINAG	-0.024583076	0.570466908
4006	LRRK2	-0.220956273	2.43E-07
4007	TOR1B	-0.046208894	0.286023833
4008	STRN4	0.558009354	4.10E-45
4009	PXMP2	0.026182628	0.545648823
4010	KCTD9	-0.323054827	1.84E-14
4011	OSTM1	-0.235384083	3.60E-08
4012	CHST14	0.145297746	0.000749529
4013	NECAP1	-0.27947136	4.68E-11
4014	MRPL30	-0.359522474	9.08E-18
4015	TPR	-0.210808089	8.65E-07
4016	JOSD2	0.639710546	6.52E-63
4017	PSKH1	-0.234699759	3.96E-08
4018	SLC25A20	-0.014292363	0.74153074
4019	RAB8B	-0.386955575	1.49E-20
4020	POLE4	0.284940454	1.88E-11
4021	PTK2	-0.328942504	5.76E-15
4022	CUL4A	-0.257435102	1.52E-09
4023	ZNF217	-0.231883117	5.79E-08
4024	CD58	0.123464417	0.00423627
4025	MFAP4	0.098452545	0.022760984
4026	TSTA3	0.655690789	4.74E-67
4027	COQ4	0.497700314	7.88E-35
4028	RPE	-0.231187729	6.36E-08
4029	PMF1	0.464091788	6.24E-30
4030	PPIH	0.553908298	2.40E-44
4031	ALDH6A1	-0.426448948	4.72E-25
4032	HHLA2	0.034641589	0.423926266
4033	PDK2	-0.220836601	2.47E-07
4034	F13A1	-0.205791576	1.58E-06
4035	NCK1	-0.175390418	4.52E-05
4036	TFCP2	-0.29105049	6.63E-12
4037	ALDH8A1	-0.071464936	0.098690751
4038	SLTM	-0.002416478	0.955530901
4039	HCCS	-0.316112819	7.02E-14
4040	PPIL4	-0.29220643	5.43E-12
4041	MOSPD3	0.354450052	2.78E-17
4042	TYMS	0.048098668	0.266755156
4043	TMED1	-0.008123329	0.851302514
4044	SLC30A5	-0.364183878	3.19E-18
4045	ABCC1	0.060771262	0.160422147
4046	MPP1	-0.15185984	0.000423841
4047	GGCT	0.221037254	2.41E-07
4048	VPS18	-0.027539052	0.525030122
4049	DNAL4	0.012637792	0.770561188
4050	PIK3C2A	-0.533059799	1.32E-40
4051	PECR	-0.091517735	0.034319042
4052	CCAR2	0.315160561	8.41E-14
4053	SMO	0.144988049	0.000769529
4054	RAI14	-0.114529938	0.008010681
4055	RBFOX2	-0.353707915	3.27E-17
4056	MAPKAP1	-0.126272579	0.003438145
4057	CCNL2	0.541309246	4.68E-42
4058	ABCD3	-0.403202893	2.48E-22

4059	NUDT5	0.259907344	1.04E-09
4060	RNF121	-0.296036445	2.78E-12
4061	CCDC86	0.317603289	5.28E-14
4062	NDN	0.063578075	0.141938162
4063	ELK1	0.22816134	9.52E-08
4064	FARSB	0.232620506	5.25E-08
4065	CIC	0.179041418	3.11E-05
4066	DBNL	0.399287848	6.79E-22
4067	DHX38	0.06715899	0.120779359
4068	BLOC1S5	-0.256136449	1.84E-09
4069	REXO4	0.280360674	4.04E-11
4070	AP3B1	-0.543617989	1.81E-42
4071	RAI2	-0.224292464	1.58E-07
4072	ANKMY2	-0.45163683	3.00E-28
4073	NUDT9	-0.154419981	0.000337188
4074	MGAT5	-0.376988716	1.64E-19
4075	STMN1	0.253794748	2.61E-09
4076	TACO1	0.264894641	4.84E-10
4077	FAAH	0.161149974	0.000181703
4078	MOCS2	-0.331170194	3.69E-15
4079	NRIP2	-0.040757898	0.346746415
4080	TRIM4	-0.327234438	8.10E-15
4081	SMU1	-0.266281892	3.90E-10
4082	TFRC	-0.03903451	0.367533816
4083	PRKACB	-0.508076567	1.86E-36
4084	NIPSNAP3A	-0.208666585	1.12E-06
4085	HSD17B12	-0.538211976	1.66E-41
4086	ARV1	0.001471456	0.972912806
4087	ITGB8	-0.299829863	1.42E-12
4088	SMARCC1	-0.44275353	4.32E-27
4089	FAM91A1	-0.286391284	1.47E-11
4090	IFIT5	-0.367492636	1.50E-18
4091	RGS14	0.40358753	2.24E-22
4092	H2AFX	0.611147067	4.27E-56
4093	MTMR2	-0.359787769	8.56E-18
4094	NMRAL1	0.513990457	2.08E-37
4095	GLT8D1	0.025604657	0.554554525
4096	DUT	0.125547712	0.003629913
4097	PXDN	-0.027123397	0.531305895
4098	ATXN7L3	0.501500708	2.03E-35
4099	PRDM4	-0.345688543	1.84E-16
4100	PDCD6	0.319895705	3.40E-14
4101	MICA	0.232584825	5.27E-08
4102	ATOX1	0.346528617	1.54E-16
4103	EBAG9	-0.311005969	1.84E-13
4104	NUFIP2	-0.179511454	2.96E-05
4105	FILIP1L	-0.13747896	0.001434549
4106	CEBPZOS	0.101537562	0.018816249
4107	IRF1	0.341796849	4.17E-16
4108	PCDH1	-0.297543354	2.13E-12
4109	KAT2A	0.677320668	4.51E-73
4110	APOO	-0.059456689	0.169679104
4111	FGFR4	0.276107223	8.13E-11
4112	CCDC25	-0.439880955	1.01E-26
4113	ASMTL	0.24155013	1.53E-08
4114	CREBZF	0.118850575	0.005916864
4115	FEM1C	-0.248402063	5.76E-09
4116	C9orf64	-0.281854682	3.16E-11

4117	FPR3	-0.125629812	0.003607716
4118	TBX2	0.163078728	0.000151504
4119	PAX2	0.024968361	0.5644402
4120	HINT2	0.251824969	3.49E-09
4121	KCTD2	0.00401012	0.926270785
4122	CDS2	-0.473107783	3.42E-31
4123	G6PD	0.402796819	2.75E-22
4124	SFT2D2	-0.172877926	5.83E-05
4125	DNM1L	-0.138901212	0.00127786
4126	HIPK1	-0.439160225	1.24E-26
4127	SSH3	0.391952038	4.33E-21
4128	SNX19	-0.312794026	1.32E-13
4129	CNIH4	-2.32E-05	0.999572726
4130	TMEM150C	-0.297566399	2.12E-12
4131	STRADB	0.009528373	0.825963512
4132	BAK1	0.380629731	6.90E-20
4133	PPP2R3A	-0.386235968	1.77E-20
4134	CRELD1	0.139121034	0.001255098
4135	SERPINI1	-0.197662739	4.09E-06
4136	SRA1	0.540153511	7.52E-42
4137	DHX32	-0.208467927	1.15E-06
4138	FAM63A	-0.354159562	2.97E-17
4139	CAMKK2	0.189656931	1.00E-05
4140	LZTS2	0.569112045	3.03E-47
4141	EVI2B	0.051383449	0.235422273
4142	MAP3K11	0.43584979	3.25E-26
4143	NDUFS1	-0.385240886	2.26E-20
4144	NUMB	-0.180134728	2.78E-05
4145	FAM199X	-0.314860777	8.91E-14
4146	RNF38	-0.504268214	7.48E-36
4147	RRP7A	0.413959472	1.45E-23
4148	LY96	0.133047053	0.002042993
4149	PDS5A	-0.406151859	1.15E-22
4150	PINK1	-0.375926673	2.11E-19
4151	ZNF672	0.27553444	8.92E-11
4152	CNNM3	-0.143620343	0.000863902
4153	SPRED2	-0.243725171	1.13E-08
4154	EDEM1	-0.31201559	1.52E-13
4155	RBM17	0.358405134	1.16E-17
4156	BIN1	0.314795728	9.02E-14
4157	ENPP4	-0.599799836	1.41E-53
4158	XPC	-0.276189142	8.02E-11
4159	NCOA7	-0.410185357	3.97E-23
4160	SIK2	-0.385158176	2.31E-20
4161	CASP7	-0.170547	7.35E-05
4162	C14orf166	0.176669133	3.97E-05
4163	PRRC1	-0.472045797	4.84E-31
4164	SLC52A2	0.646492143	1.23E-64
4165	RENBP	0.257623282	1.47E-09
4166	PIK3R1	-0.268074163	2.94E-10
4167	TEAD1	-0.4560546	7.74E-29
4168	SPRY2	-0.157903717	0.000245595
4169	RSAD1	0.350747484	6.22E-17
4170	OXSR1	-0.317938538	4.96E-14
4171	SPPL2A	-0.327821651	7.20E-15
4172	SLC22A11	0.052287312	0.227275713
4173	NT5C3A	-0.055600599	0.199131112
4174	HDAC7	0.403238773	2.46E-22

4175	WDR54	0.289544824	8.59E-12
4176	ZBTB22	0.015445192	0.721515581
4177	TNPO1	-0.409350751	4.96E-23
4178	KIAA0430	-0.413080111	1.84E-23
4179	ZFYVE27	0.333336256	2.38E-15
4180	ARHGAP29	-0.260026415	1.02E-09
4181	NUP153	-0.3530379	3.79E-17
4182	PRPF4	-0.006892856	0.873620657
4183	UBTD2	-0.371416892	6.07E-19
4184	COMMD1	0.29133584	6.31E-12
4185	ARID1A	-0.202445155	2.35E-06
4186	IQSEC1	-0.21657441	4.24E-07
4187	PLRG1	-0.346347526	1.60E-16
4188	RNF4	-0.121149555	0.005016301
4189	PPA2	-0.336776602	1.18E-15
4190	CPSF1	0.59714993	5.29E-53
4191	AGPS	-0.371780208	5.57E-19
4192	AKT1	0.125235282	0.00371552
4193	HOOK1	-0.396023579	1.56E-21
4194	GTPBP2	0.494078865	2.82E-34
4195	ARFGAP1	0.647080475	8.64E-65
4196	NUP98	-0.241803173	1.48E-08
4197	LONP2	-0.461897397	1.25E-29
4198	ARFIP1	-0.574708207	2.38E-48
4199	DENND1C	-0.022173088	0.608841142
4200	KIAA1462	-0.253467113	2.74E-09
4201	TBRG4	0.555998999	9.77E-45
4202	RITA1	0.237077464	2.85E-08
4203	KAT5	-0.280266153	4.11E-11
4204	MAPK14	-0.340484991	5.48E-16
4205	GIPC2	-0.179874051	2.85E-05
4206	SULF1	-0.113275057	0.00873189
4207	RNF186	0.020594091	0.634588154
4208	PTPN6	0.470672328	7.56E-31
4209	DPP9	0.577718825	5.93E-49
4210	MTHFD1	-0.106949369	0.013321287
4211	TAOK2	0.306647514	4.12E-13
4212	DNAJB11	0.398607886	8.07E-22
4213	CHURC1	-0.444722897	2.41E-27
4214	FAM13A	-0.295454112	3.08E-12
4215	NDUFAF1	-0.101948945	0.018338137
4216	C2orf47	-0.225970004	1.27E-07
4217	PAK1	-0.099181189	0.021769753
4218	PRADC1	0.373911299	3.39E-19
4219	FZD6	-0.328740841	6.00E-15
4220	CPPED1	-0.455583672	8.95E-29
4221	IL2RG	0.365088938	2.60E-18
4222	MRPS14	-0.288189727	1.08E-11
4223	PLA2G15	0.048981303	0.25806779
4224	PIK3AP1	-0.123243441	0.004305686
4225	TRMT10C	-0.206507052	1.45E-06
4226	AAMDC	0.335999583	1.38E-15
4227	PHAX	-0.362202295	4.98E-18
4228	C17orf89	0.559864441	1.83E-45
4229	MYH10	-0.290968144	6.72E-12
4230	TNFSF13	-0.204859755	1.77E-06
4231	SPOP	-0.060357675	0.163292568
4232	CDC27	-0.367256911	1.58E-18

4233	SNX27	-0.259320371	1.14E-09
4234	CFLAR	0.091342397	0.034666609
4235	ASPH	-0.362784324	4.37E-18
4236	DDB2	0.26822879	2.87E-10
4237	RHPN2	-0.288043878	1.11E-11
4238	VWA9	-0.244101175	1.07E-08
4239	TOR3A	0.414426941	1.28E-23
4240	RMDN3	-0.117195776	0.006652371
4241	OSBPL1A	-0.539965274	8.12E-42
4242	BLMH	0.124988153	0.003784526
4243	CERS4	-0.011965808	0.782446313
4244	MLST8	0.404785715	1.64E-22
4245	EML3	0.486481016	3.91E-33
4246	TIAL1	0.029087544	0.501987872
4247	TRIP4	-0.209978789	9.57E-07
4248	MESDC2	-0.284523607	2.02E-11
4249	EVC	-0.334451135	1.90E-15
4250	FTSJ3	0.251387026	3.73E-09
4251	PIP5K1A	0.16230408	0.000163018
4252	HLA-DQB2	0.086440235	0.045669656
4253	NOP14	-0.061852354	0.153098813
4254	TBC1D9	-0.520645877	1.68E-38
4255	C1QTNF1	0.224640524	1.51E-07
4256	FBLIM1	0.140110594	0.001157185
4257	MAD2L1BP	0.031742954	0.463751194
4258	MRPL23	0.515843136	1.04E-37
4259	PURB	-0.290487992	7.30E-12
4260	SERTAD2	-0.140061091	0.00116191
4261	FAM149A	0.002489524	0.954188127
4262	PRKRA	-0.101016037	0.01943797
4263	FYTTD1	-0.389044599	8.90E-21
4264	INPP5K	0.218377905	3.38E-07
4265	C9orf69	0.439278402	1.20E-26
4266	BARX2	0.032416901	0.454310591
4267	EPHB4	-0.034488279	0.425981342
4268	PFKM	-0.384067656	3.01E-20
4269	PLXDC2	-0.249866992	4.65E-09
4270	MED24	0.412508245	2.14E-23
4271	MMS19	0.143991189	0.000837308
4272	PEX6	0.23752233	2.68E-08
4273	ATP6V0A1	-0.00038143	0.992977227
4274	USP39	0.345290591	2.00E-16
4275	SLC41A1	-0.13283704	0.002076977
4276	QRICH1	-0.065407506	0.13079871
4277	RNF113A	0.411722312	2.64E-23
4278	RABEP1	-0.326375024	9.60E-15
4279	WAPAL	-0.412172877	2.34E-23
4280	TESK1	0.553800216	2.51E-44
4281	AGBL5	-0.011163405	0.79670453
4282	SCAF11	-0.432141228	9.43E-26
4283	FCHSD2	-0.155686303	0.000300724
4284	ACOX1	-0.29339691	4.41E-12
4285	BCL6B	-0.09958287	0.021239502
4286	ABCE1	-0.288151558	1.09E-11
4287	MTSS1	-0.378099637	1.26E-19
4288	DPYSL3	0.095949979	0.026468977
4289	IDI1	-0.376803464	1.72E-19
4290	ARHGEF1	0.673429939	5.96E-72

4291	PCYT1A	-0.109334214	0.011387314
4292	MINPP1	-0.247139336	6.91E-09
4293	SLC7A5	0.176708126	3.95E-05
4294	RFNG	0.663503721	3.62E-69
4295	DESI1	0.425561638	6.05E-25
4296	RFC1	-0.484340857	8.11E-33
4297	DKC1	0.195249755	5.38E-06
4298	CCDC23	0.214702417	5.36E-07
4299	MGAT4A	-0.378729604	1.09E-19
4300	BTBD3	-0.350731736	6.24E-17
4301	SBNO2	0.543690033	1.76E-42
4302	ZFAND2B	0.557482381	5.15E-45
4303	STK39	-0.155181467	0.000314795
4304	RPS6KA3	-0.41884443	3.86E-24
4305	SGSM3	0.532769294	1.48E-40
4306	PPP3CB	-0.372516606	4.70E-19
4307	PLEKHF2	-0.503972484	8.32E-36
4308	COX16	-0.123142467	0.004337746
4309	DECR2	0.466514428	2.88E-30
4310	C1orf123	0.440790014	7.70E-27
4311	VPS41	-0.51279217	3.26E-37
4312	STXBP3	-0.359070344	1.00E-17
4313	C3AR1	-0.054510388	0.208096817
4314	TK1	0.522407569	8.54E-39
4315	IRF2BP1	0.272503265	1.46E-10
4316	CHIC2	0.06069851	0.160924296
4317	TRABD2B	-0.035924733	0.406954106
4318	TMEM150A	0.528462723	8.11E-40
4319	CDK10	0.527899906	1.01E-39
4320	ZBTB38	-0.355878331	2.03E-17
4321	DEAF1	0.409741525	4.47E-23
4322	ADSL	0.156479347	0.0002798
4323	KIDINS220	-0.450156984	4.71E-28
4324	ARL16	0.466093079	3.30E-30
4325	SMAD3	0.027629094	0.523675632
4326	NCKAP1	-0.485655511	5.19E-33
4327	CCDC97	0.278973854	5.08E-11
4328	AGA	-0.172278357	6.19E-05
4329	ATL2	-0.322741452	1.96E-14
4330	SMARCA4	0.232040626	5.67E-08
4331	WIBG	0.58622193	1.08E-50
4332	ANAPC11	0.571713104	9.34E-48
4333	FAM219A	0.264432745	5.20E-10
4334	YDJC	0.608734049	1.49E-55
4335	METRNL	0.154833392	0.000324853
4336	MAML2	-0.341657997	4.29E-16
4337	PCDH12	-0.130353066	0.002520062
4338	GIGYF1	0.44288001	4.16E-27
4339	ALG8	-0.129017339	0.002792479
4340	ACTL6A	0.015891063	0.713824312
4341	ST3GAL1	-0.25088545	4.01E-09
4342	PLSCR4	-0.285162837	1.81E-11
4343	TMEM133	-0.187860106	1.22E-05
4344	RNF130	-0.058805363	0.174410976
4345	SLAIN2	-0.553442046	2.92E-44
4346	KPNA3	-0.307884644	3.28E-13
4347	ZDHHC20	-0.304407885	6.20E-13
4348	PIP5K1C	0.231934926	5.75E-08

4349	TUSC2	0.162438825	0.000160958
4350	JADE1	-0.282801267	2.69E-11
4351	TSPAN14	-0.135197474	0.001723152
4352	TMEM165	0.145198704	0.000755872
4353	C11orf24	0.567445536	6.40E-47
4354	AOC3	-0.137915297	0.001384695
4355	CDA	0.038084215	0.379320473
4356	CEBPG	0.057987463	0.180491296
4357	WDR43	-0.134996042	0.001751035
4358	TRAPPC4	0.254597732	2.32E-09
4359	IRF2BPL	-0.082798696	0.055626883
4360	PTPRB	-0.339748943	6.39E-16
4361	RFX5	0.083665194	0.053107323
4362	PAPD4	-0.188983378	1.08E-05
4363	KCNJ8	0.039372751	0.363394068
4364	INSIG1	-0.33968508	6.47E-16
4365	MYO1E	-0.255131954	2.14E-09
4366	SLC6A12	0.048881075	0.259044322
4367	SEC24C	-0.314297894	9.91E-14
4368	AOX1	-0.09797617	0.023429972
4369	CLK2	0.578931952	3.37E-49
4370	PLGRKT	-0.000690428	0.987288413
4371	RPF2	-0.011906428	0.783499038
4372	UBE3C	-0.422232436	1.52E-24
4373	SRCAP	0.146674754	0.000666298
4374	FAM35A	-0.284568409	2.00E-11
4375	CCBL2	-0.325108064	1.23E-14
4376	XPNPEP1	0.047436576	0.273402093
4377	SASH1	-0.418950839	3.75E-24
4378	ELOF1	0.623530618	5.78E-59
4379	KPNA4	-0.2692548	2.44E-10
4380	C1orf54	0.262977469	6.51E-10
4381	ZBTB18	-0.096943369	0.02493904
4382	PLA2G12A	-0.562757949	5.14E-46
4383	LRRC42	-0.026260017	0.544461764
4384	APBB1	-0.179462219	2.98E-05
4385	GTF3C2	0.140126881	0.001155634
4386	AKAP17A	0.538803624	1.30E-41
4387	KRBA1	0.20099614	2.78E-06
4388	DUS1L	0.726239198	8.38E-89
4389	DNAJC22	-0.047151961	0.276293842
4390	HMGCS1	-0.413801773	1.51E-23
4391	RORC	-0.200872872	2.82E-06
4392	PIK3R3	-0.272355037	1.49E-10
4393	CWC22	-0.150641503	0.00047199
4394	ERMP1	-0.566779425	8.63E-47
4395	PHF3	-0.437312673	2.13E-26
4396	FPGS	0.560199394	1.58E-45
4397	GPD1	0.219411802	2.96E-07
4398	RNPS1	0.543875549	1.63E-42
4399	SLC46A3	-0.288749251	9.85E-12
4400	HDAC5	0.09984563	0.020898739
4401	SMC1A	-0.248588634	5.60E-09
4402	EIF4E2	0.311569574	1.65E-13
4403	ZNF524	0.543161092	2.19E-42
4404	NRBF2	-0.159453827	0.000212837
4405	B3GNT9	0.062517256	0.148722395
4406	CHD1L	0.142368506	0.000959549

4407	UBA3	-0.267533029	3.20E-10
4408	ZDHHC16	0.370913661	6.82E-19
4409	SLC25A43	0.086547485	0.045400727
4410	ARHGAP5	-0.556728627	7.13E-45
4411	TCF12	-0.320894098	2.80E-14
4412	FAM57A	0.031430962	0.468158175
4413	FXR1	-0.158096333	0.000241282
4414	PIK3CB	-0.454922981	1.10E-28
4415	TSSC4	0.594523831	1.93E-52
4416	UBE2I	0.347018194	1.38E-16
4417	PAQR7	0.021930654	0.612764048
4418	PEMT	0.341469841	4.46E-16
4419	UNC93B1	0.343300807	3.04E-16
4420	ERCC1	0.531169526	2.79E-40
4421	ECHDC1	-0.357885626	1.31E-17
4422	PPIG	-0.245955139	8.19E-09
4423	RPRD2	-0.31504717	8.60E-14
4424	ARHGAP24	-0.401025879	4.35E-22
4425	KLHL24	-0.497156882	9.55E-35
4426	UBE2G1	-0.408900214	5.58E-23
4427	NUDT16	-0.125618834	0.003610677
4428	SH2D3C	0.176359649	4.10E-05
4429	CUX1	-0.116037926	0.007214689
4430	YY1AP1	0.177350361	3.70E-05
4431	B4GALT2	0.417595796	5.43E-24
4432	TBL2	0.035887706	0.407438131
4433	TBCE	0.202597786	2.31E-06
4434	RAP2B	-0.122367178	0.004591187
4435	NKIRAS2	0.428217283	2.87E-25
4436	ABCA1	-0.198366878	3.77E-06
4437	NRM	0.442389893	4.81E-27
4438	PIGK	-0.538444295	1.51E-41
4439	FNBP1	0.071998665	0.096192725
4440	PTGFRN	-0.138865192	0.001281625
4441	SLC43A2	-0.047963324	0.268104818
4442	HEXA	0.089947235	0.037540509
4443	FBXO8	-0.525825835	2.27E-39
4444	KLHDC7A	-0.184153239	1.82E-05
4445	FN3K	0.139907259	0.00117671
4446	WIPI2	0.042556894	0.325860176
4447	KCNK5	0.013491107	0.755546336
4448	MARS	0.40929308	5.03E-23
4449	BPNT1	-0.267361957	3.29E-10
4450	DHX16	0.311036771	1.83E-13
4451	TPRKB	0.201232528	2.71E-06
4452	USP19	-0.065172934	0.132188034
4453	CDC25B	0.504922878	5.90E-36
4454	RBBP8	-0.245910242	8.25E-09
4455	AGPAT6	0.135041677	0.001744682
4456	QKI	-0.448159421	8.60E-28
4457	KAT8	0.479846703	3.68E-32
4458	GNE	-0.380343351	7.39E-20
4459	MAP7	-0.287929983	1.13E-11
4460	TFPI2	0.097628544	0.023928833
4461	CCND2	0.058471622	0.176873351
4462	TRIM14	0.082415336	0.05677287
4463	FAM3A	0.487654925	2.62E-33
4464	CMIP	-0.052771673	0.222993545

4465	KIAA0020	0.12270806	0.00447815
4466	DDX27	0.457406438	5.09E-29
4467	SRRM1	0.018922221	0.662337887
4468	FTO	-0.308596531	2.88E-13
4469	GTF2A2	0.055637992	0.198828675
4470	BLCAP	0.212185515	7.31E-07
4471	MED15	0.611582877	3.40E-56
4472	SDHAF1	0.408261511	6.61E-23
4473	EIF4G3	-0.161655631	0.000173282
4474	SUPT7L	0.28929083	8.97E-12
4475	CCDC22	0.447616938	1.01E-27
4476	SNAP23	-0.359366901	9.40E-18
4477	HAUS4	-0.032429327	0.454137543
4478	LRRC75B	0.388195431	1.10E-20
4479	TRABD	0.731628939	9.54E-91
4480	ANKRD17	-0.458465776	3.66E-29
4481	PRPF40A	-0.294415217	3.69E-12
4482	PQLC1	0.364962988	2.67E-18
4483	PRPF38B	0.16470796	0.000129734
4484	EXOSC10	0.251517731	3.65E-09
4485	LTBP1	-0.029178064	0.500657593
4486	C1orf162	0.293483591	4.34E-12
4487	FAM102A	0.056875322	0.189008984
4488	LATS2	-0.207514001	1.29E-06
4489	FBXL16	0.00932416	0.829635806
4490	GPX8	0.043689674	0.313136218
4491	ASXL1	0.098551635	0.022623928
4492	C7orf26	0.423984924	9.38E-25
4493	TOM1L2	-0.069506879	0.108300047
4494	CCAR1	0.226599703	1.17E-07
4495	SLC5A3	-0.192676373	7.18E-06
4496	CAND1	-0.517557063	5.44E-38
4497	TMEM69	0.104366881	0.015736725
4498	SPRED1	-0.327284445	8.02E-15
4499	DDX58	-0.150206016	0.000490401
4500	PIGU	0.223492201	1.76E-07
4501	NPEPPS	-0.380568789	7.00E-20
4502	CD2	0.26625242	3.92E-10
4503	NIF3L1	-0.04000055	0.355787866
4504	MBD1	0.029091955	0.501923004
4505	SBF1	0.35970037	8.73E-18
4506	ARL3	-0.220808297	2.48E-07
4507	SLC35A2	0.188842293	1.10E-05
4508	ATP13A2	0.193337049	6.67E-06
4509	CAMTA2	0.356420599	1.81E-17
4510	RCN3	0.469258932	1.19E-30
4511	CFAP97	-0.51959545	2.51E-38
4512	MAP1LC3A	0.301257444	1.10E-12
4513	SWI5	0.346526655	1.54E-16
4514	FYN	0.022950175	0.596342661
4515	GRAMD3	-0.264347686	5.27E-10
4516	UCKL1	0.462522404	1.03E-29
4517	TMEM251	-0.035393672	0.41392889
4518	INTS5	-0.030848415	0.476448393
4519	AAAS	0.654707648	8.66E-67
4520	SLC31A1	-0.431511765	1.13E-25
4521	FBXO28	-0.439640983	1.08E-26
4522	PML	0.389826369	7.34E-21

4523	MTMR6	-0.424600501	7.90E-25
4524	HOMER3	0.533827762	9.68E-41
4525	SKA2	-0.001561748	0.971251371
4526	FERMT2	-0.206147813	1.52E-06
4527	ZDHHC12	0.531285346	2.66E-40
4528	SCAP	0.114323386	0.008125607
4529	C20orf24	0.256249496	1.81E-09
4530	TMEM14B	-0.105076787	0.015037163
4531	HDAC6	0.248275578	5.86E-09
4532	AKT1S1	0.569189521	2.93E-47
4533	CPSF3	0.117895833	0.006331757
4534	LSM1	0.208625287	1.13E-06
4535	TMOD3	-0.411546879	2.77E-23
4536	HPS3	-0.120449914	0.005276323
4537	HOXB2	0.054372613	0.209250302
4538	ELF3	0.082703825	0.055908676
4539	AP1AR	-0.3637792	3.49E-18
4540	HARS	0.319280978	3.83E-14
4541	LIN7C	-0.46740614	2.17E-30
4542	MYCT1	-0.262236461	7.30E-10
4543	GJA5	-0.172576849	6.01E-05
4544	SMAD5	-0.495279189	1.85E-34
4545	APPL2	-0.218097489	3.50E-07
4546	MTR	-0.249900792	4.63E-09
4547	NCOR1	-0.269064138	2.52E-10
4548	RAP2C	-0.150376186	0.000483129
4549	MVB12A	0.515371268	1.24E-37
4550	CDKN1C	-0.042843555	0.322609005
4551	TBC1D10B	0.520134852	2.04E-38
4552	YIPF6	-0.599810014	1.40E-53
4553	FAS	-0.113918982	0.008354887
4554	C19orf48	0.407806291	7.45E-23
4555	PPP1R12C	0.551829289	5.81E-44
4556	ACADSB	-0.617818635	1.26E-57
4557	FTSJ1	0.312838783	1.30E-13
4558	PLEKHA4	0.057199666	0.186494926
4559	C14orf142	-0.019353035	0.655140915
4560	PKN2	-0.404992636	1.56E-22
4561	C1orf27	-0.072419503	0.094258858
4562	PPP1R35	0.778388799	7.57E-110
4563	BAG1	-0.162322939	0.000162728
4564	NFKBIB	0.625028064	2.55E-59
4565	JAM3	-0.125135737	0.003743178
4566	DSP	-0.252150048	3.33E-09
4567	SLC35A5	-0.459435759	2.71E-29
4568	C8orf59	0.400358459	5.16E-22
4569	PHRF1	0.368220885	1.27E-18
4570	CFD	0.275847077	8.48E-11
4571	FXR2	0.185398535	1.59E-05
4572	TXNDC9	-0.231003674	6.52E-08
4573	LDOC1L	-0.404177033	1.92E-22
4574	KIFC3	0.240348181	1.81E-08
4575	SELO	0.629081251	2.72E-60
4576	TBC1D4	-0.381155301	6.08E-20
4577	RPS6KA1	0.1984073	3.76E-06
4578	COPZ2	0.194763357	5.68E-06
4579	PPP3CA	-0.477232149	8.80E-32
4580	HSF4	0.483359458	1.13E-32

4581	WWP2	-0.215263991	5.00E-07
4582	SNAP29	-0.234599541	4.01E-08
4583	SRC	0.454379143	1.30E-28
4584	PELP1	0.497583747	8.21E-35
4585	ATXN1L	-0.428818301	2.42E-25
4586	LYSMD3	-0.539261107	1.08E-41
4587	FUZ	0.241709396	1.50E-08
4588	EXOC4	-0.344716801	2.26E-16
4589	FBXO18	-0.050170764	0.246672886
4590	PRR14	0.672146514	1.38E-71
4591	ZBTB44	-0.566409624	1.02E-46
4592	ERLIN1	-0.280020906	4.28E-11
4593	PPP1R13L	0.353789276	3.22E-17
4594	TTC19	-0.313900486	1.07E-13
4595	CDC23	-0.279201989	4.90E-11
4596	C1orf122	0.424455767	8.23E-25
4597	TMEM184C	-0.608410566	1.77E-55
4598	RXRA	-0.028107058	0.516515679
4599	SKIL	-0.102083723	0.01818382
4600	CCNDBP1	-0.444099784	2.90E-27
4601	PRAP1	0.218311292	3.41E-07
4602	NEURL1B	-0.169010943	8.55E-05
4603	TIA1	0.334798289	1.77E-15
4604	ECD	-0.169965798	7.79E-05
4605	PNPLA6	0.401544737	3.80E-22
4606	PRPF4B	-0.127138007	0.003221259
4607	NIPA2	-0.388038128	1.14E-20
4608	MLLT4	-0.307467186	3.54E-13
4609	MGME1	0.167672163	9.75E-05
4610	RAD50	-0.42105373	2.11E-24
4611	BTN3A3	0.005038396	0.907441259
4612	APOL3	0.086715837	0.044981274
4613	COL8A1	-0.137003567	0.00149074
4614	ELL2	-0.096548495	0.025537797
4615	RHNO1	0.261917218	7.67E-10
4616	SLCO2B1	-0.119909597	0.005485374
4617	ARFIP2	0.476857053	9.96E-32
4618	SP3	-0.340043441	6.01E-16
4619	TNRC18	0.135749219	0.00164885
4620	ORMDL2	0.335089527	1.67E-15
4621	SP2	-0.192909468	6.99E-06
4622	NOSIP	0.526224134	1.95E-39
4623	DAPK1	-0.073372886	0.089992724
4624	ZKSCAN1	-0.308083273	3.16E-13
4625	IGF1R	-0.392833279	3.47E-21
4626	NRBP2	0.429671543	1.90E-25
4627	TMEM161A	0.452232053	2.50E-28
4628	CPSF6	-0.070702821	0.102346909
4629	MTRR	-0.15549113	0.000306093
4630	EXOC1	-0.172482259	6.06E-05
4631	LPXN	0.237870339	2.56E-08
4632	MT1F	0.140500755	0.001120553
4633	PFDN6	0.616560549	2.47E-57
4634	NCOA1	-0.462275465	1.11E-29
4635	EPN1	0.480711509	2.76E-32
4636	NRIP1	-0.416565582	7.18E-24
4637	MYO9B	0.36759122	1.47E-18
4638	CPEB4	-0.51093225	6.51E-37

4639	RANBP2	-0.48066342	2.80E-32
4640	ARFGEF2	-0.494362968	2.56E-34
4641	BNIP2	-0.300454764	1.27E-12
4642	PRRG4	-0.354197264	2.94E-17
4643	DHX29	-0.381315137	5.85E-20
4644	ECSCR	0.013111215	0.762219888
4645	PHACTR4	0.014145451	0.744094278
4646	ACSL3	-0.406191531	1.14E-22
4647	DDX39B	0.362047197	5.16E-18
4648	ANAPC5	0.328478083	6.32E-15
4649	SDCCAG3	0.571323247	1.11E-47
4650	ACSS1	0.012973787	0.764638468
4651	FAM171A1	-0.058732227	0.174948389
4652	AAGAB	-0.159229868	0.000217303
4653	ATP6V0E2	0.121879914	0.004757241
4654	OBSL1	0.143610952	0.000864586
4655	MDM2	-0.187278332	1.30E-05
4656	RAB40C	0.418829131	3.88E-24
4657	ATG9A	0.322902676	1.90E-14
4658	CACFD1	0.18792139	1.21E-05
4659	KIAA1217	-0.296024807	2.78E-12
4660	ORAI1	0.29925036	1.57E-12
4661	SAP30L	-0.414408004	1.29E-23
4662	SCAMP4	0.239805887	1.96E-08
4663	SDHC	-0.392303545	3.96E-21
4664	SNX29	-0.281683099	3.25E-11
4665	ALG3	0.538887725	1.26E-41
4666	TDRP	-0.148375818	0.000575318
4667	OPA1	-0.464737675	5.08E-30
4668	FAH	0.120383379	0.005301672
4669	TSPYL4	-0.392907865	3.41E-21
4670	ZC3H7A	-0.085416674	0.048304202
4671	CD55	-0.1160451	0.007211078
4672	ORMDL1	0.331800038	3.25E-15
4673	TMCO4	-0.148367062	0.000575755
4674	RTP4	-0.047913831	0.268599539
4675	TMPO	-0.151103405	0.000453166
4676	GGPS1	-0.19378608	6.34E-06
4677	ZCCHC3	0.178066355	3.44E-05
4678	DOLK	0.149032628	0.000543385
4679	STK16	0.18013552	2.78E-05
4680	FCGR2A	-0.017412866	0.687795022
4681	POGZ	0.179468582	2.98E-05
4682	KLHL5	-0.402235859	3.18E-22
4683	TMEM41B	-0.217092969	3.97E-07
4684	SH3BGRL2	-0.403068099	2.57E-22
4685	FDX1	-0.315093639	8.52E-14
4686	MCCC1	-0.250135862	4.47E-09
4687	ABCC4	-0.408923418	5.55E-23
4688	GPC6	-0.424478453	8.18E-25
4689	CUTC	0.175529142	4.46E-05
4690	FAM160A2	0.330484682	4.23E-15
4691	BCL7C	0.501405567	2.10E-35
4692	EFTUD2	0.103140613	0.017012528
4693	MED21	-0.476173747	1.25E-31
4694	GNL2	0.433639502	6.14E-26
4695	GLYATL1	-0.080498909	0.062799193
4696	SKIV2L	0.436176912	2.96E-26

4697	PLCG1	0.287410965	1.24E-11
4698	TSR1	-0.107333289	0.012991621
4699	MPI	0.04208662	0.331239641
4700	EXOSC4	0.546060364	6.58E-43
4701	DYRK1B	0.331204307	3.66E-15
4702	DESI2	-0.22318488	1.83E-07
4703	EHBP1L1	0.343027773	3.22E-16
4704	NPNT	-0.260927257	8.92E-10
4705	RPS6KA2	-0.363623094	3.62E-18
4706	DUSP14	0.300451732	1.27E-12
4707	METTL5	0.218362073	3.39E-07
4708	MRPL52	0.581854569	8.58E-50
4709	CPSF3L	0.594607931	1.86E-52
4710	DCAF5	-0.030351077	0.48358899
4711	CCDC43	-0.242996244	1.25E-08
4712	NFYC	0.197226589	4.30E-06
4713	GPR124	-0.034977406	0.419444984
4714	USP1	-0.404841604	1.62E-22
4715	SPON2	0.177679518	3.58E-05
4716	NOL11	-0.103666495	0.016454719
4717	ABT1	0.234089584	4.30E-08
4718	IRF7	0.623409052	6.18E-59
4719	TCEB3	-0.177320569	3.71E-05
4720	FAM98A	-0.049467725	0.25336469
4721	AGFG1	-0.284345426	2.08E-11
4722	CARD10	0.168048011	9.40E-05
4723	TRIM38	0.07729506	0.074045612
4724	PLEK	0.010840891	0.802454808
4725	MCTS1	0.159084924	0.00022024
4726	SULT1C4	-0.240793564	1.70E-08
4727	KDELR3	0.225846896	1.29E-07
4728	SLIRP	0.40799233	7.10E-23
4729	SLC25A36	-0.200634117	2.90E-06
4730	CD82	0.016857171	0.697259021
4731	DHRSX	0.03925947	0.364777268
4732	B3GALNT1	-0.276123736	8.11E-11
4733	PHF2	-0.011169061	0.79660378
4734	MZT1	-0.240969643	1.66E-08
4735	ARMC10	-0.197109774	4.36E-06
4736	SAMM50	-0.211357909	8.09E-07
4737	NIT2	0.309183019	2.58E-13
4738	AASDHPPT	-0.481148232	2.38E-32
4739	RNASEH2C	0.549839845	1.35E-43
4740	ZSWIM8	0.318269334	4.65E-14
4741	MAFK	0.192442287	7.37E-06
4742	BAIAP2L2	0.426428909	4.74E-25
4743	SLC35B1	0.403159611	2.51E-22
4744	SLC22A18	0.373298222	3.91E-19
4745	SEMA6A	0.020360934	0.638428644
4746	PDCD10	-0.320081466	3.28E-14
4747	EIF2B5	0.378751851	1.08E-19
4748	CKAP5	-0.178452902	3.31E-05
4749	ASUN	-0.083216484	0.054399929
4750	ELF4	0.080749588	0.061982126
4751	TFPT	0.558099427	3.94E-45
4752	CHPT1	-0.249114268	5.19E-09
4753	NOTCH2	-0.299844131	1.41E-12
4754	EXOC7	-0.137549364	0.001426395

4755	RMDN1	-0.25066862	4.14E-09
4756	BIVM	0.014541755	0.737185493
4757	ABCB7	-0.494411408	2.51E-34
4758	AMN	0.225178838	1.41E-07
4759	HS6ST1	-0.066460557	0.124700468
4760	AGGF1	-0.489750125	1.27E-33
4761	CTSK	0.074588613	0.08477901
4762	PEX2	-0.367609792	1.46E-18
4763	DNAJA3	-0.001898175	0.965062018
4764	SETX	-0.35792832	1.29E-17
4765	NASP	0.433355462	6.66E-26
4766	TPRA1	0.455887959	8.15E-29
4767	PCM1	-0.377936815	1.31E-19
4768	SNRPF	0.593734633	2.85E-52
4769	ETFDH	-0.342723061	3.43E-16
4770	ALCAM	-0.273299093	1.28E-10
4771	NEK9	-0.152767589	0.000390985
4772	BACE2	-0.140020821	0.001165768
4773	CELSR1	-0.032081841	0.458990521
4774	IMPACT	-0.49454967	2.39E-34
4775	UBQLN4	0.137069194	0.001482864
4776	GALC	-0.386820347	1.54E-20
4777	PIGQ	0.42113923	2.06E-24
4778	RPS6KA4	0.519016373	3.13E-38
4779	PTGS1	-0.064165803	0.138282922
4780	FUOM	0.256829515	1.66E-09
4781	VPS54	-0.330328024	4.37E-15
4782	PHLDB2	-0.33113766	3.71E-15
4783	LIMCH1	-0.518089491	4.45E-38
4784	ALOX5	0.110838984	0.010298974
4785	LYRM5	-0.381637943	5.41E-20
4786	C17orf62	0.577811451	5.68E-49
4787	XYLT2	0.311701512	1.61E-13
4788	KIAA0930	0.395351096	1.84E-21
4789	KLC4	0.124687023	0.00387018
4790	ADSSL1	0.058111322	0.179560569
4791	HTRA2	0.282858345	2.67E-11
4792	NUCB2	-0.102948736	0.017220136
4793	FGD5	-0.263771951	5.76E-10
4794	DUSP16	-0.347757432	1.18E-16
4795	GOPC	-0.283108026	2.56E-11
4796	NPM3	0.419161393	3.54E-24
4797	RNF6	-0.340351283	5.64E-16
4798	KIAA1551	-0.109936287	0.010940201
4799	ABHD6	-0.174922292	4.74E-05
4800	MAP3K3	-0.069589885	0.107878228
4801	NUS1	-0.508273738	1.73E-36
4802	KDM4A	-0.17220116	6.24E-05
4803	FHL3	0.339602362	6.59E-16
4804	TMX3	-0.177775522	3.54E-05
4805	SCGN	0.044895066	0.299959739
4806	ZNF638	-0.085511997	0.048053591
4807	SYK	-0.139763625	0.001190685
4808	ACSL5	-0.142808372	0.000924879
4809	LRRC58	-0.492514779	4.88E-34
4810	PDLIM7	0.540433388	6.70E-42
4811	LAGE3	0.458292728	3.86E-29
4812	IBTK	-0.450260084	4.56E-28

4813	DNAJC19	-0.027613192	0.523914707
4814	C14orf159	-0.0970612	0.024762734
4815	GTF2E2	0.327242838	8.08E-15
4816	ABCA3	0.116665443	0.006904863
4817	CTNNAL1	-0.263049016	6.44E-10
4818	UXS1	-0.238396363	2.38E-08
4819	FABP6	0.316224592	6.87E-14
4820	CXorf36	-0.100570803	0.019982829
4821	CNOT7	-0.452268715	2.48E-28
4822	RDH10	0.025785353	0.551762664
4823	BCL10	-0.346393392	1.58E-16
4824	ETNK1	-0.28021537	4.14E-11
4825	PPP4R1	-0.158538119	0.000231656
4826	SLFN11	0.018208362	0.67433177
4827	ZBTB7A	0.13453487	0.001816437
4828	HNF4A	-0.097759675	0.023739584
4829	STAM	-0.470529166	7.92E-31
4830	SUDS3	-0.158911872	0.000223794
4831	C4A	-0.139734661	0.001193522
4832	INADL	-0.300413198	1.28E-12
4833	SUOX	-0.018267779	0.673330269
4834	CCDC106	0.33199464	3.12E-15
4835	PARD6B	-0.29434883	3.73E-12
4836	APEX2	0.363607014	3.63E-18
4837	IFT22	0.041720157	0.335471079
4838	ARHGEF10L	0.168855504	8.68E-05
4839	COG1	0.261586625	8.06E-10
4840	BOLA1	0.372648825	4.55E-19
4841	P3H4	0.465139781	4.47E-30
4842	RPIA	0.331172448	3.69E-15
4843	KIAA0232	-0.459406013	2.73E-29
4844	5-Mar	-0.443323082	3.65E-27
4845	MRPS31	-0.0123558	0.775542312
4846	PTPN23	0.151541502	0.00043596
4847	TARS2	0.30042887	1.27E-12
4848	TEK	-0.362351211	4.82E-18
4849	FIBIN	-0.063749593	0.140863879
4850	BRPF3	-0.321342887	2.57E-14
4851	PSEN1	-0.373319858	3.89E-19
4852	TMEM64	-0.316285695	6.80E-14
4853	SMIM7	0.09412937	0.02948343
4854	AKT3	-0.335193317	1.63E-15
4855	TMEM135	-0.490060396	1.14E-33
4856	ARSB	-0.185522536	1.57E-05
4857	NOMO1	-0.028518008	0.510400316
4858	RASA1	-0.357230055	1.51E-17
4859	PIM2	0.273936503	1.16E-10
4860	OXLD1	0.713133383	2.89E-84
4861	TRA2B	-0.114318445	0.008128374
4862	HSCB	0.294334927	3.74E-12
4863	GPC1	0.312179315	1.48E-13
4864	UBXN2B	-0.452255165	2.49E-28
4865	FERMT3	0.371147314	6.46E-19
4866	SLC27A4	0.260986217	8.84E-10
4867	CCDC71	0.08440557	0.051030501
4868	CCDC58	0.299284428	1.56E-12
4869	IPO13	0.236419427	3.13E-08
4870	CLDN10	-0.161525991	0.000175405

4871	CAMSAP2	-0.402683771	2.83E-22
4872	TCEAL3	0.074457268	0.085330276
4873	SOS2	-0.50646773	3.36E-36
4874	CSGALNACT2	-0.102192897	0.018059652
4875	CCNC	-0.533693481	1.02E-40
4876	PTPRG	-0.490250897	1.07E-33
4877	POLR1E	0.005700712	0.895339831
4878	PPP4R2	-0.361267593	6.15E-18
4879	FCN3	0.041414986	0.339021164
4880	MGMT	0.268309058	2.84E-10
4881	TAF12	0.303833008	6.88E-13
4882	STARD8	-0.245454705	8.80E-09
4883	LSM3	-0.103530997	0.01659689
4884	ASPA	0.088333653	0.041114026
4885	FAM50B	-0.116537944	0.006966831
4886	ZMYM4	-0.363117946	4.06E-18
4887	EPB41L2	-0.241982241	1.44E-08
4888	IP6K2	0.398232993	8.88E-22
4889	MOV10	0.569207032	2.90E-47
4890	KLHL36	-0.217521369	3.76E-07
4891	BAMBI	-0.057945848	0.180804813
4892	C18orf8	0.226267086	1.22E-07
4893	HCFC1	-0.036414023	0.400590231
4894	NMRK1	0.041864034	0.333805656
4895	RNF25	0.676342611	8.66E-73
4896	ZNF83	0.37409307	3.25E-19
4897	XIAP	-0.37348421	3.75E-19
4898	CDC5L	-0.395591643	1.74E-21
4899	STARD3	0.572030289	8.09E-48
4900	ACSM5	-0.097529854	0.024072121
4901	TMEM33	-0.442988363	4.03E-27
4902	PAPLN	0.118035684	0.006269396
4903	AFAP1L1	-0.192973271	6.95E-06
4904	HDDC2	-0.027905354	0.519531052
4905	FBXO44	0.216761228	4.14E-07
4906	STARD10	0.177519229	3.64E-05
4907	GPN3	-0.442252156	5.01E-27
4908	MMP15	0.072763501	0.092701289
4909	TAF6	0.137234464	0.0014632
4910	CASP3	0.033430544	0.440317116
4911	TRMT1	0.762111068	1.07E-102
4912	GMCL1	-0.220869566	2.46E-07
4913	BTG3	-0.058726537	0.174990249
4914	KDM6B	0.142017481	0.000988074
4915	ATF7IP	-0.360790724	6.84E-18
4916	RRS1	0.268804393	2.62E-10
4917	RB1CC1	-0.409294446	5.03E-23
4918	PRPF38A	-0.050792054	0.240862842
4919	PATZ1	-0.061553606	0.155096703
4920	MON1B	-0.189997653	9.66E-06
4921	TSFM	0.15353881	0.000364951
4922	NR2C2AP	0.621252471	1.99E-58
4923	RELB	0.529692018	5.00E-40
4924	LIFR	-0.454061999	1.43E-28
4925	KLF4	-0.136299472	0.001577686
4926	POGK	-0.10488166	0.015226671
4927	IL17RB	-0.014750982	0.73354652
4928	RHOU	-0.30825905	3.06E-13

4929	MSL1	0.243742914	1.12E-08
4930	ASH2L	-0.302428728	8.88E-13
4931	RAD17	-0.382124631	4.81E-20
4932	VPS26B	0.123175148	0.004327346
4933	MRPL50	-0.249938447	4.61E-09
4934	MIEF1	-0.416811108	6.72E-24
4935	SPATA18	-0.306443991	4.28E-13
4936	SORL1	-0.53734637	2.35E-41
4937	RANBP1	0.423059662	1.21E-24
4938	XPR1	-0.357627078	1.38E-17
4939	MCM3AP	-0.103463443	0.016668171
4940	MKRN2	-0.284623215	1.98E-11
4941	RYBP	-0.427840982	3.19E-25
4942	CTBP2	-0.297091749	2.30E-12
4943	PREP	-0.18904715	1.07E-05
4944	SPIRE1	-0.418943458	3.76E-24
4945	C6orf223	-0.06883064	0.111785237
4946	CD109	-0.208079513	1.20E-06
4947	LIMK2	0.009187737	0.832091126
4948	GNL1	0.08147281	0.05967388
4949	JADE2	-0.21571449	4.72E-07
4950	DAZAP1	0.551416848	6.92E-44
4951	SVIL	-0.166436945	0.000109866
4952	PDCL	-0.34895346	9.16E-17
4953	DPP3	0.180258216	2.74E-05
4954	AJUBA	-0.056927105	0.188605943
4955	AFMID	0.284007967	2.20E-11
4956	CLASP1	-0.214039007	5.82E-07
4957	IL18	0.03310776	0.444746228
4958	MSRA	0.078318651	0.070286538
4959	SLPI	0.077379354	0.07372998
4960	BUB3	0.075997138	0.079046084
4961	TUSC3	-0.065946717	0.127647919
4962	PNO1	-0.051746062	0.232129612
4963	SLC25A42	0.054408429	0.208949995
4964	WDR81	0.099482585	0.021370825
4965	TSPAN17	0.233394921	4.72E-08
4966	SIVA1	0.537059393	2.64E-41
4967	UBTD1	0.400908156	4.48E-22
4968	EIF2D	0.228284904	9.37E-08
4969	TMEM98	-0.040280154	0.352432754
4970	BCAT2	0.393881944	2.67E-21
4971	HS1BP3	0.20880339	1.10E-06
4972	SYT13	-0.158779429	0.000226551
4973	NR1H3	0.34853958	1.00E-16
4974	PEX3	-0.448846941	6.99E-28
4975	APPL1	-0.587277525	6.53E-51
4976	KCNK3	0.077387337	0.073700146
4977	RP2	-0.379320258	9.43E-20
4978	AKIP1	0.265000835	4.76E-10
4979	PLAT	0.029492672	0.49604868
4980	OAS2	-0.032247617	0.4566717
4981	ARID5A	0.51914447	2.98E-38
4982	LEO1	-0.104215083	0.015889963
4983	CNPY2	0.611004363	4.60E-56
4984	SLC25A46	-0.424189689	8.86E-25
4985	TBC1D22A	0.093443025	0.030693932
4986	C4B	-0.128607431	0.002881304

4987	ULK1	0.253759592	2.63E-09
4988	THNSL2	0.073971069	0.087396093
4989	CLPX	-0.411735417	2.63E-23
4990	NADK2	-0.327055045	8.39E-15
4991	BACH1	-0.360170396	7.86E-18
4992	PIN1	0.357211398	1.52E-17
4993	TAB1	0.320043535	3.31E-14
4994	HPS6	0.100726223	0.01979114
4995	SEC23A	-0.266405064	3.82E-10
4996	PHYKPL	0.318870024	4.14E-14
4997	RHOD	0.325861514	1.06E-14
4998	ACADL	-0.055774607	0.197726551
4999	ZNF207	0.106607595	0.013620944
5000	TMEM223	0.439114495	1.26E-26
5001	TRAF4	0.589837033	1.90E-51
5002	PGM2	-0.499167186	4.68E-35
5003	PDRG1	0.356605811	1.73E-17
5004	KSR1	-0.075929004	0.079315971
5005	OSGIN2	-0.296535632	2.54E-12
5006	HAGH	0.339536856	6.68E-16
5007	FLT4	0.031400595	0.468588352
5008	RNF152	-0.370957393	6.75E-19
5009	USP4	-0.112898462	0.008959336
5010	GPR160	-0.455571421	8.98E-29
5011	NOTCH1	-0.001773432	0.967356733
5012	TMCC3	-0.409250863	5.09E-23
5013	MEN1	0.51352289	2.48E-37
5014	EHBP1	-0.244158024	1.06E-08
5015	NBL1	0.431609214	1.10E-25
5016	SORBS1	-0.259033794	1.19E-09
5017	ARHGEF2	0.196995886	4.41E-06
5018	NOV	-0.087528948	0.043000984
5019	DCK	-0.376313973	1.93E-19
5020	U2SURP	0.051710011	0.232455498
5021	ITPR3	-0.06288568	0.14633878
5022	IFT46	-0.021971724	0.612098702
5023	CYP4V2	-0.124867511	0.003818633
5024	VAPB	-0.486017224	4.58E-33
5025	C1QL1	0.36712909	1.63E-18
5026	OXCT1	-0.298371777	1.84E-12
5027	CABIN1	0.133079996	0.002037709
5028	PAFAH1B3	0.571615497	9.76E-48
5029	AP3M1	-0.386753523	1.56E-20
5030	RAB21	-0.476848705	9.99E-32
5031	GEMIN8	0.228956185	8.57E-08
5032	LY86	0.109209655	0.011481807
5033	FNIP2	-0.427721351	3.30E-25
5034	TMEM181	-0.316923939	6.02E-14
5035	CLCN5	-0.254925517	2.21E-09
5036	SMIM10	-0.074157453	0.086599456
5037	THAP7	0.581571253	9.81E-50
5038	UBE2J2	0.539040688	1.18E-41
5039	C2orf68	0.493390795	3.59E-34
5040	SH3KBP1	0.077312851	0.073978903
5041	IFIH1	-0.271162321	1.80E-10
5042	BZW2	0.044100145	0.308607215
5043	POLRMT	0.640716072	3.64E-63
5044	RAB8A	0.224511484	1.54E-07

5045	LRRC61	0.246997196	7.05E-09
5046	AKAP11	-0.497490926	8.48E-35
5047	SNX18	-0.535848753	4.30E-41
5048	UGGT1	-0.24012016	1.87E-08
5049	FAM60A	-0.401409894	3.94E-22
5050	RNF41	-0.292905643	4.80E-12
5051	MZT2A	0.544739976	1.14E-42
5052	RLIM	-0.480197089	3.28E-32
5053	SLC44A1	-0.400311052	5.22E-22
5054	HS2ST1	-0.459071077	3.03E-29
5055	CXorf56	-0.224630537	1.51E-07
5056	ASAP1	-0.170222738	7.59E-05
5057	FAM122B	-0.049313003	0.254854152
5058	LSG1	0.169290278	8.32E-05
5059	FAM53B	-0.062557427	0.148461076
5060	RASA3	0.16729663	0.000101088
5061	SESN2	0.161672027	0.000173015
5062	LGALS8	-0.092664014	0.032119192
5063	AKR7A3	0.033750708	0.435948974
5064	CRNKL1	-0.483355823	1.13E-32
5065	UBR4	-0.048418528	0.263584035
5066	EZH1	-0.006337845	0.883721373
5067	COX11	-0.336029686	1.38E-15
5068	SSSCA1	0.47295903	3.59E-31
5069	AKAP13	-0.256356342	1.78E-09
5070	LAS1L	0.29332877	4.46E-12
5071	NME1	0.584486276	2.47E-50
5072	GPR137	0.536447068	3.38E-41
5073	SLC7A1	-0.101023669	0.019428743
5074	TCTN2	-0.051792196	0.231713043
5075	YPEL2	-0.233783443	4.48E-08
5076	ODF3B	0.642052428	1.67E-63
5077	LTV1	0.1734955	5.48E-05
5078	RRNAD1	0.484571507	7.50E-33
5079	NSF	-0.247349341	6.70E-09
5080	WFS1	0.071994701	0.09621109
5081	TEAD4	0.505921734	4.10E-36
5082	EIF1AD	0.312555782	1.38E-13
5083	PLXNB1	0.221355176	2.31E-07
5084	ENKD1	0.555270849	1.34E-44
5085	PRKRIP1	0.626418164	1.19E-59
5086	MEF2C	-0.264441556	5.19E-10
5087	PSMD5	-0.247819597	6.26E-09
5088	YARS	0.246795939	7.26E-09
5089	OGFRL1	-0.188605582	1.12E-05
5090	MID1IP1	0.083812695	0.052688044
5091	ITPKB	-0.166860179	0.000105458
5092	PTPRC	-0.063500379	0.142426861
5093	COMMD2	-0.386287465	1.75E-20
5094	MEGF8	-0.193298041	6.70E-06
5095	GGT7	-0.013680828	0.75222027
5096	ATP13A1	0.609198295	1.18E-55
5097	FAM175B	-0.455533873	9.09E-29
5098	SDE2	-0.395590863	1.74E-21
5099	ELP4	-0.346917455	1.42E-16
5100	SERPINB9	0.070202608	0.104804485
5101	RSRC2	0.089633339	0.038214256
5102	AZGP1	0.04150741	0.337943462

5103	ZFAND1	-0.345996813	1.72E-16
5104	ATF1	-0.417692879	5.29E-24
5105	NISCH	0.209679648	9.93E-07
5106	PHKB	-0.58253412	6.23E-50
5107	TCEB1	0.183785313	1.89E-05
5108	RASSF2	-0.125220716	0.003719556
5109	SNX14	-0.367589146	1.47E-18
5110	DNAJC13	-0.340487276	5.48E-16
5111	ADO	-0.324870543	1.29E-14
5112	PARP9	-0.121669467	0.00483062
5113	PPP5C	0.357644213	1.38E-17
5114	SIGIRR	0.441018515	7.20E-27
5115	PLEKHA6	0.068668194	0.112635455
5116	RAP1GAP	-0.155198419	0.000314313
5117	CRIPT	-0.271079528	1.83E-10
5118	ZNF33B	-0.399276901	6.81E-22
5119	MLH1	-0.193656558	6.43E-06
5120	C15orf39	0.076386743	0.077517089
5121	MIA3	-0.301254456	1.10E-12
5122	SLC18B1	-0.3391389	7.25E-16
5123	MAN2A1	-0.507058595	2.71E-36
5124	METTL13	-0.100623821	0.019917258
5125	GTF3C1	0.006667882	0.877712609
5126	DNAJC7	0.091154711	0.035041968
5127	CFH	-0.092317258	0.032771578
5128	ACAA1	0.119271738	0.005741738
5129	CDKN2AIP	-0.334717863	1.80E-15
5130	HBEGF	0.060703345	0.160890887
5131	PRKAR2A	-0.552077116	5.23E-44
5132	NTAN1	0.010293912	0.81223149
5133	LZTR1	0.574083596	3.17E-48
5134	DHX30	0.181431031	2.42E-05
5135	SH3D19	-0.404037567	2.00E-22
5136	ATG5	-0.379952108	8.11E-20
5137	C11orf74	-0.077582366	0.072974319
5138	TXNDC17	0.396273335	1.46E-21
5139	MKL1	0.313925956	1.06E-13
5140	TRPV4	0.005306263	0.902544212
5141	PCCA	-0.419741782	3.02E-24
5142	SCAF8	-0.417182485	6.08E-24
5143	YRDC	0.030584054	0.480236793
5144	ADCK3	-0.033933975	0.433459836
5145	DHDDS	-0.075191766	0.082284296
5146	NFE2L3	0.145392993	0.000743476
5147	LZIC	-0.466460814	2.93E-30
5148	PSPC1	0.194480562	5.87E-06
5149	FAM43A	-0.011541057	0.78998513
5150	FECH	-0.485335584	5.78E-33
5151	KANSL3	0.196912002	4.46E-06
5152	RSPRY1	-0.297332994	2.21E-12
5153	HAO2	-0.123618351	0.004188515
5154	PCOLCE	0.302935623	8.10E-13
5155	PTPRJ	-0.256228604	1.82E-09
5156	PNPLA8	-0.462694997	9.72E-30
5157	8-Mar	-0.377199405	1.56E-19
5158	FAM21A	0.012994824	0.764268083
5159	MAP2K7	0.405586027	1.33E-22
5160	NOL10	-0.098750252	0.022351361

5161	FAM172A	-0.519731102	2.38E-38
5162	VKORC1L1	-0.376083107	2.03E-19
5163	MRPL19	-0.384901407	2.46E-20
5164	LPIN3	0.436023249	3.09E-26
5165	PPP2R5B	0.432108567	9.52E-26
5166	B9D2	0.399415518	6.57E-22
5167	ZNF282	0.423060279	1.21E-24
5168	FAM58A	0.417384255	5.75E-24
5169	RALGDS	0.429079169	2.25E-25
5170	C12orf49	-0.387406008	1.33E-20
5171	OXR1	-0.422130702	1.57E-24
5172	C16orf91	0.322867315	1.91E-14
5173	BOP1	0.720543273	8.47E-87
5174	CDC42BPA	-0.330221582	4.46E-15
5175	PAQR5	-0.388346199	1.06E-20
5176	COL4A3BP	-0.554776244	1.65E-44
5177	DTNBP1	0.097175685	0.024592467
5178	KLHDC2	-0.337769028	9.62E-16
5179	PJA1	-0.373605172	3.64E-19
5180	BUD13	0.129543151	0.002682192
5181	CES3	0.129893428	0.002610941
5182	MAN2B2	-0.098660052	0.022474789
5183	MOCS1	-0.176214365	4.16E-05
5184	TTLL12	0.188517	1.14E-05
5185	LGALS9	0.41545031	9.71E-24
5186	PDE8A	-0.111230633	0.010031302
5187	PNCK	0.133911282	0.001908434
5188	FARS2	-0.147888286	0.000600138
5189	TP53I13	0.717958937	6.62E-86
5190	AMPD2	0.398825609	7.64E-22
5191	WIPI1	0.065217905	0.131920798
5192	SYNJ2BP	-0.504074354	8.02E-36
5193	GMFB	-0.461569884	1.39E-29
5194	ARPC5L	0.479349864	4.35E-32
5195	EMC1	-0.352836573	3.96E-17
5196	MAP3K6	0.358485929	1.14E-17
5197	SLIT3	-0.16543872	0.000120956
5198	HGS	0.529918855	4.57E-40
5199	UBE4A	-0.457081537	5.63E-29
5200	CLCN7	0.328421455	6.39E-15
5201	CYP4A11	-0.032179102	0.457629271
5202	GTF2A1	-0.511942471	4.47E-37
5203	CMTM4	-0.536333036	3.54E-41
5204	GSK3B	-0.438262675	1.61E-26
5205	CNOT6L	-0.535322724	5.32E-41
5206	TSPYL2	0.33953175	6.68E-16
5207	BTF3L4	-0.222554317	1.98E-07
5208	C10orf76	-0.34752988	1.24E-16
5209	PPAPDC1B	0.182216134	2.23E-05
5210	MARK2	0.174982924	4.71E-05
5211	ARHGEF37	-0.349332803	8.44E-17
5212	WDSUB1	-0.316691729	6.29E-14
5213	SLC48A1	-0.066883955	0.122311804
5214	TBC1D15	-0.509091766	1.28E-36
5215	NFX1	-0.134049592	0.001887669
5216	LYSMD2	-0.012923454	0.765524846
5217	UBR2	-0.370942779	6.77E-19
5218	MTFR1	-0.213674412	6.09E-07

5219	ZNF12	-0.342352445	3.71E-16
5220	NUP133	-0.462125574	1.16E-29
5221	ERGIC2	-0.329107918	5.58E-15
5222	STX8	0.243948622	1.09E-08
5223	EHD3	-0.110617856	0.010452888
5224	RWDD1	-0.162197969	0.000164658
5225	AIMP2	0.06713034	0.120938286
5226	CASP4	0.290936518	6.76E-12
5227	PRKDC	-0.336861059	1.16E-15
5228	FZR1	0.538142647	1.70E-41
5229	FBLN2	-0.070259985	0.104520237
5230	PAK4	0.279862633	4.39E-11
5231	LRRC8D	-0.197511892	4.16E-06
5232	SPON1	-0.056114164	0.195006561
5233	DGAT1	0.57994253	2.10E-49
5234	DCUN1D5	0.099422979	0.021449211
5235	ELP2	-0.244981901	9.42E-09
5236	BRD4	0.357746705	1.35E-17
5237	SART3	-0.052512099	0.225281183
5238	WBSCR22	0.588082853	4.44E-51
5239	SYMPK	0.475965047	1.34E-31
5240	TXNL4A	0.310073573	2.19E-13
5241	GPER1	0.073772409	0.088251677
5242	LGALSL	-0.264640171	5.03E-10
5243	VPS37C	0.149275617	0.000531995
5244	SOS1	-0.418419312	4.34E-24
5245	FAM220A	-0.263919276	5.63E-10
5246	NADK	0.274749987	1.01E-10
5247	HECA	-0.324640864	1.35E-14
5248	PDGFC	-0.337107816	1.10E-15
5249	LPP	-0.417223493	6.01E-24
5250	C6orf57	-0.13383273	0.00192032
5251	TMEM254	-0.210154311	9.37E-07
5252	LRRC40	-0.28876347	9.82E-12
5253	ALDH3B1	0.203372496	2.11E-06
5254	NCOA3	-0.284179537	2.14E-11
5255	KLHL21	-0.108727533	0.011854133
5256	NF2	0.019137501	0.658737582
5257	RFWD2	-0.10134643	0.019042058
5258	NOA1	-0.335692888	1.47E-15
5259	MED12	0.025962513	0.549032128
5260	NFIA	-0.254644321	2.30E-09
5261	NAT14	0.326894881	8.66E-15
5262	UBE3A	-0.491525424	6.88E-34
5263	C11orf57	-0.398033386	9.35E-22
5264	BICD2	-0.135151318	0.001729505
5265	MX1	0.027031794	0.532694058
5266	MRPL4	0.369808908	8.80E-19
5267	MAP1S	0.438772494	1.39E-26
5268	ZNF655	-0.050102051	0.247321426
5269	NUBP2	0.590632649	1.29E-51
5270	SAR1B	-0.291250288	6.40E-12
5271	NPRL3	0.410596009	3.56E-23
5272	TPST1	-0.133246417	0.002011204
5273	KANSL2	0.136189077	0.001591733
5274	BTBD10	-0.256604404	1.72E-09
5275	SLC25A30	-0.391314307	5.07E-21
5276	TSKU	0.162364715	0.000162088

5277	BLZF1	-0.284238525	2.12E-11
5278	RPL22L1	0.457943916	4.31E-29
5279	ITGB4	0.097227788	0.024515313
5280	MRPS5	0.238525749	2.34E-08
5281	LSP1	0.363841209	3.44E-18
5282	PRF1	0.260902125	8.96E-10
5283	ZMYM6NB	0.067894631	0.116754094
5284	ACOT13	-0.050519979	0.243395231
5285	BLOC1S4	0.279356841	4.77E-11
5286	PISD	0.464552467	5.39E-30
5287	PLEKHJ1	0.390918856	5.59E-21
5288	SEMA4C	0.206031215	1.54E-06
5289	DSCR3	-0.364568661	2.92E-18
5290	RCBTB1	-0.285897122	1.60E-11
5291	MTMR10	-0.446951979	1.24E-27
5292	ELP3	-0.316228654	6.87E-14
5293	ERAP2	0.034598355	0.42450521
5294	SMURF1	-0.093263264	0.03101792
5295	TEAD2	0.094018254	0.029676582
5296	SLC20A1	0.266844553	3.57E-10
5297	ZNF385A	0.42569806	5.82E-25
5298	EMILIN1	0.198001817	3.93E-06
5299	C5AR1	0.057797052	0.18192909
5300	PFDN4	0.103871654	0.016241489
5301	ARHGEF7	-0.057165759	0.186756585
5302	AIDA	-0.389689946	7.59E-21
5303	GLRX3	-0.034520087	0.425554493
5304	POP5	0.588916295	2.97E-51
5305	PNPLA4	-0.23793602	2.54E-08
5306	DNTTIP2	-0.169319427	8.30E-05
5307	LYN	-0.116049086	0.007209072
5308	ZC3H13	-0.4046347	1.71E-22
5309	GMPPA	0.661719927	1.12E-68
5310	ITIH5	-0.099023324	0.02198127
5311	NFYA	-0.113861927	0.008387695
5312	RECQL	-0.348827772	9.41E-17
5313	SNX11	0.227747008	1.01E-07
5314	RAB36	0.015489191	0.720755325
5315	PPME1	-0.051912327	0.230630812
5316	TRMT1L	-0.4268571	4.21E-25
5317	BRD8	0.173598811	5.42E-05
5318	INTS3	0.238255557	2.43E-08
5319	NDUFA11	0.563388548	3.89E-46
5320	FBXO6	0.471250599	6.26E-31
5321	TACSTD2	-0.165936803	0.000115297
5322	TUBG2	0.49525926	1.87E-34
5323	WRB	-0.216425839	4.32E-07
5324	CD83	-0.085298596	0.048616154
5325	CCDC146	0.026400727	0.542306722
5326	TMEM70	-0.072532731	0.093743888
5327	B3GALT6	0.420760478	2.29E-24
5328	PLAA	-0.35763165	1.38E-17
5329	SPG11	-0.417949644	4.93E-24
5330	PAFAH2	-0.386970293	1.48E-20
5331	STXBP2	0.546793156	4.85E-43
5332	IL6R	-0.071747932	0.097359877
5333	TMEM106B	-0.519884152	2.25E-38
5334	MIPEP	-0.311266942	1.75E-13

5335	TPP2	-0.157859039	0.000246606
5336	TBC1D23	-0.366216896	2.01E-18
5337	HOXA5	-0.038840512	0.369921337
5338	ORC3	-0.298272253	1.87E-12
5339	BTNL9	-0.015369889	0.722817356
5340	FMR1	-0.440893829	7.47E-27
5341	PGAM5	0.348866765	9.33E-17
5342	CYTH2	0.494497802	2.44E-34
5343	DIP2C	-0.29819102	1.90E-12
5344	USP16	-0.26875116	2.64E-10
5345	FGFR1	-0.006399322	0.882601596
5346	GPANK1	0.082415839	0.056771353
5347	MS4A4A	-0.064579813	0.135751807
5348	GRIPAP1	0.459063797	3.04E-29
5349	DIMT1	0.002531226	0.95342159
5350	MCM6	0.096896314	0.025009748
5351	SASH3	0.259148247	1.17E-09
5352	TADA2B	-0.248271346	5.87E-09
5353	GJB2	0.185506524	1.57E-05
5354	SLC22A5	0.074882286	0.083556825
5355	ARHGAP12	-0.450971049	3.68E-28
5356	PTN	-0.050201781	0.24638053
5357	MFNG	0.202514897	2.33E-06
5358	FGFR1OP2	-0.064146053	0.138404564
5359	CCNL1	0.335222504	1.62E-15
5360	SLC35A1	0.086993267	0.044297159
5361	MTERF3	0.228658031	8.92E-08
5362	DYRK1A	-0.253241581	2.83E-09
5363	ACBD4	0.297710697	2.07E-12
5364	RABL6	0.457658898	4.71E-29
5365	POU5F1	0.308106457	3.15E-13
5366	LNPEP	-0.483698292	1.01E-32
5367	DERL2	0.21522683	5.02E-07
5368	NIP7	0.077118477	0.074710383
5369	MCAT	0.314763276	9.07E-14
5370	CISH	0.027484421	0.525852816
5371	TSEN15	0.172411639	6.11E-05
5372	COMMD5	0.647175378	8.16E-65
5373	GFM2	-0.356645577	1.72E-17
5374	ATG3	-0.140930881	0.00108141
5375	VPS72	0.305431019	5.15E-13
5376	FCF1	-0.385070481	2.36E-20
5377	TRIM25	-0.095262975	0.027573748
5378	CDK14	-0.301987587	9.61E-13
5379	SH3GLB2	0.431014638	1.30E-25
5380	CAAP1	-0.291007952	6.68E-12
5381	NRGN	0.27587111	8.45E-11
5382	PET100	0.536065141	3.95E-41
5383	CTHRC1	0.084524652	0.050702883
5384	TMEM19	-0.178556178	3.27E-05
5385	FAM26F	0.218502682	3.33E-07
5386	TTC17	-0.095479256	0.027221745
5387	ZNRF2	-0.343423941	2.96E-16
5388	HMGCS2	-0.120206046	0.005369771
5389	RNF126	0.650756092	9.55E-66
5390	PYCARD	0.550733264	9.25E-44
5391	CTTNBP2NL	-0.37240532	4.82E-19
5392	ENY2	0.125997965	0.003509686

5393	RQCD1	0.4295695	1.96E-25
5394	SNTB1	-0.43054035	1.49E-25
5395	MED13L	-0.402870146	2.70E-22
5396	NSRP1	-0.031854462	0.462181705
5397	MRI1	0.322970159	1.87E-14
5398	SLC5A12	-0.001109093	0.979581651
5399	OCRL	-0.263330318	6.17E-10
5400	SLC23A2	-0.241458618	1.55E-08
5401	PANX1	-0.146376765	0.000683547
5402	TPD52	-0.281178955	3.53E-11
5403	RNF216	-0.025236734	0.560260381
5404	RBMX2	0.286396386	1.47E-11
5405	RGP1	-0.395872191	1.62E-21
5406	PEX16	0.515542683	1.16E-37
5407	GNPTAB	-0.443043764	3.96E-27
5408	BECN1	-0.24658402	7.49E-09
5409	MAP1B	-0.278859493	5.18E-11
5410	CUL5	-0.517975294	4.64E-38
5411	NOC4L	0.663179242	4.44E-69
5412	SLC41A2	-0.196607952	4.61E-06
5413	PTH1R	-0.005714256	0.895092609
5414	TIMM22	0.213990882	5.85E-07
5415	ABHD15	0.128935901	0.002809925
5416	PIGBOS1	0.339897609	6.19E-16
5417	COG7	0.141728193	0.001012168
5418	MBD3	0.620684064	2.71E-58
5419	SCRIB	0.502121483	1.62E-35
5420	CARM1	0.27001199	2.17E-10
5421	MEGF9	-0.563333753	3.99E-46
5422	SETD7	-0.513985399	2.09E-37
5423	ANKS1A	-0.300785671	1.19E-12
5424	UNC5CL	0.160987901	0.000184482
5425	INO80E	0.567654199	5.83E-47
5426	CREBBP	-0.266943503	3.51E-10
5427	KANK1	-0.07964971	0.065633333
5428	HGH1	0.606460075	4.82E-55
5429	ITGB1BP1	0.263843715	5.70E-10
5430	OAS3	-0.022598555	0.601983613
5431	ATE1	-0.499459733	4.21E-35
5432	RABGGTA	0.419758806	3.01E-24
5433	TFB2M	-0.291657496	5.97E-12
5434	NETO2	-0.038126516	0.378790919
5435	NUP54	-0.31204831	1.51E-13
5436	RHOJ	-0.040939022	0.344605905
5437	STX6	-0.166392013	0.000110344
5438	FOXN3	-0.486358902	4.08E-33
5439	MSRB3	-0.119971012	0.005461242
5440	MSL2	-0.23560174	3.50E-08
5441	ADCY6	-0.073690484	0.088606468
5442	RBM6	0.387072092	1.45E-20
5443	MRC2	0.301985149	9.62E-13
5444	RBM15B	-0.048271081	0.265042602
5445	SLC22A17	0.069321413	0.109247253
5446	DNAJC11	-0.251302255	3.77E-09
5447	VASH1	-0.035015833	0.41893397
5448	GALK1	0.566240264	1.10E-46
5449	PDPR	-0.135007589	0.001749426
5450	C5orf51	-0.434370348	4.98E-26

5451	ROCK2	-0.339332696	6.96E-16
5452	NCAPD2	0.20310771	2.18E-06
5453	F8	-0.302355163	9.00E-13
5454	TRUB1	-0.538772441	1.32E-41
5455	CSTF1	-0.251788624	3.51E-09
5456	AAED1	-0.071887631	0.096708198
5457	TTC9C	0.186921965	1.35E-05
5458	NR2F1	-0.123742123	0.00415047
5459	VLDLR	-0.255715733	1.96E-09
5460	H3F3A	-0.052264347	0.227480182
5461	KIAA0355	-0.255874482	1.92E-09
5462	PNISR	0.21416091	5.73E-07
5463	GOLGA3	0.222459098	2.01E-07
5464	H2AFY	0.184347052	1.78E-05
5465	PMPCA	0.384145434	2.95E-20
5466	PSMC6	-0.046246416	0.285632345
5467	UBFD1	-0.15888799	0.000224289
5468	DOCK6	0.094935012	0.028114983
5469	URGCP	0.254704067	2.28E-09
5470	SURF6	0.176298841	4.12E-05
5471	INPP5A	-0.266628208	3.69E-10
5472	AKT2	0.201284659	2.69E-06
5473	REG1A	-0.025748902	0.5523253
5474	DHRS4	0.078232443	0.07059699
5475	RBMS1	0.000657733	0.987890304
5476	SNRNP25	0.362021329	5.19E-18
5477	EPB41	-0.057585892	0.183533452
5478	JMJD1C	-0.367073373	1.65E-18
5479	NUP43	-0.093529094	0.030539836
5480	RNMTL1	0.264822598	4.89E-10
5481	AKAP10	-0.30121471	1.10E-12
5482	PDHX	-0.467297973	2.24E-30
5483	GGNBP2	-0.062368542	0.149692872
5484	CWC25	0.253583415	2.69E-09
5485	PTPN9	-0.2965004	2.56E-12
5486	R3HDM2	0.052664927	0.223932286
5487	SYT11	-0.236990456	2.89E-08
5488	TLR4	-0.38471642	2.57E-20
5489	CDH13	-0.254284001	2.43E-09
5490	TBC1D22B	0.02257177	0.602414295
5491	TANK	-0.012727317	0.768981782
5492	ATP9A	-0.393982046	2.60E-21
5493	MITF	-0.53291931	1.39E-40
5494	LGI4	0.059966012	0.166046257
5495	RRAS2	-0.100662634	0.019869373
5496	OSBPL11	-0.247979224	6.12E-09
5497	CD3D	0.350841506	6.10E-17
5498	SRSF10	-0.025034975	0.563401313
5499	PCOLCE2	0.103955425	0.01615512
5500	CASP1	0.137128293	0.001475805
5501	CNPY4	0.362910336	4.25E-18
5502	CCDC9	0.40024897	5.31E-22
5503	EDNRA	-0.143757495	0.000853977
5504	AMMECR1L	0.033542993	0.438780087
5505	BCAP29	-0.310641744	1.97E-13
5506	TRIQK	-0.399213022	6.92E-22
5507	VEZT	-0.229320746	8.17E-08
5508	SDSL	0.215801156	4.67E-07

5509	PPP1R37	0.433104347	7.16E-26
5510	HKDC1	0.042034002	0.331845082
5511	C2CD5	-0.269502623	2.35E-10
5512	KCNE4	-0.024943256	0.564831958
5513	C8orf82	0.339420144	6.84E-16
5514	LPPR2	0.339485329	6.75E-16
5515	TIMM50	0.553848986	2.46E-44
5516	TFAM	-0.527854134	1.03E-39
5517	BAHD1	-0.19732186	4.25E-06
5518	MAP3K2	-0.366337198	1.95E-18
5519	ARHGEF6	-0.17175169	6.52E-05
5520	DENND4C	-0.539073088	1.17E-41
5521	ZNF592	-0.096878624	0.025036375
5522	CRISPLD2	-0.003554339	0.934630753
5523	MAN1A2	-0.557491215	5.13E-45
5524	GABPA	-0.411896257	2.52E-23
5525	FAM83H	0.211201417	8.25E-07
5526	TTI1	-0.196346802	4.75E-06
5527	CLIP2	0.143756796	0.000854027
5528	KRT10	0.507097105	2.67E-36
5529	DUSP11	-0.070728693	0.102221052
5530	ZNF580	0.584936583	2.00E-50
5531	ZNF33A	-0.354694785	2.64E-17
5532	SYTL2	-0.11093971	0.010229535
5533	TMEM63A	0.116395058	0.007036869
5534	CECR5	0.2147955	5.30E-07
5535	NUDT12	-0.366589566	1.84E-18
5536	GAR1	0.149732978	0.000511155
5537	GPR183	-0.005262834	0.903337925
5538	MAN2C1	0.418034645	4.82E-24
5539	SLC25A44	-0.010775736	0.803617805
5540	AKTIP	-0.389579174	7.80E-21
5541	ANO10	-0.206523008	1.45E-06
5542	FBXO34	-0.324279429	1.45E-14
5543	USP24	-0.267271799	3.34E-10
5544	SERPINA6	0.023515543	0.587323333
5545	IL18BP	0.322428406	2.08E-14
5546	PARP3	0.149174188	0.000536722
5547	ZNF106	-0.526622552	1.67E-39
5548	IDO1	0.056376656	0.192922714
5549	STRN3	-0.296574815	2.53E-12
5550	CASP6	-0.023005075	0.595464094
5551	PPP3CC	0.010671216	0.805484344
5552	DARS2	-0.287853325	1.15E-11
5553	FRMD3	-0.285365083	1.75E-11
5554	ZCCHC14	-0.316154286	6.97E-14
5555	SIRT1	-0.397217824	1.15E-21
5556	CPNE2	-0.034890784	0.420598231
5557	CHMP4C	-0.289362774	8.86E-12
5558	KAT2B	-0.520499481	1.78E-38
5559	LPL	-0.146270707	0.000689786
5560	CAMK2D	-0.326942313	8.58E-15
5561	SAP30BP	0.624421099	3.55E-59
5562	HCST	0.53047582	3.67E-40
5563	NOSTRIN	-0.037058852	0.392295034
5564	SDAD1	-0.409187693	5.18E-23
5565	ATP11B	-0.442134289	5.18E-27
5566	ADM2	0.245580372	8.65E-09

5567	CERS5	0.355546666	2.19E-17
5568	DPY19L1	-0.250611028	4.17E-09
5569	IKBKAP	-0.191769095	7.94E-06
5570	ABCA2	0.010528743	0.808030453
5571	EXOC2	-0.344115296	2.56E-16
5572	TMEM183A	-0.132253065	0.00217421
5573	PRKAA2	-0.607268555	3.18E-55
5574	EXOSC6	0.0665574	0.124150942
5575	DNASE1L1	0.007030994	0.871109779
5576	ALPK2	0.194264598	6.01E-06
5577	MRPS23	0.147716969	0.000609093
5578	TRMT2A	0.769522659	7.01E-106
5579	BACE1	-0.443575181	3.39E-27
5580	PPM1M	0.470228294	8.73E-31
5581	CCDC64	0.207023394	1.37E-06
5582	ZC3HAV1	-0.398330979	8.67E-22
5583	ZNF205	0.535828929	4.34E-41
5584	NMI	0.156915782	0.000268873
5585	NLK	-0.265096312	4.69E-10
5586	OFD1	0.267960346	3.00E-10
5587	ANO1	-0.081859537	0.058469067
5588	NUP160	-0.337214205	1.08E-15
5589	TMTC3	-0.52016724	2.02E-38
5590	BBX	-0.556715127	7.17E-45
5591	NR1D2	-0.458795572	3.30E-29
5592	DPAGT1	0.14227825	0.00096681
5593	FAM208A	-0.349864171	7.53E-17
5594	NAE1	-0.133094521	0.002035383
5595	TEX2	-0.290993105	6.69E-12
5596	AKAP8L	0.568487928	4.01E-47
5597	ZNF512	0.014670786	0.734940621
5598	SLC6A6	-0.072559338	0.0936232
5599	ANAPC2	0.466502809	2.90E-30
5600	MANEA	-0.492836945	4.36E-34
5601	GGCX	-0.335155523	1.64E-15
5602	RABGAP1	-0.248042338	6.07E-09
5603	EHMT2	0.305986102	4.65E-13
5604	CHD8	-0.245655642	8.55E-09
5605	TAF10	0.59391058	2.62E-52
5606	IMPDH1	0.456092802	7.65E-29
5607	ZYG11B	-0.635858254	5.97E-62
5608	TOR4A	0.240501254	1.77E-08
5609	CCDC92	0.434860238	4.32E-26
5610	TRAPPC11	-0.492762125	4.47E-34
5611	CD3E	0.317411918	5.48E-14
5612	ITGA7	0.062753096	0.147193189
5613	PAN3	0.026750727	0.536964738
5614	FAM122A	-0.408368371	6.43E-23
5615	ATF7	-0.181835582	2.32E-05
5616	SPOCK1	0.023506315	0.587470041
5617	KCMF1	-0.028762878	0.50677443
5618	ARHGEF15	-0.045676495	0.29161757
5619	XPA	-0.226010173	1.26E-07
5620	MADD	0.104673165	0.015431476
5621	ZDHHC3	-0.284802801	1.93E-11
5622	TCF3	0.517712565	5.13E-38
5623	SLC25A24	-0.50863873	1.52E-36
5624	GEMIN7	0.53600772	4.04E-41

5625	CCSER2	-0.511002865	6.34E-37
5626	CPNE8	-0.058320762	0.177994849
5627	TMEM18	-0.046867387	0.279205897
5628	NFATC2IP	0.323787794	1.60E-14
5629	STXBP1	-0.127794658	0.003065074
5630	DCAF15	0.723648179	6.94E-88
5631	SH2B1	0.449537532	5.68E-28
5632	CNN1	0.109116125	0.011553218
5633	PEX14	0.33217949	3.01E-15
5634	ACOT2	-0.040996705	0.343925976
5635	COMMD9	0.13197367	0.002222186
5636	SAV1	-0.434160984	5.29E-26
5637	INPP1	0.085658575	0.047670351
5638	KATNA1	-0.157047575	0.000265653
5639	RRP9	0.548069493	2.84E-43
5640	RGS19	0.454751308	1.16E-28
5641	NT5E	-0.189606508	1.01E-05
5642	APBB2	-0.323427638	1.71E-14
5643	GIGYF2	-0.327704408	7.37E-15
5644	TTC33	-0.513145263	2.86E-37
5645	DGKZ	0.577527549	6.48E-49
5646	TRIM2	-0.585636357	1.43E-50
5647	RHBDF2	0.565082216	1.84E-46
5648	PRPF3	0.442395842	4.80E-27
5649	DYNC2LI1	-0.155947219	0.000293684
5650	MAP3K5	-0.424389448	8.38E-25
5651	ZMYND8	0.132124602	0.00219615
5652	STK32B	-0.303213019	7.70E-13
5653	MTFP1	0.278017022	5.95E-11
5654	ZSWIM7	0.260995449	8.83E-10
5655	NBAS	-0.479078839	4.76E-32
5656	C17orf70	0.692632232	1.17E-77
5657	COL6A3	0.113385031	0.008666448
5658	FAM120B	-0.432783332	7.85E-26
5659	SPIDR	0.023809172	0.58266404
5660	SEC24A	-0.347959651	1.13E-16
5661	THEMIS2	0.159239843	0.000217102
5662	FAM207A	0.432310493	8.99E-26
5663	METTL1	0.502980915	1.19E-35
5664	WDFY3	-0.466123192	3.27E-30
5665	STX2	0.228853363	8.69E-08
5666	S100A8	0.330957102	3.85E-15
5667	ECHDC2	0.310329581	2.09E-13
5668	HSPH1	-0.089309067	0.038921012
5669	NUAK2	0.125982843	0.003513665
5670	LPCAT3	-0.376455526	1.86E-19
5671	INAFM1	0.591717212	7.64E-52
5672	GAREM	-0.360549221	7.22E-18
5673	PI4K2A	0.152690306	0.000393687
5674	MPST	0.593003215	4.08E-52
5675	DLC1	-0.257411097	1.52E-09
5676	PPP1R26	0.199103866	3.47E-06
5677	RAB2B	0.16858368	8.92E-05
5678	RBM25	0.194418971	5.91E-06
5679	DDA1	0.596737682	6.49E-53
5680	PAK1IP1	0.067227726	0.120398725
5681	RBM18	-0.472234824	4.55E-31
5682	DYM	-0.526104538	2.04E-39

5683	SLC30A6	-0.464308814	5.83E-30
5684	ZFAND2A	0.457989543	4.25E-29
5685	FAF1	-0.138746242	0.001294134
5686	TMF1	-0.42084438	2.23E-24
5687	SYNE2	-0.346987602	1.39E-16
5688	PIK3R4	-0.462987004	8.86E-30
5689	IAH1	0.302964745	8.06E-13
5690	SCFD1	-0.259395796	1.13E-09
5691	ANKRD33B	-0.426293288	4.93E-25
5692	TRIM69	-0.082719326	0.055862553
5693	NAAA	0.029525578	0.495567918
5694	CLDN12	-0.363527384	3.70E-18
5695	PPM1A	-0.542014016	3.51E-42
5696	TCEA2	0.500311279	3.11E-35
5697	ZNF830	0.109077678	0.011582686
5698	KIAA1671	-0.390670181	5.95E-21
5699	CCDC101	0.436126549	3.00E-26
5700	COL5A1	0.111271138	0.010003975
5701	PRKCI	-0.269124702	2.49E-10
5702	EDEM3	-0.313517299	1.15E-13
5703	HDHD1	-0.179353988	3.01E-05
5704	TLE1	0.038981259	0.368188222
5705	SLC35C2	0.5937996	2.76E-52
5706	TRIM5	-0.166688394	0.000107227
5707	ATG4D	0.269763266	2.25E-10
5708	ARHGAP31	-0.399783478	5.98E-22
5709	HSPA12A	-0.115794178	0.007338353
5710	B4GALT7	0.398228537	8.89E-22
5711	TM4SF5	0.153226293	0.000375299
5712	DTYMK	0.636788902	3.50E-62
5713	SPG7	0.517103429	6.46E-38
5714	MRPS33	-0.058826303	0.174257336
5715	LEMD2	0.471336877	6.09E-31
5716	RBBP5	-0.36377449	3.50E-18
5717	EVL	0.459016627	3.08E-29
5718	SMTN	0.197481319	4.18E-06
5719	CC2D1A	0.544444848	1.29E-42
5720	TOM1L1	-0.43048064	1.51E-25
5721	ISG20L2	0.051306546	0.236124797
5722	MFSD6	-0.42767773	3.34E-25
5723	C20orf96	0.216955752	4.04E-07
5724	UBE2E2	0.012651134	0.77032574
5725	RPA3	-0.080505618	0.062777211
5726	C16orf62	-0.267395417	3.27E-10
5727	BRAT1	0.631759549	6.07E-61
5728	WDR77	0.396845506	1.26E-21
5729	WDR75	0.274749183	1.01E-10
5730	SOCS5	-0.349072491	8.93E-17
5731	PARP12	0.495362274	1.80E-34
5732	GOLGA4	-0.370207485	8.03E-19
5733	BASP1	0.26783909	3.05E-10
5734	DDX60	-0.385714349	2.02E-20
5735	TMED8	-0.523792152	5.01E-39
5736	EMC9	0.576392969	1.09E-48
5737	FMOD	-0.055386317	0.200870727
5738	UPP1	0.30653297	4.21E-13
5739	ZC3H4	0.185534574	1.57E-05
5740	CERCAM	0.366154907	2.04E-18

5741	RBM38	0.378495619	1.15E-19
5742	RANBP3	0.478361044	6.05E-32
5743	GSTM3	-0.14478753	0.000782741
5744	MAPK6	-0.168414894	9.07E-05
5745	PCNXL3	0.366048553	2.09E-18
5746	POLR3H	0.229207479	8.29E-08
5747	MACROD1	0.202882888	2.23E-06
5748	RNF141	-0.626105392	1.41E-59
5749	CLDN5	0.146154158	0.000696702
5750	DFFA	-0.146986649	0.000648677
5751	FAR1	-0.226444491	1.19E-07
5752	EIF2AK2	-0.346989915	1.39E-16
5753	DIXDC1	-0.332997297	2.55E-15
5754	KCTD5	0.382572709	4.32E-20
5755	FAM195A	0.383421101	3.52E-20
5756	GJB1	-0.100465657	0.020113426
5757	MTMR14	0.364974306	2.66E-18
5758	PLEKHO1	0.524710111	3.51E-39
5759	UGT8	-0.435307349	3.80E-26
5760	CSNK1G3	-0.389179711	8.61E-21
5761	IPO9	-0.149740155	0.000510834
5762	RAP1GDS1	-0.39611833	1.52E-21
5763	ZNF320	-0.096754969	0.025223185
5764	FIG4	-0.387816921	1.20E-20
5765	INTS10	0.055284109	0.201704368
5766	SMIM19	-0.001223409	0.977477629
5767	GTPBP1	0.12750558	0.003132963
5768	ALOX5AP	-0.054255045	0.210238256
5769	NUP205	-0.320417703	3.08E-14
5770	FDXR	0.269803934	2.24E-10
5771	GIMAP2	-0.021259858	0.623675975
5772	HLTF	-0.391731496	4.57E-21
5773	IGIP	-0.427091627	3.94E-25
5774	CNOT6	-0.340894493	5.03E-16
5775	CSRNP2	0.040027795	0.355460068
5776	SLC39A8	-0.4259843	5.37E-25
5777	SCNM1	0.670295878	4.63E-71
5778	TK2	-0.085417784	0.048301276
5779	TMEM200A	-0.215229263	5.02E-07
5780	SFRP2	-0.03892844	0.368838027
5781	NOP9	-0.246172903	7.94E-09
5782	RAPGEF2	-0.45113302	3.50E-28
5783	FOXO4	-0.225108137	1.42E-07
5784	ALKBH3	0.070677623	0.102469605
5785	CTNS	0.210434879	9.06E-07
5786	FAM222B	0.125055983	0.003765471
5787	C17orf58	0.079993428	0.064473782
5788	ZBTB1	-0.171944988	6.40E-05
5789	TXNL1	-0.234676664	3.97E-08
5790	VIPAS39	-0.111223776	0.010035935
5791	CCHCR1	0.353115254	3.72E-17
5792	SOWAHC	-0.333085275	2.51E-15
5793	CYP1B1	-0.07749671	0.073292377
5794	LRCH1	-0.227797055	9.99E-08
5795	SUMF1	-0.290138656	7.76E-12
5796	USP47	-0.340286303	5.71E-16
5797	NSL1	-0.217371278	3.84E-07
5798	DCUN1D4	-0.023910906	0.581053753

5799	HCK	0.139685457	0.001198354
5800	WBP4	-0.176609297	4.00E-05
5801	TBK1	-0.197487924	4.17E-06
5802	KITLG	-0.405732352	1.28E-22
5803	ZDHHC8	0.557783504	4.52E-45
5804	COPG2	-0.172800154	5.87E-05
5805	CADPS2	-0.380821943	6.58E-20
5806	COPS5	0.250653269	4.15E-09
5807	RAPGEF5	-0.295765125	2.91E-12
5808	TSC2	0.288874609	9.64E-12
5809	STX7	-0.378680042	1.10E-19
5810	FASTKD5	-0.308854156	2.74E-13
5811	RAB28	-0.271838424	1.62E-10
5812	C6orf1	0.582277076	7.03E-50
5813	TMEM252	-0.310131231	2.16E-13
5814	PALD1	0.122212832	0.004643211
5815	TRIT1	0.22665507	1.16E-07
5816	DENND2D	0.135889677	0.00163041
5817	FMNL3	-0.049949787	0.248762796
5818	DPH3	-0.356339823	1.84E-17
5819	TGIF2	0.134534511	0.001816489
5820	GPX7	0.136592449	0.001540957
5821	ADCY5	-0.14000077	0.001167693
5822	ATG16L1	0.091765422	0.033833125
5823	SEMA6B	0.139452863	0.001221446
5824	SMPD4	0.440686771	7.94E-27
5825	TAF1D	0.194055613	6.15E-06
5826	CHDH	-0.243600206	1.15E-08
5827	CTNNBIP1	0.076607058	0.076663157
5828	PCGF3	0.272271995	1.51E-10
5829	MAPK13	-0.192993313	6.93E-06
5830	STAM2	-0.465952323	3.45E-30
5831	DHRS4L2	0.257576605	1.48E-09
5832	LURAP1L	-0.127296889	0.00318282
5833	TGIF1	0.285681056	1.66E-11
5834	HSBP1L1	0.317976152	4.92E-14
5835	PHF11	0.228962208	8.56E-08
5836	SLC16A2	-0.120965596	0.005083525
5837	GLIPR2	0.29352923	4.31E-12
5838	HABP4	-0.005917689	0.891380502
5839	FAM21C	0.022479832	0.603893647
5840	KRI1	0.678472745	2.08E-73
5841	CDK19	-0.371013827	6.66E-19
5842	UBE2V2	-0.244024946	1.08E-08
5843	METTL17	0.442449086	4.72E-27
5844	ZNF317	-0.181205346	2.48E-05
5845	RASIP1	0.016709609	0.699780111
5846	CASD1	-0.361805338	5.45E-18
5847	TIMM44	0.573082961	5.00E-48
5848	FNBP4	0.336061716	1.37E-15
5849	ANKZF1	0.468234515	1.66E-30
5850	YEATS4	-0.29438406	3.71E-12
5851	FXYD6	-0.041778764	0.334792041
5852	PLAC9	0.196266699	4.79E-06
5853	CDKN2C	0.221679527	2.22E-07
5854	GSE1	-0.112900258	0.008958239
5855	ARFGEF1	-0.482878995	1.33E-32
5856	ARHGAP4	0.555241621	1.35E-44

5857	DCDC2	-0.234498137	4.07E-08
5858	PRMT6	-0.290031591	7.90E-12
5859	TP53BP2	-0.152501864	0.000400349
5860	SPEN	-0.104744575	0.01536106
5861	TTC27	-0.157689973	0.000250466
5862	RNF122	0.244357205	1.03E-08
5863	ARHGEF40	0.092290633	0.032822135
5864	UQCC3	0.481795138	1.92E-32
5865	RPRD1B	-0.223715515	1.71E-07
5866	TMEM106A	0.042248838	0.329377596
5867	6-Sep	0.07168593	0.09765023
5868	CCDC3	-0.020971141	0.628398233
5869	MAP3K14	0.377251531	1.54E-19
5870	UTP11L	0.228687507	8.88E-08
5871	IMPA1	-0.183470612	1.95E-05
5872	CLIC2	-0.146407117	0.000681772
5873	NOL6	0.321632205	2.43E-14
5874	STAT5A	0.212426401	7.10E-07
5875	SCD5	-0.253522999	2.72E-09
5876	TCF7L2	-0.24324121	1.21E-08
5877	SAP130	-0.104221506	0.015883452
5878	MAP2K4	-0.339655623	6.51E-16
5879	TTC7A	0.39797708	9.48E-22
5880	ZNF770	-0.581109292	1.22E-49
5881	PANK3	-0.537497022	2.21E-41
5882	DOCK8	-0.234475785	4.08E-08
5883	ABR	0.000364501	0.993288899
5884	N4BP1	-0.321107893	2.69E-14
5885	PPM1F	0.014690907	0.734590757
5886	KRAS	-0.434645016	4.60E-26
5887	MACF1	-0.361946033	5.28E-18
5888	TMEM171	-0.051341967	0.235801037
5889	C3orf17	-0.210000518	9.55E-07
5890	ATG4B	0.69322895	7.68E-78
5891	MCU	-0.204903542	1.76E-06
5892	LEMD3	-0.563408833	3.86E-46
5893	OTUD4	-0.332625534	2.75E-15
5894	FAM189B	0.430865365	1.36E-25
5895	SPX	-0.217980213	3.55E-07
5896	STAMBP	-0.225213298	1.40E-07
5897	TCF19	0.229297798	8.19E-08
5898	MIS12	0.076464434	0.077215085
5899	NPC1	-0.051712996	0.232428506
5900	MAP3K1	-0.353428577	3.48E-17
5901	IFRD2	0.401128254	4.23E-22
5902	SLC9A1	0.105443533	0.014686576
5903	LST1	0.441106596	7.02E-27
5904	DUSP22	-0.011882467	0.783923941
5905	NIPAL3	-0.479115506	4.70E-32
5906	TUFT1	-0.117553002	0.006486993
5907	MCM5	0.378337468	1.19E-19
5908	SMARCE1	-0.078596745	0.069292679
5909	PI4KA	-0.221971901	2.14E-07
5910	DEF8	0.369384585	9.71E-19
5911	DIS3L	-0.396238279	1.47E-21
5912	ZNF706	0.374547321	2.92E-19
5913	MED13	-0.480050273	3.44E-32
5914	MORC2	0.3310973	3.74E-15

5915	EIF2B2	0.170549748	7.35E-05
5916	MRAS	-0.212150185	7.34E-07
5917	ROCK1	-0.422472153	1.43E-24
5918	TBC1D2B	-0.167907027	9.53E-05
5919	SLC7A2	-0.143085285	0.00090365
5920	ADCK4	0.478438447	5.89E-32
5921	SLFN5	-0.234136534	4.27E-08
5922	GRASP	0.226584965	1.17E-07
5923	NDUFV3	0.303797954	6.93E-13
5924	SLC4A1AP	-0.044978744	0.299058921
5925	FHL2	0.144701373	0.000788481
5926	ELMO2	0.123149827	0.004335402
5927	COQ7	-0.357548947	1.41E-17
5928	RPRD1A	-0.442148971	5.16E-27
5929	SOX13	-0.038762608	0.370882797
5930	TELO2	0.707317738	2.48E-82
5931	SENP6	-0.190775243	8.86E-06
5932	TMEM72	-0.100523512	0.020041475
5933	SACM1L	-0.400993288	4.38E-22
5934	WDR72	-0.380511351	7.09E-20
5935	PLS1	-0.345603427	1.87E-16
5936	ANKRD52	0.133078087	0.002038015
5937	ST6GALNAC4	0.244618776	9.92E-09
5938	POLR1C	0.559770016	1.90E-45
5939	NUP50	-0.129384232	0.002715099
5940	ATG4A	-0.334211241	1.99E-15
5941	SMOC2	-0.068059903	0.115864383
5942	CFL2	-0.323407894	1.72E-14
5943	NANS	0.454661229	1.19E-28
5944	CDK13	-0.157593674	0.00025269
5945	HMHA1	0.444206716	2.81E-27
5946	TOPORS	-0.381544462	5.54E-20
5947	ZMYM3	-0.011859085	0.784338631
5948	THAP5	-0.472282758	4.48E-31
5949	ARRDC1	0.582369799	6.73E-50
5950	LARP4	-0.322641905	2.00E-14
5951	NAGK	0.446538563	1.40E-27
5952	ALDH5A1	-0.303941512	6.75E-13
5953	ZHX3	-0.278341104	5.64E-11
5954	PPP2R5C	-0.374093783	3.25E-19
5955	ZRSR2	0.429327791	2.10E-25
5956	MNDA	-0.092891843	0.031696624
5957	SIPA1L1	-0.093562631	0.030479972
5958	MKKS	-0.3056069	4.98E-13
5959	MIER1	-0.324403647	1.41E-14
5960	ENTPD1	-0.155035001	0.000318992
5961	ITPRIPL2	-0.359603408	8.92E-18
5962	CDK17	-0.183594952	1.93E-05
5963	RNMT	-0.421335989	1.95E-24
5964	UBR5	-0.316187745	6.92E-14
5965	ASF1A	-0.329943562	4.72E-15
5966	FOXO1	-0.470879637	7.07E-31
5967	ZEB1	-0.223840897	1.68E-07
5968	ZNF302	0.108508833	0.01202652
5969	SETD5	0.10159858	0.018744654
5970	MRGBP	0.645126082	2.75E-64
5971	SCYL2	-0.297555436	2.12E-12
5972	C19orf66	0.629671102	1.96E-60

5973	CSRP2	0.06085467	0.159847907
5974	TMEM55B	0.251249983	3.80E-09
5975	DNALI1	-0.057256002	0.186060776
5976	PTPRS	0.159555037	0.000210847
5977	MRPL13	-0.099269258	0.021652521
5978	DROSHA	-0.121860243	0.004764057
5979	NFYB	-0.102199742	0.018051892
5980	ARMC6	0.531708852	2.25E-40
5981	SLC43A3	0.173733134	5.35E-05
5982	UTP14C	-0.514479361	1.74E-37
5983	C6orf203	-0.033817559	0.435040057
5984	CAP2	-0.212369656	7.15E-07
5985	NCOA2	-0.497336563	8.96E-35
5986	SESN1	-0.357602587	1.39E-17
5987	PDSS2	-0.57182741	8.87E-48
5988	BTD	-0.427633095	3.38E-25
5989	MATN2	-0.075201134	0.082246022
5990	ASRGL1	-0.022669161	0.600848975
5991	RANBP6	-0.345466599	1.93E-16
5992	ING2	-0.138377448	0.001333632
5993	TNFRSF10D	-0.1237045	0.004162001
5994	SNAPC5	0.168153392	9.30E-05
5995	DNAJC30	0.159789735	0.0002063
5996	SIDT2	0.086682731	0.045063499
5997	BRD7	-0.267956129	3.00E-10
5998	ROGDI	0.406665499	1.01E-22
5999	SGMS1	-0.556248416	8.77E-45
6000	GPR34	-0.278012021	5.96E-11
6001	NT5DC1	-0.610399188	6.30E-56
6002	INIP	-0.483597303	1.04E-32
6003	TSHZ1	-0.221456049	2.28E-07
6004	MYBBP1A	0.477585589	7.82E-32
6005	LTBP4	0.196271479	4.79E-06
6006	TGFBRAP1	-0.30043358	1.27E-12
6007	SS18L2	0.445514675	1.90E-27
6008	CHCHD4	-0.203247775	2.14E-06
6009	MED14	-0.351075324	5.80E-17
6010	MOB4	-0.396784651	1.28E-21
6011	SH3RF1	-0.425780951	5.69E-25
6012	RHOT1	-0.321407693	2.54E-14
6013	VPS37A	-0.292444021	5.21E-12
6014	BYSL	0.371251658	6.30E-19
6015	DMAP1	0.469472966	1.11E-30
6016	AMOTL1	-0.449052527	6.57E-28
6017	CCDC130	0.695924854	1.11E-78
6018	TRIM27	0.241926912	1.45E-08
6019	CUL2	-0.311301817	1.74E-13
6020	ASH1L	-0.44073485	7.83E-27
6021	TBC1D5	-0.514402008	1.79E-37
6022	RABL3	-0.501758713	1.85E-35
6023	RAB3IL1	0.371551558	5.88E-19
6024	LTBP2	-0.072206548	0.095233526
6025	TRUB2	0.163859463	0.000140676
6026	BBS10	-0.371760056	5.60E-19
6027	SGMS2	-0.510086344	8.90E-37
6028	MSANTD3	0.314501241	9.53E-14
6029	TAF1C	0.561263435	9.91E-46
6030	ZHX1	-0.479052427	4.80E-32

6031	DOCK9	-0.338113658	8.96E-16
6032	PPIL3	0.173893271	5.26E-05
6033	SNAPC2	0.512549782	3.57E-37
6034	SLC25A32	-0.168274543	9.19E-05
6035	SEC23IP	-0.388987315	9.03E-21
6036	BMP1	0.435714144	3.38E-26
6037	FHOD1	0.440879339	7.50E-27
6038	DPF2	0.072106628	0.09569361
6039	PPP2R2A	-0.341162627	4.76E-16
6040	PRKAR1B	0.209342568	1.03E-06
6041	METTL21B	-0.188998042	1.08E-05
6042	TAP2	0.178115829	3.42E-05
6043	GRPEL2	-0.08872218	0.040228305
6044	IL27RA	0.414823941	1.15E-23
6045	ZNF148	-0.563398216	3.87E-46
6046	SAMD9L	0.00069296	0.987241794
6047	TCF20	-0.123156632	0.004333236
6048	ABI3BP	-0.269306245	2.42E-10
6049	USP25	-0.375660302	2.25E-19
6050	PTRHD1	0.423720292	1.01E-24
6051	NUP214	-0.005010642	0.907948856
6052	MLXIP	0.246368753	7.72E-09
6053	GAS2L1	0.343416636	2.97E-16
6054	TMEM87B	-0.192577926	7.26E-06
6055	TMEM11	0.514539095	1.70E-37
6056	ARMC7	0.420581849	2.40E-24
6057	TRAF3IP2	-0.071857421	0.096848828
6058	CYTH3	-0.143578383	0.00086696
6059	SLC33A1	-0.282390332	2.89E-11
6060	PAN2	0.319640478	3.57E-14
6061	TRAM2	-0.086444106	0.045659924
6062	MCUR1	-0.275477053	9.01E-11
6063	LRBA	-0.627235705	7.56E-60
6064	ANXA9	-0.123388614	0.004259968
6065	KDELC1	0.092551067	0.032330461
6066	JAG2	0.123738619	0.004151543
6067	L3MBTL2	0.248557377	5.63E-09
6068	RILPL2	0.063285508	0.143785087
6069	PLEK2	0.029345056	0.498208402
6070	ENPP5	-0.536830743	2.90E-41
6071	LUZP1	-0.466659504	2.75E-30
6072	SLC30A7	-0.315373649	8.08E-14
6073	FBXW2	-0.38940239	8.15E-21
6074	LACTB	-0.072062966	0.095895212
6075	PPWD1	0.052637977	0.224169737
6076	MPHOSPH10	0.30384179	6.87E-13
6077	ZC3H3	0.654205945	1.18E-66
6078	DLG1	-0.321546488	2.47E-14
6079	LRRC23	0.323581397	1.66E-14
6080	SUV420H1	-0.30215089	9.33E-13
6081	IGSF3	-0.376588264	1.81E-19
6082	SNRNP40	-0.058464502	0.176926163
6083	ECM1	0.290114878	7.79E-12
6084	ACKR1	0.042916014	0.321790557
6085	ENOSF1	0.177192847	3.76E-05
6086	DDX46	-0.311570395	1.65E-13
6087	SLC35A3	-0.49707427	9.83E-35
6088	NHLRC3	-0.187102012	1.32E-05

6089	SIN3B	0.418955929	3.75E-24
6090	DENND4B	0.543532738	1.88E-42
6091	LMNB2	0.464755525	5.06E-30
6092	RBKS	0.045276843	0.295864448
6093	SIPA1L2	-0.272231031	1.52E-10
6094	RNF2	-0.246829466	7.23E-09
6095	PSPH	0.004455979	0.918100652
6096	RAB42	-0.004854555	0.910804219
6097	CBX4	0.37199283	5.31E-19
6098	SMARCAD1	-0.433278623	6.81E-26
6099	C11orf1	-0.014152331	0.743974175
6100	RASSF6	-0.114978365	0.007766159
6101	ZFYVE19	0.294723917	3.50E-12
6102	SPTY2D1	-0.589822799	1.92E-51
6103	TRPV2	0.238412916	2.37E-08
6104	CELSR2	-0.176294234	4.13E-05
6105	CPXM2	-0.274746148	1.01E-10
6106	HES4	0.533141788	1.27E-40
6107	NCDN	0.274179713	1.11E-10
6108	FAM111A	0.253970553	2.54E-09
6109	GPR126	-0.123145217	0.00433687
6110	MAPK9	-0.429162908	2.20E-25
6111	POMGNT2	-0.179189773	3.06E-05
6112	MED25	0.614314935	8.10E-57
6113	IQGAP2	-0.471561201	5.66E-31
6114	SPPL2B	0.620066493	3.78E-58
6115	DCTN5	-0.302979575	8.04E-13
6116	YIF1B	0.570230629	1.83E-47
6117	TRAPPC6B	-0.270687447	1.95E-10
6118	CD300A	0.141735484	0.001011554
6119	SLC15A3	0.223016333	1.87E-07
6120	DHCR7	-0.114714917	0.007908993
6121	BCS1L	0.662640736	6.24E-69
6122	FAM160B1	-0.324536401	1.38E-14
6123	MRS2	-0.247951465	6.15E-09
6124	LMOD1	-0.027999611	0.518120827
6125	SP100	-0.013807353	0.750004676
6126	GCA	-0.193976105	6.21E-06
6127	KLF7	-0.24728093	6.77E-09
6128	ZFYVE1	-0.339159506	7.22E-16
6129	RALGAPB	-0.430471508	1.52E-25
6130	WIZ	0.418894268	3.81E-24
6131	9-Mar	0.231447482	6.14E-08
6132	ARAP3	0.128174005	0.002978015
6133	MRPS30	-0.460706715	1.82E-29
6134	SLC22A12	-0.003906419	0.928172195
6135	TERF2	-0.181552769	2.39E-05
6136	ARHGAP17	0.170768592	7.19E-05
6137	KHNYN	-0.069827843	0.106676156
6138	AEBP2	-0.349504341	8.13E-17
6139	SMTNL2	0.026280651	0.544145485
6140	IL10RA	0.195239214	5.39E-06
6141	PIGV	-0.225664304	1.32E-07
6142	SSPN	-0.30786819	3.29E-13
6143	CAPN15	0.557793302	4.50E-45
6144	MCOLN1	0.319226386	3.87E-14
6145	GGT5	0.155590718	0.000303342
6146	PRKAG2	-0.190146925	9.50E-06

6147	NRARP	-0.012478656	0.77337104
6148	RYK	-0.244214298	1.05E-08
6149	PITPNC1	-0.050488979	0.243684956
6150	KCNQ1	-0.182109654	2.26E-05
6151	FYB	-0.07071742	0.102275877
6152	MAEA	0.182516681	2.16E-05
6153	POFUT2	0.43808699	1.70E-26
6154	DR1	-0.34374742	2.77E-16
6155	CRTC3	-0.202425273	2.36E-06
6156	ZCCHC10	-0.114506235	0.008023795
6157	SLC41A3	-0.040063404	0.355031905
6158	ANAPC7	0.617064416	1.89E-57
6159	PSMG1	0.123479455	0.004231584
6160	JAZF1	-0.311899267	1.56E-13
6161	PLD2	0.397412997	1.09E-21
6162	IER5	0.244387635	1.03E-08
6163	ZNF513	0.640575893	3.95E-63
6164	SOX12	0.452489196	2.31E-28
6165	HIRIP3	0.289763877	8.27E-12
6166	RASSF1	0.525160126	2.95E-39
6167	CYTH1	0.210327326	9.18E-07
6168	AP1S2	-0.326493758	9.37E-15
6169	MANBA	-0.367083649	1.65E-18
6170	C7orf55-LUC7L2	0.155506296	0.000305673
6171	STK17A	-0.102330784	0.017903886
6172	GUF1	-0.421841613	1.70E-24
6173	UQCC1	0.101424901	0.018949065
6174	C19orf12	0.211732952	7.73E-07
6175	PITPNM1	0.245659288	8.55E-09
6176	RNASEH2A	0.538614317	1.41E-41
6177	LZTS1	0.032696283	0.450428933
6178	LMTK2	-0.342155547	3.87E-16
6179	UPF2	-0.210759902	8.71E-07
6180	VAC14	0.281221524	3.51E-11
6181	PNKP	0.686329457	9.80E-76
6182	MMP24	-0.297721141	2.06E-12
6183	SGSH	0.520843937	1.56E-38
6184	ZNF185	-0.224143442	1.61E-07
6185	CYB561D2	0.289313127	8.94E-12
6186	RAB3D	-0.096525414	0.025573177
6187	ABCB1	-0.158184276	0.000239337
6188	IL17RC	0.313952739	1.06E-13
6189	ITGA2	-0.415054631	1.08E-23
6190	FAM177A1	-0.149466762	0.000523191
6191	MTURN	-0.359831974	8.48E-18
6192	WDR11	-0.344045436	2.60E-16
6193	GPD1L	-0.422829081	1.29E-24
6194	WAS	0.451207351	3.42E-28
6195	PPP1R16A	0.531156704	2.80E-40
6196	ABCD1	0.468612687	1.47E-30
6197	GNG10	-0.285380803	1.75E-11
6198	TEX30	0.204508252	1.84E-06
6199	GOSR1	-0.392450905	3.82E-21
6200	FEN1	0.228718923	8.85E-08
6201	PSMD14	0.079353291	0.066647033
6202	CHERP	0.218761445	3.22E-07
6203	TLE3	0.147965533	0.00059614
6204	KDM3A	-0.067603752	0.118332976

6205	WWC2	-0.48307434	1.25E-32
6206	SBSPON	-0.077909831	0.071768742
6207	DIDO1	-0.038275032	0.376935321
6208	ETNK2	-0.029985783	0.488870414
6209	FOXJ2	-0.170812616	7.16E-05
6210	IQCB1	0.017408261	0.687873245
6211	YARS2	0.196936577	4.44E-06
6212	CLN5	-0.493324243	3.68E-34
6213	RIPK2	0.162167228	0.000165135
6214	ZNF394	0.330476355	4.24E-15
6215	TCTN1	0.017936886	0.678914916
6216	TBP	0.054727401	0.206289261
6217	RUSC1	0.503842238	8.73E-36
6218	CLCF1	0.313007501	1.26E-13
6219	SLC2A2	-0.044736081	0.301676231
6220	DPH5	-0.101921599	0.018369586
6221	FLAD1	0.630980887	9.40E-61
6222	PER2	-0.051828463	0.231385941
6223	MFN1	-0.212183066	7.31E-07
6224	CCDC137	0.727302683	3.50E-89
6225	ATG101	0.205156505	1.71E-06
6226	PLEKHA1	-0.132928348	0.002062139
6227	TEAD3	0.344902588	2.17E-16
6228	STK4	-0.086160975	0.04637618
6229	FAM193B	0.565052556	1.86E-46
6230	FAM129A	-0.164802939	0.00012856
6231	FAM107A	-0.099805203	0.020950856
6232	CXorf38	0.044528253	0.303929859
6233	IREB2	-0.535999805	4.05E-41
6234	MOB2	0.361693791	5.59E-18
6235	KIAA1715	-0.419872578	2.92E-24
6236	ARHGAP30	0.118029692	0.006272056
6237	DCAF16	-0.161377626	0.000177865
6238	ADAP2	0.079681726	0.065524607
6239	FAM188A	-0.360875486	6.71E-18
6240	MAP4K3	-0.36378089	3.49E-18
6241	FGFR3	0.02517577	0.561208554
6242	MRPS28	-0.191957968	7.78E-06
6243	CCNT1	-0.374485434	2.96E-19
6244	IMMP2L	-0.318059374	4.84E-14
6245	TRRAP	-0.261931092	7.65E-10
6246	PURA	-0.531518843	2.43E-40
6247	PDE9A	0.041047817	0.343324221
6248	SH3BP5L	0.29056354	7.21E-12
6249	ZBED3	-0.250679481	4.13E-09
6250	PDCD7	0.037679775	0.384406363
6251	UBN1	-0.100822634	0.019673038
6252	SETDB1	0.132432072	0.002143972
6253	ALDH16A1	0.507247525	2.53E-36
6254	TNFRSF19	-0.239820983	1.95E-08
6255	BBS4	0.0235827	0.586256179
6256	SMOX	0.271406227	1.73E-10
6257	REEP4	0.592088895	6.38E-52
6258	GRWD1	0.254910929	2.21E-09
6259	KIAA0907	0.467335057	2.22E-30
6260	GCLM	-0.430914444	1.34E-25
6261	LHFPL2	-0.068062625	0.115849774
6262	LCP2	0.1184524	0.00608683

6263	NUDT19	0.02038559	0.638022066
6264	ANKH	-0.162880383	0.000154377
6265	NCBP1	-0.281347835	3.43E-11
6266	SUZ12	-0.384997956	2.40E-20
6267	SNRPD1	0.249636383	4.81E-09
6268	ARIH2	-0.021226435	0.624221863
6269	TLK1	-0.349996915	7.32E-17
6270	DNAJC15	0.003837005	0.929445165
6271	PTGER3	-0.18873442	1.11E-05
6272	PIGH	-0.241175921	1.61E-08
6273	RAB22A	-0.229694249	7.77E-08
6274	SLC35D1	-0.236716801	3.00E-08
6275	BCL2L11	-0.347338095	1.29E-16
6276	LLGL2	0.178299601	3.36E-05
6277	PHF13	0.160061737	0.000201145
6278	KIF13B	-0.462643074	9.88E-30
6279	GFER	0.479401258	4.28E-32
6280	CCM2	0.627119699	8.06E-60
6281	HAUS1	0.143868794	0.000846
6282	POLL	0.224603184	1.52E-07
6283	NUDT22	0.400070256	5.56E-22
6284	PIGO	-0.247847415	6.24E-09
6285	CHST2	0.07391957	0.087617243
6286	STK10	0.112387335	0.009276469
6287	COIL	-0.034948185	0.419833809
6288	MAN2A2	0.093799456	0.030060088
6289	SMAD4	-0.402738167	2.80E-22
6290	PPM1B	-0.284471103	2.04E-11
6291	ATP2A3	0.190693638	8.94E-06
6292	NXPH4	0.111969598	0.009543035
6293	NUP88	0.010482743	0.808852936
6294	ATP10D	-0.560674365	1.28E-45
6295	ELMOD2	-0.490200922	1.09E-33
6296	TRAPPC9	-0.023462316	0.588169781
6297	SIRT3	0.182414127	2.19E-05
6298	S1PR3	-0.031492742	0.467283684
6299	ERCC3	0.344901122	2.17E-16
6300	MTMR4	-0.048845778	0.259388829
6301	SHB	0.421315831	1.96E-24
6302	BMS1	0.005969973	0.890426836
6303	RAB38	-0.280466787	3.97E-11
6304	ZNF629	-0.173152977	5.67E-05
6305	HDHD2	-0.455605103	8.89E-29
6306	SLC29A4	-0.006538301	0.880070989
6307	PDZD8	-0.501247895	2.22E-35
6308	ELK4	-0.261139472	8.64E-10
6309	SLC25A17	-0.192993212	6.93E-06
6310	TUBGCP6	0.459229295	2.89E-29
6311	CHD2	-0.042664128	0.324641495
6312	PDCD2	-0.12969524	0.00265104
6313	MTUS1	-0.425594418	5.99E-25
6314	FASTKD3	-0.162394589	0.000161632
6315	POLD4	0.504761382	6.25E-36
6316	TMCO3	-0.397848461	9.80E-22
6317	MOB3B	-0.326744128	8.92E-15
6318	GPATCH8	-0.159490038	0.000212123
6319	PIAS3	0.189840243	9.82E-06
6320	SEC22B	-0.382542034	4.35E-20

6321	SVIP	-0.436844152	2.44E-26
6322	POLH	-0.168379778	9.10E-05
6323	ARHGAP21	-0.31872865	4.26E-14
6324	CDK2	0.20204352	2.46E-06
6325	NPY1R	-0.12470815	0.003864114
6326	MSR1	-0.137925672	0.001383529
6327	STK17B	-0.088360741	0.041051745
6328	SLC39A10	-0.214263247	5.66E-07
6329	CDK11B	0.511093388	6.13E-37
6330	FAM53C	-0.143746494	0.000854769
6331	KIAA1429	-0.464464971	5.55E-30
6332	SPOPL	-0.577494488	6.58E-49
6333	TRIM35	-0.10383559	0.016278795
6334	GEMIN5	-0.320445181	3.06E-14
6335	SMIM6	0.017834072	0.680653765
6336	TSEN54	0.633779301	1.94E-61
6337	TBC1D8	0.028068804	0.517086872
6338	ENC1	-0.100778319	0.019727246
6339	ETV6	0.150962227	0.000458844
6340	PAPD7	0.293747475	4.15E-12
6341	SETD2	-0.287733011	1.17E-11
6342	TMEM131	-0.353934851	3.11E-17
6343	POLR2D	0.104869953	0.015238107
6344	TSNAX	-0.354319535	2.86E-17
6345	ST7	-0.074661252	0.084475373
6346	EXT1	-0.086252085	0.046144671
6347	COMMD4	0.584417772	2.56E-50
6348	NIPBL	-0.372048527	5.24E-19
6349	RAB24	0.570464598	1.65E-47
6350	NEDD4L	-0.318314285	4.61E-14
6351	RBPMS2	-0.078421037	0.069919283
6352	PIGX	-0.151038738	0.000455759
6353	GPATCH3	0.287589221	1.20E-11
6354	ARHGEF16	0.076022914	0.078944176
6355	GAB2	-0.322051162	2.24E-14
6356	SCAMP5	-0.134748009	0.001785937
6357	GATC	-0.258542987	1.28E-09
6358	DTX3	0.210439347	9.05E-07
6359	UBXN11	0.595315088	1.31E-52
6360	FAM63B	-0.536503143	3.31E-41
6361	SLC27A3	0.462747657	9.56E-30
6362	ZSCAN26	-0.054378527	0.209200695
6363	ZNF691	0.334457041	1.90E-15
6364	ARHGEF11	0.110273619	0.010696552
6365	CHST11	0.114011798	0.00830176
6366	DUSP12	0.264999636	4.76E-10
6367	TWISTNB	-0.460227002	2.11E-29
6368	ADK	-0.2895612	8.57E-12
6369	GMPS	-0.305205619	5.36E-13
6370	ZNF316	0.448097902	8.76E-28
6371	TNFAIP8L1	0.200368824	3.00E-06
6372	SNX8	0.311972003	1.53E-13
6373	SCAF4	0.12855892	0.002891984
6374	PDE6D	0.336660099	1.21E-15
6375	REST	-0.527119572	1.37E-39
6376	SLC25A37	0.387406577	1.33E-20
6377	HIST2H2BE	0.237761591	2.60E-08
6378	PRODH2	0.2724611	1.47E-10

6379	SH3YL1	-0.160457755	0.000193855
6380	SUPV3L1	0.14861399	0.000563542
6381	P2RY8	-0.102856214	0.017321033
6382	EARS2	-0.260588999	9.39E-10
6383	RPUSD1	0.645279948	2.51E-64
6384	CCNH	0.057426084	0.184754574
6385	PRKX	-0.158398821	0.000234652
6386	DPY19L4	-0.45837029	3.77E-29
6387	NPL	0.019842033	0.647010514
6388	ZNF277	-0.424636694	7.82E-25
6389	MED1	-0.400119037	5.49E-22
6390	HMGCR	-0.508944159	1.36E-36
6391	GORASP1	0.024399211	0.573353709
6392	TDRD7	-0.261774647	7.84E-10
6393	NRN1	-0.034430693	0.426754767
6394	SCARF1	0.067177591	0.120676261
6395	ZMAT3	-0.43617544	2.96E-26
6396	UBR3	-0.513252974	2.75E-37
6397	ZNF384	-0.014642495	0.735432635
6398	C6orf136	0.332499812	2.82E-15
6399	EIF2B4	0.663381018	3.91E-69
6400	ACTR6	-0.412107855	2.38E-23
6401	NFS1	0.058283372	0.178273621
6402	PTAR1	-0.43272097	7.99E-26
6403	CREM	0.02160991	0.617971161
6404	CALHM2	0.169144562	8.44E-05
6405	ARHGEF3	-0.311628027	1.64E-13
6406	LRSAM1	0.438703607	1.42E-26
6407	FBXO9	-0.15183467	0.000424788
6408	POMT1	0.189538569	1.02E-05
6409	ALDH1L1	-0.203438292	2.09E-06
6410	KANSL1L	-0.16311401	0.000150999
6411	KRIT1	-0.148156157	0.00058638
6412	DENND6A	-0.33165892	3.34E-15
6413	F2RL3	-0.061153061	0.15780626
6414	ISCA2	-0.190402747	9.23E-06
6415	ZFP62	-0.161150531	0.000181694
6416	TTC31	0.399473583	6.47E-22
6417	NOXA1	0.4627272	9.62E-30
6418	SPRYD7	-0.186930406	1.35E-05
6419	RCC1	0.315825622	7.42E-14
6420	JRKL	-0.056690533	0.190452419
6421	MORC3	-0.310760932	1.92E-13
6422	BRAP	-0.293389309	4.42E-12
6423	SCFD2	-0.349174788	8.73E-17
6424	COMMD3	0.30348563	7.33E-13
6425	MFAP3	-0.554943302	1.54E-44
6426	GART	-0.014603031	0.736119137
6427	MARK4	0.296472764	2.57E-12
6428	ADAMTS4	0.110010529	0.01088616
6429	WDR61	-0.129078921	0.002779353
6430	AVPI1	0.183030469	2.05E-05
6431	UBE4B	-0.411756388	2.62E-23
6432	ARHGAP42	-0.400027406	5.62E-22
6433	MINOS1	0.099048212	0.021947807
6434	ITSN2	-0.318651638	4.32E-14
6435	SIKE1	-0.282420844	2.87E-11
6436	EMP2	-0.012786587	0.767936654

6437	MAP3K7	-0.246570002	7.50E-09
6438	TLR2	0.0041517	0.923675531
6439	USP36	0.234922414	3.84E-08
6440	SLC8B1	0.293935002	4.02E-12
6441	NSD1	-0.337556622	1.01E-15
6442	TRAK1	-0.28205864	3.05E-11
6443	PPIL2	0.320996664	2.75E-14
6444	CCDC59	0.088539118	0.040643598
6445	TGFBR3	-0.329588794	5.07E-15
6446	COL12A1	-0.094675588	0.028549552
6447	UNK	0.304892582	5.68E-13
6448	C1orf210	-0.216919942	4.06E-07
6449	APTX	0.204797312	1.78E-06
6450	NQO2	0.284920502	1.89E-11
6451	CCDC69	-0.074478965	0.085239011
6452	MOB1B	-0.397023464	1.21E-21
6453	MYO18A	-0.197781109	4.03E-06
6454	CUEDC1	0.306387611	4.32E-13
6455	NAPG	-0.386445476	1.69E-20
6456	ENTPD5	-0.176991441	3.84E-05
6457	ACOT9	0.248931505	5.33E-09
6458	PRPSAP2	-0.035909424	0.407154188
6459	QPCT	-0.128382051	0.002931231
6460	TFIP11	-0.219827465	2.81E-07
6461	PTDSS2	0.452538515	2.28E-28
6462	SERGEF	0.049180858	0.256131084
6463	VEGFC	0.051687811	0.232656341
6464	DPH2	0.266990456	3.49E-10
6465	ADCY9	-0.236083767	3.27E-08
6466	NUP188	0.210027019	9.52E-07
6467	SUFU	-0.137320185	0.001453096
6468	STS	-0.380264554	7.53E-20
6469	CCNYL1	-0.232792502	5.12E-08
6470	FYCO1	-0.454756739	1.15E-28
6471	C1GALT1	-0.287237003	1.27E-11
6472	ZBTB2	-0.101200207	0.019216402
6473	ZNF787	0.645427258	2.30E-64
6474	MAU2	0.25768075	1.46E-09
6475	MALT1	-0.108475964	0.012052619
6476	SUGP2	0.189102393	1.07E-05
6477	CLEC11A	0.328824034	5.90E-15
6478	ZNHIT2	0.509199898	1.23E-36
6479	TBL3	0.506018729	3.96E-36
6480	NUSAP1	0.206560976	1.45E-06
6481	CWC27	-0.313494962	1.15E-13
6482	ABHD17B	-0.360496058	7.31E-18
6483	PSEN2	0.10692082	0.013346094
6484	AP1M2	0.026040839	0.547827032
6485	NOP16	0.588330267	3.94E-51
6486	PLCB3	0.602293886	4.02E-54
6487	MR1	-0.377894917	1.32E-19
6488	LSM5	0.085711023	0.047533848
6489	ACD	0.573936543	3.39E-48
6490	DXO	0.594915949	1.60E-52
6491	MAFG	0.044303902	0.306375155
6492	AEN	0.132454075	0.002140281
6493	USP48	-0.150923013	0.000460433
6494	EPC2	-0.396511061	1.38E-21

6495	C19orf52	0.430596552	1.46E-25
6496	THBS3	0.533114784	1.29E-40
6497	KIF22	0.640001617	5.51E-63
6498	RINT1	-0.124739987	0.003854989
6499	CTBS	-0.489062849	1.61E-33
6500	SRD5A3	0.077901886	0.071797796
6501	GTF2H3	-0.257325056	1.54E-09
6502	PGAP2	0.296664928	2.49E-12
6503	DAK	0.338653967	8.02E-16
6504	ST3GAL4	-0.012282369	0.776840929
6505	MARK3	0.262392575	7.13E-10
6506	MMAB	0.124074121	0.004049961
6507	CEP120	-0.332486245	2.83E-15
6508	STK11	0.530684823	3.38E-40
6509	FEZ2	-0.426965674	4.08E-25
6510	CABLES1	-0.07412442	0.086740219
6511	REXO1	0.63446481	1.32E-61
6512	CCDC90B	-0.275509335	8.96E-11
6513	ADCY3	-0.022266038	0.607340039
6514	KCNS3	0.016628712	0.701163652
6515	FASN	0.272123009	1.55E-10
6516	UTP6	0.415309324	1.01E-23
6517	CCL20	0.328432773	6.38E-15
6518	WDR41	-0.180371333	2.71E-05
6519	SAYSD1	0.00249809	0.954030666
6520	NUDT18	0.280139775	4.19E-11
6521	AHDC1	0.252612173	3.11E-09
6522	C3orf58	-0.261772289	7.84E-10
6523	TGFB1I1	0.236694185	3.01E-08
6524	EID2	0.13716103	0.001471908
6525	SULT1C2	0.054403199	0.208993827
6526	DPYD	-0.293905442	4.04E-12
6527	EIF4ENIF1	-0.031190144	0.471575561
6528	C9orf89	0.588966986	2.90E-51
6529	GLCE	-0.421217647	2.02E-24
6530	SECTM1	0.320377261	3.10E-14
6531	GPD2	-0.488883739	1.72E-33
6532	PPARG	-0.197603879	4.12E-06
6533	CC2D1B	0.530792755	3.24E-40
6534	MED19	0.323974389	1.54E-14
6535	INO80	-0.229152612	8.35E-08
6536	ZPR1	0.125169874	0.003733672
6537	NACC2	-0.13857148	0.001312715
6538	TMEM99	0.058765161	0.174706235
6539	ANKRD13C	-0.356844453	1.64E-17
6540	OGFOD1	-0.139164083	0.001250685
6541	ARG2	-0.080778573	0.061888219
6542	PTPRU	-0.11737256	0.006570062
6543	SLC35B4	-0.356494659	1.78E-17
6544	TRIM55	-0.13451659	0.001819075
6545	EVI2A	0.075342666	0.081669529
6546	FRG1B	-0.358587253	1.12E-17
6547	RBM5	0.295613903	2.99E-12
6548	TLN2	-0.205748195	1.59E-06
6549	ABCD4	0.315125129	8.47E-14
6550	BBS2	-0.290011817	7.93E-12
6551	CHST3	-0.19653363	4.65E-06
6552	USP40	0.183157359	2.02E-05

6553	PIGC	0.254748176	2.27E-09
6554	FBXL12	0.475678141	1.47E-31
6555	AMOT	-0.527336978	1.26E-39
6556	PDCD11	0.073260473	0.090487536
6557	SMNDC1	-0.150201872	0.000490579
6558	ASB6	0.497842252	7.49E-35
6559	PARP6	0.496452446	1.22E-34
6560	MIEF2	0.068856374	0.111651016
6561	GSTM4	0.09536271	0.027410943
6562	RRP1	0.590759827	1.22E-51
6563	NATD1	-0.313767383	1.10E-13
6564	RASL12	0.076290274	0.077893423
6565	NOP2	0.724852466	2.61E-88
6566	CCL4	0.222461158	2.01E-07
6567	ICAM2	0.039901555	0.356980567
6568	DNAJC14	-0.33049347	4.23E-15
6569	CD37	0.346224743	1.64E-16
6570	DDIT4L	-0.289694507	8.37E-12
6571	PM20D2	-0.294134954	3.88E-12
6572	SH3TC1	0.156735869	0.000273329
6573	PTPN3	-0.326962735	8.54E-15
6574	USP21	0.57242029	6.77E-48
6575	ST8SIA4	-0.213178208	6.47E-07
6576	NGFR	-0.079941172	0.064648976
6577	ZNF687	-0.082158533	0.057551443
6578	CRMP1	0.049604512	0.252052917
6579	GRK6	0.501623035	1.94E-35
6580	IER5L	0.485467435	5.53E-33
6581	CUL3	-0.41061764	3.54E-23
6582	PPP2R1B	-0.247793773	6.29E-09
6583	PEX10	-0.036596412	0.398233356
6584	ESF1	-0.232634297	5.24E-08
6585	MESDC1	0.301690817	1.01E-12
6586	TMEM55A	-0.37209394	5.18E-19
6587	FOLH1	-0.12127114	0.00497231
6588	CYLD	-0.252286721	3.26E-09
6589	NICN1	0.147058484	0.00064468
6590	SKAP1	0.055907328	0.196660125
6591	RRAGC	-0.045119509	0.297547616
6592	FAM13B	-0.402163668	3.24E-22
6593	SLC17A4	-0.023751116	0.583583909
6594	SMCR8	-0.388685909	9.72E-21
6595	LIF	0.281199107	3.52E-11
6596	NDUFAF4	-0.2766209	7.48E-11
6597	ERG	-0.214409423	5.56E-07
6598	STK24	-0.428303913	2.80E-25
6599	SIN3A	-0.335567584	1.51E-15
6600	CNOT3	0.490609466	9.45E-34
6601	P3H1	0.499300085	4.46E-35
6602	KAT6A	-0.406361721	1.09E-22
6603	MIB1	-0.529918176	4.58E-40
6604	CTSW	0.362129405	5.07E-18
6605	SC5D	-0.504862363	6.03E-36
6606	GZMK	0.216438892	4.31E-07
6607	CBR4	-0.287817974	1.15E-11
6608	C18orf21	0.139367017	0.001230071
6609	HIVEP2	-0.286859114	1.36E-11
6610	RGS16	0.033254161	0.442734238

6611	EDC4	0.12899715	0.002796795
6612	C19orf54	0.484816593	6.90E-33
6613	SLC44A4	-0.227218535	1.08E-07
6614	RRP1B	0.007783185	0.857460916
6615	WARS2	-0.393508427	2.93E-21
6616	NDUFS7	0.430648453	1.44E-25
6617	MED18	0.191566201	8.12E-06
6618	HEBP2	0.119579086	0.005616895
6619	GPR176	-0.100858394	0.019629389
6620	NUPL1	-0.148696582	0.000559512
6621	FAXDC2	-0.242517574	1.34E-08
6622	FAM206A	-0.079452153	0.066307523
6623	MAPK11	0.556921267	6.56E-45
6624	FBXL14	-0.240640026	1.74E-08
6625	HIBCH	-0.265147639	4.65E-10
6626	ZNF532	-0.117323773	0.006592685
6627	FADD	0.095725213	0.02682615
6628	BMP2	-0.067436287	0.11924952
6629	EEF1E1	0.11420442	0.008192465
6630	CBLL1	-0.469680702	1.04E-30
6631	SNAPC3	-0.252061688	3.37E-09
6632	LENG1	0.383505198	3.45E-20
6633	MFSD3	0.36736338	1.54E-18
6634	ASCC1	-0.313395106	1.17E-13
6635	SUCO	-0.345188102	2.04E-16
6636	FAM210A	-0.363399365	3.81E-18
6637	CLEC2B	0.2388866	2.22E-08
6638	ANKRD11	0.078271075	0.070457731
6639	COMTD1	0.483296806	1.15E-32
6640	PCGF1	0.671638524	1.93E-71
6641	RUFY3	0.081040451	0.061045096
6642	FHL5	-0.296275531	2.66E-12
6643	RCOR1	-0.283619526	2.35E-11
6644	LRIG3	-0.310783932	1.92E-13
6645	CEP57	-0.32184173	2.33E-14
6646	RWDD2B	-0.00017871	0.996709602
6647	CD48	0.217536424	3.76E-07
6648	BIRC6	-0.387034144	1.46E-20
6649	ANTXR2	-0.191007352	8.64E-06
6650	COG3	-0.111497789	0.009852277
6651	SLC35C1	0.22366913	1.72E-07
6652	CD8A	0.238265726	2.42E-08
6653	BTAF1	0.029071664	0.502221434
6654	DHRS12	0.114941317	0.007786104
6655	MERTK	-0.35979707	8.54E-18
6656	MAFF	0.169586296	8.08E-05
6657	WDR48	-0.455146263	1.02E-28
6658	NT5DC2	0.311351566	1.72E-13
6659	NAA60	0.567353073	6.67E-47
6660	THG1L	0.004736462	0.912965302
6661	SH3PXD2A	-0.104720147	0.015385116
6662	CDS1	-0.492754208	4.49E-34
6663	KLF16	0.502525324	1.40E-35
6664	MRPS22	-0.080034195	0.06433738
6665	PEX11A	-0.251152518	3.86E-09
6666	SLC11A2	-0.128855172	0.002827317
6667	RILP	0.277288615	6.70E-11
6668	VGLL4	-0.432951357	7.48E-26

6669	DNAJC21	-0.332975492	2.56E-15
6670	RRAGB	-0.11794857	0.006308176
6671	GPCPD1	0.070614709	0.10277646
6672	DZIP3	-0.13407937	0.001883225
6673	RBM43	-0.391950622	4.33E-21
6674	RMND5B	0.101359704	0.0190263
6675	STK35	0.044896705	0.299942076
6676	RGN	-0.129609515	0.002668558
6677	MED27	0.304213582	6.42E-13
6678	CWF19L2	-0.389286686	8.38E-21
6679	ODF2	0.306518379	4.22E-13
6680	FITM2	-0.405091152	1.52E-22
6681	ABCB10	-0.275934068	8.36E-11
6682	RNF138	-0.221150759	2.37E-07
6683	DMXL1	-0.49906768	4.84E-35
6684	FMO4	-0.214151998	5.74E-07
6685	HSDL1	-0.356368727	1.83E-17
6686	C17orf80	-0.141202923	0.001057308
6687	ZFYVE9	-0.524936165	3.21E-39
6688	TAOK1	-0.427630776	3.39E-25
6689	SIRT6	0.752298618	1.16E-98
6690	PHF20	-0.373044612	4.15E-19
6691	PEG10	-0.145210388	0.000755122
6692	TMEM209	-0.323959056	1.54E-14
6693	ZNF503	-0.055790947	0.197595037
6694	ZW10	-0.408555291	6.12E-23
6695	BRD1	0.216553462	4.25E-07
6696	SEMA3G	-0.183733546	1.90E-05
6697	PCF11	0.01807439	0.676592035
6698	TYW3	-0.309369784	2.49E-13
6699	FKBP15	0.063017641	0.145492151
6700	ARFRP1	0.576019156	1.30E-48
6701	ME3	0.239408396	2.07E-08
6702	AHCYL2	-0.358504011	1.14E-17
6703	FANCL	0.189785333	9.88E-06
6704	ANKRA2	0.045514071	0.293338608
6705	GATAD2B	-0.114507151	0.008023288
6706	BID	0.470940667	6.93E-31
6707	GFM1	-0.410062164	4.11E-23
6708	LIN7A	-0.434544555	4.74E-26
6709	FBN1	-0.175035597	4.69E-05
6710	TMEM170A	-0.290282427	7.57E-12
6711	TRIM65	0.529036162	6.48E-40
6712	MSH2	-0.329120177	5.56E-15
6713	NCF2	0.025921391	0.549665339
6714	RAB11FIP2	-0.535754844	4.47E-41
6715	YEATS2	-0.025394053	0.557817158
6716	HKR1	0.148973092	0.00054621
6717	NCOA6	-0.174056849	5.18E-05
6718	NCF4	0.428397371	2.73E-25
6719	VTA1	-0.423069982	1.21E-24
6720	UFD1L	0.51640017	8.43E-38
6721	PALM	0.171772289	6.51E-05
6722	SMARCAL1	0.139677212	0.001199166
6723	SOCS6	-0.421211873	2.02E-24
6724	F8A1	0.335188614	1.63E-15
6725	TRAF2	0.636218018	4.86E-62
6726	CNST	-0.368473607	1.20E-18

6727	DLG3	-0.187387095	1.28E-05
6728	TMEM160	0.459068315	3.03E-29
6729	HERC3	-0.493252067	3.77E-34
6730	PPFIA1	-0.235186897	3.70E-08
6731	PPP1R12A	-0.236522901	3.08E-08
6732	ZBTB33	-0.519024102	3.12E-38
6733	MICAL1	0.590678897	1.27E-51
6734	GTF2H1	-0.144400212	0.000808856
6735	FAM49B	-0.125065586	0.003762781
6736	PRELP	-0.003775243	0.930577966
6737	UBE2F	0.25036163	4.33E-09
6738	RHBDD1	-0.366041435	2.09E-18
6739	MAPRE3	0.085719995	0.047510527
6740	OLR1	0.002068539	0.961928556
6741	LRRC25	0.113943062	0.008341075
6742	MACC1	-0.324770416	1.32E-14
6743	NEFL	-0.012346352	0.775709371
6744	AMIGO1	-0.206995895	1.37E-06
6745	FASTKD2	-0.324285984	1.45E-14
6746	PICK1	0.498124937	6.77E-35
6747	CRBN	-0.373810866	3.47E-19
6748	FJX1	0.239471421	2.05E-08
6749	SPATA13	-0.26817132	2.90E-10
6750	BET1	-0.11832268	0.006143143
6751	NIPAL2	-0.258143484	1.36E-09
6752	MYCBP	-0.14332813	0.000885405
6753	WDR60	0.132135719	0.002194243
6754	ERC1	-0.407582709	7.90E-23
6755	COL14A1	-0.152483286	0.000401011
6756	FNIP1	-0.404091949	1.97E-22
6757	NARS2	-0.3743863	3.03E-19
6758	NID2	-0.155560719	0.000304168
6759	BLOC1S3	0.442621767	4.49E-27
6760	RPUSD4	-0.001980867	0.963541019
6761	MRVI1	-0.189066067	1.07E-05
6762	AMACR	-0.137066551	0.001483181
6763	CWF19L1	0.189694306	9.98E-06
6764	ARPIN	-0.293229301	4.54E-12
6765	POM121C	0.00187874	0.96541953
6766	SETD1A	0.451482409	3.15E-28
6767	ABHD17A	0.629281247	2.43E-60
6768	NAA10	0.74100385	3.05E-94
6769	RLF	-0.236590711	3.05E-08
6770	SEMA5A	-0.413111355	1.82E-23
6771	ABTB1	0.442987833	4.03E-27
6772	NSMCE2	-0.009353829	0.829102054
6773	TTC28	-0.253493383	2.73E-09
6774	CNTROB	0.447524758	1.04E-27
6775	RANBP10	0.0182586	0.67348495
6776	GGA1	0.517758276	5.04E-38
6777	ITPR1	-0.386126734	1.82E-20
6778	ZKSCAN8	-0.327270332	8.04E-15
6779	INPP5D	0.129832343	0.002623241
6780	HHLA3	0.417656779	5.34E-24
6781	STEAP3	0.137331503	0.001451766
6782	RAB3GAP2	-0.482292539	1.62E-32
6783	CLIP3	0.256278931	1.80E-09
6784	FAM229B	-0.204556724	1.83E-06

6785	CDR2L	0.212580058	6.97E-07
6786	BCR	-0.076357859	0.077629614
6787	PRKCH	-0.212219621	7.28E-07
6788	TOMM40L	0.108704545	0.01187215
6789	FAM193A	0.188379927	1.15E-05
6790	APOOL	-0.62266089	9.28E-59
6791	PPRC1	0.313081645	1.25E-13
6792	WBP1	0.335915488	1.41E-15
6793	GGH	0.070741749	0.102157587
6794	ARHGAP25	0.116470734	0.006999697
6795	HEY1	-0.154857156	0.000324157
6796	TPGS2	-0.220392219	2.62E-07
6797	KDM1B	-0.293823468	4.09E-12
6798	FAM98B	-0.515768115	1.07E-37
6799	UBE3B	-0.24825813	5.88E-09
6800	UROS	0.258519245	1.29E-09
6801	CREB3L4	0.230145445	7.31E-08
6802	GTPBP4	0.075109518	0.082620947
6803	SHFM1	0.532354824	1.74E-40
6804	PGS1	0.481313338	2.25E-32
6805	CKLF	0.376043913	2.05E-19
6806	LANCL2	-0.306583242	4.17E-13
6807	GCC1	-0.352309281	4.44E-17
6808	ZNF561	-0.458930519	3.17E-29
6809	DHRS7B	0.252875196	2.99E-09
6810	ICE1	-0.363637474	3.61E-18
6811	KLHL20	-0.35610953	1.93E-17
6812	BEX5	-0.144768768	0.000783987
6813	C11orf71	0.00480325	0.911743001
6814	WDR36	-0.309358794	2.50E-13
6815	NAT9	0.684424292	3.65E-75
6816	EAF1	-0.411564932	2.75E-23
6817	DBR1	-0.319797544	3.47E-14
6818	RCSD1	-0.127615966	0.003106879
6819	ATG14	-0.108230383	0.012249208
6820	TGS1	-0.238731979	2.27E-08
6821	TBC1D25	0.143402881	0.000879858
6822	CEP170B	0.262523161	6.98E-10
6823	APPBP2	-0.457055249	5.68E-29
6824	SORBS2	-0.362820022	4.34E-18
6825	SNRPA1	0.575525107	1.63E-48
6826	HNRNPLL	-0.267842665	3.05E-10
6827	HEATR5B	-0.272834981	1.38E-10
6828	PDP1	-0.386383959	1.71E-20
6829	RUNDC1	-0.083450053	0.053723853
6830	GAK	0.288078036	1.10E-11
6831	CREB1	-0.428755397	2.47E-25
6832	MAP3K7CL	-0.045663966	0.291750083
6833	SESN3	-0.354893213	2.53E-17
6834	LMO4	-0.107731616	0.012657211
6835	GABRD	0.087330178	0.043478162
6836	DDX28	0.005900272	0.891698214
6837	ZNF3	0.063146942	0.14466622
6838	KLHL22	0.14689415	0.000653857
6839	YIPF4	-0.236631419	3.04E-08
6840	DVL2	0.299758477	1.43E-12
6841	EYA3	-0.23371869	4.52E-08
6842	TBC1D14	-0.507770269	2.09E-36

6843	ARHGEF28	-0.403328708	2.40E-22
6844	MUS81	0.645552518	2.14E-64
6845	MFAP3L	-0.399882622	5.83E-22
6846	SLC7A9	0.039028858	0.367603249
6847	FOXP4	0.211715754	7.75E-07
6848	RNF111	-0.515673178	1.11E-37
6849	HAT1	-0.286961459	1.34E-11
6850	PTPN13	-0.361551952	5.77E-18
6851	SLC5A10	0.008307031	0.847980273
6852	STK38L	-0.277710556	6.26E-11
6853	GAS8	0.158605649	0.000230217
6854	C5orf22	-0.417972147	4.90E-24
6855	LINC00116	0.514306008	1.85E-37
6856	RABIF	0.115886174	0.007291458
6857	KPNA1	-0.240406441	1.80E-08
6858	CETN3	-0.202786589	2.26E-06
6859	KDM5A	-0.409345665	4.96E-23
6860	TRAPPC8	-0.442655865	4.44E-27
6861	ZNF791	-0.174742142	4.83E-05
6862	DNAJC10	-0.139561648	0.001210595
6863	FLI1	-0.1649174	0.000127159
6864	ALKBH4	0.32246767	2.07E-14
6865	C21orf59	0.536290268	3.60E-41
6866	ATP11C	-0.390852466	5.69E-21
6867	RNF220	0.571313243	1.12E-47
6868	FLRT3	-0.341375993	4.55E-16
6869	DICER1	-0.276143326	8.08E-11
6870	EIF2AK4	-0.384207707	2.91E-20
6871	TMEM39A	0.043996514	0.309746552
6872	SYNM	-0.308429376	2.97E-13
6873	IFNAR2	0.042485752	0.32667031
6874	NLRX1	0.026913463	0.534489953
6875	SLC20A2	-0.161401129	0.000177474
6876	ETAA1	-0.113196792	0.00877873
6877	UBE2D1	-0.19608252	4.90E-06
6878	MED7	-0.15369438	0.000359899
6879	MAP4K5	-0.178010328	3.46E-05
6880	ARL2BP	-0.431599378	1.10E-25
6881	THOC2	-0.157831116	0.00024724
6882	CSTF2	-0.110151479	0.010784211
6883	ADAM17	-0.314990003	8.69E-14
6884	UBE2D4	0.021176701	0.625034528
6885	SNX24	-0.229966089	7.49E-08
6886	FMO2	-0.202925659	2.22E-06
6887	FBXL17	-0.546273404	6.02E-43
6888	AMBRA1	-0.196886322	4.47E-06
6889	ZDHHC2	-0.409853763	4.34E-23
6890	OPLAH	0.123747901	0.004148702
6891	ZNF436	-0.258360701	1.32E-09
6892	OSBPL3	0.038308698	0.37651546
6893	NGF	0.207479136	1.29E-06
6894	STX18	0.052339534	0.226811228
6895	ZNF25	-0.31378617	1.09E-13
6896	PGM3	-0.13161967	0.002284362
6897	PLEKHA5	0.006118382	0.887720723
6898	TBCD	0.360437404	7.40E-18
6899	UPRT	-0.505705792	4.44E-36
6900	WDR74	0.532522592	1.63E-40

6901	CXorf40B	0.246184366	7.93E-09
6902	ATP1B2	-0.025788706	0.55171091
6903	RAB3IP	-0.282441938	2.86E-11
6904	TM7SF2	0.015583903	0.719119752
6905	EDC3	0.15484098	0.00032463
6906	EFTUD1	-0.361800414	5.46E-18
6907	FAM73A	-0.523195978	6.30E-39
6908	ADPRH	-0.176428333	4.07E-05
6909	KIAA1143	-0.556439459	8.08E-45
6910	HOGA1	-0.000367068	0.993241647
6911	POLM	0.515577267	1.15E-37
6912	DNAJB6	-0.031294049	0.470099389
6913	CEP104	-0.268568145	2.72E-10
6914	NMT2	-0.38063075	6.89E-20
6915	PIAS4	0.481658099	2.01E-32
6916	TRIM33	-0.371376565	6.12E-19
6917	GTF2F2	-0.00839952	0.846308629
6918	NEIL2	-0.09702331	0.024819309
6919	PACS2	0.311008335	1.84E-13
6920	AFAP1L2	-0.00895757	0.836237332
6921	CCDC71L	0.036319094	0.401820223
6922	PILRA	0.265515267	4.39E-10
6923	C6orf226	0.465439248	4.07E-30
6924	ELN	0.039468167	0.362231548
6925	TRMT2B	-0.25489642	2.22E-09
6926	CCL28	-0.072485504	0.093958403
6927	IDE	-0.391309702	5.08E-21
6928	GPN2	0.242415732	1.36E-08
6929	IGFBP2	0.105513181	0.014620815
6930	C7orf55	0.394543389	2.26E-21
6931	PTCD3	0.083136673	0.054632559
6932	GZMH	0.423345562	1.12E-24
6933	DPP8	-0.46810901	1.73E-30
6934	LETM1	0.136343827	0.001572074
6935	PCDHGC3	-0.278056314	5.91E-11
6936	ABHD8	0.330670213	4.08E-15
6937	LNX2	-0.541038313	5.23E-42
6938	THUMPD3	-0.326153279	1.00E-14
6939	RBBP6	0.037836524	0.382430304
6940	PRKD3	-0.389711592	7.55E-21
6941	TXLNG	-0.199066381	3.48E-06
6942	CDC42EP5	0.410190485	3.97E-23
6943	TYW1	-0.14553049	0.000734817
6944	CREBRF	-0.50068595	2.72E-35
6945	SLC35F2	0.027139167	0.531067109
6946	DNAAF5	0.124454123	0.003937626
6947	FBXO38	-0.352949401	3.86E-17
6948	PEX7	-0.413250256	1.76E-23
6949	EPSTI1	0.012028938	0.781327566
6950	RPS6KB2	0.624691288	3.07E-59
6951	MPP5	-0.566473593	9.89E-47
6952	MVK	0.260822936	9.06E-10
6953	RBM19	0.311833383	1.57E-13
6954	POT1	-0.403350857	2.39E-22
6955	ISLR	0.111047481	0.010155699
6956	LRIG1	-0.118869727	0.005908798
6957	VPS9D1	0.564107886	2.83E-46
6958	RSRP1	0.442229207	5.04E-27

6959	ERO1LB	-0.443074838	3.93E-27
6960	DIP2B	-0.36706557	1.65E-18
6961	C10orf35	0.23604948	3.29E-08
6962	UIMC1	0.186237524	1.45E-05
6963	MTHFD2	0.181953889	2.29E-05
6964	RCOR3	-0.084380252	0.051100385
6965	PRR12	0.165328419	0.000122245
6966	POLR2M	-0.538371003	1.55E-41
6967	MAPK8IP3	0.430821805	1.37E-25
6968	GRB7	0.050285245	0.245595018
6969	EPB41L3	-0.133515743	0.001968978
6970	PLEKHG2	0.333789585	2.17E-15
6971	RAB27A	-0.175794496	4.34E-05
6972	USP38	-0.523178387	6.35E-39
6973	BPTF	-0.349046278	8.98E-17
6974	IKZF5	-0.424243093	8.73E-25
6975	SRPK2	-0.343973351	2.64E-16
6976	PPM1D	-0.238616014	2.31E-08
6977	NSMAF	0.077476643	0.073367053
6978	CCDC93	-0.110849831	0.010291476
6979	EEF2K	0.004555542	0.916277359
6980	VPS37B	0.149409749	0.000525803
6981	CLIP1	-0.282311447	2.92E-11
6982	ZNF160	0.047077061	0.277058283
6983	CGREF1	0.263974935	5.58E-10
6984	SDHAF2	0.490072823	1.14E-33
6985	NOX4	-0.205731602	1.60E-06
6986	NUP85	0.625804108	1.66E-59
6987	TMEM175	0.543953746	1.58E-42
6988	FOXC1	-0.151494997	0.000437757
6989	N6AMT2	0.188580883	1.13E-05
6990	ZBTB14	-0.250568493	4.20E-09
6991	C20orf194	-0.238460933	2.36E-08
6992	BEND5	-0.110226295	0.010730441
6993	RNF34	0.231322219	6.25E-08
6994	RNF170	-0.348624604	9.83E-17
6995	DDHD2	-0.347716131	1.19E-16
6996	VPS45	0.200322057	3.01E-06
6997	MIOS	-0.279206453	4.89E-11
6998	ATG4C	-0.363640959	3.60E-18
6999	ZDHHC18	0.521440553	1.24E-38
7000	SBNO1	-0.403575884	2.25E-22
7001	CHAMP1	-0.430739267	1.41E-25
7002	PTGDS	0.168936696	8.61E-05
7003	SLC9A6	-0.445228437	2.07E-27
7004	ZBED6CL	0.140546917	0.00111629
7005	TAOK3	-0.386584033	1.63E-20
7006	FNTA	0.200324224	3.01E-06
7007	ZNF354A	0.182418973	2.18E-05
7008	CDK5	0.452050376	2.65E-28
7009	ZNF512B	0.360845603	6.76E-18
7010	NEDD1	-0.366579807	1.85E-18
7011	ALKBH2	0.426558957	4.57E-25
7012	USP20	0.369739726	8.95E-19
7013	H2AFY2	0.097463873	0.024168333
7014	KLHL2	-0.34564544	1.85E-16
7015	IRF5	0.514667359	1.62E-37
7016	SMPD2	0.525660183	2.43E-39

7017	TM2D1	-0.317761684	5.13E-14
7018	DCAF10	-0.44884702	6.99E-28
7019	PQLC2	0.619396205	5.42E-58
7020	CIT	-0.0390459	0.36739394
7021	SMUG1	0.421044299	2.11E-24
7022	GGA3	0.396416008	1.41E-21
7023	CARD16	0.379642346	8.73E-20
7024	TNRC6A	0.146005467	0.000705619
7025	PSME4	-0.063482053	0.142542317
7026	AP1M1	0.538801847	1.30E-41
7027	FBXO3	-0.427020243	4.02E-25
7028	MID1	-0.162433586	0.000161038
7029	EIF2B3	0.049781785	0.25035992
7030	HPS4	0.157130097	0.000263655
7031	BAG4	-0.44165567	5.97E-27
7032	GNB4	-0.128485038	0.00290832
7033	SREK1IP1	-0.201212198	2.72E-06
7034	NSMCE4A	0.349771379	7.68E-17
7035	ZNF266	0.279972692	4.31E-11
7036	MAD2L2	0.327613319	7.51E-15
7037	ITFG2	0.016161283	0.709177031
7038	VPS13C	-0.40347609	2.31E-22
7039	SPATA2L	0.415593053	9.35E-24
7040	QSOX2	0.228930236	8.60E-08
7041	SPECC1L	-0.004409036	0.918960456
7042	ERCC5	0.384259661	2.87E-20
7043	ITGAL	0.270171156	2.11E-10
7044	CLASRP	0.669051848	1.04E-70
7045	SLC35E2B	0.05359782	0.215823128
7046	DHX8	-0.151200317	0.000449306
7047	C9orf41	-0.434489818	4.81E-26
7048	FZD5	-0.210373034	9.13E-07
7049	FAM103A1	-0.060289524	0.16376924
7050	RNF24	-0.078723107	0.068844901
7051	KCNK1	0.069559515	0.108032411
7052	RC3H2	-0.448725912	7.25E-28
7053	VPS37D	-0.130035728	0.002582491
7054	ANKRD46	-0.368010272	1.33E-18
7055	KIF2A	-0.238136518	2.47E-08
7056	ZNF644	-0.387241858	1.39E-20
7057	AHCTF1	-0.204406097	1.87E-06
7058	ELMSAN1	-0.113094087	0.008840538
7059	DALRD3	0.121843226	0.00476996
7060	NARF	0.650284704	1.27E-65
7061	PUS3	0.084520953	0.050713034
7062	EXD2	-0.370930267	6.79E-19
7063	ATP2B1	-0.327943317	7.03E-15
7064	ARL14EP	-0.458821704	3.28E-29
7065	ZDHHC1	0.1950226	5.52E-06
7066	CDC37L1	-0.182423388	2.18E-05
7067	ZNF627	-0.433452604	6.48E-26
7068	TMEM186	0.03573674	0.409415111
7069	CAPN5	-0.102129855	0.018131262
7070	DAAM2	-0.184091283	1.83E-05
7071	RBM26	0.100287286	0.020336672
7072	MALSU1	0.112731778	0.00906168
7073	SMURF2	-0.150937422	0.000459849
7074	CSTF3	0.089837148	0.037775643

7075	MSH3	-0.474922987	1.89E-31
7076	ACAP2	-0.416820308	6.70E-24
7077	SENP2	-0.272998857	1.34E-10
7078	C19orf25	0.310707012	1.94E-13
7079	EXOC5	-0.458585252	3.53E-29
7080	PPP2R5E	-0.448205599	8.48E-28
7081	PSMD6	-0.018137691	0.675523697
7082	UEVLD	-0.526096917	2.05E-39
7083	ZNF688	0.508709795	1.48E-36
7084	EXOSC2	0.226946244	1.12E-07
7085	ELP6	0.262310119	7.22E-10
7086	PDE7A	0.068029801	0.116026034
7087	P2RX4	0.290722337	7.01E-12
7088	EMC8	0.376927513	1.67E-19
7089	ZBTB17	0.633873755	1.84E-61
7090	RGL3	0.232097005	5.63E-08
7091	DSN1	0.176668454	3.97E-05
7092	C19orf60	0.659230418	5.30E-68
7093	LARP4B	-0.079526462	0.06605327
7094	PEX13	-0.268702659	2.66E-10
7095	MTX1	0.433576142	6.25E-26
7096	HOXA4	0.039112921	0.366571546
7097	PHGDH	0.066408939	0.124994142
7098	SUGP1	0.626701112	1.02E-59
7099	STK36	0.463487754	7.56E-30
7100	MMD	-0.120610401	0.005215627
7101	DOHH	0.4838	9.74E-33
7102	ZMYM2	-0.100710217	0.019810808
7103	PCID2	0.187301939	1.30E-05
7104	ADNP2	-0.331771599	3.27E-15
7105	SMG8	-0.407910948	7.25E-23
7106	TROVE2	-0.472928683	3.63E-31
7107	PTS	0.172126596	6.28E-05
7108	HYI	0.472361279	4.36E-31
7109	FAM46A	-0.261101026	8.69E-10
7110	TMEM134	0.522445808	8.42E-39
7111	C2	0.114530839	0.008010183
7112	SMC5	-0.235809471	3.40E-08
7113	TUBB2A	0.158091725	0.000241385
7114	YBEY	0.3482839	1.06E-16
7115	USP2	-0.033906449	0.433833184
7116	MRPL22	0.25079715	4.06E-09
7117	RNLS	-0.229794943	7.67E-08
7118	AOAH	0.011012162	0.799399775
7119	STARD13	-0.194875193	5.61E-06
7120	SECISBP2	-0.108317428	0.012179206
7121	MCEE	0.165708809	0.000117856
7122	STRN	-0.518010164	4.58E-38
7123	WASF1	-0.065281385	0.131544289
7124	TPCN2	0.172703046	5.93E-05
7125	RNF103	-0.190006769	9.65E-06
7126	GALNT10	-0.15550278	0.00030577
7127	EIF2AK3	-0.261525908	8.14E-10
7128	CCR1	-0.094733471	0.028452094
7129	ABHD14A	0.440294666	8.91E-27
7130	PEAR1	-0.047518566	0.272572897
7131	ZFP90	-0.284581585	2.00E-11
7132	TRIO	0.060338259	0.163428265

7133	SRPK1	-0.253502656	2.73E-09
7134	SLC22A6	0.043963214	0.310113242
7135	DTX4	-0.087146829	0.04392227
7136	PSAT1	0.155509055	0.000305596
7137	ACTR8	-0.387026431	1.46E-20
7138	FLYWCH1	0.43816489	1.66E-26
7139	AUTS2	-0.097722013	0.023793806
7140	C11orf95	-0.120326629	0.00532338
7141	PRDM1	-0.103723455	0.016395274
7142	NFRKB	0.041355093	0.339720726
7143	VARS2	0.349455126	8.22E-17
7144	RARB	-0.39596311	1.58E-21
7145	ARHGEF10	-0.153945013	0.000351898
7146	TYSND1	0.388666427	9.77E-21
7147	DMTF1	0.269687934	2.28E-10
7148	RUVBL1	0.330466507	4.25E-15
7149	SUSD3	0.103257039	0.016887628
7150	TAPT1	-0.399575468	6.31E-22
7151	TRMT61B	-0.320533432	3.01E-14
7152	LYVE1	-0.170929633	7.08E-05
7153	SUSD2	0.125286905	0.00370125
7154	PARVB	0.077799328	0.072173732
7155	IFT43	0.538452566	1.50E-41
7156	CIPC	-0.413045602	1.85E-23
7157	D2HGDH	0.488176148	2.19E-33
7158	AGK	0.138525782	0.001317614
7159	MRPS25	0.198197454	3.85E-06
7160	NRP2	-0.1148103	0.00785701
7161	SPTLC3	-0.355255022	2.33E-17
7162	DUS3L	0.676288554	8.98E-73
7163	EXOSC8	0.434007496	5.53E-26
7164	CASP8	0.05592218	0.196541049
7165	TCERG1	0.33784366	9.48E-16
7166	SAMD8	-0.355182694	2.37E-17
7167	RNF208	0.17490493	4.75E-05
7168	PRPF18	-0.410813325	3.36E-23
7169	WDR55	0.173145342	5.67E-05
7170	C11orf73	0.384460456	2.73E-20
7171	MYO9A	-0.464435182	5.60E-30
7172	CRELD2	0.591286611	9.42E-52
7173	PPIE	0.266182274	3.96E-10
7174	ZNF76	0.362201245	4.99E-18
7175	PPL	-0.12492109	0.003803451
7176	IFT122	0.075369821	0.081559294
7177	ILK	-0.175650321	4.41E-05
7178	N4BP2L2	0.079585781	0.065850882
7179	MED28	-0.253616856	2.68E-09
7180	HEXDC	0.556415815	8.16E-45
7181	TNFAIP8L2	0.366025038	2.10E-18
7182	PPP1R14A	0.282371761	2.89E-11
7183	GNPNAT1	-0.388647905	9.81E-21
7184	DZIP1	0.003503428	0.935565026
7185	RBM23	-0.143148314	0.000898882
7186	TGDS	-0.162732489	0.000156553
7187	ANKRD54	0.632857385	3.27E-61
7188	CCDC174	0.113420335	0.008645533
7189	CDC26	-0.023693464	0.584498029
7190	PIK3CD	0.22445707	1.55E-07

7191	PDE4A	0.109101252	0.011564609
7192	CLEC18B	0.114586109	0.00797968
7193	SEPSECS	-0.256098887	1.85E-09
7194	SLC25A12	-0.156301465	0.000284371
7195	GLB1L2	-0.003541546	0.934865506
7196	LIMS2	0.156285734	0.000284779
7197	HIST1H4H	0.02021239	0.640880482
7198	RIOK1	0.017524164	0.685905315
7199	AARS2	0.118383861	0.006116525
7200	POP4	0.303659417	7.10E-13
7201	TMEM246	-0.180861032	2.57E-05
7202	ZADH2	-0.243575496	1.15E-08
7203	ZNF598	0.591478354	8.58E-52
7204	FGFR2	-0.284222946	2.12E-11
7205	AFAP1	-0.185989457	1.49E-05
7206	MICALL1	0.130520169	0.002487745
7207	SENP3	0.378412455	1.17E-19
7208	UCHL5	-0.353555592	3.38E-17
7209	FOXN2	-0.288622221	1.01E-11
7210	RNF115	-0.013601688	0.753607157
7211	SETD1B	0.146071569	0.000701642
7212	OGFOD3	0.147667091	0.000611724
7213	SLCO4A1	0.246761243	7.30E-09
7214	RWDD4	-0.328156532	6.74E-15
7215	AREL1	-0.127576111	0.003116274
7216	MIER3	-0.300108388	1.35E-12
7217	XPO5	0.081910815	0.058310838
7218	CEP68	-0.403314348	2.41E-22
7219	LMO7	-0.349049549	8.97E-17
7220	DCBLD2	0.005537888	0.898312721
7221	VHL	-0.056012822	0.195815472
7222	ORAI2	0.256219798	1.82E-09
7223	PPT2	0.040092254	0.35468526
7224	RPP38	0.453350326	1.78E-28
7225	UVRAG	-0.352475806	4.28E-17
7226	LDHD	-0.145705416	0.000723936
7227	ZCCHC7	-0.141508893	0.001030793
7228	CASP2	0.094502158	0.028843276
7229	C3orf38	-0.358863877	1.05E-17
7230	KDM5B	-0.324886315	1.29E-14
7231	TRMT61A	0.554183003	2.13E-44
7232	MED23	-0.285242548	1.79E-11
7233	TRAF3IP1	-0.170818294	7.16E-05
7234	BCL9L	-0.017413988	0.687775963
7235	TRIM13	-0.232810909	5.11E-08
7236	BRCC3	-0.23420136	4.23E-08
7237	KIF13A	-0.431187863	1.24E-25
7238	MOSPD1	-0.33515987	1.64E-15
7239	GMIP	0.389633362	7.69E-21
7240	TPST2	-0.013741261	0.751161763
7241	SLC9A9	0.098936032	0.022098994
7242	ANKLE2	0.225806046	1.30E-07
7243	LMBR1L	0.484374916	8.02E-33
7244	RABEPK	0.456566517	6.61E-29
7245	SEC22C	-0.384104962	2.98E-20
7246	BCOR	-0.208400772	1.16E-06
7247	TAZ	0.740373134	5.29E-94
7248	KMT2D	-0.024949515	0.564734272

7249	FPGT	-0.348904462	9.25E-17
7250	ARRB1	-0.161789774	0.00017111
7251	PPP2R3C	-0.268258077	2.86E-10
7252	FAM200B	-0.040297491	0.35222537
7253	LIMK1	0.379340827	9.39E-20
7254	C2CD2	-0.089702648	0.038064616
7255	KCNJ3	-0.201245252	2.71E-06
7256	RABEP2	0.51106117	6.20E-37
7257	FBXL15	0.443379275	3.59E-27
7258	FAM104B	0.168318176	9.15E-05
7259	DCPS	0.021324838	0.62261528
7260	KIAA1598	-0.195619332	5.16E-06
7261	FBXO31	0.169491986	8.16E-05
7262	SNAI2	0.020060676	0.643388681
7263	MTMR1	-0.22411222	1.62E-07
7264	STAG1	-0.441138975	6.95E-27
7265	STAU2	-0.446316587	1.50E-27
7266	CKS1B	0.164888912	0.000127506
7267	FAM114A2	-0.448584396	7.57E-28
7268	NDUFV2	0.306304229	4.39E-13
7269	VAMP4	-0.170957302	7.06E-05
7270	IL15RA	0.482055487	1.76E-32
7271	TFEB	0.247049003	7.00E-09
7272	DLL1	0.046145033	0.286690954
7273	ZNF281	-0.181277195	2.46E-05
7274	SIPA1L3	0.207830824	1.24E-06
7275	SCN4B	-0.145330722	0.000747429
7276	PPP1R16B	-0.194599215	5.79E-06
7277	ZNF777	0.302888517	8.17E-13
7278	TMEM174	-0.082237322	0.057311636
7279	FAM208B	-0.158622399	0.000229861
7280	CLN3	0.428639674	2.55E-25
7281	ASNS	0.267256681	3.35E-10
7282	PC	-0.054388913	0.209113593
7283	ERMARD	0.125442785	0.003658462
7284	PLXNA1	0.310923401	1.87E-13
7285	FAM160B2	0.550182613	1.17E-43
7286	RBFA	0.319892691	3.40E-14
7287	PTPN14	-0.380596129	6.95E-20
7288	C11orf49	0.247534442	6.53E-09
7289	DNAJC16	-0.248298277	5.85E-09
7290	DYNC1LI1	-0.238846832	2.23E-08
7291	SNX30	-0.426096262	5.21E-25
7292	SLC1A4	-0.0634359	0.142833408
7293	SCRN3	-0.450378846	4.40E-28
7294	PCDH17	-0.320653059	2.94E-14
7295	CDC42EP2	0.134463297	0.001826786
7296	FGR	0.373692354	3.57E-19
7297	CDPF1	0.445787911	1.75E-27
7298	NGDN	0.180676488	2.62E-05
7299	POLR3K	0.265021303	4.74E-10
7300	MTM1	-0.523004376	6.79E-39
7301	NSUN5	0.56967786	2.35E-47
7302	TSC1	0.047892195	0.268815998
7303	MPHOSPH6	-0.027794745	0.521188432
7304	SLC39A11	0.06230694	0.150096281
7305	GMNN	-0.029729724	0.492590905
7306	SFR1	-0.045011727	0.298704345

7307	ZFYVE21	0.050965789	0.239255475
7308	KLF15	-0.011740177	0.786448515
7309	UBP1	-0.173980233	5.22E-05
7310	SEC24D	-0.226440127	1.20E-07
7311	EPM2AIP1	-0.140356506	0.00113397
7312	GLTSCR1L	-0.394349358	2.37E-21
7313	ARL4A	0.015884996	0.713928775
7314	ANKFY1	-0.345994248	1.72E-16
7315	ATRX	-0.534334723	7.91E-41
7316	UCHL1	0.028017585	0.517852139
7317	EPHA7	-0.160323501	0.000196298
7318	PKLR	-0.013820642	0.749772079
7319	PRKAB2	-0.349533461	8.08E-17
7320	WDR47	-0.359011608	1.02E-17
7321	CHD1	-0.098971862	0.022050607
7322	LRP6	-0.411644524	2.70E-23
7323	USP32	-0.34409782	2.57E-16
7324	DUSP7	0.001348633	0.975173017
7325	PNPT1	-0.010085437	0.815965532
7326	GTF2I	-0.523756462	5.08E-39
7327	LIAS	-0.237997176	2.51E-08
7328	EVA1C	0.223328024	1.79E-07
7329	HACL1	-0.256160717	1.84E-09
7330	FARP1	-0.298026828	1.95E-12
7331	ELL	0.348840752	9.38E-17
7332	CAMK2G	0.126116373	0.003478675
7333	RSBN1	-0.467276996	2.26E-30
7334	LAYN	-0.078195467	0.070730491
7335	CNOT2	-0.163911413	0.000139982
7336	LYRM7	-0.48412034	8.74E-33
7337	UHRF1BP1L	-0.560856293	1.18E-45
7338	BTBD7	-0.352741198	4.04E-17
7339	DIRAS2	-0.208228465	1.18E-06
7340	ZEB2	-0.230962294	6.56E-08
7341	STYX	-0.317771017	5.12E-14
7342	ZBED5	0.051459128	0.234732376
7343	DCP1A	-0.353287006	3.59E-17
7344	GPRASP2	-0.503778907	8.93E-36
7345	UBALD1	0.536556672	3.24E-41
7346	TANGO2	-0.024427075	0.572915778
7347	MECOM	-0.35501811	2.46E-17
7348	MYO10	-0.318560642	4.40E-14
7349	NEBL	-0.453077334	1.93E-28
7350	TAF2	-0.295502708	3.05E-12
7351	ZFYVE16	-0.421695207	1.77E-24
7352	RANGRF	0.515095563	1.38E-37
7353	FZD8	0.104469794	0.015633574
7354	VNN1	0.02352286	0.587207025
7355	PLXNA2	-0.187626766	1.25E-05
7356	MTOR	-0.260774802	9.13E-10
7357	IL4I1	0.394989782	2.02E-21
7358	HTT	-0.309374522	2.49E-13
7359	YLPM1	-0.358280991	1.20E-17
7360	PTH2R	0.038753821	0.370991339
7361	NXT2	-0.354586226	2.70E-17
7362	AGL	-0.589610532	2.12E-51
7363	SLC25A40	-0.531320757	2.63E-40
7364	SLC34A2	-0.026203627	0.545326597

7365	IPO11	-0.502180341	1.59E-35
7366	GALT	0.358699061	1.09E-17
7367	TMEM39B	0.479634104	3.96E-32
7368	TRIM32	-0.328638202	6.12E-15
7369	ZBTB10	-0.428525564	2.63E-25
7370	ADAT1	-0.14181125	0.001005196
7371	UBE2O	0.419230308	3.48E-24
7372	AGFG2	0.070846385	0.101650082
7373	NUDT4	-0.209468416	1.02E-06
7374	TP53RK	-0.080187312	0.063827178
7375	ATG12	0.039323352	0.363996847
7376	CCDC12	0.488767206	1.79E-33
7377	SHISA4	0.089870667	0.037703918
7378	CAPN7	-0.461151346	1.58E-29
7379	ARMCX6	0.01083119	0.802627942
7380	HIGD1B	0.07767327	0.072638008
7381	ENTPD4	0.078037192	0.071304274
7382	WBSCR27	0.13069058	0.002455178
7383	NDEL1	0.086842287	0.044668365
7384	TMEM192	-0.496832297	1.07E-34
7385	FRMD4B	-0.163229248	0.000149358
7386	RHBDF1	0.457911481	4.35E-29
7387	TFEC	-0.166440463	0.000109828
7388	NGRN	-0.178221814	3.39E-05
7389	PTPMT1	0.151313431	0.00044484
7390	B3GALTL	-0.228306933	9.34E-08
7391	PLEKHH3	0.389548223	7.86E-21
7392	PHIP	-0.205912866	1.56E-06
7393	KIF1B	-0.463570651	7.37E-30
7394	EXOC3L1	0.15644668	0.000280634
7395	ACTG2	0.079921899	0.064713688
7396	OSGEP	0.527954544	9.90E-40
7397	LYAR	0.37960395	8.81E-20
7398	EPT1	-0.366809406	1.75E-18
7399	ZNF292	-0.424995492	7.08E-25
7400	DHX58	0.490528294	9.72E-34
7401	SLC16A13	0.120192595	0.005374969
7402	FES	0.296930952	2.37E-12
7403	GXYLT1	-0.459144743	2.96E-29
7404	BCAR3	-0.212145836	7.35E-07
7405	LUC7L	0.525111835	3.00E-39
7406	PLEKHG1	-0.288800222	9.76E-12
7407	IRAK4	-0.16423235	0.000135765
7408	SMAGP	0.045931513	0.288929098
7409	METTL18	-0.168875753	8.67E-05
7410	ZDHHC24	0.275622769	8.80E-11
7411	TRAPPC12	0.515561738	1.16E-37
7412	PCSK6	-0.015165996	0.726346094
7413	MCM4	0.099922131	0.020800424
7414	RSBN1L	-0.260209141	9.95E-10
7415	MRPL48	0.066928201	0.122064257
7416	MIER2	0.506541525	3.27E-36
7417	TBC1D2	0.034230381	0.42945145
7418	HPS5	-0.204203381	1.91E-06
7419	TMEM200B	0.163082159	0.000151455
7420	SLC9A8	0.071773287	0.097241343
7421	NCS1	0.255174956	2.13E-09
7422	KIF21A	-0.321547329	2.47E-14

7423	DHRS1	0.319683906	3.54E-14
7424	WDR37	-0.210639186	8.83E-07
7425	QPCTL	0.520540279	1.75E-38
7426	IFT20	0.45916744	2.94E-29
7427	BCKDHB	-0.475471144	1.57E-31
7428	MTA1	0.442488346	4.67E-27
7429	LATS1	-0.402758955	2.78E-22
7430	DDX19A	0.010703973	0.804899255
7431	SNUPN	0.195883898	5.01E-06
7432	USP3	-0.268323075	2.83E-10
7433	CTIF	0.054601744	0.207334496
7434	HSPB11	0.412828186	1.97E-23
7435	TATDN3	-0.234621327	4.00E-08
7436	DOCK11	-0.155189487	0.000314567
7437	RARG	0.226417985	1.20E-07
7438	KLC2	0.468716321	1.42E-30
7439	C11orf84	0.537586222	2.13E-41
7440	IGSF6	0.089361147	0.038806762
7441	ZCCHC24	-0.14641535	0.000681291
7442	GAS2L3	0.115127472	0.007686342
7443	MRPS17	0.075690325	0.080267305
7444	FAM150B	0.014475215	0.738344027
7445	DHX36	-0.380000746	8.02E-20
7446	HSPA12B	-0.032588476	0.451924553
7447	RFC5	-0.023221615	0.592004528
7448	IFT140	0.191594484	8.10E-06
7449	IFFO2	0.12693024	0.003272162
7450	ADRA1B	0.01845255	0.670219573
7451	TNFAIP8	-0.04259362	0.325442465
7452	CHD9	-0.426761818	4.32E-25
7453	NELFA	0.399216053	6.91E-22
7454	GPT	0.256419245	1.77E-09
7455	PDE2A	-0.136706211	0.001526908
7456	PTK2B	0.19666925	4.58E-06
7457	SNX25	-0.276619047	7.48E-11
7458	DNAJC25	-0.153226052	0.000375308
7459	TCF4	-0.29026217	7.59E-12
7460	NUPL2	0.211894377	7.58E-07
7461	COBLL1	-0.453119704	1.91E-28
7462	FADS3	0.636342797	4.52E-62
7463	DMWD	0.375179925	2.52E-19
7464	MOB3C	0.21000965	9.54E-07
7465	AMDHD2	0.404808655	1.63E-22
7466	TRMT12	-0.085018802	0.049362088
7467	ZNF800	-0.406817897	9.66E-23
7468	HSPA14	-0.06086173	0.15979937
7469	RGS3	0.16544157	0.000120923
7470	LHPP	0.262670217	6.83E-10
7471	P4HTM	0.321512129	2.49E-14
7472	ZZZ3	-0.41449517	1.26E-23
7473	RPS6KB1	-0.153602957	0.00036286
7474	SLAMF8	0.216663021	4.19E-07
7475	DIAPH2	-0.278552945	5.45E-11
7476	ZBTB5	-0.095056765	0.027912998
7477	C9orf114	0.521755261	1.10E-38
7478	ZC3H18	0.252288785	3.26E-09
7479	TMEM164	-0.127628042	0.003104038
7480	EXOSC9	0.347210817	1.33E-16

7481	SCNN1A	-0.219824942	2.81E-07
7482	PPARA	-0.36212263	5.07E-18
7483	TMEM220	-0.299956673	1.38E-12
7484	IRF8	-0.015132836	0.72692054
7485	SENP5	0.080989117	0.061209615
7486	VSTM4	-0.325846765	1.07E-14
7487	GALNT15	-0.258032342	1.38E-09
7488	GPATCH4	0.118246919	0.006176247
7489	NOC3L	-0.228815147	8.73E-08
7490	GCC2	-0.394660225	2.20E-21
7491	FOXK1	-0.155642895	0.00030191
7492	TMC4	-0.023826457	0.5823903
7493	AGPAT9	-0.281373941	3.42E-11
7494	RCN2	-0.398246713	8.85E-22
7495	ECM2	-0.230029078	7.43E-08
7496	PWWP2A	-0.223317754	1.80E-07
7497	ALG1	0.044448127	0.304801696
7498	TRIM23	-0.481898365	1.85E-32
7499	ZDHHC17	-0.089936319	0.037563768
7500	ZNF608	-0.036862889	0.394804888
7501	CINP	0.288948162	9.52E-12
7502	TDRD3	-0.126978144	0.003260361
7503	ST3GAL2	0.208722418	1.11E-06
7504	CDK5RAP2	-0.094944556	0.028099104
7505	IDNK	0.131117884	0.002375219
7506	IL17RA	-0.035504331	0.412469737
7507	RSPH3	-0.465359013	4.17E-30
7508	ZNF704	-0.33692005	1.15E-15
7509	UPF3A	0.289674159	8.40E-12
7510	KIAA0895L	0.409121231	5.27E-23
7511	WDR44	-0.362957437	4.20E-18
7512	MYOM3	-0.004552594	0.916331327
7513	KAT7	-0.391200261	5.22E-21
7514	LSS	0.101390815	0.01898941
7515	ZNF511	0.513522085	2.48E-37
7516	CARS	0.435318789	3.79E-26
7517	CPOX	0.14354754	0.000869214
7518	MEX3D	0.19177089	7.94E-06
7519	SLC2A8	0.223219328	1.82E-07
7520	ACAD10	0.070344397	0.104103169
7521	RIMKLB	0.061198398	0.157497787
7522	ETV3	-0.32602502	1.03E-14
7523	HAUS2	-0.385856787	1.95E-20
7524	ASPN	-0.039225528	0.365192356
7525	FTCD	-0.026668181	0.538222245
7526	POLR1B	-0.29180692	5.81E-12
7527	CASK	-0.232236339	5.52E-08
7528	TRIP11	-0.523790314	5.01E-39
7529	AVEN	0.302317045	9.06E-13
7530	MED20	-0.222415284	2.02E-07
7531	WHSC1L1	-0.210992395	8.46E-07
7532	MEX3C	-0.277022952	7.00E-11
7533	EXOSC1	0.610669567	5.47E-56
7534	CLCC1	-0.36502041	2.64E-18
7535	MECP2	-0.330778503	3.99E-15
7536	GOLGA1	0.125619348	0.003610538
7537	SLC45A4	-0.127542264	0.003124273
7538	ASXL2	-0.49517439	1.92E-34

7539	KCTD15	0.109300843	0.011412562
7540	SGSM2	0.347108274	1.36E-16
7541	HDAC11	-0.078388922	0.070034308
7542	C1D	-0.32729446	8.00E-15
7543	CCDC91	-0.004976821	0.908567453
7544	SNTB2	-0.301585103	1.03E-12
7545	HLF	-0.413056309	1.85E-23
7546	STIM2	-0.041995035	0.33229391
7547	VPRBP	-0.340276555	5.73E-16
7548	PIGM	-0.247189932	6.86E-09
7549	MAST4	-0.139798389	0.001187289
7550	COX10	-0.20145215	2.64E-06
7551	LMBR1	-0.524346701	4.04E-39
7552	SLC5A8	-0.142413038	0.000955986
7553	NAA15	-0.337474436	1.02E-15
7554	ZNF747	0.187511432	1.27E-05
7555	APH1B	-0.496686159	1.13E-34
7556	RASSF8	-0.432530949	8.44E-26
7557	UBE2W	-0.484964243	6.56E-33
7558	ATG2A	0.092520096	0.032388601
7559	KIAA1109	-0.456395328	6.96E-29
7560	CNNM4	-0.114760817	0.00788394
7561	AP5Z1	0.64255056	1.25E-63
7562	ALPK3	-0.228001734	9.73E-08
7563	GCLC	-0.441642482	5.99E-27
7564	RNF166	0.573081487	5.01E-48
7565	DDO	-0.28718319	1.29E-11
7566	SNAPC1	-0.001197317	0.977957838
7567	SYNPO2	-0.217582507	3.74E-07
7568	TOPBP1	-0.113607649	0.008535313
7569	SLC5A1	-0.055047895	0.203640633
7570	CCDC51	0.221534456	2.26E-07
7571	PPP1R14C	-0.004746484	0.912781877
7572	KIF16B	-0.513897765	2.16E-37
7573	DOK1	0.372994948	4.20E-19
7574	ARL6IP6	-0.013008001	0.764036121
7575	HMG20A	-0.475887364	1.37E-31
7576	RAVER2	-0.326253033	9.83E-15
7577	SLCO3A1	-0.066112771	0.126689563
7578	CA11	0.176779198	3.93E-05
7579	TRPM7	-0.278358077	5.63E-11
7580	PCTP	-0.286833314	1.37E-11
7581	ARL5B	-0.355396995	2.26E-17
7582	SDCCAG8	-0.065861825	0.128140035
7583	SLC7A8	-0.12774233	0.003077263
7584	HIF1AN	-0.318341537	4.59E-14
7585	TADA1	-0.179464131	2.98E-05
7586	VWA8	-0.512218227	4.04E-37
7587	GCFC2	-0.288408862	1.04E-11
7588	USPL1	0.03052394	0.481100541
7589	ARHGAP23	0.040354499	0.351544012
7590	KMT2B	0.458438095	3.69E-29
7591	FANCF	-0.122824351	0.004440167
7592	SQLE	-0.109592145	0.011193825
7593	IRF6	-0.399551119	6.35E-22
7594	FBXL4	-0.539449618	1.00E-41
7595	WDR33	0.003919721	0.927928273
7596	MUC20	-0.066689657	0.123403502

7597	PLCL2	-0.417391413	5.74E-24
7598	ZBTB47	-0.113589247	0.008546086
7599	THEM4	0.047593576	0.271815798
7600	AHNAK2	0.104612306	0.015491712
7601	SLC22A4	0.050748505	0.241266935
7602	SYNRG	-0.354762814	2.60E-17
7603	ARNT2	-0.279990818	4.30E-11
7604	SMIM12	0.051411162	0.235169474
7605	SEMA4D	0.001456905	0.973180574
7606	GDA	-0.066314789	0.125531185
7607	DYRK2	-0.17214448	6.27E-05
7608	IL33	-0.209971172	9.58E-07
7609	CRY1	-0.058910769	0.173638613
7610	ADPRM	-0.003507913	0.935482726
7611	NRCAM	-0.196954584	4.43E-06
7612	ZBTB8OS	0.433523393	6.35E-26
7613	B9D1	0.269084496	2.51E-10
7614	ME2	-0.247713621	6.36E-09
7615	COL27A1	0.364229227	3.15E-18
7616	FAM104A	-0.147003542	0.000647735
7617	CADM4	-0.118996479	0.005855659
7618	ZCCHC6	-0.146171368	0.000695677
7619	ITGA8	-0.24641471	7.67E-09
7620	C2orf43	-0.200865019	2.83E-06
7621	GIT2	-0.111147558	0.010087558
7622	PRKRIR	-0.239563034	2.02E-08
7623	SNAP47	0.077669911	0.072650412
7624	DOLPP1	0.26774027	3.10E-10
7625	ERMAP	-0.383795825	3.21E-20
7626	CTDSPL2	-0.377589533	1.42E-19
7627	TARSL2	-0.202339573	2.38E-06
7628	JMJD6	0.540385888	6.84E-42
7629	MAML1	0.015769214	0.71592337
7630	TFDP2	-0.246045349	8.09E-09
7631	NAPSA	-0.068476694	0.113644254
7632	CCNT2	0.086213688	0.046242117
7633	CENPT	0.640500457	4.13E-63
7634	THAP1	-0.400376503	5.14E-22
7635	UBIAD1	-0.207840606	1.24E-06
7636	MTG2	0.583848991	3.35E-50
7637	HDAC2	-0.306086357	4.56E-13
7638	MVD	0.529281927	5.88E-40
7639	EPS15L1	0.070944737	0.101174879
7640	MSH6	-0.195115537	5.46E-06
7641	DIS3	-0.379860681	8.29E-20
7642	SOCS1	0.437698798	1.90E-26
7643	FGD4	-0.402424546	3.03E-22
7644	TMEM97	-0.203618087	2.05E-06
7645	IFRD1	0.181893765	2.31E-05
7646	DDX59	-0.148373089	0.000575454
7647	ZNF263	0.183600991	1.93E-05
7648	PEX12	-0.443625631	3.33E-27
7649	XRN1	-0.342464498	3.63E-16
7650	UTP14A	0.228475006	9.14E-08
7651	ZWINT	0.291999589	5.62E-12
7652	TMEM125	-0.159202875	0.000217847
7653	CNOT4	-0.430996951	1.31E-25
7654	ATP8B1	-0.379266126	9.56E-20

7655	SLC12A9	0.506090345	3.86E-36
7656	FIP1L1	-0.237823694	2.58E-08
7657	EDA2R	-0.24709863	6.95E-09
7658	MCM2	0.096385646	0.025788318
7659	MXRA5	-0.057535445	0.183918281
7660	NAA35	-0.289797362	8.23E-12
7661	XRCC6BP1	0.04441926	0.305116202
7662	CLK4	0.179802191	2.87E-05
7663	IL2RB	0.207992803	1.22E-06
7664	MTHFD1L	-0.020502368	0.636097818
7665	HOXA9	0.009193833	0.831981376
7666	CPSF4	0.480823046	2.66E-32
7667	QRFPR	-0.023742648	0.583718126
7668	MLLT10	-0.080911613	0.061458701
7669	USP30	-0.391264678	5.13E-21
7670	INPP5E	0.598992329	2.11E-53
7671	SCARA3	0.045448969	0.29403032
7672	PROM1	-0.247351827	6.70E-09
7673	PRKD1	-0.232308398	5.47E-08
7674	SPATS2	0.106497451	0.013718771
7675	ZSWIM1	0.283357469	2.45E-11
7676	AGAP3	0.633180904	2.73E-61
7677	HFE	-0.248339211	5.81E-09
7678	SNAI1	0.156542393	0.000278196
7679	TMEM168	-0.29729081	2.23E-12
7680	GCDH	0.12034641	0.005315804
7681	KMT2A	-0.276130206	8.10E-11
7682	SLC27A1	0.063590158	0.141862274
7683	EOGT	-0.06903505	0.110722572
7684	SLC25A10	0.338018202	9.14E-16
7685	CAMSAP1	-0.25097013	3.96E-09
7686	CBL	-0.328745389	5.99E-15
7687	CAD	0.37160727	5.80E-19
7688	NT5C2	-0.340420158	5.56E-16
7689	LYSMD1	-0.039221123	0.365246247
7690	RHOBTB3	-0.277338681	6.65E-11
7691	MYO5B	-0.398342359	8.64E-22
7692	LTB	0.487243755	3.01E-33
7693	PLEKHA7	-0.256540761	1.73E-09
7694	FRS2	-0.489650427	1.32E-33
7695	PROSER1	-0.011313949	0.794024128
7696	RFWD3	-0.069798646	0.106823077
7697	DAGLB	0.466304722	3.09E-30
7698	DNMBP	-0.220615614	2.54E-07
7699	MDC1	0.01718787	0.691621134
7700	BAZ1A	0.187677489	1.24E-05
7701	SGK2	-0.012580894	0.771565481
7702	RCE1	0.510304978	8.21E-37
7703	KIZ	-0.068064463	0.115839911
7704	TJAP1	0.419964804	2.84E-24
7705	FAM149B1	-0.251104513	3.88E-09
7706	POLR3F	-0.190936746	8.71E-06
7707	HERC1	-0.385133111	2.32E-20
7708	DEF6	0.492736258	4.51E-34
7709	CCDC82	0.01193371	0.78301532
7710	SAA1	0.198591243	3.68E-06
7711	MBIP	-0.237852725	2.57E-08
7712	ACN9	-0.039670359	0.359775785

7713	CETP	0.103099368	0.017056969
7714	FOXC2	0.047819959	0.269539568
7715	MECR	0.097505719	0.024107276
7716	PACSIN3	0.195637932	5.15E-06
7717	DGKD	0.230016222	7.44E-08
7718	PLK3	0.377042293	1.62E-19
7719	TMTC1	-0.454449647	1.27E-28
7720	pk	-0.295462028	3.07E-12
7721	PARP16	0.063058126	0.145233158
7722	SEMA3C	-0.122902786	0.004414713
7723	GTF3C3	-0.254671753	2.29E-09
7724	AP3M2	-0.064215995	0.137974146
7725	CSRP2BP	-0.303155407	7.78E-13
7726	CPA3	-0.252469098	3.18E-09
7727	COL5A3	0.216690082	4.18E-07
7728	MTHFS	-0.063221458	0.144191866
7729	PER3	-0.264427755	5.20E-10
7730	OPA3	-0.027527439	0.525204957
7731	SOGA1	-0.086540416	0.04541841
7732	LRRFIP2	0.053406939	0.217464963
7733	MSI2	-0.376457052	1.86E-19
7734	UBXN7	-0.421431892	1.90E-24
7735	MAPK8IP1	0.06891059	0.111368654
7736	APOBEC3G	0.360428156	7.42E-18
7737	PRPSAP1	0.002116227	0.961051564
7738	TCF7L1	0.050640576	0.242270464
7739	ZXDC	0.058440741	0.177102488
7740	DSTYK	-0.468744779	1.41E-30
7741	BBS12	-0.389471654	8.01E-21
7742	USB1	0.301030195	1.14E-12
7743	CX3CR1	-0.258155034	1.36E-09
7744	CCDC102A	0.392815393	3.49E-21
7745	KRTCAP3	0.188776042	1.10E-05
7746	ANKRD27	-0.064745173	0.134750883
7747	ACOX3	0.069485906	0.108406833
7748	DDX19B	0.227301693	1.07E-07
7749	LSM6	0.045514498	0.293334072
7750	HABP2	-0.14494784	0.000772162
7751	TMEM80	0.224885098	1.46E-07
7752	RPTOR	0.078005653	0.071419061
7753	FUK	0.257990998	1.39E-09
7754	HEATR3	0.206471551	1.46E-06
7755	CRYZL1	-0.206736676	1.42E-06
7756	NDC1	-0.348212674	1.07E-16
7757	NBEAL2	0.31917349	3.91E-14
7758	HOOK2	0.233482206	4.67E-08
7759	ADAMTS9	0.006925957	0.873018869
7760	ETV5	-0.079107507	0.067497291
7761	TMEM62	-0.147314464	0.000630623
7762	SIK3	-0.291679928	5.94E-12
7763	ZNF865	0.539130255	1.14E-41
7764	ELMO1	0.005495974	0.899078219
7765	COG5	-0.459538267	2.62E-29
7766	HSPB6	0.132928097	0.002062179
7767	MAPK7	0.473769521	2.75E-31
7768	TOR1AIP2	-0.229412516	8.07E-08
7769	R3HCC1L	-0.169006788	8.56E-05
7770	BLNK	-0.266695271	3.65E-10

7771	RIOK2	-0.333476525	2.31E-15
7772	TMEM187	0.136077157	0.001606091
7773	ZSCAN18	-0.053385591	0.217649134
7774	LDLRAD3	0.030889184	0.475865615
7775	RALGAPA2	-0.510951493	6.46E-37
7776	ANKRD12	-0.282268434	2.94E-11
7777	LFNG	0.23163958	5.99E-08
7778	GOSR2	-0.055848868	0.197129343
7779	SRP19	0.366313641	1.96E-18
7780	BRIX1	0.229024674	8.49E-08
7781	TESC	0.080323242	0.063377042
7782	GMEB2	0.375594526	2.28E-19
7783	AP5B1	0.029084012	0.502039822
7784	APOPT1	0.355881471	2.03E-17
7785	C12orf45	0.449689944	5.42E-28
7786	NUDT8	0.452862292	2.06E-28
7787	DCHS1	-0.186386038	1.43E-05
7788	ARID4B	-0.216075543	4.51E-07
7789	EEA1	-0.353440516	3.47E-17
7790	CCDC127	0.066405409	0.125014245
7791	DCP2	-0.275021524	9.70E-11
7792	ABCC2	0.026642538	0.538613179
7793	TRNAU1AP	0.442872284	4.17E-27
7794	PAWR	-0.230937665	6.58E-08
7795	METTL3	0.329327712	5.34E-15
7796	MYRF	0.168722254	8.80E-05
7797	MGAM	-0.203874762	1.99E-06
7798	AGAP1	-0.226156407	1.24E-07
7799	EXOC8	-0.453848809	1.53E-28
7800	PRPF39	0.323910532	1.56E-14
7801	RUNX3	0.235960054	3.33E-08
7802	MED22	0.021168392	0.625170338
7803	CCM2L	0.025469484	0.556647522
7804	TMEM242	-0.266916474	3.53E-10
7805	INTS12	-0.116727206	0.006875022
7806	RPP30	0.086699478	0.045021889
7807	TTF1	-0.088016571	0.041849033
7808	LHX4-AS1	0.42236018	1.47E-24
7809	THNSL1	-0.42961306	1.94E-25
7810	ICK	-0.482371221	1.58E-32
7811	PCCB	-0.131421212	0.00231991
7812	FGB	0.007730595	0.858413858
7813	WDR89	-0.22983371	7.63E-08
7814	CHST13	0.311614457	1.64E-13
7815	TMA16	-0.173156476	5.67E-05
7816	DCN	0.009624569	0.824234964
7817	FBLN1	0.16616452	0.000112794
7818	RCHY1	-0.546438767	5.62E-43
7819	ZSWIM6	-0.207553498	1.28E-06
7820	NPRL2	0.540777709	5.82E-42
7821	MFHAS1	-0.231784772	5.87E-08
7822	MYCBP2	-0.384104964	2.98E-20
7823	FAM173B	-0.264365129	5.25E-10
7824	CARS2	0.519898297	2.23E-38
7825	TBXAS1	0.118494958	0.006068457
7826	EPB41L4A	-0.418756546	3.96E-24
7827	CDKAL1	-0.340894066	5.03E-16
7828	YTHDC2	-0.196983366	4.42E-06

7829	C1orf174	0.389303206	8.35E-21
7830	VWA5A	-0.319660775	3.56E-14
7831	CCDC107	0.577637893	6.15E-49
7832	TMEM45A	-0.003059566	0.943714033
7833	COPS7B	0.600128088	1.20E-53
7834	RELL1	-0.446054525	1.62E-27
7835	PIAS1	-0.518848359	3.33E-38
7836	CDK5RAP1	0.458066029	4.15E-29
7837	ZNF260	-0.465753955	3.68E-30
7838	ARHGEF9	-0.254891323	2.22E-09
7839	DNMT1	0.240906203	1.68E-08
7840	MTHFR	0.024303677	0.574856381
7841	ANGEL2	-0.054509864	0.208101196
7842	UAP1L1	0.401523066	3.83E-22
7843	LMBRD2	-0.604955866	1.04E-54
7844	ACER3	-0.204673083	1.81E-06
7845	AQR	-0.496260564	1.31E-34
7846	ZNF212	0.367661797	1.44E-18
7847	CHI3L1	0.013259906	0.759605705
7848	AKAP9	-0.412883686	1.94E-23
7849	TMEM102	0.279790164	4.44E-11
7850	KMT2C	-0.342065476	3.94E-16
7851	AIFM2	0.092372934	0.032666073
7852	TATDN2	0.364479794	2.98E-18
7853	ORC4	-0.359608943	8.91E-18
7854	ARID1B	-0.341083672	4.84E-16
7855	ACE	-0.02390397	0.581163464
7856	IFT88	-0.122427239	0.004571086
7857	ATP8B2	0.074485349	0.085212176
7858	GNG2	-0.118607941	0.006019921
7859	GLIPR1	0.08278129	0.055678493
7860	DIRC2	-0.471384856	6.00E-31
7861	ZNF480	-0.26642673	3.81E-10
7862	CDKN2D	0.475278241	1.68E-31
7863	CERKL	-0.021878255	0.613613389
7864	TNFRSF10A	-0.047435855	0.273409395
7865	CEACAM1	-0.158014433	0.000243108
7866	IFFO1	0.620339907	3.26E-58
7867	VRK3	0.006844246	0.874504511
7868	SSH1	-0.168929408	8.62E-05
7869	PLXDC1	0.177743397	3.56E-05
7870	LAIR1	0.198320961	3.79E-06
7871	NOL4L	-0.025302493	0.559238487
7872	ZNF639	-0.236149944	3.24E-08
7873	WDR35	-0.29180477	5.82E-12
7874	ZNF618	-0.283462352	2.41E-11
7875	RASAL1	-0.057695243	0.182701315
7876	ATAD3A	0.570827838	1.40E-47
7877	PHLDB1	0.226434646	1.20E-07
7878	DPH1	0.185597847	1.56E-05
7879	METTL16	-0.168119362	9.33E-05
7880	CCPG1	-0.335484531	1.54E-15
7881	PHF8	-0.094923302	0.028134474
7882	KRTCAP2	0.644110101	5.01E-64
7883	METRN	0.375224906	2.49E-19
7884	TTC39A	-0.02729908	0.528648721
7885	ZBTB48	0.559482741	2.16E-45
7886	GSKIP	-0.276884168	7.16E-11

7887	KLHL7	-0.459039972	3.06E-29
7888	PDK1	-0.222784724	1.92E-07
7889	CDCA7L	-0.193973661	6.21E-06
7890	TSPYL5	-0.201424375	2.65E-06
7891	TMEM194A	-0.058099508	0.179649191
7892	MORN4	0.035446647	0.413229979
7893	PIN4	0.441271011	6.69E-27
7894	RAVER1	0.071668092	0.097733893
7895	C5orf28	0.019644347	0.650292379
7896	OSCP1	-0.11102128	0.010173606
7897	CHRNB1	0.16989996	7.84E-05
7898	HEY2	-0.125233995	0.003715877
7899	DENND1A	-0.013174054	0.761114777
7900	IKBKB	0.286048385	1.56E-11
7901	FAM109A	0.471883222	5.10E-31
7902	SREK1	0.102839057	0.017339799
7903	NRF1	0.244178091	1.06E-08
7904	CDK12	-0.281673591	3.25E-11
7905	MAPT	-0.087626377	0.042768706
7906	ZNF467	0.276180444	8.03E-11
7907	SPATA2	0.162752723	0.000156254
7908	NDUFA9	0.118249753	0.006175006
7909	NOS3	-0.048217558	0.265573429
7910	AP4M1	0.522894357	7.08E-39
7911	RIN3	0.327674701	7.42E-15
7912	AHSA2	0.442083656	5.26E-27
7913	EBF1	-0.121615934	0.004849448
7914	TMEM44	0.446415904	1.45E-27
7915	SRFBP1	-0.448046762	8.90E-28
7916	TMEM216	0.366360891	1.94E-18
7917	MAP3K13	-0.227682279	1.01E-07
7918	CNRIP1	-0.045368649	0.294885244
7919	MKLN1	-0.534242217	8.20E-41
7920	ABCC6	0.10792335	0.01249897
7921	POLR3D	0.120058132	0.005427174
7922	SLA	0.101734724	0.018585766
7923	CDC42EP3	-0.133951174	0.001902423
7924	SYTL3	0.200225944	3.05E-06
7925	SMAD9	-0.26243894	7.07E-10
7926	DOCK4	-0.343381396	2.99E-16
7927	RHBDD3	0.607104313	3.46E-55
7928	PTAFR	-0.086244251	0.046164538
7929	NR4A2	0.00660185	0.878914253
7930	ARL4D	-0.106466616	0.013746269
7931	LTN1	-0.398547999	8.20E-22
7932	SLC23A3	0.167066217	0.000103374
7933	MANSC1	-0.293386441	4.42E-12
7934	BOLA3	0.323752722	1.61E-14
7935	KIAA2026	-0.293742016	4.15E-12
7936	PNMA6A	0.138386679	0.00133263
7937	CC2D2A	-0.271864418	1.61E-10
7938	FRYL	-0.446163306	1.57E-27
7939	DENND4A	-0.448778693	7.14E-28
7940	SETD8	0.517652753	5.25E-38
7941	GPIHBP1	-0.035018838	0.418894032
7942	PIGG	0.061691666	0.154170981
7943	ARID4A	-0.272021362	1.57E-10
7944	VRK2	-0.254881614	2.22E-09

7945	IFI44L	-0.078412983	0.069948114
7946	RSRC1	-0.298575548	1.77E-12
7947	RGS12	0.191549071	8.14E-06
7948	ZNF710	-0.108411282	0.012104125
7949	PLCD3	0.169984404	7.77E-05
7950	DCUN1D3	0.144654572	0.000791616
7951	FBRSL1	0.403703541	2.18E-22
7952	CHD6	-0.391303884	5.08E-21
7953	PHYHIPL	-0.17308107	5.71E-05
7954	SHROOM4	-0.284422756	2.05E-11
7955	KTI12	0.164415246	0.000133415
7956	RNASEK	0.361560962	5.76E-18
7957	UPF3B	0.359309412	9.52E-18
7958	PKDCC	0.320174834	3.22E-14
7959	PHF14	-0.147987631	0.000595001
7960	MMP9	0.204576245	1.83E-06
7961	CD86	0.057373265	0.185159489
7962	CASP10	0.114664545	0.007936569
7963	TLE2	0.008045788	0.852705635
7964	TARBP2	0.618700876	7.87E-58
7965	ASAP2	-0.434923191	4.25E-26
7966	BTN2A2	0.089571748	0.03834765
7967	MFSD8	-0.281903947	3.13E-11
7968	ELF2	-0.251252993	3.80E-09
7969	POMZP3	0.161112348	0.000182345
7970	EIF4E3	-0.468467005	1.54E-30
7971	4-Sep	0.143730486	0.000855923
7972	ORC2	0.098745896	0.02235731
7973	FOXS1	0.246895974	7.16E-09
7974	LMNB1	0.302992496	8.02E-13
7975	TRMT11	0.056699929	0.190378826
7976	AXIN1	0.52866185	7.50E-40
7977	SESTD1	-0.442158222	5.15E-27
7978	KDM4B	0.16215216	0.00016537
7979	PI4K2B	-0.267529658	3.21E-10
7980	CSGALNACT1	-0.315640351	7.68E-14
7981	LDLRAP1	0.116248978	0.007109125
7982	DGCR8	0.385000167	2.40E-20
7983	CCDC8	-0.100770424	0.019736918
7984	UGT1A6	-0.111870147	0.009607491
7985	VEPH1	-0.154351017	0.000339287
7986	PAG1	-0.100911393	0.019564851
7987	SLFN13	0.159336044	0.000215175
7988	PIBF1	-0.081896457	0.058355109
7989	CXCL2	0.336567361	1.23E-15
7990	ZBTB42	-0.143018098	0.000908759
7991	MAST3	0.292686355	4.99E-12
7992	ANKRD37	0.139760057	0.001191034
7993	CDK6	-0.153869225	0.0003543
7994	RINL	0.239461705	2.05E-08
7995	CDYL	-0.378977486	1.02E-19
7996	MLXIPL	0.241062317	1.64E-08
7997	TSSC1	0.280998509	3.64E-11
7998	CCNB1	0.280612192	3.88E-11
7999	MRC1	-0.243566984	1.15E-08
8000	SH3BP5	-0.222655542	1.96E-07
8001	SELL	0.078364925	0.07012036
8002	IFT74	-0.009500006	0.826473413

8003	CHUK	-0.427461578	3.55E-25
8004	NUAK1	-0.250744483	4.09E-09
8005	TBRG1	0.176733239	3.94E-05
8006	RBM27	-0.351645085	5.13E-17
8007	MIS18A	-0.070141708	0.105106852
8008	MIIP	0.770427866	2.81E-106
8009	SLC37A3	-0.227317795	1.06E-07
8010	ZMAT5	0.375890762	2.13E-19
8011	TRPM4	0.300873467	1.17E-12
8012	PIKFYVE	-0.303649032	7.12E-13
8013	IFT172	0.206385762	1.48E-06
8014	SRBD1	-0.367292286	1.57E-18
8015	ZNF134	-0.423429081	1.09E-24
8016	PPP2R2D	0.066340198	0.125386072
8017	C7orf60	-0.43132168	1.19E-25
8018	DCUN1D1	-0.294586144	3.58E-12
8019	PFAS	0.058793288	0.174499623
8020	ARHGAP26	-0.113969924	0.008325691
8021	NTMT1	0.202942428	2.22E-06
8022	TRMT6	-0.009958932	0.818233444
8023	BCL7A	-0.10460487	0.015499087
8024	CRLF3	0.120631844	0.005207566
8025	GIPC3	-0.010110858	0.815509987
8026	LLGL1	0.021169539	0.625151586
8027	WDR59	0.193330827	6.67E-06
8028	PHACTR2	-0.458307979	3.85E-29
8029	FKBPL	0.306052344	4.59E-13
8030	VANGL1	-0.318100964	4.80E-14
8031	PGP	0.477159197	9.01E-32
8032	ZNF692	0.645717382	1.94E-64
8033	DNAJB14	-0.537632826	2.09E-41
8034	RASGRP3	-0.206913095	1.39E-06
8035	CYHR1	0.594291031	2.17E-52
8036	ANGEL1	-0.045560892	0.292841799
8037	CMTM7	0.439486915	1.13E-26
8038	ZNF652	-0.213892957	5.92E-07
8039	MED30	0.113820687	0.00841148
8040	KANSL1	-0.019669381	0.64987641
8041	PCNX	-0.451248619	3.38E-28
8042	ACOT8	0.379564832	8.90E-20
8043	NIPA1	-0.227563529	1.03E-07
8044	COA7	-0.113261756	0.008739835
8045	SRRD	-0.295979278	2.81E-12
8046	C1orf216	0.293478052	4.35E-12
8047	FAM214B	0.375932024	2.11E-19
8048	SFSWAP	0.513901765	2.15E-37
8049	DISP1	-0.183023561	2.05E-05
8050	HEATR1	-0.220778622	2.49E-07
8051	CDC40	-0.534548153	7.26E-41
8052	FKBP11	0.479275536	4.46E-32
8053	LIG1	0.420682478	2.34E-24
8054	AGTPBP1	-0.318003954	4.89E-14
8055	IGF2	-0.09639625	0.025771942
8056	RPAP3	-0.355570715	2.18E-17
8057	EXOSC7	0.033079352	0.445137251
8058	FAAH2	-0.23707242	2.86E-08
8059	EPOR	0.433097223	7.18E-26
8060	JAK2	-0.212847753	6.74E-07

8061	SLC2A9	-0.227100038	1.10E-07
8062	KCTD18	-0.290038441	7.89E-12
8063	ANKRD50	-0.431238129	1.22E-25
8064	NAPEPLD	-0.407550294	7.97E-23
8065	ITPR2	-0.497930538	7.26E-35
8066	DOPEY2	-0.097937091	0.023485599
8067	GCH1	0.020796595	0.631260504
8068	QSER1	-0.441213139	6.80E-27
8069	ACTR5	0.543073576	2.27E-42
8070	LRP3	0.223665701	1.72E-07
8071	POM121	-0.121175673	0.005006822
8072	SNRNP35	0.517382693	5.81E-38
8073	LRRC14	0.475222499	1.71E-31
8074	CKAP2	-0.151706697	0.000429632
8075	SLC13A1	-0.085853328	0.047165123
8076	GIMAP1	-0.004610604	0.91526919
8077	GAL3ST4	0.049759362	0.250573629
8078	FAM212A	0.252310302	3.25E-09
8079	NAA40	0.37239004	4.84E-19
8080	TRIL	-0.24538574	8.89E-09
8081	C2orf69	-0.389994881	7.04E-21
8082	BNIP1	0.422863411	1.28E-24
8083	MTERF2	0.219226601	3.03E-07
8084	DDX3Y	-0.065695069	0.129110981
8085	RNASEH1	-0.0154146	0.722044333
8086	TGFB3	0.119619514	0.005600657
8087	ZNF623	-0.27185748	1.61E-10
8088	CHM	-0.581094951	1.23E-49
8089	SLC26A11	0.404836778	1.62E-22
8090	FBXO11	-0.425809339	5.64E-25
8091	PFKFB4	0.097958883	0.023454565
8092	VCPIP1	-0.456066906	7.71E-29
8093	PAOX	0.318390988	4.54E-14
8094	RASGRP1	-0.202803607	2.25E-06
8095	ZC2HC1A	-0.336559254	1.23E-15
8096	CES4A	0.221362454	2.31E-07
8097	RAE1	0.189057762	1.07E-05
8098	SMG1	-0.129674138	0.002655343
8099	TNFSF9	0.307495803	3.52E-13
8100	RBM33	0.207485914	1.29E-06
8101	KATNB1	0.36108045	6.41E-18
8102	DMPK	0.452157125	2.56E-28
8103	OSBPL2	-0.088935698	0.039748457
8104	FAM110A	0.461276935	1.52E-29
8105	PMS2	-0.390181163	6.72E-21
8106	NHLRC2	-0.359297339	9.55E-18
8107	ABHD13	-0.417048554	6.30E-24
8108	ZFX	-0.286083533	1.55E-11
8109	PANK2	-0.108226149	0.012252622
8110	NUP107	0.125305547	0.003696109
8111	TIMM21	-0.159889188	0.000204401
8112	EFNA4	0.312160542	1.48E-13
8113	TMEM8B	-0.231506701	6.10E-08
8114	TMEM132A	0.331622665	3.37E-15
8115	GTF3C4	-0.437148341	2.23E-26
8116	SUGT1	-0.209326218	1.04E-06
8117	CLMN	-0.286580131	1.43E-11
8118	HOOK3	-0.20966869	9.94E-07

8119	ZC3HAV1L	-0.145177849	0.000757214
8120	ZNF84	-0.099788147	0.020972877
8121	MAST2	0.452468007	2.33E-28
8122	ZNF496	0.085878004	0.047101431
8123	FAM105A	-0.210416086	9.08E-07
8124	FAM178A	-0.105325374	0.014798736
8125	BTRC	-0.359165005	9.83E-18
8126	CMTM8	-0.170910454	7.09E-05
8127	TSNARE1	0.306573025	4.18E-13
8128	ARMC8	-0.223560362	1.74E-07
8129	PEX1	-0.298987948	1.65E-12
8130	N4BP2L1	0.021267192	0.623556221
8131	CNOT10	-0.111175396	0.010068676
8132	C4orf47	0.212795711	6.78E-07
8133	UBR1	-0.514698201	1.60E-37
8134	SERINC5	-0.452490131	2.31E-28
8135	TCOF1	0.548186354	2.70E-43
8136	MPV17L2	0.377966469	1.30E-19
8137	ZNF195	0.149393293	0.000526559
8138	LPCAT2	-0.379169109	9.78E-20
8139	INMT	-0.04754994	0.272256055
8140	BPHL	-0.127143132	0.003220013
8141	C2orf76	0.17909579	3.09E-05
8142	C12orf4	-0.23867568	2.29E-08
8143	NUDT1	0.579412771	2.69E-49
8144	ITGA9	-0.330505471	4.22E-15
8145	ZNF740	-0.114975753	0.007767563
8146	CROT	-0.404650503	1.70E-22
8147	ILKAP	0.639074369	9.42E-63
8148	CACUL1	-0.419468559	3.26E-24
8149	ZNRD1	0.478838775	5.16E-32
8150	AKNA	0.335792103	1.44E-15
8151	PYCRL	0.541504433	4.32E-42
8152	HELZ	-0.314734779	9.12E-14
8153	STON2	-0.009030597	0.83492133
8154	FBXL7	-0.019199941	0.657694821
8155	ZBTB40	-0.068368968	0.114214851
8156	SHC2	0.294981519	3.34E-12
8157	SLC5A6	0.413380315	1.70E-23
8158	AMT	-0.001921293	0.96463679
8159	SH2D4A	-0.358981205	1.02E-17
8160	IRS2	-0.13776703	0.001401453
8161	EDN2	0.032262033	0.456470355
8162	FADS2	0.225944	1.28E-07
8163	C16orf72	-0.49576627	1.56E-34
8164	NCKAP1L	0.001485255	0.972658882
8165	CLCN6	0.029300015	0.49886837
8166	ZNF654	-0.466765902	2.66E-30
8167	PKN3	0.368211321	1.27E-18
8168	SNX21	0.253464344	2.74E-09
8169	NUP37	-0.101924584	0.018366151
8170	NUP210	0.193148618	6.81E-06
8171	ENAH	-0.129911597	0.002607293
8172	SMCHD1	-0.264340231	5.27E-10
8173	ZNHIT3	0.054528491	0.207945592
8174	MLKL	0.351953041	4.79E-17
8175	SEH1L	-0.202135995	2.44E-06
8176	PLAUR	0.351160222	5.69E-17

8177	LPCAT4	0.570656095	1.51E-47
8178	TNFSF13B	0.087126796	0.043971024
8179	CLOCK	-0.521256523	1.33E-38
8180	NIN	-0.218674304	3.25E-07
8181	ASPHD1	0.305452678	5.13E-13
8182	DCP1B	-0.008224328	0.849475635
8183	RHOBTB2	-0.002498812	0.954017384
8184	FBXW9	0.378569314	1.13E-19
8185	HSPA2	-0.107725703	0.012662119
8186	HOXB6	0.056633791	0.190897267
8187	ASB7	-0.475344554	1.64E-31
8188	PRIMA1	0.052584384	0.224642462
8189	ALG13	0.197851881	4.00E-06
8190	PALM3	0.229251297	8.24E-08
8191	TRIM37	-0.201806038	2.53E-06
8192	EML1	-0.294456886	3.66E-12
8193	EXTL3	-0.126215029	0.003453027
8194	RAI1	0.07203311	0.096033259
8195	LRMP	-0.033851311	0.434581576
8196	C1orf50	0.251395031	3.72E-09
8197	OXSM	-0.034251214	0.429170527
8198	KIAA1468	-0.187208014	1.31E-05
8199	WDYHV1	-0.070787492	0.101935478
8200	SAMD9	-0.180563755	2.65E-05
8201	CADM1	-0.248627461	5.57E-09
8202	FAM214A	-0.377833678	1.34E-19
8203	SEMA3B	0.210050529	9.49E-07
8204	REV1	-0.085832627	0.047218613
8205	RASSF5	0.146334612	0.000686021
8206	BOD1L1	-0.374179671	3.18E-19
8207	HOXD10	-0.027402317	0.527090447
8208	TMTC2	-0.348584415	9.91E-17
8209	PIK3C2B	-0.106870974	0.013389503
8210	REPS2	-0.231578842	6.04E-08
8211	LRRC45	0.665383899	1.10E-69
8212	MSL3	0.254945323	2.20E-09
8213	DOK2	0.337344157	1.05E-15
8214	GLRX2	0.300731688	1.21E-12
8215	PCSK1N	0.241831037	1.47E-08
8216	GOLT1A	0.19749471	4.17E-06
8217	ZNF700	0.204408498	1.87E-06
8218	ZNF468	-0.443390746	3.58E-27
8219	HIST1H2BC	0.048898808	0.25887136
8220	HRCT1	0.13721698	0.001465269
8221	FMNL1	0.472411175	4.29E-31
8222	UBA6	-0.049654304	0.251576587
8223	GAPVD1	-0.324948182	1.27E-14
8224	CDCA2	0.016507622	0.70323643
8225	POLD3	-0.094688785	0.028527306
8226	FABP5	0.381049277	6.23E-20
8227	ARHGAP27	0.43182173	1.03E-25
8228	RPAP1	0.046230447	0.285798917
8229	NFAT5	-0.411862362	2.54E-23
8230	TMEM65	-0.395676616	1.70E-21
8231	TTLL1	0.116588547	0.006942177
8232	USP34	-0.477048032	9.35E-32
8233	GCAT	0.082430817	0.056726215
8234	ZNF609	-0.234005014	4.35E-08

8235	YAE1D1	0.113407356	0.008653217
8236	ABL2	-0.127706925	0.003085535
8237	C16orf45	-0.001722586	0.968292142
8238	MSANTD4	-0.428308049	2.80E-25
8239	ZNF143	-0.250093733	4.50E-09
8240	ZMYND19	0.677895554	3.07E-73
8241	CAMK1	-0.07044027	0.103631075
8242	TNKS	-0.436227888	2.91E-26
8243	RIC1	-0.340141414	5.89E-16
8244	TM2D3	0.116724057	0.006876541
8245	CFB	0.171446827	6.72E-05
8246	USP28	-0.321097927	2.70E-14
8247	GLYCTK	0.310535313	2.01E-13
8248	TTC21B	-0.128915423	0.002814328
8249	SRD5A1	-0.203605821	2.05E-06
8250	SPAST	-0.235069318	3.76E-08
8251	NLGN2	0.270905146	1.88E-10
8252	C1orf52	0.299199265	1.59E-12
8253	DDX51	0.470036621	9.29E-31
8254	KIAA0226	0.058910551	0.173640205
8255	DEXI	0.049761408	0.250554126
8256	PBRM1	-0.391652545	4.66E-21
8257	CEP70	-0.25085952	4.03E-09
8258	PHF21A	0.17718748	3.77E-05
8259	OARD1	-0.2515814	3.62E-09
8260	ALKBH1	-0.103029196	0.017132812
8261	FKBP7	-0.134546234	0.001814799
8262	ZZEF1	-0.139241299	0.001242804
8263	CREB3L3	0.254265638	2.44E-09
8264	DUSP2	0.3568177	1.65E-17
8265	SUSD1	-0.384826846	2.50E-20
8266	FBXO33	-0.320572285	2.98E-14
8267	PARP8	-0.117018716	0.006735733
8268	KLHL42	-0.192498506	7.32E-06
8269	VPS33B	0.368823572	1.10E-18
8270	KDM6A	-0.316402443	6.65E-14
8271	TNFRSF4	0.394078829	2.54E-21
8272	CDCP1	-0.057188125	0.186583953
8273	GSPT2	-0.300378407	1.28E-12
8274	PCYT2	0.330174693	4.50E-15
8275	PLEKHG3	-0.012995835	0.764250284
8276	BRAF	-0.421250111	2.00E-24
8277	OMA1	-0.176707402	3.96E-05
8278	DBNDD1	-0.032836802	0.448483693
8279	HOXA10	-0.054927105	0.204635956
8280	NADSYN1	0.487064431	3.21E-33
8281	SLC12A6	-0.337196461	1.08E-15
8282	EPB41L5	-0.487016177	3.26E-33
8283	INTS4	-0.063951961	0.139604402
8284	LRRC16A	-0.271729892	1.65E-10
8285	ENTPD2	0.238501609	2.34E-08
8286	ACAD8	-0.152766674	0.000391017
8287	CYP7B1	-0.484239431	8.39E-33
8288	NT5DC3	0.008861186	0.837974943
8289	INO80C	0.243716538	1.13E-08
8290	SCIN	-0.304271795	6.36E-13
8291	CYTIP	0.025191533	0.560963315
8292	SLC25A16	0.108162291	0.012304215

8293	CTPS2	-0.082264929	0.057227803
8294	DHX33	0.023793433	0.582913356
8295	B4GALT4	0.044157625	0.307976464
8296	FZD7	-0.238062661	2.49E-08
8297	LIMD2	0.618425386	9.12E-58
8298	BRD3	-0.21549678	4.85E-07
8299	CLPB	-0.118067395	0.006255332
8300	ADAMTSL2	-0.004685101	0.913905397
8301	TRAF6	-0.480239904	3.23E-32
8302	C2orf49	-0.148655843	0.000561496
8303	HNF4G	-0.080006124	0.064431278
8304	POLK	-0.447019539	1.21E-27
8305	RNGTT	-0.45849189	3.63E-29
8306	SHROOM1	0.455946331	8.00E-29
8307	PPFIBP2	-0.211874848	7.60E-07
8308	KLHL8	-0.629987106	1.64E-60
8309	TAF1B	-0.277490555	6.49E-11
8310	FBXO25	-0.334139707	2.02E-15
8311	FBXL19	0.544273756	1.38E-42
8312	PDK3	-0.348723574	9.62E-17
8313	SLC4A7	-0.090953368	0.035448484
8314	MED9	-0.087349953	0.043430491
8315	TMEM104	0.200861577	2.83E-06
8316	LRRC19	-0.209431001	1.02E-06
8317	NAA30	-0.549826431	1.36E-43
8318	MGAT3	-0.062743257	0.147256747
8319	CNEP1R1	-0.004328081	0.920443464
8320	TOE1	0.318413582	4.52E-14
8321	NEDD4	-0.259166786	1.17E-09
8322	CD27	0.325146115	1.22E-14
8323	FOXRED1	0.359920229	8.31E-18
8324	SENP7	-0.070307214	0.104286723
8325	METTL14	-0.539913454	8.29E-42
8326	FPR1	-0.027219564	0.52985055
8327	CCR5	0.117161825	0.006668283
8328	GSAP	0.180431888	2.69E-05
8329	RP11-54C4.3	0.051841743	0.231266247
8330	EMB	0.004327132	0.920460851
8331	ZSWIM4	0.29443816	3.68E-12
8332	CMTR2	-0.366999472	1.68E-18
8333	NLRC5	0.320588938	2.98E-14
8334	SLC29A3	0.14548812	0.000737475
8335	RSF1	-0.534633221	7.01E-41
8336	AC131263.1	-0.15315478	0.000377706
8337	PAAF1	0.061953454	0.152427138
8338	PIGA	-0.299360365	1.54E-12
8339	MLK4	-0.219914541	2.78E-07
8340	FAM117A	0.061639483	0.154520385
8341	SLC9A3	0.021685175	0.616747532
8342	CEP350	-0.298111362	1.92E-12
8343	ZBTB41	-0.474791414	1.97E-31
8344	TAF1	-0.193342955	6.66E-06
8345	ISY1	0.580187442	1.88E-49
8346	RBM4B	0.218877978	3.17E-07
8347	TMEM234	0.504540108	6.78E-36
8348	ADAMTS5	-0.271398972	1.74E-10
8349	SNIP1	-0.253656137	2.67E-09
8350	GLIS3	-0.315145903	8.44E-14

8351	COG6	-0.432225955	9.21E-26
8352	KANK3	0.131345491	0.002333605
8353	GNL3L	-0.264584448	5.08E-10
8354	HEATR6	-0.032157532	0.457930968
8355	MORC4	-0.255363318	2.07E-09
8356	ASCC3	-0.469553128	1.09E-30
8357	MST1	0.335835292	1.43E-15
8358	C14orf169	0.209333905	1.04E-06
8359	ATM	-0.146925061	0.000652122
8360	QRSL1	-0.260051067	1.02E-09
8361	MUM1	0.317424661	5.47E-14
8362	FBXO46	0.572068174	7.95E-48
8363	TUBGCP3	-0.129916277	0.002606354
8364	MITD1	0.471921769	5.04E-31
8365	NUDCD1	-0.198764952	3.60E-06
8366	MON2	-0.212815187	6.77E-07
8367	TDG	0.11455376	0.00799752
8368	HDDC3	0.250238503	4.41E-09
8369	TTC30A	-0.352870564	3.93E-17
8370	TMEM177	0.200038286	3.11E-06
8371	PRKCA	-0.201131732	2.74E-06
8372	UPB1	-0.029121751	0.501484954
8373	TVP23B	-0.257812945	1.43E-09
8374	PIR	0.161634661	0.000173624
8375	ERCC2	0.288875867	9.64E-12
8376	HSF2	-0.188950147	1.08E-05
8377	TMEM107	-0.071734439	0.097423007
8378	INPP5F	-0.212288796	7.22E-07
8379	OLFML1	0.019716141	0.649099707
8380	AP3S2	-0.30669287	4.08E-13
8381	KCTD17	0.45589828	8.12E-29
8382	LTF	-0.153360601	0.000370819
8383	ERICH5	-0.078968063	0.067983622
8384	STARD4	-0.204615462	1.82E-06
8385	KRT7	-0.133377519	0.001990547
8386	MAP2K5	-0.462253662	1.12E-29
8387	MTF2	0.035308731	0.415050984
8388	TRIM39	0.129362366	0.002719655
8389	TC2N	-0.279741145	4.48E-11
8390	PTK7	0.003742446	0.931179565
8391	TOMM5	0.290187587	7.69E-12
8392	GRK5	-0.024541789	0.571114539
8393	UMPS	-0.182462725	2.17E-05
8394	MRPS11	0.376729257	1.75E-19
8395	ACSM3	-0.255327529	2.08E-09
8396	TBCCD1	-0.155286713	0.000311812
8397	RTN4IP1	-0.026102956	0.546872244
8398	GFOD2	0.053678206	0.215134372
8399	RBMXL1	-0.331126748	3.72E-15
8400	BATF2	0.242188617	1.40E-08
8401	BRI3BP	-0.311352255	1.72E-13
8402	HELQ	-0.330367031	4.33E-15
8403	PTPN2	0.090141592	0.037128414
8404	ZNF776	-0.417159373	6.12E-24
8405	PANK4	0.143886875	0.000844711
8406	TMEM189	0.556008741	9.73E-45
8407	PIK3CA	-0.530076215	4.30E-40
8408	SSH2	-0.062077903	0.151603419

8409	EXOSC3	0.075268159	0.081972606
8410	SMC2	-0.396785549	1.28E-21
8411	CLP1	-0.196873724	4.47E-06
8412	SUGCT	-0.156905342	0.00026913
8413	CCL21	0.150164733	0.000492181
8414	TRAPPC2B	0.109695884	0.01111683
8415	CEPT1	-0.366005478	2.11E-18
8416	C11orf80	0.149297349	0.000530987
8417	CHKA	0.266799627	3.59E-10
8418	MBTPS2	-0.493257229	3.76E-34
8419	NUP155	-0.366555547	1.86E-18
8420	ZNF219	0.163097635	0.000151233
8421	CMKLR1	-0.131026856	0.00239205
8422	FGGY	-0.099404527	0.021473528
8423	PAXBP1	0.230609093	6.88E-08
8424	LYRM2	-0.411621856	2.71E-23
8425	ARSJ	-0.314030531	1.04E-13
8426	C16orf70	-0.062163391	0.151039543
8427	TRAF3	0.077032798	0.075034681
8428	OSBPL5	0.255066926	2.16E-09
8429	CCL3	0.214366847	5.59E-07
8430	NCALD	-0.289676266	8.40E-12
8431	GJC1	-0.080035715	0.064332297
8432	CCDC159	0.524715424	3.50E-39
8433	PUS7	0.065370193	0.13101895
8434	GPR125	-0.260971287	8.86E-10
8435	LCK	0.312998691	1.27E-13
8436	LONRF1	-0.158812134	0.000225868
8437	DHX37	0.37891734	1.04E-19
8438	SLC6A19	-0.093224762	0.031087694
8439	SEMA4G	0.029729873	0.492588736
8440	CHCHD5	0.438779229	1.39E-26
8441	NOL8	0.132748151	0.002091516
8442	FAM118B	-0.034416405	0.426946788
8443	MAP7D2	0.008304607	0.8480241
8444	HRH2	-0.165214736	0.000123586
8445	SOCS4	-0.309372894	2.49E-13
8446	LAMB3	0.005608484	0.897023574
8447	SH3PXD2B	-0.019193401	0.657804008
8448	TCAIM	-0.558378329	3.49E-45
8449	ZBTB45	0.340083146	5.96E-16
8450	RIF1	-0.336634588	1.22E-15
8451	PLAGL1	-0.022882788	0.597421871
8452	PAMR1	-0.043681766	0.313223899
8453	JAK3	0.380410144	7.27E-20
8454	SBF2	-0.51391934	2.14E-37
8455	ITGAM	0.049492759	0.253124255
8456	ITGAX	0.372046248	5.24E-19
8457	PPARGC1A	-0.441856571	5.63E-27
8458	FAM200A	-0.230240933	7.22E-08
8459	CARD8	0.102315648	0.017920927
8460	SGK223	0.061721165	0.153973723
8461	GK	-0.072384003	0.094420783
8462	OTULIN	-0.03064951	0.479297265
8463	FAM76A	-0.064063607	0.138913259
8464	TANC1	-0.295288371	3.17E-12
8465	NF1	-0.493275994	3.74E-34
8466	PARP2	0.25877726	1.24E-09

8467	COQ3	0.009665986	0.82349101
8468	ESCO1	-0.2300845	7.37E-08
8469	PARG	-0.374868167	2.71E-19
8470	SNX13	-0.549027836	1.90E-43
8471	THAP8	0.476238289	1.22E-31
8472	IL7R	0.006702903	0.877075406
8473	BIN2	0.212008147	7.47E-07
8474	ACOT4	-0.054880678	0.205019453
8475	ABO	0.019080686	0.659686983
8476	COG8	-0.130825883	0.002429597
8477	CCDC80	-0.042215091	0.329764408
8478	CXADR	-0.285781631	1.63E-11
8479	GNAI3	-0.293638842	4.23E-12
8480	FBXO42	-0.068212217	0.11504914
8481	MPDZ	-0.408456716	6.28E-23
8482	INTS9	0.0197871	0.647921793
8483	TEX10	-0.121454128	0.00490676
8484	C9orf91	0.038041103	0.379860642
8485	MYO1F	0.383068042	3.83E-20
8486	CHML	-0.336841251	1.16E-15
8487	GALNS	0.302162236	9.32E-13
8488	VPS13D	-0.50729849	2.48E-36
8489	ZNF142	0.256061257	1.86E-09
8490	TXNL4B	0.105408988	0.01471929
8491	MAP3K4	-0.249815134	4.69E-09
8492	SLC25A25	-0.155580019	0.000303637
8493	MRM1	0.432613986	8.24E-26
8494	CAMSAP3	0.184570481	1.74E-05
8495	ORC5	-0.060256464	0.164000844
8496	C22orf39	-0.107439985	0.012901291
8497	GPRIN3	-0.433340676	6.69E-26
8498	PLCB1	-0.364095627	3.25E-18
8499	AGO1	-0.353117231	3.72E-17
8500	ZNF408	0.5111518	6.00E-37
8501	HIRA	0.194212435	6.05E-06
8502	FAM173A	0.555837321	1.05E-44
8503	C18orf25	-0.327568022	7.58E-15
8504	FOXQ1	0.033482641	0.439604632
8505	EBI3	0.341388043	4.54E-16
8506	XKR8	0.434263056	5.14E-26
8507	SPSB2	0.54371291	1.74E-42
8508	GEMIN4	-0.129577849	0.002675056
8509	GZF1	-0.224769363	1.49E-07
8510	GADD45G	0.140620746	0.001109504
8511	CLN8	-0.188614324	1.12E-05
8512	EML2	0.270097444	2.14E-10
8513	PAGR1	0.407680008	7.70E-23
8514	CRADD	-0.284599891	1.99E-11
8515	VSIG10	-0.143577203	0.000867046
8516	GALNT12	-0.088923865	0.039774923
8517	CLUAP1	-0.233003647	4.98E-08
8518	CTPS1	0.184237622	1.80E-05
8519	CAMTA1	0.003300141	0.939296463
8520	SLC19A2	-0.171470275	6.71E-05
8521	LCAT	0.550358791	1.08E-43
8522	HMGXB4	-0.19509783	5.47E-06
8523	STX17	-0.480356972	3.11E-32
8524	C17orf85	0.121721422	0.00481241

8525	MYO5A	-0.418704678	4.02E-24
8526	FBXW8	-0.185913772	1.51E-05
8527	APC	-0.48572136	5.07E-33
8528	MB21D2	-0.129057554	0.002783901
8529	ZNF184	-0.252231504	3.29E-09
8530	VPS8	-0.091600861	0.034155305
8531	IQCE	0.202337725	2.38E-06
8532	E2F3	0.06159983	0.154786288
8533	MKS1	0.459294998	2.83E-29
8534	ZNF304	-0.449765829	5.30E-28
8535	BCL9	-0.262519447	6.99E-10
8536	MLF1	-0.102620408	0.017580525
8537	PDPK1	-0.467952817	1.82E-30
8538	C21orf2	0.393770001	2.75E-21
8539	SHISA3	-0.183005861	2.05E-05
8540	EFNA5	0.0562415	0.193993627
8541	GTF2IRD1	0.263686156	5.84E-10
8542	C7orf43	0.658692373	7.41E-68
8543	MED6	-0.037457045	0.387224898
8544	B3GALT4	0.214590439	5.43E-07
8545	EXTL2	-0.308762831	2.79E-13
8546	GTF2E1	-0.143293899	0.000887956
8547	VPS13A	-0.197053528	4.38E-06
8548	TIMP3	-0.326040289	1.03E-14
8549	FUT6	-0.041196229	0.341580726
8550	GPATCH11	-0.304480342	6.12E-13
8551	CDH11	0.00570868	0.895194393
8552	CTTNBP2	-0.143103104	0.0009023
8553	FASTKD1	-0.078021493	0.071361391
8554	GAB1	-0.478130853	6.53E-32
8555	PRR14L	-0.312681952	1.34E-13
8556	FABP4	-0.026289584	0.544008572
8557	RAB6B	-0.04861622	0.261637113
8558	UBXN8	-0.159134639	0.000219228
8559	TP53BP1	-0.313352187	1.18E-13
8560	RNF214	-0.17572954	4.37E-05
8561	PCBP4	0.435883686	3.22E-26
8562	KCTD14	-0.280196139	4.16E-11
8563	MOSPD2	-0.360771795	6.87E-18
8564	ARL13B	-0.135985076	0.001617993
8565	APBB3	0.488102173	2.25E-33
8566	ZNF784	0.311055812	1.82E-13
8567	CHIC1	-0.573545101	4.05E-48
8568	RFX1	0.281183599	3.53E-11
8569	ZMYM5	0.005810546	0.893335275
8570	HOXC9	0.354597817	2.69E-17
8571	RAP1B	-0.276445681	7.69E-11
8572	THOC5	0.182066075	2.27E-05
8573	PKHD1	-0.219565972	2.91E-07
8574	SLC26A2	-0.265062782	4.71E-10
8575	PDE3A	-0.24199354	1.44E-08
8576	RAB40B	-0.154895456	0.000323038
8577	PBX3	-0.053335912	0.218078165
8578	WDR24	0.398326711	8.68E-22
8579	FANCG	0.477614705	7.75E-32
8580	DNAJC2	0.300532784	1.25E-12
8581	TPRN	0.094127791	0.029486168
8582	XXYLT1	0.196119265	4.87E-06

8583	HUS1	-0.191930998	7.80E-06
8584	SIRT5	-0.267860297	3.04E-10
8585	CYTH4	0.300578754	1.24E-12
8586	SLC36A1	0.054190271	0.21078401
8587	PET117	-0.053166826	0.219542946
8588	ZNF275	-0.319046209	4.01E-14
8589	FAM219B	-0.210199207	9.32E-07
8590	TMEM41A	0.110139862	0.010792581
8591	DRG2	0.344763522	2.23E-16
8592	JDP2	0.111317915	0.009972498
8593	FHOD3	-0.20371319	2.03E-06
8594	PLCG2	-0.3169101	6.03E-14
8595	TCAF1	-0.394066472	2.55E-21
8596	PARK2	-0.378551732	1.13E-19
8597	MXD1	0.021444151	0.6206697
8598	ZBTB11	-0.222128764	2.09E-07
8599	ZNF274	0.028813655	0.506024257
8600	ZCCHC8	0.135955531	0.001621829
8601	C5orf46	0.145285353	0.00075032
8602	IPP	-0.392770583	3.53E-21
8603	PSD4	0.311550972	1.66E-13
8604	PPHLN1	-0.061137447	0.157912605
8605	EPN2	0.015566997	0.719411619
8606	FUT8	-0.024453829	0.572495449
8607	NDUFAF7	0.186773635	1.37E-05
8608	FAM117B	-0.377118248	1.59E-19
8609	HERC6	-0.356497368	1.78E-17
8610	ALG12	-0.042028552	0.331907823
8611	CHCHD7	-0.191202391	8.45E-06
8612	C8orf58	0.406617759	1.02E-22
8613	CCL4L1	0.118156049	0.006216165
8614	ST3GAL3	0.09752244	0.024082915
8615	TOP3A	0.186248915	1.45E-05
8616	NLE1	0.096758098	0.025218444
8617	TPSB2	0.102834566	0.017344715
8618	DDI2	-0.34445308	2.39E-16
8619	SCN1B	0.218534747	3.31E-07
8620	NAP1L5	-0.226609335	1.17E-07
8621	BRPF1	0.198199447	3.85E-06
8622	PIGN	-0.350485824	6.58E-17
8623	HBS1L	-0.432634849	8.19E-26
8624	CTC1	0.184328008	1.78E-05
8625	ZBED8	-0.003119056	0.942621472
8626	IGLL5	0.184740727	1.71E-05
8627	ANKRD49	0.162285512	0.000163304
8628	CSAD	0.447999502	9.03E-28
8629	UTP23	-0.401402823	3.95E-22
8630	WDR53	0.367803431	1.40E-18
8631	AP1G2	0.513067596	2.94E-37
8632	XRCC4	-0.233502664	4.65E-08
8633	LZTFL1	-0.532561127	1.61E-40
8634	NTPCR	-0.148996785	0.000545085
8635	PRIMPOL	-0.158712859	0.000227949
8636	PELI3	0.002944978	0.945818773
8637	HCFC2	-0.444102026	2.90E-27
8638	FAM98C	0.55289393	3.69E-44
8639	ANKRD28	-0.397499415	1.07E-21
8640	SUCNR1	-0.195275125	5.36E-06

8641	MAGI1	-0.322167136	2.19E-14
8642	TMEM42	0.370641057	7.26E-19
8643	GTF2H5	0.077551748	0.073087879
8644	PHLDA2	0.524920584	3.23E-39
8645	STEAP4	-0.168404665	9.07E-05
8646	RACGAP1	-0.010864041	0.802041693
8647	RP9	0.439114328	1.26E-26
8648	RREB1	-0.171634468	6.60E-05
8649	PLLP	-0.081532614	0.059486236
8650	PHF12	0.292522313	5.14E-12
8651	SLC5A9	0.135015458	0.00174833
8652	TAF3	-0.259824463	1.06E-09
8653	PDE1A	-0.203103748	2.18E-06
8654	WHAMM	0.259226283	1.16E-09
8655	CERS6	-0.466347145	3.04E-30
8656	CLDN15	0.528809597	7.08E-40
8657	C19orf71	0.079017607	0.067810503
8658	ZNF611	-0.31878213	4.22E-14
8659	FOXK2	0.416791366	6.76E-24
8660	TBCEL	-0.394007587	2.59E-21
8661	PTHLH	0.103925669	0.016185752
8662	LARS2	-0.349220057	8.65E-17
8663	ZFC3H1	0.122938875	0.004403046
8664	CYB561D1	-0.149677302	0.000513651
8665	ADARB1	-0.14881781	0.000553643
8666	LACC1	-0.269894897	2.21E-10
8667	TMEM86A	-0.1179063	0.006327071
8668	GATS	-0.016493431	0.703479484
8669	C17orf75	-0.030620793	0.479709327
8670	RBM7	-0.48694131	3.34E-33
8671	ZNF697	-0.325688051	1.10E-14
8672	NME5	-0.141780929	0.001007736
8673	DENND3	0.221685461	2.22E-07
8674	METTL2A	0.022843398	0.598053114
8675	ING3	-0.406591184	1.03E-22
8676	FAM109B	0.181460864	2.42E-05
8677	FNTB	0.208000762	1.22E-06
8678	ZNF174	0.088105427	0.041641951
8679	TMEM199	0.300725081	1.21E-12
8680	EXOC6B	-0.460528692	1.92E-29
8681	TMEM130	-0.179815218	2.87E-05
8682	ZFP1	-0.485461737	5.54E-33
8683	RHPN1	0.541373331	4.56E-42
8684	DENND2A	0.002370993	0.956367079
8685	FADS1	0.064935187	0.133607759
8686	RNASEL	-0.373353513	3.86E-19
8687	KIAA1161	-0.325509921	1.14E-14
8688	RARRES1	0.05998905	0.165883325
8689	NECAB3	0.267737444	3.10E-10
8690	RAB33B	-0.363225333	3.96E-18
8691	TXNDC16	-0.426645008	4.46E-25
8692	UQCC2	0.539568663	9.54E-42
8693	RUNX1	0.001905257	0.964931756
8694	GON4L	0.070350404	0.104073541
8695	FBXO32	-0.150242163	0.000488848
8696	FLCN	0.261390208	8.31E-10
8697	JARID2	-0.03362382	0.437677184
8698	ANK2	-0.096068384	0.026282474

8699	SST	0.003586176	0.934046557
8700	NR2C2	-0.198477297	3.73E-06
8701	ZNF318	-0.164030864	0.000138398
8702	PYROXD1	-0.327850846	7.16E-15
8703	TRIM68	-0.321075929	2.71E-14
8704	ADA	0.418407885	4.35E-24
8705	IPMK	-0.267934958	3.01E-10
8706	TRIM15	0.199736969	3.22E-06
8707	BMF	0.040939694	0.344597984
8708	TPSAB1	0.069952141	0.106052476
8709	PPOX	0.566507468	9.74E-47
8710	TAF5L	-0.090802068	0.035756594
8711	SUPT20H	0.158749189	0.000227185
8712	ITGA4	-0.266253646	3.92E-10
8713	AMIGO2	-0.157041182	0.000265808
8714	INTS8	0.129538944	0.002683058
8715	WWOX	-0.059252634	0.17115112
8716	MYNN	-0.202459412	2.35E-06
8717	ZNF319	-0.033338401	0.441578892
8718	RPS6KC1	-0.1817154	2.35E-05
8719	LRIF1	-0.17460855	4.90E-05
8720	CARD6	-0.253049232	2.92E-09
8721	GMDS	0.07099646	0.100925683
8722	TATDN1	-0.330972083	3.84E-15
8723	BMP4	-0.112118158	0.009447466
8724	ZNHIT6	-0.267413737	3.26E-10
8725	TSC22D2	-0.02098582	0.628157782
8726	LIPC	-0.026976281	0.533536188
8727	HLCS	-0.332765066	2.67E-15
8728	ASAP3	-0.084189141	0.05163047
8729	POPDC2	0.060793861	0.160266404
8730	CRYM	0.069293313	0.109391334
8731	APIP	-0.23541822	3.59E-08
8732	FAM89A	-0.00380284	0.930071787
8733	LOXL4	-0.096969303	0.024900143
8734	C9orf3	0.110214225	0.0107391
8735	UNC119	0.279638162	4.56E-11
8736	NTHL1	0.541118823	5.07E-42
8737	CD1D	0.143194977	0.000895367
8738	NVL	0.16179299	0.000171058
8739	AGO4	0.001096591	0.979811769
8740	CACNA1H	0.065159503	0.132267922
8741	KRR1	-0.490583914	9.54E-34
8742	ASB1	-0.140999638	0.001075271
8743	ABHD5	-0.364955945	2.68E-18
8744	CYP3A5	0.160836953	0.000187107
8745	TARBP1	0.286682762	1.40E-11
8746	GPHN	-0.278949715	5.10E-11
8747	HMGB3	0.075814285	0.079772072
8748	RNF219	-0.178864606	3.17E-05
8749	KDM7A	-0.338569094	8.16E-16
8750	PDE4D	-0.328446675	6.36E-15
8751	WDR83	0.692685673	1.13E-77
8752	ARL6	-0.457450668	5.02E-29
8753	MKNK1	0.291163557	6.50E-12
8754	PREX2	-0.123345597	0.004273469
8755	WSCD1	-0.131267713	0.00234775
8756	FOCAD	-0.344494892	2.37E-16

8757	PRRG1	-0.522037219	9.85E-39
8758	PPP1R13B	-0.075640277	0.080467952
8759	CYB561A3	0.081703507	0.058952717
8760	ATXN7	-0.175280546	4.57E-05
8761	ZBTB46	0.122623116	0.00450608
8762	RNF144A	-0.225089253	1.43E-07
8763	GID4	-0.351103359	5.76E-17
8764	ME1	-0.211052026	8.40E-07
8765	JAM2	-0.198887945	3.55E-06
8766	HSPB2	0.260617643	9.35E-10
8767	CTF1	0.016370128	0.705592661
8768	ZBTB21	-0.37709223	1.60E-19
8769	CMSS1	0.107755717	0.012637224
8770	PLAGL2	-0.156436603	0.000280892
8771	PXK	-0.355174719	2.37E-17
8772	MDM4	0.265053485	4.72E-10
8773	HHEX	-0.050642409	0.242253397
8774	MYSM1	-0.051845523	0.231232187
8775	CCDC186	-0.524259388	4.18E-39
8776	ZNF14	-0.397475013	1.08E-21
8777	MAPK8	-0.342566055	3.55E-16
8778	RICTOR	-0.324847086	1.30E-14
8779	PDE5A	-0.297054027	2.32E-12
8780	RC3H1	-0.278566911	5.44E-11
8781	MAP7D3	-0.098683311	0.022442907
8782	ZNF576	0.249058424	5.23E-09
8783	FGD6	-0.043552533	0.314659058
8784	KATNAL1	-0.311997624	1.53E-13
8785	MAK16	-0.070394179	0.103857823
8786	ZNF211	0.030366065	0.483372951
8787	DOCK7	-0.441232349	6.76E-27
8788	TUT1	0.624774294	2.93E-59
8789	PHC3	-0.392216877	4.05E-21
8790	RAB11FIP1	-0.498694962	5.53E-35
8791	TMEM25	-0.253984703	2.54E-09
8792	SCO1	-0.241250611	1.60E-08
8793	ESPN	0.030083037	0.487461294
8794	TRAF1	0.507325439	2.46E-36
8795	PGGT1B	-0.464020929	6.39E-30
8796	SORD	-0.305044828	5.52E-13
8797	RNF168	-0.215252229	5.00E-07
8798	KLRB1	0.185120977	1.64E-05
8799	CDADC1	-0.254293703	2.43E-09
8800	BDP1	-0.285706892	1.65E-11
8801	ABCG2	-0.315393135	8.05E-14
8802	ZNF248	-0.016902643	0.696482801
8803	NRROS	0.034113397	0.431030887
8804	IL34	0.282425635	2.87E-11
8805	KMO	-0.052200086	0.228053034
8806	PKD1	0.312451334	1.40E-13
8807	ANKS6	-0.010809959	0.803006878
8808	HIST1H4I	0.123591644	0.004196765
8809	ZNF486	-0.199150914	3.45E-06
8810	AR	-0.325443606	1.15E-14
8811	MTX3	-0.378823542	1.06E-19
8812	GMPR	-0.155433239	0.000307703
8813	PRDM2	-0.232109834	5.62E-08
8814	LARP6	0.091180688	0.034989812

8815	ARHGEF25	0.252402135	3.21E-09
8816	AP5M1	-0.491635681	6.62E-34
8817	CCL18	-0.029851772	0.490815674
8818	ZNF641	-0.150904402	0.000461189
8819	KLF12	-0.443635779	3.32E-27
8820	PHF6	-0.377281934	1.53E-19
8821	DNAAF2	-0.205419316	1.66E-06
8822	CELF2	-0.139294737	0.001237377
8823	AVL9	-0.289097863	9.28E-12
8824	FKTN	-0.400012984	5.64E-22
8825	DCAF13	0.011363707	0.793138731
8826	KIAA0922	-0.199818015	3.19E-06
8827	ZNF827	-0.319217883	3.88E-14
8828	BMP6	-0.199891778	3.17E-06
8829	FAM49A	-0.146876675	0.00065484
8830	RASA2	0.011305835	0.794168534
8831	RFC4	0.465294714	4.26E-30
8832	CAPN12	0.452753122	2.13E-28
8833	RBAK	-0.314879113	8.88E-14
8834	ZNF45	-0.357364191	1.47E-17
8835	ZNF574	0.3494584	8.22E-17
8836	B3GNT3	0.058019708	0.180248653
8837	TMED3	0.258102157	1.37E-09
8838	C17orf59	0.373979465	3.34E-19
8839	MBP	-0.466735871	2.69E-30
8840	GCHFR	0.126101533	0.003482548
8841	BBS7	-0.391771752	4.52E-21
8842	RPUSD2	0.221500538	2.27E-07
8843	ANXA13	0.016252545	0.707609928
8844	HOXC6	0.357880343	1.31E-17
8845	C12orf5	-0.137481066	0.001434304
8846	TTC32	0.316868979	6.08E-14
8847	EPC1	-0.101082194	0.019358123
8848	DTD2	-0.293736494	4.16E-12
8849	DST	-0.327220422	8.12E-15
8850	TERF1	-0.358345089	1.18E-17
8851	PTPRE	-0.06083422	0.159988559
8852	IQSEC2	0.344318852	2.45E-16
8853	ZCCHC9	0.10826017	0.012225213
8854	THADA	-0.170162748	7.64E-05
8855	RASD2	0.04489652	0.299944071
8856	TOP1MT	0.480695588	2.77E-32
8857	NPR2	0.154196471	0.000344037
8858	PPP1R12B	-0.286618963	1.42E-11
8859	PNMAL1	-0.148938452	0.000547861
8860	SIGLEC10	0.18269315	2.12E-05
8861	GNG7	-0.255701956	1.97E-09
8862	RECQL5	0.485772375	4.98E-33
8863	VDR	-0.052056119	0.229340136
8864	HSPA4L	-0.563881808	3.13E-46
8865	STRIP1	0.092642728	0.032158918
8866	AMPD3	-0.237942716	2.53E-08
8867	BRD9	0.453347939	1.78E-28
8868	CEP95	0.371012021	6.66E-19
8869	RBSN	-0.347781733	1.18E-16
8870	GLIS1	0.177046872	3.82E-05
8871	ACOX2	-0.133107612	0.002033289
8872	ENDOG	0.209612125	1.00E-06

8873	HELZ2	0.155682864	0.000300818
8874	POLR1A	-0.054776005	0.205885993
8875	TIFA	-0.199163671	3.44E-06
8876	SLC25A29	0.368978688	1.07E-18
8877	ZNF548	-0.089184685	0.039195025
8878	MTA3	-0.117475756	0.006522438
8879	ARID2	-0.347712039	1.19E-16
8880	DBT	-0.585617905	1.44E-50
8881	ZNF331	-0.154390227	0.000338092
8882	RNF31	0.016655704	0.700701917
8883	R3HDM1	-0.09036944	0.036650205
8884	NYNRIN	-0.201318379	2.68E-06
8885	ZMAT1	0.194430163	5.90E-06
8886	CLYBL	-0.154231966	0.000342941
8887	PDZD2	-0.298500698	1.80E-12
8888	LPAR2	0.466395106	3.00E-30
8889	OSBPL10	-0.26903103	2.53E-10
8890	CAB39L	-0.411561132	2.76E-23
8891	KLF5	-0.138278862	0.001344377
8892	TRAPPC13	-0.258119235	1.37E-09
8893	RDH13	-0.023074176	0.594359099
8894	HLX	0.257035086	1.61E-09
8895	PSMA6	-0.138732262	0.001295611
8896	MRPL42	-0.314435133	9.65E-14
8897	PVRL3	-0.379056896	1.00E-19
8898	CDC14B	-0.407580283	7.91E-23
8899	ZNF251	0.360691498	6.99E-18
8900	SLC25A22	0.635920974	5.76E-62
8901	FBXO4	0.030099244	0.487226679
8902	TAF6L	0.49378861	3.13E-34
8903	PALMD	-0.221376228	2.31E-07
8904	FAIM3	0.150888151	0.00046185
8905	PTPRN2	-0.082580824	0.056275791
8906	KIT	-0.370359375	7.75E-19
8907	FMO3	-0.087056627	0.044142153
8908	TECPR1	0.485780296	4.97E-33
8909	CIART	0.119018741	0.005846371
8910	PRR3	0.350123539	7.12E-17
8911	DCLRE1A	-0.412567157	2.11E-23
8912	DHX57	-0.165024087	0.000125866
8913	ZNF329	-0.299801337	1.42E-12
8914	ACSS3	-0.520358546	1.87E-38
8915	WDR70	0.018481925	0.669725537
8916	PPAPDC2	-0.072834842	0.092380861
8917	KCNAB2	0.305848911	4.77E-13
8918	NSUN4	-0.054112954	0.21143677
8919	DGCR14	0.627591752	6.21E-60
8920	RAD1	-0.214092137	5.78E-07
8921	MIB2	0.49077994	8.91E-34
8922	CDYL2	-0.26789493	3.03E-10
8923	SS18L1	0.113231261	0.008758074
8924	ZNF646	0.050535227	0.243252817
8925	WEE1	-0.158796931	0.000226185
8926	NXN	-0.037911205	0.38149102
8927	CEBPA	0.137862376	0.001390655
8928	C4orf19	-0.375222922	2.49E-19
8929	EBF4	0.13950398	0.001216336
8930	L3HYPDH	0.388899144	9.22E-21

8931	CBFA2T2	0.003002026	0.944770872
8932	CDK8	-0.070752263	0.102106504
8933	ACAT2	0.032130384	0.458310836
8934	ATXN1	-0.349331398	8.44E-17
8935	TMLHE	-0.518289652	4.12E-38
8936	ACACB	-0.093540702	0.030519104
8937	TIMELESS	0.241010375	1.65E-08
8938	LIG4	-0.158905133	0.000223934
8939	NEURL4	0.422714725	1.33E-24
8940	POGLUT1	-0.034188345	0.430018595
8941	GLDC	-0.058308374	0.178087175
8942	SLC12A2	-0.448325923	8.18E-28
8943	ENGASE	0.499658454	3.92E-35
8944	TPPP	-0.197778957	4.04E-06
8945	NFATC3	-0.439727324	1.05E-26
8946	CREB5	-0.034255857	0.429107932
8947	RFT1	-0.080625735	0.062384711
8948	PAQR8	-0.193855002	6.29E-06
8949	FAM65C	0.005714619	0.895085983
8950	TTC14	0.210923602	8.53E-07
8951	CCDC61	0.490658958	9.29E-34
8952	SLC2A13	-0.445642975	1.83E-27
8953	IDH3A	-0.411032069	3.17E-23
8954	UTP20	-0.365685716	2.27E-18
8955	CXorf40A	-0.01918816	0.657891514
8956	GRB14	-0.127078056	0.003235873
8957	FAN1	-0.444103576	2.89E-27
8958	UBE2C	0.534775035	6.63E-41
8959	RASEF	-0.257846313	1.42E-09
8960	ZNF703	0.29192255	5.70E-12
8961	ATP7A	-0.623759367	5.10E-59
8962	TMEM143	0.241453997	1.55E-08
8963	TIMD4	-0.022590786	0.602108516
8964	E4F1	0.734488343	8.49E-92
8965	HERC2	-0.225435246	1.36E-07
8966	ZNF544	-0.094624175	0.028636356
8967	MFSD7	0.033619293	0.437738912
8968	TECPR2	-0.192720974	7.14E-06
8969	CACNA2D1	-0.027178336	0.530474228
8970	ARIH1	-0.467048114	2.43E-30
8971	RORA	-0.425538407	6.09E-25
8972	ANAPC4	0.172353316	6.14E-05
8973	SP140L	0.28980885	8.21E-12
8974	NARFL	0.536776984	2.96E-41
8975	TMTC4	-0.155463365	0.000306864
8976	TRMT10B	0.17978774	2.88E-05
8977	CAMK1D	-0.281550925	3.32E-11
8978	UBAP2	-0.006429253	0.882056482
8979	FAIM	-0.16798675	9.45E-05
8980	TBKBP1	0.318968	4.07E-14
8981	C1QTNF3	-0.135299447	0.001709193
8982	LRRC1	-0.281370626	3.42E-11
8983	DHX34	0.671129091	2.69E-71
8984	GPT2	-0.030298272	0.484350527
8985	CPSF2	-0.287848518	1.15E-11
8986	KLHDC9	0.245719766	8.47E-09
8987	ARHGAP32	-0.235844942	3.38E-08
8988	MKL2	-0.479169687	4.62E-32

8989	ZNF444	0.574434203	2.70E-48
8990	TNIK	-0.185645865	1.55E-05
8991	TMC6	0.48388841	9.45E-33
8992	ZNF253	-0.514167463	1.95E-37
8993	EGR2	-0.079748763	0.065297426
8994	STK11IP	0.403072549	2.56E-22
8995	FBXO27	-0.020630811	0.633984194
8996	TMCO6	0.501260465	2.21E-35
8997	POLI	-0.070422325	0.10371931
8998	LZTS3	-0.063803204	0.140529375
8999	NR2C1	0.221840625	2.17E-07
9000	OXER1	0.472945522	3.61E-31
9001	RPL39L	0.091503334	0.034347477
9002	SAMSN1	0.037278387	0.389494784
9003	COA1	-0.024317515	0.574638605
9004	SEC22A	-0.023061955	0.594554454
9005	MLH3	-0.011162179	0.79672636
9006	CXCL1	0.231253745	6.31E-08
9007	WDR3	-0.383329373	3.60E-20
9008	PPIP5K2	-0.308100953	3.15E-13
9009	MFSD11	-0.011903408	0.783552586
9010	MBTD1	-0.228426076	9.20E-08
9011	CLCN4	-0.338501999	8.27E-16
9012	CCDC134	-0.279520478	4.65E-11
9013	MPPE1	-0.090476675	0.036426957
9014	TRIM24	-0.295236777	3.20E-12
9015	ATG2B	-0.291420408	6.22E-12
9016	SMIM13	-0.217244716	3.90E-07
9017	PLCD1	-0.067481439	0.119001855
9018	THAP3	0.486685269	3.65E-33
9019	TBC1D19	-0.423373444	1.11E-24
9020	WDR19	0.05396983	0.212648974
9021	DDR2	-0.244152266	1.06E-08
9022	BAIAP2	0.195979512	4.95E-06
9023	PLXNA3	0.355277651	2.32E-17
9024	MRE11A	-0.161340063	0.000178493
9025	GATA2	-0.109703272	0.011111364
9026	TAF8	0.041925709	0.333093378
9027	PARD6G	-0.086843577	0.044665182
9028	LAT2	0.270604465	1.97E-10
9029	MTFMT	-0.248085092	6.03E-09
9030	ZNF844	-0.442900581	4.13E-27
9031	HOXB8	-0.044255864	0.306900426
9032	DNAJA4	-0.216252788	4.42E-07
9033	CEP89	0.410506276	3.65E-23
9034	ZNF397	-0.026294061	0.543939967
9035	PIK3C3	-0.44066517	7.99E-27
9036	HEPH	-0.168500672	8.99E-05
9037	TMEM138	0.556599103	7.54E-45
9038	TCP11L1	-0.117487779	0.00651691
9039	MEDAG	0.036704679	0.396838246
9040	CCDC74A	0.444949935	2.25E-27
9041	MAD1L1	0.424962748	7.15E-25
9042	SLC37A1	0.10604647	0.014125808
9043	SELP	-0.221155021	2.37E-07
9044	DDX10	-0.043972473	0.310011255
9045	GALK2	-0.357218711	1.51E-17
9046	GZMB	0.442385674	4.81E-27

9047	MASTL	-0.006120564	0.887680933
9048	ZNF121	0.282841904	2.68E-11
9049	S100A2	0.136684178	0.00152962
9050	ADAMTS2	0.087862421	0.042210345
9051	MYPOP	0.591785948	7.39E-52
9052	JMJD4	0.453682701	1.61E-28
9053	CHCHD6	0.268304108	2.84E-10
9054	NUDT3	-0.301604625	1.03E-12
9055	ARSK	-0.405729841	1.28E-22
9056	PLA2G4C	0.234888826	3.85E-08
9057	FAM69A	-0.157872094	0.00024631
9058	TAB3	-0.455501481	9.18E-29
9059	SLC23A1	-0.116064708	0.007201216
9060	C12orf65	0.376085588	2.03E-19
9061	UHRF2	0.049787037	0.250309888
9062	THOP1	0.595341105	1.29E-52
9063	CCRN4L	0.031426487	0.468221553
9064	GEMIN6	0.45064482	4.06E-28
9065	PCED1B	0.337799718	9.56E-16
9066	SSX2IP	-0.174569248	4.92E-05
9067	FBXL6	0.790498473	1.43E-115
9068	THEM5	0.024845824	0.566353632
9069	KLHL23	-0.157914236	0.000245358
9070	C1orf109	-0.204971838	1.75E-06
9071	MFSD4	-0.319557612	3.63E-14
9072	RRP12	0.365592604	2.31E-18
9073	KBTBD4	-0.454870465	1.12E-28
9074	FRK	-0.616512238	2.53E-57
9075	TLK2	-0.096037325	0.026331286
9076	JADE3	-0.351826206	4.93E-17
9077	SLMAP	-0.445051883	2.18E-27
9078	FGF2	-0.224778762	1.49E-07
9079	CRACR2B	0.462784221	9.45E-30
9080	BBS9	-0.324167524	1.48E-14
9081	UGT3A1	-0.033090746	0.444980399
9082	NINL	0.101471903	0.018893556
9083	USP53	-0.543197104	2.16E-42
9084	NFKBIZ	0.374382447	3.04E-19
9085	SAMD10	0.529005275	6.56E-40
9086	SLC38A7	0.263133763	6.36E-10
9087	RAD9A	0.630477867	1.25E-60
9088	ZSCAN29	-0.103376399	0.016760413
9089	PEAK1	-0.327508345	7.67E-15
9090	RASAL2	-0.260979512	8.85E-10
9091	ZNF37A	-0.156995347	0.000266925
9092	RASAL3	0.482381898	1.57E-32
9093	BST1	0.102382487	0.017845782
9094	TTC23	-0.098205972	0.023105159
9095	RECK	-0.307010299	3.85E-13
9096	BRWD1	-0.247003795	7.05E-09
9097	TNK2	0.482618436	1.45E-32
9098	SETBP1	-0.446350073	1.48E-27
9099	CASP9	0.306391789	4.32E-13
9100	NCKAP5L	0.472746685	3.85E-31
9101	POLR3E	0.276779503	7.29E-11
9102	FAM46C	-0.165015491	0.00012597
9103	A1CF	-0.190752905	8.89E-06
9104	ETV7	0.247758559	6.32E-09

9105	PTGER4	-0.127715502	0.003083529
9106	ERN1	0.003279339	0.93967836
9107	PTPN21	-0.277938345	6.03E-11
9108	SOCS2	-0.14884249	0.000552456
9109	GPC3	-0.101377207	0.019005538
9110	NOL9	0.009634403	0.824058311
9111	TASP1	-0.255498716	2.03E-09
9112	PEX11G	0.272776103	1.39E-10
9113	TRIM6	-0.168870805	8.67E-05
9114	TRMT5	-0.363326563	3.87E-18
9115	KBTBD6	-0.327965616	7.00E-15
9116	GCSH	-0.095949427	0.02646985
9117	ACSF2	0.124894404	0.003811006
9118	TRIM41	0.341614555	4.33E-16
9119	SYNE1	-0.223987362	1.65E-07
9120	ANAPC15	0.157344516	0.000258529
9121	EPG5	-0.30703532	3.84E-13
9122	ZNF579	0.620166402	3.58E-58
9123	ABCC5	0.096179212	0.026108934
9124	POLD1	0.584066329	3.02E-50
9125	ALS2	-0.222410042	2.02E-07
9126	LAD1	-0.002383476	0.956137591
9127	ZNF264	-0.286741123	1.39E-11
9128	MFSD9	-0.441158843	6.91E-27
9129	TMEM119	0.044560552	0.303578893
9130	SOCS7	-0.260866528	9.00E-10
9131	ACCS	0.40392056	2.06E-22
9132	TEP1	0.135722281	0.001652408
9133	PPP1R1A	0.141674791	0.001016675
9134	PDS5B	-0.432093932	9.56E-26
9135	ZBTB6	-0.487470989	2.79E-33
9136	EMILIN2	0.222662421	1.95E-07
9137	LINGO1	0.086409154	0.04574784
9138	ANKS4B	-0.057350489	0.185334293
9139	C10orf128	-0.044534683	0.303859968
9140	DDX52	-0.185851882	1.52E-05
9141	CSNK1G1	-0.444769566	2.37E-27
9142	HOMEZ	-0.268469086	2.76E-10
9143	ZNF18	0.457750715	4.57E-29
9144	PTRH2	0.592228899	5.95E-52
9145	IDUA	0.660126182	3.03E-68
9146	C12orf43	0.076922291	0.075454643
9147	ANKRD13D	0.684721489	2.98E-75
9148	ZNF671	-0.104641314	0.015462975
9149	STK19	0.270869233	1.89E-10
9150	PSMA2	0.136134068	0.001598775
9151	FKRP	0.298272812	1.87E-12
9152	HNF1A	-0.004949333	0.909070256
9153	C10orf2	0.207810514	1.24E-06
9154	CLDN23	-0.094973576	0.028050869
9155	LMCD1	0.066768142	0.122961611
9156	RNF123	-0.175282346	4.57E-05
9157	NKRF	0.042361844	0.328084442
9158	NKTR	0.215818419	4.66E-07
9159	PLCB4	-0.060134017	0.164860819
9160	ZNF28	-0.507163491	2.61E-36
9161	ZNF41	-0.396570164	1.36E-21
9162	SENP1	-0.244338295	1.03E-08

9163	APBA3	0.636046567	5.36E-62
9164	RBM48	-0.181984446	2.29E-05
9165	PPP1R3D	-0.465691096	3.75E-30
9166	RSG1	-0.255651104	1.98E-09
9167	XPO4	-0.506859618	2.91E-36
9168	ZNF335	0.576380399	1.10E-48
9169	ATP5S	-0.242108463	1.42E-08
9170	CBLB	-0.032547042	0.452500111
9171	PIGB	-0.280888227	3.71E-11
9172	LRG1	0.022590818	0.602107992
9173	PEX26	0.013421463	0.756768425
9174	RAPGEF4	-0.263042018	6.45E-10
9175	MRRF	0.146388519	0.000682859
9176	TBXA2R	0.082350194	0.056969533
9177	ABI2	-0.337062954	1.11E-15
9178	FAM217B	-0.324377654	1.42E-14
9179	TXNRD2	0.257449759	1.51E-09
9180	SRGAP2	0.127492632	0.003136035
9181	NUP35	0.044710725	0.301950585
9182	EXOC6	-0.466335958	3.05E-30
9183	RPAIN	0.272939766	1.36E-10
9184	PRMT9	-0.373471516	3.76E-19
9185	PXYLP1	-0.045584315	0.292593466
9186	IMMP1L	0.149875734	0.000504807
9187	PLD1	-0.276008741	8.26E-11
9188	USP8	-0.549466579	1.58E-43
9189	KIF3A	-0.245332502	8.96E-09
9190	HMBS	0.428135279	2.94E-25
9191	DGKQ	0.505465028	4.84E-36
9192	HECW2	-0.32556496	1.13E-14
9193	METTL10	-0.195181879	5.42E-06
9194	MNAT1	-0.233699989	4.53E-08
9195	RIC8B	-0.205971038	1.55E-06
9196	SMC6	0.013741562	0.7511565
9197	CTDP1	0.353063005	3.77E-17
9198	C19orf68	0.181209866	2.48E-05
9199	NFATC2	-0.058859997	0.174010324
9200	IQCK	-0.290168931	7.72E-12
9201	MID2	-0.421117854	2.07E-24
9202	ZNF131	-0.041018908	0.343664502
9203	BICD1	-0.080590825	0.062498575
9204	TFB1M	0.160924798	0.000185575
9205	ZNF267	-0.070484405	0.103414317
9206	VMO1	0.465868615	3.55E-30
9207	ZNF48	0.237945871	2.53E-08
9208	CCDC88A	-0.088293595	0.041206275
9209	PIPOX	-0.002300765	0.957658248
9210	ZNF44	-0.038295146	0.376684437
9211	UCK2	0.319015567	4.03E-14
9212	GLI4	0.613719922	1.11E-56
9213	SH3D21	0.298857599	1.68E-12
9214	PITPNM2	0.354135298	2.98E-17
9215	UBOX5	0.048992559	0.257958281
9216	PDE12	-0.404294487	1.87E-22
9217	SH3BGR	-0.05022884	0.246125677
9218	ZNF75D	-0.05210652	0.228888955
9219	TNK1	0.085037795	0.049311151
9220	GCNT2	-0.474505634	2.16E-31

9221	GTPBP3	0.595153585	1.42E-52
9222	SEMA4A	0.233879802	4.42E-08
9223	BEND7	-0.263378708	6.12E-10
9224	DENND6B	0.581203608	1.17E-49
9225	MPV17L	-0.131348401	0.002333078
9226	APOD	0.099409471	0.021467011
9227	C16orf52	-0.335152252	1.65E-15
9228	DIP2A	0.17056994	7.33E-05
9229	C1orf86	0.571777635	9.07E-48
9230	TPX2	0.362387922	4.78E-18
9231	PHKG2	0.673452361	5.87E-72
9232	ENTPD7	-0.224099022	1.62E-07
9233	ABCB8	0.480104882	3.38E-32
9234	ZNF34	0.305981588	4.65E-13
9235	LRRC8C	-0.293120845	4.63E-12
9236	MYL3	-0.0522992	0.227169914
9237	ZNF721	-0.233921239	4.40E-08
9238	ZKSCAN5	-0.111113211	0.010110898
9239	KRBOX4	0.125241641	0.003713759
9240	METTL2B	0.212322289	7.19E-07
9241	TTC30B	-0.357085625	1.56E-17
9242	KCNJ2	-0.110926443	0.010238657
9243	ITGB6	0.00253725	0.953310858
9244	CLASP2	-0.502534274	1.40E-35
9245	FOXP1	-0.204761459	1.79E-06
9246	RWDD2A	-0.166343428	0.000110863
9247	CEP131	0.596969291	5.78E-53
9248	ARVCF	0.221154304	2.37E-07
9249	SPATA24	0.158050127	0.000242311
9250	KIAA1467	-0.493824694	3.09E-34
9251	CSF2RB	-0.06654572	0.124217116
9252	EMG1	0.397643857	1.03E-21
9253	POLR3A	-0.040868006	0.345444166
9254	SDS	0.158424838	0.00023409
9255	HMGN5	-0.12520221	0.003724688
9256	FRA10AC1	-0.026423132	0.541963968
9257	ZBED4	-0.034095116	0.431278013
9258	OTUD7B	-0.297659669	2.08E-12
9259	SMYD4	-0.032001221	0.460120582
9260	FKBP14	-0.188811075	1.10E-05
9261	ZNF346	0.066494998	0.124504821
9262	RNFT1	-0.050858212	0.240249862
9263	KBTBD7	-0.464772602	5.03E-30
9264	NAGPA	0.551310657	7.24E-44
9265	STX11	0.012449882	0.773879403
9266	PLA2G12B	-0.046157897	0.28655649
9267	ZNF764	0.234973059	3.81E-08
9268	C2orf42	0.051847038	0.231218539
9269	WDR5B	0.071019508	0.100814794
9270	AKR1C1	-0.145388496	0.000743761
9271	CBLN3	0.294017072	3.96E-12
9272	GRTP1	0.122642526	0.004499684
9273	OSGEPL1	-0.078232134	0.070598106
9274	HOXC4	0.119389435	0.005693639
9275	GNLY	0.410888173	3.30E-23
9276	GPR155	-0.218519739	3.32E-07
9277	AGO2	-0.151813234	0.000425596
9278	P2RX7	0.031726177	0.463987576

9279	IRGQ	0.071912818	0.096591078
9280	BBC3	0.500977235	2.45E-35
9281	GYLTL1B	-0.008965081	0.836101964
9282	SPATA6	-0.422982549	1.24E-24
9283	NPAT	-0.498478652	5.97E-35
9284	ACAP3	0.593630979	3.00E-52
9285	ZSCAN21	0.161975858	0.00016814
9286	ZNF615	-0.302023717	9.55E-13
9287	DDX55	0.428421564	2.71E-25
9288	EXOC3L4	0.388060593	1.13E-20
9289	RRP8	0.183812025	1.88E-05
9290	CTU2	0.649236036	2.38E-65
9291	C9orf116	0.300363879	1.29E-12
9292	C9orf66	0.007220021	0.867676028
9293	TBCK	-0.29776376	2.05E-12
9294	C3orf33	-0.120742898	0.005165992
9295	INTS7	-0.020129941	0.642243055
9296	PLCL1	-0.369941515	8.54E-19
9297	NGLY1	-0.245941567	8.21E-09
9298	PANK1	-0.330540814	4.19E-15
9299	ZNF182	0.108880138	0.011735143
9300	GPATCH1	0.198469695	3.73E-06
9301	TTC13	0.364801089	2.77E-18
9302	CYP20A1	0.018570189	0.668241987
9303	DHDH	-0.011719441	0.786816603
9304	C1orf56	0.281011232	3.63E-11
9305	DDX20	-0.165403855	0.000121362
9306	STON1	-0.204874688	1.77E-06
9307	UBXN10	-0.335627928	1.49E-15
9308	MICAL2	-0.081983871	0.058086022
9309	ZC3H12A	0.38031841	7.43E-20
9310	EP400	0.02361785	0.585697979
9311	ANK3	-0.218190104	3.46E-07
9312	FAM69B	0.083058057	0.054862518
9313	ULK2	-0.15369349	0.000359928
9314	KAT6B	-0.452692747	2.17E-28
9315	HOXA3	0.250096625	4.50E-09
9316	CD79B	0.247395305	6.66E-09
9317	C21orf91	-0.35058332	6.45E-17
9318	GALNT7	-0.173000253	5.76E-05
9319	MLLT3	-0.211658249	7.80E-07
9320	DOCK10	-0.158431281	0.000233951
9321	MEST	-0.030654134	0.479230929
9322	ATXN2	0.101148754	0.019278081
9323	PLEKHM1	0.082305239	0.057105582
9324	CLEC7A	0.057137662	0.186973614
9325	ZNF689	-0.119110592	0.005808187
9326	GSTM1	0.017794601	0.681321765
9327	C1orf106	-0.013548112	0.75454649
9328	PHTF2	-0.121755436	0.004800521
9329	TCFL5	-0.188597671	1.13E-05
9330	C9orf9	0.245630364	8.58E-09
9331	TRIM52	0.219362017	2.98E-07
9332	NAT6	0.461900986	1.25E-29
9333	ZNF140	-0.066388796	0.125108892
9334	RPSAP58	-0.119221309	0.005762459
9335	GNPDA2	-0.484102366	8.79E-33
9336	PGM5	-0.334555027	1.86E-15

9337	RBMS3	-0.31227281	1.45E-13
9338	CAPN6	-0.030000135	0.488662317
9339	BTBD19	0.333792609	2.17E-15
9340	COG2	-0.19241023	7.39E-06
9341	IRAK2	0.070270854	0.104466462
9342	CDKN2B	-0.099251423	0.021676217
9343	MNS1	-0.168191456	9.27E-05
9344	APOBR	0.328272715	6.59E-15
9345	POLB	0.135319042	0.001706522
9346	TRIOBP	0.281147727	3.55E-11
9347	ANKS3	0.584310841	2.69E-50
9348	NDOR1	0.561958347	7.31E-46
9349	ABTB2	0.013510031	0.755214368
9350	SLC25A26	-0.097175585	0.024592615
9351	SYNJ1	-0.367742749	1.42E-18
9352	MPLKIP	0.074870768	0.083604492
9353	MRPS24	0.234926466	3.83E-08
9354	CSF2RA	0.117320374	0.006594264
9355	AGTR1	-0.133427587	0.001982709
9356	PWWP2B	0.222099291	2.10E-07
9357	HAUS5	0.545186259	9.46E-43
9358	SMAP1	-0.383259006	3.66E-20
9359	PTGES	0.124321286	0.00397657
9360	LSM8	0.309642763	2.37E-13
9361	LIN52	-0.172973862	5.77E-05
9362	LOXL1	0.250540329	4.22E-09
9363	GNA14	-0.197660032	4.09E-06
9364	TTC12	-0.191811429	7.90E-06
9365	SLAMF7	0.182247466	2.22E-05
9366	RPP21	0.62543947	2.03E-59
9367	DBP	0.095157347	0.027747077
9368	RPH3AL	-0.073274293	0.090426586
9369	PIGP	-0.287653435	1.19E-11
9370	CACTIN	0.555844316	1.04E-44
9371	ADORA3	0.031569381	0.466200113
9372	CA4	-0.112372612	0.009285751
9373	PCNXL4	-0.349779286	7.67E-17
9374	RPUSD3	0.471070583	6.64E-31
9375	ATPAF2	0.233231996	4.83E-08
9376	TANGO6	-0.388285721	1.07E-20
9377	GNB5	-0.357039018	1.58E-17
9378	MSANTD2	0.329555322	5.10E-15
9379	BAIAP2L1	0.009062893	0.834339496
9380	DIEXF	-0.317273149	5.63E-14
9381	PAPD5	-0.376965461	1.65E-19
9382	KIAA0753	0.095343785	0.027441772
9383	PRMT7	0.449900345	5.09E-28
9384	RNF180	-0.415898954	8.60E-24
9385	MASP1	-0.252606327	3.11E-09
9386	ACYP1	0.425178638	6.73E-25
9387	SHE	-0.331508229	3.45E-15
9388	DNAL1	-0.36307061	4.10E-18
9389	WDR12	-0.104353043	0.01575064
9390	C15orf48	0.296520635	2.55E-12
9391	MCC	-0.388951935	9.10E-21
9392	CDHR2	-0.039231676	0.365117142
9393	PLA2G7	-0.066158783	0.126424999
9394	VPS13B	-0.360238896	7.74E-18

9395	HDAC8	0.233723583	4.52E-08
9396	TMC8	0.469196709	1.22E-30
9397	ZRANB1	-0.45667244	6.39E-29
9398	RAB15	0.152933484	0.000385243
9399	ATG7	-0.196276125	4.79E-06
9400	PARS2	-0.273098094	1.32E-10
9401	C5orf30	-0.141787892	0.001007152
9402	TLR1	-0.317266416	5.64E-14
9403	TRPC1	0.069170789	0.110021311
9404	RFC3	-0.162892505	0.0001542
9405	RMI1	-0.283956224	2.22E-11
9406	SLC10A2	-0.143729522	0.000855993
9407	LIMD1	-0.199452392	3.33E-06
9408	RIMKLA	-0.265923813	4.12E-10
9409	UGGT2	-0.245766698	8.42E-09
9410	PDE4B	-0.120157334	0.005388615
9411	UCHL3	0.159065184	0.000220642
9412	FGD1	0.108216832	0.012260137
9413	C10orf88	-0.109781893	0.011053349
9414	ZNF350	-0.205968717	1.55E-06
9415	FRAT2	-0.069992113	0.105852524
9416	FAM174B	-0.012189653	0.778481474
9417	UHRF1BP1	-0.394801548	2.12E-21
9418	ALG6	-0.076627034	0.076586111
9419	WDR20	-0.259867522	1.05E-09
9420	CGRRF1	-0.307429149	3.57E-13
9421	ZNF398	-0.260320724	9.79E-10
9422	EFHC1	0.076223837	0.07815346
9423	TMEM206	0.13075136	0.002443656
9424	MCF2L	-0.002920199	0.946273966
9425	CLEC4A	0.080485256	0.062843947
9426	MTF1	-0.418144367	4.68E-24
9427	THSD7A	-0.470475651	8.06E-31
9428	RFFL	-0.070907085	0.101356594
9429	LAMC3	0.020429881	0.637291962
9430	HMBOX1	-0.067586353	0.118427947
9431	ZNF414	0.57299119	5.22E-48
9432	LILRB2	0.327426374	7.79E-15
9433	FLVCR2	0.020000464	0.644385254
9434	MMP11	0.238729932	2.27E-08
9435	ZNF232	0.152499552	0.000400431
9436	DSC2	-0.399279928	6.80E-22
9437	ANAPC10	-0.276294503	7.89E-11
9438	FAM84A	-0.184654465	1.72E-05
9439	SETDB2	-0.231783748	5.87E-08
9440	DPEP1	-0.020224967	0.640672741
9441	C1orf35	0.68283318	1.09E-74
9442	RABL2B	0.397868013	9.75E-22
9443	TUB	-0.204003323	1.96E-06
9444	SLC16A5	-0.074673778	0.084423101
9445	PLXNC1	-0.1333616	0.001993045
9446	SMG9	0.660461377	2.46E-68
9447	BCORL1	-0.011010177	0.799435159
9448	LIN54	-0.457832786	4.46E-29
9449	DUSP10	0.114245504	0.008169321
9450	LIPT1	-0.059167003	0.171771679
9451	MMAA	-0.416640322	7.04E-24
9452	NUDCD2	-0.178849041	3.17E-05

9453	TM9SF1	-0.192458874	7.35E-06
9454	SMAD1	-0.415217478	1.03E-23
9455	ABHD3	-0.060262479	0.163958691
9456	C22orf46	0.226353987	1.21E-07
9457	AP5S1	-0.013763239	0.750776928
9458	CDCA4	0.431438253	1.15E-25
9459	KCNIP4	-0.30770504	3.39E-13
9460	INCENP	-0.088520127	0.040686887
9461	FAM131C	0.156830262	0.000270983
9462	TRMT13	0.142639253	0.00093807
9463	SOX17	-0.042963487	0.321255076
9464	HSD17B7	0.216935617	4.05E-07
9465	PODXL2	0.106160377	0.01402201
9466	SNRNP48	-0.202513791	2.33E-06
9467	SPAG5	0.308606253	2.87E-13
9468	ANKRD42	-0.294661458	3.54E-12
9469	C12orf29	-0.298108849	1.92E-12
9470	NLGN1	-0.204882272	1.76E-06
9471	NEMF	-0.278132417	5.84E-11
9472	METTL15	-0.51838864	3.97E-38
9473	NR3C2	-0.557136874	5.98E-45
9474	SMARCD3	0.320664863	2.93E-14
9475	FRMD4A	-0.137501308	0.001431956
9476	SYNGR1	-0.111860061	0.00961405
9477	NLN	-0.337149934	1.09E-15
9478	PGM2L1	-0.336632573	1.22E-15
9479	KISS1R	0.016524784	0.702942521
9480	ZNF621	0.002462283	0.954688863
9481	PPM1L	-0.450118505	4.76E-28
9482	VAV1	0.218284821	3.42E-07
9483	NUP93	0.218753127	3.22E-07
9484	C9orf156	-0.141391846	0.001040863
9485	URB1	-0.154952475	0.000321379
9486	C11orf98	0.532769525	1.48E-40
9487	PLCB2	0.418601746	4.13E-24
9488	COL4A4	-0.333560752	2.28E-15
9489	ZNF526	0.159885685	0.000204468
9490	DLG5	-0.01267077	0.769979279
9491	C14orf79	0.306171004	4.49E-13
9492	GLTPD2	0.192576217	7.26E-06
9493	ZNF518A	-0.09916012	0.02179788
9494	OSGIN1	0.266044588	4.05E-10
9495	CYP2U1	-0.557903357	4.29E-45
9496	ZNF438	0.196657299	4.59E-06
9497	5-Sep	0.267718802	3.11E-10
9498	DPH7	0.640405327	4.36E-63
9499	KCTD13	0.663349217	3.99E-69
9500	PBX1	-0.416180972	7.97E-24
9501	REPS1	-0.356720791	1.69E-17
9502	C17orf97	0.130809874	0.002432611
9503	FOXRED2	0.02054108	0.635460475
9504	MZF1	0.457876919	4.40E-29
9505	KIAA1211L	-0.072007349	0.096152501
9506	AKAP7	-0.294652431	3.54E-12
9507	THTPA	-0.206940717	1.38E-06
9508	SPATA5L1	0.029856575	0.490745884
9509	LILRB4	0.110480409	0.010549581
9510	HRH1	-0.019503384	0.652636717

9511	ST3GAL5	0.066063295	0.126974524
9512	SMC4	-0.093728271	0.030185772
9513	SLC1A3	-0.089399426	0.038722969
9514	LSM12	-0.129217796	0.002749956
9515	TTC22	-0.013436368	0.756506825
9516	CUL9	0.209873742	9.70E-07
9517	LNP1	0.049800915	0.2501777
9518	GHR	-0.327427151	7.79E-15
9519	ATP7B	-0.23901383	2.18E-08
9520	ABAT	-0.138552545	0.001314743
9521	ZXDB	-0.514153103	1.96E-37
9522	BCL2A1	0.281696596	3.24E-11
9523	ACAN	-0.047424877	0.273520545
9524	FAM204A	-0.211625638	7.83E-07
9525	ENOX2	-0.383029514	3.87E-20
9526	CCDC14	0.358054308	1.26E-17
9527	PARD6A	0.249102112	5.20E-09
9528	PABPC1L	0.538858711	1.27E-41
9529	DHFRL1	-0.333174307	2.46E-15
9530	GMPPB	0.329339152	5.32E-15
9531	ING1	0.010136496	0.815050625
9532	CCDC109B	0.13447698	0.001824803
9533	FANCC	-0.130597235	0.002472968
9534	HVCN1	0.048543188	0.262355187
9535	CHST7	0.057705769	0.182621368
9536	CCDC132	-0.398707823	7.87E-22
9537	SOWAHB	-0.467565308	2.06E-30
9538	MTSS1L	0.085247008	0.048752974
9539	TBC1D12	-0.306090645	4.56E-13
9540	TRANK1	-0.06420357	0.138050534
9541	ZNF462	-0.181035542	2.53E-05
9542	FAM110D	0.054659756	0.206851473
9543	CNKSR3	-0.194942085	5.57E-06
9544	ZNF213	0.420791321	2.27E-24
9545	ZNF138	-0.16604725	0.000114076
9546	C5orf45	0.333397336	2.35E-15
9547	FCER1A	-0.113602686	0.008538218
9548	TOP2A	0.230386488	7.08E-08
9549	C4orf32	-0.197708061	4.07E-06
9550	CIITA	0.148246465	0.000581809
9551	ZSCAN25	-0.129767399	0.002636376
9552	ITGB3	-0.356443594	1.80E-17
9553	ST5	-0.135367389	0.001699949
9554	ZDHHC13	-0.334765175	1.78E-15
9555	RAB4B	0.713270087	2.60E-84
9556	CIB2	-0.040576607	0.348897337
9557	LDLRAD4	-0.281928681	3.12E-11
9558	POLA1	-0.353708463	3.27E-17
9559	MNT	0.268290685	2.84E-10
9560	PKNOX1	-0.13416645	0.001870286
9561	BCO1	-0.115430723	0.007526271
9562	USP27X	-0.192207303	7.56E-06
9563	MED17	-0.208891871	1.09E-06
9564	DHX35	0.162576951	0.000158872
9565	TMEM136	-0.056167763	0.194579719
9566	PPCDC	0.43943497	1.15E-26
9567	PAM16	0.580482747	1.63E-49
9568	IFI27L1	0.47857609	5.63E-32

9569	PRKCQ	-0.110867358	0.010279372
9570	CTB-55O6.8	0.44517553	2.10E-27
9571	ADRA2C	0.232939145	5.02E-08
9572	HRC	0.061937292	0.152534363
9573	SH3RF3	0.15225286	0.000409312
9574	HERC5	-0.250907044	4.00E-09
9575	ZNF823	-0.019155721	0.658433243
9576	SOX7	-0.057435496	0.184682489
9577	INVS	-0.189995941	9.66E-06
9578	DTWD2	-0.502263119	1.54E-35
9579	OPN3	-0.299158906	1.60E-12
9580	APAF1	-0.285518622	1.71E-11
9581	APCDD1	-0.054730158	0.206266372
9582	UBE2T	0.461411259	1.46E-29
9583	COX18	-0.216064602	4.52E-07
9584	EMX1	0.185619537	1.55E-05
9585	P2RY13	-0.158223996	0.000238463
9586	SAMD4A	-0.068892995	0.111460229
9587	DPY19L3	-0.271468393	1.72E-10
9588	BTBD9	-0.425787907	5.68E-25
9589	NBPF15	0.115869458	0.007299959
9590	DMXL2	-0.089157891	0.039254268
9591	PPP1R14D	0.161412329	0.000177287
9592	PVRL1	0.131715859	0.002267312
9593	MTRF1L	-0.327982088	6.98E-15
9594	POU6F1	0.302604107	8.60E-13
9595	ILDR1	-0.414544918	1.24E-23
9596	NME7	-0.18982508	9.84E-06
9597	SMG6	-0.415977836	8.42E-24
9598	COLEC11	0.121639559	0.00484113
9599	MT3	-0.01974055	0.648694432
9600	ADAMTSL3	-0.359144491	9.88E-18
9601	CENPV	0.09473198	0.0284546
9602	DEPDC7	0.103469184	0.016662103
9603	PDE7B	-0.34906932	8.93E-17
9604	DNAJB5	0.208170725	1.19E-06
9605	CEP170	-0.06078191	0.160348757
9606	SAC3D1	0.611092545	4.39E-56
9607	MPP6	-0.128514676	0.002901757
9608	CDK20	0.070948175	0.101158301
9609	ATP8A1	-0.455305933	9.75E-29
9610	ECT2	-0.030256702	0.48495049
9611	PDE6B	0.092912726	0.031658131
9612	CARD11	0.295665446	2.96E-12
9613	ZNF559	-0.127343634	0.00317159
9614	C1QTNF6	0.502764026	1.29E-35
9615	1-Sep	0.557122774	6.01E-45
9616	RGAG4	-0.222412392	2.02E-07
9617	FAM151A	0.030288521	0.484491226
9618	FAM216A	0.07275521	0.092738585
9619	ZSCAN9	0.328362802	6.47E-15
9620	LRRC6	-0.049013793	0.257751783
9621	MTERF4	0.048056691	0.267173256
9622	LARP1B	-0.237769981	2.59E-08
9623	ZNF761	-0.047623928	0.271509862
9624	LRRC17	-0.001307684	0.975926623
9625	ZNF816	-0.388654103	9.80E-21
9626	FOSL1	0.336795022	1.18E-15

9627	OTUD6B	-0.266258555	3.91E-10
9628	GDF6	-0.14714957	0.000639645
9629	EVI5L	0.075861522	0.079584011
9630	TMEM260	-0.25013978	4.47E-09
9631	PROSER2	-0.047922324	0.268514601
9632	DFNA5	0.092473174	0.032476853
9633	TRAPPC2	0.039435956	0.362623732
9634	LRCH3	-0.003539599	0.934901248
9635	MROH1	0.314186811	1.01E-13
9636	SNAPC4	0.491638791	6.61E-34
9637	TRAM1L1	-0.175580847	4.44E-05
9638	CXCL11	0.072497447	0.093904118
9639	LRRCC1	-0.331009347	3.81E-15
9640	ICA1	-0.122970913	0.004392711
9641	HOXB3	0.206785682	1.41E-06
9642	PTTG1	0.520667703	1.67E-38
9643	ZUFSP	-0.121603513	0.004853826
9644	PRKG1	-0.325369721	1.17E-14
9645	NMNAT1	-0.265710465	4.26E-10
9646	ZNF516	-0.278743973	5.28E-11
9647	NEK1	-0.509124434	1.27E-36
9648	MAP3K8	0.244708254	9.79E-09
9649	ZNF432	0.148515459	0.000568387
9650	PPP1R3E	0.188617821	1.12E-05
9651	ZNF766	-0.254490399	2.36E-09
9652	ACACA	-0.158168406	0.000239687
9653	CEP250	0.190304358	9.34E-06
9654	PIFO	-0.201106266	2.75E-06
9655	ZNF841	0.205089524	1.72E-06
9656	CD84	-0.117130926	0.006682795
9657	OGG1	0.269246546	2.45E-10
9658	C2orf44	-0.051693	0.232609389
9659	TTPAL	0.020055906	0.643467602
9660	GBP5	0.173997493	5.21E-05
9661	S100PBP	0.133831781	0.001920464
9662	DOC2A	0.384303888	2.84E-20
9663	RRP15	-0.343002575	3.24E-16
9664	VRK1	-0.056002439	0.195898489
9665	TMOD2	-0.334867371	1.74E-15
9666	DNAJC18	-0.298040923	1.95E-12
9667	SFRP4	0.089320133	0.038896711
9668	NUDT7	-0.318271863	4.65E-14
9669	ZNF343	0.228911778	8.62E-08
9670	KPTN	0.669637115	7.10E-71
9671	EPHA4	-0.28301135	2.60E-11
9672	PSMC1	-0.302789769	8.32E-13
9673	RBM4	-0.109051735	0.011602608
9674	CLEC1A	-0.055682655	0.198467868
9675	RCL1	-0.027078997	0.531978517
9676	PDLIM3	0.074051819	0.087050235
9677	ZFYVE26	-0.197863252	4.00E-06
9678	HOXC8	0.21851247	3.32E-07
9679	C19orf44	0.325954555	1.04E-14
9680	AGMO	-0.108985143	0.011653883
9681	SLC25A51	-0.060285123	0.163800052
9682	HECTD4	-0.099839138	0.0209071
9683	KLHDC4	0.510403199	7.91E-37
9684	VPS33A	0.062617142	0.148073254

9685	CLEC16A	-0.116222772	0.007122156
9686	CD69	0.118406186	0.006106839
9687	CENPQ	-0.310570749	1.99E-13
9688	CRTC1	0.077241355	0.07424728
9689	ZNF616	-0.406509249	1.05E-22
9690	ZSCAN16	0.213037052	6.58E-07
9691	LRWD1	0.631377342	7.53E-61
9692	MEGF6	0.279137	4.95E-11
9693	PPAT	-0.265682301	4.28E-10
9694	ZNF133	0.438922093	1.33E-26
9695	UBXN2A	-0.314402035	9.71E-14
9696	AZI2	-0.518734929	3.48E-38
9697	SALL2	-0.319696666	3.53E-14
9698	FBXL20	-0.245371405	8.91E-09
9699	MAP3K10	0.597023818	5.63E-53
9700	PUS1	0.757761064	6.94E-101
9701	MAML3	-0.345153385	2.06E-16
9702	NHSL1	-0.073967349	0.087412051
9703	C10orf99	0.135618489	0.001666184
9704	MARS2	-0.241393332	1.57E-08
9705	POC1B	-0.528855547	6.95E-40
9706	DTX2	0.669135597	9.83E-71
9707	RABGAP1L	-0.280640833	3.86E-11
9708	TTC26	-0.223757879	1.70E-07
9709	ABHD17C	-0.172756674	5.90E-05
9710	GABPB1	0.031832601	0.462489171
9711	MAPK15	0.360942907	6.61E-18
9712	ZFHX3	-0.409143975	5.24E-23
9713	ARHGAP6	-0.366269176	1.98E-18
9714	DENND5B	-0.402905682	2.68E-22
9715	FAM110B	-0.252988258	2.94E-09
9716	SAMD5	-0.256619979	1.71E-09
9717	SZT2	0.17861082	3.25E-05
9718	PDIK1L	-0.377671962	1.40E-19
9719	TBC1D8B	-0.317026629	5.90E-14
9720	NEK11	-0.089833435	0.037783596
9721	RBP1	0.206376522	1.48E-06
9722	RAP1GAP2	0.021290898	0.623169205
9723	CGN	-0.253617638	2.68E-09
9724	LRRC20	0.168715673	8.80E-05
9725	EMC6	0.202498533	2.34E-06
9726	PALB2	-0.145798958	0.000718179
9727	DNM1	0.297420328	2.17E-12
9728	CCDC34	0.067097168	0.121122506
9729	WASH1	0.534194233	8.36E-41
9730	ENDOV	0.518409452	3.94E-38
9731	APITD1	0.175524435	4.46E-05
9732	EGLN2	0.581973428	8.12E-50
9733	INPP4B	-0.357163754	1.53E-17
9734	C20orf196	0.145417462	0.000741928
9735	EPHB6	0.182952216	2.06E-05
9736	RCAN3	-0.118520386	0.006057503
9737	TTC39B	-0.262041981	7.52E-10
9738	SAAL1	0.181295689	2.46E-05
9739	SNX16	-0.212291048	7.22E-07
9740	PXMP4	-0.214685259	5.37E-07
9741	MED31	0.186319647	1.44E-05
9742	TLDC1	-0.08176519	0.058761123

9743	CCDC24	0.479867096	3.66E-32
9744	LCLAT1	-0.31878772	4.21E-14
9745	TMEM53	0.028943487	0.504108753
9746	PDE4DIP	-0.17872829	3.21E-05
9747	MICALL2	0.592148684	6.19E-52
9748	EHMT1	0.158347531	0.000235764
9749	NKAP	0.017414058	0.687774778
9750	ABCC9	-0.245121178	9.23E-09
9751	SWT1	-0.334691471	1.81E-15
9752	DNAJC9	0.048144446	0.26629971
9753	BCL2L12	0.593423527	3.32E-52
9754	AP1S3	-0.035164014	0.416966866
9755	DHFR	-0.089327802	0.03887988
9756	POLG2	0.401882267	3.49E-22
9757	ZNF558	0.120022048	0.005441261
9758	SLC39A3	0.459062259	3.04E-29
9759	ADCY4	0.199267989	3.40E-06
9760	FIZ1	0.525548719	2.53E-39
9761	WDR4	0.410832531	3.35E-23
9762	DNMT3A	0.23550232	3.54E-08
9763	BDKRB2	-0.187990939	1.20E-05
9764	ZCCHC2	-0.080824394	0.061740009
9765	MYL5	0.516980221	6.77E-38
9766	LNX1	-0.458782228	3.32E-29
9767	PIGW	-0.060729154	0.160712637
9768	BRF2	-0.254583403	2.32E-09
9769	GATA3	0.067954129	0.116433185
9770	BCAS3	-0.247874419	6.21E-09
9771	ZNF354B	0.271285701	1.77E-10
9772	CHST1	0.101565673	0.018783235
9773	MBOAT1	-0.1294156	0.002708575
9774	FICD	-0.006994908	0.871765571
9775	ICE2	-0.468377437	1.59E-30
9776	CEP63	-0.100860332	0.019627025
9777	ZBTB24	-0.340235873	5.77E-16
9778	SLAMF6	0.195961483	4.96E-06
9779	PHF19	0.440879638	7.50E-27
9780	RAB3A	-0.076077789	0.078727577
9781	BATF	0.505167544	5.40E-36
9782	PCDHB14	-0.106675758	0.013560711
9783	ZNF737	-0.414083984	1.40E-23
9784	SLITRK4	-0.13076222	0.002441603
9785	VCPKMT	0.341275386	4.65E-16
9786	PKP2	-0.28399345	2.21E-11
9787	NHS	0.019135948	0.658763538
9788	IFT80	0.095566703	0.027080525
9789	ATG16L2	0.467751379	1.94E-30
9790	THUMPD2	0.230403051	7.07E-08
9791	SMIM1	0.188120554	1.19E-05
9792	INHBA	-0.074611761	0.084682151
9793	ZSWIM5	-0.239337387	2.09E-08
9794	AASDH	-0.411996515	2.45E-23
9795	MTPAP	-0.168735775	8.79E-05
9796	CCBL1	0.181682263	2.36E-05
9797	C2orf74	0.086131761	0.046450618
9798	CCDC84	0.491224947	7.64E-34
9799	ZDHHC21	-0.270198511	2.10E-10
9800	KAZN	-0.24149295	1.54E-08

9801	FAM76B	0.083231266	0.054356932
9802	TLCD1	0.403651503	2.21E-22
9803	POC5	0.217269392	3.89E-07
9804	QTRTD1	-0.159130857	0.000219305
9805	SHQ1	-0.269396011	2.39E-10
9806	DDX31	0.112153463	0.009424879
9807	VNN2	-0.001384743	0.974508497
9808	TMEM218	0.01911625	0.659092637
9809	DCLK2	0.003740356	0.931217901
9810	DET1	-0.27372665	1.20E-10
9811	ZNF614	-0.336095932	1.36E-15
9812	TRMU	0.587472752	5.95E-51
9813	TOX2	0.154691762	0.00032903
9814	INCA1	0.235164323	3.71E-08
9815	NEK3	0.305667769	4.93E-13
9816	FRY	-0.345245927	2.02E-16
9817	MIS18BP1	-0.251132238	3.87E-09
9818	COQ2	-0.192284939	7.50E-06
9819	TULP4	-0.348394035	1.03E-16
9820	WISP1	-0.095496162	0.027194395
9821	FAM73B	0.455117634	1.03E-28
9822	SGTB	-0.428819466	2.42E-25
9823	RBM15	-0.089614009	0.038256078
9824	DOPEY1	-0.254064928	2.51E-09
9825	CHAF1A	0.289083209	9.30E-12
9826	PHTF1	-0.066559971	0.124136376
9827	TSPAN2	-0.087792205	0.042375794
9828	C14orf93	0.148362033	0.000576006
9829	SLC39A4	0.247776316	6.30E-09
9830	SMIM4	0.338403248	8.44E-16
9831	GRAMD1C	-0.317427751	5.46E-14
9832	NR4A3	-0.140783341	0.001094692
9833	WDR90	0.479314448	4.40E-32
9834	PLEKHH2	0.011557897	0.789685864
9835	ZNF429	-0.290167463	7.72E-12
9836	RSAD2	-0.153019432	0.000382299
9837	PAIP2B	-0.374907096	2.68E-19
9838	PHYHD1	-0.117809975	0.006370318
9839	KIAA1586	-0.328360322	6.47E-15
9840	ASTE1	-0.048287627	0.264878648
9841	TOR2A	0.645749576	1.90E-64
9842	C9orf72	-0.018929685	0.66221293
9843	ATOH8	-0.036023855	0.405660057
9844	CMPK2	-0.198328131	3.79E-06
9845	PRR15L	-0.317765044	5.12E-14
9846	KPNA5	-0.271475686	1.72E-10
9847	MTCL1	0.190554934	9.08E-06
9848	PCDHGB7	-0.219017987	3.12E-07
9849	S100A14	0.101323815	0.019068932
9850	BIN3	0.273702262	1.20E-10
9851	CBR3	0.107121759	0.013172357
9852	EAF2	0.024628466	0.569755327
9853	DES	0.064052924	0.138979278
9854	NKIRAS1	-0.50930813	1.19E-36
9855	MDN1	-0.207171346	1.34E-06
9856	SIRT7	0.737676317	5.52E-93
9857	PDLIM4	0.036219129	0.40311792
9858	SUV39H1	0.458227656	3.94E-29

9859	GLCCI1	-0.159160777	0.000218698
9860	ASF1B	0.451512522	3.12E-28
9861	KCTD6	-0.095661263	0.026928527
9862	RPP25	0.240550091	1.76E-08
9863	HID1	0.072205446	0.095238589
9864	BMPR1A	-0.351560321	5.22E-17
9865	ASB9	-0.130383062	0.002514233
9866	C12orf73	0.275423272	9.09E-11
9867	C3orf18	-0.15624572	0.000285818
9868	UMOD	-0.05072098	0.241522584
9869	ZNF136	-0.406032459	1.19E-22
9870	MAP2	-0.326312341	9.72E-15
9871	DOK3	0.466310806	3.08E-30
9872	ZNF326	-0.030787661	0.477317579
9873	SKP2	0.160113776	0.000200172
9874	TACC3	0.616434797	2.64E-57
9875	FBXO22	-0.104801964	0.015304673
9876	ARMCX5	-0.36904919	1.05E-18
9877	NMNAT3	-0.251909157	3.45E-09
9878	ACVR2A	-0.433879382	5.73E-26
9879	SYNDIG1	0.011189651	0.796237044
9880	BRMS1L	-0.455747937	8.51E-29
9881	FAM78A	0.280176698	4.17E-11
9882	NEXN	-0.090644204	0.036080499
9883	CRIPAK	0.275906724	8.40E-11
9884	MRPS18C	-0.188285644	1.16E-05
9885	FCHSD1	0.386254829	1.77E-20
9886	ZSCAN30	0.015876051	0.714082798
9887	SLC37A2	-0.008210867	0.849719072
9888	HPD	0.003537636	0.934937264
9889	INTS2	-0.292407154	5.24E-12
9890	SH2B2	0.514961151	1.45E-37
9891	TPBG	-0.065123199	0.132484062
9892	C2orf81	0.420903713	2.20E-24
9893	PCGF6	-0.056270109	0.193766584
9894	CCDC112	-0.092944785	0.031599113
9895	COL4A3	-0.017834633	0.68064426
9896	CD302	-0.405511784	1.36E-22
9897	TAGAP	-0.087208164	0.043773279
9898	NAA25	-0.045874845	0.289525058
9899	U2AF1L4	0.59306156	3.96E-52
9900	MYEOV	0.060713583	0.160820159
9901	XPNPEP3	0.039618368	0.360406261
9902	TBC1D10A	0.252676884	3.08E-09
9903	CLK3	0.428479944	2.67E-25
9904	B3GNT5	-0.11996108	0.005465138
9905	RASGRF2	-0.298241211	1.88E-12
9906	AAK1	-0.294454843	3.67E-12
9907	RAPGEF3	0.136182558	0.001592566
9908	RFX7	-0.337069284	1.11E-15
9909	ZNF528	0.062120318	0.151323452
9910	F3	0.136245675	0.001584517
9911	SFRP1	-0.115167137	0.007665233
9912	PPM1H	-0.207858486	1.24E-06
9913	MAGOHB	0.238075216	2.49E-08
9914	GBGT1	0.270550617	1.99E-10
9915	SRPX	0.033324103	0.441774863
9916	TUBD1	-0.014229556	0.742626335

9917	CENPC	-0.303380896	7.47E-13
9918	ZFP3	-0.308467125	2.95E-13
9919	NUFIP1	-0.140377693	0.00113199
9920	SERPINB8	-0.053676402	0.215149818
9921	PLEKHF1	0.484389161	7.98E-33
9922	APOBEC3F	0.358802272	1.07E-17
9923	OTUD3	-0.012755546	0.76848396
9924	NAPB	0.068022468	0.116065439
9925	SEMA6C	0.208413619	1.16E-06
9926	CCL19	0.207685163	1.26E-06
9927	MBOAT2	-0.261086732	8.71E-10
9928	TIGD2	-0.337116917	1.10E-15
9929	NOM1	0.099714603	0.021068064
9930	COQ10A	0.168026969	9.42E-05
9931	SETMAR	-0.176152207	4.19E-05
9932	PADI2	0.052639431	0.22415692
9933	ADAMTS10	0.437290755	2.14E-26
9934	DYRK3	-0.148474493	0.000570412
9935	SSBP2	-0.218369367	3.38E-07
9936	GFRA1	-0.062798089	0.146902818
9937	IFT27	0.469075378	1.27E-30
9938	CEP290	0.097639614	0.023912806
9939	MAP4K2	0.159227909	0.000217342
9940	RCCD1	0.440736042	7.83E-27
9941	ROBO1	-0.176473024	4.05E-05
9942	FAM20A	0.24259053	1.32E-08
9943	ZSCAN31	-0.308626035	2.86E-13
9944	ACOT1	-0.039994666	0.35585869
9945	NRXN2	0.196367121	4.74E-06
9946	KLHL26	-0.093202661	0.031127804
9947	FAHD2A	0.405000347	1.55E-22
9948	TXNRD3	-0.142260987	0.000968205
9949	PCDHB16	-0.112415366	0.009258822
9950	NSUN6	0.311789272	1.59E-13
9951	PHC1	0.031708114	0.464242163
9952	RBM12B	-0.220875724	2.46E-07
9953	MARVELD2	-0.377702852	1.39E-19
9954	ZFAT	-0.166472274	0.000109491
9955	LLPH	-0.131280342	0.002345448
9956	BSPRY	-0.290126034	7.77E-12
9957	APOBEC3D	0.448737848	7.23E-28
9958	ZBTB26	-0.132140572	0.002193411
9959	BIRC7	0.016810063	0.698063501
9960	IL1R2	0.016556271	0.702403407
9961	PTGIS	0.083664964	0.05310798
9962	ADAMTSL4	0.240567541	1.76E-08
9963	FBXO45	-0.108898625	0.011720801
9964	PCNT	0.12463388	0.003885477
9965	ESRP2	-0.033398097	0.440761197
9966	HIST1H2AE	0.285499816	1.71E-11
9967	LRRC57	-0.246874706	7.18E-09
9968	NPEPL1	0.596207846	8.43E-53
9969	PDSS1	0.272129454	1.55E-10
9970	THOC1	0.350989338	5.91E-17
9971	COLCA2	0.075847111	0.079641347
9972	SLC51B	0.034992653	0.419242178
9973	KIAA0556	0.087209081	0.043771055
9974	N6AMT1	-0.062137281	0.151211595

9975	KRT80	-0.055981655	0.196064739
9976	KCNMA1	0.040269097	0.352565052
9977	LPHN1	0.01040063	0.810321671
9978	ANKRD6	-0.192461343	7.35E-06
9979	SLFN12	-0.160399029	0.00019492
9980	F10	0.127462629	0.003143165
9981	MEF2BNB	0.46482809	4.94E-30
9982	EVI5	-0.371216911	6.35E-19
9983	BSCL2	-0.089846566	0.037755478
9984	ZNF585B	-0.194302124	5.98E-06
9985	FAM45A	-0.289416616	8.78E-12
9986	NFAM1	0.216355783	4.36E-07
9987	OASL	0.326833663	8.76E-15
9988	ZNF280D	-0.226868324	1.13E-07
9989	SLC35G2	0.115741993	0.007365073
9990	ZNF420	-0.410254081	3.90E-23
9991	RNF169	-0.497286192	9.12E-35
9992	PORCN	0.210199271	9.32E-07
9993	ZNF584	0.097014192	0.02483294
9994	CPED1	-0.290456373	7.34E-12
9995	ZNF227	-0.145380106	0.000744292
9996	ZNF441	-0.326627454	9.13E-15
9997	DONSON	0.469935723	9.60E-31
9998	ZKSCAN4	-0.133836286	0.001919781
9999	TACC2	-0.200980107	2.79E-06
10000	GNA15	0.331345021	3.56E-15
10001	MPZL3	-0.070557462	0.103056306
10002	TMEM185A	-0.1357148	0.001653397
10003	ZNF746	0.288269018	1.07E-11
10004	KATNBL1	0.094981275	0.028038086
10005	MILR1	0.163162333	0.000150308
10006	ZNF808	-0.15206069	0.000416357
10007	PRICKLE3	0.387546124	1.29E-20
10008	PHKA1	-0.147923133	0.000598331
10009	TMEM5	-0.175986145	4.26E-05
10010	TIGD5	0.319519091	3.66E-14
10011	GATB	-0.050492146	0.243655346
10012	LGALS4	0.19345728	6.58E-06
10013	SIGLEC1	0.138649528	0.001304386
10014	SYPL2	-0.086179752	0.046328388
10015	DCLRE1B	-0.06729403	0.120032441
10016	PELI2	-0.405739766	1.28E-22
10017	TIRAP	-0.283622061	2.35E-11
10018	PMS1	0.121745268	0.004804072
10019	REV3L	-0.327281959	8.02E-15
10020	ARMC5	0.539889063	8.37E-42
10021	DNAJC17	0.544353952	1.34E-42
10022	MGA	-0.385135269	2.32E-20
10023	SDR39U1	0.298162519	1.91E-12
10024	NFXL1	-0.373848422	3.44E-19
10025	LYRM9	0.091661848	0.034035601
10026	ZNF202	0.35727054	1.50E-17
10027	CENPW	0.499035995	4.90E-35
10028	RWDD3	0.195170861	5.43E-06
10029	MYEF2	-0.112022755	0.00950874
10030	SEC14L2	0.308286526	3.05E-13
10031	HIVEP1	-0.220125561	2.71E-07
10032	MTMR9	-0.39699899	1.22E-21

10033	AP4B1	0.407830874	7.40E-23
10034	SCARF2	0.25283231	3.01E-09
10035	ZNF43	-0.168782579	8.75E-05
10036	CRABP2	0.26397266	5.58E-10
10037	RNF8	-0.2407455	1.71E-08
10038	ZNF711	-0.069698003	0.107330737
10039	RILPL1	0.321767515	2.37E-14
10040	CXCL8	0.062502212	0.148820351
10041	USP42	-0.141352196	0.001044295
10042	PSMD9	0.297199886	2.26E-12
10043	HAPLN3	0.430449672	1.53E-25
10044	LILRB1	0.250459839	4.27E-09
10045	FXN	0.023045334	0.59482019
10046	NAIF1	0.196504172	4.67E-06
10047	DSE	-0.15724488	0.000260899
10048	HAUS6	-0.056626623	0.190953518
10049	ZBTB43	-0.102109572	0.018154354
10050	FRAS1	-0.177520883	3.64E-05
10051	TMEM255B	0.240249648	1.84E-08
10052	USP18	-0.012419546	0.774415494
10053	E2F6	0.060292693	0.163747052
10054	FAM102B	-0.2012489	2.70E-06
10055	FBXO30	-0.435115763	4.02E-26
10056	NEK4	-0.394072128	2.54E-21
10057	ZNF79	-0.029056279	0.502447771
10058	MTCP1	0.164578128	0.000131355
10059	NFATC1	0.106247333	0.013943223
10060	INPP5B	0.073377279	0.08997343
10061	ZNF510	-0.42723425	3.78E-25
10062	JMY	-0.370589561	7.35E-19
10063	MAPKAPK5	0.004428473	0.918604437
10064	DOCK2	-0.02688853	0.534868746
10065	GPRASP1	-0.192459672	7.35E-06
10066	DCAF17	-0.188665798	1.12E-05
10067	SMAD2	-0.486261602	4.22E-33
10068	ZNF507	-0.306162724	4.50E-13
10069	GTPBP8	-0.35220982	4.53E-17
10070	PRTFDC1	0.01099112	0.799774944
10071	TDP1	-0.142452697	0.000952822
10072	ZNF426	-0.421291757	1.98E-24
10073	SLC9A7	-0.276297469	7.88E-11
10074	SPN	0.132501895	0.002132281
10075	PLEKHG5	0.229528346	7.94E-08
10076	AGER	0.57959463	2.48E-49
10077	PTGR2	-0.19416673	6.08E-06
10078	WHSC1	0.190116193	9.53E-06
10079	VIL1	-0.060579446	0.161748642
10080	THAP6	-0.282554666	2.81E-11
10081	ZFPL1	0.003079547	0.943347061
10082	CD247	0.324801269	1.31E-14
10083	MLYCD	-0.081050381	0.061013313
10084	IRS1	-0.268514688	2.75E-10
10085	LTB4R	0.516530375	8.02E-38
10086	ANO8	0.408729413	5.84E-23
10087	GMEB1	0.007724633	0.858521898
10088	TTC25	0.209477164	1.02E-06
10089	DNASE1L3	-0.062152664	0.151110213
10090	EEPD1	0.138936035	0.001274229

10091	ZGPAT	0.533879179	9.49E-41
10092	SERPINA5	-0.098133391	0.023207324
10093	UBBP4	0.090764846	0.035832742
10094	SLC25A14	0.308816392	2.76E-13
10095	SIT1	0.339493036	6.74E-16
10096	LEPR	-0.45650155	6.74E-29
10097	STRBP	-0.300808174	1.19E-12
10098	ITGB3BP	0.094808074	0.028326904
10099	SPRYD4	0.178355586	3.34E-05
10100	HERC4	-0.018039813	0.677175862
10101	FAM179B	-0.417411065	5.71E-24
10102	SULT1A1	0.216357159	4.36E-07
10103	HCN3	0.382282692	4.63E-20
10104	IL20RB	0.21841921	3.36E-07
10105	FAR2	-0.172822036	5.86E-05
10106	NAV2	-0.041585871	0.337030297
10107	CD8B	0.276616279	7.48E-11
10108	SETD6	0.350092094	7.17E-17
10109	CHST10	0.074057977	0.087023903
10110	MYH14	-0.084261404	0.051429497
10111	PRIM1	-0.201810725	2.53E-06
10112	POMT2	0.131174564	0.002364793
10113	CCDC126	-0.387504395	1.30E-20
10114	ZNF506	-0.152946972	0.000384779
10115	DYRK4	0.485012016	6.46E-33
10116	FAM92A1	-0.103437131	0.016696007
10117	RAB37	0.274310907	1.09E-10
10118	ANKRD16	0.474360166	2.27E-31
10119	TFF3	0.204083507	1.94E-06
10120	RUFY2	-0.172807611	5.87E-05
10121	FAM131A	0.362424641	4.74E-18
10122	ZFP64	-0.276585408	7.52E-11
10123	DUS2	0.34035541	5.63E-16
10124	HTRA3	0.248581371	5.61E-09
10125	TMEM201	0.174438107	4.98E-05
10126	AASS	-0.148544194	0.00056697
10127	PRMT3	-0.110751091	0.010359909
10128	TUBGCP5	0.054812237	0.205585742
10129	IGHMBP2	0.468949278	1.32E-30
10130	ZNF69	0.096992142	0.024865932
10131	ABCA5	-0.155273255	0.000312192
10132	FAM135A	-0.317883858	5.01E-14
10133	SIAH1	-0.103969314	0.01614084
10134	EVPL	0.254511965	2.35E-09
10135	RALGPS2	-0.403004808	2.61E-22
10136	MGARP	0.014472738	0.738387179
10137	NLRP1	0.398978287	7.35E-22
10138	STK32C	0.501247719	2.22E-35
10139	BCKDHA	-0.313267266	1.20E-13
10140	IFT81	-0.002041475	0.962426302
10141	LMF1	0.26520534	4.61E-10
10142	ZNF880	-0.292628432	5.04E-12
10143	MOXD1	-0.074878844	0.083571068
10144	DHODH	0.077843471	0.072011725
10145	TMEM68	-0.23681321	2.96E-08
10146	MTAP	-0.291709894	5.91E-12
10147	ZBTB34	-0.182126276	2.25E-05
10148	IKBKE	0.370083467	8.26E-19

10149	TRNT1	-0.169241519	8.36E-05
10150	INPP4A	-0.199468012	3.32E-06
10151	UBE2S	0.603287913	2.43E-54
10152	ARNTL	0.00586794	0.892288079
10153	PAPOLG	-0.211271487	8.18E-07
10154	FBXL8	0.461647866	1.35E-29
10155	NFATC4	0.241280567	1.59E-08
10156	PPFIA4	0.292839367	4.86E-12
10157	ZNF521	-0.176067188	4.22E-05
10158	MYO19	0.346238897	1.63E-16
10159	C19orf47	0.46892233	1.33E-30
10160	MICAL3	-0.121907412	0.004747727
10161	MBNL3	-0.321433205	2.53E-14
10162	PHLDB3	0.23149266	6.11E-08
10163	NDE1	0.173038357	5.74E-05
10164	CDON	-0.086133446	0.046446323
10165	ARHGEF5	-0.029141473	0.501195116
10166	TSPAN5	-0.243488045	1.16E-08
10167	TNRC6B	-0.323669365	1.63E-14
10168	LPAR1	-0.124136347	0.004031369
10169	FAM126B	-0.214737695	5.33E-07
10170	ARHGAP9	0.453667884	1.61E-28
10171	PACRGL	-0.234785792	3.91E-08
10172	KCNN3	-0.352355553	4.39E-17
10173	GORAB	0.177834642	3.52E-05
10174	ZNF586	-0.092103735	0.033178904
10175	GPR180	-0.293975835	3.99E-12
10176	SETD9	-0.201028588	2.77E-06
10177	ZNF92	-0.320057557	3.30E-14
10178	PRSS16	-0.157970259	0.000244097
10179	USP15	-0.086833736	0.044689467
10180	CDAN1	0.345981302	1.73E-16
10181	DMKN	-0.014733189	0.733855744
10182	ALKBH8	-0.369311686	9.87E-19
10183	MUTYH	0.680438771	5.54E-74
10184	POU2F1	-0.210684225	8.79E-07
10185	TADA2A	0.356889593	1.63E-17
10186	SFN	0.297149601	2.28E-12
10187	ZCCHC11	-0.173343671	5.56E-05
10188	ZNF256	-0.172015699	6.35E-05
10189	GGACT	-0.083321166	0.054096053
10190	PRKCE	-0.397694107	1.02E-21
10191	ELMOD3	0.493754427	3.16E-34
10192	P2RY11	0.626234894	1.31E-59
10193	MREG	-0.004546354	0.9164456
10194	TBPL1	-0.215532478	4.83E-07
10195	SPAG1	0.004294553	0.921057754
10196	SFMBT1	-0.259821014	1.06E-09
10197	TTI2	-0.041112755	0.342560658
10198	RNASEH2B	0.160231243	0.000197994
10199	FCAMR	0.129156164	0.002762967
10200	ZNF451	-0.317203495	5.70E-14
10201	FAM118A	0.338035115	9.11E-16
10202	IKZF1	0.015461113	0.721240439
10203	POLR3B	-0.398751294	7.78E-22
10204	LDLR	-0.105920991	0.014240934
10205	PDCD2L	0.518191477	4.28E-38
10206	STEAP2	-0.28476568	1.94E-11

10207	KLHL15	-0.500798313	2.61E-35
10208	P3H3	0.245138256	9.21E-09
10209	TMEM237	-0.219764778	2.83E-07
10210	PRR5L	0.145321479	0.000748017
10211	AMMECR1	-0.28082062	3.75E-11
10212	NME6	-0.176991021	3.84E-05
10213	SPHK2	0.274516723	1.05E-10
10214	EPHA3	-0.158957807	0.000222846
10215	ZCCHC4	-0.286111171	1.54E-11
10216	ERV3-1	-0.030441435	0.482287371
10217	ERICH2	0.007295316	0.866308952
10218	CAPS	0.409324433	4.99E-23
10219	AREG	0.10172266	0.018599797
10220	ACY1	0.203236118	2.14E-06
10221	NXNL2	0.155089328	0.000317429
10222	ZNF226	0.078672955	0.069022334
10223	RAD52	0.370469296	7.56E-19
10224	C22orf29	-0.097441452	0.024201102
10225	MAPKBP1	0.13745386	0.001437466
10226	ZNF649	-0.396727532	1.30E-21
10227	SLC2A10	-0.07605854	0.0788035
10228	JPH2	0.025528617	0.555731449
10229	E2F1	0.397624628	1.04E-21
10230	SLC38A6	0.052568753	0.224780466
10231	FAT4	-0.417433699	5.68E-24
10232	IL18R1	0.057668964	0.182901042
10233	THSD1	-0.010672106	0.805468452
10234	CDC20	0.488005608	2.32E-33
10235	PYROXD2	0.265578554	4.35E-10
10236	L3MBTL3	-0.161966176	0.000168293
10237	KLHL18	-0.061084685	0.158272352
10238	KCTD7	0.011011846	0.799405406
10239	CAPRIN2	0.278628831	5.38E-11
10240	C7orf31	-0.177333038	3.71E-05
10241	WDR25	0.20803605	1.21E-06
10242	ZNF785	0.290161649	7.73E-12
10243	ITSN1	-0.166952939	0.000104515
10244	CD7	0.514055264	2.03E-37
10245	SLC26A1	0.131836335	0.002246121
10246	NAF1	-0.487431929	2.83E-33
10247	ZNF276	0.531151093	2.81E-40
10248	KLF8	0.002430694	0.955269559
10249	ZNF514	0.2040807	1.94E-06
10250	PRC1	0.275086473	9.60E-11
10251	MSTO1	0.624538778	3.33E-59
10252	MAMDC4	0.482590335	1.47E-32
10253	DOCK5	0.092732031	0.031992536
10254	ELOVL6	-0.052046246	0.229428589
10255	PAH	-0.080752603	0.061972353
10256	ZC3H12C	-0.43650222	2.69E-26
10257	DOC2B	-0.007634272	0.860159774
10258	KCNC3	-0.024681162	0.568929723
10259	LRRC29	0.353581066	3.36E-17
10260	MTRF1	0.148895736	0.000549902
10261	CSTA	0.272866669	1.37E-10
10262	GSTM2	0.26027783	9.85E-10
10263	TIMM8A	-0.158865753	0.000224751
10264	ZNF16	-0.001440988	0.973473469

10265	USP13	-0.099970946	0.020737901
10266	C5orf49	0.098652048	0.022485771
10267	ZNF197	-0.350415586	6.69E-17
10268	KIAA2018	-0.530150191	4.17E-40
10269	ZNF862	0.257049481	1.61E-09
10270	SFMBT2	-0.045567248	0.292774399
10271	ANO9	0.4652667	4.30E-30
10272	PUSL1	0.598202417	3.13E-53
10273	LIPT2	-0.279522563	4.64E-11
10274	FCGR1A	0.24756024	6.50E-09
10275	SLC22A23	-0.113179583	0.00878906
10276	INO80D	-0.107439275	0.01290189
10277	SMPDL3B	-0.025400829	0.557712042
10278	HHAT	-0.454502193	1.25E-28
10279	PYCR1	0.298498361	1.80E-12
10280	SCML1	0.147881893	0.00060047
10281	KIAA1407	0.266867833	3.56E-10
10282	ADH1B	-0.071625235	0.097935132
10283	SLX4IP	-0.069495884	0.108356021
10284	C16orf74	0.22976405	7.70E-08
10285	PPM1K	-0.167015451	0.000103884
10286	KIF3C	0.003226062	0.940656553
10287	DCUN1D2	0.302513531	8.74E-13
10288	CEP192	-0.147964338	0.000596202
10289	MMRN1	-0.128430979	0.002920326
10290	CCDC102B	-0.036842274	0.395069475
10291	CSDC2	0.034399671	0.427171745
10292	LRRC28	-0.327194628	8.16E-15
10293	SLC45A3	0.155293863	0.00031161
10294	MTHFSD	0.330584727	4.15E-15
10295	SETD4	0.359018654	1.02E-17
10296	KIFC2	0.591932939	6.88E-52
10297	AVPR1A	-0.325372132	1.17E-14
10298	CDH4	-0.053393602	0.217580006
10299	ZNF416	-0.343115612	3.16E-16
10300	SARDH	0.065249421	0.131733767
10301	CXCR3	0.339760209	6.37E-16
10302	MTRNR2L12	0.119457823	0.005665857
10303	DLG4	0.401030455	4.34E-22
10304	GDAP1	-0.134877404	0.001767651
10305	POLA2	0.340767201	5.17E-16
10306	PIGF	-0.307184428	3.73E-13
10307	FAM65B	-0.123769528	0.004142089
10308	FILIP1	-0.299599353	1.48E-12
10309	KIAA0895	-0.181086454	2.51E-05
10310	LYL1	0.423543489	1.06E-24
10311	LAMC2	-0.014528339	0.737419045
10312	RTN2	0.39991016	5.79E-22
10313	S1PR4	0.407731604	7.60E-23
10314	STK3	-0.40919677	5.16E-23
10315	DYNC2H1	-0.455746102	8.51E-29
10316	CASQ2	-0.19619812	4.83E-06
10317	EGR3	-0.1225919	0.004516383
10318	VTI1A	-0.325101028	1.23E-14
10319	EEF2KMT	0.171320897	6.81E-05
10320	ZNF552	-0.171810229	6.49E-05
10321	SLC25A15	-0.304676108	5.91E-13
10322	MT1A	0.182761989	2.11E-05

10323	CYB5R4	-0.365363381	2.44E-18
10324	NOD1	0.189702263	9.97E-06
10325	TTLL4	0.365822124	2.20E-18
10326	INO80B	0.651108229	7.72E-66
10327	ADHFE1	0.157367609	0.000257983
10328	ATR	-0.131372749	0.002328667
10329	METTL4	-0.217584095	3.74E-07
10330	PIAS2	-0.206030705	1.54E-06
10331	STAMBPL1	0.075761417	0.07998298
10332	PLA2G4A	-0.271461618	1.72E-10
10333	PRICKLE2	-0.098173716	0.023150515
10334	GPALPP1	-0.266642449	3.68E-10
10335	UTP15	-0.12809232	0.002996569
10336	SFXN2	-0.203602622	2.05E-06
10337	CYP2R1	0.241021462	1.65E-08
10338	GOLGA8A	0.336382015	1.28E-15
10339	ZMYM1	-0.215874893	4.63E-07
10340	C15orf65	0.202739945	2.27E-06
10341	PRKCZ	-0.161656447	0.000173269
10342	DIS3L2	0.394830056	2.10E-21
10343	PSTPIP2	0.111892515	0.009592961
10344	SHANK2	-0.3749218	2.67E-19
10345	PINX1	0.398308234	8.72E-22
10346	FGD3	0.274841695	9.98E-11
10347	TMED6	0.096749024	0.025232198
10348	PARD3B	-0.434300513	5.08E-26
10349	ALPK1	0.104258837	0.01584566
10350	LYRM4	0.219125704	3.07E-07
10351	ZNF628	0.312520301	1.38E-13
10352	LRFN3	-0.026502829	0.540745628
10353	KIAA1549	-0.30718253	3.73E-13
10354	KIAA1958	-0.419502317	3.23E-24
10355	MICB	0.273319256	1.28E-10
10356	ZNF181	-0.310482132	2.03E-13
10357	S100A3	0.295897648	2.85E-12
10358	MRPL53	0.567108232	7.45E-47
10359	TUBE1	0.087343905	0.043445065
10360	CXorf23	-0.155822078	0.000297041
10361	TTC8	-0.097767848	0.023727832
10362	TRAF5	0.351895431	4.85E-17
10363	PCBD2	-0.13354648	0.001964211
10364	TPD52L1	-0.150172676	0.000491838
10365	FER	-0.428033464	3.02E-25
10366	ADCK5	0.709345964	5.32E-83
10367	RAD54L2	-0.19402201	6.18E-06
10368	KIAA1919	-0.086562835	0.045362346
10369	ERICH1	0.066290134	0.125672113
10370	PRR5	0.412408853	2.20E-23
10371	TRIM11	0.538965072	1.22E-41
10372	ERI1	-0.049683154	0.251300891
10373	C4orf29	-0.100583806	0.01996673
10374	MTO1	-0.227392781	1.05E-07
10375	MOCS3	-0.290097834	7.81E-12
10376	USP51	-0.458345315	3.80E-29
10377	PDLIM2	0.383815466	3.20E-20
10378	CD5	0.274740883	1.01E-10
10379	ANKRD29	-0.169583868	8.08E-05
10380	ZNF595	-0.335840508	1.43E-15

10381	ERP27	-0.200369597	2.99E-06
10382	TMEM38A	-0.205561951	1.63E-06
10383	CDKL5	-0.425677775	5.85E-25
10384	AP4E1	-0.297680705	2.08E-12
10385	KDF1	-0.279339322	4.79E-11
10386	TNFRSF10C	-0.111654891	0.009748332
10387	EED	0.235116927	3.74E-08
10388	RFXAP	-0.129516466	0.002687692
10389	ATAT1	0.433342448	6.69E-26
10390	RNPC3	0.12420584	0.004010698
10391	PARP11	-0.383347125	3.58E-20
10392	ZNF587B	-0.038739401	0.371169509
10393	DCAF4	0.085029118	0.049334415
10394	RRM2	0.310922606	1.87E-13
10395	PDE1B	0.102563687	0.017643448
10396	NPAS2	0.275078999	9.61E-11
10397	DUSP4	-0.016739102	0.699275971
10398	IRX5	0.076583709	0.076753292
10399	TBC1D24	-0.021949868	0.612452737
10400	TLR7	-0.234832576	3.88E-08
10401	ARHGAP19	-0.252078658	3.37E-09
10402	SPINK13	0.095829779	0.026659472
10403	CCR2	0.016687531	0.700157603
10404	CCDC149	-0.280342568	4.06E-11
10405	TMEM82	0.042856166	0.322466466
10406	SERP2	0.057224919	0.186300221
10407	MRPL46	0.025310198	0.559118806
10408	TCTEX1D2	0.373448044	3.78E-19
10409	SUPT3H	-0.07758503	0.072964445
10410	GCNT3	-0.004452393	0.918166334
10411	SGK3	-0.066284515	0.125704248
10412	TMEM74B	0.410247741	3.91E-23
10413	ARHGAP10	-0.084825038	0.049884255
10414	BTK	0.087170178	0.043865501
10415	CROCC	0.521046486	1.44E-38
10416	ANGPT1	-0.371608725	5.80E-19
10417	DSCAML1	0.084838694	0.049847306
10418	SERTAD4	-0.280839594	3.74E-11
10419	FRMD6	-0.046155059	0.286586156
10420	NAA16	-0.029507494	0.495832099
10421	PTPN4	-0.454556274	1.23E-28
10422	MAP4K1	0.46939855	1.14E-30
10423	SLC2A11	0.068236612	0.114918987
10424	KIAA0513	-0.079730126	0.065360521
10425	CEP112	-0.086699051	0.045022948
10426	EGFLAM	0.073122203	0.091099157
10427	ZNF91	-0.33458683	1.85E-15
10428	C2orf40	-0.166710772	0.000106995
10429	ANKRD9	0.000881152	0.983777383
10430	SP4	-0.407972333	7.13E-23
10431	C10orf11	0.171304089	6.82E-05
10432	ALDH1A3	-0.029585708	0.494690065
10433	CCL15	0.124849628	0.003823713
10434	ZNF415	-0.319574066	3.62E-14
10435	PFKFB2	-0.179260504	3.04E-05
10436	ZNF35	-0.217495402	3.78E-07
10437	TRAPPC10	-0.282462725	2.85E-11
10438	EFNA3	0.328470462	6.33E-15

10439	ATP9B	-0.283343456	2.46E-11
10440	NGEF	0.189425708	1.03E-05
10441	ZNF268	-0.436407508	2.77E-26
10442	PIK3R5	0.141632925	0.001020221
10443	LCOR	-0.380965579	6.36E-20
10444	C2CD3	-0.120026535	0.005439508
10445	DUSP8	0.045416681	0.29437379
10446	ZNF680	-0.346232604	1.64E-16
10447	LCA5	-0.402436131	3.02E-22
10448	MCPH1	-0.379830553	8.35E-20
10449	ACYP2	-0.021810379	0.614714384
10450	UBN2	-0.091588326	0.034179954
10451	CCDC176	-0.160766334	0.000188347
10452	ZC4H2	-0.204939985	1.75E-06
10453	WRN	-0.331285857	3.60E-15
10454	CLIC6	-0.202078142	2.45E-06
10455	FCN1	0.129229136	0.002747568
10456	MT1H	-0.018145833	0.675386337
10457	GPSM1	0.344796142	2.22E-16
10458	ADRBK2	-0.159397269	0.000213957
10459	LRRC8B	-0.338410024	8.43E-16
10460	PCDH18	-0.184007167	1.85E-05
10461	GABBR1	0.381172598	6.05E-20
10462	GRIK3	-0.195213261	5.40E-06
10463	SYNJ2	-0.088123958	0.041598873
10464	CD300LF	0.185846554	1.52E-05
10465	TRIM62	0.133178941	0.002021912
10466	ADAM28	-0.048957614	0.25829836
10467	CDK1	0.310886086	1.88E-13
10468	HIST3H2A	0.133990421	0.001896527
10469	USP54	-0.207817969	1.24E-06
10470	CACHD1	-0.188249273	1.17E-05
10471	TLR5	-0.240051258	1.89E-08
10472	CAMKK1	0.199516596	3.31E-06
10473	KLHL28	-0.401022775	4.35E-22
10474	KNSTRN	0.229401745	8.08E-08
10475	RPP14	-0.354526849	2.74E-17
10476	GLMN	0.149442238	0.000524313
10477	NCAPG2	-0.059019817	0.172842244
10478	YOD1	-0.154507319	0.000334546
10479	GTPBP10	-0.375516389	2.32E-19
10480	PAQR4	0.397362032	1.11E-21
10481	TMEM233	-0.143221709	0.000893358
10482	FBXW7	0.024888159	0.565692204
10483	VAMP1	0.450555025	4.17E-28
10484	ALMS1	-0.386476402	1.67E-20
10485	PIDD1	0.61689136	2.07E-57
10486	PCDHB15	-0.016883951	0.696801842
10487	ZNF529	0.109122559	0.011548292
10488	GZMM	0.435883293	3.22E-26
10489	TRPS1	-0.216521455	4.27E-07
10490	OSCAR	0.408507432	6.19E-23
10491	HSPA6	0.349723675	7.76E-17
10492	ABCC10	0.505896671	4.14E-36
10493	SLC25A19	0.527040368	1.42E-39
10494	ZNF445	-0.232575794	5.28E-08
10495	CCDC120	0.233829137	4.45E-08
10496	TEFM	0.069882041	0.106403856

10497	SPINK1	-0.017698357	0.682951639
10498	TANC2	-0.252518345	3.15E-09
10499	NT5M	0.225418785	1.37E-07
10500	ADAM19	0.228798173	8.75E-08
10501	NANP	-0.30193722	9.70E-13
10502	CCP110	-0.303814886	6.91E-13
10503	KHDRBS3	-0.195582352	5.18E-06
10504	LCMT2	-0.2860208	1.57E-11
10505	HPGD	-0.232414654	5.39E-08
10506	KCTD21	0.112085575	0.009468353
10507	LYNX1	-0.149080749	0.000541112
10508	MT1M	0.044150553	0.308054026
10509	ZNF562	-0.161960985	0.000168375
10510	DPY19L2	0.107268828	0.013046465
10511	ACSF3	0.276170183	8.05E-11
10512	DACT1	-0.083298782	0.054160912
10513	NFKBID	0.453816077	1.54E-28
10514	NAV1	0.027152924	0.530858834
10515	CEP164	0.410762737	3.41E-23
10516	DOT1L	0.409209033	5.15E-23
10517	KCNJ5	-0.132626794	0.002111515
10518	SHROOM3	-0.370785981	7.02E-19
10519	GABPB2	0.128278506	0.002954431
10520	STEAP1	-0.006308024	0.884264638
10521	MAP3K12	0.421633016	1.80E-24
10522	CDC14A	-0.371886425	5.44E-19
10523	PTGER2	-0.013563025	0.754284992
10524	KDM2B	0.094505998	0.028836745
10525	NKAIN4	0.131792912	0.002253738
10526	TTL	0.034579182	0.424762108
10527	CSPP1	0.034845186	0.421206051
10528	SCYL3	-0.098331864	0.022928875
10529	PARVG	0.451323767	3.30E-28
10530	USP46	-0.449353458	6.00E-28
10531	FCGBP	-0.013418084	0.756827741
10532	MBLAC2	-0.487694187	2.58E-33
10533	HGF	-0.084765681	0.050045138
10534	SYBU	0.030306954	0.484225265
10535	COX19	0.405288677	1.44E-22
10536	CPNE5	0.165675992	0.000118229
10537	SATB1	-0.421696858	1.77E-24
10538	CEP44	-0.108386632	0.012123805
10539	LPIN1	-0.117855931	0.006349652
10540	EIF4E	-0.430088133	1.69E-25
10541	MYO7A	0.114843538	0.007838967
10542	CDC42BPG	-0.152347143	0.000405896
10543	ZNF836	-0.300875365	1.17E-12
10544	WRAP53	0.587579796	5.65E-51
10545	CDH24	0.281265826	3.48E-11
10546	ZC3H14	-0.294914329	3.38E-12
10547	C17orf107	-0.036874264	0.39465893
10548	COBL	-0.256467205	1.75E-09
10549	RNF215	0.619046834	6.54E-58
10550	GEMIN2	0.099204135	0.021739157
10551	CYP4X1	-0.083723668	0.052940777
10552	CNTNAP1	0.352910992	3.89E-17
10553	KIAA1244	-0.368925757	1.08E-18
10554	PODN	0.047350022	0.274279305

10555	C1orf21	-0.010606334	0.806643588
10556	FUT4	-0.028975185	0.503641676
10557	HOXB9	0.031204423	0.471372543
10558	LPAR5	0.052244081	0.227660733
10559	TCHP	0.270680447	1.95E-10
10560	HOXB5	0.185609194	1.56E-05
10561	HSD11B1L	0.385004839	2.40E-20
10562	ZNF517	0.417654693	5.34E-24
10563	CAPN10	0.585221007	1.74E-50
10564	VPS53	0.01616356	0.709137924
10565	MTRNR2L1	0.046718747	0.280735172
10566	CEP162	-0.034267629	0.42894925
10567	TFCP2L1	-0.228770443	8.79E-08
10568	C2orf72	-0.021473152	0.620197195
10569	ANKRD22	0.107429197	0.012910398
10570	IL17RD	-0.365686646	2.27E-18
10571	WASF3	-0.227097984	1.10E-07
10572	TIGD1	0.490552073	9.64E-34
10573	REC8	0.493517264	3.44E-34
10574	LEF1	0.034154133	0.430480517
10575	TTC5	-0.279586408	4.60E-11
10576	PTPRD	-0.09995082	0.020763659
10577	SLC30A4	-0.446518123	1.41E-27
10578	C15orf57	-0.150147588	0.000492921
10579	ZNF845	-0.522032675	9.87E-39
10580	IL22RA1	0.189701748	9.97E-06
10581	PPP1R9A	-0.343615142	2.85E-16
10582	THOC3	0.217949622	3.57E-07
10583	HINFP	0.365567096	2.33E-18
10584	C4orf46	-0.142732644	0.000930764
10585	BBIP1	-0.169797421	7.92E-05
10586	SWSAP1	0.456060835	7.72E-29
10587	ARAP2	-0.186412193	1.43E-05
10588	P2RY1	-0.267800937	3.07E-10
10589	ZNF500	0.218045743	3.52E-07
10590	TET2	-0.347041178	1.38E-16
10591	ST3GAL6	-0.076907887	0.075509526
10592	L2HGDH	-0.431568535	1.11E-25
10593	SLC43A1	0.065416245	0.130747171
10594	TET3	0.087977583	0.041940168
10595	HAUS3	-0.112149241	0.009427578
10596	TIGD6	-0.204601021	1.82E-06
10597	TRNP1	0.044044122	0.309222801
10598	PRODH	0.110932138	0.01023474
10599	CHRD	0.309071005	2.63E-13
10600	SCGB1D2	0.125442059	0.00365866
10601	LAG3	0.413239178	1.76E-23
10602	HDGFRP3	-0.264748623	4.95E-10
10603	KCNAB1	-0.244544799	1.00E-08
10604	TRIM3	0.510854013	6.70E-37
10605	RBM41	-0.101255052	0.019150847
10606	SPDL1	0.15303503	0.000381767
10607	FAM162B	-0.063421616	0.142923585
10608	KLHL13	-0.367332339	1.56E-18
10609	ING5	0.384693993	2.58E-20
10610	TAF4	0.166001003	0.000114586
10611	ZNF790	-0.352396028	4.35E-17
10612	SPDYE3	0.056174605	0.19452528

10613	FOXF1	-0.007048934	0.8707838
10614	CENPU	0.199741973	3.22E-06
10615	WRAP73	0.651070693	7.90E-66
10616	BAHCC1	0.155238456	0.000313177
10617	OPN1SW	0.104524916	0.015578569
10618	LMLN	-0.118335958	0.006137357
10619	CCNJL	-0.024960984	0.564555296
10620	CCRL2	-0.104620558	0.015483533
10621	ZNF366	-0.384089541	2.99E-20
10622	FAM161B	-0.221578856	2.25E-07
10623	SPAG16	-0.175016471	4.70E-05
10624	STRA8	0.173622317	5.41E-05
10625	MVB12B	-0.148620162	0.00056324
10626	BRWD3	-0.351091467	5.78E-17
10627	SPA17	-0.049505273	0.253004134
10628	ZNF254	-0.360884215	6.70E-18
10629	ODF2L	0.115505811	0.007487099
10630	IGFBP1	0.136465466	0.001556779
10631	SEMA4F	0.04002479	0.355496208
10632	ZBTB16	-0.20070809	2.88E-06
10633	IFITM10	0.246225224	7.88E-09
10634	ZNF736	-0.116464396	0.007002804
10635	FGA	0.060948862	0.159201288
10636	NXPE3	-0.09309243	0.031328527
10637	ACER2	-0.468078828	1.75E-30
10638	KCP	0.281140521	3.55E-11
10639	AQP7	0.068587997	0.113057066
10640	SNAP25	0.11708044	0.006706567
10641	TMEM198	0.229558662	7.91E-08
10642	TBX3	-0.143223342	0.000893236
10643	PRIM2	-0.121629498	0.004844671
10644	SLC24A1	-0.402999796	2.61E-22
10645	TLL1	-0.213023196	6.60E-07
10646	DEPDC5	-0.008155637	0.850718043
10647	GDPD3	0.52457579	3.70E-39
10648	FIGNL1	-0.168097747	9.35E-05
10649	TMEM231	-0.207308489	1.32E-06
10650	ATL1	-0.277197448	6.81E-11
10651	CH507-42P11.8	0.052288874	0.227261802
10652	GPR65	0.051087398	0.23813487
10653	ENTHD2	0.657890805	1.22E-67
10654	SRPX2	0.180446286	2.69E-05
10655	ACAP1	0.578411389	4.30E-49
10656	AMICA1	0.240217119	1.85E-08
10657	ZNF367	-0.098349325	0.022904518
10658	STOML1	0.343529074	2.90E-16
10659	ARL10	-0.174892858	4.76E-05
10660	C8orf37	-0.217352206	3.85E-07
10661	TAL2	-0.132872577	0.00207119
10662	RAMP1	0.114609247	0.00796694
10663	NUP62CL	-0.152220459	0.000410492
10664	HOXB4	0.167697814	9.72E-05
10665	CACNB3	0.343867013	2.70E-16
10666	CCDC88C	0.280623502	3.87E-11
10667	CASP8AP2	-0.21277068	6.80E-07
10668	GABRE	0.240169686	1.86E-08
10669	MSX1	0.30261433	8.59E-13
10670	ABCB6	0.424884686	7.30E-25

10671	ZNF440	-0.203682654	2.03E-06
10672	JRK	-0.032471522	0.453550229
10673	ANKRD44	-0.119209956	0.005767133
10674	CCNA2	0.377663957	1.40E-19
10675	ZNF653	0.446719218	1.33E-27
10676	NEU3	-0.113732541	0.008462522
10677	MYO5C	-0.277010603	7.02E-11
10678	SRR	-0.044552628	0.303664969
10679	AK1	0.350113944	7.13E-17
10680	APOL4	0.09300982	0.03147968
10681	NRTN	0.253625076	2.68E-09
10682	AC087350.1	0.020211467	0.640895724
10683	ANKEF1	-0.406590365	1.03E-22
10684	FGG	-0.005159345	0.905229669
10685	DDX11	0.495709018	1.59E-34
10686	DFNB31	0.362795164	4.36E-18
10687	TMEM81	0.498102311	6.83E-35
10688	PLD6	0.078265856	0.070476532
10689	MYO7B	0.014442895	0.738906979
10690	HLA-DOB	0.226128272	1.25E-07
10691	OR51E1	-0.086038307	0.046689417
10692	CLIC5	-0.226889948	1.13E-07
10693	CLIP4	-0.117512946	0.006505352
10694	NBEA	-0.472774779	3.81E-31
10695	SORCS2	-0.088528271	0.040668319
10696	ZNF75A	0.186000022	1.49E-05
10697	ZNF587	0.101499765	0.018860717
10698	VWCE	0.190196714	9.45E-06
10699	TMEM161B	-0.370528035	7.45E-19
10700	CSF3R	0.322618729	2.01E-14
10701	SPATA7	-0.245658576	8.55E-09
10702	TMEM56	-0.396879832	1.25E-21
10703	RAD51C	-0.038514735	0.373952218
10704	GIN1	-0.351991688	4.75E-17
10705	CLEC2D	0.370349436	7.77E-19
10706	COQ6	0.246704203	7.36E-09
10707	RMI2	0.122522343	0.004539419
10708	C12orf66	-0.263778889	5.75E-10
10709	NFASC	-0.05104445	0.238530205
10710	TRPM2	0.284362738	2.07E-11
10711	XAF1	0.235135347	3.73E-08
10712	MAPK10	-0.235041597	3.78E-08
10713	ZNF175	-0.301837088	9.88E-13
10714	SIGLEC8	-0.057708344	0.182601813
10715	YAF2	-0.261907861	7.68E-10
10716	CENPH	0.279478004	4.68E-11
10717	CCDC89	-0.079222732	0.067097584
10718	ZNF180	-0.349383819	8.35E-17
10719	AURKA	0.240426141	1.79E-08
10720	PDCD1LG2	-0.058958075	0.173292808
10721	TMEM116	-0.152328613	0.000406566
10722	MCTP2	-0.076927784	0.075433724
10723	CXorf57	-0.09184186	0.033684357
10724	PLA2G6	0.50166047	1.92E-35
10725	LRFN4	0.413521454	1.63E-23
10726	GPRIN2	0.123432079	0.004246365
10727	IL16	0.155635626	0.000302109
10728	CXCL13	0.314335365	9.84E-14

10729	LBP	0.062074956	0.151622888
10730	MBD5	-0.369423464	9.62E-19
10731	RBM45	0.223432188	1.77E-07
10732	LRRK1	0.087035953	0.044192679
10733	NEK8	0.400189124	5.39E-22
10734	CCL8	0.098247048	0.023047512
10735	TTLL5	-0.124013385	0.004068182
10736	SAMD11	0.141307251	0.001048197
10737	DGKH	-0.192691765	7.17E-06
10738	TMEM79	0.526282735	1.90E-39
10739	C4orf33	-0.211944872	7.53E-07
10740	TRIM16L	0.001925927	0.964551551
10741	NCAPD3	-0.101429087	0.018944116
10742	FNDC4	0.046867458	0.279205166
10743	ERI2	-0.236761956	2.98E-08
10744	ZXDA	-0.473821196	2.71E-31
10745	ANKRD39	0.476392513	1.16E-31
10746	MINA	-0.253220662	2.84E-09
10747	NUBPL	-0.513138384	2.87E-37
10748	ARHGAP15	0.130785964	0.002437119
10749	C9orf40	0.025823821	0.551169195
10750	PRKAR2B	-0.143925815	0.000841941
10751	SATB2	-0.097529312	0.02407291
10752	ATXN7L1	-0.103891436	0.016221057
10753	EGF	-0.295667348	2.96E-12
10754	RNF217	-0.173361238	5.55E-05
10755	SLC36A4	-0.346586611	1.52E-16
10756	TNFRSF25	0.48962144	1.33E-33
10757	GLUD2	-0.397067841	1.19E-21
10758	C11orf30	-0.318708738	4.28E-14
10759	LRIG2	-0.022906865	0.597036186
10760	ATAD2	0.085295248	0.048625023
10761	NDUFA7	0.426690687	4.41E-25
10762	NUDT17	0.2425235	1.34E-08
10763	PLEKHA3	-0.374689122	2.82E-19
10764	SLC22A3	0.044163456	0.307912529
10765	METAP1D	0.239210026	2.12E-08
10766	SLC25A34	0.198378812	3.77E-06
10767	FGFR1OP	0.076620562	0.076611065
10768	IKZF4	-0.058267218	0.178394162
10769	CHST12	0.437780404	1.86E-26
10770	PCYOX1L	0.241812911	1.48E-08
10771	GPATCH2	-0.065237504	0.131804463
10772	ZNF10	0.005971947	0.890390834
10773	FARP2	-0.118585595	0.006029492
10774	PMM2	0.020244409	0.640351646
10775	SPATC1L	0.314602618	9.35E-14
10776	DNAJC27	-0.348785408	9.49E-17
10777	PGBD2	0.030261526	0.484880846
10778	GNGT2	0.194345594	5.96E-06
10779	CTXN1	0.220743036	2.50E-07
10780	EIF1AY	-0.04012194	0.354328785
10781	LMNTD2	0.534175373	8.43E-41
10782	TIPIN	0.091317107	0.034716986
10783	CLEC10A	0.103183835	0.016966066
10784	PIEZO2	-0.220387376	2.62E-07
10785	NUMBL	0.593338899	3.46E-52
10786	EXO5	0.047637602	0.271372104

10787	ZBTB12	0.249117148	5.19E-09
10788	WDR7	-0.506435675	3.40E-36
10789	PHF20L1	-0.210918345	8.54E-07
10790	TCEAL7	-0.126411614	0.00340243
10791	NAT1	-0.106752437	0.013493234
10792	MDM1	0.049857678	0.249637551
10793	C16orf86	0.128269848	0.002956379
10794	FAM228B	0.167844374	9.58E-05
10795	USHBP1	0.203572463	2.06E-06
10796	MDP1	0.153653289	0.000361227
10797	ADAM8	0.461396342	1.46E-29
10798	FLVCR1	0.233512619	4.65E-08
10799	ZNF433	-0.221088272	2.39E-07
10800	MEIS2	-0.166014064	0.000114442
10801	MMP1	0.018512216	0.669216268
10802	RBM28	0.197722715	4.06E-06
10803	SH2D2A	0.514242843	1.90E-37
10804	CCDC125	0.081563859	0.059388394
10805	FRS3	0.551672998	6.21E-44
10806	CHN1	0.055754116	0.197891577
10807	STAC3	0.497151687	9.57E-35
10808	KIN	0.164123085	0.000137187
10809	SH3BP1	0.41932113	3.39E-24
10810	ZNF550	-0.174537411	4.93E-05
10811	CCDC28B	0.33346586	2.32E-15
10812	ZMYND12	-0.207671625	1.27E-06
10813	PABPC4L	-0.43106623	1.28E-25
10814	ZNF780A	-0.405659509	1.31E-22
10815	IL11RA	0.380284849	7.49E-20
10816	CRAMP1L	0.235477568	3.56E-08
10817	PLEKHG4	0.32863019	6.13E-15
10818	GPR143	-0.069429472	0.108694588
10819	IL1RL2	-0.003107723	0.942829612
10820	PRX	-0.104613684	0.015490347
10821	KDM4C	0.035527762	0.412161172
10822	PGBD5	-0.068814281	0.111870632
10823	TTYH2	-0.088337435	0.041105326
10824	DDX60L	0.036913338	0.394157828
10825	CCDC77	0.157767642	0.000248686
10826	GPATCH2L	-0.298249665	1.88E-12
10827	DAAM1	-0.299281088	1.56E-12
10828	CLCNKB	-0.252168452	3.32E-09
10829	UBE2V1	-0.195133917	5.45E-06
10830	ZNF449	0.054028776	0.212149121
10831	MAP9	-0.2958823	2.85E-12
10832	OSBPL7	0.518584476	3.69E-38
10833	RDH5	0.264500129	5.15E-10
10834	SLAIN1	-0.198329682	3.79E-06
10835	LRR1	0.090336912	0.036718155
10836	NOMO2	-0.35263322	4.14E-17
10837	BCAT1	-0.109952419	0.010928439
10838	TM6SF1	-0.284431558	2.05E-11
10839	ANXA3	-0.029166815	0.500822802
10840	VWA7	-0.118858176	0.005913661
10841	C3orf70	-0.288186057	1.08E-11
10842	SLC46A1	-0.237896104	2.55E-08
10843	RNF157	0.086605527	0.045255745
10844	KCNK6	0.336998029	1.13E-15

10845	FAM120C	-0.34471962	2.26E-16
10846	C12orf76	0.262246875	7.29E-10
10847	FAM83D	0.165766696	0.000117201
10848	CALML4	0.133310743	0.002001044
10849	GDF11	0.04437934	0.305551477
10850	CCDC121	-0.480438523	3.02E-32
10851	C2orf15	-0.01333488	0.758288607
10852	ZNF600	0.283353106	2.46E-11
10853	SHROOM2	-0.2821325	3.01E-11
10854	ZNF786	0.243526603	1.16E-08
10855	TXNDC5	-0.189691286	9.99E-06
10856	ARHGAP33	0.550439003	1.05E-43
10857	C8orf76	0.414178681	1.37E-23
10858	RERGL	-0.048558319	0.262206305
10859	KNOP1	0.060841334	0.159939615
10860	SCAPER	-0.50613759	3.79E-36
10861	TMEM170B	-0.512982848	3.04E-37
10862	SIX5	0.385435065	2.16E-20
10863	PDE10A	-0.152585942	0.000397363
10864	SNED1	-0.143041593	0.00090697
10865	CTH	-0.224084659	1.63E-07
10866	FBXO2	0.046751014	0.280402712
10867	HEXIM2	0.199937122	3.15E-06
10868	ATG10	-0.089892895	0.037656418
10869	G6PC	-0.149048338	0.000542642
10870	FHIT	0.072069651	0.095864324
10871	HAPLN1	-0.090672425	0.036022412
10872	MMACHC	-0.038769109	0.370802507
10873	RNASE4	-0.072122145	0.095622046
10874	TOX	0.07099071	0.100953362
10875	ELAC1	-0.117077989	0.006707723
10876	TBC1D10C	0.528213062	8.95E-40
10877	EMID1	0.149998466	0.000499409
10878	TNRC6C	-0.07562554	0.080527116
10879	CCDC88B	0.588742107	3.23E-51
10880	HPSE	-0.257705751	1.45E-09
10881	DERL3	0.318650299	4.32E-14
10882	PUS7L	-0.41404019	1.42E-23
10883	THRB	-0.462148294	1.15E-29
10884	TSTD2	-0.304108049	6.55E-13
10885	DCBLD1	0.136763795	0.001519842
10886	ZNF783	0.429780801	1.85E-25
10887	CCNJ	-0.081800806	0.058650731
10888	NKD2	0.255058768	2.16E-09
10889	MTERF1	-0.149130674	0.000538762
10890	BOC	-0.081819055	0.058594234
10891	ANGPTL3	-0.158323848	0.000236279
10892	CXorf21	-0.052745484	0.223223595
10893	PPAPDC3	0.014027768	0.746149851
10894	CD72	0.407779035	7.50E-23
10895	HENMT1	0.125642532	0.003604288
10896	C5orf42	-0.303958813	6.73E-13
10897	NSUN3	-0.374630989	2.86E-19
10898	LRRC48	0.158004888	0.000243321
10899	PCDHB10	-0.121450879	0.004907917
10900	OLFM2	0.241343051	1.58E-08
10901	SYTL4	0.002439718	0.955103667
10902	SIGLEC9	0.146549604	0.000673493

10903	GPR153	0.265418131	4.46E-10
10904	SMAD6	-0.01677659	0.698635335
10905	ZNF234	-0.104089892	0.016017326
10906	LYST	-0.064396498	0.136868094
10907	TCAF2	-0.039645286	0.360079743
10908	ZSCAN2	-0.095276734	0.027551238
10909	CPXM1	0.185694411	1.54E-05
10910	RGS18	-0.085764166	0.047395867
10911	PDZD3	0.360045348	8.08E-18
10912	C19orf38	0.396435353	1.40E-21
10913	COL16A1	0.19779929	4.03E-06
10914	PGBD1	0.075060191	0.082823382
10915	PDF	0.099078045	0.021907751
10916	EFS	0.02442334	0.572974481
10917	HIATL2	0.034881345	0.420724021
10918	ZBED6	-0.369094384	1.04E-18
10919	FAM175A	-0.482629167	1.45E-32
10920	MAP1A	-0.075206212	0.082225282
10921	GOLGA8B	0.422519241	1.41E-24
10922	COX20	0.003765557	0.930755635
10923	ELFN1	0.093044748	0.031415695
10924	CKMT2	-0.153878222	0.000354014
10925	NDNF	-0.079459158	0.066283522
10926	GFPT2	0.210733342	8.73E-07
10927	BMP2K	-0.337083231	1.11E-15
10928	ASPDH	0.174089161	5.16E-05
10929	SLC8A1	-0.321991575	2.27E-14
10930	DSEL	-0.143949634	0.00084025
10931	SDCBP2	-0.078214924	0.070660217
10932	MSLN	0.063956461	0.139576488
10933	NOVA2	0.001182403	0.978232339
10934	LRTOMT	0.032232183	0.45688731
10935	KIF26A	-0.078827806	0.068475684
10936	IL1B	0.052944851	0.221476565
10937	FBXO36	-0.106787681	0.013462318
10938	FGF1	-0.183724754	1.90E-05
10939	ZNF775	0.390397786	6.37E-21
10940	NAGS	-0.0724779	0.093992982
10941	HS3ST1	0.006802412	0.875265298
10942	ISG20	0.569191766	2.92E-47
10943	ARSG	-0.160305033	0.000196636
10944	RBL1	-0.125941995	0.003524432
10945	PTPN7	0.403087496	2.55E-22
10946	NTRK2	-0.159722972	0.000207584
10947	ATP6V1B1	-0.152125418	0.000413972
10948	POP1	0.169437493	8.20E-05
10949	ST20	0.497106588	9.72E-35
10950	C4orf48	0.472914245	3.64E-31
10951	PLCE1	-0.239798045	1.96E-08
10952	CD300C	0.043249461	0.318041628
10953	PTGIR	0.253750946	2.63E-09
10954	LINS	0.026632442	0.538767138
10955	SCTR	0.088387315	0.040990721
10956	SPECC1	-0.047738987	0.270352223
10957	FAM133B	-0.004827223	0.911304328
10958	CD79A	0.237885967	2.55E-08
10959	PCSK5	-0.088402913	0.040954939
10960	ADAM22	-0.334867572	1.74E-15

10961	SIGLEC14		0.060206228	0.164353259
10962	TRERF1		0.069428983	0.108697086
10963	AQP2		-0.092650648	0.032144132
10964	GANC		-0.125843678	0.003550471
10965	KCTD1		-0.305124431	5.44E-13
10966	ZNF7		0.368715862	1.13E-18
10967	SSC5D		0.152890297	0.00038673
10968	TMEM194B		-0.248734755	5.49E-09
10969	ZNF589		0.110940424	0.010229044
10970	SLC25A27		0.170626222	7.29E-05
10971	ZFP14		-0.254433006	2.38E-09
10972	BNC2		-0.041557055	0.337365485
10973	C1QL4		0.129411155	0.002709498
10974	PAXIP1		-0.063976887	0.139449868
10975	AACS		-0.03713337	0.39134317
10976	METTL21A		0.107205562	0.01310049
10977	ZCWPW1		0.280453463	3.98E-11
10978	ASTN2		-0.312528055	1.38E-13
10979	DIABLO		-0.126966289	0.003263278
10980	GSTCD		-0.447338858	1.10E-27
10981	SLC29A2		-0.072922727	0.091987346
10982	CYSLTR2		-0.081769249	0.058748535
10983	ADRA2B		0.023763559	0.583386696
10984	ZNF101		0.135794445	0.001642891
10985	SLC14A1		-0.199942737	3.15E-06
10986	ADAL		-0.281765341	3.20E-11
10987	P2RY14		-0.12894667	0.002807613
10988	PDZRN3		-0.168274934	9.19E-05
10989	CHORDC1		0.132862866	0.00207277
10990	ZNF224		0.212623392	6.93E-07
10991	CLCN2		0.401440711	3.91E-22
10992	DOK5		-0.042868268	0.322329717
10993	HIST1H3H		0.175142697	4.64E-05
10994	HNRNPA1L2		0.311105052	1.80E-13
10995	EFHC2		-0.241043726	1.64E-08
10996	LRCH4		0.337033942	1.12E-15
10997	ZNF446		0.519512827	2.59E-38
10998	RAB23		-0.285440274	1.73E-11
10999	ARHGEF19		0.40634322	1.09E-22
11000	CCDC57		0.539001658	1.20E-41
11001	RSPH14		0.126673565	0.003336057
11002	STK26		-0.049513811	0.252922197
11003	ZNF607		-0.201901163	2.51E-06
11004	SLC16A7		-0.336083145	1.36E-15
11005	RASGRP2		0.344397936	2.41E-16
11006	CD96		0.198976751	3.52E-06
11007	PPP1R3G		-0.077548063	0.073101559
11008	PPAN		0.61437794	7.84E-57
11009	SPICE1		-0.082434496	0.056715134
11010	ZNF613		-0.291936466	5.69E-12
11011	ZNF718		-0.391323628	5.06E-21
11012	GUCY1A2		-0.312179612	1.48E-13
11013	FAM185A		-0.181482187	2.41E-05
11014	HMCN1		-0.304232842	6.40E-13
11015	GULP1		-0.363585595	3.65E-18
11016	TCP11L2		-0.513775436	2.26E-37
11017	SLC6A18		-0.017370284	0.688518532
11018		1-Mar	-0.193207431	6.77E-06

11019	BAZ2B	-0.210349907	9.15E-07
11020	DZIP1L	0.323430975	1.71E-14
11021	TUBGCP4	-0.255094532	2.15E-09
11022	ASPSCR1	0.715283688	5.43E-85
11023	ZNF250	-0.096894111	0.025013062
11024	ASPG	-0.047124905	0.276569813
11025	PHLPP1	-0.32830228	6.55E-15
11026	MYO1G	0.366248866	1.99E-18
11027	TMEM92	0.093868767	0.029938144
11028	PHOSPHO2	-0.381164379	6.06E-20
11029	AQP9	0.068741083	0.112253339
11030	HEATR5A	-0.404863569	1.61E-22
11031	MRPL38	0.603648915	2.02E-54
11032	ZNF669	-0.365504965	2.36E-18
11033	CENPBD1	-0.374907854	2.68E-19
11034	MAGI3	-0.40450897	1.77E-22
11035	SFI1	0.515271696	1.29E-37
11036	SDK1	-0.02917983	0.500631668
11037	DNAH11	-0.041696073	0.335750386
11038	IRAK3	-0.17000508	7.76E-05
11039	MON1A	0.293810597	4.10E-12
11040	MAN1C1	-0.164716485	0.000129628
11041	SACS	0.034286593	0.4286937
11042	S1PR2	0.287200304	1.28E-11
11043	TMEM144	-0.353503037	3.42E-17
11044	MANEAL	0.186300791	1.44E-05
11045	SUV39H2	-0.127686685	0.003090273
11046	SSTR2	0.017934924	0.678948069
11047	PDP2	-0.355427078	2.25E-17
11048	GAS7	0.017762386	0.68186716
11049	LRP12	-0.33798744	9.20E-16
11050	URB2	-0.072985891	0.091705354
11051	AHI1	0.029835867	0.491046833
11052	ZWILCH	0.03099022	0.47442301
11053	EPO	-0.009548611	0.82559979
11054	C18orf32	-0.336861487	1.16E-15
11055	KDM5D	0.066748248	0.123073503
11056	MCHR1	0.032585948	0.451959646
11057	ITGA11	-0.005745293	0.894526109
11058	ZFYVE28	0.131071831	0.002383721
11059	ST6GALNAC3	-0.310499368	2.02E-13
11060	LOXL3	0.215006476	5.16E-07
11061	FREM2	-0.273674765	1.21E-10
11062	TMEM30B	-0.271112516	1.82E-10
11063	TTBK2	-0.277711243	6.26E-11
11064	CABLES2	0.297407589	2.18E-12
11065	CCL3L3	0.076656514	0.076472522
11066	RASGEF1B	-0.181944515	2.30E-05
11067	PF4V1	0.013443995	0.756372983
11068	PRAME	0.216321463	4.38E-07
11069	CLEC4E	0.231765368	5.89E-08
11070	DTNB	0.097928118	0.023498388
11071	C3orf62	0.447952004	9.16E-28
11072	PDE8B	-0.333184505	2.46E-15
11073	GXYLT2	-0.160511952	0.000192876
11074	ZNRF1	0.308950367	2.69E-13
11075	CNTRL	0.153862394	0.000354517
11076	CRYBG3	-0.527710505	1.09E-39

11077	UNC13D	0.563698404	3.39E-46
11078	C16orf54	0.216130834	4.48E-07
11079	TRHDE	-0.398415876	8.48E-22
11080	HSPBAP1	0.340696348	5.25E-16
11081	DMRTA1	-0.268316287	2.83E-10
11082	ITGB7	0.053306692	0.218330793
11083	METTL25	-0.250936774	3.98E-09
11084	FOXM1	0.366172618	2.03E-18
11085	NAP1L2	-0.239312673	2.09E-08
11086	HOXD4	0.020196062	0.641150227
11087	SFXN5	0.250318299	4.36E-09
11088	SPRTN	-0.219973143	2.76E-07
11089	AMDHD1	-0.040896384	0.345109041
11090	SLC2A6	0.555480047	1.22E-44
11091	ZC3H6	-0.330786836	3.98E-15
11092	RPP40	0.289989929	7.96E-12
11093	EDRF1	0.042623824	0.32509919
11094	ZNF606	-0.108914459	0.011708528
11095	KIF11	0.161670348	0.000173042
11096	MAGEE1	-0.342983672	3.25E-16
11097	RNF125	-0.191768568	7.94E-06
11098	PCDHB4	-0.123068759	0.004361285
11099	MYBL2	0.487667938	2.61E-33
11100	GINS2	0.298885722	1.68E-12
11101	CD274	-0.017074026	0.693560095
11102	VMAC	0.340490784	5.48E-16
11103	DDX26B	0.33080708	3.97E-15
11104	TAF4B	-0.134577719	0.001810268
11105	DHRS13	-0.090361651	0.036666467
11106	GFOD1	-0.167299274	0.000101062
11107	ZNF543	-0.388405898	1.04E-20
11108	CEP85	-0.003531241	0.935054625
11109	KCNMB4	0.069488453	0.108393862
11110	PCDHB5	-0.064379099	0.136974411
11111	NUDT13	0.034060091	0.431751708
11112	FANCI	0.140577234	0.001113499
11113	IQCC	0.227800994	9.99E-08
11114	CDKL2	-0.489818281	1.24E-33
11115	THAP10	-0.491285127	7.48E-34
11116	ZNF626	-0.384521856	2.69E-20
11117	C15orf52	0.156842253	0.000270686
11118	FAM86C1	0.277493191	6.48E-11
11119	TMEM120B	0.271564216	1.69E-10
11120	HIST1H3E	-0.003683608	0.932258931
11121	ZNF792	-0.29017784	7.70E-12
11122	PTPLAD2	-0.158838796	0.000225312
11123	LAMA1	-0.099458348	0.021402668
11124	LIG3	0.093766041	0.03011903
11125	ZNF570	-0.332101495	3.06E-15
11126	C1orf53	0.075727131	0.080120001
11127	GPR157	0.061528185	0.15526761
11128	WDR31	-0.302198499	9.25E-13
11129	ZMYND15	0.453279883	1.82E-28
11130	HACE1	-0.329158888	5.52E-15
11131	TBC1D7	0.381652946	5.39E-20
11132	GTDC1	-0.216131103	4.48E-07
11133	ERCC8	-0.332660632	2.73E-15
11134	LRP4	-0.22336415	1.78E-07

11135	NDUFAF6	0.185276374	1.61E-05
11136	ZNF2	-0.146073482	0.000701527
11137	G2E3	-0.333891455	2.13E-15
11138	PRICKLE4	0.416112389	8.12E-24
11139	FGFBP2	0.005824798	0.893075231
11140	DUS4L	-0.193955744	6.22E-06
11141	FRRS1	-0.232215218	5.54E-08
11142	ZNF417	-0.09318905	0.03115253
11143	RAC3	0.32834774	6.49E-15
11144	C9orf85	-0.177665801	3.58E-05
11145	MMP19	0.168913455	8.63E-05
11146	BEX1	-0.048412131	0.263647197
11147	CADM3	-0.024112961	0.577861705
11148	PTBP2	0.118455019	0.006085698
11149	OXNAD1	-0.203603723	2.05E-06
11150	TLCD2	0.153873743	0.000354156
11151	CDHR1	0.104507141	0.015596287
11152	CABP1	0.17030822	7.53E-05
11153	TRO	0.1972667	4.28E-06
11154	EZH2	0.446063956	1.61E-27
11155	AMN1	-0.39859565	8.10E-22
11156	GPRC5A	0.115178767	0.007659054
11157	TUBA3D	0.130130691	0.002563663
11158	ADAMTS15	0.096438966	0.02570606
11159	RTN4RL2	0.131579182	0.002291573
11160	ZNF605	-0.01563963	0.71815802
11161	C8orf88	-0.271535748	1.70E-10
11162	ZMYM6	0.004000694	0.926443597
11163	EVC2	-0.124198322	0.00401293
11164	EID2B	0.259919559	1.04E-09
11165	DGKA	0.426086559	5.22E-25
11166	ITGAE	0.508993318	1.33E-36
11167	ZNF300	0.157327244	0.000258938
11168	PRDM10	-0.247638444	6.43E-09
11169	GLTSCR1	0.559205692	2.44E-45
11170	SPIN4	-0.213707308	6.06E-07
11171	KBTBD3	-0.470991381	6.82E-31
11172	GSTZ1	0.176526964	4.03E-05
11173	C16orf87	-0.159998262	0.000202337
11174	SLC10A7	-0.442402	4.79E-27
11175	USP31	0.03196369	0.460647192
11176	BAIAP3	0.024594252	0.570291657
11177	ZNF17	-0.27797037	6.00E-11
11178	ZNF684	-0.221752973	2.20E-07
11179	DNAJC12	0.143539735	0.000869785
11180	DCLK1	-0.020176322	0.641476411
11181	VANGL2	0.05267597	0.223835041
11182	ANXA2R	0.361061536	6.44E-18
11183	LAMA3	0.009465894	0.827086652
11184	GAPT	-0.030393817	0.482973078
11185	GKAP1	-0.041758726	0.335024103
11186	C1orf131	0.338748833	7.86E-16
11187	C2CD2L	0.492596077	4.74E-34
11188	SEMA7A	0.267336643	3.30E-10
11189	CMC2	0.172889844	5.82E-05
11190	MPP7	-0.410020058	4.15E-23
11191	CYP26B1	-0.106293725	0.013901349
11192	PLEKHH1	-0.044167199	0.307871494

11193	ZNF117	0.21772711	3.67E-07
11194	PHACTR1	-0.03942901	0.362708342
11195	UVSSA	0.230151852	7.31E-08
11196	PLA2R1	-0.13058213	0.002475858
11197	ZSWIM3	0.011662479	0.78782801
11198	ZNF569	-0.340214149	5.80E-16
11199	GAB3	0.188422756	1.15E-05
11200	TMEM52B	-0.224164011	1.61E-07
11201	TUBB4A	0.107460674	0.012883839
11202	TENM1	0.159179934	0.00021831
11203	ERCC6L2	-0.441676193	5.93E-27
11204	SLA2	0.318175127	4.74E-14
11205	TRIM16	0.132761113	0.00208939
11206	SCIMP	-0.014407726	0.739519698
11207	PLEKHA8	-0.307135184	3.77E-13
11208	ZNF141	-0.156834122	0.000270887
11209	HUNK	-0.250822983	4.05E-09
11210	ZBTB25	0.168850676	8.69E-05
11211	AC245100.1	0.351520311	5.27E-17
11212	NLRC3	0.167241106	0.000101634
11213	ZNF287	-0.42559727	5.99E-25
11214	ATAD3C	0.10305858	0.017101017
11215	SLC25A33	-0.028642138	0.508560584
11216	ZNF821	-0.148954845	0.000547079
11217	LOH12CR1	-0.208641331	1.13E-06
11218	ZP3	0.317946889	4.95E-14
11219	EXD3	0.515471902	1.20E-37
11220	RALGAPA1	-0.371041348	6.62E-19
11221	LRRN4	-0.032406142	0.454460452
11222	EPM2A	-0.451703996	2.94E-28
11223	TLE4	-0.145737705	0.000721944
11224	ZNF57	-0.178197666	3.39E-05
11225	ZNF853	-0.097121591	0.024672792
11226	GPR132	0.298562118	1.78E-12
11227	ZNF525	-0.329159892	5.52E-15
11228	FEZ1	0.037824271	0.382584539
11229	EBF2	-0.217422646	3.81E-07
11230	EIF5A2	-0.18429242	1.79E-05
11231	CD6	0.330933674	3.87E-15
11232	WDR76	0.024161299	0.577099301
11233	ZNF473	-0.090494549	0.036389859
11234	TTC7B	-0.185812736	1.52E-05
11235	PTGS2	-0.110217302	0.010736891
11236	LENG9	0.396104981	1.52E-21
11237	ZNF383	-0.19803382	3.92E-06
11238	ADH6	-0.104389482	0.015714021
11239	DPT	0.075041843	0.082898784
11240	MPHOSPH9	-0.149830155	0.000506826
11241	TRIM9	0.053967401	0.212669585
11242	RNF207	0.509351948	1.17E-36
11243	DACT3	0.071948094	0.096427229
11244	BIRC5	0.474734274	2.01E-31
11245	FAM171B	-0.269166975	2.48E-10
11246	ZNF132	-0.33226838	2.96E-15
11247	MZB1	0.267376767	3.28E-10
11248	ADAMTS7	0.141335345	0.001045756
11249	TPK1	-0.039264524	0.364715489
11250	CD3G	0.0920775	0.033229247

11251	EME2	0.5423312	3.08E-42
11252	TGFB2	-0.36871206	1.13E-18
11253	PROSER3	0.321931254	2.29E-14
11254	ZNF780B	-0.138468665	0.001323761
11255	TTC9	-0.24872929	5.49E-09
11256	ROM1	0.375053966	2.59E-19
11257	ARHGEF35	-0.134451172	0.001828544
11258	KLHL17	0.640952476	3.17E-63
11259	SOX6	-0.370340202	7.79E-19
11260	STXBP5	-0.124132825	0.00403242
11261	SH2D1A	0.201458292	2.64E-06
11262	KNG1	-0.170595387	7.32E-05
11263	EDA	-0.337827001	9.51E-16
11264	ZNF74	0.292982896	4.74E-12
11265	AADAT	-0.107045781	0.013237813
11266	LILRA5	0.226254906	1.22E-07
11267	SH2D3A	0.266127904	3.99E-10
11268	USP6NL	-0.31323883	1.21E-13
11269	PTPRO	-0.098325715	0.022937458
11270	SPIN3	0.031674274	0.46471933
11271	TAL1	0.008498013	0.844529229
11272	WIPF3	0.100556365	0.020000717
11273	WNK4	0.076311468	0.077810615
11274	FZD2	0.337629697	9.90E-16
11275	SYNGAP1	0.34761841	1.22E-16
11276	GPS2	0.573735334	3.71E-48
11277	DENND1B	-0.314032524	1.04E-13
11278	ZNF236	-0.074904299	0.083465792
11279	RIN1	0.42406188	9.18E-25
11280	HDAC10	0.734238647	1.05E-91
11281	COL9A2	0.240500205	1.77E-08
11282	THAP9	-0.286077398	1.55E-11
11283	TSHZ3	-0.029334457	0.498363657
11284	ZNF484	-0.318439269	4.50E-14
11285	NABP1	0.175230079	4.60E-05
11286	PTPDC1	-0.047328071	0.274502087
11287	DPEP2	0.313146787	1.23E-13
11288	PIK3CG	-0.27380516	1.18E-10
11289	C9orf172	0.481435047	2.16E-32
11290	SP110	0.123599354	0.004194382
11291	TMEM229B	0.036467268	0.399901317
11292	CXCR6	0.164950091	0.000126761
11293	AK8	0.025279961	0.55958853
11294	CHEK2	0.404257863	1.88E-22
11295	USP45	-0.186997245	1.34E-05
11296	ZNF385B	-0.186657567	1.39E-05
11297	SEMA6D	-0.294256131	3.80E-12
11298	IQCA1	-0.057523934	0.184006174
11299	ZNF461	-0.212938948	6.66E-07
11300	GNAZ	0.085507232	0.048066093
11301	HIC1	0.261043883	8.76E-10
11302	PCDH10	-0.080890384	0.061527072
11303	CCNB2	0.379812218	8.39E-20
11304	CEP19	-0.017061348	0.693776143
11305	PRELID2	-0.121650659	0.004837227
11306	IRF9	0.4663179	3.07E-30
11307	GAREML	0.123014928	0.00437855
11308	ZNF347	-0.330960356	3.85E-15

11309	CHEK1		0.135158464	0.00172852
11310	NDUFAF5		0.116208179	0.007129423
11311	ZNF71		0.068801684	0.111936417
11312	N4BP3		-0.061210934	0.157412574
11313	CYP4F11		-0.034560293	0.425015286
11314	TMEM45B		-0.135650539	0.001661919
11315		1-Mar	-0.074997292	0.083082096
11316	SHISA9		-0.17515778	4.63E-05
11317	PPIP5K1		-0.056583954	0.191288604
11318	CLEC18A		0.099896128	0.020833797
11319	NTM		0.118861918	0.005912085
11320	PROM2		-0.073130945	0.091060386
11321	FMO5		-0.336000445	1.38E-15
11322	ZNF566		-0.271111522	1.82E-10
11323	COL21A1		-0.209595342	1.00E-06
11324	NCR3LG1		-0.345290515	2.00E-16
11325	DHRS11		0.15771406	0.000249913
11326	CNNM2		-0.396500045	1.38E-21
11327	SLC13A3		0.013847623	0.749299922
11328	TMEM67		-0.143350287	0.000883757
11329	DDTL		0.249283881	5.07E-09
11330	ZSCAN12		-0.288778252	9.80E-12
11331	CDCA8		0.405696442	1.30E-22
11332	TMEM17		-0.210968322	8.49E-07
11333	ANLN		0.180366693	2.71E-05
11334	ZNF789		0.338943951	7.55E-16
11335	COL8A2		0.071591815	0.098092283
11336	ORAOV1		0.475773899	1.42E-31
11337	PKIA		-0.177984806	3.47E-05
11338	SHPK		0.302344259	9.01E-13
11339	CAMKMT		0.134730825	0.001788379
11340	SNX20		0.232131358	5.60E-08
11341	BZRAP1		0.23756828	2.67E-08
11342	CORO7		0.591033723	1.07E-51
11343	ZNF551		-0.375716394	2.22E-19
11344	PNMA3		0.103896941	0.016215375
11345	PID1		-0.122973865	0.00439176
11346	ZFP2		-0.111952085	0.009554358
11347	CHKB		0.586402329	9.93E-51
11348	FXYD4		-0.121444887	0.004910051
11349	AGPAT4		0.069126633	0.110249048
11350	ABCA7		0.498959198	5.04E-35
11351	AP4S1		-0.138018646	0.001373122
11352	ZNF557		-0.063039609	0.14535157
11353	ZNF772		-0.446673658	1.34E-27
11354	CDT1		0.403840639	2.10E-22
11355	ZDHHC14		-0.005636638	0.896509531
11356	ZNF239		0.177306715	3.72E-05
11357	ATP6V0D2		-0.261340967	8.37E-10
11358	ZNF708		-0.109474611	0.011281629
11359	RAB7B		0.179775598	2.88E-05
11360	TRIM66		0.200553266	2.93E-06
11361	CYP4A22		-0.01678595	0.69847542
11362	AFF3		-0.13340184	0.001986736
11363	PGAP1		-0.410236614	3.92E-23
11364	IBA57		-0.367545062	1.48E-18
11365	IL6		0.223088847	1.85E-07
11366	IL7		-0.103090252	0.017066805

11367	C11orf63	-0.257175588	1.58E-09
11368	PIH1D2	-0.054770561	0.205931136
11369	ALG9	-0.092647654	0.032149721
11370	CCDC142	0.412467037	2.16E-23
11371	KIFC1	0.480922351	2.57E-32
11372	LSM11	-0.254648865	2.30E-09
11373	SPNS3	0.262717031	6.78E-10
11374	GPR3	0.085262889	0.04871082
11375	SEC61A2	0.431182871	1.24E-25
11376	CBX8	0.4716535	5.50E-31
11377	EIF4A1	0.408530396	6.16E-23
11378	PLG	-0.103335859	0.016803527
11379	USP35	0.420215372	2.66E-24
11380	CCDC65	0.133171162	0.00202315
11381	GDAP2	-0.324435824	1.41E-14
11382	ALG10B	-0.420445838	2.49E-24
11383	METTL6	-0.042818292	0.322894677
11384	DMD	-0.323915159	1.56E-14
11385	FLNC	0.140523928	0.001118411
11386	DACH1	-0.234123743	4.28E-08
11387	CD68	0.339868695	6.23E-16
11388	CCSAP	-0.078767279	0.068688932
11389	PDCD1	0.38701467	1.47E-20
11390	HSPA1L	-0.154295279	0.000340993
11391	KNTC1	0.246239854	7.87E-09
11392	CPEB3	-0.453145342	1.89E-28
11393	ATP6V0A2	-0.1993934	3.35E-06
11394	HYLS1	0.130145758	0.002560688
11395	TEX9	-0.316871921	6.08E-14
11396	MICU3	-0.208009191	1.21E-06
11397	OBSCN	0.190540021	9.10E-06
11398	GALNT3	-0.015731332	0.716576374
11399	PLCXD1	0.481650464	2.01E-32
11400	SARS2	0.615828705	3.64E-57
11401	SLC45A1	0.168685029	8.83E-05
11402	ZNF846	0.026638734	0.538671195
11403	XYLB	-0.136453154	0.001558321
11404	MAPK12	0.375503351	2.33E-19
11405	IFNLR1	-0.155362208	0.000309688
11406	LAMP3	0.09071761	0.035929576
11407	DNAJC6	-0.436081488	3.04E-26
11408	GAS1	-0.000420719	0.992253855
11409	ZNF563	-0.186699639	1.38E-05
11410	FATE1	0.130126878	0.002564417
11411	KIAA1107	-0.385478893	2.13E-20
11412	ACRBP	0.278671706	5.34E-11
11413	CD209	-0.060506732	0.162253644
11414	NEIL1	0.312749904	1.33E-13
11415	TTLL3	0.416541731	7.23E-24
11416	SIMC1	-0.083875733	0.052509701
11417	TNFSF8	-0.116994872	0.006747031
11418	SYT9	-0.085683842	0.04760455
11419	MLLT11	0.198041194	3.92E-06
11420	HECTD2	-0.066448102	0.124771281
11421	TMEM178A	0.163490481	0.0001457
11422	ZNF696	0.245577655	8.65E-09
11423	SLC12A1	-0.104848963	0.015258631
11424	L3MBTL4	-0.174745399	4.83E-05

11425	TESK2	-0.179272154	3.04E-05
11426	CHST9	-0.289057029	9.34E-12
11427	METTL8	-0.071297957	0.099482784
11428	HK3	0.386941032	1.49E-20
11429	MEIS1	-0.033228554	0.443085788
11430	CYTL1	-0.057243816	0.186154624
11431	POLE	0.498105495	6.82E-35
11432	TIAM1	-0.079873942	0.064874949
11433	ACP6	0.187976436	1.20E-05
11434	E2F5	0.12590813	0.003533382
11435	KIAA0586	-0.313157866	1.23E-13
11436	SORCS3	-0.056250731	0.193920351
11437	IKZF3	0.197582775	4.13E-06
11438	KDM8	0.253208782	2.85E-09
11439	RNASE2	0.169969861	7.78E-05
11440	RFX2	0.18980767	9.86E-06
11441	PMAIP1	0.04399837	0.309726123
11442	ZKSCAN3	-0.196000904	4.94E-06
11443	MTG1	0.469060159	1.27E-30
11444	ZFP69	0.057519165	0.184042593
11445	ZNF324	0.045944408	0.288793594
11446	ZBTB49	0.18616208	1.47E-05
11447	PLD4	0.081950599	0.058188321
11448	RGS9	0.1638266	0.000141117
11449	ZBTB39	-0.264485736	5.16E-10
11450	C8orf44	0.343952985	2.65E-16
11451	CYP8B1	-0.083260101	0.054273144
11452	TDRKH	-0.139033473	0.00126412
11453	MKI67	0.262712635	6.78E-10
11454	ZNF518B	-0.343906734	2.68E-16
11455	CFAP53	0.066202248	0.126175478
11456	XCL2	0.335912168	1.41E-15
11457	ZNF222	-0.210335841	9.17E-07
11458	BANK1	-0.24458024	9.97E-09
11459	IL1RAP	-0.248163209	5.96E-09
11460	CLEC18C	0.094123516	0.029493579
11461	C3orf36	-0.054405403	0.208975357
11462	SPTBN2	-0.057503958	0.184158777
11463	RAB19	-0.246157104	7.96E-09
11464	FANK1	0.145407019	0.000742588
11465	ZNF100	-0.245725611	8.47E-09
11466	FDX1L	0.514837592	1.52E-37
11467	SPHK1	0.492155031	5.53E-34
11468	KCNJ1	-0.186614469	1.40E-05
11469	TMEM178B	-0.156180419	0.000287522
11470	VILL	0.223916301	1.66E-07
11471	ASPHD2	0.161450041	0.000176661
11472	KIF20A	0.403826432	2.11E-22
11473	ZC2HC1C	-0.091824062	0.033718946
11474	REM1	0.086832848	0.04469166
11475	ZNF443	-0.071681967	0.097668811
11476	KLHDC1	-0.080757053	0.06195793
11477	AGAP6	0.529964565	4.49E-40
11478	EFCAB7	-0.178279452	3.37E-05
11479	ARHGAP44	-0.015647166	0.718027986
11480	RAB30	-0.390071405	6.90E-21
11481	ERCC4	-0.454749138	1.16E-28
11482	C4orf36	-0.267747995	3.10E-10

11483	CENPM	0.503533033	9.76E-36
11484	ARMC9	-0.026236574	0.544821213
11485	CENPN	0.062941224	0.145981968
11486	C6	-0.114797181	0.007864142
11487	CES1	-0.112046221	0.009493636
11488	NEURL3	0.278821753	5.21E-11
11489	STOX2	-0.210039645	9.50E-07
11490	SLC6A1	0.059019552	0.172844174
11491	GK5	-0.127543219	0.003124047
11492	GPAM	-0.416399809	7.51E-24
11493	SIRPG	0.343761353	2.76E-16
11494	RTTN	-0.024584426	0.570445744
11495	PLCH1	-0.258544561	1.28E-09
11496	PSTPIP1	0.49500531	2.04E-34
11497	MUC3A	0.215498008	4.85E-07
11498	MB21D1	0.209199394	1.05E-06
11499	GLT8D2	0.019365326	0.654936056
11500	THSD4	-0.198990457	3.51E-06
11501	TMEM255A	-0.256742861	1.68E-09
11502	CREB3L1	0.111432031	0.009896077
11503	SPATA33	0.514111396	1.99E-37
11504	ARHGAP28	-0.357820486	1.33E-17
11505	VSIG2	0.090429539	0.036524944
11506	LARGE	-0.222346785	2.04E-07
11507	ANAPC1	-0.360770504	6.87E-18
11508	TIMM23B	0.065969139	0.127518186
11509	ZAP70	0.534191443	8.37E-41
11510	RP11-11N7.5	0.0114758	0.791145117
11511	CRTAC1	-0.1844811	1.75E-05
11512	ALS2CL	0.163679155	0.00014311
11513	IL1RL1	0.010108077	0.815559825
11514	RABL2A	0.3816848	5.35E-20
11515	SLC6A9	0.151733228	0.000428623
11516	IP6K3	0.0809663	0.061282859
11517	SCG2	0.122679035	0.004487676
11518	PYURF	0.093730152	0.030182446
11519	DCLRE1C	0.141221197	0.001055707
11520	TAF5	0.042313261	0.328639982
11521	KIAA1377	-0.09399443	0.029718136
11522	ZNF70	0.018179825	0.674812979
11523	TMEM180	0.533545704	1.08E-40
11524	KCNMB1	0.173824434	5.30E-05
11525	FANCE	0.095514435	0.027164858
11526	IL12RB1	0.305346309	5.23E-13
11527	TRPC6	-0.225176858	1.41E-07
11528	HIST1H2BG	0.234148894	4.26E-08
11529	COL4A5	-0.010420706	0.809962507
11530	ZNF112	-0.362367745	4.80E-18
11531	LRRC10B	0.077452065	0.0734586
11532	STAC2	-0.225304059	1.39E-07
11533	ALPI	0.06631494	0.125530323
11534	INTS6	-0.201448123	2.64E-06
11535	ZC3H10	-0.038114128	0.378945963
11536	CDC7	0.315173405	8.39E-14
11537	LY75	0.065637147	0.129449565
11538	DUSP19	-0.291998321	5.63E-12
11539	KIF20B	-0.182145414	2.25E-05
11540	LYSMD4	0.129986339	0.002592334

11541	PIGL	0.425649347	5.90E-25
11542	RTN1	-0.044043498	0.309229662
11543	ZNF594	0.019486235	0.652922144
11544	MAATS1	0.065077992	0.13275358
11545	ADORA1	0.154025167	0.000349374
11546	SLC16A14	-0.324292791	1.45E-14
11547	CHFR	0.526850098	1.53E-39
11548	RHCG	-0.184864996	1.68E-05
11549	CASC10	0.026373051	0.542730248
11550	ZBTB3	0.036384512	0.40097236
11551	FUT1	-0.213115025	6.52E-07
11552	ZNF354C	-0.295487481	3.06E-12
11553	SOSTDC1	-0.176012075	4.25E-05
11554	SHH	0.081608421	0.059249079
11555	MARVELD3	-0.076553321	0.076870732
11556	CCDC68	-0.268228597	2.87E-10
11557	SIGLEC7	0.040646609	0.348065801
11558	ARID3A	0.162975301	0.000152996
11559	AGBL3	0.084834214	0.049859425
11560	GRAMD1B	0.053726525	0.214721133
11561	BCDIN3D	-0.144543823	0.00079908
11562	PNPLA7	0.35181906	4.94E-17
11563	SIRT4	0.0700839	0.105394508
11564	ZNF439	-0.107032865	0.013248969
11565	SLC19A1	0.443664176	3.30E-27
11566	MMP28	0.098429248	0.022793311
11567	GPR39	0.013095803	0.762491006
11568	KLC1	0.36585349	2.18E-18
11569	LRRC27	0.155649771	0.000301722
11570	CDNF	-0.017926089	0.679097441
11571	RHBDL1	0.458773248	3.33E-29
11572	EOMES	0.153838595	0.000355275
11573	DNAJC24	-0.345187299	2.04E-16
11574	REEP6	0.16011014	0.00020024
11575	OSBP2	0.001129588	0.979204425
11576	FIGN	-0.000302007	0.994439499
11577	ATP6V0A4	-0.276321981	7.85E-11
11578	OSM	0.152013899	0.00041809
11579	ZFP30	-0.236050993	3.29E-08
11580	F12	0.312916947	1.29E-13
11581	ACAD11	-0.262179309	7.36E-10
11582	C2orf88	-0.249462565	4.94E-09
11583	CENPO	0.28275243	2.72E-11
11584	CNTLN	-0.245755545	8.43E-09
11585	ZNF577	0.007465744	0.863216111
11586	PLEKHB1	-0.019918025	0.645750756
11587	TLR8	-0.141400207	0.001040141
11588	PRRX1	0.024057839	0.578731699
11589	ABHD16A	0.283320361	2.47E-11
11590	UNKL	0.16087449	0.000186451
11591	TIMP4	-0.095159533	0.027743482
11592	CCDC64B	0.057749263	0.182291272
11593	THAP2	-0.349909433	7.46E-17
11594	EFCAB2	0.066534559	0.124280382
11595	TMOD1	-0.194145287	6.09E-06
11596	RAD51D	0.364096848	3.25E-18
11597	ZNF214	-0.374280413	3.11E-19
11598	PRR7	0.482386132	1.57E-32

11599	TMEM213	-0.270472674	2.01E-10
11600	ZNF670	-0.407859307	7.35E-23
11601	CORO2B	0.062727047	0.1473615
11602	SUV420H2	0.597871707	3.69E-53
11603	ESRRG	-0.371436291	6.04E-19
11604	ZNF720	-0.344934586	2.16E-16
11605	P2RY6	0.315670845	7.64E-14
11606	GINS1	0.193422299	6.61E-06
11607	CTD-2192J16.22	0.038285994	0.376798584
11608	CDKN2A	0.421284799	1.98E-24
11609	RAD51AP1	0.103682057	0.016438459
11610	ZNF107	0.022709477	0.600201533
11611	BBS5	0.204276871	1.90E-06
11612	B3GNT8	0.033949848	0.433244639
11613	TTF2	-0.060902943	0.159516268
11614	PPP1R3F	0.327959527	7.01E-15
11615	CMC1	0.074963819	0.083220044
11616	CPLX1	0.313482365	1.16E-13
11617	METTL22	0.502070769	1.65E-35
11618	ADAMDEC1	0.034770994	0.422196136
11619	FAM26E	-0.239736242	1.97E-08
11620	SLC25A35	0.252005166	3.40E-09
11621	HOXA6	-0.251726593	3.54E-09
11622	BCAS4	-0.042511361	0.326378534
11623	DTWD1	-0.1422369	0.000970154
11624	ATAD2B	-0.006824009	0.874872532
11625	TRIP13	0.430994641	1.31E-25
11626	MBLAC1	0.352655014	4.12E-17
11627	CHMP4A	0.513871871	2.18E-37
11628	SLIT2	-0.218582762	3.29E-07
11629	RFX3	-0.245281341	9.02E-09
11630	STRADA	0.339426736	6.83E-16
11631	TBX19	0.369726668	8.97E-19
11632	ENPP1	-0.07793663	0.071670804
11633	RSPH1	-0.01337206	0.757635702
11634	N4BP2	-0.298471627	1.80E-12
11635	TRMT10A	-0.241830829	1.47E-08
11636	AC090154.1	0.101544501	0.018808094
11637	ALB	0.036546505	0.398877442
11638	CEP55	0.369351544	9.78E-19
11639	PRR16	0.130546788	0.002482632
11640	FAHD2B	0.093574896	0.030458104
11641	NSUN7	-0.29944425	1.52E-12
11642	S1PR5	0.154759307	0.000327032
11643	TSPAN8	0.028060221	0.517215067
11644	PCDHB11	-0.093158708	0.031207708
11645	B4GALT6	-0.220086925	2.72E-07
11646	ZDHHC23	-0.236321983	3.17E-08
11647	UBE2QL1	-0.246383785	7.70E-09
11648	HS3ST2	-0.077996975	0.071450672
11649	HOPX	0.123364249	0.00426761
11650	EID3	0.066433697	0.124853217
11651	FAM161A	-0.232201281	5.55E-08
11652	LIN7B	0.540094175	7.70E-42
11653	ASPRV1	0.16814007	9.31E-05
11654	ALG14	-0.07428766	0.086046407
11655	SLC38A5	0.21649988	4.28E-07
11656	TOX3	-0.399535073	6.37E-22

11657	SLC26A6	0.540974686	5.37E-42
11658	ZNF554	-0.010034851	0.816872235
11659	HRASLS2	0.146313028	0.00068729
11660	BHLHB9	-0.344491026	2.37E-16
11661	L1CAM	0.066718065	0.123243417
11662	FAM19A5	-0.00352754	0.935122541
11663	TMEM117	-0.231752958	5.90E-08
11664	CEP97	-0.300871153	1.18E-12
11665	IKBKG	0.106628965	0.013602034
11666	OIT3	-0.085902978	0.047037043
11667	PCDHB6	-0.026453608	0.541497904
11668	P4HA3	0.198573364	3.68E-06
11669	ZNF407	-0.369912055	8.60E-19
11670	FKBP1B	0.134782634	0.001781027
11671	EPHB3	0.073000032	0.091642318
11672	KCNIP3	0.063171688	0.144508561
11673	SLC16A6	-0.044180989	0.307720333
11674	NLRP6	0.088125247	0.041595879
11675	SCN9A	-0.141706592	0.001013989
11676	GALNT9	0.064740731	0.134777693
11677	TRMT44	0.027601054	0.524097236
11678	XPNPEP2	0.019949089	0.645236076
11679	C16orf89	-0.122476836	0.004554546
11680	SLC17A9	0.415117039	1.06E-23
11681	ZNF200	-0.125685445	0.003592745
11682	NLRP3	-0.066767132	0.122967289
11683	MYOM2	0.091272225	0.034806544
11684	CA5B	-0.404220956	1.90E-22
11685	DAGLA	0.124453247	0.003937882
11686	RASGEF1A	0.206916106	1.39E-06
11687	TM6SF2	0.267596715	3.17E-10
11688	TMEM184A	0.349183527	8.72E-17
11689	LIN37	0.562019383	7.11E-46
11690	PRR11	0.176714603	3.95E-05
11691	PVALB	-0.210087273	9.45E-07
11692	HSPB7	-0.145062418	0.000764682
11693	HOXA7	-0.223523141	1.75E-07
11694	MCM9	-0.299262217	1.57E-12
11695	CCDC85C	-0.133880789	0.00191304
11696	ANKRD32	-0.116265766	0.007100787
11697	FAM126A	-0.068319542	0.114477399
11698	SYCE1L	0.447237507	1.14E-27
11699	ALDH1A2	-0.179025482	3.12E-05
11700	ANKRD26	-0.181158679	2.49E-05
11701	PDGFRA	-0.027280284	0.528932698
11702	ZNF154	-0.110749135	0.010361268
11703	TMEM100	-0.055902635	0.196697763
11704	OCLN	-0.414631919	1.21E-23
11705	CEP135	-0.004010118	0.926270816
11706	BTC	-0.315045185	8.60E-14
11707	PLCD4	0.149616304	0.000516398
11708	ZNF430	-0.304667605	5.91E-13
11709	TTC39C	0.095029259	0.02795852
11710	CHSY3	-0.14103099	0.001072482
11711	FGD2	0.210466215	9.02E-07
11712	C14orf28	-0.041670455	0.336047637
11713	AGAP10	0.400743069	4.67E-22
11714	IQCG	0.150952797	0.000459226

11715	WDFY2	0.02851145	0.510497607
11716	STPG1	-0.000791943	0.98541958
11717	PRKCB	-0.017965518	0.678430968
11718	KLKB1	0.217387127	3.83E-07
11719	STXBP4	-0.445331843	2.01E-27
11720	RAPGEF6	-0.149275886	0.000531982
11721	STAT4	0.329014463	5.68E-15
11722	FRAT1	0.031712428	0.464181359
11723	EIF3C	-0.330551003	4.18E-15
11724	SLC25A45	0.499813125	3.71E-35
11725	FUT10	-0.35488153	2.53E-17
11726	KIAA0101	0.428488096	2.66E-25
11727	NBEAL1	-0.488140506	2.22E-33
11728	OPHN1	-0.499156767	4.69E-35
11729	MARCO	0.137827826	0.001394559
11730	CYP39A1	-0.356493008	1.78E-17
11731	PERM1	0.179121602	3.08E-05
11732	GJC2	0.144519485	0.000800729
11733	FBXL2	-0.114441252	0.008059847
11734	MALL	-0.222894485	1.90E-07
11735	MISP	0.267854509	3.05E-10
11736	CLCNKA	-0.126419043	0.003400532
11737	TNFAIP8L3	-0.0988828	0.022171052
11738	KIAA1524	0.087562395	0.042921124
11739	RIPK3	0.282968487	2.62E-11
11740	DAO	0.030012726	0.488479811
11741	C15orf40	0.113343331	0.008691211
11742	CA13	-0.185804561	1.52E-05
11743	ZNF630	-0.00252322	0.953568743
11744	APOBEC3B	0.26933065	2.41E-10
11745	CCDC110	-0.347852171	1.16E-16
11746	ROR1	-0.350422333	6.68E-17
11747	EEF1A2	0.115617044	0.007429406
11748	SLC51A	0.079188652	0.067215601
11749	RPGRIP1L	-0.11358144	0.00855066
11750	AC104650.2	0.083149176	0.054596061
11751	CASC1	-0.232577339	5.28E-08
11752	DTX1	0.132740637	0.002092749
11753	XRRA1	-0.146994642	0.000648231
11754	C5orf56	0.244826658	9.63E-09
11755	ZC3H8	0.217330206	3.86E-07
11756	ZNF701	-0.148440868	0.000572079
11757	FAM47E-STBD1	-0.246170046	7.94E-09
11758	NIPSNAP3B	0.008055867	0.852523231
11759	GATA6	0.159839025	0.000205357
11760	NOVA1	0.06946263	0.108525444
11761	ZFP41	0.282129434	3.01E-11
11762	LCORL	-0.420934557	2.18E-24
11763	CCR7	0.169772747	7.94E-05
11764	FBXO5	0.083940145	0.052327987
11765	SLC26A9	0.091059144	0.035234422
11766	DBF4	0.03469648	0.423191871
11767	SCNN1G	0.099812369	0.02094161
11768	CHD7	-0.027039133	0.532582779
11769	MN1	0.016651701	0.700770387
11770	WDR78	-0.258318211	1.33E-09
11771	ZNF567	-0.106932285	0.013336126
11772	TMEM182	-0.438323427	1.59E-26

11773	PSRC1	0.539649722	9.23E-42
11774	KAL1	-0.30965236	2.36E-13
11775	CEP57L1	-0.142097218	0.000981527
11776	CLIC3	0.409359831	4.95E-23
11777	ZNF322	-0.442135001	5.18E-27
11778	BATF3	0.476994229	9.52E-32
11779	CBWD2	-0.200704414	2.88E-06
11780	FCGR2B	0.12065634	0.005198369
11781	B3GNT4	0.100171386	0.020482882
11782	RND1	0.09646913	0.025659626
11783	RECQL4	0.628125552	4.62E-60
11784	PCSK7	0.057940067	0.180848396
11785	ATAD3B	0.619376119	5.48E-58
11786	CYP24A1	0.092057395	0.033267872
11787	APOBEC3H	0.437997438	1.74E-26
11788	RELT	0.417522865	5.54E-24
11789	AGO3	-0.134686326	0.001794715
11790	P2RY12	-0.240267464	1.83E-08
11791	LURAP1	-0.250954946	3.97E-09
11792	DUSP18	0.188635399	1.12E-05
11793	ZNF471	-0.176926948	3.87E-05
11794	PHLPP2	-0.196233674	4.81E-06
11795	EMR2	0.005640196	0.896444587
11796	MEIS3	0.317648897	5.24E-14
11797	ANKRD13B	0.556558347	7.67E-45
11798	USP9Y	-0.061170542	0.157687267
11799	EXOG	0.065667189	0.129273868
11800	DNASE1	0.064124883	0.138535045
11801	TCF21	-0.044981202	0.299032491
11802	BANP	0.241476118	1.55E-08
11803	MRGPRF	0.07393232	0.08756245
11804	ZFY	-0.119868012	0.005501768
11805	SLC4A1	-0.227673254	1.02E-07
11806	ZNF391	-0.258981272	1.20E-09
11807	SLC11A1	0.423192733	1.17E-24
11808	RPGR	0.048081393	0.266927165
11809	HAUS7	0.329265884	5.40E-15
11810	LEAP2	0.184156094	1.82E-05
11811	HDAC4	-0.018236093	0.673864272
11812	MAMLD1	0.101794404	0.018516486
11813	NBPF1	-0.336108483	1.35E-15
11814	SLC35E3	-0.008818375	0.838746998
11815	ZNF839	0.081037679	0.061053972
11816	ST7L	-0.034671831	0.423521563
11817	AARSD1	0.490616772	9.43E-34
11818	SLC31A2	-0.132016871	0.002214705
11819	SMN1	0.364046881	3.29E-18
11820	SPATA5	-0.379363493	9.34E-20
11821	ZNF707	0.458664793	3.44E-29
11822	C1orf233	0.276152303	8.07E-11
11823	RAB25	-0.157248321	0.000260817
11824	TRIM10	0.026488249	0.540968411
11825	IL15	0.093586161	0.03043803
11826	FXYD1	0.079383918	0.066541704
11827	PKIB	-0.15580489	0.000297505
11828	MOGAT3	0.256089146	1.86E-09
11829	FAM24B	-0.026615685	0.539022727
11830	MAD2L1	-0.035698481	0.409917035

11831	SIM1	-0.431976789	9.88E-26
11832	TMEM132E	-0.020675596	0.633247936
11833	RNF150	-0.354793234	2.58E-17
11834	TMEM241	-0.080870408	0.061591469
11835	CXCL6	0.020293332	0.639543986
11836	CARF	-0.10450821	0.015595222
11837	CEP83	0.247230009	6.82E-09
11838	DFFB	0.360630396	7.09E-18
11839	ETV1	-0.11918012	0.005779433
11840	SLC38A11	-0.123727589	0.004154921
11841	CMYA5	-0.121577292	0.00486308
11842	AVPR1B	-0.157225295	0.000261368
11843	SLC12A8	0.158071665	0.000241831
11844	KALRN	-0.247353257	6.70E-09
11845	TFAP4	0.404164683	1.93E-22
11846	ATP10A	0.175055518	4.68E-05
11847	RFTN2	-0.212761454	6.81E-07
11848	TMEM238	0.279383491	4.75E-11
11849	RAB11FIP4	-0.252270334	3.27E-09
11850	CAMK1G	-0.037702185	0.38412346
11851	GPR114	0.114665104	0.007936262
11852	IQSEC3	-0.032256501	0.456547614
11853	P2RY10	0.046734429	0.280573568
11854	F5	-0.018987468	0.66124589
11855	BMP8B	-0.051385465	0.235403881
11856	EPS8L1	0.068546474	0.113275844
11857	PCDHB7	-0.059042795	0.172674787
11858	B3GALNT2	-0.287452775	1.23E-11
11859	ZNF30	0.088894864	0.03983985
11860	ZNF334	0.058421579	0.177244788
11861	GALNT6	0.128894609	0.002818809
11862	SCG5	0.118609197	0.006019383
11863	RAB33A	0.208303906	1.17E-06
11864	CHI3L2	0.126781855	0.003308963
11865	SLC9B2	-0.122838404	0.004435597
11866	NECAB2	0.232998506	4.98E-08
11867	CDKL1	-0.316318914	6.75E-14
11868	TSEN2	0.143089102	0.000903361
11869	TEX11	-0.031655891	0.464978656
11870	SPEF2	0.037220151	0.390236419
11871	CTU1	0.572997401	5.20E-48
11872	C21orf62	-0.185477922	1.58E-05
11873	RAD18	-0.155143048	0.000315891
11874	ADAP1	0.275415767	9.10E-11
11875	MCM8	-0.019612978	0.650813781
11876	CDKN3	0.365407454	2.41E-18
11877	DIRAS3	-0.179123257	3.08E-05
11878	GDPD5	0.344547232	2.34E-16
11879	ZNF502	-0.09223116	0.032935307
11880	WDR27	0.328533998	6.25E-15
11881	RUNX2	0.084091025	0.051904392
11882	KIF2C	0.438343614	1.58E-26
11883	CHTF18	0.659029505	6.01E-68
11884	GTF2IRD2B	0.078562674	0.069413819
11885	SPESP1	-0.005048861	0.907249883
11886	C1orf159	0.620761497	2.60E-58
11887	RPAP2	-0.137414148	0.001442093
11888	DBNDD2	0.245419856	8.85E-09

11889	PUS10	-0.032606831	0.451669705
11890	HSD11B1	0.091988228	0.033401044
11891	MATK	0.399837283	5.90E-22
11892	MYO3A	-0.002261148	0.958386673
11893	CXCL5	0.076231965	0.078121609
11894	ENAM	-0.346701654	1.48E-16
11895	MAGIX	0.151995794	0.000418762
11896	GUCA2B	0.189983703	9.67E-06
11897	MEI4	-0.285478917	1.72E-11
11898	PCDHB13	-0.154713761	0.000328378
11899	MAP1LC3B2	0.134943137	0.001758427
11900	PCNXL2	0.200102823	3.09E-06
11901	SVEP1	-0.199255993	3.41E-06
11902	ZNF165	-0.192904539	7.00E-06
11903	FO538757.2	0.504241802	7.55E-36
11904	TMEM150B	0.332316858	2.93E-15
11905	ZNF77	0.091303106	0.034744902
11906	ZNF597	-0.282317874	2.92E-11
11907	SLC34A1	-0.01498313	0.729515866
11908	MED26	0.480348599	3.11E-32
11909	FAM132A	0.538560476	1.44E-41
11910	SCNN1B	0.145508138	0.000736218
11911	CNFN	0.249777112	4.71E-09
11912	SPIN2B	0.18705818	1.33E-05
11913	USP37	-0.431219529	1.23E-25
11914	ANO4	-0.150497158	0.00047802
11915	CDHR3	0.161604721	0.000174113
11916	LRRC4	-0.277998447	5.97E-11
11917	C1QL3	-0.232913133	5.04E-08
11918	NLRC4	-0.028281526	0.513914799
11919	HARBI1	0.046043305	0.287755825
11920	CDCA5	0.368932465	1.08E-18
11921	FAM212B	-0.105293711	0.014828919
11922	AZIN2	0.245025145	9.36E-09
11923	C1orf95	-0.066031546	0.127157645
11924	ZIK1	-0.391084914	5.37E-21
11925	RGS4	-0.032998613	0.446249614
11926	CHRDL1	0.001972183	0.963700747
11927	RIBC1	0.184159015	1.82E-05
11928	PRLR	-0.291348593	6.30E-12
11929	HOMER1	-0.147080978	0.000643433
11930	ADCK1	0.224328989	1.57E-07
11931	FAM183A	0.252092553	3.36E-09
11932	SENP8	-0.479828668	3.71E-32
11933	SLC17A2	0.001306761	0.975943599
11934	ZNF124	-0.158933933	0.000223338
11935	POU2F2	0.337770603	9.62E-16
11936	SNCA	-0.191744117	7.96E-06
11937	GABRB3	-0.180741446	2.61E-05
11938	GCNT4	-0.282904421	2.65E-11
11939	TNFSF4	0.093365499	0.030833302
11940	SSTR1	-0.113973952	0.008323386
11941	SLC38A9	-0.217011028	4.01E-07
11942	SGIP1	-0.022571214	0.602423228
11943	ACOT11	-0.186389974	1.43E-05
11944	HCG27	0.341915042	4.07E-16
11945	ZNF85	-0.24295216	1.26E-08
11946	EXPH5	-0.37428864	3.10E-19

11947	DTL	0.084781749	0.050001543
11948	SIRPB2	0.087236948	0.043703509
11949	S100A12	0.152840555	0.00038845
11950	CD180	-0.085721314	0.047507102
11951	CYP2S1	0.314349699	9.81E-14
11952	ZNF229	-0.32513897	1.22E-14
11953	SPATA25	0.250361938	4.33E-09
11954	KLRF1	-0.0616609	0.15437691
11955	RTN4RL1	0.172151535	6.27E-05
11956	FTCDNL1	-0.161583836	0.000174455
11957	PRR15	-0.03817519	0.378182155
11958	PTPN22	0.135102623	0.001736231
11959	AQP11	-0.115731392	0.007370512
11960	METTL24	-0.316776702	6.19E-14
11961	ZNF805	-0.381937766	5.04E-20
11962	CENPJ	0.199272652	3.40E-06
11963	CTC-534A2.2	0.147267514	0.00063318
11964	KRT17	0.026464556	0.541330544
11965	GPRIN1	0.351053375	5.83E-17
11966	MAGI2	-0.226124845	1.25E-07
11967	FOXL1	0.187812242	1.23E-05
11968	C6orf132	-0.052732729	0.223335699
11969	ZFAND4	-0.144088657	0.000830445
11970	INTU	-0.167376077	0.000100311
11971	KLHL6	0.10759465	0.012771332
11972	LGALS12	0.028687305	0.507892034
11973	SAMD14	0.274830348	1.00E-10
11974	RNF183	-0.072910671	0.092041251
11975	NPHP3	0.37080244	7.00E-19
11976	MX2	0.228045164	9.67E-08
11977	NOL12	0.633524074	2.25E-61
11978	MAP10	-0.216977966	4.03E-07
11979	ARSF	-0.033872555	0.434293131
11980	ZSCAN22	0.11089094	0.010263104
11981	DLX5	0.196630045	4.60E-06
11982	PSMG4	0.443426151	3.54E-27
11983	EPHX3	-0.032958541	0.446802298
11984	IQGAP3	0.506978986	2.79E-36
11985	UBASH3B	-0.151088233	0.000453773
11986	LIPE	-0.040469341	0.350173961
11987	NPTXR	0.089941369	0.037553007
11988	ZNF485	0.048742746	0.260396264
11989	C3orf14	0.066586421	0.123986631
11990	PSMC3IP	0.346082048	1.69E-16
11991	MDFI	0.231225699	6.33E-08
11992	ZNF345	0.155372117	0.000309411
11993	TBX15	-0.006528992	0.880240455
11994	PDGFRL	0.2078876	1.23E-06
11995	PILRB	0.386020915	1.87E-20
11996	METTL20	-0.271376984	1.74E-10
11997	DSCC1	0.152703057	0.00039324
11998	SFTPD	-0.034947729	0.419839884
11999	FAM83G	0.068657754	0.11269027
12000	NAP1L3	-0.098000544	0.023395334
12001	PHF7	-0.010276447	0.812544138
12002	ATP2B2	-0.129847352	0.002620214
12003	GPM6A	-0.065702871	0.129065425
12004	NBPF9	0.093624041	0.030370614

12005	LRRC37B	0.281093233	3.58E-11
12006	NBPF12	0.082880322	0.055385374
12007	FASLG	0.253888892	2.58E-09
12008	NHLRC1	-0.055388426	0.200853551
12009	ATF7IP2	0.061712924	0.15402881
12010	KIAA1614	-0.049436095	0.253668691
12011	POC1A	0.35116555	5.69E-17
12012	GCNT1	-0.137086307	0.001480817
12013	TLDC2	0.307256442	3.68E-13
12014	CRIP1	0.460751042	1.79E-29
12015	LIME1	0.606471257	4.79E-55
12016	SGSM1	-0.230520435	6.96E-08
12017	USP43	0.318109696	4.80E-14
12018	GATSL2	-0.303753453	6.98E-13
12019	ZNF593	0.441770614	5.77E-27
12020	FBLN7	0.007947535	0.85448421
12021	UST	-0.190297943	9.34E-06
12022	GTF2H4	0.410342452	3.81E-23
12023	ZNF333	0.191677532	8.02E-06
12024	ATP6V1C2	-0.008008446	0.853381521
12025	CCDC152	-0.210130173	9.40E-07
12026	EFCC1	0.121890607	0.004753539
12027	IQCD	0.141548323	0.001027421
12028	PIP5K1B	-0.230122851	7.34E-08
12029	CCDC160	-0.217707368	3.68E-07
12030	ANGPTL1	-0.223838136	1.68E-07
12031	BTBD16	0.219460894	2.95E-07
12032	TRAF3IP3	0.284198596	2.13E-11
12033	CORO2A	0.09152916	0.034296499
12034	ZNF470	-0.327722225	7.35E-15
12035	NDC80	0.436438497	2.74E-26
12036	ZNF778	-0.289678053	8.40E-12
12037	SPC24	0.615301427	4.81E-57
12038	CLDN8	-0.240593695	1.75E-08
12039	CEP295	-0.066651561	0.123618435
12040	ZBTB37	-0.158223581	0.000238472
12041	SLC2A4	-0.167058817	0.000103448
12042	DUSP28	0.388116878	1.12E-20
12043	C10orf107	-0.164000754	0.000138796
12044	ARHGEF18	-0.097820314	0.023652509
12045	KIAA1324L	-0.25701409	1.61E-09
12046	ZNF879	-0.212153875	7.34E-07
12047	SLC52A3	-0.002835246	0.947834688
12048	TUBB2B	-0.024784487	0.567312575
12049	CLEC5A	-0.03298995	0.446369061
12050	KATNAL2	-0.165640474	0.000118633
12051	PTCD2	-0.336944249	1.14E-15
12052	PRR29	0.100717901	0.019801364
12053	ZNF619	-0.495965204	1.45E-34
12054	HES6	0.459407708	2.73E-29
12055	PAQR3	-0.321069346	2.71E-14
12056	SLC7A6	0.146320179	0.000686869
12057	MPZ	0.364021643	3.31E-18
12058	RMDN2	-0.245297104	9.00E-09
12059	ANKRD65	-0.022830139	0.598265666
12060	SLX4	0.328788688	5.94E-15
12061	HEMK1	0.297001424	2.34E-12
12062	PRR22	0.603212146	2.53E-54

12063	PRRG2	-0.150337353	0.000484779
12064	CEP85L	-0.162668664	0.000157501
12065	NR5A2	-0.214326071	5.61E-07
12066	C15orf61	-0.023706137	0.584297043
12067	ZNF135	-0.156836089	0.000270838
12068	OLFM1	0.155567205	0.00030399
12069	SV2A	0.117996181	0.006286954
12070	BRF1	0.34959292	7.98E-17
12071	SNPH	0.136826926	0.001512129
12072	ZNF493	-0.091264289	0.034822401
12073	DDHD1	-0.027240844	0.529528784
12074	CCDC148	-0.158781279	0.000226513
12075	TTLL11	-0.11363708	0.00851811
12076	NMUR1	0.09900044	0.022012081
12077	LCN12	0.124383123	0.003958398
12078	IHH	0.127901475	0.003040329
12079	PRR26	-0.024083036	0.578333938
12080	ABLIM2	0.139886685	0.001178702
12081	AMY2B	0.359796057	8.54E-18
12082	ALG11	-0.478661265	5.47E-32
12083	CLEC12A	0.172714091	5.93E-05
12084	NRG3	-0.239232816	2.12E-08
12085	WDFY4	-0.04473634	0.30167343
12086	ZNF324B	0.049597549	0.252119577
12087	FBXO48	-0.243408276	1.18E-08
12088	MROH6	0.536541936	3.26E-41
12089	ZNF596	-0.087371427	0.043378772
12090	SLC15A1	0.016264664	0.707401913
12091	TMEM26	-0.01610457	0.710151507
12092	NECAB1	-0.201561327	2.61E-06
12093	PTCH1	-0.413913398	1.47E-23
12094	AMBP	-0.005194663	0.904584012
12095	SRGAP2C	-0.098179438	0.023142462
12096	TMPRSS3	-0.056398783	0.192747804
12097	ZNF782	-0.133825152	0.001921471
12098	SASS6	-0.051610107	0.233360295
12099	PPFIA3	0.317092541	5.83E-14
12100	HOXD11	0.120725902	0.005172335
12101	GPR171	0.267052869	3.45E-10
12102	CHAC1	0.2287877	8.77E-08
12103	PRAM1	0.433884726	5.72E-26
12104	LONRF3	-0.133849854	0.001917723
12105	CTAGE5	-0.049946224	0.24879659
12106	ZNF610	-0.285399562	1.74E-11
12107	ZNF837	0.3466326	1.50E-16
12108	GC	0.015365247	0.722897623
12109	NBPF3	-0.350455719	6.63E-17
12110	PRDM11	-0.159833192	0.000205468
12111	FAM46B	0.029286079	0.499072662
12112	CBLN4	-0.006385438	0.882854465
12113	HIC2	0.092472755	0.032477642
12114	GPSM2	-0.141286741	0.001049983
12115	NBPF11	0.197610523	4.11E-06
12116	EPHA1	0.120474366	0.005267034
12117	DUSP15	-0.175128324	4.65E-05
12118	ARHGAP22	0.398416821	8.48E-22
12119	CD300E	0.225299798	1.39E-07
12120	GPR37	-0.164053975	0.000138094

12121	CCDC74B	0.318390799	4.54E-14
12122	DOK6	-0.299575079	1.48E-12
12123	MCTP1	-0.146843264	0.000656724
12124	PPIL6	-0.241789424	1.48E-08
12125	CASS4	-0.066210917	0.126125756
12126	CD1C	0.092832302	0.031806598
12127	TAF1A	-0.083086349	0.054779669
12128	CPAMD8	-0.019985869	0.644626914
12129	ZNF624	-0.338305252	8.61E-16
12130	SCLT1	0.001002068	0.981551602
12131	ATP1A2	-0.196569524	4.63E-06
12132	PAQR6	0.580255309	1.82E-49
12133	CRYGS	0.462968155	8.91E-30
12134	XYLT1	-0.147151623	0.000639532
12135	MRO	-0.171956664	6.39E-05
12136	RHOV	0.137084403	0.001481045
12137	SARM1	-0.066546793	0.124211035
12138	ZNF738	0.241023217	1.65E-08
12139	AURKB	0.602695247	3.28E-54
12140	ST8SIA6	-0.200686193	2.89E-06
12141	ZNF419	0.105827575	0.014327177
12142	SLC7A6OS	-0.182490736	2.17E-05
12143	RP11-192H23.4	0.230496061	6.98E-08
12144	ARMCX4	0.016164368	0.709124043
12145	PPAP2C	0.282257621	2.95E-11
12146	ZNF568	-0.317912541	4.98E-14
12147	PYHIN1	0.229106231	8.40E-08
12148	TMSB15A	0.023452165	0.588331269
12149	GPR27	-0.036497876	0.399505622
12150	CACNA1C	-0.04178843	0.334680135
12151	AMER1	-0.225318964	1.38E-07
12152	KLHL11	-0.482325253	1.60E-32
12153	KCNK13	-0.062630618	0.147985846
12154	CDK11A	0.549150543	1.80E-43
12155	C18orf54	-0.162246434	0.000163907
12156	CH25H	0.0421928	0.330020074
12157	ZNF749	-0.220580659	2.55E-07
12158	CALCR	-0.263974524	5.58E-10
12159	PIK3R6	0.247217821	6.83E-09
12160	IL2RA	0.085535151	0.047992882
12161	BARD1	-0.003567247	0.934393886
12162	NINJ2	0.081251177	0.060373568
12163	CEACAM21	0.221594812	2.24E-07
12164	ADAM12	0.07909628	0.067536342
12165	FAM124A	0.113264852	0.008737985
12166	LRRN2	-0.122183773	0.004653065
12167	OVGP1	0.227757084	1.00E-07
12168	GDPD1	-0.106106489	0.014071031
12169	LHX6	-0.02845694	0.511306688
12170	ZNF230	-0.259155721	1.17E-09
12171	CRHBP	-0.118715623	0.005973986
12172	GSDMB	0.559854584	1.84E-45
12173	NCF1	0.304610034	5.98E-13
12174	DZANK1	0.150372089	0.000483303
12175	ZNF431	-0.099943275	0.020773323
12176	PBK	0.231642252	5.99E-08
12177	ATXN7L2	0.520677955	1.66E-38
12178	THPO	0.092278207	0.032845753

12179	SPC25	0.398216091	8.92E-22
12180	PCP4	-0.1661337	0.000113129
12181	ACTR3B	0.050778741	0.240986318
12182	ACHE	0.428189794	2.89E-25
12183	ADRA2A	0.004124653	0.924171244
12184	GSTT2B	0.172577643	6.01E-05
12185	TPSG1	0.186542639	1.41E-05
12186	FMN1	-0.44512979	2.13E-27
12187	UCN	0.650993998	8.28E-66
12188	ZKSCAN2	-0.407821275	7.42E-23
12189	CD163L1	0.012395121	0.774847183
12190	SELE	-0.162835255	0.000155038
12191	IL13RA2	-0.022512303	0.603370976
12192	ARHGAP39	0.325918465	1.05E-14
12193	KLHL29	0.211589414	7.87E-07
12194	CCDC96	0.219446411	2.95E-07
12195	GPR35	0.27712896	6.88E-11
12196	LRRC37A3	0.319589269	3.61E-14
12197	MELK	0.358433819	1.16E-17
12198	MGAT2	-0.119054068	0.005831658
12199	ARHGAP11A	0.115802038	0.007334335
12200	LIN9	-0.184789051	1.70E-05
12201	MYOZ1	-0.045926855	0.288978058
12202	TNXB	-0.151155262	0.000451097
12203	ZNF527	-0.214632876	5.40E-07
12204	WNT5A	0.009146639	0.832831118
12205	AK7	-0.316587194	6.42E-14
12206	C1orf74	-0.22714612	1.09E-07
12207	PCSK4	0.461600003	1.37E-29
12208	DGKE	-0.195794671	5.06E-06
12209	FAM160A1	-0.521609651	1.16E-38
12210	GRM8	-0.223535112	1.75E-07
12211	SPEF1	0.188674735	1.12E-05
12212	ACVR2B	-0.345737469	1.82E-16
12213	ADCY7	0.038703833	0.371609193
12214	ULBP2	0.093880902	0.029916838
12215	CCL14	0.118692244	0.005983932
12216	MARK1	-0.167602706	9.81E-05
12217	FAM111B	0.015437266	0.721652561
12218	GLI3	0.002287069	0.957910075
12219	THBS4	-0.049178343	0.256155427
12220	CD3EAP	0.400848953	4.55E-22
12221	ISPD	-0.510534547	7.54E-37
12222	CACNB2	-0.126090609	0.003485402
12223	UFSP1	0.481511429	2.11E-32
12224	ANKMY1	0.33596714	1.39E-15
12225	CFAP221	-0.096045771	0.026318005
12226	PMF1-BGLAP	0.40776867	7.53E-23
12227	FAM189A2	-0.117892624	0.006333195
12228	RNF212	0.002816588	0.948177485
12229	ACKR4	0.010150599	0.814797959
12230	ZNF487	0.233878373	4.42E-08
12231	SGCA	0.069038357	0.110705444
12232	C1orf226	-0.121772048	0.004794725
12233	MOCOS	0.230328355	7.14E-08
12234	CD38	0.18438659	1.77E-05
12235	GPR161	-0.095861163	0.026609621
12236	PROC	-0.114314843	0.008130391

12237	TTLL7	-0.10238327	0.017844903
12238	NRG1	-0.124810427	0.003834869
12239	IL17RE	-0.019643112	0.650312913
12240	PROB1	0.099565785	0.021261824
12241	MFSD2A	0.264766473	4.94E-10
12242	RASL11B	-0.009600666	0.824664402
12243	ZNF225	-0.286407124	1.47E-11
12244	GPR146	-0.170925163	7.08E-05
12245	FNDC1	-0.045834333	0.289951631
12246	TYW1B	0.063185108	0.144423112
12247	LAMA2	-0.152317388	0.000406972
12248	NEGR1	-0.139251796	0.001241736
12249	TAMM41	0.103741419	0.016376564
12250	ZNF699	-0.474154326	2.43E-31
12251	GIMAP5	0.012264418	0.777158487
12252	HTR2B	-0.093885685	0.029908442
12253	UPK1B	-0.075147205	0.082466555
12254	ZDBF2	-0.271326486	1.76E-10
12255	CLDN19	-0.217293121	3.87E-07
12256	GGT6	-0.28450796	2.02E-11
12257	RUNDC3B	-0.274094263	1.13E-10
12258	C19orf40	0.401121109	4.24E-22
12259	IRAK1BP1	-0.401460377	3.89E-22
12260	NPHP1	-0.026407719	0.542199744
12261	MOK	0.402972069	2.63E-22
12262	ZNF337	0.305565286	5.02E-13
12263	IGF2BP2	0.123417334	0.004250975
12264	TEC	-0.196249085	4.80E-06
12265	ENO3	0.412244393	2.30E-23
12266	PTCD1	0.589328748	2.43E-51
12267	AKR1C2	0.011099166	0.797849014
12268	CNTN4	-0.197346879	4.24E-06
12269	ZNF814	0.210193977	9.33E-07
12270	KIF9	0.019436166	0.653755808
12271	RABGEF1	0.189337678	1.04E-05
12272	CCDC40	0.464949421	4.75E-30
12273	INPP5J	-0.184691539	1.72E-05
12274	PSPN	0.264733456	4.96E-10
12275	CHAC2	0.097192586	0.024567417
12276	DYNC1I1	-0.186505167	1.41E-05
12277	PXDNL	-0.105485566	0.014646857
12278	CENPF	0.271409437	1.73E-10
12279	DYNLRB2	0.10461309	0.015490935
12280	ZNF675	-0.273703027	1.20E-10
12281	PSORS1C1	0.307354718	3.62E-13
12282	PASK	0.257987649	1.39E-09
12283	CYSLTR1	-0.323284379	1.76E-14
12284	CCZ1	0.088637498	0.040419966
12285	CCDC66	0.190452002	9.18E-06
12286	EYA2	0.094527403	0.028800361
12287	UTY	-0.10864342	0.011920174
12288	RGS7BP	-0.294778309	3.46E-12
12289	RP11-345J4.5	-0.232020521	5.69E-08
12290	ZNF677	-0.359157893	9.85E-18
12291	GATSL3	0.427267648	3.75E-25
12292	TNFRSF9	0.195165466	5.43E-06
12293	AGAP2	0.404370938	1.83E-22
12294	BAG2	-0.042954302	0.321358633

12295	ARNTL2	0.149857562	0.000505611
12296	TCEANC2	-0.175230891	4.60E-05
12297	TBX6	0.373866141	3.43E-19
12298	GNRH1	0.451086965	3.55E-28
12299	FAM13C	-0.322136953	2.20E-14
12300	ANKDD1B	-0.078726688	0.068832245
12301	ZNF799	0.080100846	0.064114878
12302	ZNF599	0.056036097	0.195629475
12303	ZNF155	-0.29549752	3.05E-12
12304	SOWAHD	0.299758259	1.43E-12
12305	B4GALNT3	-0.127281897	0.003186429
12306	TMEM169	-0.166028172	0.000114286
12307	SLC12A3	-0.191603044	8.09E-06
12308	ZNF678	-0.255977311	1.89E-09
12309	KIF4A	0.336167824	1.34E-15
12310	TRIM45	0.308286283	3.05E-13
12311	CYP2C8	0.156997669	0.000266868
12312	SHCBP1	0.228955908	8.57E-08
12313	DAPP1	-0.023824362	0.582423467
12314	SLC19A3	0.046444509	0.283571534
12315	MYRIP	-0.329289702	5.38E-15
12316	RASL10B	0.141931877	0.000995148
12317	SAMD15	-0.225574902	1.34E-07
12318	RAPGEFL1	0.411187547	3.05E-23
12319	GRAP	-0.027012381	0.532988471
12320	C8orf22	0.079844892	0.06497279
12321	ACTR3C	-0.176820116	3.91E-05
12322	CD33	0.167016655	0.000103871
12323	IPO4	0.538729493	1.34E-41
12324	MYBL1	0.150738826	0.000467964
12325	PCDHB9	-0.019288672	0.656214122
12326	GDPGP1	0.326647586	9.09E-15
12327	PLEKHG6	0.020227405	0.640632469
12328	SNAI3	0.365881626	2.17E-18
12329	SERAC1	-0.315826073	7.42E-14
12330	RP11-644F5.10	0.408804456	5.73E-23
12331	TRPM3	-0.165053408	0.000125513
12332	PLEKHN1	0.318331858	4.60E-14
12333	PLCXD2	-0.023762307	0.583406539
12334	ATP8B3	0.153403369	0.000369403
12335	LRRC73	0.214836373	5.27E-07
12336	GLRB	-0.190248954	9.39E-06
12337	PRSS35	-0.005172514	0.904988922
12338	DNAH1	0.390528044	6.16E-21
12339	ZNF8	-0.01250099	0.77297651
12340	DISC1	-0.208173813	1.19E-06
12341	PLK1	0.488986761	1.66E-33
12342	CEP72	0.1578867	0.00024598
12343	DNER	0.101655924	0.018677586
12344	LIX1	-0.222673924	1.95E-07
12345	SRGAP1	0.055208816	0.202320092
12346	TONSL	0.542012449	3.51E-42
12347	STARD9	0.039294048	0.364354718
12348	ROR2	-0.002823643	0.948047871
12349	DNMT3B	0.391145289	5.29E-21
12350	IGFLR1	0.538688362	1.37E-41
12351	DPH6	-0.315393454	8.05E-14
12352	STAP1	-0.17048563	7.40E-05

12353	SLC22A7	0.03906637	0.367142635
12354	FAM227B	-0.307342835	3.62E-13
12355	TNFSF14	0.343903146	2.68E-16
12356	DPF3	0.234306666	4.17E-08
12357	DEGS2	0.047873872	0.26899941
12358	HYAL3	0.302910217	8.14E-13
12359	LRRC75A	-0.106552658	0.01366966
12360	NPHP4	0.134494662	0.001822244
12361	SPRN	0.530342062	3.87E-40
12362	ANKDD1A	0.316385474	6.67E-14
12363	KLHL32	-0.36465109	2.87E-18
12364	TMPRSS2	-0.191147929	8.51E-06
12365	SAMD12	-0.413033358	1.86E-23
12366	LRRC4B	0.224142814	1.61E-07
12367	OR51E2	-0.071281171	0.099562686
12368	GPR68	0.320119184	3.26E-14
12369	ZFP82	-0.301012287	1.15E-12
12370	TPGS1	0.381378874	5.76E-20
12371	NRDE2	-0.076878406	0.07562195
12372	GPM6B	-0.102130267	0.018130793
12373	ITK	0.151306721	0.000445104
12374	PYGM	0.027758383	0.521733867
12375	CYB5D1	-0.253822966	2.60E-09
12376	RGMB	-0.06883912	0.111740997
12377	MYOM1	-0.128275808	0.002955038
12378	CBLC	0.132911749	0.002064829
12379	GPR162	0.42197637	1.64E-24
12380	ZNF540	-0.062050304	0.151785803
12381	MTRNR2L8	0.095807622	0.026694717
12382	MTMR3	-0.297700898	2.07E-12
12383	PRICKLE1	-0.145167504	0.000757881
12384	ZNF813	-0.405009759	1.55E-22
12385	ALOX12	0.221659743	2.22E-07
12386	LRRC49	-0.250251998	4.40E-09
12387	RASSF9	-0.378056987	1.27E-19
12388	ADORA2B	0.17228427	6.19E-05
12389	OGN	-0.048141771	0.266326311
12390	SLC15A2	-0.074566054	0.084873485
12391	CNNM1	-0.00235918	0.95658427
12392	PCDHB2	-0.068739188	0.11226326
12393	C5	-0.034150396	0.430530993
12394	LBHD1	0.525518827	2.56E-39
12395	GPR174	0.047941474	0.268323147
12396	FAM222A	-0.024180943	0.576789593
12397	CEP78	-0.176079159	4.22E-05
12398	APOH	0.07625864	0.078017152
12399	PCDHB12	-0.218311065	3.41E-07
12400	TYRO3	0.172465149	6.07E-05
12401	SUSD4	-0.11953154	0.005636047
12402	ELOVL2	0.107074176	0.013213317
12403	AC105009.1	0.182465813	2.17E-05
12404	ASAH2B	-0.299757888	1.43E-12
12405	KCNC4	0.001919375	0.964672068
12406	BAALC	-0.175192953	4.62E-05
12407	FBXO41	0.1921883	7.58E-06
12408	FCRL6	0.177955398	3.48E-05
12409	GINS3	0.071529367	0.098386477
12410	ZNF717	-0.179243754	3.05E-05

12411	POF1B	-0.068441961	0.113827983
12412	LRP5L	0.387409575	1.33E-20
12413	ZNF404	0.074301954	0.085985869
12414	TREH	0.014932227	0.730399021
12415	BRICD5	0.570738707	1.45E-47
12416	HS3ST3B1	-0.021542176	0.619073236
12417	LACE1	-0.531318551	2.63E-40
12418	CD28	0.006895934	0.873564701
12419	MFAP2	0.185336178	1.60E-05
12420	SMYD3	0.106336923	0.013862457
12421	SLC22A15	-0.123606047	0.004192314
12422	BMX	-0.2364472	3.11E-08
12423	TCF7	0.195277615	5.36E-06
12424	HIST1H3D	0.253360707	2.78E-09
12425	ISM1	0.117919252	0.006321276
12426	FOXP3	0.360803282	6.82E-18
12427	KIAA1456	-0.233470144	4.68E-08
12428	WDHD1	-0.035846761	0.407973761
12429	LPPR5	0.05016978	0.246682167
12430	CYSRT1	0.330326519	4.37E-15
12431	NOS2	-0.077725351	0.072445896
12432	ZNF546	-0.034202325	0.429829937
12433	KIF7	0.31201719	1.52E-13
12434	CCNK	-0.322918986	1.89E-14
12435	DUSP9	0.042729896	0.323895537
12436	CHAF1B	0.106216766	0.013970873
12437	PPP2R3B	0.609072803	1.25E-55
12438	S100B	0.189775927	9.89E-06
12439	CLTCL1	0.095847134	0.026631895
12440	AJAP1	-0.180221866	2.75E-05
12441	POMK	-0.357166562	1.53E-17
12442	CCL15-CCL14	0.230139527	7.32E-08
12443	ALOX15B	0.114279162	0.008150404
12444	RHOH	0.210710072	8.76E-07
12445	CCZ1B	-0.03604113	0.405434777
12446	EBF3	-0.105051936	0.015061182
12447	RSPH9	0.14772918	0.000608451
12448	ITGA10	0.160359641	0.000195637
12449	ZNF81	-0.332582818	2.77E-15
12450	PLCH2	0.161482598	0.000176122
12451	BDH1	-0.160897504	0.00018605
12452	B4GALNT1	-0.075306024	0.081818466
12453	CD40LG	0.130490972	0.002493364
12454	PCDHGA9	-0.174480122	4.96E-05
12455	FAM189A1	0.042329847	0.328450253
12456	B3GNT7	0.131642712	0.002280267
12457	ZNF788	-0.325510845	1.14E-14
12458	ZNF549	-0.298072825	1.94E-12
12459	STXBP6	-0.07177468	0.097234832
12460	PRRT2	0.425136436	6.81E-25
12461	CD244	0.256547923	1.73E-09
12462	FZD3	-0.289085474	9.30E-12
12463	NHLRC4	-0.034414792	0.426968467
12464	FABP1	0.044261173	0.30684234
12465	SYT17	-0.096010452	0.026373582
12466	SEMA3D	-0.121822132	0.004777287
12467	ZNF585A	-0.330199688	4.48E-15
12468	C14orf37	-0.382423223	4.48E-20

12469	CCDC163P	0.332054414	3.09E-15
12470	PDE1C	-0.33936725	6.92E-16
12471	CDC6	0.303295326	7.59E-13
12472	VTCN1	-0.169381979	8.25E-05
12473	TRDMT1	-0.408293968	6.55E-23
12474	XRCC3	0.469220171	1.21E-30
12475	GSTO2	-0.075287793	0.08189265
12476	ZNF765	-0.392968293	3.36E-21
12477	DCAF12L1	-0.130220999	0.002545875
12478	ZNF763	-0.048395651	0.263809972
12479	KIF17	0.180453544	2.69E-05
12480	MAP2K6	-0.23830401	2.41E-08
12481	KLRG1	0.088742636	0.040182122
12482	FCRLB	0.002057712	0.962127692
12483	NCAM1	0.025128807	0.561939504
12484	SYDE2	-0.393537261	2.91E-21
12485	IPCEF1	-0.214153883	5.74E-07
12486	GRIA4	-0.016660765	0.700615348
12487	KCNA5	0.031466215	0.467659058
12488	CACNA1D	-0.200350063	3.00E-06
12489	IL21R	0.194032433	6.17E-06
12490	WTIP	0.158697638	0.00022827
12491	ZNF341	0.562198113	6.58E-46
12492	YJEFN3	0.504367485	7.21E-36
12493	ST6GALNAC2	0.103860421	0.016253101
12494	TRPC4	-0.115443468	0.007519609
12495	GARNL3	-0.266372331	3.84E-10
12496	TIGIT	0.252010079	3.40E-09
12497	CLDN16	-0.133760554	0.001931301
12498	DGCR6	0.226542275	1.18E-07
12499	NPIPB15	0.387555896	1.28E-20
12500	AVPR2	0.070545695	0.103113905
12501	PLEKHM3	-0.406175588	1.14E-22
12502	ARC	0.027936586	0.519063556
12503	SLC24A3	-0.061938366	0.152527237
12504	LILRB3	0.396247719	1.47E-21
12505	SMLR1	0.010472455	0.809036918
12506	AC004381.6	0.151804805	0.000425914
12507	FCHO1	0.366209191	2.01E-18
12508	HPGDS	-0.15615216	0.000288262
12509	RP11-295P9.3	0.048108833	0.266653981
12510	CASR	-0.158265239	0.000237559
12511	ZNF280C	-0.013309968	0.758726169
12512	PIGZ	0.260804326	9.09E-10
12513	BHLHA15	0.100085852	0.020591371
12514	ZNF396	-0.30683584	3.98E-13
12515	EFCAB11	-0.247586471	6.48E-09
12516	ROBO3	0.376476858	1.85E-19
12517	COMP	0.09807666	0.023287451
12518	THEMIS	0.015857251	0.714406555
12519	IGSF21	0.018169286	0.674990714
12520	CYP51A1	-0.441140804	6.95E-27
12521	HSH2D	0.480351081	3.11E-32
12522	LILRB5	-0.003527592	0.935121579
12523	WT1	0.050356368	0.244927033
12524	PCDHB3	-0.109398701	0.011338662
12525	ITGBL1	-0.0394608	0.362321218
12526	PRPF40B	0.220414185	2.61E-07

12527	AC024060.1	0.048955474	0.2583192
12528	ATXN3	-0.033515284	0.439158537
12529	TNFRSF18	0.525498599	2.58E-39
12530	ARID3B	0.288324075	1.06E-11
12531	TRIM54	0.16871037	8.81E-05
12532	MFI2	0.29563435	2.98E-12
12533	ZNF418	-0.034803261	0.421765367
12534	TRPA1	-0.107277382	0.013039175
12535	ZFP28	-0.277564391	6.41E-11
12536	CRTAM	0.139690048	0.001197903
12537	GDF7	-0.188959862	1.08E-05
12538	KIAA1841	0.209050956	1.07E-06
12539	ZNF555	-0.422051901	1.60E-24
12540	TEKT2	0.108741717	0.011843029
12541	ARHGEF39	0.512898146	3.13E-37
12542	ZNF683	0.361449361	5.90E-18
12543	IKZF2	-0.172237548	6.21E-05
12544	COLEC12	-0.14563769	0.000728131
12545	BCL11B	0.120858449	0.005123054
12546	NAALADL1	0.245476979	8.77E-09
12547	ZNF423	-0.112063019	0.009482837
12548	LIPH	-0.151751637	0.000427925
12549	WFDC1	0.08823496	0.041341623
12550	CRYBB3	0.261927104	7.65E-10
12551	GALNT16	-0.137278276	0.001458028
12552	SCAI	-0.156196804	0.000287094
12553	MEX3A	0.183484904	1.95E-05
12554	TBX21	0.333664564	2.23E-15
12555	LDLRAD2	0.032352173	0.455212585
12556	CRISPLD1	-0.075506896	0.081004684
12557	CEP41	-0.09774885	0.023755158
12558	GEN1	0.189093621	1.07E-05
12559	AIM2	0.260573108	9.42E-10
12560	HIVEP3	0.13439881	0.001836156
12561	HAGHL	0.235507082	3.54E-08
12562	ANKRD2	0.11468866	0.007923356
12563	HMMR	0.2572645	1.55E-09
12564	TRAT1	0.113055597	0.0088638
12565	LGI2	-0.175614015	4.42E-05
12566	KIAA0391	-0.222452381	2.01E-07
12567	CFAP69	0.093668701	0.030291295
12568	SAA2	0.12145154	0.004907681
12569	ICAM3	0.18167794	2.36E-05
12570	NACAD	0.048341203	0.264348248
12571	SEC31B	0.447246141	1.13E-27
12572	C17orf96	0.401440827	3.91E-22
12573	PCDHGB2	-0.173754156	5.34E-05
12574	TYW5	-0.044505579	0.304176405
12575	SLC5A4	-0.047287637	0.274912761
12576	MS4A14	0.341198735	4.72E-16
12577	LRRC56	0.349630152	7.92E-17
12578	PACRG	-0.292301154	5.34E-12
12579	CENPL	0.081577509	0.059345691
12580	KIF27	-0.256440029	1.76E-09
12581	ALG10	-0.48046005	3.00E-32
12582	PI3	0.286831342	1.37E-11
12583	CDK5R1	0.232015782	5.69E-08
12584	CBWD1	-0.11875934	0.005955428

12585	UBASH3A	0.317206805	5.70E-14
12586	CCDC122	-0.192633426	7.21E-06
12587	TESPA1	0.154383184	0.000338306
12588	TMEM158	0.275755619	8.61E-11
12589	ZNF668	0.384478384	2.72E-20
12590	CYP4F3	0.086955859	0.044388888
12591	FBXL18	-0.116247534	0.007109842
12592	RTN4R	0.359202536	9.75E-18
12593	EMR1	0.097092154	0.024716597
12594	ZNF572	-0.224093943	1.62E-07
12595	PPP1R32	0.451573566	3.06E-28
12596	FAM229A	0.481141495	2.39E-32
12597	KLHL25	0.197762045	4.04E-06
12598	CCR4	0.009965142	0.818122085
12599	TSNAXIP1	0.405445998	1.38E-22
12600	SLC26A7	-0.22886668	8.67E-08
12601	SCNN1D	0.585818416	1.31E-50
12602	DIO1	-0.019570263	0.651524038
12603	ANK1	-0.017773324	0.681681956
12604	ZNF620	-0.311334532	1.73E-13
12605	BUB1	0.26280021	6.69E-10
12606	ZBED9	-0.390602556	6.05E-21
12607	CD200R1	-0.004853486	0.910823763
12608	TMEM121	0.20067138	2.89E-06
12609	MTHFD2L	0.045669899	0.291687327
12610	HOXA11	0.084406632	0.051027573
12611	AATK	0.178118321	3.42E-05
12612	BRINP1	0.031325203	0.469657277
12613	PTCHD4	-0.391688216	4.62E-21
12614	PKP1	0.0388594	0.36968846
12615	FOXI1	-0.215516797	4.84E-07
12616	MYLK4	-0.118545602	0.006046658
12617	RASGRP4	0.080359506	0.063257396
12618	TRIM59	0.140104308	0.001157784
12619	VSIG1	0.078365967	0.070116621
12620	PTGFR	-0.187737785	1.24E-05
12621	LRRC70	-0.238475281	2.35E-08
12622	ZNF571	-0.126065728	0.003491909
12623	KCNJ10	0.01840681	0.67098909
12624	CEP76	-0.109742774	0.011082182
12625	PLAC8	0.129723592	0.002645269
12626	LILRA2	-0.018111283	0.675969314
12627	SMN2	0.124341997	0.003970475
12628	CDCA7	0.248305519	5.84E-09
12629	FBXO10	0.240453719	1.79E-08
12630	TCEANC	-0.053306966	0.218328424
12631	OR2A4	0.215161507	5.06E-07
12632	SIRPB1	-0.021228464	0.624188721
12633	SOBP	-0.059326787	0.170615096
12634	BRCA1	0.001114747	0.979477582
12635	SMIM8	-0.170223715	7.59E-05
12636	PDZD4	0.243820264	1.11E-08
12637	YPEL1	-0.177929336	3.49E-05
12638	WDR17	-0.175085036	4.67E-05
12639	PDE6G	0.24877479	5.45E-09
12640	SYTL1	0.426020272	5.32E-25
12641	LILRA1	-0.026535688	0.540243708
12642	LIPG	-0.051747587	0.232115828

12643	NCAPH	0.398613427	8.06E-22
12644	TREX2	0.145563422	0.000732757
12645	CRLF1	0.058878567	0.173874302
12646	PADI1	0.014059945	0.745587639
12647	IL1RN	0.066000274	0.127338214
12648	APLP1	0.229368481	8.11E-08
12649	C1orf116	-0.180918927	2.56E-05
12650	ADRB2	-0.263938799	5.61E-10
12651	ZNF286A	-0.195391437	5.29E-06
12652	PRND	-0.014063191	0.745530927
12653	GRAMD2	0.141106219	0.001065818
12654	ERCC6	-0.27271735	1.41E-10
12655	TTC4	-0.079825456	0.065038321
12656	ADTRP	-0.090585946	0.03620066
12657	FAM167A	-0.024107754	0.577943869
12658	RPS6KA6	-0.331958332	3.15E-15
12659	CHIT1	0.007115625	0.869572122
12660	ZFP37	-0.230923211	6.59E-08
12661	MXD3	0.705969124	6.85E-82
12662	C15orf41	-0.039104633	0.366673188
12663	FHDC1	-0.174737868	4.83E-05
12664	SLC4A11	0.076847283	0.075740789
12665	C10orf12	-0.397871277	9.74E-22
12666	PBX4	0.530387147	3.80E-40
12667	MDH1B	0.078392965	0.07001982
12668	SHF	-0.001028135	0.981071802
12669	CYP17A1	-0.110895327	0.010260081
12670	ULBP3	-0.327949545	7.02E-15
12671	CEACAM19	0.484763115	7.03E-33
12672	IRX6	0.197117509	4.35E-06
12673	SLC35E4	0.397433445	1.09E-21
12674	CCNF	0.491968909	5.90E-34
12675	ZNF565	0.128359784	0.002936206
12676	ATP6V1E2	0.042528077	0.326188173
12677	RNF43	-0.297950052	1.98E-12
12678	PINLYP	-0.006789578	0.875498728
12679	MAP6	-0.051030566	0.238658108
12680	EFNB3	-0.054964552	0.204327006
12681	C12orf60	0.052201218	0.22804293
12682	AKAP6	-0.458714448	3.39E-29
12683	BEST4	0.097463035	0.024169556
12684	ZBTB8A	-0.013505922	0.755286445
12685	TNFSF15	-0.215040964	5.14E-07
12686	BUB1B	0.298642704	1.75E-12
12687	CDC45	0.498260198	6.46E-35
12688	DNAJC28	-0.234024788	4.34E-08
12689	APOB	-0.153090832	0.000379869
12690	C11orf45	0.028696945	0.507749403
12691	PIWIL4	0.202223429	2.41E-06
12692	ILDR2	-0.184300632	1.79E-05
12693	TSSK4	-0.017642765	0.683893742
12694	AVIL	0.30285913	8.21E-13
12695	UHRF1	0.391005546	5.48E-21
12696	IL24	0.194818433	5.65E-06
12697	MYOC	-0.012263098	0.77718183
12698	FAM71E1	0.130301061	0.002530198
12699	STAG3L5P-PVRIG2P-PI	0.485853179	4.85E-33
12700	ZNF296	0.56569849	1.40E-46

12701	UQCRHL		0.228933968	8.60E-08
12702	ZNF682		0.3064379	4.28E-13
12703	CEP152		0.003733568	0.931342418
12704	FLT3LG		0.634180619	1.55E-61
12705	ZNF283		-0.298599234	1.76E-12
12706	CIB4		0.162405188	0.00016147
12707	PCDHGB5		-0.198426312	3.75E-06
12708	BEST1		0.10908362	0.011578127
12709	MEOX2		0.009203909	0.831799986
12710	GRK4		0.171664921	6.58E-05
12711	ZNF425		-0.078585563	0.069332417
12712	HOXD3		-0.083025705	0.054957382
12713	GAS2		-0.223241283	1.81E-07
12714	OPRL1		0.212253913	7.25E-07
12715	PTX3		0.146124203	0.00069849
12716	HELLS		0.191552677	8.13E-06
12717	MCCD1		-0.153059694	0.000380927
12718	ARMC2		-0.032120231	0.458452954
12719	CAPS2		-0.211483757	7.97E-07
12720	LY6G5C		0.429221921	2.16E-25
12721	ZNF662		-0.059334384	0.170560249
12722	ZMYND10		0.15582274	0.000297023
12723	LRCH2		-0.232728597	5.17E-08
12724	RBM24		-0.092775747	0.031911357
12725	C8orf48		0.093869419	0.029936999
12726	KNDC1		0.027043379	0.532518388
12727	ACTRT3		-0.061871512	0.152971362
12728	METTL12		0.272476518	1.46E-10
12729	GNAL		0.050092904	0.247407852
12730	SLC38A4		-0.219581313	2.90E-07
12731	RNF32		0.129896786	0.002610266
12732	SPAG17		0.115744292	0.007363894
12733	CHN2		-0.129285465	0.002735735
12734	SLC34A3		0.153074969	0.000380408
12735	ZNF547		0.179540686	2.95E-05
12736	PCP2		0.289647956	8.44E-12
12737	KCNF1		0.161726273	0.000172135
12738	FAM124B		-0.167732308	9.69E-05
12739	SLC6A16		-0.015679184	0.717475663
12740	OVOL1		-0.197970006	3.95E-06
12741	CFP		0.315656478	7.66E-14
12742	ENPP7		-0.003615821	0.933502616
12743	CACNA2D4		0.202219051	2.41E-06
12744	KIF23		0.367779763	1.40E-18
12745	FAP		0.053415529	0.217390885
12746	KIAA1328		-0.316082098	7.06E-14
12747	ZNF491		0.021323011	0.622645092
12748	RALGPS1		-0.217720383	3.67E-07
12749	CCDC113		-0.135428905	0.00169162
12750	CLMP		0.191892091	7.83E-06
12751	NPM2		0.148787656	0.000555098
12752	SPNS1		0.2758927	8.42E-11
12753	C11orf70		0.02180696	0.614769869
12754		3-Mar	0.100746113	0.019766725
12755	HJURP		0.529482247	5.43E-40
12756	CPN2		-0.029319582	0.498581605
12757	ZNF582		-0.228650491	8.93E-08
12758	TMCC2		0.081742068	0.058832882

12759	FXYD3	0.002232456	0.958914242
12760	REL	-0.33237763	2.89E-15
12761	ATP6V1G3	-0.232966005	5.01E-08
12762	MYCL	8.00E-05	0.998526263
12763	ATP6AP1L	0.192439595	7.37E-06
12764	SHPRH	-0.469153524	1.24E-30
12765	MORN1	0.226797829	1.14E-07
12766	PDPN	0.064419017	0.136730588
12767	ZNF501	-0.376516936	1.84E-19
12768	OR2T10	-0.108364525	0.012141478
12769	MAP6D1	0.448683537	7.35E-28
12770	ARL11	0.084617531	0.050448574
12771	TXK	0.044020163	0.309486311
12772	AC091180.1	0.246195879	7.92E-09
12773	TMEM163	0.026554877	0.5399507
12774	FAM81A	-0.164879833	0.000127617
12775	HAUS8	0.692781494	1.06E-77
12776	MUC13	0.067474371	0.119040598
12777	ZNF860	-0.193602351	6.47E-06
12778	CTXN3	-0.017794482	0.681323782
12779	DNAH10OS	-0.121354567	0.004942328
12780	NCKAP5	-0.396286226	1.46E-21
12781	KLHDC7B	0.214979621	5.18E-07
12782	SMIM5	-0.072011181	0.096134754
12783	DLK2	0.378805028	1.07E-19
12784	CCDC117	-0.49086251	8.66E-34
12785	XCL1	0.301478175	1.05E-12
12786	OIP5	0.285354021	1.75E-11
12787	CCL17	0.319836936	3.44E-14
12788	BCL2L10	-0.143395144	0.00088043
12789	IGLON5	-0.037160365	0.390998687
12790	HP	0.030521739	0.481132171
12791	RSPO3	-0.20474846	1.79E-06
12792	C22orf23	0.045582945	0.292607995
12793	DLGAP5	0.284695273	1.96E-11
12794	C8orf46	0.074196073	0.08643512
12795	SLC25A48	0.029030721	0.502823881
12796	LY6G5B	0.352219412	4.52E-17
12797	NEURL2	0.200197296	3.06E-06
12798	GPR89A	0.24858795	5.60E-09
12799	PRRX2	0.292125875	5.50E-12
12800	DNAJB13	0.041464201	0.338447026
12801	TENM4	-0.143849382	0.000847387
12802	CYB5RL	-0.168938982	8.61E-05
12803	ZNF26	0.383745925	3.25E-20
12804	ZNF93	0.016064971	0.710832194
12805	CLSTN2	-0.136470025	0.001556208
12806	ESPL1	0.525648542	2.44E-39
12807	TSSK6	0.333179879	2.46E-15
12808	WNT5B	-0.000468356	0.991376815
12809	SLC35F1	-0.166566727	0.000108496
12810	SLC30A2	-0.03612373	0.404358654
12811	KLHL4	-0.070357119	0.104040424
12812	TMEM71	0.14159858	0.001023138
12813	TIGD7	-0.049013241	0.25775715
12814	OPCML	-0.088420089	0.040915567
12815	IFI30	0.52209826	9.62E-39
12816	NCAPG	0.317523543	5.37E-14

12817	RAD54L	0.297097681	2.30E-12
12818	SOWAHA	-0.195724815	5.10E-06
12819	RDH12	-0.014760951	0.733373274
12820	SCUBE3	-0.061510372	0.15538746
12821	SLC16A11	-0.110923837	0.01024045
12822	FST	0.033416272	0.440512413
12823	AK9	-0.267630683	3.16E-10
12824	C5AR2	-0.055079064	0.203384364
12825	NOD2	0.247102242	6.95E-09
12826	COX6A2	0.253397916	2.77E-09
12827	MESP1	0.303943864	6.75E-13
12828	CNIH3	0.032249491	0.456645523
12829	LRRN4CL	0.055484607	0.200071404
12830	FOXD2	0.198911276	3.54E-06
12831	SSC4D	0.280711143	3.82E-11
12832	MAMDC2	-0.188895363	1.09E-05
12833	RANBP3L	-0.243738145	1.12E-08
12834	FOXP2	-0.013495996	0.755460577
12835	ULK4	-0.041334656	0.339959643
12836	ZBTB7C	-0.013201401	0.76063398
12837	DBF4B	0.488341131	2.07E-33
12838	ZDHHC11B	0.184339184	1.78E-05
12839	KLHL3	-0.199761517	3.21E-06
12840	ABCA12	-0.070345101	0.104099698
12841	GFI1	0.305116473	5.45E-13
12842	BORA	0.117901609	0.006329171
12843	EHF	-0.241535308	1.53E-08
12844	NLGN4Y	-0.152322877	0.000406773
12845	TMEM156	0.219668635	2.87E-07
12846	DLEU1	-0.234934936	3.83E-08
12847	RHBDL2	0.226235153	1.23E-07
12848	MATN3	-0.044045927	0.309202955
12849	RAD51	0.315719916	7.57E-14
12850	NUGGC	-0.07575059	0.08002623
12851	KCNK9	0.08721119	0.043765941
12852	TCTE3	0.339970424	6.10E-16
12853	RHEBL1	0.607557973	2.74E-55
12854	WNT3	0.294736347	3.49E-12
12855	ARL6IP4	0.628342652	4.09E-60
12856	LCN2	0.05719534	0.186528296
12857	NOG	0.10938757	0.011347046
12858	SH3GL2	-0.094155422	0.029438301
12859	NEK5	0.020309633	0.639274967
12860	ZFPM2	-0.053990965	0.212469652
12861	LSMEM1	0.361431796	5.93E-18
12862	APBA1	-0.133383523	0.001989606
12863	GPR89B	0.256026947	1.87E-09
12864	TSHZ2	-0.097673353	0.023864019
12865	ZNF90	0.203312086	2.12E-06
12866	PMEL	-0.007377091	0.864824675
12867	HIST1H2AI	-0.034021641	0.43227206
12868	PLK4	0.158506366	0.000232336
12869	VAT1L	-0.157825912	0.000247358
12870	ZKSCAN7	-0.054818717	0.20553208
12871	NPHS2	-0.055514686	0.199827263
12872	CXXC4	-0.297988167	1.97E-12
12873	REEP1	-0.086373974	0.04583647
12874	KCNN4	0.528926123	6.76E-40

12875	KAZALD1	0.209671942	9.94E-07
12876	FOXD1	0.082281784	0.057176672
12877	GLOD5	-0.075150835	0.082451695
12878	PRRT1	0.393819156	2.71E-21
12879	C14orf80	0.584148627	2.90E-50
12880	DNA2	0.485469183	5.53E-33
12881	SCN4A	-0.068403772	0.114030258
12882	SLC10A6	-0.208678875	1.12E-06
12883	KIF21B	0.203002912	2.20E-06
12884	OLIG1	0.116790281	0.006844667
12885	GTSE1	0.456777611	6.19E-29
12886	GET4	0.267415337	3.26E-10
12887	CATSPER3	0.240683854	1.73E-08
12888	KCND3	-0.131315207	0.002339104
12889	VSIG10L	0.290571589	7.20E-12
12890	KIF5C	-0.12469812	0.003866993
12891	DNM3	-0.208096937	1.20E-06
12892	ADAMTS12	-0.028077115	0.516962738
12893	TTLL6	0.239036761	2.18E-08
12894	TSPAN11	-0.095440644	0.027284301
12895	APCDD1L	0.198178572	3.85E-06
12896	KLK1	-0.184772823	1.70E-05
12897	ADH1C	-0.119312955	0.005724852
12898	ADAT3	0.490958844	8.38E-34
12899	SYCE3	0.21103092	8.42E-07
12900	GLYATL2	0.040097868	0.354617828
12901	ZMAT4	-0.037256459	0.389773929
12902	ADAT2	0.282178606	2.99E-11
12903	IL23A	0.44318286	3.80E-27
12904	CCDC151	-0.005119147	0.905964613
12905	AKR1B10	0.061936424	0.152540125
12906	ESRP1	-0.234120683	4.28E-08
12907	ABCA8	-0.238834563	2.24E-08
12908	RP11-477N12.3	-0.133063294	0.002040386
12909	NHSL2	-0.322154101	2.20E-14
12910	KCNQ3	-0.111462019	0.009876081
12911	SP140	0.292389877	5.26E-12
12912	SLC4A3	0.096304131	0.025914512
12913	BTBD11	0.077214353	0.074348843
12914	ACTC1	-0.044897691	0.29993146
12915	LAX1	0.113690435	0.008487
12916	SHISA2	-0.138649095	0.001304432
12917	FAM196B	-0.321011745	2.74E-14
12918	SPIRE2	0.226472747	1.19E-07
12919	CCDC85A	-0.261196225	8.56E-10
12920	ZFPM1	0.443580066	3.38E-27
12921	SARNP	0.321245562	2.62E-14
12922	ANKHD1	0.067641772	0.11812566
12923	ARIH2OS	0.264787755	4.92E-10
12924	MPP3	0.490350799	1.03E-33
12925	SGOL2	-0.011750648	0.786262643
12926	ENOX1	0.054363285	0.209328567
12927	COL25A1	-0.179431173	2.99E-05
12928	CHL1	-0.191616803	8.08E-06
12929	FA2H	-0.040137268	0.354144813
12930	TJP3	-0.032392407	0.454651796
12931	ALDH1L2	-0.019905922	0.64595132
12932	LY6H	0.137356462	0.001448839

12933	AL138706.2	0.034470017	0.426226521
12934	BVES	-0.149255341	0.000532937
12935	IQCJ-SCHIP1	0.033604406	0.437941945
12936	HIST1H4E	0.109232075	0.011464747
12937	ADCY1	-0.327488547	7.70E-15
12938	SLC35G1	-0.224066129	1.63E-07
12939	PEG3	-0.385872801	1.94E-20
12940	GPR135	-0.020899856	0.629566512
12941	MTL5	-0.120407498	0.00529247
12942	FAM153C	0.130959093	0.002404651
12943	C8G	0.320644453	2.94E-14
12944	SYNE3	-0.103121713	0.01703288
12945	IPPK	-0.247151981	6.90E-09
12946	WDR73	0.155597379	0.000303159
12947	DIAPH3	-0.129882316	0.002613174
12948	CD300LB	0.142843744	0.000922142
12949	ANKS1B	-0.114005117	0.008305574
12950	SH3RF2	0.078155807	0.070873913
12951	HIF3A	0.184941778	1.67E-05
12952	ZNF235	-0.135767912	0.001646384
12953	DFNB59	0.377167877	1.57E-19
12954	IQUB	-0.286604967	1.42E-11
12955	SPAG8	0.313353504	1.18E-13
12956	ASB2	0.323011819	1.86E-14
12957	MACROD2	-0.082212734	0.057386382
12958	CLDND2	0.598378831	2.87E-53
12959	POLE2	0.239239266	2.12E-08
12960	MCOLN2	0.14308249	0.000903862
12961	ZNF771	0.300908817	1.17E-12
12962	FANCD2	0.266624269	3.69E-10
12963	ZNF460	-0.235402833	3.59E-08
12964	GPR173	0.3339257	2.11E-15
12965	MC1R	0.450131993	4.74E-28
12966	KIF24	0.02436275	0.57392699
12967	HDX	-0.344640541	2.29E-16
12968	C17orf53	0.496980821	1.02E-34
12969	SKA3	0.365574648	2.32E-18
12970	LTB4R2	0.290919495	6.78E-12
12971	FSD1L	-0.03953071	0.361470809
12972	HPCA	0.300952307	1.16E-12
12973	NTS	0.012262255	0.777196744
12974	SCGB3A1	0.230861239	6.65E-08
12975	AQP4	-0.28980088	8.22E-12
12976	B3GNTL1	0.599457526	1.67E-53
12977	PAPPA	-0.110428196	0.010586521
12978	TBC1D30	-0.109123955	0.011547225
12979	PCLO	-0.249093309	5.21E-09
12980	PLCXD3	-0.179523319	2.96E-05
12981	ABCB4	-0.092294516	0.032814759
12982	ANKRD24	0.471894078	5.08E-31
12983	TDRD10	0.255402664	2.06E-09
12984	ITPKA	0.348080653	1.10E-16
12985	BMPR1B	-0.153692824	0.00035995
12986	CCDC183	0.294071953	3.92E-12
12987	FAM78B	0.209840874	9.74E-07
12988	CDH3	-0.010846668	0.802351705
12989	ZNF658	-0.358034468	1.26E-17
12990	SLC22A8	-0.042174938	0.330225025

12991	TBC1D31	0.035285551	0.415357509
12992	WNT9A	0.25710132	1.59E-09
12993	GAFA3	-0.457350037	5.18E-29
12994	C1orf112	-0.014456893	0.73866315
12995	NEK2	0.312319167	1.44E-13
12996	TCEAL2	-0.16281258	0.000155371
12997	NHEJ1	-0.076816281	0.075859312
12998	C15orf62	0.203977733	1.96E-06
12999	MEX3B	0.153295745	0.000372976
13000	UNC5C	-0.224284465	1.58E-07
13001	CA3	0.084708112	0.050201586
13002	ZNF284	0.013972125	0.74712239
13003	LRRC66	0.37086611	6.89E-19
13004	PTPN20A	-0.120608408	0.005216377
13005	SLC16A10	-0.320438353	3.06E-14
13006	BBS1	0.096026331	0.026348582
13007	FAM81B	0.024143903	0.577373622
13008	CTLA4	0.345469676	1.92E-16
13009	STAC	-0.101265579	0.019138286
13010	MPPED2	-0.29616115	2.72E-12
13011	CBFA2T3	0.125081089	0.00375844
13012	KLHDC8A	0.042113313	0.330932771
13013	CHADL	0.221312589	2.33E-07
13014	HIST4H4	0.010277251	0.812529745
13015	RAB9B	-0.163378699	0.000147254
13016	FDXACB1	0.07510177	0.082652721
13017	FAM27C	0.048936051	0.258508364
13018	C20orf202	-0.064761383	0.134653068
13019	CARD9	0.442774498	4.29E-27
13020	INHA	0.066187123	0.126262263
13021	DMRT2	-0.21522244	5.02E-07
13022	TLR6	0.038851745	0.369782836
13023	CD22	0.046878598	0.279090779
13024	NRXN3	-0.271678443	1.66E-10
13025	STX1A	0.55375532	2.56E-44
13026	CCNE1	0.353846951	3.17E-17
13027	C1QTNF7	-0.182338159	2.20E-05
13028	C2CD4B	0.20816521	1.19E-06
13029	MLPH	0.195248053	5.38E-06
13030	FAM101A	0.137697882	0.001409333
13031	STRIP2	0.136307546	0.001576663
13032	ZNF311	0.121042777	0.005055222
13033	ATP8B4	-0.299631472	1.47E-12
13034	UBE3D	-0.127594447	0.003111948
13035	C16orf46	-0.090709369	0.035946491
13036	EPPK1	0.018549437	0.668590683
13037	USP49	0.083742758	0.0528865
13038	ANKAR	-0.041433586	0.338804102
13039	CALB1	-0.138353918	0.001336189
13040	EPS8L3	0.178358689	3.34E-05
13041	AL158801.1	-0.045689945	0.291475354
13042	KIAA0226L	0.047613895	0.271610966
13043	SLMO1	0.502769529	1.29E-35
13044	NPIPA5	0.385706112	2.02E-20
13045	KCNJ11	-0.053775479	0.214303048
13046	F11	-0.131251586	0.002350693
13047	ZNF773	-0.198127379	3.88E-06
13048	F2	0.090567063	0.036239681

13049	DIO2	0.020040489	0.643722734
13050	PSD3	-0.189264764	1.05E-05
13051	FGF12	-0.26686463	3.56E-10
13052	VGLL3	-0.053432782	0.217242155
13053	MATR3	-0.07330624	0.090285817
13054	BIK	0.099369101	0.02152028
13055	C3orf52	-0.024579278	0.570526472
13056	THSD7B	-0.222798808	1.92E-07
13057	WDPCP	-0.217061356	3.99E-07
13058	TBC1D32	-0.287532614	1.21E-11
13059	ACPP	-0.164680594	0.000130074
13060	ZNF575	0.390598417	6.06E-21
13061	CHAD	0.11966803	0.005581225
13062	FERMT1	-0.082715702	0.055873334
13063	SLC47A2	-0.011588184	0.789147705
13064	ADAMTS16	-0.0106109	0.806561997
13065	LCA5L	-0.057252325	0.186089091
13066	CFAP70	0.100013055	0.020684099
13067	LRP2BP	-0.064188705	0.138141964
13068	WNT2B	-0.002558181	0.95292615
13069	CTSE	0.082470961	0.056605383
13070	AC253572.1	-0.060662918	0.161170388
13071	LETM2	-0.015558445	0.719559266
13072	AFM	-0.068732718	0.112297143
13073	FCGR3B	-0.016059022	0.710934469
13074	MND1	0.243724893	1.13E-08
13075	HEPACAM2	-0.233712659	4.52E-08
13076	CPNE7	0.48732627	2.93E-33
13077	NCR3	0.331375037	3.54E-15
13078	VPREB3	0.308212472	3.09E-13
13079	C2CD4C	0.179460782	2.98E-05
13080	CCDC150	0.236844332	2.95E-08
13081	TMC7	-0.019783102	0.647988144
13082	ECE2	0.473055206	3.48E-31
13083	TROAP	0.634726458	1.14E-61
13084	C1orf204	0.098680793	0.022446356
13085	PRDM15	0.314800597	9.01E-14
13086	ZNF583	-0.344265731	2.48E-16
13087	ZSCAN5A	-0.001974617	0.963655984
13088	AQP6	-0.166509018	0.000109103
13089	SNX22	0.413221308	1.77E-23
13090	PRRT3	0.077845033	0.072005998
13091	SLC22A13	-0.001934837	0.964387664
13092	CHRNE	0.24358619	1.15E-08
13093	EP400NL	0.424195386	8.85E-25
13094	NIPAL1	-0.40960007	4.64E-23
13095	PFN4	0.367074636	1.65E-18
13096	CCDC30	-0.058618851	0.175783912
13097	ADAMTS3	-0.050687299	0.24183567
13098	FOXO6	0.234833207	3.88E-08
13099	SDR42E1	-0.32486492	1.29E-14
13100	SLC35E2	-0.011015092	0.799347533
13101	TREM1	0.142311913	0.000964096
13102	CPT1C	0.286952187	1.34E-11
13103	EPHB2	0.167875522	9.56E-05
13104	ANO7	0.074842379	0.083722068
13105	SAPCD2	0.420922861	2.19E-24
13106	CAND2	-0.100538367	0.020023037

13107	ART4	-0.092658781	0.032128956
13108	TMEM61	-0.250293381	4.37E-09
13109	MSH5	0.3958086	1.64E-21
13110	FANCM	-0.206050876	1.54E-06
13111	GRIN2A	-0.144468998	0.00080416
13112	CMTM1	0.0519248	0.230518647
13113	PTK6	0.300532592	1.25E-12
13114	ORC1	0.278856463	5.18E-11
13115	SRXN1	-0.147899635	0.000599549
13116	FAM64A	0.417788013	5.15E-24
13117	RXFP1	-0.106481405	0.013733075
13118	SPINK5	-0.003123143	0.942546432
13119	TNNC1	0.035480563	0.412782891
13120	CRYBB1	0.156277193	0.000285001
13121	GPBAR1	0.318855913	4.16E-14
13122	CNGA1	-0.272985172	1.35E-10
13123	ZSCAN20	-0.34797388	1.13E-16
13124	CLHC1	0.200435002	2.97E-06
13125	EGFL8	0.395901997	1.61E-21
13126	ZNF812	-0.161814701	0.00017071
13127	C19orf18	0.312918665	1.28E-13
13128	NTF3	0.064757148	0.134678618
13129	CACNB1	0.440487708	8.42E-27
13130	SMKR1	0.25999262	1.03E-09
13131	EPHB1	-0.101774733	0.018539296
13132	CAPN3	0.160746739	0.000188692
13133	KCNN1	0.093368789	0.030827376
13134	ICA1L	0.049252152	0.255441614
13135	HIST2H2AC	0.170370965	7.48E-05
13136	TMEM86B	0.62162827	1.63E-58
13137	HDAC9	-0.241695506	1.50E-08
13138	RAD51B	-0.103123469	0.017030988
13139	MMP25	0.375435288	2.37E-19
13140	PCDHGB6	-0.165842973	0.000116344
13141	WISP2	0.215518319	4.84E-07
13142	RP11-793H13.10	0.510715736	7.05E-37
13143	PCDHGA6	0.02024598	0.640325709
13144	RP11-864I4.1	0.428676441	2.52E-25
13145	SLAMF1	0.255017484	2.18E-09
13146	FUT3	0.012113703	0.779826097
13147	SUSD5	-0.100988306	0.019471524
13148	SLC13A2	-0.054710786	0.206427254
13149	CRIP3	0.252867465	3.00E-09
13150	UPP2	-0.057801444	0.181895831
13151	ANO5	-0.288142857	1.09E-11
13152	NAALADL2	-0.492346494	5.17E-34
13153	L3MBTL1	0.298050736	1.94E-12
13154	SRSF12	-0.044082497	0.308801049
13155	SYT7	-0.059085188	0.172366154
13156	KYNU	-0.00635381	0.883430558
13157	SLC44A5	-0.0052107	0.904290849
13158	HAS3	0.231177896	6.37E-08
13159	CCDC184	0.070012703	0.105749644
13160	HESX1	0.275452768	9.04E-11
13161	CH507-9B2.3	0.059500317	0.169365612
13162	MYRFL	0.149813607	0.000507561
13163	NTN1	-0.150245626	0.000488699
13164	ALKBH6	0.490624551	9.41E-34

13165	CENPK	0.289615876	8.49E-12
13166	STX1B	0.370497033	7.51E-19
13167	CTSG	-0.070381797	0.103918803
13168	RPS6KL1	0.274342687	1.08E-10
13169	CYP27B1	0.155582282	0.000303574
13170	GAN	-0.114113194	0.008244066
13171	ESR1	-0.169500103	8.15E-05
13172	DTNA	-0.19797917	3.94E-06
13173	UBAP1L	0.277459786	6.52E-11
13174	OR2A7	0.081787493	0.058691974
13175	AKAP5	0.094996596	0.02801266
13176	CDCA3	0.596308061	8.02E-53
13177	ANKRD53	0.150230866	0.000489333
13178	GPR20	-0.163007036	0.000152537
13179	CCT6B	-0.051567895	0.233743335
13180	ZNF674	-0.20209018	2.45E-06
13181	ZFP92	-0.175427168	4.51E-05
13182	WFIKKN1	0.277964501	6.00E-11
13183	NR0B2	-0.22012044	2.71E-07
13184	BSND	-0.228679614	8.89E-08
13185	C1QTNF2	0.084657642	0.050339079
13186	ASGR2	0.180682148	2.62E-05
13187	SYN1	0.132293104	0.002167413
13188	ZNF530	0.07495793	0.08324433
13189	PPAPDC1A	-0.062441726	0.149214689
13190	ERICH4	0.013007918	0.76403759
13191	ZFP69B	-0.07136258	0.099175661
13192	GRAP2	0.200509142	2.95E-06
13193	STOX1	-0.087845179	0.042250922
13194	BRSK1	0.462634685	9.90E-30
13195	FREM1	-0.314417068	9.69E-14
13196	CAPN13	0.229478407	8.00E-08
13197	AKR1E2	0.170214627	7.60E-05
13198	KCNH6	0.17156802	6.64E-05
13199	C5orf34	0.104890748	0.015217798
13200	CWH43	-0.22065904	2.53E-07
13201	NUF2	0.451512573	3.12E-28
13202	TTC21A	0.401490927	3.86E-22
13203	TGM1	0.020258645	0.640116587
13204	GLI1	0.211970431	7.51E-07
13205	PKP3	0.129520103	0.002686942
13206	ARHGEF26	-0.141531459	0.001028862
13207	RAG1	-0.439531529	1.11E-26
13208	ANO3	-0.276713505	7.36E-11
13209	FBXO16	0.036945612	0.39374421
13210	HYKK	-0.036782486	0.395837468
13211	FGF7	-0.057241222	0.186174608
13212	ZNF835	-0.174305676	5.05E-05
13213	MUC15	-0.166423694	0.000110006
13214	CFAP44	0.297532995	2.13E-12
13215	SMOC1	-0.135983054	0.001618256
13216	GRIP1	-0.093133563	0.031253499
13217	NKAIN1	0.164705497	0.000129765
13218	TSGA10	-0.202447838	2.35E-06
13219	LPHN3	-0.174416987	4.99E-05
13220	ZNF19	-0.159498685	0.000211953
13221	GP1BA	0.071260897	0.099659255
13222	LYPD1	0.189017202	1.08E-05

13223	TCF15	0.223775294	1.69E-07
13224	IL18RAP	0.291293465	6.36E-12
13225	CD101	0.177371487	3.69E-05
13226	PTPLA	0.133193078	0.002019665
13227	C11orf86	0.185033402	1.65E-05
13228	RP11-195F19.5	0.320006917	3.33E-14
13229	CHGB	-0.006963196	0.872341962
13230	MUM1L1	0.006096203	0.888125047
13231	PLB1	0.13162028	0.002284253
13232	WDR66	0.000162602	0.99700619
13233	PAPPA2	-0.204138697	1.93E-06
13234	STIL	0.203963927	1.97E-06
13235	GPR110	-0.264470075	5.17E-10
13236	ROPN1L	-0.040388635	0.351136415
13237	MS4A4E	0.130987505	0.00239936
13238	RBM34	0.335109616	1.66E-15
13239	C17orf49	0.511532911	5.21E-37
13240	HELB	-0.019221042	0.657342579
13241	CA8	-0.118995821	0.005855934
13242	SP6	-0.017569892	0.685129466
13243	OMD	-0.095060402	0.027906983
13244	SBK1	0.116617412	0.006928149
13245	SIK1	0.043391706	0.316451092
13246	ZNF793	-0.040896139	0.345111939
13247	RNF165	0.023727482	0.583958572
13248	FANCA	0.383069693	3.83E-20
13249	SLC35F3	0.021194746	0.624739606
13250	GLI2	0.159886506	0.000204452
13251	APLF	-0.189582169	1.01E-05
13252	ZRANB3	-0.184388984	1.77E-05
13253	TFAP2A	0.026129325	0.546467173
13254	DNHD1	0.372331496	4.90E-19
13255	MAMSTR	0.121128322	0.005024019
13256	HTR6	0.017110193	0.692943884
13257	TMSB4Y	-0.034284599	0.428720572
13258	VENTX	0.298060183	1.94E-12
13259	ZNF385C	0.033539913	0.438822142
13260	RASSF10	-0.177313154	3.72E-05
13261	KIAA1683	0.152363738	0.000405298
13262	F2RL2	-0.079771866	0.065219284
13263	MEGF11	-0.020486545	0.636358404
13264	KRT86	-0.010214982	0.813644726
13265	CLDN14	-0.007244765	0.867226728
13266	CHRM3	-0.224599432	1.52E-07
13267	P2RX1	0.227457867	1.05E-07
13268	KIF26B	-0.025350404	0.558494524
13269	SPSB3	0.274892556	9.90E-11
13270	IGF2BP1	0.017373888	0.68845729
13271	ZNF660	-0.233178818	4.86E-08
13272	SLC6A20	-0.108385605	0.012124625
13273	RBM20	-0.166135481	0.00011311
13274	TNNI2	0.436056469	3.06E-26
13275	CNTN1	-0.147531543	0.000618925
13276	TF	0.115084308	0.007709371
13277	PLA2G2D	0.187890811	1.22E-05
13278	TAS2R5	0.228938133	8.59E-08
13279	PCDHGA12	-0.084831923	0.049865624
13280	NUPR1L	-0.248654529	5.55E-09

13281	ZNF714	-0.123003881	0.0043821
13282	SGK494	0.333914726	2.12E-15
13283	C10orf126	-0.077850546	0.071985789
13284	TMEM217	0.074422094	0.085478394
13285	CCL22	0.084799614	0.049953113
13286	KLHL14	-0.13251723	0.002129722
13287	IGDCC4	0.12534469	0.003685336
13288	STAG3	0.161024303	0.000183855
13289	IRX1	0.049975948	0.248514736
13290	CCDC18	0.24315705	1.22E-08
13291	INHBE	0.23718899	2.81E-08
13292	ATAD5	0.176597012	4.00E-05
13293	HIST1H2BJ	0.134136646	0.001874705
13294	CCL13	0.074324953	0.085888533
13295	RIMS3	-0.05643932	0.192427663
13296	SYP	0.153754229	0.000357973
13297	COL22A1	0.189323752	1.04E-05
13298	CASZ1	-0.166231876	0.000112063
13299	NRL	0.103952901	0.016157716
13300	NUDT6	-0.110720919	0.0103809
13301	NKX3-1	0.091326542	0.034698184
13302	NXPH3	0.017093119	0.693234766
13303	GOLGA7B	0.304326084	6.29E-13
13304	PATL2	0.327947827	7.03E-15
13305	NAT8L	-0.169875467	7.86E-05
13306	CYP3A7	-0.221629664	2.23E-07
13307	CLGN	-0.066412958	0.124971257
13308	ZNF573	-0.257970884	1.40E-09
13309	C17orf51	-0.093800577	0.030058112
13310	NELL2	0.138583028	0.001311479
13311	MFAP5	0.026046798	0.547735396
13312	NOMO3	-0.334559734	1.86E-15
13313	LRFN1	0.463707362	7.05E-30
13314	PCDHGA10	-0.166433071	0.000109907
13315	IZUMO4	0.586294845	1.05E-50
13316	IRX2	0.0216914	0.616646372
13317	PHOSPHO1	0.047574608	0.272007119
13318	PCDHB8	-0.148923795	0.00054856
13319	ZDHHC15	-0.392313006	3.95E-21
13320	OXTR	0.162949327	0.000153373
13321	CLEC9A	0.038115604	0.37892749
13322	ELOVL4	-0.15315078	0.000377841
13323	CD207	-0.011007422	0.799484274
13324	CCDC157	0.467510263	2.10E-30
13325	GLIPR1L2	-0.243112805	1.23E-08
13326	LRRC39	0.144996255	0.000768993
13327	SAA2-SAA4	0.133751493	0.001932684
13328	NDST2	0.316918574	6.02E-14
13329	ASCL2	0.40029757	5.24E-22
13330	ZNF681	-0.341888029	4.09E-16
13331	FAM218A	0.183924565	1.86E-05
13332	RP11-295K3.1	0.242104764	1.42E-08
13333	TRAIP	0.481288012	2.27E-32
13334	EFCAB13	0.00311538	0.942688984
13335	PGBD4	0.198763227	3.60E-06
13336	AC055866.1	0.105728119	0.014419501
13337	TSPAN32	0.448370152	8.07E-28
13338	HIST1H2AD	0.143651057	0.00086167

13339	CACNA2D3	0.142144243	0.000977684
13340	LRRC36	0.218885123	3.17E-07
13341	IRF4	0.197147952	4.34E-06
13342	AURKC	0.432132312	9.46E-26
13343	DEPDC1B	0.213807576	5.99E-07
13344	LDB3	-0.081967465	0.058136447
13345	STK33	-0.117721066	0.006410471
13346	GDF3	0.129944663	0.002600665
13347	HIST1H2BE	0.179742287	2.89E-05
13348	ADAMTSL1	-0.156458903	0.000280322
13349	KDM4D	-0.190468683	9.17E-06
13350	ITIH3	0.065259897	0.131671643
13351	SERHL2	0.184069424	1.83E-05
13352	FGF9	-0.212042416	7.44E-07
13353	ICOS	0.13512952	0.001732513
13354	HOXC11	0.253087215	2.90E-09
13355	HTR1F	-0.142553778	0.000944803
13356	ART5	-0.111215507	0.010041524
13357	C19orf80	0.10019694	0.020450567
13358	HOXA2	0.202406039	2.36E-06
13359	C2orf82	0.506238788	3.65E-36
13360	DRD4	0.419602495	3.14E-24
13361	ZNF829	-0.291734687	5.89E-12
13362	LAMP5	0.11642623	0.007021536
13363	BFSP1	0.160086969	0.000200673
13364	GRID1	0.040499298	0.349817123
13365	CD5L	-0.056759283	0.18991445
13366	CBX2	0.158256258	0.000237755
13367	SLC27A5	0.140615788	0.001109958
13368	PARPBP	0.061538615	0.155197468
13369	AXIN2	0.098451085	0.022763009
13370	C9orf24	-0.064564633	0.135843975
13371	CPT1B	0.426700074	4.40E-25
13372	KCNT2	0.026651317	0.538479327
13373	ACSM1	0.071818975	0.097028035
13374	PDE3B	-0.056059336	0.195443893
13375	EPHX4	-0.102590456	0.017613727
13376	GTF2IRD2	0.151370923	0.000442586
13377	MAP1LC3C	0.096525446	0.025573127
13378	SLC36A2	-1.16E-05	0.99978667
13379	ATG9B	0.129193714	0.002755033
13380	CENPA	0.475824529	1.40E-31
13381	MSS51	0.399573775	6.31E-22
13382	ASB16	0.156189119	0.000287294
13383	FAM3B	-0.13451737	0.001818963
13384	PRDM16	-0.23808645	2.48E-08
13385	ZNF215	-0.109227754	0.011468034
13386	DGKI	-0.302553011	8.68E-13
13387	GPR141	-0.098624245	0.022523951
13388	ZNF169	0.229096574	8.41E-08
13389	HOXA1	0.090574886	0.036223511
13390	TIAF1	0.259070003	1.18E-09
13391	TDRD9	0.050394882	0.244565842
13392	TMPPE	-0.430457079	1.52E-25
13393	SMIM22	0.073347244	0.090105399
13394	PRDM5	-0.177133052	3.79E-05
13395	KIF18B	0.561610191	8.51E-46
13396	ZNF541	-0.018335145	0.672195474

13397	BEND3	-0.0480444	0.267295765
13398	ADAMTS13	0.43631675	2.84E-26
13399	WBSCR17	-0.16233068	0.000162609
13400	PPARGC1B	-0.013535464	0.754768298
13401	RNF182	-0.13241385	0.002147032
13402	HTRA4	0.211228329	8.22E-07
13403	ITPRIPL1	0.303018897	7.98E-13
13404	NANOS1	-0.217961637	3.56E-07
13405	KCND1	0.412641122	2.07E-23
13406	GRIK5	-0.111341249	0.009956829
13407	ZNF781	0.023671536	0.584845899
13408	ZNF667	-0.247915918	6.18E-09
13409	SMCO3	0.004201814	0.922757081
13410	COLQ	0.371626735	5.78E-19
13411	EFCAB6	-0.182245569	2.22E-05
13412	BCHE	0.031070746	0.473274976
13413	ZNF114	0.007208526	0.867884777
13414	MIA2	-0.421232358	2.01E-24
13415	B4GALNT2	-0.259241592	1.15E-09
13416	C5orf63	0.020234885	0.640508937
13417	SYNE4	-0.040866745	0.34545906
13418	LILRA4	-0.002781813	0.948816439
13419	DNAH5	-0.067219826	0.120442422
13420	CCR10	0.314500604	9.53E-14
13421	PRSS36	0.420384933	2.53E-24
13422	GFRA2	0.12741019	0.003155663
13423	TMIE	0.031410098	0.468453706
13424	RND2	0.194679651	5.74E-06
13425	YBX2	0.222571867	1.98E-07
13426	MYOT	0.07410602	0.086818705
13427	LRRC3	-0.010124667	0.815262551
13428	ZNF382	-0.143565451	0.000867904
13429	CNKSR1	0.03505483	0.418415763
13430	ASPM	0.211680279	7.78E-07
13431	KIF18A	0.152156929	0.000412815
13432	HSD17B3	0.498694538	5.53E-35
13433	POLR3G	0.005245039	0.903663179
13434	DACT2	-0.127506673	0.003132704
13435	CATIP	0.19758537	4.13E-06
13436	RAB27B	-0.17166902	6.58E-05
13437	IL12RB2	-0.096569449	0.025505716
13438	LRAT	-0.025723013	0.552725074
13439	C15orf27	0.162335656	0.000162533
13440	GPR18	0.078412823	0.069948686
13441	MAT1A	0.072191051	0.095304766
13442	TNF	0.10285152	0.017326166
13443	RP11-35N6.1	-0.149753679	0.00051023
13444	GPR64	-0.04938025	0.254206042
13445	ANO2	0.019381529	0.654666027
13446	COL7A1	0.358477651	1.15E-17
13447	OTUB2	-0.205205696	1.70E-06
13448	HRASLS	-0.015169179	0.726290957
13449	TTC36	-0.015667006	0.717685716
13450	PTGDR	0.233856208	4.44E-08
13451	SLITRK2	0.03811062	0.378989865
13452	MAB21L3	-0.321614236	2.44E-14
13453	FKSG62	0.171016592	7.02E-05
13454	KLLN	-0.270271241	2.08E-10

13455	PRDM8	0.318822468	4.18E-14
13456	ATP5EP2	0.210965529	8.49E-07
13457	MORN3	0.184059777	1.84E-05
13458	CD1E	-0.001837937	0.966170095
13459	SRGAP2B	-0.276503617	7.62E-11
13460	NRIP3	-0.163875222	0.000140465
13461	GNB1L	0.56934461	2.73E-47
13462	CRB2	0.069679257	0.107425508
13463	TRIM7	0.150011043	0.000498858
13464	LRRC34	-0.181392808	2.43E-05
13465	POMC	0.278141838	5.83E-11
13466	RIC3	-0.114457745	0.008050683
13467	CKAP2L	0.214936462	5.20E-07
13468	VN1R1	0.08059854	0.062473396
13469	PARP15	0.304995864	5.57E-13
13470	CSRNP3	-0.298899633	1.67E-12
13471	ADCY8	-0.142097765	0.000981482
13472	ATP6V1G2	0.09961013	0.021203926
13473	BGLAP	0.540877387	5.59E-42
13474	MROH8	-0.105322841	0.014801149
13475	ANKRD45	-0.233171854	4.87E-08
13476	C14orf180	0.031492912	0.467281269
13477	RNF224	0.221323479	2.32E-07
13478	KCNIP2	0.273230943	1.29E-10
13479	VTN	0.092927102	0.031631655
13480	RAPH1	-0.41847665	4.27E-24
13481	PROCA1	0.361486744	5.85E-18
13482	KCTD16	-0.245053498	9.32E-09
13483	C15orf59	-0.171452596	6.72E-05
13484	MEMO1	0.014293913	0.741503691
13485	SH2D1B	-0.157561442	0.000253438
13486	WDR88	0.179697789	2.91E-05
13487	TTK	0.202261797	2.40E-06
13488	TIAM2	-0.016722447	0.699560648
13489	CDO1	-0.060328257	0.163498198
13490	LILRA6	0.232905494	5.05E-08
13491	CFAP43	-0.08778322	0.042397005
13492	CD300LG	-0.049876551	0.249458148
13493	DENND2C	-0.27962816	4.56E-11
13494	BDNF	-0.043043645	0.32035223
13495	C5orf38	0.202744845	2.27E-06
13496	PLA2G1B	0.184761922	1.70E-05
13497	BMP8A	0.105191222	0.014926989
13498	TWIST1	0.241385898	1.57E-08
13499	GPR84	0.288722143	9.89E-12
13500	YPEL4	0.175476943	4.48E-05
13501	ZBTB20	-0.287232761	1.28E-11
13502	NPIPA1	0.496007943	1.43E-34
13503	FAM221A	0.003916938	0.927979311
13504	NKAPL	-0.119963371	0.005464239
13505	NLGN4X	-0.191383638	8.29E-06
13506	CYP21A2	0.332103906	3.06E-15
13507	GTF2H2C	-0.160335662	0.000196075
13508	RAB43	-0.174493658	4.95E-05
13509	KBTBD8	-0.156944789	0.000268162
13510	GPR85	-0.012923943	0.765516235
13511	CCDC138	-0.024245894	0.575766146
13512	RUNX1T1	-0.183228437	2.01E-05

13513	OCA2	0.000614078	0.988694001
13514	CCDC153	0.398165495	9.04E-22
13515	C11orf21	0.391714281	4.59E-21
13516	SIDT1	0.032065445	0.459220226
13517	NMU	0.145029584	0.000766819
13518	RFX8	0.278731637	5.29E-11
13519	EIF5AL1	0.197422695	4.20E-06
13520	SOX11	-0.010831758	0.802617808
13521	FAM184A	-0.135610416	0.00166726
13522	PRSS53	0.475149071	1.75E-31
13523	C17orf67	0.305136587	5.43E-13
13524	AC110602.1	0.095164185	0.027735829
13525	GUCA1B	0.332311826	2.93E-15
13526	C9orf173	0.181388981	2.43E-05
13527	DAPK2	-0.086755025	0.044884105
13528	ELFN2	-0.028150272	0.515870845
13529	ACTL10	0.378003287	1.29E-19
13530	ZNF223	-0.195904913	4.99E-06
13531	KCNJ12	-0.008447013	0.845450513
13532	FLT3	0.014981857	0.729537947
13533	GPR1	-0.044534458	0.303862418
13534	PLIN4	0.069569016	0.107984159
13535	CCDC170	-0.06992516	0.10618761
13536	LMTK3	-0.004135787	0.923967171
13537	RAB39A	-0.163585293	0.000144393
13538	FCN2	-0.05269009	0.223710746
13539	CYP4F2	0.008697229	0.84093256
13540	C3orf67	-0.119361131	0.005705172
13541	PCDH7	-0.007103594	0.869790688
13542	GPX2	0.048917543	0.25868871
13543	TPSD1	-0.02016726	0.641626163
13544	RAET1G	-0.00721251	0.867812433
13545	FSIP1	-0.107149435	0.013148585
13546	HIST1H2AG	-0.026092706	0.547029738
13547	ESRRB	-0.113126099	0.008821231
13548	SPATA6L	0.126427278	0.003398428
13549	FAM171A2	0.223526189	1.75E-07
13550	MYO3B	-0.168530191	8.96E-05
13551	PROX1	-0.013079929	0.762770284
13552	PLIN5	-0.024517722	0.571492223
13553	AC079907.1	0.239307906	2.10E-08
13554	GVQW1	0.042702488	0.32420627
13555	RHBG	-0.195699173	5.11E-06
13556	CAPN11	0.214018563	5.83E-07
13557	OSR2	0.158759589	0.000226967
13558	STK32A	-0.154639763	0.000330577
13559	ZBED2	0.252477264	3.17E-09
13560	CCDC15	-0.211518392	7.94E-07
13561	MDGA1	0.118052915	0.00626175
13562	TMEM179	0.144339699	0.000813008
13563	TENM3	0.070994472	0.100935252
13564	CLECL1	0.147413262	0.000625274
13565	PTGER1	0.175310322	4.56E-05
13566	RAB39B	0.060941495	0.159251789
13567	FAM151B	-0.114292062	0.008143163
13568	SERPINA4	-0.128847123	0.002829057
13569	LYPD3	0.15833979	0.000235932
13570	KCNK2	-0.087179119	0.043843779

13571	C21orf58	0.536361875	3.50E-41
13572	CHRDL2	0.165599602	0.000119101
13573	LRRC37A2	0.220903104	2.45E-07
13574	ENTPD8	0.292345708	5.30E-12
13575	CLDN11	0.072463037	0.094060596
13576	TMEM155	0.175895738	4.30E-05
13577	IFNG	0.271254202	1.78E-10
13578	CCL23	0.188690177	1.11E-05
13579	MS4A2	-0.212493697	7.04E-07
13580	SRL	-0.12473216	0.00385723
13581	MMS22L	-0.178530982	3.28E-05
13582	REP15	0.161473179	0.000176277
13583	SYCP2	0.013993968	0.746740572
13584	FBF1	0.625610827	1.85E-59
13585	GNGT1	0.007693248	0.859090719
13586	ZC3H12D	0.198189981	3.85E-06
13587	P2RY2	0.183132142	2.03E-05
13588	AGAP4	0.404599494	1.72E-22
13589	WNT10A	0.247970446	6.13E-09
13590	AGBL2	0.401349383	4.00E-22
13591	SKA1	0.39209287	4.18E-21
13592	ICOSLG	0.063770537	0.140733126
13593	SCUBE2	-0.047859317	0.269145164
13594	FAM122C	0.086227517	0.046207002
13595	FAM163A	0.004545597	0.916459461
13596	SLC5A2	0.004384945	0.919401752
13597	SLC25A53	-0.06288241	0.146359808
13598	COL13A1	0.213447946	6.26E-07
13599	ANKRD1	0.002997424	0.944855403
13600	ENKUR	-0.031163315	0.471957124
13601	LRRC43	0.082326881	0.057040054
13602	NPAS1	0.330453009	4.26E-15
13603	CCDC178	-0.149317112	0.000530072
13604	NPHS1	0.080573375	0.062555557
13605	TNFRSF11A	0.006789503	0.875500092
13606	C2orf54	-0.016139123	0.70955775
13607	SLN	0.119575601	0.005618297
13608	LTA	0.365936724	2.14E-18
13609	CASC5	0.116146695	0.007160111
13610	PLA2G4F	-0.208353359	1.17E-06
13611	MYOCD	-0.121661987	0.004833246
13612	FBXO15	-0.1474105	0.000625423
13613	C1orf168	-0.201290436	2.69E-06
13614	ADAMTS14	0.321929351	2.29E-14
13615	MYL4	0.325424289	1.16E-14
13616	PVRL4	0.01970504	0.649284067
13617	PODNL1	0.138226911	0.001350071
13618	MAP3K9	-0.120632337	0.00520738
13619	LRRC2	-0.203264728	2.14E-06
13620	CCNO	0.234984828	3.80E-08
13621	ZNF713	-0.195599398	5.17E-06
13622	FAM180A	0.016459936	0.704053292
13623	SCX	0.440411229	8.61E-27
13624	PPBP	0.021415054	0.621143932
13625	THRSP	-0.139640086	0.001202827
13626	JMJD7-PLA2G4B	0.290909187	6.79E-12
13627	DYX1C1	0.15498377	0.000320472
13628	PQLC2L	0.091874701	0.033620612

13629	PTPRN	0.145646039	0.000727613
13630	SIM2	0.105029593	0.015082806
13631	A4GNT	0.043496794	0.315279391
13632	RP11-691N7.6	0.019817648	0.64741496
13633	LRRC46	0.36405591	3.28E-18
13634	AHRR	0.156773063	0.000272402
13635	NDRG4	0.114424857	0.008068966
13636	PRKG2	-0.210610633	8.87E-07
13637	FAM83F	-0.233316798	4.77E-08
13638	NMNAT2	0.063116926	0.14485763
13639	GYG2	0.069760723	0.107014146
13640	SLC9A4	-0.193754924	6.36E-06
13641	CPS1	-0.012832475	0.767127773
13642	CCDC173	-0.042292685	0.328875456
13643	ORC6	0.454789967	1.14E-28
13644	RELL2	0.443245285	3.73E-27
13645	FGFBP3	0.240975343	1.66E-08
13646	ZNF442	-0.198665004	3.65E-06
13647	C19orf67	0.082425509	0.056742207
13648	TCAP	0.389728232	7.52E-21
13649	AC242988.1	0.094256014	0.029264603
13650	CPA4	0.110492274	0.010541203
13651	RAD54B	0.176098074	4.21E-05
13652	PCDHGA2	-0.191918965	7.81E-06
13653	SLFN12L	0.147770437	0.000606285
13654	PCDHGA7	-0.089480994	0.038544927
13655	KCNA3	-0.071462666	0.098701481
13656	C6orf52	0.261189589	8.57E-10
13657	GRHL1	-0.043617775	0.313934001
13658	PTCH2	0.373195398	4.01E-19
13659	GABRP	-0.088700861	0.040276484
13660	SOX15	0.279782287	4.45E-11
13661	MTTP	0.063663543	0.141402064
13662	MSI1	0.180546733	2.66E-05
13663	TNMD	-0.075197334	0.082261546
13664	PDXP	0.033568496	0.438431917
13665	SH2D5	0.247474391	6.58E-09
13666	FIBCD1	0.188295643	1.16E-05
13667	RP11-166B2.1	0.299535638	1.49E-12
13668	JPH4	-0.119223318	0.005761632
13669	SAPCD1	0.528162187	9.13E-40
13670	PLAC8L1	0.452147894	2.57E-28
13671	BAI2	0.156496168	0.000279371
13672	AOC2	0.332647897	2.74E-15
13673	EXO1	0.260012771	1.03E-09
13674	CACNA2D2	0.010412093	0.810116597
13675	CTC-479C5.12	0.523870819	4.86E-39
13676	CTD-2313N18.7	-0.218198204	3.46E-07
13677	KCNJ13	-0.019830334	0.647204547
13678	B4GALNT4	0.171984613	6.37E-05
13679	RGS7	-0.005500202	0.89900099
13680	TAS2R14	0.124472707	0.003932206
13681	PDE11A	-0.104541824	0.015561731
13682	LEFTY1	0.011575846	0.78936693
13683	ERBB4	-0.247720076	6.36E-09
13684	DHRS9	-0.051871502	0.230998188
13685	HS6ST2	-0.054548347	0.207779822
13686	SOX5	-0.178534519	3.28E-05

13687	NR6A1	0.175341836	4.55E-05
13688	CPO	0.196530665	4.65E-06
13689	SCGB2A1	0.04105669	0.343219833
13690	AC019206.1	0.402713026	2.81E-22
13691	GPR17	0.074441527	0.085396535
13692	RAB41	0.09676412	0.025209321
13693	ZSCAN32	-0.27007406	2.14E-10
13694	LBX2	0.558224564	3.73E-45
13695	AGXT	0.093015494	0.031469279
13696	WDR93	0.116668169	0.006903543
13697	PKMYT1	0.583096513	4.78E-50
13698	HOXD1	-0.189028117	1.07E-05
13699	MTFR2	0.338796165	7.78E-16
13700	MYH8	-0.042212939	0.329789078
13701	SULT1A2	0.141522765	0.001029606
13702	FAM90A1	0.185077964	1.65E-05
13703	ZNF727	-0.34152671	4.41E-16
13704	NNAT	0.038728778	0.371300799
13705	CEACAM4	0.371822327	5.52E-19
13706	JAKMIP1	0.311474455	1.68E-13
13707	FAM155B	0.033370342	0.441141274
13708	AC073657.1	0.097116135	0.024680905
13709	DGKG	0.025340349	0.558650615
13710	APBA2	0.238050374	2.50E-08
13711	KCNB1	-0.064918315	0.133708957
13712	ANKHD1-EIF4EBP3	0.279353014	4.78E-11
13713	HIST1H2BH	0.252502415	3.16E-09
13714	SLC2A12	-0.310979394	1.85E-13
13715	EPN3	-0.179111886	3.09E-05
13716	JSRP1	0.345717441	1.83E-16
13717	DIRAS1	-0.106662147	0.01357272
13718	AC012363.2	0.051547745	0.233926344
13719	MYZAP	-0.198652704	3.65E-06
13720	CST6	0.080756159	0.061960828
13721	TLR10	0.039950764	0.356387381
13722	MAEL	-0.108803006	0.011795152
13723	OGFOD2	0.235982057	3.32E-08
13724	ODF3L1	0.219376867	2.98E-07
13725	CHODL	-0.081571083	0.059365791
13726	CCL11	0.148618595	0.000563317
13727	COL11A1	0.050238059	0.24603889
13728	AC114494.1	0.187470667	1.27E-05
13729	MEI1	0.40158571	3.76E-22
13730	SLC9A2	-0.222876976	1.90E-07
13731	OTUD7A	-0.259125106	1.17E-09
13732	C19orf35	0.145489252	0.000737404
13733	RP11-903H12.5	-0.193104812	6.84E-06
13734	MYCN	-0.067251195	0.120268976
13735	CDKL3	0.095335682	0.027454981
13736	FGF13	-0.260496646	9.53E-10
13737	BTBD8	-0.409918925	4.27E-23
13738	ASXL3	-0.349571122	8.02E-17
13739	DKK1	0.069132821	0.110217109
13740	SEC14L4	0.269845136	2.22E-10
13741	IL10	-0.05297275	0.221232867
13742	EME1	0.527720904	1.09E-39
13743	FCGR1B	0.302529421	8.72E-13
13744	LYPD5	0.041949769	0.332815774

13745	XCR1	-0.013518723	0.755061903
13746	PLIN1	0.17789796	3.50E-05
13747	E2F8	0.092431496	0.032555413
13748	GPR82	-0.183555706	1.94E-05
13749	NBPF26	-0.007901386	0.855319852
13750	CXCL3	0.327680307	7.41E-15
13751	PDE4C	0.115106211	0.007697678
13752	AC024361.1	0.215344099	4.95E-07
13753	AKAP3	-0.304352463	6.26E-13
13754	SPRY3	-0.272595761	1.43E-10
13755	SYCE2	0.476693824	1.05E-31
13756	ZNF852	-0.223374806	1.78E-07
13757	CCL26	0.294304029	3.76E-12
13758	GINS4	0.077897594	0.071813497
13759	SYT12	0.047565452	0.272099495
13760	POU2AF1	0.203139314	2.17E-06
13761	DDX43	-0.011083433	0.798129386
13762	PANX2	0.206179386	1.51E-06
13763	GPR75-ASB3	0.097675304	0.0238612
13764	GAD1	0.088741661	0.040184322
13765	APOC3	0.093278726	0.030989939
13766	ZNF497	0.236934244	2.91E-08
13767	TMEM110	-0.012198349	0.778327561
13768	TMEM154	-0.050993554	0.238999298
13769	CEMIP	0.033793384	0.435368618
13770	PRR33	0.228084382	9.62E-08
13771	FAM153A	0.132333744	0.002160533
13772	GCGR	-0.178516712	3.28E-05
13773	IBSP	0.024990425	0.564095985
13774	DDX47	0.325688134	1.10E-14
13775	ZNF273	-0.000897373	0.983478777
13776	HOMER2	-0.055479251	0.200114903
13777	CEP128	0.040200235	0.353389703
13778	C21orf33	0.088317185	0.04115193
13779	SCGB3A2	0.034798034	0.421835127
13780	TSPAN10	0.14307188	0.000904668
13781	BCAN	0.037060933	0.39226844
13782	MMP16	-0.072677918	0.093086858
13783	LONRF2	-0.200919033	2.81E-06
13784	PI16	0.034591226	0.424600728
13785	BCL11A	-0.048849983	0.259347775
13786	ITGAD	0.08887287	0.039889148
13787	ABCB9	0.14969855	0.000512697
13788	CEL	-0.019157379	0.658405554
13789	CDH23	-0.034925282	0.420138722
13790	HRG	-0.10752059	0.012833417
13791	FOLR3	-0.136274566	0.001580845
13792	PSD	0.405604644	1.33E-22
13793	WDR86	0.072168751	0.095407354
13794	CTSV	0.144476987	0.000803616
13795	TMEM232	-0.312630537	1.36E-13
13796	PHKG1	0.322439605	2.08E-14
13797	QRICH2	0.372392077	4.83E-19
13798	ARHGEF38	-0.211821086	7.65E-07
13799	ENPP6	-0.145495128	0.000737035
13800	LRP8	0.267856899	3.04E-10
13801	CABYR	0.139383669	0.001228394
13802	CAMK4	-0.087419333	0.043263582

13803	TMIGD1	0.099904676	0.020822821
13804	MYH3	0.427875631	3.16E-25
13805	SGCD	-0.06202477	0.151954689
13806	AC006547.14	0.43488063	4.30E-26
13807	WNT6	0.133848668	0.001917903
13808	SPATA17	-0.010331409	0.811560311
13809	RLTPR	0.541361515	4.59E-42
13810	CCNE2	0.094607515	0.028664534
13811	CXCR2	-0.089393998	0.038734842
13812	DNAJC5B	0.070050295	0.105562015
13813	GPR75	-0.05687495	0.189011882
13814	FCRL3	0.200258287	3.03E-06
13815	ZBP1	0.402899072	2.68E-22
13816	TFR2	0.042701363	0.324219033
13817	CTC-260F20.3	0.532784099	1.47E-40
13818	TLE6	0.276648906	7.44E-11
13819	ZNF665	-0.141668826	0.001017179
13820	GRHL2	-0.209656062	9.96E-07
13821	ZNF66	-0.030628643	0.479596676
13822	TNFRSF17	0.199929797	3.15E-06
13823	GALNT5	-0.089335454	0.038863089
13824	SLC10A5	-0.161538521	0.000175199
13825	FIGF	0.122715008	0.004475872
13826	KCNH2	0.04242496	0.327363623
13827	HIST1H2BN	0.04027211	0.352528994
13828	ZNF285	-0.221458359	2.28E-07
13829	PCDHGB1	-0.120372746	0.005305733
13830	FAM43B	0.131916188	0.002232176
13831	SPRED3	0.226404955	1.20E-07
13832	HBA1	0.136461068	0.001557329
13833	ASGR1	0.347858075	1.16E-16
13834	CYP2D6	0.30781584	3.32E-13
13835	HSD17B6	-0.014469164	0.738449423
13836	PNPLA3	-0.008282466	0.848424381
13837	DIRC3	0.08133981	0.060092949
13838	SRGAP3	-0.184506772	1.75E-05
13839	RP11-849H4.2	0.03863644	0.372443181
13840	CCDC7	-0.055942664	0.196376906
13841	MRAP2	-0.000803771	0.985201849
13842	SLC22A24	-0.110085463	0.010831854
13843	GSDMA	0.070674623	0.102484221
13844	PRR36	0.052235587	0.22773643
13845	NELL1	0.02233012	0.606306119
13846	NPIPB5	0.397102535	1.18E-21
13847	ZNF891	-0.073895223	0.087721953
13848	CLNK	-0.202352822	2.38E-06
13849	HIST1H2AM	0.185831065	1.52E-05
13850	EPB41L4B	-0.182947308	2.07E-05
13851	TRIM46	0.409253615	5.09E-23
13852	UNC5A	0.113568069	0.008558498
13853	MCOLN3	-0.116468281	0.007000899
13854	CATSPER2	0.270678745	1.95E-10
13855	RNFT2	0.383458056	3.49E-20
13856	TMEM145	0.208829879	1.10E-06
13857	AP000866.1	0.200247735	3.04E-06
13858	DEPDC1	0.154189273	0.00034426
13859	NIM1K	0.097997433	0.023399753
13860	CLSPN	0.068404569	0.114026034

13861	ZNF469	0.092405328	0.032604821
13862	DPYSL4	0.097852626	0.023606222
13863	HYPK	0.380586563	6.97E-20
13864	ANKRD36	0.030289528	0.484476697
13865	FGF18	0.1432365	0.000892249
13866	PSTK	0.02608081	0.547212551
13867	CYB5R2	0.051593223	0.23351345
13868	FFAR4	-0.038435888	0.374931854
13869	ZNF385D	-0.02255576	0.602671797
13870	ADM5	0.594271873	2.19E-52
13871	EBLN2	0.016665672	0.700531418
13872	TDO2	0.102394902	0.017831854
13873	FOXI2	-0.220871963	2.46E-07
13874	C19orf73	0.434488568	4.81E-26
13875	SLC4A9	-0.145491119	0.000737287
13876	TREML1	0.324697782	1.34E-14
13877	ZCWPW2	-0.399715703	6.08E-22
13878	IQCH	-0.282312029	2.92E-11
13879	CH507-9B2.5	0.062245838	0.150497234
13880	GPAT2	0.227840987	9.94E-08
13881	CNTN3	-0.246074424	8.05E-09
13882	MORN5	0.119642676	0.005591372
13883	AGR2	-0.051438631	0.23491909
13884	BRCA2	-0.06019776	0.164412718
13885	PABPC5	-0.178575554	3.26E-05
13886	TEN1-CDK3	0.46550819	3.98E-30
13887	SP5	0.145939162	0.00070963
13888	TVP23C	0.112822502	0.009005847
13889	VSTM2L	0.16530793	0.000122485
13890	LYPD8	0.001138665	0.979037358
13891	NLRP2	0.084515889	0.050726932
13892	ALDH3A1	0.212658862	6.90E-07
13893	PAEP	0.219351375	2.99E-07
13894	ZC3H12B	-0.206374281	1.48E-06
13895	KAAG1	0.16857239	8.93E-05
13896	TPBGL	0.187987283	1.20E-05
13897	PNMAL2	0.004863483	0.910640864
13898	C9orf50	0.300543557	1.25E-12
13899	RP11-111M22.2	0.052600493	0.224500291
13900	KLRD1	-0.005136362	0.905649868
13901	RELN	-0.044927718	0.299608022
13902	KIAA1755	0.210854513	8.61E-07
13903	CENPP	0.121693438	0.00482221
13904	C16orf59	0.536505002	3.30E-41
13905	IYD	-0.215175804	5.05E-07
13906	ZNF454	-0.112970432	0.008915466
13907	MTMR7	-0.101719354	0.018603644
13908	MEFV	0.177339997	3.71E-05
13909	PNPLA1	0.043419264	0.316143557
13910	APOBEC3A	0.16290943	0.000153953
13911	EFR3B	-0.144824126	0.000780314
13912	TMIGD2	0.375996261	2.08E-19
13913	GABRQ	-0.107297396	0.013022134
13914	XRCC2	0.27629049	7.89E-11
13915	SPTBN5	0.378698235	1.09E-19
13916	HAMP	0.267051308	3.46E-10
13917	RP11-505K9.4	0.13572585	0.001651936
13918	CCDC181	0.009323988	0.829638908

13919	BLM	0.283796305	2.28E-11
13920	AC022431.2	0.063322513	0.143550471
13921	EYA4	-0.080276197	0.063532537
13922	YY2	-0.04211755	0.330884086
13923	ZNF221	-0.275552678	8.90E-11
13924	CREG2	-0.16491723	0.000127161
13925	HIST1H1E	0.004495381	0.917379033
13926	BRIP1	0.116753035	0.006862577
13927	FDCSP	0.207391051	1.31E-06
13928	NPIPB3	0.408804576	5.73E-23
13929	SNURF	-0.035127693	0.417448527
13930	CD226	-0.065466539	0.130450859
13931	RFESD	-0.248895496	5.36E-09
13932	SLC26A4	-0.225684562	1.32E-07
13933	MTMR8	-0.173337668	5.57E-05
13934	BAAT	-0.055063404	0.203513092
13935	KCNE2	0.155889055	0.00029524
13936	AC073130.2	-0.002828542	0.947957849
13937	NME2	0.297371406	2.19E-12
13938	FAM132B	0.204474146	1.85E-06
13939	JPH1	-0.013764472	0.750755343
13940	GCSAM	0.031966649	0.460605669
13941	FAM159A	0.283837433	2.26E-11
13942	SLC25A18	0.106471498	0.013741912
13943	RHOF	0.192429272	7.38E-06
13944	SCUBE1	0.027231949	0.529663273
13945	CUZD1	0.149455675	0.000523698
13946	ALG1L	-0.119285047	0.005736281
13947	SAMD3	0.192026791	7.72E-06
13948	CNTFR	-0.013325527	0.758452885
13949	ATP13A4	-0.21124121	8.21E-07
13950	ZDHHC11	0.409310005	5.01E-23
13951	COL10A1	-0.030668344	0.479027104
13952	ASIC3	0.613595167	1.18E-56
13953	NRG2	-0.136801613	0.001515217
13954	RTEL1-TNFRSF6B	0.463361266	7.87E-30
13955	EFCAB12	0.15346189	0.000367473
13956	ZNF410	0.004747998	0.912754171
13957	HHIPL1	0.065635747	0.129457758
13958	RBM14-RBM4	0.22066145	2.53E-07
13959	FAM166B	0.450680738	4.01E-28
13960	NRK	-0.126339357	0.003420949
13961	GSTM5	0.057829265	0.181685258
13962	PYGO1	-0.042928468	0.321650024
13963	LRRC16B	0.374884597	2.70E-19
13964	CENPE	0.238580188	2.32E-08
13965	KIAA0825	-0.361787012	5.47E-18
13966	UNC5D	-0.241771844	1.48E-08
13967	RP11-514O12.4	0.264887379	4.84E-10
13968	TNNT1	0.375855943	2.15E-19
13969	ASIP	0.296651847	2.49E-12
13970	KCNJ14	0.401032242	4.34E-22
13971	CPA6	-0.078615215	0.06922708
13972	KHDC1	0.324775459	1.32E-14
13973	NPY5R	-0.062924635	0.146088467
13974	SULT2B1	0.217696806	3.68E-07
13975	LRGUK	0.161730054	0.000172074
13976	TMEM240	0.296383021	2.61E-12

13977	MS4A1	0.177041678	3.82E-05
13978	PHYHIP	0.257112781	1.59E-09
13979	NBPF14	0.187320611	1.29E-05
13980	OCLM	0.057453296	0.184546217
13981	RHOXF1	0.367868003	1.38E-18
13982	INSL3	0.44285211	4.19E-27
13983	CRABP1	0.045731382	0.29103752
13984	ZNF490	-0.332621597	2.75E-15
13985	CDC25C	0.405639076	1.32E-22
13986	SCN3B	-0.055263027	0.201876637
13987	ST6GAL2	-0.077539066	0.07313496
13988	GUCY2C	-0.327561477	7.59E-15
13989	NPIPB4	0.273782117	1.18E-10
13990	CDC25A	0.281436908	3.38E-11
13991	LRRIQ1	-0.09477205	0.028387295
13992	PRR4	0.087759425	0.042453219
13993	CCBE1	-0.09103547	0.035282236
13994	DUOX1	0.000140802	0.99740757
13995	RP3-461F17.3	0.238384127	2.38E-08
13996	WNT11	0.025568512	0.555113809
13997	PCDHGA4	-0.228340809	9.30E-08
13998	HAS2	0.079487531	0.066186376
13999	FKBP1C	-0.026544243	0.540113068
14000	ETV2	0.535220377	5.54E-41
14001	LAIR2	0.294657492	3.54E-12
14002	ZGRF1	0.209631127	9.99E-07
14003	RNF175	0.109281505	0.011427215
14004	MTBP	0.126859451	0.003289672
14005	FGF11	-0.003952983	0.927318375
14006	TDGF1	-0.197027632	4.40E-06
14007	KCNK15	-0.180820274	2.58E-05
14008	SRRM3	0.023271807	0.591203932
14009	ANKRD34A	0.28929571	8.97E-12
14010	TTC34	0.169380358	8.25E-05
14011	ERVMER34-1	-0.066795096	0.122810135
14012	CCDC78	0.450261991	4.56E-28
14013	VIP	0.26628543	3.90E-10
14014	ATRNL1	-0.258824598	1.23E-09
14015	KCNN2	-0.07370035	0.088563683
14016	SLC16A8	0.322870481	1.91E-14
14017	PLXNB3	0.353063528	3.77E-17
14018	FAM86B1	0.083793367	0.052742828
14019	CXCR1	0.029935414	0.489601063
14020	CYP4F12	0.305252048	5.32E-13
14021	PGR	-0.316913141	6.03E-14
14022	REEP2	0.218586448	3.29E-07
14023	SCHIP1	0.040023032	0.355517356
14024	RPS6KA5	-0.252711742	3.07E-09
14025	CFTR	-0.207475508	1.30E-06
14026	NEFM	0.009842818	0.820316402
14027	FEM1A	0.118655018	0.0059998
14028	SLC9A5	0.441051574	7.13E-27
14029	RNF39	-0.040867419	0.345451099
14030	E2F2	0.381282004	5.90E-20
14031	HCAR1	-0.187771317	1.23E-05
14032	KIF15	0.287696753	1.18E-11
14033	FAM131B	-0.095390588	0.027365583
14034	ZNF233	-0.147172893	0.000638362

14035	NEB	0.186462075	1.42E-05
14036	RETN	0.274525618	1.05E-10
14037	ATP2A1	0.482462684	1.53E-32
14038	TEX15	0.022350163	0.605982895
14039	PRR34	-0.128291904	0.00295142
14040	ZHX1-C8orf76	0.572805026	5.68E-48
14041	TFAP2B	-0.156401482	0.000281793
14042	SFTA2	-0.131651042	0.002278788
14043	CYP2C9	0.172543643	6.03E-05
14044	SLCO1C1	-0.092883237	0.0317125
14045	NTNG2	0.2903281	7.51E-12
14046	FAM177B	0.110147025	0.010787419
14047	AC018867.1	0.037352332	0.388554313
14048	PM20D1	-0.063806166	0.140510909
14049	CRACR2A	0.037625238	0.385095337
14050	PRSS12	0.08400528	0.052144768
14051	SPATA4	-0.124030031	0.004063181
14052	LRRC14B	-0.144606991	0.000794815
14053	TRIM36	0.087419863	0.043262309
14054	EDARADD	-0.065668477	0.129266343
14055	PPM1N	0.476684057	1.05E-31
14056	CCDC87	-0.10857653	0.011972923
14057	MYH7B	0.38595366	1.90E-20
14058	PTPRH	0.270659398	1.95E-10
14059	C9orf43	0.257254562	1.56E-09
14060	PITPNM3	0.026982452	0.533442542
14061	GNMT	0.219968962	2.76E-07
14062	TM4SF4	-0.028547021	0.509970015
14063	RYR1	0.155296886	0.000311525
14064	LYPD6B	-0.138700375	0.001298987
14065	HES2	0.070379468	0.103930278
14066	ARTN	0.188737793	1.11E-05
14067	ADPRHL1	0.113446433	0.008630101
14068	VIPR1	-0.060952574	0.159175849
14069	OSR1	0.410234836	3.92E-23
14070	ZNF843	-0.065037237	0.132996924
14071	C3orf80	0.028090681	0.516760171
14072	TNFSF18	-0.134634186	0.001802166
14073	ACRC	0.417604687	5.42E-24
14074	GHRL	0.308102246	3.15E-13
14075	BDKRB1	-0.031097068	0.472900035
14076	CCER2	0.555385596	1.27E-44
14077	TIGD4	-0.207925598	1.23E-06
14078	DNAAF3	0.289315734	8.94E-12
14079	FRMD1	-0.040349559	0.351603028
14080	RAB26	0.232916664	5.04E-08
14081	GTF2H2	-0.032105929	0.458653175
14082	TACR1	-0.261707349	7.92E-10
14083	DBH	0.253214319	2.85E-09
14084	ADAM23	0.009313385	0.829829683
14085	KLK6	-0.006301464	0.884384148
14086	XKR9	0.006049153	0.888982886
14087	IL17B	0.228384711	9.25E-08
14088	OVCH2	-0.121308645	0.004958811
14089	LPPR4	-0.018656508	0.666792376
14090	BMP7	-0.025084712	0.562626226
14091	SIX1	-0.022949126	0.596359462
14092	FOXD4	0.349412466	8.30E-17

14093	HTR7	-0.105319477	0.014804354
14094	WDR92	0.180647191	2.63E-05
14095	HCAR2	0.097997439	0.023399744
14096	PCDHGA5	-0.111706795	0.009714204
14097	CCDC17	0.507732756	2.12E-36
14098	MCM10	0.288946327	9.52E-12
14099	CNTD1	0.343075822	3.19E-16
14100	CH507-9B2.9	0.177254592	3.74E-05
14101	TVP23A	0.197108501	4.36E-06
14102	CCDC39	0.08650735	0.045501208
14103	OR52N4	0.085115109	0.049104259
14104	ETV4	0.124817193	0.003832941
14105	ZNF257	-0.250732356	4.10E-09
14106	PLA2G2A	0.169408898	8.22E-05
14107	PEBP4	-0.144157902	0.000825601
14108	FOXJ1	0.120544781	0.005240368
14109	ZFHX2	0.036731324	0.396495362
14110	AC092384.1	0.19137875	8.29E-06
14111	RLN2	-0.124038993	0.00406049
14112	NUDT10	0.050426468	0.244269902
14113	C10orf67	-0.122266309	0.004625126
14114	DGAT2	0.095609428	0.027011757
14115	PTPRR	-0.125617296	0.003611092
14116	ABHD1	0.221081502	2.40E-07
14117	HRASLS5	-0.195144729	5.44E-06
14118	HSD17B13	-0.059919779	0.166373598
14119	RIBC2	0.199002075	3.51E-06
14120	HIST1H2BF	0.176649798	3.98E-05
14121	PDZD7	0.455684356	8.68E-29
14122	CENPI	0.224333998	1.57E-07
14123	KIAA1324	0.231452951	6.14E-08
14124	POLR2J3	0.23826244	2.42E-08
14125	RUNDC3A	0.184355762	1.78E-05
14126	LMO1	0.20828912	1.17E-06
14127	FAM169A	-0.211929326	7.55E-07
14128	TAS2R20	0.163604201	0.000144134
14129	LHX1	-0.02650466	0.540717647
14130	RGL4	0.206263044	1.50E-06
14131	UCP3	0.416060261	8.24E-24
14132	C2CD4A	0.115906437	0.007281165
14133	PPM1J	0.171522336	6.67E-05
14134	PAX6	-0.105870095	0.014287865
14135	TMEM56-RWDD3	-0.118037388	0.006268639
14136	MST1R	0.157286604	0.000259904
14137	PHEX	0.009279772	0.830434515
14138	CATSPERG	0.141582787	0.001024482
14139	LY9	0.228065252	9.65E-08
14140	ADAMTS18	0.047262465	0.275168639
14141	MASP2	-0.027768039	0.521589003
14142	ANKRD61	-0.04693584	0.278503521
14143	KCNS1	0.053474959	0.216878876
14144	TENM2	-0.025337675	0.558692136
14145	GNG8	0.350047907	7.24E-17
14146	CRP	0.007473815	0.86306968
14147	NEIL3	0.298290371	1.86E-12
14148	TOMM20L	-0.225930582	1.28E-07
14149	PNOC	0.202600924	2.31E-06
14150	ZNF483	-0.263252672	6.24E-10

14151	KIR2DL4	0.252927922	2.97E-09
14152	MTRNR2L9	0.152714795	0.000392829
14153	GRIA3	0.034004529	0.432503765
14154	GLDN	-0.14123655	0.001054364
14155	D4S234E	-0.018663529	0.66667453
14156	PCDHGA11	-0.094652335	0.028588783
14157	KIAA1644	-0.022803777	0.598688364
14158	TUBA3E	0.087234862	0.043708561
14159	FAM188B	0.177403596	3.68E-05
14160	DDN	-0.029927308	0.489718704
14161	CR1	-0.083804429	0.052711469
14162	HIST2H2BF	0.225878048	1.29E-07
14163	TTBK1	0.067362142	0.119657084
14164	AL049872.1	-0.043417282	0.316165671
14165	MIPOL1	-0.285383204	1.75E-11
14166	MPP2	0.113997211	0.008310089
14167	SLC7A11	-0.045509171	0.293390631
14168	RASL10A	0.447723419	9.81E-28
14169	APCS	-0.004641004	0.914712642
14170	FPR2	0.014767245	0.733263919
14171	NCMAP	-0.030092127	0.487329698
14172	MCMDC2	-0.18088534	2.57E-05
14173	DRICH1	0.310391968	2.06E-13
14174	M1AP	0.165010045	0.000126035
14175	CALCA	-0.188615709	1.12E-05
14176	PGA5	0.191073934	8.58E-06
14177	FBXL13	-0.165267771	0.000122958
14178	CFAP57	0.094686791	0.028530666
14179	KCNAB3	0.485985762	4.63E-33
14180	ZGLP1	0.69272453	1.10E-77
14181	HMCN2	-0.002005098	0.963095359
14182	OAZ3	0.363197016	3.98E-18
14183	SYNC	-0.066916757	0.122128246
14184	FAM83B	-0.29197027	5.65E-12
14185	CD160	0.038707256	0.37156686
14186	TNNC2	0.095153083	0.027754095
14187	KCNIP1	-0.187992859	1.20E-05
14188	GRIN2D	0.292551871	5.11E-12
14189	APITD1-CORT	0.153099763	0.000379567
14190	LHFPL3	0.029411129	0.497241092
14191	ADAMTS8	0.103684316	0.0164361
14192	TMEM52	0.125440783	0.003659008
14193	TMEM207	-0.097806891	0.02367176
14194	C16orf93	0.487488029	2.77E-33
14195	C16orf71	0.274499899	1.06E-10
14196	ADCYAP1	-0.178231387	3.38E-05
14197	ABCA4	-0.087510793	0.043044383
14198	AC234582.1	-0.047897032	0.268767595
14199	ALAS2	0.022451309	0.604352937
14200	GREM1	0.105292957	0.014829638
14201	GABRR2	0.142466643	0.000951712
14202	WFDC3	0.3190474	4.01E-14
14203	TXLNB	-0.006698477	0.877155936
14204	CARD14	0.182066357	2.27E-05
14205	GNAO1	0.010655709	0.805761366
14206	PSORS1C2	0.32209024	2.22E-14
14207	PF4	0.213474745	6.24E-07
14208	BEND6	-0.068118427	0.115550607

14209	HSD17B1	0.144463927	0.000804505
14210	C1orf228	0.521360864	1.28E-38
14211	SNCAIP	-0.176329913	4.11E-05
14212	C2CD4D	0.159730001	0.000207449
14213	CABP4	0.210847423	8.61E-07
14214	PPP1R36	-0.075176872	0.082345176
14215	PROZ	-0.033396195	0.440787243
14216	BMP5	-0.076145735	0.078460059
14217	GREB1	-0.010765956	0.803792403
14218	ELF5	-0.059072701	0.17245702
14219	CBWD5	0.137286876	0.001457015
14220	OXT	0.085420775	0.048293398
14221	SIX2	0.151913254	0.000421839
14222	TNFRSF8	0.312364132	1.43E-13
14223	CEND1	-0.091402898	0.034546343
14224	EML6	-0.088478795	0.040781238
14225	ATP1A3	0.155628402	0.000302307
14226	B3GALT5	0.012491787	0.773139075
14227	KCNJ4	0.185341016	1.60E-05
14228	AARD	0.064458439	0.136490118
14229	HSD17B2	0.035051974	0.418453699
14230	KRBA2	-0.254295753	2.42E-09
14231	GOLGA8N	0.184169346	1.81E-05
14232	ABCD2	-0.033438523	0.440207947
14233	MFSD6L	-0.163191008	0.0001499
14234	NOX1	0.276648135	7.44E-11
14235	C9orf117	0.259584445	1.09E-09
14236	SYNGR3	-0.045005412	0.298772211
14237	PKNOX2	0.066178918	0.126309363
14238	ADAM18	-0.134224866	0.001861651
14239	APOC2	0.226768572	1.14E-07
14240	RAET1E	-0.130295847	0.002531216
14241	C2orf70	0.256753852	1.68E-09
14242	TMPRSS4	-0.123439339	0.004244097
14243	S100A5	0.054473188	0.208407806
14244	C6orf141	0.290156594	7.73E-12
14245	NOXRED1	0.132415315	0.002146786
14246	TNN	-0.039573331	0.360952956
14247	TFAP2C	-0.080646359	0.062317525
14248	ZNF831	0.111865576	0.009610464
14249	CLUL1	-0.197927829	3.97E-06
14250	C9	0.057563517	0.183704057
14251	CDK3	0.527745779	1.07E-39
14252	GOLGA8M	0.148065048	0.000591026
14253	CLEC4F	0.238370944	2.39E-08
14254	COCH	-0.04015194	0.353968774
14255	CMTM2	0.226768568	1.14E-07
14256	ALOX12B	0.132147611	0.002192205
14257	FBXL22	-0.005552969	0.898037306
14258	ARMC12	0.527444222	1.21E-39
14259	MEOX1	0.034896174	0.420526425
14260	KCTD4	0.067698133	0.117818859
14261	BNIPL	0.34639672	1.58E-16
14262	4-Mar	-0.03197009	0.460557372
14263	COLGALT2	0.102615177	0.01758632
14264	FOXF2	0.194540802	5.83E-06
14265	TNIP3	0.098351966	0.022900836
14266	NLGN3	0.325983699	1.04E-14

14267	PCDHGA1	-0.012373131	0.775235915
14268	KLRG2	0.047873334	0.269004798
14269	KANK4	-0.076571384	0.076800908
14270	PRPH2	-0.055735222	0.19804383
14271	SLC18A2	-0.269537404	2.34E-10
14272	TYRP1	-0.058438054	0.17712244
14273	ATP8A2	-0.139077716	0.001259554
14274	CCNI2	0.104790794	0.015315633
14275	SKIDA1	-0.096283091	0.025947171
14276	C11orf65	-0.146579049	0.000671794
14277	SIGLEC15	-0.075169397	0.082375747
14278	FAM47E	-0.156641715	0.000275687
14279	C19orf57	0.349875572	7.51E-17
14280	IGSF22	-0.028178621	0.515448032
14281	CCDC154	0.537415517	2.29E-41
14282	OXGR1	-0.231828268	5.84E-08
14283	KISS1	0.216529285	4.26E-07
14284	TRABD2A	0.184664504	1.72E-05
14285	SMPD3	0.266162283	3.97E-10
14286	ANKRD7	-0.073349674	0.09009472
14287	TRIM50	-0.208557877	1.14E-06
14288	CYP4B1	-0.014042958	0.745884423
14289	GOLGA6L9	0.260937382	8.91E-10
14290	GLIPR1L1	-0.081100327	0.060853662
14291	TP53TG5	-0.08324019	0.054330988
14292	HDC	0.126682856	0.003333724
14293	NPC1L1	-0.014832895	0.73212347
14294	MBOAT4	0.158004271	0.000243335
14295	CORO6	0.447869514	9.39E-28
14296	UPK3B	0.372566652	4.64E-19
14297	CNR1	-0.075590931	0.080666186
14298	SIGLEC11	0.214301929	5.63E-07
14299	CBLN1	-0.083398334	0.053872951
14300	NR2E1	0.053658241	0.215305294
14301	CHRNA10	0.327943151	7.03E-15
14302	SOST	-0.095545588	0.027114566
14303	SULT1B1	-0.126339785	0.003420839
14304	COL26A1	0.047176745	0.276041208
14305	XK	-0.1001111	0.020559296
14306	ADAMTSL5	0.261503424	8.17E-10
14307	SMTNL1	0.145323309	0.0007479
14308	ZNF850	-0.002528388	0.95347376
14309	NCCRP1	0.177282301	3.73E-05
14310	WNT9B	-0.082851019	0.055471972
14311	CAMK2N2	0.317062134	5.86E-14
14312	KIAA0319	-0.03761133	0.385271158
14313	NEFH	0.074888451	0.083531323
14314	GGT2	0.149132348	0.000538684
14315	BTLA	0.05131446	0.236052438
14316	PACSIN1	0.141371694	0.001042606
14317	SLC7A13	-0.065837797	0.12827959
14318	COLEC10	-0.184886753	1.68E-05
14319	ACTN2	0.102374963	0.017854227
14320	PCBP3	0.237791256	2.59E-08
14321	EPHA6	-0.101025091	0.019427025
14322	3-Sep	0.073395939	0.089891517
14323	BCO2	0.106303356	0.01389267
14324	SEMA3A	0.116503627	0.006983595

14325	TFF1	0.180473106	2.68E-05
14326	CCSER1	-0.347146157	1.35E-16
14327	AC008641.1	-0.111961085	0.009548537
14328	FAM71F2	0.246881788	7.17E-09
14329	MAPK8IP2	0.266505786	3.76E-10
14330	GPLD1	-0.11565894	0.007407779
14331	BAI1	0.236731733	2.99E-08
14332	AC241585.2	0.225690167	1.32E-07
14333	DOK7	0.083872108	0.052519943
14334	OTOGL	-0.212647706	6.91E-07
14335	TTR	-0.075893863	0.079455458
14336	CIDEB	-0.102015907	0.018261325
14337	SIGLEC12	0.125878229	0.003541301
14338	KLHL31	0.017624621	0.68420134
14339	TNNT3	0.085045234	0.049291214
14340	GFAP	-0.00669316	0.87725268
14341	CA14	0.044568866	0.303488589
14342	RIPPLY1	0.152318224	0.000406941
14343	KLHL30	0.054528917	0.207942039
14344	GDF9	0.22099919	2.42E-07
14345	GPR137C	-0.226684928	1.16E-07
14346	CFAP45	0.388481942	1.02E-20
14347	ABCA9	0.064513506	0.136154767
14348	ASB14	0.006985184	0.87194231
14349	C1orf145	0.293175429	4.58E-12
14350	AS3MT	-0.167505334	9.91E-05
14351	MSX2	0.107617794	0.012751985
14352	LHX4	0.103041435	0.017119563
14353	DNAH7	-0.208909134	1.09E-06
14354	NLRP11	-0.065766494	0.128694409
14355	HCN2	-0.111883518	0.009598803
14356	DUSP26	0.033354501	0.441358266
14357	OLFM4	0.000136674	0.997483572
14358	PAK3	-0.029563527	0.495013789
14359	CD80	0.105992669	0.014175069
14360	SYS1-DBNDD2	0.008986947	0.835707892
14361	PON3	0.07460416	0.084713947
14362	TWIST2	0.209646146	9.97E-07
14363	CXorf65	0.462355237	1.08E-29
14364	SHISA6	-0.12252799	0.004537544
14365	CILP	0.166947396	0.000104571
14366	BCL2L15	-0.000388934	0.992839059
14367	STARD5	0.193752625	6.37E-06
14368	SCLY	0.288497534	1.03E-11
14369	CTB-133G6.1	0.05839262	0.177459996
14370	RAB40A	0.110458647	0.010564963
14371	SCN8A	0.167622067	9.79E-05
14372	CYP2B6	-0.041247608	0.340978466
14373	ABCA6	-0.132023529	0.002213554
14374	TAGLN3	0.127330732	0.003174686
14375	NME9	-0.016457265	0.704099062
14376	KCNMB2	-0.104913039	0.015196054
14377	PCDHGB4	-0.094525766	0.028803141
14378	KLC3	-0.079532547	0.066032486
14379	NAT2	0.022957879	0.596219338
14380	ADAMTS17	-0.004090834	0.924791135
14381	TFAP2E	0.470229897	8.72E-31
14382	EGFL6	0.09072946	0.035905263

14383	VAX2	0.231552744	6.06E-08
14384	ACR	0.185029914	1.65E-05
14385	FNDC5	-0.037821527	0.382619094
14386	NMRK2	-0.0907273	0.035909693
14387	STX16-NPEPL1	0.374036013	3.29E-19
14388	KCNQ4	0.080273519	0.063541398
14389	GPR97	0.219318407	3.00E-07
14390	NBPF19	0.180022867	2.81E-05
14391	ARSI	0.192453698	7.36E-06
14392	CCDC158	-0.226611733	1.17E-07
14393	PRSS22	-0.063622266	0.141660776
14394	DEPDC4	-0.048049913	0.267240808
14395	HPCAL4	-0.035156947	0.417060548
14396	GAL3ST3	-0.062625124	0.148021478
14397	FUT2	0.020887393	0.629770867
14398	MMP17	0.237761428	2.60E-08
14399	WDR62	0.544731207	1.14E-42
14400	IGSF23	0.144593807	0.000795703
14401	RNF112	0.364784898	2.78E-18
14402	REG3G	0.011073927	0.798298798
14403	PRR35	-0.006940378	0.872756721
14404	PLA2G5	0.115813097	0.007328687
14405	C12orf79	0.300645472	1.22E-12
14406	S100P	0.073825485	0.088022435
14407	NTRK3	-0.089693726	0.038083851
14408	TRIM34	-0.099269133	0.021652688
14409	ZNF774	-0.053884244	0.213376238
14410	PIF1	0.626893293	9.13E-60
14411	AMH	0.509258521	1.21E-36
14412	GPC5	-0.074684583	0.084378033
14413	AC104581.1	0.177952088	3.48E-05
14414	TMEM262	0.445072473	2.17E-27
14415	NPPC	0.123030326	0.004373605
14416	ZNF564	-0.219775331	2.83E-07
14417	TMEM59L	0.065575465	0.129810884
14418	CLDN9	0.300519433	1.25E-12
14419	AMPH	0.000370967	0.993169849
14420	LRRN3	-0.152150317	0.000413058
14421	CRYBB2	0.443177811	3.81E-27
14422	MAP3K15	-0.082201623	0.057420188
14423	ZYG11A	0.116301275	0.007083181
14424	WNK3	-0.342388774	3.68E-16
14425	NCR1	0.177739371	3.56E-05
14426	SYT6	-0.045140563	0.297322004
14427	TRIM29	0.004930922	0.909407061
14428	FITM1	0.423016647	1.23E-24
14429	ARHGAP20	-0.225897286	1.28E-07
14430	ZNF676	-0.227360464	1.06E-07
14431	VSTM5	0.103029671	0.017132297
14432	SMIM2	0.046829144	0.279598821
14433	AC092835.2	-0.368146569	1.29E-18
14434	CNTF	-0.081169016	0.060634665
14435	ENHO	0.1631641	0.000150283
14436	ARRDC5	0.331512143	3.44E-15
14437	RP11-434D12.1	0.280037752	4.27E-11
14438	TEX29	0.415225445	1.03E-23
14439	SCML2	0.156041882	0.000291168
14440	RNASEK-C17orf49	0.362271641	4.91E-18

14441	ANGPT4	-0.105454738	0.014675979
14442	E2F7	0.240703559	1.72E-08
14443	WNT7B	0.285588147	1.69E-11
14444	NPIPB11	0.375180648	2.52E-19
14445	CBY3	0.113119991	0.008824912
14446	SYTL5	-0.101999017	0.018280673
14447	CALB2	0.1625109	0.000159866
14448	SLITRK5	-0.053760527	0.214430677
14449	RSPH4A	0.109688072	0.011122611
14450	DHH	-0.077400401	0.07365134
14451	UCN3	0.140872451	0.001086652
14452	ARHGAP11B	0.349270355	8.55E-17
14453	MMP12	0.091224675	0.034901643
14454	UPK3BL	0.144265363	0.000818136
14455	SLC22A1	0.159372171	0.000214455
14456	ITLN1	0.056775161	0.189790364
14457	HS3ST3A1	0.223114849	1.84E-07
14458	ZNF208	-0.164966772	0.000126559
14459	HAPLN2	0.154994182	0.00032017
14460	CYP46A1	0.160706992	0.000189394
14461	CIDEC	0.189867493	9.79E-06
14462	UBQLNL	0.387093893	1.44E-20
14463	SRCIN1	0.256460938	1.75E-09
14464	PPP2R2B	0.014625465	0.735728862
14465	ITGB1BP2	0.316784779	6.18E-14
14466	FOXA3	-0.065214798	0.131939247
14467	TMEM256-PLSCR3	0.509462374	1.12E-36
14468	GNG3	0.411635991	2.70E-23
14469	MSTN	0.322844292	1.92E-14
14470	EFHB	-0.042499762	0.326510671
14471	PCDHGB3	-0.142966484	0.000912702
14472	SRRM5	0.53156051	2.39E-40
14473	KIF14	0.271496599	1.71E-10
14474	ADORA2A	0.238994315	2.19E-08
14475	MLIP	-0.213987725	5.85E-07
14476	TNFRSF13C	0.283460453	2.41E-11
14477	AL358852.1	0.119156697	0.005789105
14478	CFAP58	0.097817402	0.023656684
14479	SLC14A2	-0.142936993	0.000914962
14480	CAPN14	-0.140956993	0.001079075
14481	CTB-102L5.4	0.198678443	3.64E-06
14482	PRRG3	-0.107511376	0.012841159
14483	IGSF11	-0.024091091	0.578206812
14484	SLC18A3	0.243769881	1.12E-08
14485	NFE2	0.215569264	4.81E-07
14486	TDRD6	-0.167736806	9.69E-05
14487	SLC1A7	0.098616592	0.022534471
14488	SHBG	0.214905967	5.22E-07
14489	SLC5A11	0.084258494	0.051437577
14490	C1orf101	0.104026049	0.016082619
14491	TET1	-0.233490821	4.66E-08
14492	C2orf73	-0.162756306	0.000156201
14493	TBX18	0.083699034	0.053010889
14494	ANKRD35	0.179928632	2.84E-05
14495	DNAAF1	0.215870587	4.63E-07
14496	KIAA1211	0.022998358	0.595571551
14497	LTK	0.208976524	1.08E-06
14498	TAS2R4	0.045395918	0.294594809

14499	ANKRD36C	-0.039818078	0.357988246
14500	DLEU7	-0.161535751	0.000175245
14501	ADD2	0.163818074	0.000141231
14502	NR1I3	0.020991697	0.628061522
14503	CA10	-0.187625963	1.25E-05
14504	COL11A2	0.402537231	2.94E-22
14505	B3GALT2	-0.163199072	0.000149786
14506	DAPL1	-0.066241502	0.125950459
14507	RTEL1	0.076495914	0.077092987
14508	AKAP2	-0.372322479	4.91E-19
14509	KCNK17	0.051854155	0.231154421
14510	REG1B	-0.033737057	0.43613472
14511	SLC6A17	-0.093095783	0.031322406
14512	GYPE	-0.119128931	0.00580059
14513	HTR1B	-0.085171366	0.048954172
14514	GP6	0.127039317	0.003245348
14515	AIFM3	0.434476941	4.83E-26
14516	PITX2	0.097434952	0.024210609
14517	HR	0.111861927	0.009612836
14518	CCL24	0.053962437	0.212711721
14519	NME8	0.32900505	5.69E-15
14520	C6orf163	0.04743614	0.273406502
14521	FAM198A	-0.026128918	0.546473436
14522	CNIH2	0.518399584	3.95E-38
14523	PRSS27	0.438161647	1.66E-26
14524	SYNJ2BP-COX16	-0.153355172	0.000370999
14525	PKD2L1	0.068705912	0.112437591
14526	KLRK1	0.326312345	9.72E-15
14527	KRT81	0.028318343	0.513366819
14528	TPRG1	-0.101513785	0.018844211
14529	UGT3A2	0.142354019	0.000960711
14530	TMEM35	-0.146391077	0.00068271
14531	CAMK2B	-0.023641541	0.585321902
14532	ECEL1	-0.014158736	0.743862359
14533	BACH2	-0.159062185	0.000220704
14534	KCNK7	0.460245304	2.10E-29
14535	EFCAB10	0.193423944	6.60E-06
14536	SMIM17	-0.014523472	0.73750377
14537	MAPK4	-0.184547334	1.74E-05
14538	ADCY2	-0.032636186	0.45126232
14539	CHRNA1	0.018130789	0.675640149
14540	CNTNAP5	-0.198855542	3.57E-06
14541	ANKK1	0.423607582	1.04E-24
14542	HBD	-0.02688042	0.534991985
14543	C10orf55	0.231778695	5.88E-08
14544	CACNA1F	0.293451839	4.37E-12
14545	PLAG1	-0.123944746	0.004088863
14546	BMPER	0.162214175	0.000164406
14547	FAM53A	0.304470585	6.13E-13
14548	EDAR	-0.112251479	0.009362425
14549	GSG2	0.337332441	1.05E-15
14550	CASP5	0.193941118	6.23E-06
14551	ZNF280B	-0.016738577	0.699284938
14552	ZNF724P	-0.328209043	6.67E-15
14553	RHD	0.14090896	0.001083374
14554	COL4A6	-0.039582807	0.360837889
14555	ARHGAP8	-0.173039528	5.74E-05
14556	STRA6	0.199662923	3.25E-06

14557	CCDC171	-0.284476319	2.03E-11
14558	POU5F1B	0.028529365	0.510231857
14559	SPDYE6	0.232631137	5.24E-08
14560	PIK3C2G	-0.185106419	1.64E-05
14561	C16orf95	0.405042704	1.54E-22
14562	PSCA	-0.128854857	0.002827386
14563	ASIC1	0.111540796	0.009823724
14564	TRIM17	0.017760854	0.681893095
14565	RALYL	-0.023112059	0.593753707
14566	MSH5-SAPCD1	0.505916278	4.11E-36
14567	LRRC15	0.040892441	0.345155591
14568	RGS11	0.332998111	2.55E-15
14569	MEF2B	0.506468085	3.36E-36
14570	CNTD2	0.249912238	4.62E-09
14571	RAD9B	-0.079519061	0.066078555
14572	DLX4	0.221264765	2.34E-07
14573	FGF14	-0.146035009	0.000703839
14574	FAM3D	0.006739898	0.876402376
14575	DNASE1L2	0.52785738	1.03E-39
14576	CTAGE9	0.083519395	0.053524494
14577	RP4-583P15.15	0.528708815	7.37E-40
14578	ZNF365	0.032374002	0.454908283
14579	STKLD1	0.405414775	1.39E-22
14580	WISP3	-0.104229174	0.015875683
14581	IGSF9B	0.00229322	0.957796965
14582	SGOL1	0.272158942	1.54E-10
14583	DNAI1	0.182090629	2.26E-05
14584	SPINK2	0.102382974	0.017845235
14585	PANO1	0.598271765	3.03E-53
14586	FSCN2	0.121500578	0.004890245
14587	AMZ1	0.108249735	0.012233614
14588	GLT1D1	0.048229189	0.265458015
14589	HOXA13	0.028861061	0.505324391
14590	ALX1	0.134752247	0.001785336
14591	FAM155A	0.014352314	0.740485428
14592	HIST1H4K	0.026889603	0.534852437
14593	SLC9B1	-0.04516973	0.297009655
14594	ACKR2	-0.202127624	2.44E-06
14595	TCTEX1D4	0.190813275	8.83E-06
14596	LGR6	0.17663059	3.99E-05
14597	ANKRD36B	-0.043090716	0.319822817
14598	MCEMP1	0.213887212	5.93E-07
14599	SCML4	0.226821201	1.14E-07
14600	FSTL4	-0.089709943	0.038048895
14601	AUNIP	0.231989167	5.71E-08
14602	GATA5	0.014062351	0.745545608
14603	SAMD13	-0.150945846	0.000459507
14604	BLOC1S5-TXNDC5	0.01112563	0.797377477
14605	GIPR	0.366226174	2.00E-18
14606	HES5	0.196500145	4.67E-06
14607	CDH17	0.013669139	0.75242507
14608	RGMA	0.075824034	0.079733231
14609	HHIP	-0.024040272	0.579009089
14610	RET	0.085276892	0.048673679
14611	ATOH7	0.326753508	8.90E-15
14612	ZBTB32	0.395496446	1.78E-21
14613	C9orf92	0.154276042	0.000341584
14614	ZFP57	0.05348445	0.216797184

14615	COL17A1	-0.093183254	0.031163064
14616	DNAH14	0.120893534	0.00511008
14617	TACR2	-0.037427282	0.387602478
14618	HIST1H4C	-0.08729507	0.043562907
14619	ZFHX4	0.101624024	0.018714869
14620	NUDT11	0.108576474	0.011972968
14621	C2orf16	-0.249915076	4.62E-09
14622	CECR6	0.143745516	0.00085484
14623	TMEM200C	0.067060516	0.121326308
14624	FAM169B	0.020632192	0.633961485
14625	RBP2	-0.132969678	0.002055454
14626	GPR15	-0.018997224	0.661082675
14627	HS6ST3	-0.238757405	2.26E-08
14628	CR1L	-0.131421265	0.0023199
14629	FLRT1	-0.043148647	0.319172054
14630	SHC3	0.141203592	0.00105725
14631	RAB3C	-0.20824857	1.18E-06
14632	UNC93A	0.026649265	0.538510616
14633	GPR113	-0.003317851	0.938971334
14634	HPX	0.233572465	4.61E-08
14635	RNF212B	-0.153586329	0.000363401
14636	PHACTR3	-0.058104284	0.179613362
14637	CD177	0.051813762	0.231518494
14638	CCDC13	-0.042476181	0.3267794
14639	DDIAS	0.204230479	1.91E-06
14640	TRIM63	0.008605656	0.842585426
14641	AL158839.1	0.112126017	0.009442433
14642	TNNI1	-0.013575043	0.75407426
14643	PRPH	0.194448873	5.89E-06
14644	UGT2B11	0.013999209	0.746648962
14645	ZNF286B	-0.057263656	0.186001846
14646	C12orf56	0.059198566	0.171542748
14647	PMCH	0.192635546	7.21E-06
14648	AC108938.5	-0.155714648	0.000299952
14649	RBM11	-0.227985629	9.75E-08
14650	PRSS3	0.215711901	4.72E-07
14651	GDPD2	-0.191291433	8.37E-06
14652	PPM1E	-0.224806496	1.48E-07
14653	DNAH6	-0.088244641	0.041319249
14654	GGN	0.258530906	1.28E-09
14655	LINGO4	-0.028586269	0.509388188
14656	GJC3	0.057241	0.186176315
14657	PCDHAC2	-0.265889794	4.14E-10
14658	PADI3	0.119211806	0.005766371
14659	H3F3C	0.126577358	0.003360296
14660	TEX22	0.35442515	2.80E-17
14661	BHLHE22	0.038964517	0.368394114
14662	APOA1	0.088883951	0.039864303
14663	HIST1H4D	-0.042625804	0.325076704
14664	LMO3	-0.004088358	0.924836521
14665	CTD-2192J16.20	-0.22715531	1.09E-07
14666	ITIH2	0.018720484	0.665718794
14667	BCAS1	-0.0280416	0.517493255
14668	EYA1	0.064988303	0.133289556
14669	DKK2	-0.059700125	0.167935406
14670	FZD10	0.067217644	0.120454493
14671	DLX1	0.085934611	0.046955591
14672	WFDC5	0.17614712	4.19E-05

14673	CYP11A1	-0.119729722	0.005556604
14674	PSD2	0.098541034	0.022638556
14675	RCOR2	0.124862905	0.003819941
14676	DLG2	-0.251044098	3.92E-09
14677	C7orf25	-0.06068167	0.161040697
14678	TFF2	0.01203986	0.781134055
14679	ITGA2B	0.237265964	2.78E-08
14680	PCDHA10	-0.178501256	3.29E-05
14681	MEF2BNB-MEF2B	0.374611115	2.88E-19
14682	TNS4	-0.025877828	0.550336529
14683	NALCN	-0.010004222	0.817421339
14684	LAT	0.505646859	4.53E-36
14685	AGR3	0.042727338	0.323924526
14686	ABCA10	0.143538887	0.000869847
14687	HIST1H2BL	-0.020121969	0.642374871
14688	NYX	0.042031836	0.331870016
14689	TAC4	0.117512354	0.006505623
14690	TUBAL3	-0.019739638	0.648709559
14691	MLC1	0.083352218	0.054006186
14692	FAIM2	0.064513273	0.136156186
14693	TCTEX1D1	0.001378696	0.974619784
14694	FAM154B	0.144941156	0.0007726
14695	ATP12A	-0.095949254	0.026470122
14696	PITX1	0.271921657	1.60E-10
14697	SLC38A3	-0.112371761	0.009286287
14698	AFF2	-0.259531811	1.10E-09
14699	NPAS3	-0.073537054	0.089274005
14700	DCST2	0.495712847	1.59E-34
14701	LRRC4C	-0.029371224	0.497825177
14702	CORT	0.459790399	2.42E-29
14703	RP11-314N13.10	-0.192330592	7.46E-06
14704	GREB1L	0.064606173	0.135591867
14705	LRRC31	-0.200914052	2.81E-06
14706	FAM227A	0.283890199	2.24E-11
14707	KLK7	-0.021957007	0.612337083
14708	NTSR1	-0.135946254	0.001623036
14709	LYPD6	0.00018707	0.996555682
14710	FKSG48	0.299742333	1.44E-12
14711	SAA4	0.121977865	0.004723431
14712	CNTNAP3	0.023915752	0.580977103
14713	C11orf85	0.089321985	0.038892647
14714	CNDP1	-0.03575097	0.409228528
14715	DNASE2B	-0.067367537	0.11962739
14716	CR2	0.050754223	0.241213847
14717	IGSF1	0.045800846	0.290304537
14718	TMEM31	0.165626358	0.000118795
14719	VSIG8	0.096939725	0.024944508
14720	POLN	0.213466148	6.24E-07
14721	APC2	-0.01024268	0.813148715
14722	RTKN2	0.129203871	0.002752891
14723	SYT1	0.066826859	0.122631826
14724	DMC1	0.052207737	0.227984777
14725	CFAP46	-0.0851752	0.048943958
14726	VGLL1	-0.124104361	0.004040916
14727	C7orf61	0.469115342	1.25E-30
14728	SPIB	0.252515222	3.16E-09
14729	FFAR2	0.190880747	8.76E-06
14730	PCDHGA3	-0.051813133	0.231524169

14731	CH17-140K24.5	-0.156814398	0.000271376
14732	CARNS1	0.256886211	1.65E-09
14733	ZSCAN23	-0.282061264	3.05E-11
14734	WNT4	0.114058757	0.008274996
14735	PIP	-0.132407633	0.002148077
14736	ACSL6	-0.096327894	0.02587767
14737	XKR6	-0.101998819	0.0182809
14738	CST2	0.163365481	0.000147439
14739	CATSPER1	0.415796735	8.85E-24
14740	FAM182B	0.111186364	0.010061245
14741	LEKR1	0.036042613	0.405415443
14742	HIST1H1D	-0.014455877	0.738680839
14743	ROBO2	-0.261540997	8.12E-10
14744	TCP10L	0.230964794	6.56E-08
14745	GPRC5D	0.132111941	0.002198323
14746	ZNF519	-0.030125118	0.48685225
14747	PPEF1	0.182310594	2.21E-05
14748	NAALAD2	-0.049211975	0.255830001
14749	AICDA	0.112566921	0.009163923
14750	AC004754.3	0.286212701	1.52E-11
14751	MROH2A	0.152695089	0.000393519
14752	GRIP2	0.026772983	0.536625942
14753	FOXA2	0.132991989	0.002051854
14754	CD19	0.265592106	4.34E-10
14755	ASB12	0.074152013	0.086622625
14756	SPTBN4	-0.012852806	0.766769493
14757	CACNA1E	0.097509194	0.024102211
14758	COL9A3	0.058182289	0.179028898
14759	ELAVL2	-0.237160651	2.82E-08
14760	PCSK1	0.053918069	0.213088598
14761	SNX15	0.227463131	1.04E-07
14762	PPT2-EGFL8	0.406048741	1.18E-22
14763	ELMOD1	-0.015840429	0.714696297
14764	ANKRD23	0.503540252	9.73E-36
14765	GRIK4	0.094244133	0.029285072
14766	CHKB-CPT1B	0.426227024	5.02E-25
14767	TRAPPC5	0.285911998	1.60E-11
14768	ENO4	-0.188635249	1.12E-05
14769	MYBPC2	0.148245814	0.000581842
14770	GNG4	0.140595097	0.001111857
14771	C11orf94	0.194276158	6.00E-06
14772	SPDYA	0.243887539	1.10E-08
14773	CKLF-CMTM1	0.120102596	0.005409861
14774	NEURL1	-0.150914476	0.000460779
14775	TICRR	0.3653051	2.47E-18
14776	CCR8	0.131566458	0.002293844
14777	RHCE	0.034288272	0.428671084
14778	GRIN3B	0.338898535	7.62E-16
14779	QRFP	0.082938228	0.055214573
14780	HEATR4	0.105434552	0.014695074
14781	KRT5	0.220647906	2.53E-07
14782	ERCC6L	0.173890869	5.26E-05
14783	GPR182	-0.108143958	0.012319062
14784	LRRC7	-0.053080514	0.220293369
14785	CCDC136	0.109672322	0.011134276
14786	KLK4	-0.172467898	6.07E-05
14787	SPTB	-0.073991242	0.087309587
14788	SNX32	0.204409896	1.87E-06

14789	PLSCR2	0.045636602	0.292039648
14790	AHSP	0.021786215	0.615106548
14791	ADRB1	-0.1762202	4.16E-05
14792	TSHR	0.079313799	0.066783054
14793	LDHC	-0.030340118	0.483746973
14794	GJB5	0.03606268	0.405153867
14795	PIANP	0.159088727	0.000220162
14796	MUC4	0.071089451	0.100478884
14797	DNAH17	0.322894032	1.90E-14
14798	PI15	0.12900268	0.002795612
14799	KCNH3	0.305862865	4.76E-13
14800	C4BPA	-0.006921453	0.873100756
14801	COL28A1	0.062670406	0.147727989
14802	IGFALS	0.297123175	2.29E-12
14803	CELF4	0.093175778	0.031176654
14804	B3GAT1	0.205832809	1.58E-06
14805	SDK2	0.007208503	0.867885192
14806	TTC23L	0.027038011	0.532599779
14807	NACA2	0.132185205	0.002185774
14808	MAK	-0.047161888	0.276192626
14809	TRPM6	-0.061652499	0.154433178
14810	PIK3R2	0.545393498	8.68E-43
14811	ISM2	-0.045253011	0.296118986
14812	NXPH2	-0.190996675	8.65E-06
14813	KLB	-0.014662376	0.735086875
14814	HPDL	0.26750884	3.22E-10
14815	MLANA	-0.102608356	0.017593878
14816	ARR3	0.17564205	4.41E-05
14817	ADAM11	0.420806201	2.26E-24
14818	BOLA2B	0.401886293	3.48E-22
14819	PLXNA4	0.049864921	0.24956869
14820	SOX8	0.027557717	0.524749201
14821	ETNPPL	-0.04400696	0.309631579
14822	SLC13A4	0.187561438	1.26E-05
14823	C19orf81	0.223070216	1.85E-07
14824	XG	0.023484866	0.587811104
14825	RP11-812E19.9	0.127068075	0.003238312
14826	AP3B2	0.078505542	0.069617346
14827	HPSE2	0.063114742	0.144871564
14828	PPP2R2C	0.243127882	1.23E-08
14829	CRHR2	0.062995099	0.145636511
14830	KIAA1024	0.162180079	0.000164936
14831	EIF2S3L	-0.170234536	7.58E-05
14832	ANKRD55	0.009000238	0.83546837
14833	LRRC69	0.115943845	0.007262197
14834	KLRC1	0.136772955	0.00151872
14835	ZP1	0.133138006	0.002028434
14836	SPDEF	0.198735298	3.62E-06
14837	FAM209B	0.426730906	4.36E-25
14838	UPK2	0.207581879	1.28E-06
14839	NANOS3	0.266597625	3.71E-10
14840	IGF2BP3	0.159463477	0.000212647
14841	SERPINC1	0.021493973	0.619858074
14842	STEAP1B	0.114250475	0.008166525
14843	ST8SIA1	0.179229122	3.05E-05
14844	SCT	0.167916848	9.52E-05
14845	RP11-544M22.13	-0.00143201	0.973638686
14846	NWD1	-0.149633316	0.00051563

14847	ZNF726	0.005083354	0.90661911
14848	RGS17	0.200365904	3.00E-06
14849	HECW1	-0.051979297	0.230029049
14850	MYOZ3	0.129371692	0.002717711
14851	AC007326.1	0.185763071	1.53E-05
14852	DTHD1	0.100910575	0.019565846
14853	SULT4A1	0.017378507	0.688378793
14854	ANGPTL6	0.410688348	3.48E-23
14855	AC006994.3	0.060033702	0.165567874
14856	TMEM221	0.299144437	1.60E-12
14857	CCDC81	0.104162868	0.015942975
14858	DCSTAMP	0.035497267	0.412562801
14859	GPR19	0.418845473	3.86E-24
14860	SPEG	0.290248407	7.61E-12
14861	ZG16B	0.062669909	0.147731206
14862	PTCRA	0.231098222	6.44E-08
14863	TBC1D3L	0.428823957	2.42E-25
14864	HSF2BP	0.099993019	0.020709683
14865	RANBP17	0.041112307	0.342565928
14866	TG	0.03881314	0.370258979
14867	VWA2	-0.068232653	0.1149401
14868	STMN2	0.077674859	0.072632139
14869	CMA1	-0.117889604	0.006334548
14870	GJB3	0.152807306	0.000389603
14871	PRR19	0.344902437	2.17E-16
14872	PLEKHG4B	0.163420685	0.000146669
14873	TRIM71	0.01480043	0.732687358
14874	NAV3	0.178102716	3.43E-05
14875	IGFN1	0.119263044	0.005745306
14876	RUFY4	0.440049869	9.57E-27
14877	CYP3A4	-0.069816998	0.106730711
14878	VASH2	0.127572791	0.003117058
14879	KCNMB3	0.20741618	1.30E-06
14880	HHATL	-0.093448845	0.03068349
14881	ARHGAP40	0.010219415	0.813565339
14882	RASGEF1C	0.12860416	0.002882023
14883	LSAMP	-0.216282949	4.40E-07
14884	TSPO2	-0.005957078	0.890662021
14885	TIGD3	0.185440903	1.58E-05
14886	CDH9	-0.169963159	7.79E-05
14887	CCDC42B	0.366957615	1.70E-18
14888	PDZRN4	-0.195458865	5.25E-06
14889	RSPO4	0.271336738	1.75E-10
14890	PRCD	0.301831215	9.89E-13
14891	CHRNA6	-0.065932485	0.127730322
14892	APOBEC2	-0.018050126	0.677001696
14893	AC009336.19	0.114217465	0.00818511
14894	SCARA5	0.024341188	0.574266131
14895	CDH22	0.023982284	0.579925186
14896	SCRG1	0.004465452	0.917927152
14897	NKD1	-0.10636562	0.013836674
14898	PRDM6	0.134439245	0.001830276
14899	GAP43	0.048828534	0.25955725
14900	ALS2CR12	0.040406655	0.35092138
14901	TMPRSS5	0.239760378	1.97E-08
14902	GABRA2	-0.188057145	1.19E-05
14903	DRC7	0.194277278	6.00E-06
14904	TUBB3	0.255337058	2.08E-09

14905	CALML3	0.078044769	0.071276716
14906	FRMPD1	-0.011886346	0.783855149
14907	CLEC17A	0.078506036	0.069615584
14908	IGF1	-0.173989213	5.21E-05
14909	RP11-795F19.5	0.325626469	1.11E-14
14910	ESCO2	0.102761585	0.017424762
14911	FLRT2	-0.030978061	0.47459649
14912	HIST1H4J	0.272267277	1.51E-10
14913	TCN1	0.075702195	0.080219775
14914	DCDC2B	0.129439555	0.002703602
14915	TECTA	0.038694707	0.371722062
14916	PIP5KL1	0.283633531	2.34E-11
14917	AC241377.2	0.033796677	0.435323858
14918	AC069499.1	0.037089943	0.391897725
14919	TMPRSS13	0.066437144	0.124833605
14920	CLDN20	-0.141311352	0.001047841
14921	SV2B	-0.045264339	0.295997976
14922	DHRS2	-0.088333124	0.041115244
14923	KCNRG	-0.028146627	0.515925221
14924	TP73	0.180758517	2.60E-05
14925	FBN2	0.100348315	0.020260047
14926	FCRLA	0.167131539	0.000102721
14927	MKRN2OS	-0.052848325	0.222321186
14928	SIX4	-0.048521922	0.262564544
14929	C11orf42	-0.029481083	0.49621806
14930	GRHL3	-0.222821981	1.91E-07
14931	CDRT4	0.218960841	3.14E-07
14932	TNNT2	0.148558275	0.000566277
14933	DCDC1	-0.083249002	0.054305383
14934	LAMB4	-0.133278527	0.002006127
14935	SLC4A8	0.065923111	0.127784619
14936	TAS2R19	0.018871938	0.663179944
14937	GPR55	0.125697947	0.003589388
14938	LIPM	0.052261749	0.227503319
14939	WDR63	-0.190979165	8.67E-06
14940	RGS20	0.253453031	2.75E-09
14941	POLQ	0.315029645	8.63E-14
14942	CLDN18	0.030168468	0.486225278
14943	ORM1	0.030438368	0.482331512
14944	AL445183.1	0.035259708	0.415699407
14945	GSC	0.221219531	2.35E-07
14946	GMNC	-0.161361464	0.000178135
14947	C9orf47	0.04262687	0.325064588
14948	HSD3B2	0.1161689	0.007149014
14949	ZNF23	0.104876691	0.015231523
14950	SSTR5	0.028980109	0.503569149
14951	GTSF1	0.136553925	0.001545741
14952	PCDHA3	-0.105607064	0.01453258
14953	PRTG	-0.151069418	0.000454527
14954	CYP2E1	0.252853964	3.00E-09
14955	KCNV1	-0.066307473	0.125572989
14956	KRTAP5-8	-0.165076183	0.000125239
14957	MB	-0.063962285	0.139540376
14958	SLC25A21	-0.163451285	0.000146243
14959	OMG	0.008466185	0.845104152
14960	LRCOL1	0.250547754	4.21E-09
14961	HIST1H2BO	0.08996642	0.03749966
14962	UGT1A10	0.134378797	0.001839073

14963	NTNG1	-0.018646974	0.666952429
14964	HNRNPUL2-BSCL2	-0.082944974	0.055194704
14965	PPP1R1B	-0.112557776	0.009169625
14966	OVOL2	-0.063360937	0.143307171
14967	GALNT4	-0.410138801	4.02E-23
14968	GNB3	0.377452403	1.47E-19
14969	TNFSF11	-0.009014994	0.83520247
14970	CCNA1	0.255939062	1.90E-09
14971	CBWD7	0.322446548	2.07E-14
14972	TAS1R3	0.219772407	2.83E-07
14973	RYR2	0.080424545	0.063043279
14974	LRRN1	-0.051489586	0.23445512
14975	ZSCAN1	-0.028269181	0.514098612
14976	TAS2R31	-0.081521027	0.059522554
14977	NAIP	-0.103808166	0.016307214
14978	IL17D	0.149665836	0.000514166
14979	GPR115	0.130388469	0.002513184
14980	FLG	-0.119702357	0.005567513
14981	C9orf84	-0.197276469	4.27E-06
14982	LINGO3	0.171891189	6.43E-05
14983	CKMT1A	-0.09851261	0.022677819
14984	CKMT1B	-0.119626717	0.005597768
14985	ADRA1A	-0.137570212	0.001423988
14986	TMPRSS6	0.126239281	0.003446748
14987	AGRP	0.151212136	0.000448838
14988	GPR133	0.016590737	0.701813451
14989	GCM1	-0.022331906	0.606277305
14990	CSNK2A3	-0.297023779	2.33E-12
14991	ESPNL	0.152744205	0.000391801
14992	SERF1B	-0.053454072	0.217058725
14993	GP2	0.058041099	0.180087819
14994	RTP2	-0.10160437	0.018737872
14995	ARX	0.041607784	0.336775538
14996	HOXD13	0.170189618	7.62E-05
14997	SFTPB	-0.189896372	9.76E-06
14998	CPEB1	0.05046336	0.243924566
14999	TGFBR3L	0.271185107	1.80E-10
15000	GRPR	-0.008063647	0.852382437
15001	TSSK3	0.139096848	0.001257584
15002	STYK1	0.219160375	3.06E-07
15003	HPR	0.012687484	0.769684397
15004	AC069063.1	-0.067264961	0.12019292
15005	ENTPD3	-0.19523323	5.39E-06
15006	AC005042.1	-0.116824869	0.006828072
15007	PGAM4	-0.189827097	9.84E-06
15008	TRIM58	-0.141947651	0.000993841
15009	AK5	-0.039477484	0.362118153
15010	SYN2	-0.035532414	0.412099919
15011	WBP2NL	0.084529229	0.050690325
15012	AC006116.20	-0.11656102	0.006955578
15013	SCN2A	-0.13973775	0.001193219
15014	CTD-2616J11.11	0.342864736	3.33E-16
15015	GSG1	0.120420122	0.00528766
15016	GLP2R	-0.15632998	0.000283634
15017	DLGAP3	0.322083998	2.23E-14
15018	CELF5	-0.037931517	0.381235802
15019	KRTAP5-9	0.14059882	0.001111515
15020	PRH1	-0.038248262	0.377269378

15021	SPDYE5	0.108019834	0.012420003
15022	WNK2	0.077805553	0.072150867
15023	IMPG1	-0.123466572	0.004235599
15024	RPRM	0.017256287	0.690456848
15025	HYDIN	-0.187676888	1.24E-05
15026	ULBP1	0.069102134	0.11037556
15027	NOS1	-0.035342037	0.414610791
15028	TM4SF19	0.159107673	0.000219776
15029	ZCCHC18	0.367205897	1.60E-18
15030	LRIT3	0.068928436	0.111275829
15031	C1orf195	-0.103799933	0.016315754
15032	GPR98	-0.171394997	6.76E-05
15033	KRT14	0.20489655	1.76E-06
15034	FANCB	0.004994538	0.908243386
15035	FUT7	0.323906798	1.56E-14
15036	SPATC1	0.356212407	1.89E-17
15037	DIO3	-0.001717048	0.96839403
15038	FAM163B	0.073482356	0.089512953
15039	PRSS50	0.348604872	9.87E-17
15040	C11orf52	0.026763138	0.536775792
15041	SRMS	0.173761387	5.33E-05
15042	ARL9	0.249520713	4.89E-09
15043	LMX1B	0.029055334	0.502461676
15044	RP11-1220K2.2	-0.045253618	0.296112501
15045	AC004076.7	0.148357108	0.000576253
15046	ZNF804A	0.005605189	0.897083732
15047	CELSR3	0.33261575	2.76E-15
15048	OSBPL6	0.08029742	0.063462352
15049	NRG4	-0.018791982	0.664519783
15050	FAT3	-0.296779525	2.44E-12
15051	DPP6	-0.005046353	0.907295749
15052	SLC52A1	0.186020972	1.49E-05
15053	NLRP12	0.080134543	0.064002631
15054	ABHD12B	-0.115317947	0.007585448
15055	AKR1C4	-0.058382234	0.177537226
15056	GYPA	-0.052048908	0.22940474
15057	PIWIL2	0.161776368	0.000171326
15058	ACTA1	0.146955871	0.000650397
15059	WSCD2	-0.147553485	0.000617754
15060	CFAP52	0.169415308	8.22E-05
15061	NEU4	0.084105396	0.051864195
15062	MYLPF	0.308267533	3.06E-13
15063	DCT	0.013255743	0.759678863
15064	C19orf26	0.15886842	0.000224695
15065	NOS1AP	-0.175416621	4.51E-05
15066	TTC16	0.416645284	7.03E-24
15067	ESYT3	-0.134075076	0.001883865
15068	MKX	-0.014897673	0.730998736
15069	MESP2	0.081551504	0.059427066
15070	AC008759.1	0.064821608	0.134290143
15071	DCLK3	-0.045802681	0.290285197
15072	TSACC	0.384458456	2.74E-20
15073	SOAT2	0.237072319	2.86E-08
15074	ERVV-1	-0.033010284	0.44608872
15075	RP11-894J14.5	-0.063183431	0.144433786
15076	TMEM108	0.070316839	0.104239185
15077	ADAMTS6	0.240451245	1.79E-08
15078	FBXO24	0.269352698	2.40E-10

15079	CST5	0.001354642	0.975062435
15080	KIAA1875	0.475626536	1.50E-31
15081	MYH15	0.188209467	1.17E-05
15082	CDH26	0.119330859	0.005717532
15083	CACNG4	0.139244979	0.00124243
15084	PRTN3	0.112352868	0.00929821
15085	C6orf195	-0.088061285	0.041744717
15086	ABHD14A-ACY1	0.298345496	1.85E-12
15087	SLC45A2	0.132165707	0.002189108
15088	KIAA1549L	0.095475226	0.027228268
15089	GBP7	0.011526989	0.790235164
15090	SHC4	0.00280916	0.948313965
15091	RDM1	0.378967254	1.03E-19
15092	PCDHA11	-0.138521594	0.001318064
15093	EMR3	0.03480774	0.421705589
15094	ADAM33	0.249036263	5.25E-09
15095	SLFNL1	0.162276995	0.000163435
15096	CILP2	0.179286907	3.03E-05
15097	PMFBP1	0.290908827	6.79E-12
15098	DCHS2	-0.112508702	0.009200275
15099	GRID2IP	0.015539512	0.719886187
15100	LRRC55	-0.230626152	6.86E-08
15101	SCGB2B2	-0.00430307	0.920901702
15102	KRT23	-0.108308788	0.012186138
15103	RASA4	0.100507417	0.020061469
15104	S100Z	0.177444313	3.67E-05
15105	SGCG	0.040926619	0.344752218
15106	KSR2	-0.059228041	0.171329174
15107	TPH1	0.201010411	2.78E-06
15108	CACNB4	-0.132376526	0.002153313
15109	HOXB13	0.151033858	0.000455955
15110	SIGLEC5	0.070234297	0.104647419
15111	NOL4	-0.039561859	0.361092294
15112	KIAA2022	-0.220265793	2.66E-07
15113	DRD1	0.035992836	0.406064751
15114	CHRNA5	0.157073673	0.00026502
15115	P2RX5	0.437197532	2.20E-26
15116	ATP2C2	-0.004364704	0.919772548
15117	PWP2	0.035250142	0.415826006
15118	CARD17	0.342850697	3.34E-16
15119	APOA2	0.038882116	0.369408509
15120	RP5-966M1.6	0.334530335	1.87E-15
15121	NAP1L6	-0.050135209	0.247008321
15122	SYNDIG1L	0.052828505	0.222494897
15123	C1QTNF4	0.32302417	1.85E-14
15124	RP11-407P15.2	0.096846925	0.02508415
15125	AC087392.1	0.142615205	0.00093996
15126	C16orf96	-0.02952242	0.49561405
15127	FCAR	0.077654826	0.072706142
15128	PYY	-0.068105544	0.115619619
15129	MLNR	-0.135660236	0.00166063
15130	CD1A	0.026512774	0.540593692
15131	PTGDR2	0.072254813	0.095011921
15132	CNTNAP3B	-0.02837775	0.512483255
15133	PCP4L1	-0.017019261	0.694493548
15134	C11orf91	0.190019426	9.63E-06
15135	GPR63	-0.138120656	0.001361787
15136	TCHH	0.120080682	0.005418387

15137	ZNF20	0.060412499	0.162909866
15138	FAM19A2	-0.104096423	0.016010659
15139	CNTN6	-0.078085194	0.071129854
15140	C7orf57	-0.08911991	0.039338373
15141	GPR88	0.001691914	0.968856443
15142	MTRNR2L10	0.198804314	3.59E-06
15143	LRRIQ3	-0.356586841	1.74E-17
15144	ELOVL3	0.140458113	0.001124504
15145	POU3F1	0.133045516	0.00204324
15146	TMC5	-0.037928835	0.381269494
15147	RP11-437B10.1	-0.017620064	0.684278608
15148	C10orf131	-0.151569001	0.000434901
15149	FER1L6	-0.143734833	0.00085561
15150	GRIN3A	-0.088512058	0.040705293
15151	PVRIG	0.0018981	0.965063408
15152	MURC	0.135980481	0.001618589
15153	RAB3B	0.052479058	0.225573575
15154	PKD1L1	-0.062071447	0.151646071
15155	ZNF878	-0.012348237	0.775676044
15156	CKM	0.215344611	4.95E-07
15157	ODF3L2	0.237349464	2.75E-08
15158	FCRL5	0.175693609	4.39E-05
15159	CACNA1A	0.287163264	1.29E-11
15160	AQP5	-0.137700884	0.00140899
15161	PDE6C	0.308479678	2.94E-13
15162	MPL	-0.199397546	3.35E-06
15163	MADCAM1	0.400155273	5.44E-22
15164	RP11-33O4.2	0.323116591	1.82E-14
15165	BCL2L2-PABPN1	0.412375414	2.22E-23
15166	UPK3A	0.135374746	0.001698951
15167	FAM153B	0.017061818	0.693768136
15168	RPGRIP1	0.142628266	0.000938933
15169	BEAN1	0.107689658	0.012692075
15170	IL12A	0.283603529	2.36E-11
15171	FAM186B	-0.00981774	0.820766441
15172	TSLP	-0.071602328	0.098042825
15173	INHBC	0.133869407	0.001914762
15174	LRRC71	0.348171497	1.08E-16
15175	RNASE3	0.08360175	0.05328852
15176	ANKLE1	0.593987754	2.52E-52
15177	HMGCLL1	-0.12391713	0.00409721
15178	AFP	0.075689718	0.080269733
15179	C9orf135	-0.048225264	0.26549696
15180	C2orf50	-0.107296458	0.013022932
15181	EEF1G	0.287158049	1.29E-11
15182	C1orf189	0.121715129	0.004814612
15183	MTRNR2L3	0.116225148	0.007120974
15184	PCDH9	-0.104016806	0.016092091
15185	CAMP	0.079775917	0.065205592
15186	GREM2	0.064998354	0.133229411
15187	PGLYRP1	0.120348862	0.005314866
15188	ACOT6	0.004888378	0.910185369
15189	UTS2	0.115938341	0.007264986
15190	C5orf58	0.154158349	0.000345218
15191	S100G	0.084875801	0.049747013
15192	FRG2C	-0.014861625	0.731624564
15193	SPOCD1	0.267722076	3.11E-10
15194	CORIN	-0.208353676	1.17E-06

15195	AP000275.65	0.057268974	0.185960916
15196	LY6K	0.041162697	0.341974154
15197	GOLGA8H	0.116130165	0.007168382
15198	ARHGEF33	0.14933863	0.000529078
15199	PLEKHS1	0.065732787	0.128890871
15200	PCDHA4	-0.108881887	0.011733786
15201	OOEP	0.035699245	0.40990701
15202	KLK14	0.447466901	1.06E-27
15203	UGT2B17	-0.061510749	0.155384922
15204	ANKRD18A	0.08904753	0.039499073
15205	MCF2	-0.023876644	0.581595841
15206	CDH8	-0.218766037	3.22E-07
15207	KRT13	0.213702226	6.07E-07
15208	TCL1A	0.179045731	3.11E-05
15209	EPHA10	0.15623088	0.000286205
15210	RGPD8	-0.062950076	0.145925161
15211	C10orf62	0.245833925	8.34E-09
15212	REM2	0.506390562	3.46E-36
15213	LYG2	0.16928935	8.32E-05
15214	MYB	0.189363567	1.04E-05
15215	NRAP	-0.080705954	0.062123715
15216	ARMC3	-0.052014224	0.229715651
15217	BAI3	-0.202839277	2.25E-06
15218	SYT10	-0.235676613	3.46E-08
15219	KIF19	0.284427204	2.05E-11
15220	WFDC10B	0.2749886	9.75E-11
15221	CCL16	0.150695925	0.000469735
15222	ZNF625	-0.084059363	0.051993045
15223	AANAT	0.349932711	7.42E-17
15224	GLP1R	-0.104209945	0.015895173
15225	FOXA1	0.131230883	0.002354475
15226	CPN1	-0.010194937	0.814003721
15227	UNC79	0.035895371	0.407337907
15228	SCG3	-0.118071104	0.006253689
15229	GLB1L3	0.036754892	0.396192213
15230	MICALCL	-0.093325001	0.030906321
15231	HMHB1	0.118862819	0.005911706
15232	SYN3	-0.091289288	0.034772474
15233	ARL17B	0.071067806	0.100582746
15234	ERVFRD-1	-0.144270616	0.000817773
15235	LECT2	-0.050999638	0.238943189
15236	PFKFB1	0.111353011	0.00994894
15237	INSRR	0.04156677	0.337252458
15238	KLHL38	-0.037243993	0.389932685
15239	OLIG2	0.042829357	0.322769534
15240	NTN5	0.350457685	6.63E-17
15241	APOL5	0.029614625	0.494268186
15242	CTD-2574D22.6	0.242970658	1.25E-08
15243	ANGPTL7	-0.005708111	0.895204778
15244	PRG4	0.05824603	0.178552357
15245	SYT8	-0.014174381	0.743589238
15246	GPC2	0.432617989	8.23E-26
15247	ASIC2	-0.08657788	0.045324754
15248	ARL13A	-0.120385628	0.005300814
15249	RP11-407N17.3	-0.421489347	1.87E-24
15250	NEK10	-0.105348331	0.014776886
15251	AC109829.1	0.322403925	2.09E-14
15252	SORCS1	-0.003638525	0.933086037

15253	AC018512.1	0.196472781	4.68E-06
15254	PNMT	-0.100457422	0.020123686
15255	CSMD2	0.027206353	0.530050364
15256	CA1	-0.099382643	0.021502399
15257	KLRC4	0.090474713	0.036431032
15258	TMEM63C	0.048938027	0.258489115
15259	USP44	-0.085673927	0.047630361
15260	TMED7-TICAM2	-0.205964844	1.55E-06
15261	CTD-2583A14.10	-0.081470008	0.059682684
15262	IL11	0.193588935	6.48E-06
15263	AC005480.1	0.017651933	0.683738342
15264	HIST1H3G	-0.021618069	0.617838465
15265	SEC14L5	-0.016092723	0.710355121
15266	UGT1A8	0.074504635	0.08513114
15267	IL27	0.372046894	5.24E-19
15268	RFPL3S	0.491593834	6.72E-34
15269	ITIH1	-0.032451751	0.453825362
15270	SLC25A41	0.215922479	4.60E-07
15271	SRPK3	0.294452908	3.67E-12
15272	DOCK3	-0.002140634	0.960602713
15273	PP2D1	-0.203107821	2.18E-06
15274	MOGAT1	0.055336783	0.201274431
15275	FRMD7	-0.124370565	0.003962082
15276	ARL14	0.08042602	0.063038428
15277	ORM2	0.078493469	0.069660418
15278	ZNF750	-0.146097248	0.000700103
15279	HUS1B	0.445353626	1.99E-27
15280	CAPN8	0.049266378	0.255304192
15281	C19orf84	0.203970443	1.97E-06
15282	C1orf127	0.288928328	9.55E-12
15283	RP11-1035H13.3	0.063648785	0.141494522
15284	CSAG1	0.151867949	0.000423537
15285	HSPB9	0.330809787	3.97E-15
15286	CCDC114	0.258647696	1.26E-09
15287	CLEC4D	0.123324537	0.004280093
15288	HIST1H4B	-0.066863071	0.122428781
15289	SERPIND1	-0.052155496	0.228451131
15290	RADIL	0.22188821	2.16E-07
15291	AIM1L	0.22705645	1.10E-07
15292	WNT2	0.001707876	0.968562773
15293	SPSB4	-0.154245978	0.000342509
15294	FAM228A	0.066518646	0.124370618
15295	FAM135B	-0.002201564	0.959482272
15296	TTYH1	0.146576267	0.000671954
15297	TRPV6	-0.036344231	0.401494304
15298	CASP14	-0.095672481	0.026910544
15299	ITIH4	0.115595672	0.00744046
15300	GDF10	0.025119738	0.562080717
15301	TMEM132D	-0.014414256	0.739405907
15302	DLGAP1	-0.084789893	0.049979461
15303	SIGLEC6	-0.161435798	0.000176897
15304	SALL4	0.223614308	1.73E-07
15305	KIF25	0.281307009	3.46E-11
15306	CCDC36	0.013231488	0.760105126
15307	DKKL1	0.218628125	3.27E-07
15308	GLS2	-0.128790034	0.002841422
15309	CRHR1	-0.09272361	0.032008195
15310	ACBD7	0.109132242	0.011540884

15311	WNT10B	0.46671308	2.71E-30
15312	PCDHA6	-0.107849343	0.012559841
15313	GRM1	-0.137959602	0.001379723
15314	PRSS57	0.228828241	8.72E-08
15315	MUC6	0.072612702	0.093381525
15316	KRTAP5-1	0.204525697	1.84E-06
15317	DAND5	0.327910765	7.08E-15
15318	TUBB1	0.009221683	0.831480026
15319	GS1-259H13.10	-0.179964481	2.83E-05
15320	PROK2	0.0264336	0.541803863
15321	TDRD5	0.012209	0.77813906
15322	CCNB3	0.033282782	0.442341512
15323	LOXHD1	-0.117787824	0.006380301
15324	SLC4A5	-0.074830697	0.083770488
15325	ESR2	0.150298744	0.000486426
15326	IL9R	0.158582254	0.000230715
15327	WDR38	0.189003201	1.08E-05
15328	SPATA9	0.144453977	0.000805183
15329	LRFN5	-0.134671789	0.00179679
15330	LEFTY2	0.079855312	0.064937682
15331	RRH	0.01451229	0.737698448
15332	OMP	0.133224142	0.002014733
15333	LRRC8E	-0.062098323	0.151468583
15334	POLR2J2	-0.038048754	0.379764744
15335	VWA5B1	-0.179574829	2.94E-05
15336	HBG2	-0.028698555	0.507725583
15337	NYAP1	0.346178571	1.66E-16
15338	HIST3H2BB	0.179657606	2.92E-05
15339	COL24A1	0.014039912	0.745937655
15340	FBN3	-0.146635346	0.000668556
15341	BLK	0.249966186	4.59E-09
15342	PTPN5	0.015075338	0.727916959
15343	PCDHA12	-0.177868509	3.51E-05
15344	RYR3	0.069053243	0.110628379
15345	OR1L8	-0.208014167	1.21E-06
15346	SLC12A5	0.311374303	1.72E-13
15347	ZPLD1	0.011326342	0.793803579
15348	FZD9	-0.00974715	0.822033543
15349	GOLGA8R	-0.000545081	0.98996425
15350	SCN3A	-0.106237038	0.01395253
15351	RP11-20J15.5	-0.090413507	0.036558324
15352	BNC1	0.010584533	0.807033198
15353	PPFIA2	-0.156760494	0.000272715
15354	KLHL41	-0.029767823	0.492036368
15355	AP006285.2	0.139203468	0.00124666
15356	STX19	-0.12602092	0.003503655
15357	GP9	0.115869336	0.007300021
15358	MSH4	0.110302291	0.010676066
15359	VSTM1	0.244525082	1.01E-08
15360	AGBL4	-0.287474569	1.22E-11
15361	CASQ1	0.270230889	2.09E-10
15362	SPIC	-0.083712408	0.052972816
15363	GABRB2	-0.042274488	0.329083785
15364	NPPA	0.369944619	8.53E-19
15365	MYBPH	0.140306063	0.001138697
15366	MDS2	0.277610947	6.36E-11
15367	EN1	0.081260003	0.060345576
15368	CST1	0.056715683	0.190255492

15369	LRRTM2	-0.159491367	0.000212097
15370	VSNL1	-0.009042491	0.834707049
15371	ARPP21	-0.102007733	0.018270687
15372	HIST1H3A	-0.08811969	0.041608791
15373	FAM181B	-0.037970868	0.38074164
15374	GRP	0.057625267	0.183233493
15375	GPR150	0.043260222	0.317921124
15376	RLN1	-0.008467641	0.845077849
15377	PPP5D1	0.000939257	0.982707784
15378	SLITRK6	-0.009713851	0.822631411
15379	CCDC141	-0.131205791	0.002359067
15380	SPATA45	0.129379551	0.002716074
15381	MYLK3	-0.190046124	9.60E-06
15382	SYCP2L	0.189747544	9.92E-06
15383	KIF5A	0.083388412	0.053901593
15384	MTNR1A	-0.070043335	0.105596732
15385	FAM196A	0.050579601	0.242838705
15386	P2RX5-TAX1BP3	-0.013966089	0.747227924
15387	DLX6	0.157501624	0.000254832
15388	TTC24	0.354763734	2.60E-17
15389	OXCT2	0.209369548	1.03E-06
15390	LGR5	0.005565313	0.897811875
15391	KRTAP5-10	0.277878799	6.09E-11
15392	TRPC7	0.00453214	0.916705872
15393	TMPRSS9	0.202275916	2.40E-06
15394	KIF6	-0.102715503	0.017475472
15395	SUMO4	0.113012391	0.008889978
15396	RIMBP2	-0.152529327	0.000399371
15397	GJA3	-0.06292716	0.146072253
15398	ELANE	0.152161704	0.00041264
15399	FBXO43	0.510670246	7.17E-37
15400	GYS2	-0.029402974	0.497360429
15401	ARID3C	0.046142034	0.286722308
15402	BRSK2	0.211877653	7.59E-07
15403	FBLL1	0.140133431	0.00115501
15404	XDH	0.030304827	0.484255952
15405	MGAT5B	0.185807798	1.52E-05
15406	RNASE10	-0.042097792	0.331111179
15407	PCDHGC5	0.169716673	7.98E-05
15408	VIPR2	-0.049529447	0.252772194
15409	ANKRD31	-0.21284345	6.74E-07
15410	SBK3	0.134256485	0.001856993
15411	ODAM	-0.144927786	0.000773478
15412	ERMN	0.225525274	1.35E-07
15413	RP11-723O4.6	0.138075617	0.001366781
15414	ICAM5	0.072831416	0.092396226
15415	MAG	-0.066679786	0.123459166
15416	CSMD1	-0.024944432	0.564813604
15417	FADS6	-0.013169508	0.761194697
15418	C15orf38-AP3S2	-0.23293006	5.03E-08
15419	AL365181.2	-0.081689412	0.058996573
15420	AL139333.1	-0.077925634	0.071710976
15421	NDP	0.011630447	0.788396915
15422	NTRK1	0.293732245	4.16E-12
15423	SERPINE3	0.049549076	0.252583969
15424	IGSF9	0.105998639	0.014169596
15425	TEKT3	0.236543767	3.07E-08
15426	LECT1	-0.153833008	0.000355453

15427	FHAD1	0.153219379	0.000375531
15428	NUTM2D	-0.107424732	0.01291417
15429	ZNF98	-0.204440363	1.86E-06
15430	CCDC180	0.204071077	1.94E-06
15431	COL19A1	-0.089722934	0.038020911
15432	FGFBP1	0.049637921	0.251733245
15433	FBXO39	0.210018965	9.53E-07
15434	RP5-1042K10.14	0.23678015	2.97E-08
15435	MUC12	0.387960102	1.16E-20
15436	10-Mar	-0.007686812	0.859207365
15437	KREMEN2	0.413333078	1.72E-23
15438	RDH16	0.139585168	0.001208261
15439	NLRP14	-0.233870568	4.43E-08
15440	CAPSL	0.070457479	0.103546516
15441	DLX3	-0.003563278	0.934466723
15442	CECR2	0.06667959	0.123460268
15443	SVOPL	-0.280963068	3.66E-11
15444	KCNG1	0.128452025	0.002915646
15445	C3orf49	-0.212627901	6.92E-07
15446	WFDC12	0.072196502	0.095279703
15447	FAT2	0.023848855	0.582035667
15448	KCNK10	-0.146710203	0.000664273
15449	TBL1Y	-0.117633435	0.006450269
15450	CNGA4	0.289783821	8.25E-12
15451	LGSN	-0.084212395	0.051565727
15452	ACOT12	-0.018729131	0.665573732
15453	C17orf64	0.331902871	3.18E-15
15454	RAPSN	0.286634349	1.41E-11
15455	ADCY10	0.298240487	1.88E-12
15456	RP11-872D17.8	0.036821424	0.395337203
15457	TAC1	-0.014045098	0.745847041
15458	SCN2B	-0.088840485	0.039961831
15459	RIPPLY3	0.190820648	8.82E-06
15460	TAS2R10	-0.049664831	0.251475962
15461	TRIM61	-0.00445001	0.918209971
15462	MROH7	0.10242317	0.017800179
15463	SULT1E1	0.139485268	0.001218204
15464	LYPD2	0.068343423	0.11435049
15465	MTRNR2L6	0.175412471	4.51E-05
15466	KLHL35	0.122034152	0.004704101
15467	GPR87	-0.079174155	0.067265857
15468	RASGRF1	0.00696195	0.872364611
15469	BEST3	-0.049433406	0.253694543
15470	ZNF488	0.026220438	0.545068709
15471	TP63	0.049653901	0.251580437
15472	SPINK7	-0.187561162	1.26E-05
15473	ASB4	0.006388202	0.882804112
15474	C10orf82	0.116909753	0.006787499
15475	ARG1	-0.088337981	0.04110407
15476	HGFAC	0.212842577	6.74E-07
15477	ZNF474	0.088331016	0.041120093
15478	RGS22	-0.135565848	0.001673211
15479	TIFAB	0.096455156	0.025681129
15480	GRIN2C	0.181637708	2.37E-05
15481	ABCG5	0.106754162	0.013491718
15482	CYP4F22	0.102694081	0.017499089
15483	ASB15	0.041451405	0.338596246
15484	SLC2A14	0.153679117	0.000360392

15485	IGFL2	0.126608904	0.003352331
15486	MMEL1	0.066110999	0.126699763
15487	RP11-286N22.8	0.083694995	0.053022391
15488	FSD1	0.369347283	9.79E-19
15489	RP11-176H8.1	-0.127816806	0.003059928
15490	UNC13A	0.147689743	0.000610528
15491	FGF20	-0.041353142	0.339743526
15492	SPATA32	0.203325807	2.12E-06
15493	NODAL	0.021587977	0.618327924
15494	SKOR1	0.371853241	5.48E-19
15495	FAXC	-0.089580301	0.038329102
15496	NETO1	-0.035582106	0.411446021
15497	CTNNA3	-0.319010323	4.03E-14
15498	LPAR4	0.030444903	0.482237452
15499	KRBOX1	0.061744603	0.153817137
15500	RP11-38C17.1	-0.127963796	0.003025975
15501	B3GAT2	0.068500495	0.113518487
15502	RP3-509I19.11	0.160119994	0.000200057
15503	DNAH9	-0.125114416	0.003749126
15504	GDNF	0.041642996	0.336366446
15505	HBE1	0.15331389	0.000372372
15506	VGF	0.250154347	4.46E-09
15507	PCYT1B	0.008735619	0.840239851
15508	LEMD1	0.121712641	0.004815483
15509	SLC28A2	0.027682043	0.522879958
15510	VWDE	-0.14927056	0.00053223
15511	GUCY2D	0.208335511	1.17E-06
15512	DISP2	0.163526339	0.000145204
15513	FSIP2	-0.08896019	0.039693728
15514	FAM184B	-0.113173199	0.008792894
15515	PURG	-0.14041319	0.00112868
15516	CTD-3088G3.8	0.215855473	4.64E-07
15517	HIST2H3D	0.065266065	0.131635077
15518	ALG1L2	0.307030877	3.84E-13
15519	CYP1A1	-0.08521055	0.04884986
15520	SYT3	0.118006175	0.006282508
15521	IGFL1	0.055103773	0.203181384
15522	SLC26A10	0.32880015	5.93E-15
15523	FAM86B2	0.003350074	0.938379797
15524	ASAH2	-0.222039167	2.12E-07
15525	C9orf153	-0.03667132	0.397267793
15526	TTN	-0.081942507	0.058213222
15527	PLA2G4B	0.342781353	3.39E-16
15528	SPDYE1	-0.044344282	0.305934083
15529	MMP13	0.073189526	0.090800949
15530	ST6GALNAC5	0.023179301	0.592679831
15531	PADI4	0.114656886	0.007940769
15532	CCL7	0.128701229	0.002860754
15533	ST8SIA5	-0.182581428	2.15E-05
15534	SPDYE2	0.032663612	0.450881882
15535	RP11-302B13.5	0.293608162	4.25E-12
15536	SAP25	0.503537011	9.74E-36
15537	EVPLL	0.039890243	0.357117013
15538	AC231657.1	-0.057967956	0.180638206
15539	PALM2-AKAP2	-0.316522807	6.49E-14
15540	JAKMIP2	0.082567704	0.056315067
15541	NPIPA3	0.433067166	7.24E-26
15542	CLDN24	-0.087840475	0.042261998

15543	KBTBD12	-0.231901596	5.78E-08
15544	ALS2CR11	0.037333922	0.388788331
15545	CFHR1	0.038220568	0.377615161
15546	AC003002.6	-0.184192659	1.81E-05
15547	ZNF559-ZNF177	-0.116148926	0.007158995
15548	HAL	0.018309681	0.672624329
15549	CSPG5	0.09011921	0.037175675
15550	APELA	-0.118938841	0.005879769
15551	LHB	0.420985376	2.15E-24
15552	HIGD1C	-0.08596459	0.046878509
15553	GCNT7	-0.10173637	0.018583851
15554	KIR2DL1	0.1351939	0.001723644
15555	ISLR2	0.037396903	0.387988112
15556	CITED1	0.16794993	9.49E-05
15557	KCNQ5	-0.071264953	0.099639931
15558	KRT6A	0.212037432	7.45E-07
15559	PZP	0.013895274	0.748466267
15560	ARL17A	0.03036998	0.483316533
15561	BPI	0.104620845	0.015483249
15562	C6orf201	-0.042866248	0.322352532
15563	KRT222	0.061228555	0.157292848
15564	PSG4	-0.147615755	0.000614442
15565	BSN	-0.093172057	0.031183423
15566	MNX1	0.281420002	3.39E-11
15567	GALR1	-0.219420663	2.96E-07
15568	MYO1H	-0.068527582	0.113375494
15569	ZNF157	-0.099299759	0.021612049
15570	TDRD1	-0.070934075	0.101226309
15571	ACSBG2	0.167196741	0.000102073
15572	CHRM4	-0.007196234	0.868107997
15573	ZNF709	0.178774375	3.20E-05
15574	KLK15	-0.184081173	1.83E-05
15575	SLC30A8	-0.041459432	0.338502631
15576	BRINP3	0.069816699	0.106732216
15577	TMEM253	0.221238733	2.35E-07
15578	PALM2	-0.100669427	0.019861003
15579	SYCP3	-0.120034599	0.005436358
15580	UGT2A1	-0.039936925	0.356554141
15581	EFNA2	0.188636578	1.12E-05
15582	AC025048.1	-0.159505453	0.00021182
15583	IFNE	0.188856585	1.09E-05
15584	RP11-468E2.4	0.150126268	0.000493844
15585	EPX	0.035288638	0.41531668
15586	OPN4	0.072396585	0.094363367
15587	DAW1	0.309880865	2.27E-13
15588	C15orf26	-0.029668624	0.493480912
15589	SPTSSB	-0.031927833	0.461150631
15590	DNAH10	-0.06577675	0.128634679
15591	KIAA1045	0.150768968	0.000466724
15592	TEX14	0.097468237	0.02416196
15593	OTOA	0.080375812	0.063203657
15594	SERPINA7	-0.070174903	0.104941955
15595	HIST1H3B	-0.012877261	0.766338591
15596	PLA2G10	0.068148356	0.115390397
15597	TICAM2	-0.141642535	0.001019406
15598	C1QTNF9	-0.042060363	0.331541667
15599	TMEM191B	0.270859208	1.89E-10
15600	FCER2	0.205461875	1.65E-06

15601	AL135745.1	0.200610916	2.91E-06
15602	IL12B	-0.154529373	0.000333882
15603	SLC24A4	0.038605799	0.372822744
15604	KCNH4	0.238978872	2.19E-08
15605	ASB17	-0.060936112	0.1592887
15606	C20orf195	0.1368291	0.001511864
15607	SCEL	-0.012869237	0.766479952
15608	RNF148	-0.04713719	0.276444481
15609	GAS2L2	0.077180747	0.074475404
15610	AF131216.1	0.181044746	2.52E-05
15611	ERICH6	0.209003542	1.08E-06
15612	AMPD1	0.098587616	0.022574338
15613	ARPC4-TTLL3	0.220038167	2.74E-07
15614	HOXC13	0.151514658	0.000436997
15615	KY	-0.011961415	0.782524197
15616	HOXC5	0.1203104	0.005329603
15617	AL589743.1	0.297445211	2.17E-12
15618	MYO1A	0.187295685	1.30E-05
15619	FRMPD2	-0.273646281	1.21E-10
15620	TBX5	0.092213011	0.032969908
15621	MOV10L1	0.153454939	0.000367702
15622	SALL3	-0.190060947	9.59E-06
15623	AL591806.1	0.163725809	0.000142477
15624	UPK1A	0.0724569	0.094088522
15625	POU2F3	0.003370287	0.938008746
15626	VSX1	0.067311108	0.119938243
15627	CERS1	0.169764768	7.94E-05
15628	KRT16	0.185476005	1.58E-05
15629	ELL3	0.344196142	2.52E-16
15630	RPL3L	0.281165072	3.54E-11
15631	PCDH19	-0.101668666	0.018662712
15632	PGLYRP2	0.149892227	0.000504079
15633	SH3GL3	0.063885054	0.140019848
15634	EN2	0.12832979	0.00294292
15635	SLC46A2	-0.019500326	0.652687608
15636	GCKR	0.139377631	0.001229002
15637	FXYD7	0.369535905	9.38E-19
15638	GRIK1	-0.039704023	0.359367925
15639	GNRHR	-0.179759071	2.89E-05
15640	CTD-3105H18.18	-0.031584203	0.465990712
15641	ZCCHC16	-0.113072676	0.008853471
15642	FAM72B	0.310664653	1.96E-13
15643	AC008622.1	0.22956065	7.91E-08
15644	SEC16B	0.131577366	0.002291897
15645	ZNF728	-0.268847154	2.60E-10
15646	ASCL5	0.182374738	2.19E-05
15647	KCND2	0.022100737	0.610010701
15648	GJB4	0.050227822	0.246135256
15649	MYT1	-0.005153402	0.905338329
15650	FAM72A	0.366974104	1.69E-18
15651	FAM83E	0.084932668	0.049593639
15652	SCN11A	-0.028641943	0.508563479
15653	OCM	0.328124847	6.78E-15
15654	HAS1	0.100543314	0.020016901
15655	CPNE9	0.310834051	1.90E-13
15656	AC087762.1	0.142763514	0.000928361
15657	TTLL9	0.158220502	0.00023854
15658	OR2W3	0.002001322	0.9631648

15659	AC008074.1	0.238021557	2.51E-08
15660	RP11-371E8.4	-0.089594828	0.038297617
15661	KRT15	0.2671303	3.41E-10
15662	LDHAL6A	-0.169921854	7.82E-05
15663	APOC4-APOC2	0.134249232	0.00185806
15664	NBPF10	0.040907421	0.344978756
15665	LHX8	0.107779779	0.012617296
15666	ASB5	-0.217131321	3.95E-07
15667	PLEKHD1	-0.093705242	0.030226529
15668	GPR111	-0.016919113	0.696201721
15669	CDC20B	-0.037538575	0.386191713
15670	PYDC1	0.098700191	0.022419792
15671	CFAP74	0.232157497	5.58E-08
15672	L1TD1	0.098462141	0.02274768
15673	STAB2	0.085869154	0.047124266
15674	ST6GALNAC1	0.04887636	0.25909032
15675	SIAH3	-0.002066429	0.961967375
15676	CCR6	-0.133516369	0.001968881
15677	RP11-571M6.15	-0.075709169	0.08019186
15678	LRRTM4	0.000664829	0.987759678
15679	RBM44	0.227510454	1.04E-07
15680	ZNF556	-0.075073569	0.082768439
15681	CD1B	0.068181289	0.115214311
15682	CLC	-0.067806001	0.117233417
15683	ELAVL3	0.094628126	0.028629677
15684	CATSPERB	0.208420487	1.16E-06
15685	SSPO	0.428254635	2.84E-25
15686	CTD-2369P2.10	0.495764038	1.56E-34
15687	AGAP5	0.230164404	7.30E-08
15688	PABPC3	0.015587365	0.719059995
15689	CACNG8	0.14538613	0.000743911
15690	GPR62	-0.030307845	0.484212425
15691	GFRA3	0.113251276	0.008746099
15692	C4orf51	-0.102130994	0.018129965
15693	PTGES3L	0.176685805	3.96E-05
15694	ALDH3B2	0.081456015	0.059726666
15695	AC008686.1	0.153466737	0.000367314
15696	GPA33	0.017523359	0.685918962
15697	PRSS21	0.101301675	0.019095273
15698	PDIA2	0.130118753	0.002566023
15699	NIPAL4	0.212381338	7.14E-07
15700	TMEM211	0.108815586	0.011785347
15701	ABCG4	0.120705631	0.00517991
15702	RBM46	0.047458436	0.273180845
15703	MYCBPAP	0.209008849	1.08E-06
15704	FAM19A3	0.226783309	1.14E-07
15705	TEN1	0.165183943	0.000123951
15706	CDK15	-0.09069653	0.035972861
15707	CNBD2	0.098896356	0.022152682
15708	CCDC67	-0.135904724	0.001628445
15709	SNX31	-0.102949428	0.017219384
15710	SOX30	-0.023317424	0.590476765
15711	EREG	0.077788562	0.072213288
15712	RP11-106M3.2	0.357496666	1.42E-17
15713	PRH1-PRR4	0.186083163	1.48E-05
15714	STPG2	-0.17234091	6.15E-05
15715	GALNT13	-0.080855377	0.061639957
15716	KLHL33	-0.13504007	0.001744906

15717	MYLK2	0.257512461	1.50E-09
15718	KLRC4-KLRK1	0.146421163	0.000680952
15719	USP6	0.039249505	0.364899101
15720	C1orf64	-0.109412201	0.011328501
15721	C6orf25	0.071635796	0.097885507
15722	BTNL8	-0.00634864	0.883524726
15723	TMEM236	-0.051182978	0.237256716
15724	ZBBX	0.095422849	0.027313174
15725	PPP1R1C	-0.026082637	0.547184466
15726	TAF7L	-0.115854657	0.007307493
15727	IMPG2	0.038482369	0.374354159
15728	ARMS2	0.326523454	9.32E-15
15729	GS1-114I9.3	-0.041368537	0.33956362
15730	DCAF12L2	-0.069291398	0.109401155
15731	TRPC3	-0.12606766	0.003491403
15732	U2AF1	0.165398588	0.000121424
15733	PNLDC1	0.045736893	0.290979323
15734	HIST1H3C	-0.066107847	0.126717898
15735	SYT16	-0.271809753	1.63E-10
15736	LIPN	-0.038431674	0.374984253
15737	KIR3DL1	0.020678675	0.633197324
15738	KRTDAP	0.243674197	1.13E-08
15739	C6orf165	-0.073416895	0.0897996
15740	NT5C1B	-0.212760273	6.81E-07
15741	GGTLC2	0.080043685	0.064305658
15742	PRH2	-0.098075742	0.023288749
15743	CBLN2	0.04988167	0.249409497
15744	RGS6	-0.039680778	0.35964953
15745	SYNGR4	0.236152421	3.24E-08
15746	NR2E3	0.085067583	0.049231353
15747	WNT8B	0.043704928	0.312967132
15748	DAB1	-0.041383676	0.339386761
15749	OLAH	0.052839034	0.222402609
15750	MMP3	0.118250699	0.006174592
15751	CTB-50L17.14	-0.063428216	0.142881912
15752	SLC22A14	0.280911161	3.69E-11
15753	SLC17A7	0.025052269	0.563131744
15754	TRPV5	-0.027083898	0.53190425
15755	C4BPB	-0.063482099	0.142542029
15756	KEL	0.249975174	4.58E-09
15757	TRAPPC3L	-0.037616245	0.38520902
15758	VWA3A	0.190166656	9.48E-06
15759	SERPINA3	0.13276555	0.002088663
15760	DLEC1	0.109416587	0.011325201
15761	IL20RA	-0.009055566	0.83447148
15762	SLC1A2	-0.179664292	2.92E-05
15763	RP4-734P14.4	0.08865779	0.040373969
15764	HFE2	0.00152213	0.971980344
15765	ERICH6B	0.330250018	4.44E-15
15766	GJB6	0.067740908	0.117586432
15767	CCDC73	0.184587629	1.74E-05
15768	GTSF1L	0.314026933	1.04E-13
15769	DGKB	-0.173585268	5.43E-05
15770	C1orf194	0.197619097	4.11E-06
15771	SLC7A4	0.196376739	4.73E-06
15772	KCNE5	0.073964601	0.087423843
15773	FPGT-TNNI3K	-0.051045983	0.238516085
15774	TMEM132C	0.011684421	0.787438364

15775	ZNF732	-0.020946672	0.628799153
15776	KRT25	0.125071495	0.003761126
15777	HCAR3	0.067721823	0.117690091
15778	FAM69C	0.048220086	0.265548336
15779	A3GALT2	0.212193001	7.31E-07
15780	CC2D2B	-0.043406864	0.316281912
15781	RP11-216L13.17	0.245917847	8.24E-09
15782	AC004076.9	-0.096651065	0.025381088
15783	AC092881.1	-0.22778383	1.00E-07
15784	CYP2W1	0.097141191	0.024643661
15785	CHGA	0.037328096	0.388862402
15786	SMCO2	0.308790588	2.77E-13
15787	CXorf22	0.034009539	0.432435915
15788	SRRM4	0.033391094	0.440857074
15789	PCDHGC4	-0.020881562	0.629866482
15790	PCDHA7	0.066974885	0.12180349
15791	IZUMO1	0.184596917	1.73E-05
15792	HEPHL1	0.060182627	0.164519013
15793	POU3F4	-0.106139521	0.014040965
15794	CPB1	0.017480916	0.686639383
15795	SYT2	0.00567249	0.895855006
15796	GOLGA6L10	0.221651696	2.23E-07
15797	CDH15	0.106243517	0.013946672
15798	AL008723.1	0.115700031	0.007386622
15799	PCDHA2	0.051112812	0.237901154
15800	CPNE6	-0.027775482	0.521477344
15801	MIXL1	0.212935398	6.67E-07
15802	LYZL1	-0.072375311	0.094460463
15803	PCDHA5	0.049461452	0.253424957
15804	SEMA3E	0.081793839	0.05867231
15805	WBSCR28	0.233272763	4.80E-08
15806	NHLH1	0.439345606	1.18E-26
15807	GP5	-0.004167192	0.9233916
15808	NPTX1	0.02892932	0.504317584
15809	ZACN	0.267755937	3.09E-10
15810	KIRREL2	0.224416543	1.56E-07
15811	KLK10	0.17204789	6.33E-05
15812	IFITM5	0.159443874	0.000213034
15813	UGT1A7	0.012104154	0.779995207
15814	RP13-672B3.2	0.052157628	0.228432086
15815	GRIA1	-0.015346867	0.723215492
15816	GPRC6A	-0.208446482	1.15E-06
15817	PGPEP1L	-0.149674572	0.000513773
15818	P2RX6	0.269174374	2.47E-10
15819	TMEM151A	0.130330126	0.002524529
15820	RHBDL3	-0.025629272	0.554173807
15821	XKRX	0.181535015	2.40E-05
15822	FAM215A	0.105367054	0.014759086
15823	C22orf15	0.04765349	0.2712121
15824	ATP4B	-0.047623372	0.271515461
15825	SCN7A	-0.082140564	0.057606251
15826	SLC7A10	-0.059137941	0.171982668
15827	LCN1	0.128012999	0.003014687
15828	HTR1D	0.160848879	0.000186898
15829	TTPA	-0.093923498	0.029842151
15830	GDF5	0.119743095	0.00555128
15831	SPAG6	0.003727589	0.931452088
15832	HSPB3	0.1545108	0.000334441

15833	HRK	0.094792289	0.028353353
15834	ADAM32	0.057199612	0.186495338
15835	CCDC116	0.410488069	3.67E-23
15836	CLEC4G	0.11556049	0.007458689
15837	PPP1R42	-0.00640586	0.882482517
15838	IGLL1	0.180693974	2.62E-05
15839	SSUH2	0.161123874	0.000182148
15840	FAM129C	0.257475755	1.51E-09
15841	UNC45B	0.009409146	0.828107072
15842	TRPV3	0.242759255	1.29E-08
15843	AC244230.1	0.327497854	7.68E-15
15844	UGT1A3	-0.070291872	0.104362532
15845	TRPM8	0.06557496	0.129813846
15846	RP11-87C12.2	0.560978142	1.12E-45
15847	LHX2	0.151284317	0.000445986
15848	GATA1	0.050913431	0.239739081
15849	C18orf65	-0.074376452	0.085670902
15850	PCDHAC1	-0.240201396	1.85E-08
15851	SLC9C1	-0.017684926	0.683179198
15852	TGM5	0.050183221	0.246555446
15853	ARHGEF4	0.177086176	3.80E-05
15854	FMN2	0.025384615	0.557963597
15855	PRSS45	0.057789544	0.181985958
15856	SHOX2	0.245319292	8.97E-09
15857	SBSN	0.137951904	0.001380585
15858	GABBR2	0.021529956	0.61927216
15859	KIRREL3	0.19980521	3.20E-06
15860	RDH8	0.175640498	4.41E-05
15861	DPEP3	0.109454553	0.011296675
15862	TMC1	0.028780706	0.506510981
15863	CACNG5	0.067192759	0.120592239
15864	AC008914.1	0.238853704	2.23E-08
15865	SPINK9	-0.188000636	1.20E-05
15866	CCDC169	-0.053404873	0.217482782
15867	CTD-2006C1.13	-0.029689387	0.493178374
15868	ADARB2	-0.025844953	0.550843319
15869	TKTL1	-0.013915586	0.748111016
15870	ADH4	-0.089670412	0.038134151
15871	MPO	0.023687536	0.584592064
15872	KLRF2	0.129079457	0.002779239
15873	RP11-26J3.4	-0.039826375	0.357888014
15874	MYOZ2	-0.020841596	0.630522022
15875	LRRC37A	0.072266881	0.094956573
15876	AC011380.1	0.002374568	0.956301357
15877	NR1I2	0.151047833	0.000455393
15878	EIF3CL	-0.01080405	0.803112351
15879	BARX1	0.110356804	0.010637214
15880	C8orf34	-0.055870345	0.196956863
15881	TLL2	0.088543201	0.040634296
15882	HAP1	-0.011409259	0.792328411
15883	ACVR1C	-0.130314226	0.002527629
15884	TTC9B	0.393589849	2.87E-21
15885	MMP10	0.07977591	0.065205616
15886	BEGAIN	0.171788589	6.50E-05
15887	OTOS	0.05775472	0.182249889
15888	FAM209A	0.450816903	3.85E-28
15889	SERPINB7	0.071720302	0.097489184
15890	TREML2	0.045428436	0.294248716

15891	PROX2	-0.097982309	0.023421244
15892	EXTL1	0.052356187	0.226663252
15893	LRRC52	-0.170375407	7.48E-05
15894	CHP2	0.036256668	0.402630307
15895	UGT1A1	-0.070764975	0.102044763
15896	USH1G	-0.104020779	0.016088019
15897	CELA2B	0.087696672	0.042601776
15898	NKX2-3	-0.086075577	0.046594059
15899	TRIM31	0.179585113	2.94E-05
15900	ADRA1D	0.206257124	1.50E-06
15901	CFHR3	-0.014682717	0.73473317
15902	MRGPRE	-0.041011271	0.343754424
15903	GNRH2	0.270579213	1.98E-10
15904	PKDREJ	-0.114125642	0.008237008
15905	TP53AIP1	-0.023654192	0.585121111
15906	PLEKHG7	-0.106910773	0.013354833
15907	LGALS7B	0.13396577	0.001900228
15908	TBX1	0.16451247	0.000132182
15909	GPR83	0.197774338	4.04E-06
15910	GPR45	0.036923989	0.394021293
15911	SLC22A9	-0.069643621	0.107605846
15912	TRIM74	0.352838638	3.96E-17
15913	DCST1	0.308956946	2.69E-13
15914	CCIN	0.164583129	0.000131292
15915	DEFA3	-0.003814515	0.929857643
15916	PEX5L	0.071725581	0.097464469
15917	CHRNB2	0.113365339	0.008678134
15918	ACSBG1	-0.036702967	0.396860283
15919	POPDC3	0.157604267	0.000252444
15920	KLK5	0.095354727	0.027423943
15921	GCK	0.154376151	0.000338521
15922	CTB-54O9.9	0.208828924	1.10E-06
15923	KRT1	0.001558064	0.971319156
15924	ZCCHC12	0.139738821	0.001193114
15925	NBPF8	0.214193598	5.71E-07
15926	HIPK4	0.205482872	1.64E-06
15927	AC010642.1	0.091020743	0.035312007
15928	TUBA8	0.196979286	4.42E-06
15929	ST20-MTHFS	0.07626799	0.077980566
15930	CFAP61	-0.058429272	0.177187651
15931	GDPD4	0.025633111	0.554114443
15932	MUCL1	-0.122509007	0.004543847
15933	PLA2G4D	-0.011400998	0.792475349
15934	XXbac-BPG181M17.5	-0.049747309	0.250688556
15935	RPL36A-HNRNPH2	0.033748908	0.435973465
15936	ENDOU	0.077601295	0.072904185
15937	SH3TC2	0.001523256	0.971959637
15938	RP11-766F14.2	-0.062484979	0.148932621
15939	F7	-0.08836175	0.041049426
15940	FGF5	0.134300492	0.001850527
15941	PAX5	0.100264495	0.020365351
15942	CAMK2A	0.096136168	0.026176216
15943	RNF133	-0.069349853	0.109101581
15944	PDZD9	-0.116194454	0.007136263
15945	STH	-0.066129602	0.126592739
15946	OR10Q1	0.088974247	0.039662343
15947	VNN3	0.153937344	0.00035214
15948	TGIF2-C20orf24	-0.143849655	0.000847367

15949	GRIK2	0.029092551	0.501914235
15950	C11orf53	-0.130874912	0.002420388
15951	SLCO1A2	-0.044057386	0.30907698
15952	AC005779.2	0.18938319	1.03E-05
15953	GABRB1	0.006514525	0.880503823
15954	JMJD7	-0.029838947	0.491002066
15955	RP11-231C14.4	0.220821208	2.48E-07
15956	AC018902.1	-0.095721604	0.026831918
15957	GABRR1	0.014004741	0.746552275
15958	GGTLC1	0.242841276	1.28E-08
15959	KCNE1	0.134188319	0.001867049
15960	MTUS2	-0.089724228	0.038018125
15961	HIST1H1T	0.107354247	0.012973834
15962	TSKS	0.241608683	1.52E-08
15963	DDX25	-0.055974407	0.196122737
15964	IL1A	-0.034828238	0.421432105
15965	KLRC2	0.267980109	2.99E-10
15966	CGA	-0.03424424	0.429264558
15967	SH2D6	0.295979309	2.80E-12
15968	HIST1H2BI	-0.043377101	0.316614169
15969	GBP6	0.055516914	0.199809183
15970	IGSF5	0.014097361	0.744934052
15971	BRS3	0.0207929	0.631321154
15972	CRYBA1	0.078812677	0.068528937
15973	SLC6A4	-0.064737882	0.134794895
15974	MSMB	0.127381611	0.003162493
15975	SOHLH2	0.066559833	0.12413716
15976	GAL	0.15274294	0.000391845
15977	RGS9BP	0.062278545	0.150282511
15978	TMEM8C	0.027985071	0.518338244
15979	SHANK1	-0.039022395	0.367682633
15980	MTRNR2L4	-0.010304696	0.812038437
15981	EDDM3A	-0.049922452	0.249022174
15982	GAL3ST2	0.293532845	4.31E-12
15983	SPRR1A	0.1569953	0.000266926
15984	FAM57B	0.199581413	3.28E-06
15985	FCRL1	0.171042215	7.00E-05
15986	BTNL2	0.051184607	0.237241764
15987	CYP2C18	-0.114488551	0.008033592
15988	ART1	-0.014301844	0.74136539
15989	SYT15	0.100362452	0.020242334
15990	CEACAM3	0.191041625	8.61E-06
15991	HEMGN	-0.037500398	0.386675302
15992	GPHA2	0.235518638	3.54E-08
15993	KPNA7	0.170287055	7.54E-05
15994	CABP7	0.437391874	2.08E-26
15995	RP11-690P14.4	-0.077913698	0.071754604
15996	RPA4	0.209403337	1.03E-06
15997	C8orf89	0.141414676	0.001038892
15998	ERVV-2	-0.025416152	0.557474376
15999	B3GALT1	-0.033146587	0.444212132
16000	TNNI3	0.276503863	7.62E-11
16001	CEBPE	0.237204932	2.81E-08
16002	RXRG	0.034567417	0.424919791
16003	KIR2DL3	0.140906891	0.001083559
16004	RP5-874C20.8	0.145505842	0.000736363
16005	GSDMC	-0.043406833	0.316282253
16006	GCSAML	-0.250273707	4.39E-09

16007	KRTAP2-3	0.171134551	6.94E-05
16008	SLAMF9	0.140761673	0.001096655
16009	GOLGA6L2	0.203194415	2.15E-06
16010	CRLF2	0.157497715	0.000254924
16011	IGSF10	-0.022743175	0.599660598
16012	AHSG	0.018198338	0.674500771
16013	SLC25A47	0.058780402	0.174594256
16014	KIR3DL2	0.084059661	0.051992211
16015	SMC1B	0.085706237	0.047546291
16016	EMILIN3	0.226826464	1.14E-07
16017	AC124312.1	-0.170651171	7.28E-05
16018	RP11-190A12.7	-0.097513978	0.024095241
16019	LCN6	0.049860216	0.249613423
16020	RP11-322E11.6	0.157329762	0.000258879
16021	DUOX2	-0.080834273	0.061708093
16022	NOX5	0.062779748	0.14702113
16023	TEX38	0.001311093	0.975863892
16024	RP11-392E22.9	0.308300839	3.04E-13
16025	MAST1	0.330380197	4.32E-15
16026	ACSM4	0.09330746	0.030937995
16027	TGM3	0.15890916	0.000223851
16028	FAM186A	0.196419988	4.71E-06
16029	CLLU1OS	-0.060174603	0.164575399
16030	AC010287.1	-0.020790697	0.631357313
16031	ZSCAN4	0.003999907	0.92645803
16032	ZDHHC19	0.173126731	5.69E-05
16033	CCR9	-0.003743452	0.931161101
16034	SLC2A7	0.052064768	0.229262663
16035	MED12L	-0.171408023	6.75E-05
16036	ABCG8	0.049386936	0.254141664
16037	CTD-3105H18.14	0.1921047	7.65E-06
16038	WNT7A	0.041203296	0.341497851
16039	TMEM14E	-0.135737196	0.001650437
16040	FAM166A	0.218752277	3.22E-07
16041	AC087651.2	0.282458552	2.85E-11
16042	MALRD1	-0.138659583	0.001303317
16043	AZU1	0.162636285	0.000157983
16044	FRMD5	0.176830104	3.91E-05
16045	DUOXA1	-0.099106276	0.021869906
16046	NCAM2	0.133560291	0.001962073
16047	SDR42E2	0.205559387	1.63E-06
16048	NPW	0.305585065	5.00E-13
16049	TMEM105	0.106656215	0.013577956
16050	ERC2	0.050142404	0.24694042
16051	RP11-520P18.5	-0.041565161	0.337271174
16052	RTP3	0.001500025	0.972387095
16053	TTLL2	-0.021605489	0.618043054
16054	TCEAL5	0.049579511	0.252292315
16055	HIST1H2AA	0.059084588	0.17237052
16056	BPIFA2	-0.032814774	0.44878832
16057	TNFRSF13B	0.261667161	7.97E-10
16058	C17orf104	-0.009749056	0.821999318
16059	KCTD8	-0.077237412	0.074262102
16060	KRT20	0.058386675	0.1775042
16061	CLEC6A	-0.002559665	0.952898868
16062	MAGEL2	-0.006193992	0.886342525
16063	RORB	-0.000402309	0.992592811
16064	LINGO2	0.004977408	0.908556708

16065	DRAXIN	-0.03267225	0.450762096
16066	ERAS	0.409995919	4.18E-23
16067	EYS	-0.265869956	4.16E-10
16068	P2RX3	0.123790284	0.004135751
16069	CLDN6	0.19032262	9.32E-06
16070	NOTUM	0.149792788	0.000508487
16071	RGS13	-0.131852347	0.002243318
16072	RP11-762I7.5	0.244327984	1.03E-08
16073	PAQR9	0.118856148	0.005914516
16074	POU4F1	0.11934754	0.005710718
16075	LHCGR	0.01508848	0.727689171
16076	CHST6	0.193920997	6.25E-06
16077	CHD5	-0.068720184	0.112362796
16078	EDN3	0.099252481	0.021674812
16079	C1orf177	0.125752939	0.003574658
16080	ZNF578	-0.048969541	0.258182254
16081	TULP2	0.147374374	0.000627374
16082	AP000295.9	-0.151586145	0.000434241
16083	NPFFR1	0.003640392	0.933051792
16084	EPYC	0.105106972	0.015008032
16085	RNF223	-0.005744193	0.894546199
16086	KLK3	-0.136566846	0.001544135
16087	GRM3	-0.064532305	0.136040431
16088	FAM106A	0.028455756	0.511324263
16089	DCAF4L1	0.235078719	3.76E-08
16090	CCDC42	0.275018661	9.70E-11
16091	NT5C1A	-0.160827925	0.000187265
16092	AC013286.1	-0.151810383	0.000425703
16093	ZNF80	0.28194286	3.11E-11
16094	RP11-248J23.6	-0.131863124	0.002241434
16095	SLC35F4	-0.153835322	0.000355379
16096	RBBP8NL	-0.116988007	0.006750286
16097	CTAGE8	0.173677683	5.38E-05
16098	HEATR9	0.313800171	1.09E-13
16099	RIMS2	0.048774264	0.260087796
16100	PTCHD2	0.114089943	0.008257264
16101	HFM1	-0.007121252	0.869469911
16102	NXF3	0.192256485	7.52E-06
16103	TEPP	0.174830009	4.79E-05
16104	LDHAL6B	0.029367226	0.497883718
16105	FGF17	0.369650342	9.13E-19
16106	PRSS42	0.122132058	0.004670648
16107	ADCYAP1R1	-0.00712353	0.869428521
16108	KCTD19	0.25837277	1.32E-09
16109	PSG5	-0.143703207	0.000857893
16110	CASP16	0.024853578	0.566232462
16111	TMPRSS11E	-0.1170909	0.006701635
16112	UBE2Q2L	-0.07680587	0.075899149
16113	ROS1	0.173574022	5.44E-05
16114	NXF5	0.15262596	0.00039595
16115	C3orf20	-0.151601836	0.000433639
16116	C11orf16	-0.023513201	0.58736056
16117	ECT2L	-0.089425391	0.038666218
16118	MANSC4	0.052592258	0.224572959
16119	MATN1	0.267124695	3.42E-10
16120	LEP	0.012377359	0.775161164
16121	EML5	-0.325485602	1.14E-14
16122	UNC13C	-0.152645103	0.000395275

16123	SCGB2A2	0.15855518	0.000231292
16124	TOP3B	0.068551759	0.113247979
16125	NOTCH2NL	-0.306113045	4.54E-13
16126	MEIG1	0.250171472	4.45E-09
16127	NXPE4	-0.106837937	0.013418342
16128	KCNA2	-0.031362604	0.469126834
16129	DYDC2	0.03748805	0.386831792
16130	TNNI3K	0.083632666	0.05320016
16131	BFSP2	0.139921908	0.001175293
16132	FKSG54	0.195040415	5.51E-06
16133	LANCL3	-0.11562452	0.007425543
16134	CNTNAP2	-0.041300276	0.340361795
16135	LRRC3B	-0.039875191	0.357298627
16136	HIST2H2AB	-0.095029992	0.027957306
16137	GRAPL	-0.003009385	0.944635715
16138	PLP1	-0.048297198	0.264783844
16139	HES7	0.411203293	3.03E-23
16140	IQCF6	0.009083822	0.833962466
16141	ELSPBP1	0.034015204	0.432359211
16142	ZIC2	0.259542337	1.10E-09
16143	HMGA2	0.271536613	1.70E-10
16144	RTP5	0.379377923	9.30E-20
16145	SLC10A4	0.067389839	0.119504708
16146	RBAK-RBAKDN	0.183688962	1.91E-05
16147	RP11-12J10.3	-0.102511383	0.017701646
16148	A1BG	0.112052923	0.009489326
16149	INSC	0.130055319	0.002578597
16150	MOGAT2	0.034947588	0.419841757
16151	ZNF730	-0.003223369	0.940705991
16152	EQTN	0.077669187	0.072653086
16153	CCDC27	0.007710078	0.858785694
16154	ANKRD34B	0.023486526	0.587784712
16155	ARMC4	0.014955924	0.729987845
16156	CTD-2370N5.3	-0.202281248	2.40E-06
16157	MYO15A	0.128281479	0.002953763
16158	SIRPD	-0.026097203	0.546960634
16159	CHRNA7	-0.026833254	0.535709006
16160	DLX2	0.093574097	0.030459527
16161	TCP11	0.0051037	0.906247073
16162	PRSS46	0.172059412	6.33E-05
16163	C1orf100	0.194367699	5.94E-06
16164	MATN4	0.235245365	3.67E-08
16165	LCTL	0.173074317	5.72E-05
16166	MAGEB17	0.121911381	0.004746356
16167	ADAM21	-0.200091086	3.09E-06
16168	GPR25	0.231541383	6.07E-08
16169	CCL25	0.24156791	1.53E-08
16170	RFPL2	0.182036067	2.27E-05
16171	PTTG2	-0.029958619	0.489264373
16172	TPPP2	-0.174440781	4.98E-05
16173	OVOL3	0.237393629	2.73E-08
16174	GUCA1C	0.111455714	0.009880282
16175	IL1RAPL2	-0.005837228	0.892848416
16176	S100A7	0.261857262	7.74E-10
16177	NR0B1	0.025154332	0.56154217
16178	SLC26A5	0.052415518	0.226136609
16179	UTS2B	-0.042211155	0.329809536
16180	CDX1	0.163594249	0.00014427

16181	WNT3A	0.015805841	0.715292177
16182	TMEM110-MUSTN1	0.400182696	5.40E-22
16183	SLC9C2	-0.006499652	0.880774607
16184	BCL2L14	0.136195258	0.001590944
16185	C4orf26	0.086759804	0.044872265
16186	HIST2H4A	0.167136552	0.000102671
16187	SLC22A16	-0.071969644	0.096327243
16188	PRSS37	-0.053612283	0.215699093
16189	FKSG52	0.127643977	0.003100292
16190	CYP26A1	0.044327091	0.306121806
16191	HBM	0.004587754	0.915687555
16192	BMP3	-0.001915231	0.964748289
16193	NKX6-1	-0.05901446	0.172881303
16194	COL9A1	0.008750134	0.839977964
16195	KHDRBS2	0.044997291	0.298859504
16196	ONECUT2	0.151106546	0.000453041
16197	VWA5B2	0.277190807	6.81E-11
16198	CTNND2	-0.074014234	0.087211075
16199	IL31RA	0.155365855	0.000309586
16200	SLC30A10	-0.145650378	0.000727344
16201	SLC28A3	0.147330108	0.000629773
16202	PRAC2	0.148346039	0.000576806
16203	C1QTNF9B	0.044182264	0.307706359
16204	PPP4R4	-0.077589809	0.072946733
16205	FAM92B	0.209598351	1.00E-06
16206	LMOD3	0.095653917	0.026940308
16207	DMP1	0.019843312	0.646989303
16208	LSMEM2	0.066654853	0.123599851
16209	ZNF804B	-0.198757703	3.61E-06
16210	HAO1	0.009184747	0.83214496
16211	TERT	0.147357425	0.000628292
16212	ALX4	0.017145711	0.69233894
16213	CCDC144A	-0.160770708	0.00018827
16214	C20orf144	0.055237758	0.202083253
16215	MYH13	0.061782271	0.153565736
16216	SLCO5A1	0.155320953	0.000310847
16217	HIST1H3F	-0.069785014	0.106891728
16218	CPZ	0.074989106	0.083115815
16219	AC104532.2	0.093136938	0.031247349
16220	OBP2A	0.201475648	2.63E-06
16221	SEZ6L	-0.10331129	0.016829702
16222	CHRNB4	0.007166618	0.868645868
16223	KCNV2	0.036189083	0.403508444
16224	FER1L5	0.086068809	0.046611363
16225	LGI3	-0.033894124	0.4340004
16226	ATP5L2	-0.0777249	0.072447556
16227	MAB21L1	-0.179402804	3.00E-05
16228	RFPL4A	0.180985665	2.54E-05
16229	OR5K2	0.042618103	0.325164194
16230	UCN2	0.235574497	3.51E-08
16231	HMX2	0.049500565	0.253049321
16232	RP11-1012A1.4	0.022248515	0.607622911
16233	RP1-130H16.18	0.418207151	4.60E-24
16234	RP11-404P21.8	-0.240120651	1.87E-08
16235	CTAGE4	0.266623754	3.70E-10
16236	SPANXB2	0.187208999	1.31E-05
16237	GRM2	0.192416279	7.39E-06
16238	CPNE4	0.041073408	0.343023188

16239	SMIM23	0.339053946	7.38E-16
16240	XKR5	-0.003359921	0.938199029
16241	LHFPL1	-0.021020673	0.627587001
16242	OVCH1	-0.04500893	0.298734402
16243	TUBA3C	0.128648332	0.002872327
16244	FAM72D	0.350045031	7.24E-17
16245	ADIPOQ	0.034928245	0.420099263
16246	TTLL10	0.086871697	0.044595852
16247	EFCAB5	0.107193532	0.013110786
16248	CDK5R2	0.190237245	9.40E-06
16249	CDH20	-0.063233001	0.144118493
16250	CDRT15	0.260329724	9.77E-10
16251	CTC-435M10.3	-0.064310453	0.137394489
16252	CRYGN	0.021888585	0.613445912
16253	TAS2R3	-0.185498238	1.57E-05
16254	SCN1A	-0.078063822	0.071207467
16255	HTR2A	-0.002145595	0.960511478
16256	SEMG2	-0.028248852	0.514401376
16257	AL138751.1	-0.123176559	0.004326898
16258	TSHB	0.007800376	0.85714947
16259	TEX12	0.018123423	0.675764439
16260	AC037459.4	0.217627167	3.72E-07
16261	CPB2	-0.026440712	0.541695104
16262	STON1-GTF2A1L	0.036915609	0.39412871
16263	GABRG1	-0.187634043	1.25E-05
16264	CLEC12B	0.182158916	2.25E-05
16265	UGT2B4	-0.058861491	0.173999381
16266	FCRL2	0.2444664	1.01E-08
16267	SERPINA12	0.048019804	0.267541026
16268	SPRR2A	0.149824028	0.000507098
16269	LRRTM1	-0.171652151	6.59E-05
16270	CFAP54	0.059733798	0.167695272
16271	SLC10A1	-0.077984897	0.071494686
16272	XIRP1	0.078780016	0.068644012
16273	ABCB5	0.018550406	0.668574389
16274	MS4A10	0.016676085	0.700353335
16275	HIST1H4F	-0.120209989	0.005368249
16276	HRNR	-0.120045434	0.005432128
16277	SPATA12	0.002138076	0.960649757
16278	FAM19A1	-0.011688203	0.787371216
16279	HIST1H4A	-0.070361587	0.104018395
16280	OPRD1	0.199122889	3.46E-06
16281	KRT36	0.157386409	0.000257538
16282	ADAM7	0.103898683	0.016213578
16283	HIST1H1B	-0.077829917	0.072061437
16284	RS1	0.124584028	0.003899876
16285	DRC1	0.145449557	0.000739903
16286	SLIT1	0.032726727	0.450007081
16287	WEE2	-0.062306954	0.150096191
16288	UNCX	0.027686654	0.522810698
16289	CCDC38	-0.072306317	0.094775896
16290	RP11-444E17.6	0.139914953	0.001175966
16291	RAET1L	0.042439085	0.32720244
16292	SLC35G5	0.219548918	2.91E-07
16293	IGFBPL1	-0.0288104	0.506072322
16294	GPR156	0.035159132	0.417031586
16295	PHF21B	0.183370317	1.98E-05
16296	RP11-20I23.1	0.278040878	5.93E-11

16297	CXCL17	-0.003967225	0.927057233
16298	HTR3A	0.152413387	0.000403512
16299	TMEM196	0.030346183	0.483659532
16300	C17orf98	0.206240116	1.50E-06
16301	SMIM18	-0.142116443	0.000979954
16302	ZNRF3	-0.356531628	1.76E-17
16303	RP11-244H3.4	0.052633651	0.224207866
16304	LENEP	0.097187407	0.02457509
16305	MT1HL1	0.155268768	0.000312319
16306	C9orf152	-0.016611091	0.701465144
16307	CSF3	0.189205007	1.05E-05
16308	HORMAD2	-0.165712404	0.000117815
16309	KRT32	0.15113039	0.000452088
16310	ACPT	0.171128354	6.94E-05
16311	GADL1	0.04471518	0.301902374
16312	CHRNA4	-0.019776918	0.648090759
16313	KLK13	0.045621673	0.292197706
16314	ACRV1	0.27643423	7.71E-11
16315	ASTN1	-0.055902421	0.196699478
16316	TCTE1	0.138408087	0.001330309
16317	CALML6	0.474351033	2.28E-31
16318	PAGE2B	0.13106322	0.002385314
16319	SLC30A3	0.182070212	2.27E-05
16320	C12orf54	0.122702627	0.004479932
16321	FOXE1	0.172070345	6.32E-05
16322	ERVW-1	-0.190280145	9.36E-06
16323	TVP23C-CDRT4	-0.336180884	1.33E-15
16324	CRISP2	-0.020915754	0.629305895
16325	KRT2	0.020505724	0.636042565
16326	RNF113B	0.019548687	0.651882911
16327	VWC2	-0.053572459	0.216040754
16328	DEFB124	0.228327284	9.32E-08
16329	C2orf66	0.181685046	2.36E-05
16330	DMBT1	0.138237084	0.001348955
16331	CTC-487M23.8	-0.10858297	0.011967836
16332	RP11-45M22.4	0.189502407	1.02E-05
16333	PLD5	0.011609634	0.788766623
16334	DUSP27	-0.057732111	0.18242139
16335	PAGE5	0.121441889	0.00491112
16336	GPR31	-0.005366547	0.901442637
16337	C17orf78	-0.025648746	0.55387269
16338	SPATA22	0.17792319	3.49E-05
16339	CYP3A43	0.114320746	0.008127085
16340	SNCB	-0.020818237	0.630905314
16341	PON1	-0.012116996	0.779767781
16342	HIST1H2AJ	-0.030767092	0.477612044
16343	AC068533.7	0.064013205	0.139224941
16344	KCNH8	0.124115969	0.004037449
16345	HIST1H2BM	0.011861723	0.784291856
16346	AC138969.4	0.323556094	1.67E-14
16347	OR52E8	0.045733054	0.291019866
16348	NDST3	0.002056472	0.962150496
16349	FETUB	-0.072673526	0.093106679
16350	FAM180B	0.121627887	0.004845238
16351	APOA4	0.053196447	0.219285834
16352	RP11-411B6.6	0.269311296	2.42E-10
16353	STOML3	-0.012559767	0.771938498
16354	PAX9	0.021038168	0.627300583

16355	COX8C	0.145492992	0.000737169
16356	AL162426.1	0.093079237	0.031352626
16357	FGF10	-0.102199252	0.018052447
16358	FGL1	-0.006380935	0.882936483
16359	HBQ1	0.300941232	1.16E-12
16360	PDC	-0.121126674	0.005024618
16361	C11orf88	0.029544199	0.495295979
16362	CSF2	0.234053831	4.32E-08
16363	ERICH3	-0.005618936	0.896832737
16364	IGDCC3	0.086323324	0.045964329
16365	RP11-65B7.2	-0.025765561	0.552068119
16366	HIST1H2AH	-0.065627201	0.129507777
16367	HCN4	0.082422612	0.05675094
16368	U51561.1	-0.129764157	0.002637033
16369	IDO2	0.220744352	2.50E-07
16370	OR2C1	-0.062947142	0.145943987
16371	AL109659.1	0.147013639	0.000647173
16372	SLC25A52	0.135899686	0.001629103
16373	HIST1H3J	0.072141128	0.095534552
16374	SNTG2	0.087201409	0.043789668
16375	DEFA4	-0.006784192	0.87559668
16376	RAB44	0.146897053	0.000653694
16377	AC009060.2	0.091412149	0.034527984
16378	HYAL4	-0.135732002	0.001651123
16379	CYP1A2	-0.09483032	0.028289665
16380	SETSIP	-0.084792782	0.049971629
16381	IL4	0.375634185	2.26E-19
16382	CYP2A6	-0.068154143	0.115359443
16383	LIPI	0.074286431	0.086051614
16384	CLEC4C	0.028756311	0.506871501
16385	CDHR4	0.333599694	2.26E-15
16386	URGCP-MRPS24	-0.01820763	0.674344097
16387	MYBPC3	0.376761723	1.73E-19
16388	HORMAD1	0.093600427	0.030412626
16389	CNR2	0.161165682	0.000181436
16390	TMEM132B	-0.075960845	0.07918975
16391	ACOXL	0.172815433	5.87E-05
16392	AXDND1	0.116565833	0.006953234
16393	SLC6A15	0.04625075	0.285587153
16394	CBWD3	0.161881467	0.00016964
16395	CMTM5	0.085727803	0.047490243
16396	TRPV1	0.15663693	0.000275808
16397	GPR37L1	0.241382702	1.57E-08
16398	CYP27C1	0.141895594	0.00099816
16399	CHRM1	-0.072896112	0.092106376
16400	DPF1	0.27689732	7.15E-11
16401	UTS2R	0.026457279	0.541441782
16402	HS3ST6	0.183220377	2.01E-05
16403	AQP8	0.146957731	0.000650293
16404	KLF1	0.066955952	0.121909191
16405	RP4-777O23.3	0.374181575	3.18E-19
16406	LRP1B	-0.125052235	0.003766522
16407	G6PC2	-0.013459884	0.756094157
16408	DQX1	0.256630737	1.71E-09
16409	AVP	0.147142455	0.000640037
16410	CCDC62	0.154417042	0.000337277
16411	KIF1A	-0.030245797	0.485107945
16412	CLVS2	-0.141388361	0.001041164

16413	XAGE3	0.041471183	0.338365623
16414	C4orf22	0.009891361	0.819445439
16415	NUTM2G	0.106884892	0.01337737
16416	SMPX	0.035037375	0.418647659
16417	TAT	-0.007070698	0.870388343
16418	PCDHB1	-0.074009401	0.087231773
16419	SPINK4	0.086959676	0.044379521
16420	RP11-729L2.2	-0.420204853	2.66E-24
16421	ATP11AUN	-0.019560516	0.651686149
16422	OR7D2	0.114342133	0.008115116
16423	NUTM2A	-0.050915205	0.239722687
16424	CYP4Z1	0.117483811	0.006518734
16425	ALK	-0.090719319	0.035926069
16426	PROKR2	-0.020416842	0.637506866
16427	NPY	0.015256791	0.724773993
16428	BTNL3	0.064901193	0.133811716
16429	RXFP4	0.087331366	0.043475297
16430	MOG	-0.177319213	3.71E-05
16431	ZNF492	-0.049493339	0.253118686
16432	OR2G6	-0.085284321	0.048653982
16433	CCDC108	0.112259051	0.009357616
16434	ASMT	0.345950059	1.74E-16
16435	RHAG	0.005024334	0.907698436
16436	RP11-214K3.25	0.140759684	0.001096836
16437	ZRSR1	0.029414823	0.497187032
16438	KCNS2	-0.113738728	0.00845893
16439	MIA	0.044224814	0.307240252
16440	AC100821.2	-0.120168179	0.005384415
16441	ABCC11	-0.012927121	0.765460251
16442	LHFPL4	-0.017766635	0.681795206
16443	ART3	-0.076362548	0.077611337
16444	C12orf71	0.193613895	6.47E-06
16445	TAAR1	-0.24855252	5.63E-09
16446	PRG2	0.271058318	1.83E-10
16447	TCF24	0.019909696	0.645888766
16448	OR7A5	0.006274317	0.884878769
16449	AL023806.1	0.119777467	0.005537617
16450	CALY	0.149963472	0.000500942
16451	MCF2L2	0.145754474	0.000720912
16452	PLGLB1	0.284596312	1.99E-11
16453	TPTE2	0.150643148	0.000471922
16454	NMBR	-0.161976208	0.000168134
16455	CLEC1B	0.047467508	0.273089069
16456	WNT1	0.328799364	5.93E-15
16457	IZUMO1R	0.112556611	0.009170351
16458	WIF1	0.075363756	0.081583907
16459	MMP21	0.160781516	0.000188079
16460	CASKIN1	0.038425048	0.375066666
16461	CLCN1	0.111450764	0.009883582
16462	HIST1H2AB	-0.034236183	0.429373197
16463	IL2	0.202308189	2.39E-06
16464	SLC38A8	-0.064836553	0.134200204
16465	ACTBL2	-0.09242545	0.032566822
16466	HOXC12	0.044991106	0.298925995
16467	OR14I1	-0.063023356	0.145455571
16468	GRM6	0.276350971	7.81E-11
16469	PTRH1	0.198483504	3.72E-06
16470	AC069063.2	-0.183185836	2.01E-05

16471	SH2D4B	0.113448008	0.00862917
16472	HIST1H2BA	-0.002903841	0.946574471
16473	ANXA10	-0.101075185	0.019366569
16474	CHAT	0.179668535	2.91E-05
16475	AC026449.1	-0.132740261	0.002092811
16476	OR2B6	0.317409358	5.48E-14
16477	SLC1A6	0.002234622	0.958874409
16478	NKAIN2	-0.019746457	0.648596362
16479	SLC24A2	-0.198555238	3.69E-06
16480	CAPN9	0.120469523	0.005268873
16481	DNLZ	0.385086557	2.35E-20
16482	FAM178B	0.237260884	2.78E-08
16483	PXT1	0.126197847	0.003457481
16484	C6orf58	-0.030141685	0.486612586
16485	GDAP1L1	-0.029269234	0.499319653
16486	CDKL4	-0.035948013	0.406649967
16487	MAFA	-0.133908105	0.001908913
16488	HELT	-0.063850486	0.140234866
16489	LTC4S	0.140702376	0.001102045
16490	IL5RA	0.051852456	0.231169725
16491	TAS1R1	0.159267631	0.000216544
16492	LIM2	0.203742554	2.02E-06
16493	TDRD12	0.073907171	0.087670558
16494	BPIFB1	0.123238695	0.004307188
16495	OR13A1	0.184411653	1.77E-05
16496	NKPD1	0.301999754	9.59E-13
16497	TEX101	0.160886424	0.000186243
16498	BRINP2	-0.082983433	0.055081542
16499	MUSTN1	0.210281999	9.23E-07
16500	IRGM	0.121063152	0.005047775
16501	CXorf58	-0.122327123	0.004604637
16502	PGC	0.057336191	0.185444088
16503	LHX9	-0.170262958	7.56E-05
16504	C4orf45	-0.226087893	1.25E-07
16505	CD164L2	-0.030749209	0.477868135
16506	TNFSF12-TNFSF13	0.014013685	0.746395954
16507	SIX3	0.13387037	0.001914616
16508	AKR1B15	0.021223361	0.624272077
16509	OR9Q1	0.098943655	0.022088692
16510	DMRT3	0.169071531	8.50E-05
16511	SLC4A10	-0.043195455	0.318646875
16512	AC008810.1	0.143035953	0.000907399
16513	PKD2L2	-0.054810412	0.20560086
16514	TCF23	0.107000645	0.013276835
16515	DNAJB7	0.022493384	0.603675487
16516	TMSB15B	0.095404715	0.027342623
16517	TSGA10IP	0.310072599	2.19E-13
16518	DNAI2	0.130362332	0.00251826
16519	RP11-347C12.3	0.179661373	2.92E-05
16520	NKX2-5	0.21181628	7.65E-07
16521	AC107081.1	-0.071019161	0.100816467
16522	NMUR2	0.087457171	0.043172784
16523	LRRC63	0.185989678	1.49E-05
16524	CRB1	-0.117280764	0.006612687
16525	NPIPB6	0.322459987	2.07E-14
16526	PRR5-ARHGAP8	-0.051103495	0.237986815
16527	C1orf105	0.020218033	0.640787268
16528	MYO16	-0.119361768	0.005704913

16529	IVL	0.070212861	0.104753644
16530	CTAG2	-0.009903551	0.819226767
16531	STK31	0.129900575	0.002609505
16532	PCDHA1	0.033618876	0.437744604
16533	NUP210L	0.069004816	0.110879248
16534	CXorf67	0.302212515	9.23E-13
16535	TNR	0.015849557	0.71453907
16536	SCN5A	0.0012885	0.976279677
16537	TUSC5	0.053193429	0.219312021
16538	FP325331.1	-0.027313547	0.528430223
16539	KIAA1257	0.077583369	0.0729706
16540	FAM217A	-0.047872879	0.269009354
16541	GPR61	0.054272089	0.210094821
16542	FUT9	-0.063950811	0.139611532
16543	GCOM1	-0.172388735	6.12E-05
16544	CCDC60	-0.124380538	0.003959156
16545	MS4A6E	-0.002197913	0.959549409
16546	PAK7	-0.170280865	7.55E-05
16547	EPGN	0.07515669	0.082427731
16548	KLHL10	0.064263021	0.137685327
16549	ST8SIA2	0.004362861	0.919806307
16550	OR2A1	0.075413347	0.081382854
16551	DNTT	-0.085600218	0.047822624
16552	CYP26C1	0.062151199	0.151119867
16553	FOXH1	0.438163818	1.66E-26
16554	FOXD4L1	0.430352659	1.57E-25
16555	YIPF7	0.041805279	0.334485122
16556	RP11-212D19.4	0.067601122	0.118347328
16557	SLC17A8	0.09510875	0.027827137
16558	JPH3	0.139387343	0.001228024
16559	RP4-583P15.14	0.168152538	9.30E-05
16560	SPTA1	0.042573314	0.325673375
16561	RFPL4B	-0.118862859	0.005911689
16562	DSCR8	0.046594619	0.282016603
16563	CHST8	0.097966382	0.023443894
16564	CHRM5	0.08576932	0.047382504
16565	DACH2	-0.026666125	0.538253575
16566	ABCC8	0.159200011	0.000217905
16567	C6orf222	-0.014064617	0.745506016
16568	MAGEA12	0.106640498	0.013591839
16569	FAM183B	0.352722358	4.06E-17
16570	PTX4	0.193213866	6.76E-06
16571	AC004158.1	0.146290462	0.00068862
16572	LCNL1	0.140033328	0.001164569
16573	SLC8A3	0.009362045	0.828954247
16574	VIT	0.018788076	0.664585256
16575	CTAGE15	0.069211062	0.109813929
16576	DLK1	0.069557302	0.108043655
16577	C12orf74	-0.220403627	2.61E-07
16578	ZSCAN5D	0.087119273	0.043989343
16579	CDH19	-0.085862553	0.047141304
16580	LPA	-0.000919696	0.983067862
16581	RIIAD1	0.131412999	0.002321392
16582	OR7C1	-0.046544366	0.282536513
16583	MAGEC3	0.175574775	4.44E-05
16584	POC1B-GALNT4	-0.026036383	0.547895557
16585	ABRA	-0.108867931	0.011744622
16586	AADAC	-0.002731736	0.949736595

16587	CTNNA2	-0.163358207	0.000147541
16588	CACNA1S	-0.043722228	0.31277544
16589	RP11-697E2.12	0.031848872	0.462260325
16590	FAM154A	-0.024171449	0.576939263
16591	IFIT1B	-0.156457243	0.000280364
16592	RP1	0.103905944	0.016206087
16593	DNAH2	0.091981726	0.033413586
16594	SSX1	0.130826284	0.002429522
16595	NEDD8-MDP1	-0.006663838	0.877786191
16596	TEDDM1	-0.053032178	0.220714415
16597	IL23R	0.058760784	0.174738402
16598	FNDC9	-0.138035412	0.001371253
16599	C1orf234	0.210456251	9.03E-07
16600	AC022819.2	-0.162548879	0.000159294
16601	USP50	0.090880578	0.035596432
16602	IL5	-0.140337905	0.001135711
16603	IL1RAPL1	0.025900599	0.549985637
16604	DLGAP2	-0.010693167	0.805092245
16605	CRYBA4	0.244065111	1.07E-08
16606	ASCL3	-0.019908892	0.645902102
16607	AC108925.1	0.103015948	0.017147163
16608	CALCB	-0.04824177	0.265333209
16609	JAKMIP3	0.128959948	0.002804764
16610	ACSM6	-0.101336282	0.019054113
16611	TBX10	0.270266218	2.08E-10
16612	TREX1	0.156755575	0.000272837
16613	SYT14	-0.015255936	0.724788789
16614	AQPEP	0.065420753	0.130720592
16615	AKR1D1	-0.050611523	0.2425411
16616	CCKAR	-0.072462231	0.094064261
16617	TSPAN16	0.150590717	0.000474103
16618	KRTAP5-7	-0.060302746	0.163676676
16619	C1orf146	0.148929825	0.000548272
16620	CDR1	-0.093828398	0.030009117
16621	FRMPD3	0.228872659	8.67E-08
16622	REC114	-0.098536422	0.022644923
16623	MSANTD1	0.155019985	0.000319425
16624	LPPR3	0.159330179	0.000215292
16625	FAM150A	0.019564078	0.651626896
16626	LY6G6C	0.268359642	2.81E-10
16627	FBP2	0.069692232	0.107359906
16628	BTBD18	-0.162566266	0.000159032
16629	SH2D7	-0.033362492	0.44124879
16630	FO538757.3	0.103962524	0.016147819
16631	MSLNL	0.182364199	2.20E-05
16632	AJ239318.1	0.069497188	0.108349381
16633	OTC	-0.088002788	0.041881232
16634	EGR4	0.055077938	0.203393625
16635	ASTL	0.27773583	6.23E-11
16636	SFRP5	0.125601022	0.003615486
16637	ATP2B3	0.023748096	0.583631769
16638	MSANTD3-TMEFF1	-0.199442056	3.33E-06
16639	XKR3	0.027647793	0.523394563
16640	DRD2	-0.001207833	0.977764305
16641	ZNF695	0.171163288	6.92E-05
16642	CHRND	0.144234826	0.000820251
16643	LUZP2	-0.020086261	0.642965408
16644	NLRP7	0.245983131	8.16E-09

16645	SV2C	-0.041575332	0.33715286
16646	RSPO1	0.004566814	0.916070955
16647	GJD3	0.102400109	0.017826017
16648	PCDHA13	-0.0827071	0.05589893
16649	RP11-347C12.1	0.256910815	1.64E-09
16650	ASCL4	-0.199922267	3.15E-06
16651	PCDHGA8	-0.069196361	0.109889592
16652	CHURC1-FNTB	-0.280828716	3.74E-11
16653	MDGA2	-0.015522726	0.720176066
16654	RP11-849F2.7	-0.234042287	4.33E-08
16655	LA16c-431H6.6	0.069270271	0.109509592
16656	SMIM9	0.104142713	0.01596348
16657	KRT6B	0.145833964	0.000716036
16658	P2RY4	-0.05176069	0.231997469
16659	COX6B2	0.186524539	1.41E-05
16660	LPAR3	0.058485725	0.176768779
16661	MAGEC2	0.179103852	3.09E-05
16662	GPR21	-0.154436007	0.000336701
16663	C17orf50	0.227708818	1.01E-07
16664	HMP19	0.162626538	0.000158129
16665	OR10AD1	0.037611964	0.38526314
16666	SLC25A2	0.211518794	7.93E-07
16667	SYT5	0.201472788	2.63E-06
16668	LRRIQ4	0.041818893	0.3343276
16669	SRD5A2	0.027342018	0.528000328
16670	FAM133A	0.126840665	0.003294333
16671	LY6D	0.21781478	3.63E-07
16672	RIMBP3	-0.075594069	0.080653569
16673	ZNF99	-0.211925664	7.55E-07
16674	CPA1	0.063355852	0.143339346
16675	MAGEA3	0.115764799	0.007353385
16676	LCN8	0.06376112	0.140791904
16677	MFSD2B	0.169997729	7.76E-05
16678	GBX2	0.223404646	1.78E-07
16679	KLRC3	0.063525725	0.142267296
16680	CELA1	0.306536706	4.20E-13
16681	XAGE2B	0.155259384	0.000312584
16682	SLC5A5	0.230247734	7.22E-08
16683	FNDC8	0.031138956	0.472303715
16684	C5orf47	-0.161994993	0.000167837
16685	SBK2	0.16951855	8.14E-05
16686	STAR	0.17528576	4.57E-05
16687	CNKSR2	-0.055901038	0.196710572
16688	ISL2	0.241624414	1.52E-08
16689	GPR22	-0.183769826	1.89E-05
16690	NXPE2	-0.115909832	0.007279442
16691	MCIDAS	0.220833705	2.47E-07
16692	PTPRZ1	0.045086577	0.297900728
16693	SLC22A31	0.124538629	0.003913032
16694	PCSK9	0.00633771	0.883723835
16695	CDH12	-0.080336199	0.063334272
16696	OTOF	0.321830674	2.34E-14
16697	GATA4	0.179612491	2.93E-05
16698	CNTN5	-0.001032523	0.980991018
16699	ALLC	0.104991452	0.015119783
16700	ADAM20	-0.122857746	0.004429314
16701	CAPNS2	0.037667143	0.384565878
16702	HHIPL2	0.180440491	2.69E-05

16703	SLC39A2	0.018757827	0.665092449
16704	IFNL1	0.230429905	7.04E-08
16705	TRIM73	0.422913644	1.26E-24
16706	PIWIL1	-0.025924608	0.549615785
16707	LCE1C	0.062531993	0.148626491
16708	FAM72C	0.37850417	1.15E-19
16709	POLR2F	0.235849363	3.38E-08
16710	HBG1	0.004465837	0.917920091
16711	OR3A2	0.010444396	0.809538758
16712	TMEM244	0.157687562	0.000250521
16713	DSCAM	0.026584581	0.539497293
16714	C1orf111	-0.121128029	0.005024125
16715	CTD-2583A14.9	0.0740896	0.086888793
16716	FSCN3	0.232723045	5.17E-08
16717	SP8	0.057873328	0.181352114
16718	HIST1H2AL	0.03222568	0.456978171
16719	RP11-249C24.12	-0.005051377	0.907203861
16720	RP5-877J2.1	-0.098856554	0.022206655
16721	GGTLC3	0.174832737	4.79E-05
16722	MEGF10	-0.006652943	0.877984452
16723	NAT16	0.097041145	0.024792665
16724	MEP1A	-0.038763717	0.3708691
16725	INSL5	0.26080855	9.08E-10
16726	RNASE7	-0.062541354	0.148565591
16727	C19orf45	0.255354117	2.07E-09
16728	BTN1A1	0.097442099	0.024200156
16729	CCR3	0.090723682	0.035917116
16730	PLK5	0.034867103	0.420913835
16731	PKD1L3	0.101389315	0.018991188
16732	KRT72	0.297787094	2.04E-12
16733	GIP	0.255711688	1.96E-09
16734	ACTL8	0.128498121	0.002905421
16735	RP11-475E11.9	-0.121169492	0.005009063
16736	TEX43	0.166402233	0.000110235
16737	LKAAEAR1	0.226190619	1.23E-07
16738	AC110615.1	0.33386702	2.14E-15
16739	SPIN2A	-0.123694326	0.004165124
16740	KCNG2	0.016622585	0.701268483
16741	CHRFAM7A	-0.07213099	0.095581273
16742	RTL1	0.08262738	0.056136603
16743	RPS10-NUDT3	0.074451744	0.085353522
16744	RP11-136C24.3	0.083667168	0.053101694
16745	SUN3	0.137964738	0.001379147
16746	ZFR2	0.130752356	0.002443468
16747	GJD2	-0.104340486	0.015763277
16748	KLK11	0.053224137	0.219045679
16749	CPA2	0.115766198	0.007352669
16750	TAC3	0.320565162	2.99E-14
16751	AP002884.2	0.01478907	0.732884706
16752	CEACAM6	0.08243975	0.056699307
16753	TEX40	0.180413504	2.70E-05
16754	ATP5J2-PTCD1	0.183259937	2.00E-05
16755	FAM221B	0.038323805	0.376327158
16756	DMBX1	0.071103642	0.100410842
16757	INSL6	-0.136506279	0.001551677
16758	RFPL3	0.117442072	0.006537949
16759	MS4A8	0.156997195	0.00026688
16760	FOXN4	0.189300041	1.04E-05

16761	DPPA2	0.116639681	0.006917344
16762	IL17REL	0.129490384	0.002693078
16763	AL365202.1	-0.160323085	0.000196306
16764	SERTM1	-0.013023719	0.76375945
16765	KCNJ9	0.083945453	0.052313037
16766	NPFFR2	0.107430559	0.012909249
16767	PRR18	0.007599426	0.860791564
16768	ELAVL4	0.032966903	0.446686932
16769	TMPRSS11D	-0.204101919	1.94E-06
16770	CRYAA	0.073422278	0.089776002
16771	AC055733.1	0.082474439	0.056594926
16772	FOXN1	0.143079894	0.000904059
16773	SCGB1A1	0.046120643	0.286946022
16774	ATP10B	-0.09014464	0.037121982
16775	LRIT2	0.179270609	3.04E-05
16776	GALNT8	0.109133278	0.011540092
16777	HSPE1-MOB4	0.027113716	0.531452522
16778	IFNK	-0.094619808	0.02864374
16779	HIST3H3	0.064021687	0.139172453
16780	CHST4	0.054565372	0.207637757
16781	GRIA2	-0.140721784	0.001100278
16782	SVOP	0.076981516	0.075229331
16783	DCC	0.072717523	0.092908268
16784	ZNF536	-0.112253478	0.009361155
16785	OBP2B	0.321411202	2.54E-14
16786	TMEM249	0.490847718	8.71E-34
16787	AD000671.6	0.432033431	9.73E-26
16788	RP11-178L8.4	-0.026167219	0.545885333
16789	WFDC13	0.066761332	0.122999901
16790	PAGE2	0.087520911	0.043020192
16791	MAGEB2	0.158370951	0.000235256
16792	TMC3	0.106128695	0.014050812
16793	EPB42	0.015908447	0.71352501
16794	GJA8	-0.115766761	0.00735238
16795	NLRP9	0.157261567	0.000260501
16796	MUC17	0.037595056	0.38547695
16797	PFN3	0.054108786	0.211472001
16798	ANKRD33	0.155436139	0.000307622
16799	CPA5	0.288685525	9.95E-12
16800	KRT4	0.132284766	0.002168827
16801	SLC26A3	-0.113297772	0.008718337
16802	FAM179A	0.390708282	5.89E-21
16803	SPERT	-0.156297414	0.000284476
16804	RAB40AL	-0.08906657	0.039456746
16805	ABCA13	0.032773682	0.449356895
16806	ADH1A	-0.095398943	0.027352002
16807	RP11-565P22.6	-0.175129823	4.64E-05
16808	RP1L1	0.071595396	0.098075435
16809	POU1F1	-0.189696021	9.98E-06
16810	DPP10	-0.175627027	4.42E-05
16811	UROC1	0.26385623	5.69E-10
16812	TEKT4	0.099584256	0.021237692
16813	TSPAN19	0.044197827	0.307535816
16814	SERPINB2	0.05602981	0.195679702
16815	RP11-219A15.1	-0.097949878	0.023467385
16816	TMEM88B	0.120115503	0.005404844
16817	CIDEA	0.067779736	0.11737576
16818	HIST1H2BB	-0.107188556	0.013115046

16819	TMX2-CTNND1	-0.187852113	1.22E-05
16820	ADAMTS19	-0.041669923	0.336053812
16821	TAS2R43	-0.105047184	0.015065779
16822	CES5A	0.014525588	0.737466923
16823	RGPD2	0.06653086	0.124301351
16824	CTD-2410N18.5	-0.07468161	0.084390429
16825	MAGEE2	-0.254732396	2.27E-09
16826	CACNG6	0.263498923	6.01E-10
16827	ATCAY	0.083389683	0.053897923
16828	TMEM257	0.054084199	0.211679914
16829	RP11-343C2.7	-0.055862638	0.197018742
16830	RAB6C	-0.025382264	0.55800007
16831	FAM26D	-0.059441691	0.169786971
16832	RP5-864K19.6	0.064488122	0.136309276
16833	WFIKKN2	-0.108700744	0.011875131
16834	UBE2U	-0.04232442	0.328512326
16835	AC007040.11	0.038293597	0.376703763
16836	UNC80	-0.059078698	0.172413374
16837	FSD2	-0.07847886	0.069712564
16838	PSG9	-0.154135439	0.00034593
16839	TCEAL6	-0.070854493	0.101610841
16840	EFCAB3	0.209323549	1.04E-06
16841	RP11-697E2.6	0.293100863	4.64E-12
16842	TCERG1L	0.06463233	0.135433304
16843	PPP1R27	0.286685722	1.40E-11
16844	IL26	0.051989297	0.229939286
16845	DNMT3L	0.105379736	0.014747041
16846	SNAP91	0.105123779	0.014991834
16847	SPRR1B	0.191450318	8.23E-06
16848	ZNF534	0.095745836	0.026793206
16849	FSHR	-0.019678908	0.649718129
16850	C18orf42	0.054768824	0.205945538
16851	STARD6	-0.001684821	0.968986933
16852	CH507-396I9.6	-0.103449258	0.016683173
16853	ACER1	0.231746957	5.90E-08
16854	RPH3A	0.082275429	0.057195948
16855	RGPD3	0.016702863	0.699895444
16856	INA	0.208884191	1.09E-06
16857	RASA4B	0.133544026	0.001964591
16858	DCD	0.13782154	0.001395271
16859	KCNK12	0.05877731	0.174616967
16860	TAS2R50	-0.187637405	1.25E-05
16861	DSC3	0.111894032	0.009591976
16862	ASB11	-0.11616006	0.00715343
16863	C10orf113	-0.113578183	0.008552568
16864	FAM83A	0.308439757	2.96E-13
16865	HCRTR1	0.082785904	0.055664808
16866	NTN3	-0.138712088	0.001297746
16867	KRT33B	-0.055396254	0.200789808
16868	TMEM74	-0.064960251	0.133457535
16869	PNMA5	0.033959089	0.433119382
16870	TEKT5	0.294366336	3.72E-12
16871	MKRN3	0.151107671	0.000452996
16872	GRIN2B	0.122479054	0.004553808
16873	NUTM2B	-0.019785915	0.64794146
16874	FMO6P	-0.089694432	0.038082327
16875	DPYSL5	0.206210163	1.51E-06
16876	MPP4	0.193647364	6.44E-06

16877	EVX1	0.146824919	0.00065776
16878	C14orf183	0.054535638	0.207885911
16879	NT5DC4	0.018404643	0.671025566
16880	RPTN	0.055841987	0.197184619
16881	AGTR2	-0.041749055	0.335136151
16882	SPRR3	0.150779354	0.000466297
16883	PRB3	-0.179878083	2.85E-05
16884	ANXA8	0.133397115	0.001987476
16885	SERPINA10	-0.043159961	0.319045064
16886	KRTAP1-5	0.036024811	0.405647594
16887	CGB7	0.316513698	6.51E-14
16888	GOLGA8Q	0.022651878	0.601126615
16889	TYR	-0.024743765	0.567949661
16890	AC002398.9	0.268447681	2.77E-10
16891	HMSD	0.378111031	1.26E-19
16892	MEP1B	-0.121860742	0.004763884
16893	TBX4	0.083475729	0.053649964
16894	FKSG68	0.145016825	0.00076765
16895	MIP	0.093895414	0.029891375
16896	SOGA3	0.154167185	0.000344944
16897	C14orf178	0.176368054	4.09E-05
16898	RP11-574K11.31	0.176769992	3.93E-05
16899	PRSS48	-0.142883628	0.000919065
16900	WNT16	0.116149267	0.007158824
16901	C16orf90	0.136472985	0.001555838
16902	RP11-196G11.1	0.091177056	0.0349971
16903	AC243945.1	0.119827796	0.005517664
16904	ADAM2	-0.177647318	3.59E-05
16905	GABRA5	0.035081719	0.418058654
16906	RPE65	-0.073243053	0.090564411
16907	GFI1B	0.065937639	0.127700474
16908	RP11-683L23.1	0.159534299	0.000211254
16909	CADPS	-0.039356308	0.363594645
16910	CCDC37	0.189084474	1.07E-05
16911	SULT2A1	0.0073737	0.864886229
16912	OTOG	0.149815381	0.000507482
16913	AL356585.2	0.020734393	0.632281852
16914	RP1-66C13.4	-0.161120777	0.000182201
16915	SSTR3	0.294707496	3.51E-12
16916	UGT1A4	-0.061422864	0.15597723
16917	PKHD1L1	-0.010893183	0.801521725
16918	MYH1	-0.016207751	0.708378945
16919	GRIFIN	0.003350153	0.938378334
16920	GPR158	0.130652814	0.002462362
16921	GPR33	-0.101905672	0.018387925
16922	GPR128	-0.013880701	0.748721199
16923	SOX2	0.11462208	0.007959883
16924	POU6F2	0.019880869	0.646366583
16925	SPOCK3	-0.069313191	0.109289395
16926	SPATA21	0.208697082	1.12E-06
16927	NKX3-2	0.204208628	1.91E-06
16928	TAS2R30	-0.184570338	1.74E-05
16929	CHST5	0.137098166	0.0014794
16930	FGF22	0.104546162	0.015557413
16931	DLL3	0.296021416	2.78E-12
16932	KCNG3	-0.040775288	0.346540542
16933	SLFN14	0.159520314	0.000211528
16934	OSTN	0.03935798	0.363574245

16935	HCRT	0.20085725	2.83E-06
16936	KRT27	0.114755645	0.007886759
16937	SLC35G6	0.026988703	0.533347684
16938	DCX	0.032605567	0.451687249
16939	C3orf22	0.148529564	0.000567691
16940	FP325317.1	0.135176404	0.00172605
16941	PRR25	0.328592332	6.18E-15
16942	EFCAB8	0.193525392	6.53E-06
16943	AL596220.1	-0.126455902	0.003391125
16944	FAM71D	0.122291515	0.004616624
16945	SPDYC	0.103292121	0.01685015
16946	DPCR1	0.038962682	0.368416683
16947	KIR3DX1	0.152829377	0.000388837
16948	EFCAB1	0.066639749	0.123685139
16949	WDR64	-0.001725687	0.968235088
16950	POU4F3	0.02798403	0.518353804
16951	IL9	0.032852744	0.448263314
16952	AC009977.1	-0.031523092	0.466854399
16953	PMP2	-0.021291513	0.623159162
16954	AC016734.1	0.03654974	0.398835671
16955	AC002365.5	-0.108729612	0.011852506
16956	LRRC9	-0.244384869	1.03E-08
16957	GOLGA8O	0.03457518	0.424815745
16958	POU5F2	-0.129773512	0.002635137
16959	TRH	0.086023646	0.046726973
16960	MMP23B	0.266285087	3.90E-10
16961	GRM5	-0.109394871	0.011341547
16962	UGT2B15	-0.032832523	0.448542861
16963	USH2A	0.00494696	0.90911367
16964	SYCE1	-0.000341769	0.993707435
16965	SYNPO2L	0.156864737	0.00027013
16966	ADAMTS20	-0.050437557	0.244166062
16967	UGT2B10	-0.054065862	0.211835066
16968	SLC35G3	0.167689287	9.73E-05
16969	AC009022.1	0.116803029	0.006838546
16970	SLC8A2	0.181397568	2.43E-05
16971	GALR2	0.194730784	5.70E-06
16972	HTR3B	-0.077984206	0.071497206
16973	TSPEAR	-0.041179706	0.341774557
16974	C17orf47	0.097518334	0.024088895
16975	PLA2G2C	0.143775746	0.000852664
16976	MAS1L	-0.048575376	0.262038537
16977	CFAP99	0.303016854	7.98E-13
16978	SLC6A2	-0.091275237	0.034800529
16979	RGAG1	0.048913945	0.258723787
16980	OTX1	0.339585576	6.61E-16
16981	TMEM190	0.217156281	3.94E-07
16982	C9orf171	0.062767965	0.147097179
16983	HCRTR2	0.073375504	0.089981227
16984	NPPB	0.189589222	1.01E-05
16985	OR52N2	0.127329763	0.003174918
16986	FAM156A	0.202557397	2.32E-06
16987	HS3ST5	0.028143098	0.51597786
16988	HIST1H3I	-0.063639219	0.141554474
16989	CTRC	0.216259165	4.41E-07
16990	C8orf44-SGK3	-0.089384851	0.038754855
16991	MAGEA6	0.120441839	0.005279394
16992	GABRA3	0.122687474	0.004484905

16993	VSTM2A	0.022540561	0.602916287
16994	CEACAM5	0.060996243	0.15887677
16995	GSTA3	-0.117788632	0.006379937
16996	PTPRT	0.049868761	0.249532187
16997	WDR49	0.028971567	0.503694974
16998	CSNK2B-LY6G5B-562	0.009792686	0.821216102
16999	CTC-429P9.4	-0.021274134	0.62344288
17000	SLC13A5	0.01367762	0.752276485
17001	PROK1	0.114674857	0.007930917
17002	TAS2R46	-0.147767901	0.000606418
17003	KIAA1731NL	-0.006082348	0.888377649
17004	FAM25A	0.123621422	0.004187567
17005	CT62	-0.018151695	0.675287455
17006	TEX19	0.081255013	0.060361399
17007	VWC2L	-0.069466836	0.108504004
17008	C9orf129	0.027832341	0.520624779
17009	MYOG	0.116272699	0.007097346
17010	RP11-574F21.3	0.005895686	0.891781881
17011	NPAS4	0.140439924	0.001126193
17012	FABP2	-0.138780316	0.001290539
17013	RP11-248J23.7	-0.074386355	0.085629101
17014	OR56B4	0.138500857	0.001320293
17015	GJD4	0.095666467	0.026920183
17016	C10orf71	-0.158761579	0.000226925
17017	ALOXE3	0.116904602	0.006789955
17018	SLC5A7	-0.020058408	0.6434262
17019	CTC-360G5.8	0.113199825	0.008776911
17020	C9orf131	-0.115418892	0.007532459
17021	UBL4B	0.215359112	4.94E-07
17022	HRH4	0.046921362	0.278651977
17023	CA7	0.150864438	0.000462816
17024	MUC5B	0.053464351	0.216970204
17025	ZIC5	0.165048042	0.000125577
17026	PDILT	0.074174323	0.086527642
17027	PLAC1	0.281047235	3.61E-11
17028	CCKBR	0.043731005	0.312678222
17029	FKSG61	0.057555184	0.18376763
17030	CYP19A1	0.138615488	0.001308013
17031	TXNDC2	-0.059449685	0.169729469
17032	C10orf105	0.091126153	0.035099386
17033	TREML4	0.111230993	0.010031059
17034	RP11-468E2.6	0.037479552	0.386939504
17035	AL021546.6	-0.134276203	0.001854093
17036	RNASE13	0.243396779	1.18E-08
17037	PAK6	-0.057159582	0.186804286
17038	KRT34	0.111092187	0.010125209
17039	SLC26A8	0.103459622	0.016672211
17040	C17orf61-PLSCR3	0.2014199	2.65E-06
17041	SPATA8	0.006652545	0.877991688
17042	RIMS4	0.089856333	0.037734576
17043	AIRE	0.171867025	6.45E-05
17044	MFRP	0.135197451	0.001723155
17045	AC016549.1	-0.170964013	7.05E-05
17046	ERCC6-PGBD3	-0.281299337	3.46E-11
17047	LRRC74A	0.014083029	0.745184375
17048	CDSN	-0.038779715	0.370671534
17049	MLN	0.225317474	1.38E-07
17050	SPACA4	0.166949615	0.000104549

17051	TMEM89	0.324116744	1.50E-14
17052	TBATA	-0.096522969	0.025576927
17053	CIB3	0.177677564	3.58E-05
17054	AL645728.1	0.112908141	0.008953425
17055	CUX2	0.09367913	0.030272799
17056	H1FNT	0.152299433	0.000407622
17057	ARL14EPL	0.147318789	0.000630388
17058	LHFPL5	0.080775026	0.061899707
17059	GRID2	-0.101859827	0.018440801
17060	C7orf34	0.200574302	2.92E-06
17061	PRKCG	0.333997779	2.08E-15
17062	GLRA2	0.000990064	0.981772561
17063	ATP13A5	-0.041229616	0.341189291
17064	NKX2-2	0.084957388	0.049527094
17065	C20orf141	0.299256732	1.57E-12
17066	CNGB1	-0.000341801	0.993706832
17067	SPACA3	0.112625626	0.009127398
17068	TRIML2	0.247837249	6.25E-09
17069	HSPC047	0.135851681	0.001635379
17070	GAGE10	0.302374901	8.96E-13
17071	IL17C	0.252681841	3.08E-09
17072	CACNA1I	0.119265012	0.005744498
17073	CRH	-0.092048196	0.033285556
17074	TBC1D3B	0.226006241	1.27E-07
17075	PPP1R17	-0.192735268	7.13E-06
17076	ANXA8L1	0.127523815	0.00312864
17077	AC069368.3	0.003915154	0.928012029
17078	XKR4	-0.045318651	0.295418245
17079	SNTG1	0.07364228	0.088815758
17080	KIAA1210	-0.17117542	6.91E-05
17081	ANKFN1	0.071431783	0.098847602
17082	AMELX	-0.191903376	7.82E-06
17083	ACTN3	0.25093293	3.98E-09
17084	C3orf30	-0.050549497	0.243119594
17085	DNAH3	0.121141491	0.005019231
17086	RBMXL2	0.249579186	4.85E-09
17087	C2orf91	0.105002281	0.015109277
17088	C17orf99	0.237140645	2.83E-08
17089	IGFL3	0.049082872	0.257080788
17090	GOLGA8K	0.080600005	0.062468619
17091	GHRHR	0.134979999	0.001753274
17092	TRPM1	0.042397655	0.327675335
17093	SULT1C3	-0.212541353	7.00E-07
17094	ALOX15	0.047358692	0.274191351
17095	ASCL1	0.04185064	0.333960472
17096	SERPINB5	0.200301189	3.02E-06
17097	CDH18	0.11495706	0.007777623
17098	A2ML1	0.078336373	0.070222859
17099	SEZ6	0.040379598	0.351244301
17100	CNTN2	0.080390373	0.063155704
17101	BANF2	0.062572458	0.148363382
17102	UMODL1	-0.071903895	0.096632555
17103	GRK7	0.207402156	1.31E-06
17104	FOXB1	0.121490008	0.004893999
17105	TAS2R13	-0.179209276	3.06E-05
17106	MMP8	0.059466386	0.169609387
17107	ERN2	0.137828385	0.001394496
17108	TTC29	-0.002971742	0.945327155

17109	ANGPTL5	-0.055192831	0.202450989
17110	NEUROG3	0.175507933	4.47E-05
17111	SHISA8	0.053457093	0.217032704
17112	FBXW12	0.177637099	3.60E-05
17113	DRP2	0.184363752	1.78E-05
17114	SERF1A	-0.038781901	0.37064455
17115	PRSS1	-0.054964571	0.204326853
17116	CNGB3	-0.128850991	0.002828221
17117	GPR52	-0.15936516	0.000214595
17118	PNLIPRP3	-0.123838817	0.004120966
17119	DGKK	-0.100406162	0.02018765
17120	CDH10	0.024077726	0.578417756
17121	C18orf63	0.013744386	0.751107049
17122	C17orf74	0.152793516	0.000390082
17123	FAM71F1	0.227262446	1.07E-07
17124	KRT12	0.038781185	0.370653387
17125	C4orf50	0.115175492	0.007660794
17126	SGCZ	0.118798693	0.005938766
17127	RP11-449H3.3	-0.141947282	0.000993872
17128	ITLN2	0.068376966	0.114172414
17129	ENTHD1	0.182228433	2.23E-05
17130	AC016753.2	0.037943773	0.381081853
17131	CNPY1	0.176053828	4.23E-05
17132	KCNH1	0.066890018	0.122277858
17133	MYO18B	0.026595564	0.539329692
17134	SDR16C5	0.128052571	0.003005636
17135	HIST1H4L	-0.079670534	0.065562597
17136	ATRIP	-0.031087188	0.473040744
17137	FGF8	0.171197082	6.89E-05
17138	AC104981.1	0.11796262	0.006301907
17139	BEND4	0.158154787	0.000239988
17140	AC015688.3	0.022970449	0.596018151
17141	PPIAL4B	0.173673496	5.38E-05
17142	C6orf10	-0.109300843	0.011412562
17143	KRTAP5-6	0.166077518	0.000113744
17144	ZAN	0.198032169	3.92E-06
17145	RNF103-CHMP3	0.155945584	0.000293727
17146	CEACAM20	0.159667302	0.000208661
17147	RP4-613B23.5	-0.036727667	0.396542415
17148	CRISP3	0.016904751	0.696446819
17149	TRPM5	0.02721983	0.529846538
17150	TUBB8	0.209615172	1.00E-06
17151	CTD-2006C1.10	-0.025730972	0.552602156
17152	SERPINA11	-0.019027441	0.660577226
17153	CTRB1	0.141655605	0.001018298
17154	TMCO2	0.133438099	0.001981067
17155	CA5A	0.04935851	0.25441544
17156	PAPL	0.23430745	4.17E-08
17157	OLFM3	0.012543102	0.772232753
17158	MEPE	-0.202705872	2.28E-06
17159	TAAR6	0.032152558	0.458000547
17160	XKR7	0.104639473	0.015464798
17161	KCNT1	0.140772798	0.001095647
17162	COL6A6	-0.142287377	0.000966074
17163	KERA	0.046900433	0.278866671
17164	STXBP5L	-0.177712288	3.57E-05
17165	PTPRQ	0.138599187	0.001309753
17166	CLVS1	0.039259572	0.364776023

17167	TRIM72	0.082720071	0.055860337
17168	C6orf118	0.128100035	0.002994813
17169	NPY4R	0.095583403	0.027053628
17170	RBPJL	0.178652323	3.24E-05
17171	DSC1	-0.051524424	0.23413827
17172	LIPJ	0.010556537	0.807533587
17173	HOXB1	-0.059294262	0.170850049
17174	FKSG51	0.051298214	0.236201004
17175	RBFOX1	0.006038218	0.889182275
17176	REG3A	0.103089999	0.017067078
17177	C12orf42	0.061315311	0.156704409
17178	GOLGA6L4	0.091342972	0.034665463
17179	FOXG1	0.161033417	0.000183698
17180	VCX	0.153969567	0.000351123
17181	SOX1	0.165303696	0.000122535
17182	OR2AE1	-0.092048462	0.033285046
17183	PLGLB2	0.013052046	0.763260917
17184	ST18	0.026127042	0.546502237
17185	ZCCHC5	0.077177746	0.074486718
17186	SOX21	-0.000986691	0.981834646
17187	CELA2A	0.183499003	1.95E-05
17188	PLET1	-0.128576573	0.002888094
17189	GALNTL6	-0.099714511	0.021068183
17190	LMO7DN	0.091141233	0.035069056
17191	CCDC54	-0.135091643	0.001737751
17192	ANKRD18B	0.267994082	2.98E-10
17193	FAM9C	0.01388738	0.748604362
17194	KLK8	0.147843398	0.000602473
17195	CDH7	-0.108403671	0.012110198
17196	CYP7A1	-0.043297747	0.317501124
17197	LRRC3C	0.099666086	0.021131064
17198	RP11-618P17.4	0.024131905	0.577562854
17199	MBL2	-0.108111239	0.012345599
17200	CRNN	-0.00149741	0.972435221
17201	VCX3B	0.06893681	0.111232295
17202	SPACA1	-0.113992016	0.008313058
17203	AP000721.4	0.100894934	0.019584873
17204	FRG2B	-0.001102548	0.979702112
17205	PRDM14	0.064797097	0.13443776
17206	CTXN2	0.04498728	0.298967128
17207	RP11-403P17.5	0.003751117	0.931020497
17208	PLA2G3	-0.07454392	0.084966268
17209	MMP26	-0.051403766	0.235236923
17210	LRRC38	0.150555666	0.000475567
17211	TACR3	-0.08500682	0.049394246
17212	SPINK6	0.071155655	0.100161753
17213	DPPA4	0.032742649	0.449786547
17214	ANKRD20A4	-0.081880967	0.058402897
17215	CBS	0.186856109	1.36E-05
17216	PAGE4	0.015380576	0.72263256
17217	LA16c-306E5.2	-0.002786654	0.948727481
17218	RIMS1	-0.17172743	6.54E-05
17219	KLHL34	-0.014999587	0.729230403
17220	FFAR3	0.103889973	0.016222567
17221	MMP20	-0.04864844	0.261320742
17222	AC006386.1	0.006809893	0.875129245
17223	AKNAD1	0.098291789	0.022984864
17224	RBFOX3	0.136326131	0.001574311

17225	FREM3	-0.222195091	2.08E-07
17226	TPRXL	0.102665096	0.017531089
17227	PTCHD1	0.013535751	0.754763262
17228	KRT40	0.150597366	0.000473826
17229	RLN3	0.031093893	0.472945248
17230	HSPB2-C11orf52	0.007771779	0.857667575
17231	TMEM40	0.017404313	0.687940325
17232	P2RX2	0.064239111	0.137832114
17233	MGC50722	-0.046121539	0.286936653
17234	C10orf90	0.107369519	0.012960886
17235	SLC18A1	0.13419528	0.00186602
17236	IRG1	0.085771027	0.047378079
17237	FRMPD4	-0.04154706	0.337481798
17238	RP11-1212A22.4	0.254956085	2.20E-09
17239	CPSF4L	0.049497009	0.253083462
17240	SFTPA2	0.113874561	0.00838042
17241	GH1	0.268215972	2.88E-10
17242	FKSG56	0.152133565	0.000413673
17243	C2orf71	0.028044371	0.517451851
17244	CTRL	0.268301301	2.84E-10
17245	RSPH6A	0.165437293	0.000120973
17246	CHRNG	0.05655296	0.191532277
17247	NKX2-8	0.183931793	1.86E-05
17248	SULT1A3	0.329844303	4.81E-15
17249	SERPINA9	0.033491862	0.439478601
17250	NANOGP8	0.076842143	0.075760429
17251	HSF5	0.087978634	0.04193771
17252	PRMT8	-0.119271931	0.005741659
17253	AL357140.1	0.171231574	6.87E-05
17254	PLCZ1	-0.113045018	0.008870203
17255	TMPRSS11A	-0.184948288	1.67E-05
17256	SEC14L3	0.115319932	0.007584403
17257	TRIM67	0.016607282	0.70153032
17258	CH17-140K24.8	-0.018182209	0.67477277
17259	C16orf78	0.062861409	0.146494907
17260	LYPD4	0.169861338	7.87E-05
17261	KRT33A	0.01844005	0.67042983
17262	GPR123	-0.008579141	0.843064136
17263	CATSPERD	0.15544425	0.000307396
17264	PPIAL4C	0.271687028	1.66E-10
17265	APOF	-0.042787083	0.32324781
17266	CTC-454I21.3	-0.015978011	0.712327802
17267	DNAH12	0.11055546	0.010496685
17268	RTBDN	0.228459137	9.16E-08
17269	CTD-2116N17.1	-0.016015791	0.711677887
17270	CST9	0.053440925	0.217171985
17271	ASIC4	0.227381031	1.06E-07
17272	UGT2B28	-0.138693182	0.001299749
17273	HEPACAM	0.095676965	0.026903358
17274	PSKH2	-0.125356143	0.003682189
17275	AL356289.1	0.075862166	0.07958145
17276	SHD	0.052582229	0.224661486
17277	NPBWR1	0.013029729	0.763653675
17278	CHRNA3	0.050979079	0.239132829
17279	HSFX1	-0.055116454	0.203077268
17280	SLC22A10	-0.054403694	0.20898968
17281	PDE6H	0.05109955	0.238023096
17282	CCDC185	-0.080549367	0.062634021

17283	AC004953.1	0.159517664	0.00021158
17284	GOLGA8T	0.056140867	0.19479382
17285	C2orf61	0.132057187	0.002207744
17286	VWA3B	0.178245199	3.38E-05
17287	RP11-446E24.4	0.016142701	0.709496265
17288	AC004813.1	0.137172536	0.00147054
17289	C15orf53	0.19460791	5.78E-06
17290	CACNA1B	0.056728723	0.190153449
17291	MYL10	0.215297994	4.97E-07
17292	SCGB1C2	0.141566983	0.001025829
17293	RPL10L	0.078380841	0.070063277
17294	TM4SF19-TCTEX1D2	0.225282029	1.39E-07
17295	KIAA0408	-0.025501182	0.55615637
17296	ADAD2	0.223413923	1.77E-07
17297	OR5C1	0.105801639	0.014351202
17298	FGF21	0.120711956	0.005177546
17299	CDCP2	0.150176837	0.000491658
17300	AL137164.1	-0.174103655	5.15E-05
17301	PDE6A	0.071462789	0.0987009
17302	IL37	0.135805409	0.00164145
17303	CTRB2	0.213010562	6.61E-07
17304	ARHGDIG	0.147943581	0.000597274
17305	ZSCAN5B	0.246621303	7.45E-09
17306	11-Mar	-0.153775648	0.000357287
17307	GYPB	-0.078570234	0.069386926
17308	MAB21L2	-0.155965915	0.000293185
17309	TRDN	-0.021772517	0.615328903
17310	MS4A15	0.078099151	0.071079207
17311	PITX3	0.253450301	2.75E-09
17312	FKSG63	0.075117063	0.082590019
17313	OR13J1	0.24008568	1.88E-08
17314	CALR3	0.119988664	0.005454324
17315	NPIPB7	0.305886864	4.73E-13
17316	FBXW10	0.156280073	0.000284926
17317	RP11-613M10.9	-0.165492248	0.000120336
17318	NTSR2	0.057434154	0.184692763
17319	ATP1A4	0.100658902	0.019873974
17320	CEACAM7	-0.04087026	0.34541754
17321	DEFB132	-0.052910601	0.221775994
17322	CLPSL1	0.205254604	1.69E-06
17323	KRT39	0.169725506	7.97E-05
17324	CPLX2	0.083561932	0.053402503
17325	MAGEA10	0.164761518	0.000129071
17326	PAGE1	0.183587741	1.93E-05
17327	KCNA4	-0.053018742	0.220831556
17328	FAM19A4	0.092388237	0.032637125
17329	LCE5A	0.205707938	1.60E-06
17330	CDRT1	0.314163603	1.02E-13
17331	CT45A10	0.034321432	0.428224457
17332	PRSS55	0.274626615	1.03E-10
17333	MRAP	-0.075844533	0.079651606
17334	AQP10	0.070476437	0.103453424
17335	AL049794.1	-0.112084213	0.009469227
17336	MEIOB	0.098162775	0.023165917
17337	AC104809.3	0.262856871	6.63E-10
17338	LRRC18	-0.140432468	0.001126886
17339	SCGB1C1	0.073562925	0.089161163
17340	MYBPHL	0.174340084	5.03E-05

17341	CAMKV	0.13833373	0.001338387
17342	UBE2F-SCLY	0.063686675	0.14125723
17343	LCN10	-0.079082987	0.0675826
17344	APOA5	-0.042904971	0.321915205
17345	FAM9B	0.034350894	0.427827867
17346	PRSS38	0.03177486	0.463301815
17347	REG4	0.011009542	0.799446478
17348	OR56B1	-0.007315088	0.865950028
17349	DEFA6	-0.011296835	0.79432872
17350	C8A	-0.032930632	0.447187441
17351	GJB7	-0.126813151	0.00330117
17352	KRTAP17-1	0.039204091	0.365454656
17353	LIPF	0.040316745	0.351995157
17354	FMR1NB	0.089085158	0.039415461
17355	PDCL2	0.116752821	0.00686268
17356	FIGLA	0.111942767	0.009560387
17357	ANKRD30B	0.0223765	0.60555829
17358	PROP1	0.158154071	0.000240004
17359	HIGD2B	0.126625503	0.003348146
17360	ZNF560	0.112575352	0.009158669
17361	NEUROD4	0.1108286	0.010306157
17362	CHIA	-0.077950374	0.071620621
17363	AC073072.1	0.091828324	0.033710661
17364	PCSK2	0.057507554	0.184131298
17365	PRRT4	0.197571013	4.13E-06
17366	CLPSL2	0.157721717	0.000249737
17367	ZSCAN5C	0.081901676	0.058339013
17368	RP11-977G19.10	0.070014369	0.105741323
17369	OR2AT4	0.068229462	0.114957121
17370	NRSN1	0.011976208	0.782261979
17371	SENP3-EIF4A1	0.238813643	2.25E-08
17372	TBC1D3E	0.068168846	0.115280816
17373	KCNH7	-0.053554287	0.216196786
17374	EFCAB9	0.07000743	0.105775985
17375	NPAP1	0.051405035	0.235225349
17376	DUOXA2	-0.075144322	0.082478357
17377	PRL	0.111543083	0.009822208
17378	DNAH8	0.064553278	0.135912954
17379	RP11-287D1.3	-0.027665328	0.523131062
17380	AC092159.1	0.123682925	0.004168627
17381	CLPS	0.101678658	0.018651054
17382	LRFN2	-0.048085984	0.266881443
17383	NRXN1	0.030567678	0.480472013
17384	CCDC175	0.107430106	0.012909631
17385	OR3A1	0.034126062	0.430859736
17386	TMPRSS7	-0.069622211	0.107714305
17387	ADIG	-0.023071468	0.594402388
17388	SPDYE4	0.123572627	0.004202649
17389	GPR179	0.175718662	4.38E-05
17390	ROPN1B	-0.118092756	0.006244105
17391	DKK4	0.062765444	0.147113451
17392	THEG	0.096363769	0.025822134
17393	FBXO40	-0.169116794	8.46E-05
17394	ZNF625-ZNF20	-0.087678187	0.042645621
17395	FAM170A	0.261967252	7.61E-10
17396	CADM2	0.032217338	0.45709474
17397	PNLIP	0.044345316	0.305922788
17398	PRG3	0.100186241	0.020464092

17399	CCDC79	0.129270956	0.002738778
17400	CEACAM16	0.109308323	0.011406898
17401	HIST1H1A	-0.118546779	0.006046152
17402	TMEFF2	0.0241753	0.576878544
17403	MYL2	0.214537399	5.47E-07
17404	DEFB118	0.125789935	0.003564778
17405	RP3-468K18.5	0.113885458	0.00837415
17406	NUTM2F	0.138193058	0.001353794
17407	PGBD3	-0.250444819	4.28E-09
17408	AADACL4	0.114969065	0.007771161
17409	CAPZA3	-0.081386632	0.059945141
17410	OR51B4	-0.05488786	0.204960096
17411	ZIC1	0.14678814	0.000659842
17412	TBC1D29	0.293792399	4.12E-12
17413	CSRP3	0.047232856	0.27546982
17414	TMEM151B	0.059241778	0.171229698
17415	CACNA1G	0.06886143	0.111624659
17416	ADRB3	0.148993728	0.00054523
17417	SCRT1	0.011879974	0.783968147
17418	FSTL5	0.063894227	0.139962834
17419	NCBP2L	0.018814904	0.66413556
17420	LRRC26	0.121927427	0.004740814
17421	KIF4B	-0.059663571	0.168196376
17422	OR1K1	0.162945081	0.000153435
17423	CLCA2	0.12527625	0.003704191
17424	EPHA5	-0.004223689	0.922356225
17425	AC022210.1	0.238406697	2.38E-08
17426	PTCHD3	0.044716974	0.301882959
17427	OR6T1	-0.021891676	0.613395803
17428	OR2A42	-0.020498965	0.636153865
17429	CATSPER4	0.16281345	0.000155358
17430	GOLGA8S	0.108076037	0.012374207
17431	PCDHA8	-0.064262931	0.137685883
17432	MYPN	0.170070884	7.71E-05
17433	12-Sep	0.232285069	5.49E-08
17434	FAM9A	0.095058013	0.027910934
17435	GSTA5	0.012249935	0.777414707
17436	PRDM12	0.339739483	6.40E-16
17437	RP11-343C2.11	-0.083396939	0.053876977
17438	DPRX	0.118378056	0.006119046
17439	RP11-508N12.4	-0.045563544	0.292813675
17440	AP003419.11	0.025097102	0.562433225
17441	MUC16	0.066455387	0.124729861
17442	STMN4	0.089546239	0.038403014
17443	SAGE1	0.131469194	0.002311269
17444	TM4SF20	0.01476244	0.733347407
17445	XXbac-BPG246D15.9	0.025274011	0.559680983
17446	NANOS2	0.209293623	1.04E-06
17447	CLEC2L	0.055610397	0.199051834
17448	PCDHA9	-0.114453366	0.008053116
17449	RIPPLY2	0.21041753	9.08E-07
17450	KRT31	0.039640664	0.360135793
17451	CER1	-0.066685661	0.123426035
17452	IL13	0.307840233	3.31E-13
17453	OR52B6	-0.091747312	0.033868454
17454	RP11-123K3.4	0.144816901	0.000780792
17455	TGM7	-0.038917697	0.36897028
17456	BHMG1	0.18291735	2.07E-05

17457	ETV3L	0.167215156	0.000101891
17458	UGT1A5	-0.058099704	0.179647718
17459	SPRR2E	0.103372414	0.016764647
17460	FAM25E	0.165970921	0.000114919
17461	ARHGAP36	0.024449108	0.572569613
17462	MYH4	-0.063926203	0.139764228
17463	KRT85	0.016526406	0.702914736
17464	FAM187B	0.179947978	2.83E-05
17465	FAM159B	0.12342188	0.004249553
17466	RP11-745O10.4	0.040182591	0.353601183
17467	DEFB136	0.000829902	0.984720806
17468	ZIM2	0.031336739	0.469493637
17469	IL20	-0.038637963	0.372424323
17470	CFHR4	-0.040096128	0.354638724
17471	RAG2	-0.116922798	0.006781283
17472	MAGEA8	0.119167536	0.005784627
17473	KRTAP16-1	0.210885687	8.57E-07
17474	KRT79	0.101750073	0.018567927
17475	IL17F	0.099271013	0.021650192
17476	CRX	0.124061688	0.004053685
17477	CT55	0.094512278	0.028826065
17478	C20orf62	0.1492999	0.000530869
17479	OR2AG2	-0.169005127	8.56E-05
17480	FAM170B	-0.028663472	0.508244745
17481	ZNF648	0.057777087	0.182080338
17482	MROH7-TTC4	0.253857139	2.59E-09
17483	BAGE5	0.088929756	0.039761746
17484	DUSP13	0.107036915	0.013245471
17485	OR2L13	-0.127769538	0.003070919
17486	DYDC1	0.101206903	0.019208387
17487	GALP	0.198736886	3.62E-06
17488	CACNG1	0.066245019	0.125930314
17489	IGFL4	0.246873567	7.18E-09
17490	LGALS9B	0.172358073	6.14E-05
17491	C1orf87	-0.038246634	0.377289705
17492	MYOD1	0.13457155	0.001811155
17493	TMC2	0.219221433	3.04E-07
17494	CCDC169-SOHLH2	-0.119085862	0.005818446
17495	AWAT1	-0.054191466	0.21077393
17496	CERS3	0.020910149	0.629397774
17497	FOXE3	0.254417857	2.38E-09
17498	RNF151	0.281212389	3.51E-11
17499	MTRNR2L13	0.109904184	0.010963643
17500	NR5A1	0.024063509	0.578642183
17501	VHLL	0.168798775	8.73E-05
17502	AC003006.7	-0.176700398	3.96E-05
17503	C8B	-0.044859143	0.300347016
17504	RHO	0.27898493	5.08E-11
17505	CALML5	0.190591431	9.04E-06
17506	GJA9	-0.046891536	0.278957967
17507	CST8	0.077443695	0.0734898
17508	CELA3A	0.110329755	0.010656477
17509	OR52W1	-0.184157883	1.82E-05
17510	OR4C6	0.107069155	0.013217646
17511	KHDC1L	0.188322275	1.16E-05
17512	RFPL4AL1	0.152335228	0.000406327
17513	KRT24	0.089858162	0.037730662
17514	KDM4E	-0.033599931	0.438002992

17515	CTAGE6	0.075388649	0.081482934
17516	C1QL2	-0.001732821	0.968103852
17517	RNF17	0.020507335	0.636016027
17518	OR1N1	-0.094802622	0.028336037
17519	RP4-576H24.4	-0.09173905	0.033884581
17520	RP5-1021I20.4	0.006227598	0.885730073
17521	SEL1L2	0.086514237	0.045483953
17522	C3orf84	0.008086651	0.851966164
17523	TMEM225	0.089092271	0.039399673
17524	SPRR2D	0.162011585	0.000167575
17525	ONECUT1	0.035101066	0.417801838
17526	LGALS9C	0.267896364	3.03E-10
17527	DEFB125	-0.011457976	0.791462035
17528	PSMA8	0.137273478	0.001458593
17529	SYT4	-0.006225317	0.885771637
17530	LCN15	0.22955901	7.91E-08
17531	TP53TG3D	-0.029653591	0.493700009
17532	MYF6	0.001631481	0.969968321
17533	LGALS7	0.00104817	0.980703023
17534	ACTL6B	0.12323496	0.004308371
17535	CACNG7	0.17742548	3.67E-05
17536	CELF3	0.105007693	0.015104029
17537	TBC1D3D	0.254812162	2.24E-09
17538	MS4A3	0.052627081	0.224265789
17539	BSPH1	0.058464928	0.176923003
17540	TAS2R42	0.039070189	0.367095762
17541	SLC6A7	0.189926193	9.73E-06
17542	RP11-426L16.10	0.17475667	4.82E-05
17543	C17orf105	0.095836257	0.026649176
17544	PROKR1	0.195720561	5.10E-06
17545	DUXA	0.107186403	0.01311689
17546	IZUMO2	0.144476852	0.000803625
17547	DHRS7C	0.067549203	0.118630918
17548	PCDH11Y	-0.317338911	5.56E-14
17549	FOXL2	0.138398272	0.001331373
17550	MC4R	0.172501015	6.05E-05
17551	CCL1	0.192295392	7.49E-06
17552	METTL21C	-0.091468452	0.034416432
17553	CU104787.1	0.137879183	0.00138876
17554	KRTAP5-4	0.093735931	0.030172228
17555	PPEF2	0.157185412	0.000262324
17556	GIF	-0.02586179	0.550583728
17557	SELV	0.122669457	0.004490824
17558	SLC7A3	0.130447392	0.002501773
17559	LRTM1	0.07188605	0.096715554
17560	AL133335.1	0.076775568	0.076015195
17561	LIN28A	0.07683788	0.075776722
17562	TEX13B	-0.024026523	0.579226235
17563	PPY	0.069048637	0.110652221
17564	SERPINB3	0.265918506	4.13E-10
17565	CST4	0.041680984	0.335925444
17566	CELF6	0.174712286	4.85E-05
17567	AC017028.10	0.026219919	0.545076663
17568	COL2A1	0.063165206	0.144549842
17569	PHGR1	-0.040348083	0.351620655
17570	CTD-2349B8.1	0.008048531	0.852655997
17571	PRSS33	0.104568269	0.015535428
17572	OR10K2	0.093597153	0.030418455

17573	IFNA5	0.077976459	0.071525451
17574	RP11-162P23.2	0.024275566	0.575298888
17575	GRM7	0.033921809	0.433624829
17576	RPRML	0.175040477	4.69E-05
17577	ANTXRL	-0.023692145	0.584518958
17578	PRSS56	-0.048604128	0.261755913
17579	CCDC177	0.167277035	0.000101281
17580	ANKRD30A	-0.06822691	0.114970736
17581	CLEC3A	0.066104027	0.126739886
17582	OLIG3	0.031651633	0.465038731
17583	R3HDML	0.107598948	0.012767738
17584	RP11-507M3.1	-0.0431443	0.319220858
17585	KRT78	0.21741183	3.82E-07
17586	CEACAM8	0.044203454	0.307474177
17587	ANKRD63	-0.004713821	0.913379705
17588	DRD5	-0.093332861	0.030892138
17589	CTD-2207O23.12	-0.094755892	0.02841442
17590	KRTAP1-1	0.035760856	0.409098921
17591	ODF4	0.158724798	0.000227698
17592	KRT73	0.172902377	5.81E-05
17593	OR2B11	0.022094298	0.610114853
17594	FRRS1L	-0.004878684	0.910362727
17595	AC109829.2	0.120808991	0.005141393
17596	DEFB108B	-0.139766357	0.001190418
17597	GDF5OS	0.100427844	0.020160572
17598	NEUROD2	0.094599087	0.028678796
17599	CAV3	0.030520691	0.481147248
17600	BLID	0.067398113	0.119459218
17601	MAGEA4	0.155553153	0.000304377
17602	PTH	5.17E-05	0.999047373
17603	TEKT1	0.177222513	3.75E-05
17604	DPPA3	0.125584745	0.003619885
17605	PCDH15	0.059655232	0.168255953
17606	SPDYE2B	0.019008422	0.660895348
17607	RGS8	-0.113837865	0.008401566
17608	FGF16	0.01010468	0.815620701
17609	IRS4	0.004970781	0.908677928
17610	CTD-2528L19.4	-0.080129377	0.064019829
17611	NUTM2E	0.151836319	0.000424726
17612	STRC	0.286769477	1.38E-11
17613	TMPRSS12	-0.05554302	0.199597478
17614	SHOX	0.090795158	0.035770721
17615	HTR3E	0.136408667	0.001563904
17616	SLX1B	0.331360343	3.55E-15
17617	AMHR2	0.252037397	3.39E-09
17618	RLBP1	0.016125988	0.709783437
17619	ZPBP	-0.091947774	0.033479143
17620	MT1B	0.145054084	0.000765224
17621	ZFP42	0.071273174	0.099600769
17622	TSPYL6	-0.170680895	7.25E-05
17623	IRX4	0.146989597	0.000648513
17624	LPO	0.159139662	0.000219126
17625	HSFX2	0.004380294	0.919486959
17626	INSM1	0.010894334	0.801501189
17627	GALR3	0.177173649	3.77E-05
17628	RPS4Y2	0.06865746	0.112691812
17629	NTF4	0.242498197	1.34E-08
17630	COL20A1	0.123813788	0.004128585

17631	GRM4	0.111750282	0.009685693
17632	AC007163.2	0.111788932	0.009660416
17633	GPR142	0.12768943	0.00308963
17634	CTD-3138B18.4	0.031704601	0.464291697
17635	RPL17-C18orf32	-0.014274455	0.741843058
17636	LGI1	-0.023319137	0.590449466
17637	EPHA8	0.073827175	0.088015143
17638	NXPE1	-0.143973988	0.000838525
17639	USP41	0.05427553	0.210065877
17640	AC093323.1	0.007816784	0.856852217
17641	IL36B	0.066068921	0.126942095
17642	KHDC3L	0.164368515	0.000134012
17643	AKAP14	0.160281962	0.00019706
17644	C1orf110	0.092991931	0.031512493
17645	AC016577.1	-0.09165406	0.034050868
17646	SPANXN3	0.048812975	0.259709283
17647	IL21	0.122156406	0.004662362
17648	BPIFB4	0.093077216	0.031356318
17649	C1orf61	0.126527201	0.003372997
17650	CRYGA	0.120153479	0.005390109
17651	KRTAP21-3	0.134784363	0.001780782
17652	GLRA1	0.011131923	0.797265359
17653	UTF1	0.293341143	4.45E-12
17654	CSN3	0.022554948	0.602684856
17655	TMPRSS11F	0.098987531	0.022029476
17656	SERPINB4	0.176645153	3.98E-05
17657	BPIFC	0.118183486	0.006204088
17658	TPO	0.033418391	0.440483419
17659	OR1J1	0.082281754	0.057176766
17660	MAGEB1	0.146740972	0.00066252
17661	CXCR5	0.091623935	0.034109973
17662	BSX	0.118315203	0.006146403
17663	DDX4	0.12218458	0.004652791
17664	PENK	0.047210315	0.275699265
17665	SDR9C7	0.295805413	2.89E-12
17666	TGM4	0.130727618	0.002448151
17667	FKSG70	0.08820196	0.041417959
17668	C19orf83	0.142010917	0.000988615
17669	GUCA2A	0.129491645	0.002692817
17670	AF165138.7	0.076123378	0.078548002
17671	CCDC83	0.081357262	0.060037821
17672	SLC7A14	0.048421932	0.263550423
17673	TMEM30C	0.105323959	0.014800084
17674	CCDC155	0.110525629	0.010517682
17675	KIAA2012	0.075198736	0.08225582
17676	RP11-315D16.2	0.17819403	3.39E-05
17677	IL22RA2	0.117621566	0.006455676
17678	CYP2A7	-0.075508849	0.080996804
17679	GKN1	0.028757059	0.506860438
17680	PRKAG3	0.136948823	0.001497339
17681	MTRNR2L11	0.09842518	0.022798961
17682	AC010547.9	0.047960102	0.268137003
17683	FGF23	0.091710352	0.033940653
17684	TH	0.014925125	0.730522284
17685	OR1Q1	-0.058401128	0.177396746
17686	RP5-850E9.3	0.015499364	0.720579585
17687	RD3	0.038780587	0.370660773
17688	GTF2A1L	-0.041909839	0.33327656

17689	AL354828.2	0.090958948	0.035437163
17690	SLC15A5	0.141132111	0.001063534
17691	VSTM2B	0.016018593	0.711629706
17692	TLR9	0.314573749	9.40E-14
17693	ASB18	0.015318962	0.723698196
17694	TCEB3B	-0.183726221	1.90E-05
17695	PADI6	0.198522346	3.71E-06
17696	KCNU1	-0.096778529	0.025187501
17697	CABP5	0.150282525	0.000487119
17698	KCNC1	0.044902937	0.299874931
17699	METTL11B	0.099622789	0.021187424
17700	WFDC6	0.161459671	0.000176501
17701	FEV	0.126003306	0.003508282
17702	RCVRN	0.220140907	2.70E-07
17703	CFHR5	-0.039360997	0.363537442
17704	SLX1A-SULT1A3	0.329861557	4.80E-15
17705	MROH9	-0.100781059	0.019723892
17706	SFTPA1	0.068653663	0.112711753
17707	OR2T2	-0.088043063	0.041787202
17708	F13B	-0.04191423	0.333225875
17709	C5orf60	0.156353538	0.000283026
17710	GRIN1	0.14333936	0.00088457
17711	CSTL1	0.131110045	0.002376664
17712	DGAT2L6	-0.03822999	0.377497498
17713	TAS2R1	0.056742475	0.190045874
17714	RSPH10B	0.34243429	3.65E-16
17715	OR10A2	-0.01894737	0.661916898
17716	TMEM114	0.084752085	0.05008205
17717	HAND1	0.104806335	0.015300385
17718	RP11-569G13.3	0.060603451	0.161582187
17719	RPEL1	0.040538295	0.349352966
17720	MYH7	0.10257222	0.017633969
17721	MUC22	0.060179814	0.16453878
17722	TAAR5	0.098232128	0.023068436
17723	ZDHHC22	0.075779402	0.079911182
17724	SLC6A14	0.153242604	0.000374753
17725	PRB4	-0.170349645	7.50E-05
17726	OR9A4	-0.04394692	0.310292773
17727	SPANXC	0.180949523	2.55E-05
17728	NLRP4	0.079568661	0.065909241
17729	NPIPB9	0.303205145	7.71E-13
17730	1-Dec	0.054323007	0.209666742
17731	OR4K2	0.111094252	0.010123803
17732	RP11-159D12.5	0.155374416	0.000309346
17733	CA6	0.157415042	0.000256863
17734	TMEFF1	0.081384405	0.059952163
17735	NEUROD1	0.097743518	0.023762833
17736	RP11-321N4.5	0.024272401	0.57534872
17737	DKFZp434P228	0.152569593	0.000397942
17738	TRPC5	-0.116216965	0.007125047
17739	DDI1	-0.142208684	0.000972441
17740	ACCSL	0.056095636	0.195154268
17741	RP11-77K12.1	-0.061811699	0.153369538
17742	GSG1L	0.052019596	0.229667476
17743	UCP1	-0.062179855	0.150931129
17744	TMPRSS15	0.133714973	0.001938266
17745	OR2T35	-0.073885213	0.087765032
17746	DMRT1	0.053836166	0.213785563

17747	GSX2	0.097415104	0.02423966
17748	C11orf40	0.131302135	0.002341481
17749	PIWIL3	0.105840249	0.014315449
17750	FGF3	0.128202744	0.002971512
17751	C1orf167	0.166393726	0.000110325
17752	SPINT4	-0.043180561	0.318813923
17753	KLK12	0.128826017	0.002833623
17754	GPR42	0.026004045	0.548392967
17755	CYP2F1	0.13091682	0.002412542
17756	FOXB2	-0.040573762	0.348931154
17757	AC016142.1	0.076961909	0.075303863
17758	POU3F2	0.157280639	0.000260046
17759	ONECUT3	0.127787983	0.003066626
17760	BPIFB2	0.021567146	0.618666857
17761	GAPDHS	0.008654012	0.84171253
17762	KLF17	0.233283317	4.80E-08
17763	GLRA4	0.133859141	0.001916316
17764	VCX3A	0.182331017	2.20E-05
17765	OR6S1	0.14073497	0.001099079
17766	TEX37	0.09483132	0.028287993
17767	GPR32	0.128983695	0.002799675
17768	GPR152	0.132576073	0.002119926
17769	SOX3	0.150551727	0.000475732
17770	CCK	0.100094703	0.020580121
17771	AGBL1	0.069106934	0.110350767
17772	ZP2	0.03988214	0.357214767
17773	HSD3B1	0.011959724	0.782554158
17774	DNAJC5G	0.105078865	0.015035155
17775	PCDH20	-0.064187881	0.138147033
17776	FANCD2OS	0.057495287	0.184225045
17777	MYH2	-0.075309587	0.081803975
17778	LMOD2	-0.021458489	0.620436083
17779	FKSG66	0.0770122	0.075112814
17780	KLF14	0.029921724	0.489799751
17781	CCDC179	-0.101898616	0.018396054
17782	OR52H1	-0.144688313	0.000789355
17783	TFAP2D	0.079576957	0.065880953
17784	DSG3	0.163133534	0.000150719
17785	SATL1	0.039103173	0.366691091
17786	KRTAP29-1	0.162475041	0.000160409
17787	CHRNA9	0.152451068	0.000402162
17788	CTCFL	0.070727396	0.10222736
17789	C14orf39	-0.083878607	0.052501581
17790	C1orf185	0.06232406	0.149984089
17791	RNF222	0.05541612	0.200628113
17792	BEST2	0.218418094	3.36E-07
17793	INMT-FAM188B	-0.032424557	0.454203973
17794	DBX2	0.054508059	0.208116273
17795	OR1G1	0.085833079	0.047217443
17796	GABRG3	0.051686094	0.232671878
17797	CNGA3	0.103341378	0.016797652
17798	TEX26	0.06659605	0.12393215
17799	NRN1L	0.268608104	2.70E-10
17800	DSG1	-0.081597738	0.059282453
17801	SPINT3	-0.00146562	0.973020199
17802	ASIC5	0.052793078	0.222805645
17803	CCDC168	0.296217666	2.69E-12
17804	C1QTNF3-AMACR	-0.057408218	0.184891459

17805	RP5-907C10.4	-0.067448175	0.119184273
17806	GPR112	-0.01034079	0.811392414
17807	C7orf33	0.13182453	0.002248189
17808	MUSK	-0.025233964	0.56030344
17809	SLC39A12	0.079386758	0.066531942
17810	CELA3B	0.060257647	0.163992553
17811	C9orf57	0.008304506	0.848025919
17812	KRTAP10-12	0.160625411	0.000190844
17813	RP11-108K14.8	0.348185823	1.08E-16
17814	MROH2B	0.00191556	0.964742237
17815	KBTBD13	-0.024945313	0.564799857
17816	MRGPRD	0.179148737	3.08E-05
17817	ATP4A	0.077489943	0.073317553
17818	RP5-1052I5.2	-0.12069848	0.005182584
17819	RP11-234B24.6	-0.009245562	0.831050205
17820	OR5A1	0.144609735	0.00079463
17821	SP7	0.090445157	0.036492452
17822	TRIM40	0.104576839	0.015526912
17823	FAM71C	-0.033312947	0.441927803
17824	CLRN2	0.050519521	0.243399515
17825	TBPL2	0.068294481	0.114610704
17826	FLG2	-0.13223059	0.002178034
17827	IFNB1	0.125972822	0.003516304
17828	ANKRD34C	-0.020104547	0.642662975
17829	ISX	-0.009621417	0.824291587
17830	SLCO1B1	-0.029031137	0.50281776
17831	FKBP6	0.140098105	0.001158375
17832	NANOG	0.094586628	0.028699893
17833	RNASE8	0.08483276	0.049863358
17834	SSMEM1	0.171903581	6.43E-05
17835	OR2H2	0.306895665	3.93E-13
17836	CD300LD	0.126350818	0.003418006
17837	SLITRK1	0.079623395	0.065722811
17838	SPINK8	0.040633902	0.348216652
17839	FBXO47	0.095955889	0.026459641
17840	ZIC4	0.141790409	0.001006941
17841	CACNG2	0.107510208	0.012842142
17842	DEFB135	-0.136058361	0.001608514
17843	DMRTC2	0.160997273	0.000184321
17844	SLX1A	0.360432382	7.41E-18
17845	KRTAP5-11	0.141472601	0.001033906
17846	SEMG1	-0.01303647	0.763535041
17847	SPACA5	0.112256813	0.009359037
17848	MMP27	0.135171541	0.001726719
17849	SERPINI2	0.105739816	0.014408615
17850	KRTAP5-3	-0.049332933	0.254661954
17851	MAP3K19	0.117428967	0.006543993
17852	C16orf92	0.176329554	4.11E-05
17853	TBC1D21	0.110446295	0.010573703
17854	DEFB127	0.054802015	0.205670424
17855	HS3ST4	0.005954657	0.890706189
17856	IFNA1	0.117376283	0.006568338
17857	NANOGNB	0.092150299	0.03308971
17858	ANP32D	-0.06812592	0.115510477
17859	ZNF280A	0.211344823	8.11E-07
17860	CCDC144NL	0.039584786	0.36081385
17861	DCAF4L2	0.099786773	0.020974653
17862	CTD-2207O23.10	0.006486637	0.881011572

17863	C2orf83	0.059743359	0.16762713
17864	AMELY	-0.029600628	0.494472371
17865	CYP2A13	0.122657005	0.004494918
17866	PNPLA5	0.149819674	0.000507291
17867	SPATA31E1	-0.096645717	0.025389238
17868	CAGE1	0.094850575	0.028255797
17869	GNG13	0.040610041	0.348500023
17870	AC007906.1	-0.148005667	0.000594073
17871	STMND1	0.028096447	0.51667409
17872	TULP1	0.148735346	0.000557629
17873	OCM2	0.113802083	0.00842223
17874	SULT6B1	0.129440897	0.002703323
17875	MORC1	0.099441361	0.021425012
17876	NKAIN3	0.059649698	0.1682955
17877	PBOV1	-0.151600966	0.000433672
17878	APOC4	0.104072382	0.01603521
17879	HIST2H2AA4	0.175756545	4.36E-05
17880	AWAT2	0.070611498	0.102792139
17881	DSCR4	0.116433134	0.007018144
17882	C20orf173	0.13990026	0.001177387
17883	HOXD12	0.146501577	0.000676273
17884	LDLRAD1	-0.02651982	0.540486057
17885	CTD-2535L24.2	0.047878699	0.268951081
17886	NCR2	0.037487472	0.386839114
17887	FOXR1	0.154908853	0.000322647
17888	RXFP2	0.008015552	0.853252896
17889	NKX6-3	0.125605121	0.003614379
17890	HBZ	0.101334755	0.019055927
17891	HNRNPCL1	0.022829702	0.598272677
17892	PHOX2A	0.112156081	0.009423207
17893	RP11-96O20.4	0.039420693	0.362809666
17894	ZSCAN10	0.156722903	0.000273652
17895	OR3A3	0.263041121	6.45E-10
17896	CALHM1	0.11534844	0.007569406
17897	RP11-552F3.12	0.266642688	3.68E-10
17898	HTR4	-0.02756645	0.524617797
17899	WFDC8	0.065339675	0.131199296
17900	GHRH	0.162754222	0.000156231
17901	C20orf85	0.057287319	0.185819757
17902	ADAM29	0.084709968	0.050196534
17903	MTRNR2L5	-0.00800306	0.85347901
17904	LRRD1	0.007029472	0.871137442
17905	GABRA4	-0.05015831	0.246790345
17906	TCP10L2	0.055901532	0.196706611
17907	LGALS14	0.169826134	7.89E-05
17908	KCNJ6	0.139483212	0.00121841
17909	KCNA7	0.105875848	0.014282553
17910	PLA2G2E	0.103721525	0.016397285
17911	IL19	0.086527483	0.045450781
17912	OR9I1	-0.12614971	0.003469989
17913	TRIML1	0.111662994	0.009742997
17914	SPATA3	0.097040402	0.024793775
17915	RFX4	0.046449587	0.283518839
17916	NCAN	0.205783713	1.59E-06
17917	CCDC63	0.146481841	0.000677418
17918	POM121L2	0.110997242	0.01019006
17919	MYL7	0.211403521	8.05E-07
17920	IFNL2	0.187735339	1.24E-05

17921	KCNB2	0.01586284	0.7143103
17922	KLK2	0.000990496	0.981764605
17923	OR2H1	-0.008721811	0.840488978
17924	THEGL	0.09656347	0.025514866
17925	KRT75	0.090077178	0.037264563
17926	PRB2	-0.100483039	0.020091785
17927	ABCB11	-0.021101456	0.626264914
17928	GAD2	0.090313342	0.036767457
17929	FKSG59	0.061566701	0.155008714
17930	MAGEA1	-0.030305805	0.484241849
17931	FAM205A	-0.048857841	0.259271051
17932	OR8S1	0.104900214	0.015208561
17933	OTOP2	0.078181437	0.070781201
17934	IL36G	0.245582764	8.64E-09
17935	C5orf52	0.105835618	0.014319733
17936	CORO7-PAM16	0.286907481	1.35E-11
17937	PABPN1L	0.150143801	0.000493085
17938	AMY2A	0.1351062	0.001735736
17939	ANKUB1	-0.072420331	0.094255084
17940	PRDM7	0.06990908	0.106268212
17941	NUTM1	0.068457158	0.113747567
17942	RP11-385D13.1	0.106293704	0.013901368
17943	RTP1	0.059003936	0.172958053
17944	RP11-613M10.8	-0.164886247	0.000127539
17945	EXD1	0.094521833	0.028809825
17946	TRIM42	-0.008727807	0.84038079
17947	TBX20	0.158417329	0.000234252
17948	OR2T8	0.029432387	0.496930078
17949	NPB	-0.076426113	0.077363926
17950	C12orf40	-0.122459424	0.004560347
17951	PANX3	0.153705908	0.000359528
17952	RETNLB	0.135681251	0.001657841
17953	NYAP2	0.087412613	0.043279726
17954	TLX1	0.205396582	1.66E-06
17955	ARL5C	0.1165643	0.00695398
17956	ZNF177	-0.023195827	0.592416044
17957	NXNL1	0.146403055	0.000682009
17958	ANKRD20A1	-0.052798641	0.22275683
17959	RP11-468E2.2	0.126618925	0.003349804
17960	OR51J1	-0.032455143	0.453778157
17961	LRRTM3	-0.15014058	0.000493225
17962	HAND2	0.094510676	0.028828789
17963	SLC6A11	0.182644114	2.13E-05
17964	RSPO2	0.06860386	0.112973569
17965	HTR3C	0.128198225	0.002972534
17966	RP11-500M8.7	-0.095648109	0.026949627
17967	RP11-934B9.3	0.117180527	0.006659513
17968	IL22	0.08914901	0.039273921
17969	FRG2	0.026845711	0.535519589
17970	C6orf229	-0.042536525	0.326091995
17971	CCDC129	-0.198242647	3.83E-06
17972	PRSS54	0.083863238	0.05254501
17973	ISL1	0.130463926	0.00249858
17974	SIGLECL1	-0.112155609	0.009423508
17975	OR1J4	0.075374357	0.081540892
17976	CCDC105	0.096554137	0.025529155
17977	PRORY	0.070824686	0.10175516
17978	MAGEC1	0.009284442	0.830350478

17979	CCDC182	0.197341143	4.24E-06
17980	OR2A25	-0.072477029	0.09399694
17981	GCM2	-0.161327773	0.000178699
17982	RNF225	-0.01645123	0.704202479
17983	GUCA1A	0.148171623	0.000585595
17984	GLRA3	0.036611121	0.39804364
17985	TMEM95	0.052424703	0.22605516
17986	ADGB	-0.012407632	0.774626051
17987	SOHLH1	-0.020910065	0.629399141
17988	BRDT	0.115320157	0.007584284
17989	TRHR	-0.137615318	0.001418795
17990	RP11-834C11.12	-0.001584587	0.970831128
17991	HMX3	0.087025117	0.044219181
17992	SPHKAP	0.009580433	0.825027936
17993	LCT	0.113692006	0.008486086
17994	SLURP1	-0.052272126	0.227410902
17995	LRRC74B	0.127161864	0.00321546
17996	SHISA7	0.21332014	6.36E-07
17997	DAZL	0.098603452	0.022552541
17998	IFNA21	0.017285489	0.689960127
17999	PLA2G4E	0.117876303	0.00634051
18000	SLC24A5	0.072861209	0.092262659
18001	PRR30	0.155864336	0.000295903
18002	BTG4	0.165002974	0.000126121
18003	KRT6C	0.09587352	0.026590014
18004	OR1N2	-0.043328713	0.317154819
18005	ANKRD60	0.09947021	0.021387079
18006	AMTN	0.006619737	0.878588731
18007	CLLU1	0.059480928	0.169504875
18008	RAD21L1	0.114827109	0.007847881
18009	ZG16	-0.037158005	0.391028791
18010	SPRR2F	0.158478126	0.000232942
18011	OR52D1	-0.115741818	0.007365163
18012	CSNK1A1L	-0.003620903	0.933409362
18013	SLCO6A1	0.033602044	0.437974165
18014	ZNF705A	0.039125904	0.366412362
18015	F8A3	0.129096003	0.002775721
18016	RIT2	0.046707452	0.280851614
18017	PGA3	0.105177655	0.014940013
18018	KCNJ18	0.104312502	0.01579147
18019	INO80B-WBP1	0.209625334	9.99E-07
18020	OR2T5	-0.076304453	0.077838016
18021	GOLGA6B	0.081703708	0.058952092
18022	OR1F1	0.041622431	0.336605328
18023	FOXD3	-0.000945738	0.982588491
18024	HCN1	0.020716617	0.632573852
18025	FFAR1	0.147468021	0.000622327
18026	HTR3D	0.08688865	0.044554097
18027	MS4A18	0.066393853	0.125080077
18028	CLEC4M	0.062476988	0.148984706
18029	OR2A14	0.006394081	0.882697042
18030	TLX2	0.208029615	1.21E-06
18031	IFNA14	-0.033007373	0.446128852
18032	MBD3L1	0.07320398	0.090737028
18033	PGLYRP4	0.159130414	0.000219314
18034	PTF1A	0.068767241	0.112116459
18035	PNLIPRP1	0.076552491	0.076873941
18036	TLX3	0.184094054	1.83E-05

18037	SLC25A31	0.110925168	0.010239534
18038	C12orf50	0.173024942	5.74E-05
18039	WFDC11	0.201314485	2.68E-06
18040	AF196779.12	0.06877474	0.112077241
18041	DMRTA2	0.259503235	1.11E-09
18042	APOBEC1	0.151578527	0.000434534
18043	B3GNT6	0.136244867	0.00158462
18044	GALNTL5	0.102534699	0.017675681
18045	PASD1	-0.014238822	0.742464664
18046	LUC7L2	-0.110565917	0.010489334
18047	USP17L7	-0.134050424	0.001887544
18048	RHOXF2	-0.061737449	0.153864923
18049	DEFA5	0.114629416	0.007955851
18050	C6orf15	0.065964367	0.127545789
18051	AC011530.4	-0.000470324	0.991340582
18052	FOXL2NB	0.162792614	0.000155665
18053	MRGPRX3	0.094258213	0.029260815
18054	URAD	-0.069791965	0.106856718
18055	SFTA3	0.115611543	0.007432249
18056	C11orf87	-0.009399639	0.828278061
18057	SPATA16	0.085917024	0.047000861
18058	AIPL1	0.113083184	0.008847122
18059	OTP	0.164748603	0.00012923
18060	OR51B5	-0.104035854	0.016072576
18061	L34079.2	0.144120245	0.000828232
18062	GLT6D1	0.113626657	0.008524199
18063	SERPINB10	0.029400217	0.497400771
18064	INSL4	0.148058289	0.000591373
18065	GRXCR2	0.07999343	0.064473776
18066	C22orf42	0.089532324	0.038433243
18067	NPIPB8	0.022958261	0.596213223
18068	AC010655.1	-0.008333606	0.847499885
18069	IDI2	-0.009228458	0.831358067
18070	AC073333.1	0.089781254	0.037895504
18071	AMER2	-0.017864855	0.680132955
18072	KLHL1	-0.002113512	0.961101482
18073	TMEM78	0.056637146	0.190870943
18074	COL6A5	0.010762931	0.803846425
18075	UCMA	0.147542782	0.000618325
18076	RD3L	0.05351068	0.216571542
18077	CALN1	-0.041526548	0.337720574
18078	PSG8	0.025416505	0.5574689
18079	GRK1	0.00641392	0.88233573
18080	CTD-3105H18.16	-0.066269108	0.125792397
18081	MOBP	0.122778037	0.004455259
18082	TRIM39-RPP21	0.183127161	2.03E-05
18083	PSG1	0.033963546	0.43305898
18084	ODF3	-0.103765086	0.016351945
18085	LMNTD1	0.092479046	0.032465797
18086	CST11	-0.045436122	0.294166955
18087	CRYGB	0.152618608	0.000396209
18088	TAS2R60	-0.115987246	0.007240247
18089	IZUMO3	0.085407365	0.048328734
18090	NBPF4	0.048452128	0.263252429
18091	PSG2	0.05974585	0.167609381
18092	NLRP5	0.119448164	0.005669773
18093	RP11-166N6.3	-0.006884367	0.873775
18094	SSX5	0.11236454	0.009290842

18095	AKAP4	-0.047102123	0.276802341
18096	AMER3	0.000860581	0.984156053
18097	AC118758.1	0.179191758	3.06E-05
18098	MGAT4C	0.039798268	0.358227639
18099	ZNF321P	-0.088372873	0.041023877
18100	RFX6	0.06411646	0.138586986
18101	DRGX	0.090810473	0.035739418
18102	OR1E1	0.091669085	0.03402142
18103	AMIGO3	0.176483548	4.05E-05
18104	NPHP3-ACAD11	-0.046555815	0.282418003
18105	ST8SIA3	0.103546664	0.016580396
18106	PIH1D3	0.025597144	0.554670752
18107	AP000350.10	0.154107347	0.000346804
18108	DEFB123	0.091368241	0.034615191
18109	PTGES3L-AARSD1	0.199035455	3.49E-06
18110	SSX3	0.065306144	0.131397668
18111	OR2D2	-0.140604897	0.001110958
18112	TAAR8	0.082149406	0.057579276
18113	C1orf94	0.026356159	0.542988834
18114	KCNA1	-0.002713139	0.950078328
18115	CT45A1	0.025998711	0.548475032
18116	ITIH6	0.03471116	0.422995587
18117	KRT84	0.023668888	0.584887916
18118	FEZF1	0.050912538	0.239747336
18119	PPAN-P2RY11	0.046636551	0.281583284
18120	CRYBA2	0.229274558	8.22E-08
18121	FCRL4	0.145202093	0.000755655
18122	BIVM-ERCC5	-2.69E-05	0.999505126
18123	EBLN1	0.111827429	0.009635297
18124	SAG	0.104230406	0.015874436
18125	CCDC70	-0.066667238	0.123529955
18126	SLC27A6	-0.029721604	0.49270914
18127	MPPED1	0.08301234	0.054996614
18128	LY75-CD302	-0.122211973	0.004643502
18129	AL354828.1	0.062804559	0.146861093
18130	PAX3	0.242199638	1.40E-08
18131	DPPA5	0.177228656	3.75E-05
18132	TBC1D26	0.263498984	6.01E-10
18133	CLEC19A	0.094517811	0.02881666
18134	ZNF729	0.013910355	0.748202503
18135	DEFB131	0.053704369	0.214910545
18136	HOXA10-HOXA9	-0.069528067	0.10819225
18137	GFRA4	0.0244797	0.572089137
18138	H2BFWT	0.019058826	0.660052426
18139	IL31	0.091966922	0.033442157
18140	TAS2R38	-0.027874287	0.519996293
18141	PRAMEF17	0.103861067	0.016252432
18142	PP2672	-0.03314025	0.444299278
18143	PRB1	-0.138234968	0.001349187
18144	FNDC7	0.152208075	0.000410944
18145	NAA11	-0.021441618	0.62071098
18146	C1orf158	0.097889223	0.023553893
18147	CABP2	0.199461613	3.33E-06
18148	RP11-155D18.12	0.107445359	0.012896755
18149	CGB8	0.174266287	5.07E-05
18150	TTC6	0.041979611	0.33247167
18151	DUPD1	0.12844431	0.002917361
18152	ZAR1L	0.252008181	3.40E-09

18153	GNAT1	0.105539595	0.014595942
18154	OR2A2	-0.184890088	1.68E-05
18155	F9	-0.032015423	0.459921407
18156	SLITRK3	0.037612977	0.38525034
18157	NPIPA7	0.10985016	0.011003192
18158	KRT37	0.081067215	0.060959467
18159	KRT35	0.065970168	0.127512235
18160	LCE2D	0.125239285	0.003714412
18161	OR6A2	-0.138523947	0.001317811
18162	SERPINB13	0.115933711	0.007267332
18163	H2AFB1	0.107298571	0.013021134
18164	RAB4B-EGLN2	0.232027377	5.68E-08
18165	PRAC1	0.043564968	0.314520786
18166	RP11-20I23.3	0.011591134	0.789095293
18167	MYBPC1	0.094867526	0.028227479
18168	AC003005.4	0.00507711	0.906733275
18169	LCE3A	0.059605112	0.168614363
18170	RP11-277P12.6	0.141170111	0.001060189
18171	AL022578.1	0.077741198	0.072387522
18172	IRGC	0.192416531	7.39E-06
18173	OR52I1	-0.131215329	0.002357321
18174	ARSH	0.073776071	0.088235848
18175	OCSTAMP	0.121229497	0.004987338
18176	C10orf120	0.148007339	0.000593987
18177	OR5AK2	-0.062240625	0.150531481
18178	OR2T34	-0.096581307	0.025487575
18179	OR10J3	0.131950979	0.002226124
18180	TKTL2	0.016579474	0.702006223
18181	CYLC2	0.080802008	0.061812383
18182	AC017081.1	0.171881001	6.44E-05
18183	POTEF	4.07E-05	0.999250308
18184	GPR78	0.206626751	1.43E-06
18185	NGB	0.002280973	0.95802215
18186	OPTC	-0.072727794	0.092862001
18187	RGPD1	0.162721187	0.00015672
18188	ZBTB8B	0.083377147	0.053934127
18189	OR5W2	0.058930921	0.173491237
18190	DCDC2C	-0.025606937	0.554519258
18191	OR51I1	-0.046370245	0.284342941
18192	OR10G2	0.153136054	0.000378338
18193	BHLHE23	0.04013723	0.354145275
18194	OR11A1	0.026558419	0.539896633
18195	GPR26	-0.164415569	0.000133411
18196	ZBTB9	0.097188582	0.024573349
18197	FLJ20019	0.074121909	0.086750927
18198	RP11-506B6.7	-0.054965873	0.204316121
18199	SPP2	-0.064826882	0.134258397
18200	OR5B2	0.09042193	0.036540785
18201	OR5B12	-0.025043247	0.563272362
18202	SPECC1L-ADORA2A	-0.023404547	0.589089084
18203	RP11-310N16.1	0.056570427	0.191394925
18204	WNT8A	0.12232159	0.004606498
18205	TMEM229A	0.079048963	0.067701123
18206	SMCO1	0.264833072	4.89E-10
18207	LYZL6	0.074104944	0.086823297
18208	FABP12	0.161369715	0.000177998
18209	MYADML2	0.287248987	1.27E-11
18210	MAGEA11	0.038356033	0.37592563

18211	CHRNA2	-0.015983052	0.712241068
18212	NOBOX	-0.02911606	0.501568596
18213	RP6-24A23.6	0.011453712	0.791537857
18214	LY6G6E	0.058733647	0.174937937
18215	PPIAL4E	0.119855779	0.005506599
18216	OR51A7	0.115936212	0.007266064
18217	OR13D1	-0.069890072	0.106363554
18218	KIAA1024L	0.075631726	0.080502278
18219	FGF19	0.030705602	0.478492931
18220	LEUTX	0.101712778	0.018611298
18221	CCDC33	0.012388674	0.774961154
18222	DYTN	0.091102957	0.03514608
18223	FOXD4L4	0.156627141	0.000276054
18224	FAM83C	0.167640314	9.78E-05
18225	C1QTNF8	0.140274325	0.00114168
18226	PSG3	0.079842756	0.064979991
18227	FAM46D	-0.232597881	5.26E-08
18228	LELP1	0.0935548	0.030493941
18229	OR51I2	-0.087090229	0.044060133
18230	EVX2	0.070397787	0.103840059
18231	SPRR2G	0.099802524	0.020954313
18232	EEF1E1-BLOC1S5	-0.134192853	0.001866378
18233	AC112719.2	0.073976147	0.087374312
18234	NLRP13	0.113662956	0.00850301
18235	USP17L2	-0.13213248	0.002194799
18236	GBX1	0.188527561	1.13E-05
18237	BOLL	-0.046113122	0.287024712
18238	APOBEC4	-0.185276155	1.61E-05
18239	FEZF2	-0.032029253	0.45972748
18240	CNTNAP4	0.034997567	0.419176837
18241	LCE6A	0.099888811	0.020843196
18242	IQCF1	0.198833715	3.58E-06
18243	ADH7	0.08940206	0.038717207
18244	CACNG3	0.078076514	0.071161367
18245	LYZL4	0.056977167	0.188216891
18246	CARTPT	0.103979282	0.016130597
18247	OR2T3	-0.127844366	0.003053536
18248	SYNPR	0.061677873	0.154263276
18249	PLA2G2F	0.114691178	0.007921978
18250	IL3	0.047796515	0.269774685
18251	RSPH10B2	0.256814734	1.66E-09
18252	HIST2H3C	0.086987759	0.044310654
18253	PAX1	0.080271115	0.06354935
18254	LY6G6F	0.144441291	0.000806048
18255	C8orf86	0.039929961	0.356638067
18256	IQCF3	0.042789868	0.323216283
18257	KRTAP10-11	0.131926638	0.002230356
18258	OR2A5	-0.122777371	0.004455476
18259	MINOS1-NBL1	0.047885802	0.268879981
18260	LRRC72	0.068353538	0.114296767
18261	OR10A4	-0.087970264	0.041957296
18262	PHOX2B	0.087975228	0.04194568
18263	RP11-447L10.1	0.052262142	0.227499823
18264	SYCN	0.112660608	0.009105694
18265	SFTPC	-0.069934214	0.106142249
18266	TMEM202	0.097535992	0.024063188
18267	SRY	0.021882434	0.613545631
18268	14-Sep	0.057732462	0.18241873

18269	FAM25C	0.101059391	0.019385613
18270	GH2	0.101779137	0.018534188
18271	OR5AP2	0.080886425	0.061539832
18272	NPSR1	0.135934294	0.001624592
18273	CTD-2207O23.3	-0.029271054	0.499292969
18274	MYL1	0.005791718	0.893678859
18275	OPN5	0.037987275	0.380535729
18276	COX7B2	0.096954536	0.024922284
18277	C2orf80	0.09257749	0.032280931
18278	NPIPA8	0.093205125	0.031123331
18279	TARM1	0.104708823	0.015396279
18280	RP11-432B6.3	0.267890363	3.03E-10
18281	IL36A	0.128921869	0.002812941
18282	GPR144	0.097025173	0.024816524
18283	KCNQ2	0.110466537	0.010559385
18284	TSNAX-DISC1	-0.073675687	0.08867067
18285	RP11-546B8.6	0.108747526	0.011838485
18286	CCDC103	-0.084078523	0.051939384
18287	SEBOX	-0.07599415	0.079057902
18288	NLRP10	0.134429981	0.001831621
18289	OR52N1	-0.166029877	0.000114268
18290	TMEM212	0.090830042	0.035699457
18291	CSMD3	0.129592897	0.002671966
18292	PGK2	0.080946607	0.061346133
18293	KRTAP6-2	0.186523046	1.41E-05
18294	CTC-398G3.6	0.026871486	0.535127764
18295	LMX1A	-0.105939363	0.014224026
18296	NKX6-2	-0.049154964	0.256381817
18297	TEX33	0.083851226	0.052578974
18298	AC004824.2	0.028239728	0.514537285
18299	TECTB	-0.069899479	0.10631636
18300	MAS1	0.053590693	0.215884271
18301	RIMBP3C	0.146404638	0.000681917
18302	SCN10A	0.019338062	0.655390516
18303	BARHL2	0.141092656	0.001067017
18304	SUN5	0.100854689	0.019633906
18305	OR10W1	0.004268492	0.921535247
18306	OR4N2	0.087650766	0.042710728
18307	OR2C3	0.038167604	0.378277004
18308	ZPBP2	0.172492646	6.06E-05
18309	C15orf43	0.064835884	0.134204229
18310	SPINK14	-0.19205472	7.69E-06
18311	DEFB133	0.039714264	0.359243914
18312	PRM1	0.106478351	0.013735799
18313	RP11-148K1.1	0.13224373	0.002175797
18314	ZIM3	0.075665586	0.080366436
18315	RGSL1	0.099007097	0.022003114
18316	TPH2	0.062090965	0.151517162
18317	PPIAL4D	0.108254727	0.012229594
18318	OR51Q1	0.040856379	0.345581529
18319	KRTAP19-8	0.046742299	0.280492481
18320	KCNH5	0.103044135	0.017116641
18321	MTRNR2L7	0.073580653	0.089083906
18322	OR10P1	0.071581833	0.098139264
18323	SPPL2C	0.026957752	0.533817422
18324	HNRNPCL2	0.064135476	0.138469743
18325	IL36RN	0.061599147	0.154790873
18326	CSN1S1	0.018430618	0.670588512

18327	CALHM3	0.245606592	8.61E-09
18328	COMMD3-BMI1	-0.153281632	0.000373447
18329	MC3R	-0.017209376	0.691255081
18330	RP13-279N23.2	0.122369672	0.004590351
18331	GNAT3	-0.027063652	0.532211064
18332	PIRT	-0.000498425	0.990823224
18333	GUCY2F	0.065277988	0.131564417
18334	PRDM9	0.145934021	0.000709941
18335	TMEM215	-0.085518633	0.048036184
18336	AC007557.5	0.008324725	0.847660418
18337	SLCO1B3	0.220711559	2.51E-07
18338	SAMD7	0.085201729	0.048873327
18339	CNBD1	0.067992146	0.116228492
18340	SHCBP1L	0.075657594	0.080398481
18341	DEFB134	-0.092878374	0.031721473
18342	KRTAP11-1	0.19148482	8.19E-06
18343	OR8G5	0.02046171	0.63676749
18344	CLCA1	0.026845999	0.535515199
18345	KRT77	0.11535484	0.007566043
18346	H2BFM	-0.005323944	0.902221116
18347	BPIFA1	0.086537302	0.045426203
18348	MS4A13	0.090772465	0.035817145
18349	MAGEB10	0.040010455	0.355668676
18350	PRSS58	0.092946045	0.031596796
18351	POTEM	0.054169877	0.210956046
18352	CTD-3214H19.4	0.136479502	0.001555023
18353	ZFP91-CNTF	-0.054827738	0.20545739
18354	RP11-542C16.2	0.385777223	1.98E-20
18355	CD200R1L	0.056765455	0.189866209
18356	PPP3R2	0.002869822	0.947199439
18357	OR2Z1	0.080925178	0.061415047
18358	SPACA5B	-0.034179722	0.430134993
18359	GLYATL3	0.075328402	0.08172748
18360	GHSR	0.063126349	0.14479752
18361	OR52B2	-0.027848598	0.520381144
18362	ZNRF4	0.135581756	0.001671085
18363	TMEM210	0.197030371	4.40E-06
18364	XAGE5	0.012513004	0.772764309
18365	FAM90A26	0.034730833	0.422732641
18366	PATE2	-0.087222931	0.043737473
18367	DEFB4A	0.025510997	0.556004342
18368	PATE4	-0.089934146	0.037568399
18369	NDST4	0.045713785	0.291223401
18370	GABRG2	0.04885454	0.259303276
18371	ANKRD62	0.005413262	0.900589129
18372	GOLGA6A	0.066812968	0.122709782
18373	WFDC10A	0.106685908	0.013551761
18374	C2orf57	0.108055436	0.012390976
18375	OR11H4	0.083491562	0.053604441
18376	IQCF2	0.133147026	0.002026995
18377	RP11-322N21.2	-0.020153171	0.641859036
18378	BEND2	0.069246293	0.109632757
18379	DIRC1	0.084373048	0.051120285
18380	IFNW1	-0.103257725	0.016886894
18381	SLC36A3	0.081500499	0.059586942
18382	MCHR2	0.090545443	0.036284402
18383	ARGFX	0.050191069	0.246481472
18384	XXcos-LUCA11.5	0.111400596	0.009917077

18385	KRT82	0.097194646	0.024564365
18386	TMEM235	0.110430447	0.010584926
18387	KCNK16	-0.00279713	0.948535001
18388	OPRK1	-0.093621567	0.030375013
18389	CGB2	0.080974469	0.06125663
18390	OTX2	0.074482728	0.085223195
18391	TSGA13	0.088630909	0.040434911
18392	CABS1	0.10524821	0.014872388
18393	MYT1L	0.064201906	0.138060764
18394	SPEM1	0.056473545	0.192157683
18395	CTAGE1	0.017472352	0.686784767
18396	LIN28B	0.184370219	1.78E-05
18397	FAM25G	0.088999178	0.039606735
18398	SPAM1	0.077382373	0.073718696
18399	MS4A12	0.138499467	0.001320443
18400	NBPF6	0.064250986	0.1377592
18401	OR13C5	-0.074200146	0.086417804
18402	LOR	0.157360826	0.000258143
18403	PDX1	0.054618622	0.207193886
18404	FAM216B	0.058010888	0.180314996
18405	DEFB105A	0.049433154	0.253696971
18406	ALPPL2	0.038102111	0.379096385
18407	VAX1	0.107935451	0.012489042
18408	OR10V1	0.123941899	0.004089723
18409	INSM2	0.077922679	0.071721775
18410	POTEE	0.04539834	0.294569019
18411	GPR50	0.026377214	0.542666538
18412	OR5V1	-0.058988803	0.173068457
18413	OR10K1	0.071801187	0.097111037
18414	PCDH8	-0.002768932	0.949053112
18415	CRYGD	-0.0477475	0.270266705
18416	GAST	0.157237479	0.000261076
18417	PSG6	0.06513173	0.132433246
18418	SP9	0.078511163	0.069597301
18419	GPR151	-0.028001056	0.518099233
18420	CPLX4	0.093846401	0.029977447
18421	ODF1	0.186033518	1.49E-05
18422	FOXI3	0.159573416	0.000210488
18423	RESP18	0.083873947	0.052514746
18424	H2BFS	0.033255156	0.442720582
18425	C8orf74	0.112735568	0.009059341
18426	OR1L3	-0.080946946	0.061345045
18427	LCE1E	-0.050242926	0.245993082
18428	CLCA4	0.137028903	0.001487695
18429	SCGB1D4	0.069495487	0.108358042
18430	RP11-831H9.11	-0.005358234	0.901594532
18431	S100A7L2	0.131151203	0.002369085
18432	BARHL1	0.023896677	0.581278849
18433	CRISP1	0.070612424	0.102787621
18434	NPS	-0.158870236	0.000224658
18435	H1FOO	-0.011537964	0.790040097
18436	GKN2	0.003812563	0.929893447
18437	RP11-307N16.6	-0.208609025	1.13E-06
18438	DSG4	0.011085528	0.798092045
18439	PSMB11	0.141591515	0.001023739
18440	RAX	0.120195879	0.005373699
18441	RP11-195F19.29	-0.077430247	0.073539947
18442	GPR12	0.039880128	0.357239045

18443	DBX1	0.106242456	0.013947631
18444	SPAG11A	0.10576655	0.014383764
18445	LHX3	0.049016106	0.257729293
18446	C2orf78	0.078535337	0.069511144
18447	DEFB119	0.127781679	0.003068093
18448	GAGE2A	0.148827287	0.000553187
18449	ISY1-RAB43	-0.109551875	0.011223841
18450	OR10A5	-0.094714519	0.028483972
18451	OR2T29	-0.110272518	0.01069734
18452	AC104534.3	0.110471399	0.010555948
18453	RP11-257K9.8	0.062711906	0.147459399
18454	OR2B2	0.081250037	0.060377184
18455	LACTBL1	0.071568628	0.098201437
18456	VGLL2	0.090467592	0.036445822
18457	OR14K1	-0.20303938	2.19E-06
18458	DDX53	0.108827498	0.011776068
18459	AC010184.1	0.037249352	0.389864432
18460	KIR3DL3	0.101365802	0.019019064
18461	TGM6	0.170125543	7.66E-05
18462	DAOA	0.073160063	0.090931359
18463	ZNF716	0.066762707	0.12299217
18464	CTD-2369P2.12	0.362716531	4.44E-18
18465	PRAMEF20	0.093194355	0.031142891
18466	RAD51AP2	0.088364853	0.041042298
18467	NLRP8	0.114263109	0.008159421
18468	LCE1A	-0.056577328	0.191340679
18469	MTNR1B	-0.06006932	0.165316568
18470	KRTAP5-5	0.089763309	0.037934055
18471	OTUD6A	0.193547171	6.51E-06
18472	POTEJ	0.068808405	0.111901315
18473	TMEM189-UBE2V1	-0.001683193	0.96901689
18474	BTBD17	0.157670756	0.000250908
18475	KRT9	0.00464041	0.914723505
18476	PRLH	0.187871245	1.22E-05
18477	VN1R2	-0.060182967	0.164516629
18478	ASZ1	0.047011452	0.277729078
18479	CLEC2A	0.047866771	0.269070507
18480	LRRC53	-0.096633996	0.025407108
18481	OR1E2	0.151275237	0.000446344
18482	RGR	0.097604329	0.023963922
18483	ALPP	0.197963552	3.95E-06
18484	ZNF645	0.077053668	0.074955581
18485	OR1L4	-0.192805641	7.08E-06
18486	VPREB1	0.150264077	0.000487908
18487	NWD2	-0.08595731	0.046897217
18488	DSPP	-0.147672946	0.000611414
18489	DRD3	0.173361946	5.55E-05
18490	CTD-2501B8.1	0.212310388	7.20E-07
18491	ZAR1	0.017941348	0.678839485
18492	ADAD1	0.05201556	0.229703675
18493	OR56A3	0.034141543	0.43065057
18494	CST9L	0.099946395	0.020769326
18495	SLC22A25	-0.071261961	0.099654184
18496	TAAR9	-0.080533143	0.062687092
18497	OR2D3	-0.092518606	0.0323914
18498	AC002985.3	0.13424536	0.00185863
18499	TOPAZ1	0.049115505	0.256764227
18500	MC5R	0.072301037	0.094800071

18501	PDYN	0.081999948	0.058036646
18502	NKX2-6	0.113296299	0.008719215
18503	NOX3	0.06923545	0.10968849
18504	MS4A5	-0.079898163	0.064793463
18505	ACTL7B	0.124076944	0.004049116
18506	VRTN	0.101935723	0.018353337
18507	HTR1E	-0.053206859	0.21919551
18508	TBC1D3	0.15482238	0.000325176
18509	LBX1	0.179438867	2.98E-05
18510	OFCC1	0.079488198	0.066184094
18511	CGB5	0.195461843	5.25E-06
18512	SPRR2B	0.091016379	0.035320834
18513	KRTAP7-1	-0.050353261	0.244956185
18514	FAM71A	-0.056899204	0.188823023
18515	TAF1L	0.110205471	0.010745383
18516	AC005477.1	-0.175103254	4.66E-05
18517	LHX5	0.080685532	0.062190074
18518	TBX22	0.086327978	0.045952569
18519	PCDH11X	-0.056980702	0.188189444
18520	LCE3D	0.0558464	0.197149168
18521	KLK9	0.127928021	0.003034207
18522	TRIM6-TRIM34	-0.009909469	0.819120608
18523	C1orf141	0.041706274	0.335632068
18524	HTR5A	0.0246466	0.569471148
18525	DKFZp313A047	0.089119764	0.039338695
18526	USP29	0.090221181	0.036960775
18527	UBQLN3	-0.128924776	0.002812316
18528	VCX2	0.055044601	0.203667728
18529	NKX2-4	0.094031909	0.029652786
18530	TBR1	0.071597801	0.098064122
18531	ALX3	0.053672078	0.215186828
18532	GPR148	0.109937965	0.010938977
18533	OR2K2	0.168006304	9.43E-05
18534	AP000304.12	0.041255025	0.340891585
18535	ANKRD30BL	0.048996097	0.257923857
18536	AC104073.2	0.105031878	0.015080593
18537	PAGE3	0.057147535	0.186897334
18538	OR2L2	-0.103994792	0.016114672
18539	CCT8L2	0.105750236	0.014398924
18540	OR2A12	-0.109631375	0.011164653
18541	OR5B21	-0.015564615	0.719452737
18542	WDR87	0.07885583	0.068377137
18543	RXFP3	0.034867471	0.420908927
18544	PPIAL4G	0.104021868	0.016086903
18545	TCL1B	0.032743905	0.44976915
18546	PSAPL1	-0.168393965	9.08E-05
18547	RP1-27O5.3	0.070154919	0.105041204
18548	KRT83	-0.015398321	0.722325749
18549	RAX2	0.049870866	0.249512178
18550	CT83	-0.004260322	0.921684958
18551	OR4A47	-0.052675157	0.223842199
18552	GAGE1	-0.012209366	0.778132594
18553	SPAG11B	0.059009844	0.17291496
18554	GCG	0.008062624	0.852400952
18555	MAGEB18	0.000592768	0.989086319
18556	OR1J2	0.041143738	0.342196727
18557	LRTM2	0.156444612	0.000280687
18558	TMEM247	0.004503092	0.917237816

18559	NHLH2	0.027471	0.526055012
18560	PATE1	-0.068254782	0.114822123
18561	OR7G2	0.148682882	0.000560178
18562	RP11-540D14.8	0.060534852	0.162058214
18563	EPPIN	0.01817536	0.674888282
18564	OR7C2	0.100047982	0.020639564
18565	TTLL8	0.060612008	0.161522884
18566	OR51B6	-0.144149178	0.00082621
18567	ZNF705G	0.023378604	0.589502137
18568	CLRN1	-0.057425456	0.184759386
18569	RGPD4	-0.015106742	0.727372679
18570	CTD-2132N18.3	-0.018689576	0.666237371
18571	SYCP1	0.080031049	0.064347895
18572	OR2G3	0.054473735	0.208403236
18573	RP11-178C3.1	0.07837338	0.07009003
18574	HTR2C	0.149286453	0.000531492
18575	VSX2	0.062502384	0.148819231
18576	AMBN	0.142046764	0.000985665
18577	TFDP3	0.049979927	0.24847702
18578	CFHR2	-0.056105699	0.195074034
18579	PAPOLB	-0.038902486	0.369157588
18580	NOTO	0.057990465	0.180468697
18581	PGLYRP3	0.19468809	5.73E-06
18582	PRLHR	0.083478787	0.053641167
18583	DCAF8L2	0.039582628	0.360840057
18584	FAM24A	-0.137854261	0.001391571
18585	OR4D1	0.172061881	6.32E-05
18586	RP11-637O19.3	-0.046490429	0.283095253
18587	TAS2R41	-0.109453813	0.01129723
18588	HTR1A	0.060786021	0.160320426
18589	AL645922.1	0.042112193	0.330945644
18590	TXNDC8	-0.003088581	0.943181153
18591	RP11-463D19.2	-0.106836285	0.013419786
18592	TEX36	0.099468372	0.021389492
18593	LUZP4	0.050906208	0.239805859
18594	TMEM239	0.093469149	0.03064709
18595	OR10H1	0.195085039	5.48E-06
18596	KRTAP21-2	0.098383389	0.022857062
18597	GOT1L1	0.109617986	0.011174602
18598	OR4D5	0.090971709	0.035411287
18599	TAS2R8	-0.055871337	0.196948902
18600	FOXD4L5	0.140269964	0.00114209
18601	XXbac-BPG116M5.17	-0.111479309	0.009864569
18602	LGALS16	0.091616923	0.034123744
18603	OR7A17	0.082366411	0.056920524
18604	HZGJ	-0.112299476	0.009331977
18605	ASB10	0.040312216	0.352049297
18606	DMRTC1B	-0.059583117	0.168771835
18607	RP11-152F13.10	0.134815259	0.001776412
18608	OR8K1	0.075864283	0.079573027
18609	LYZL2	0.065167533	0.132220153
18610	KRT26	0.049222723	0.25572606
18611	OR2AK2	-0.163657415	0.000143406
18612	OR1L6	-0.137710108	0.001407937
18613	AC012005.3	0.018615248	0.66748514
18614	WFDC9	0.038990228	0.368077949
18615	ZNF735	0.059038225	0.172708084
18616	IQCJ	-0.094553822	0.028755508

18617	OR51M1	-0.114832379	0.007845021
18618	OR2B3	0.047373866	0.274037459
18619	C11orf44	0.083791286	0.052748728
18620	FSHB	-0.054916677	0.204722047
18621	TRIM49B	0.051992674	0.229908986
18622	CH17-270A2.2	0.045656976	0.291824037
18623	GPR149	0.084402078	0.051040136
18624	AL356135.1	-0.067536945	0.118697949
18625	OR4C5	0.118353322	0.006129799
18626	NPY2R	-0.018655082	0.666816326
18627	ZCCHC13	0.135431008	0.001691336
18628	TDRD15	-0.094988715	0.028025736
18629	IAPP	-0.022779541	0.599077108
18630	IL25	0.078154107	0.070880063
18631	OR4D9	0.109374223	0.011357107
18632	FABP9	0.075514138	0.080975469
18633	TNP1	-0.067042045	0.121429114
18634	OPALIN	0.025421598	0.557389904
18635	RP11-514P8.7	0.038942544	0.368664446
18636	ATP1B4	-0.139570192	0.001209747
18637	TEX35	0.13217521	0.002187483
18638	OR4D11	0.097279552	0.024438867
18639	CDX2	0.152011042	0.000418196
18640	PRAMEF18	0.124147008	0.004028192
18641	OR2V1	0.044506072	0.304171041
18642	KRTAP12-3	0.134235859	0.00186003
18643	NKX2-1	0.133339352	0.001996541
18644	ZIC3	-0.017661613	0.683574285
18645	OR5M11	0.079528689	0.066045662
18646	KCNK4	0.131274554	0.002346503
18647	CHRNB3	0.124239362	0.004000761
18648	OR2L3	-0.14146555	0.001034512
18649	OR10A3	-0.003576669	0.934221006
18650	SSX7	0.057513592	0.184085167
18651	OR10G3	0.150400193	0.000482111
18652	OR6M1	0.075761502	0.079982642
18653	TPTE	0.127991787	0.003019549
18654	OR7E24	0.058289082	0.178231024
18655	CTC-554D6.1	-0.083227496	0.054367895
18656	MAGEB16	0.058096676	0.179670439
18657	OR1L1	-0.129194842	0.002754795
18658	OR7G1	0.144829805	0.000779938
18659	CYP4F8	0.063946776	0.139636563
18660	OR10J5	0.061209432	0.15742278
18661	ZC2HC1B	-0.0557742	0.19772983
18662	C1orf137	0.080806404	0.061798162
18663	FOXD4L3	0.227957624	9.78E-08
18664	GPHB5	0.110155687	0.01078118
18665	CRCT1	0.176899919	3.88E-05
18666	SMIM11	0.059752523	0.167561848
18667	NEUROG2	0.10363085	0.016492016
18668	OR6J1	0.049233546	0.255621429
18669	GML	-0.061420143	0.155995596
18670	OR6K3	0.067719944	0.117700298
18671	SIX6	-0.055050184	0.203621805
18672	SERPINB12	0.074636812	0.084577435
18673	PRR9	0.053343635	0.218011431
18674	BHLHA9	0.016267705	0.707349726

18675	OR6C65	0.049241456	0.255544969
18676	KRT71	0.051624501	0.233229778
18677	TBC1D3K	-0.000810944	0.9850698
18678	PRR23A	0.106959103	0.013312838
18679	OR1C1	-0.138996523	0.001267944
18680	FGF6	0.148184455	0.000584944
18681	OR7D4	0.123787383	0.004136636
18682	SKOR2	0.053085836	0.220247041
18683	SMIM21	0.047425609	0.273513138
18684	PTH2	0.030448246	0.482189328
18685	SLC6A5	0.020908237	0.629429124
18686	CEACAM18	0.136440346	0.001559926
18687	HRH3	0.138196753	0.001353387
18688	KCNG4	0.105052282	0.015060847
18689	TGIF2LX	0.149441074	0.000524366
18690	LRRC24	-0.068076281	0.115776503
18691	FAM156B	0.342893457	3.31E-16
18692	C14orf177	0.157474788	0.00025546
18693	OR9A2	-0.107008335	0.01327018
18694	SPRR4	0.032738405	0.44984533
18695	MYF5	-0.106460921	0.013751353
18696	PRR27	0.056274103	0.193734903
18697	PATE3	-0.104080554	0.016026861
18698	DUSP21	0.103927919	0.016183434
18699	PABPC1L2A	-0.021445185	0.620652853
18700	MYH6	0.178489165	3.29E-05
18701	ZNF679	0.053199644	0.219258091
18702	OR5F1	0.07484758	0.083700518
18703	SI	0.05745418	0.184539453
18704	GOLGA8J	0.160317206	0.000196413
18705	PGA4	-0.018104612	0.676081896
18706	FUT5	0.106041239	0.014130591
18707	RBP3	-0.02856879	0.509647264
18708	DEFB126	0.043530595	0.314903118
18709	HYPM	0.05103998	0.238571374
18710	OR2G2	0.047288229	0.274906748
18711	OR11G2	0.063161625	0.144572656
18712	SMCP	0.082111889	0.057693804
18713	GOLGA8F	0.061858534	0.153057692
18714	RP4-559A3.7	0.03658544	0.398374905
18715	CARD18	0.063881591	0.140041381
18716	CTD-2135J3.4	-0.062792446	0.14693921
18717	LMAN1L	0.159046361	0.000221027
18718	OR14J1	-0.056507495	0.19189014
18719	PRM3	0.100242544	0.020393007
18720	KRTAP8-1	0.035142027	0.417258391
18721	AC006486.9	0.059784722	0.167332601
18722	CGB	0.199179569	3.44E-06
18723	BLACE	0.133107248	0.002033347
18724	SPANXD	0.078715072	0.068873302
18725	GFRAL	0.05530151	0.201562264
18726	TSSK1B	0.071625076	0.097935877
18727	ARL2-SNX15	0.076737538	0.076161039
18728	TAS2R9	-0.089909787	0.037620353
18729	SLCO1B7	0.096363498	0.025822553
18730	DEFB116	0.114977877	0.007766421
18731	CCDC140	0.068701948	0.112458373
18732	PDHA2	-0.082675576	0.055992815

18733	PSG11	0.052816253	0.222602331
18734	KRT3	0.065578162	0.12979507
18735	BMP10	0.063305104	0.143660808
18736	C10orf53	0.127356885	0.003168413
18737	TPRX1	0.094699947	0.028508503
18738	KRTAP10-1	0.079153877	0.067336204
18739	EIF4E1B	0.107888724	0.012527418
18740	RNASE9	-0.005935805	0.891050038
18741	RIMBP3B	0.074397525	0.085581978
18742	DYNAP	0.062645507	0.14788931
18743	GABRA1	0.051250119	0.236641215
18744	OR4F15	-0.070518586	0.103246692
18745	IFNA8	0.037251782	0.389833491
18746	LRRC10	0.170086383	7.69E-05
18747	GABRR3	0.048444997	0.263322777
18748	KRTAP1-4	-0.004948057	0.909093601
18749	PLSCR5	0.125775353	0.003568669
18750	RP11-47I22.4	-0.156480464	0.000279772
18751	ZP4	0.028874977	0.50511906
18752	TAS2R40	-0.088170207	0.041491525
18753	DAZ1	-0.044275493	0.306685719
18754	ANKRD66	-0.098660962	0.022473542
18755	POTEH	0.038469992	0.374507936
18756	OR6B1	-0.179154672	3.07E-05
18757	PRR23C	0.103058239	0.017101385
18758	GOLGA8G	0.08768208	0.042636384
18759	GFY	0.182495543	2.17E-05
18760	AC009491.2	0.054432022	0.208752349
18761	TPD52L3	0.091835305	0.033697092
18762	ATOH1	0.065960259	0.127569559
18763	OR6C75	0.137865794	0.001390269
18764	PRKACG	0.053894402	0.213289827
18765	TISP43	0.058086341	0.179748002
18766	OR8A1	0.114120022	0.008240194
18767	IFNL3	0.151216687	0.000448658
18768	SPANXN1	0.168595745	8.91E-05
18769	OR12D3	-0.040416785	0.350800525
18770	SPACA7	0.102965234	0.017202199
18771	ABCC12	0.049531664	0.252750929
18772	ROPN1	0.111426484	0.00989978
18773	KCNA10	0.073920132	0.087614827
18774	GPX5	0.122147426	0.004665417
18775	SLC35D3	0.048888151	0.258975294
18776	HMX1	0.022439106	0.604549486
18777	OTOR	0.048882548	0.259029953
18778	OR11H6	0.030849808	0.476428468
18779	OR13G1	-0.156895206	0.000269379
18780	DNAJC25-GNG10	-0.080865774	0.061606415
18781	OR4F6	0.080511079	0.062759322
18782	OPRM1	0.047383321	0.273941599
18783	MRGPRX2	0.084780693	0.050004407
18784	OR52N5	0.040417357	0.350793703
18785	OR52K1	-0.039985724	0.35596634
18786	RFPL1	0.044393978	0.305391823
18787	TRIM60	0.055342833	0.201225094
18788	GJE1	-0.097839051	0.02362566
18789	MSGN1	0.17591131	4.29E-05
18790	PRPS1L1	0.098613405	0.022538853

18791	DEFB129	-0.167436736	9.97E-05
18792	OR10D3	0.04240542	0.32758667
18793	SPATA19	0.124139641	0.004030387
18794	MT4	0.11576236	0.007354634
18795	ANHX	0.109504131	0.011259519
18796	OR52K2	-0.082301255	0.057117652
18797	KNCN	0.043706681	0.312947709
18798	OTOL1	0.146329051	0.000686348
18799	TEX13A	0.067586774	0.118425646
18800	GJA10	0.071365542	0.099161599
18801	SCRT2	0.085048784	0.049281699
18802	RP1-37E16.12	0.291568636	6.06E-12
18803	OR56A5	0.111975103	0.009539478
18804	OOSP2	0.033532973	0.438916912
18805	LACRT	0.070912619	0.101329871
18806	RHOXF2B	-0.021167087	0.625191666
18807	KRTAP23-1	0.103495328	0.016634494
18808	LY6G6D	0.117839568	0.006357004
18809	KRTAP6-3	0.088670091	0.040346106
18810	IFNA7	-0.022422099	0.604823446
18811	OTOP3	0.056118869	0.194969058
18812	FSBP	-0.127157207	0.003216592
18813	FGF4	0.065431025	0.13066004
18814	CCDC166	0.184045154	1.84E-05
18815	OR6C68	0.021339915	0.622369278
18816	AC011155.1	-0.112100309	0.009458903
18817	GABRA6	0.05866321	0.175456658
18818	RP11-598P20.5	0.020966202	0.628479146
18819	OR8H2	0.056916306	0.188689938
18820	TAS2R39	-0.069790284	0.106865186
18821	EDDM3B	0.011627324	0.788452381
18822	OR1A1	0.068877656	0.111540107
18823	ACTL7A	-0.055661836	0.198635992
18824	LCN9	0.019994752	0.644479824
18825	ARHGAP19-SLIT1	0.063578119	0.141937888
18826	ACTRT1	0.090762323	0.035837909
18827	SCGB1D1	0.050575463	0.242877302
18828	HDGFL1	0.206471749	1.46E-06
18829	TNP2	0.058957884	0.1732942
18830	TMPRSS11B	0.085086389	0.04918103
18831	OR7G3	0.170112192	7.67E-05
18832	PAX7	0.082236188	0.057315081
18833	KRTAP3-3	0.034713716	0.422961426
18834	OR13C2	0.014373449	0.740117039
18835	KIF2B	0.10964356	0.011155605
18836	SSTR4	-0.047541593	0.272340331
18837	OR56A1	0.038844936	0.369866784
18838	TCP10	-0.052251426	0.227595285
18839	BMP15	-0.028740441	0.507106108
18840	LIPK	-0.049529327	0.252773347
18841	USP26	0.067954412	0.116431659
18842	LCE1B	0.071271059	0.099610843
18843	RP11-468E2.1	0.019337507	0.655399763
18844	ESX1	0.12510331	0.003752227
18845	MIA-RAB4B	0.196094728	4.89E-06
18846	ATP6V1G2-DDX39B	0.118107451	0.006237608
18847	PRAMEF9	0.081776394	0.058726376
18848	OR14A2	-0.202539436	2.33E-06

18849	LALBA	0.158883683	0.000224379
18850	KRTAP10-10	0.060401223	0.162988522
18851	MRGPRX4	0.092027342	0.03332568
18852	KRTAP12-2	0.075240928	0.082083601
18853	MMD2	0.166075972	0.000113761
18854	POTEG	0.063609899	0.141738365
18855	RP11-886H22.1	0.007299456	0.866233794
18856	TSPY2	-0.071360165	0.09918712
18857	PPIAL4F	0.087205903	0.043778766
18858	OR6V1	-0.024427313	0.572912045
18859	PAX4	0.104310985	0.015793
18860	OR6F1	-0.108049756	0.012395603
18861	USP17L1	-0.027622281	0.52377805
18862	OR2V2	0.088310737	0.041166778
18863	OR52L1	0.101292511	0.019106186
18864	PROL1	0.032165574	0.457818467
18865	KRTAP27-1	0.051487023	0.234478436
18866	ZSWIM2	0.104096555	0.016010524
18867	OR13C3	-0.028286628	0.513838842
18868	OR51D1	0.041303221	0.340327331
18869	SPATA31D1	0.156422376	0.000281257
18870	KRTAP13-1	0.060586499	0.161699726
18871	OR13C8	0.029967963	0.489128837
18872	OR6B2	0.078365285	0.07011907
18873	OR9Q2	-0.065393459	0.130881591
18874	KRT28	0.06688228	0.122321183
18875	TRIM64C	0.051692316	0.232615573
18876	BPIFA3	0.057968174	0.180636566
18877	RP11-330H6.5	0.009359583	0.828998538
18878	FOXD4L6	0.160826443	0.000187291
18879	SMR3B	-0.075753844	0.080013229
18880	CH507-9B2.2	0.000972286	0.98209981
18881	RP11-761B3.1	0.069331544	0.109195347
18882	ZNF705B	-0.022353874	0.605923049
18883	S100A7A	0.027938171	0.51903984
18884	GPR119	0.014389742	0.739833087
18885	OR2T33	0.037375863	0.388255333
18886	OR8D4	0.049619155	0.251912772
18887	NMS	0.062696182	0.147561122
18888	SNTN	0.046209282	0.28601978
18889	NXPH1	0.007611933	0.860564796
18890	MC2R	0.010744442	0.804176537
18891	CRYGC	0.108003283	0.012433517
18892	BPIFB6	0.129861233	0.002617417
18893	OR52E2	0.069966711	0.105979556
18894	MUC21	0.177210874	3.76E-05
18895	OR2F2	0.05888752	0.173808747
18896	DCAF8L1	-0.059057193	0.172569917
18897	KRTAP12-4	0.072040461	0.095999257
18898	PRR32	0.037954534	0.380946713
18899	AC104389.1	-0.012517578	0.772683509
18900	RAD51L3-RFFL	-0.042815393	0.322927465
18901	POTEI	-0.008083405	0.852024903
18902	KRT74	0.140691466	0.001103039
18903	CHRM2	0.012819818	0.767350858
18904	IFNA6	0.036054448	0.405261153
18905	CH507-9B2.4	0.011986297	0.782083175
18906	ACTL9	0.171195757	6.89E-05

18907	OR2T6	0.087230414	0.043719338
18908	KRTAP26-1	-0.023550317	0.586770652
18909	OC90	0.047742415	0.270317779
18910	PRAMEF19	0.116175909	0.007145515
18911	KRTAP3-1	0.123889652	0.004105532
18912	PRR23B	0.12202447	0.004707421
18913	SPO11	0.046905518	0.278814492
18914	KRTAP13-2	0.07030586	0.104293414
18915	IFNA13	0.022970342	0.596019869
18916	MUC7	-0.029555358	0.495133054
18917	SMYD1	0.09412568	0.029489827
18918	GSC2	0.063130574	0.144770572
18919	ADIRF	0.047845575	0.269282825
18920	TRIM49C	0.039474765	0.36215124
18921	OR6C76	0.045904942	0.289208438
18922	CCDC172	0.023652159	0.585153379
18923	C4orf17	0.137374892	0.001446681
18924	IFNA16	-0.035015111	0.418943567
18925	OR5B17	-0.068965511	0.111083188
18926	TRIM48	0.03282367	0.44866528
18927	KLHL40	0.110057784	0.010851886
18928	KRTAP19-6	0.062137525	0.151209989
18929	HIST2H4B	0.066330138	0.125443509
18930	SPANXN4	0.03638826	0.400923822
18931	TAS1R2	0.107461455	0.012883181
18932	C7orf62	0.045305492	0.295558643
18933	PSG7	0.005048731	0.907252248
18934	TBC1D28	0.074888844	0.083529697
18935	CXorf66	0.080392055	0.063150166
18936	AC104057.1	0.053448505	0.217106678
18937	IL17A	-0.01266637	0.770056904
18938	TBC1D3G	0.170096587	7.69E-05
18939	OR8B3	-0.008819491	0.838726867
18940	RP11-944L7.5	-0.065576948	0.12980219
18941	KRTAP5-2	0.043157197	0.319076084
18942	SULT1A4	0.166629813	0.000107836
18943	ANKRD20A2	0.038412685	0.375220442
18944	NEUROG1	-0.010170083	0.814448916
18945	PRNT	0.076681801	0.0763752
18946	GPX6	0.043573165	0.31442965
18947	SPATS1	0.075694959	0.080248746
18948	RP3-468K18.7	0.1307583	0.002442344
18949	RP11-5A19.5	0.17580171	4.34E-05
18950	FAM231C	-0.047218215	0.275618837
18951	HIST2H3PS2	-0.036647563	0.397573866
18952	NKX1-2	0.08240142	0.056814835
18953	CTD-3214H19.16	0.114154127	0.008220877
18954	OR1A2	0.066326605	0.125463687
18955	RP11-400G3.5	-0.08982859	0.037793975
18956	MAGEB3	0.064739881	0.134782823
18957	OR1D2	0.06745283	0.11915873
18958	TAS2R7	-0.056046769	0.195544232
18959	NPVF	-0.007263445	0.866887568
18960	UBTFL1	0.04658559	0.282109972
18961	KRTAP3-2	0.080199052	0.063788195
18962	KRTAP4-1	0.011059296	0.798559558
18963	KRT38	0.179138059	3.08E-05
18964	OR2L5	-0.105441364	0.014688628

18965	DNAJB8	0.046932929	0.278533358
18966	RNASE11	0.046081784	0.287352721
18967	AC087651.1	0.041387952	0.339336809
18968	OR51T1	-0.053690787	0.215026726
18969	RP11-101E3.5	-0.095372557	0.027394913
18970	KRTAP10-2	-0.032070006	0.459156321
18971	PABPC1L2B	-0.018307967	0.672653209
18972	CYP2C19	-0.066560831	0.124131504
18973	PRAMEF2	0.048888295	0.258973893
18974	ADAM30	0.129346985	0.002722864
18975	PRAMEF10	0.028379017	0.512464422
18976	GPR101	0.004805822	0.911695942
18977	OR51F2	0.153360404	0.000370826
18978	C7orf72	0.165551326	0.000119655
18979	RP11-77K12.7	0.067975166	0.11631988
18980	OR11L1	0.087503045	0.043062917
18981	OR5M10	0.030122476	0.486890477
18982	TRIM49	0.061030864	0.158639961
18983	XIRP2	0.123278053	0.004294746
18984	PRM2	0.081320777	0.060153118
18985	PYDC2	0.117901839	0.006329068
18986	PRR21	0.119112959	0.005807206
18987	FAM181A	0.248546264	5.64E-09
18988	CTD-3222D19.2	-0.190399319	9.24E-06
18989	MUC5AC	0.053444084	0.217144763
18990	AADACL2	0.018550511	0.66857263
18991	OR2Y1	0.106806343	0.013445973
18992	CSH2	0.078213788	0.070664318
18993	FOXR2	0.156974434	0.000267436
18994	GPR139	0.143025591	0.000908188
18995	RP11-73M18.2	-0.143325489	0.000885602
18996	KCNK18	0.021257318	0.623717461
18997	MGAT4D	0.011587743	0.789155535
18998	HHLA1	0.041865025	0.333794203
18999	OR51G2	0.049279569	0.255176817
19000	DEFB105B	0.04515267	0.297192328
19001	LGALS13	0.082708926	0.055893495
19002	OR1M1	0.162859069	0.000154689
19003	KRTAP1-3	0.107678234	0.012701582
19004	SLC32A1	0.076493246	0.077103329
19005	OR5AU1	0.089372455	0.038781992
19006	OR52E6	0.076818842	0.075849517
19007	AC234582.2	-0.098753492	0.022346939
19008	TECRL	-0.034374044	0.427516394
19009	DEFB121	0.049329946	0.254690751
19010	OR6C2	0.104159309	0.015946595
19011	KRTAP21-1	0.043644373	0.313638719
19012	OR52J3	0.024595154	0.570277514
19013	OR4D10	0.070501076	0.103332535
19014	OR5M8	0.042267646	0.329162135
19015	PRDM13	0.215345353	4.95E-07
19016	RP11-514P8.6	0.112536849	0.009182684
19017	BIRC8	0.107830035	0.012575765
19018	CPXCR1	0.186602789	1.40E-05
19019	OR10J1	0.053895546	0.213280099
19020	CCER1	0.034687665	0.423309754
19021	KRTAP24-1	-0.071571846	0.098186283
19022	GIMAP1-GIMAP5	-0.033818609	0.435025793

19023	OR1S2	-0.107321772	0.013001405
19024	MAGEB5	0.074783867	0.083964822
19025	OR52E5	0.078187316	0.070759948
19026	OR5K1	-0.023815941	0.58255684
19027	GRXCR1	0.09395615	0.029785008
19028	SPZ1	0.042973377	0.321143587
19029	FAM71B	0.075655233	0.080407949
19030	OR8B12	0.116008929	0.007229302
19031	RP1-4G17.5	0.039160116	0.36599311
19032	OR51V1	0.083047637	0.054893057
19033	CYP11B1	0.039772625	0.358537675
19034	KCNC2	-0.044961368	0.299245834
19035	TLX1NB	0.183609295	1.93E-05
19036	RP1-138B7.6	0.037594235	0.385487342
19037	CTD-2105E13.6	0.039006741	0.367874979
19038	SMR3A	0.083718442	0.052955645
19039	OR4K14	0.184943669	1.67E-05
19040	DEFB128	0.011752053	0.786237715
19041	MRGPRX1	-0.019182893	0.657979467
19042	OR6C74	0.022521785	0.603218389
19043	GPR6	0.059444624	0.169765875
19044	OR5B3	-0.069580656	0.107925062
19045	TRIM64B	0.061375692	0.156295853
19046	NOXO1	0.094166609	0.02941894
19047	AC084219.2	-0.064175821	0.138221249
19048	FAM231D	0.082839577	0.055505817
19049	OR4C3	-0.048361395	0.264148543
19050	OR2T7	-0.055507122	0.199888633
19051	EPPIN-WFDC6	0.065176826	0.13216489
19052	DEFB103B	0.054614528	0.207227982
19053	KRTAP20-4	0.108464781	0.01206151
19054	T	0.067563011	0.118555443
19055	RP11-345J4.3	-0.024832258	0.56656565
19056	OR8I2	0.055246725	0.202009917
19057	OR1I1	0.146303187	0.00068787
19058	OR8B2	0.050660916	0.24208111
19059	CYLC1	-0.039890445	0.35711457
19060	OR10H2	0.109968506	0.01091672
19061	SCP2D1	0.010472709	0.809032373
19062	RP11-548K23.11	-0.105715248	0.014431487
19063	INS-IGF2	-0.111980006	0.009536311
19064	OR5AN1	-0.08975014	0.037962365
19065	LCE1F	-0.041663439	0.336129072
19066	RGS21	-0.14951912	0.000520803
19067	OR4E2	0.033335271	0.441621786
19068	RP11-229P13.27	0.057337288	0.185435666
19069	GOLGA6C	0.115142231	0.007678481
19070	HIST1H4G	0.112337796	0.009307731
19071	KRTAP15-1	0.156632429	0.000275921
19072	OR5H2	-0.053727043	0.214716706
19073	SSX4B	0.080239338	0.063654582
19074	PRAMEF12	0.095330296	0.027463763
19075	OR13F1	0.107162026	0.013137782
19076	CFC1	0.038723295	0.371368573
19077	TMCO5A	-0.04081649	0.346053057
19078	RBMXL3	-0.137571886	0.001423795
19079	KRTAP12-1	0.073528377	0.089311876
19080	LCE1D	-0.045816691	0.290137518

19081	CDX4	0.091294756	0.034761562
19082	PRAMEF14	0.063747196	0.140878846
19083	RP11-302M6.4	-0.040127597	0.354260884
19084	RGPD6	0.029787543	0.491749484
19085	F8A2	0.043968427	0.310055824
19086	AC024592.12	0.101266065	0.019137706
19087	TRIM51	0.063674358	0.141334338
19088	LCE2A	0.072161406	0.095441162
19089	OR10T2	-0.002968638	0.945384164
19090	KRTAP10-3	0.045842282	0.289867896
19091	BPIFB3	0.118863026	0.005911619
19092	MRGPRG	0.015691107	0.717270019
19093	OR4F21	-0.0153612	0.722967609
19094	INS	0.041178391	0.341789991
19095	OR5P2	0.063639426	0.141553179
19096	OR4K1	0.074769208	0.08402573
19097	LRRC30	0.088212511	0.04139354
19098	OR6N1	0.017782457	0.681527344
19099	TCHHL1	0.095253384	0.027589447
19100	OR2M7	0.065110135	0.132561902
19101	OR4B1	-0.097493044	0.024125756
19102	OR5P3	0.005732267	0.894763868
19103	FTMT	0.098795441	0.022289747
19104	OR14A16	-0.171579021	6.64E-05
19105	OR52E4	0.051664468	0.232867662
19106	OR10G7	-0.027077113	0.532007063
19107	IL1F10	0.008345797	0.847279539
19108	DEFB110	0.02276626	0.599290165
19109	KPRP	0.050789551	0.240886055
19110	OPN1LW	0.075211213	0.08220486
19111	IFNA17	-0.009272303	0.830568929
19112	PRAMEF4	0.121456834	0.004905796
19113	DEFB115	0.003304343	0.939219312
19114	AMY1B	-0.112980795	0.008909165
19115	OTOP1	0.12735744	0.00316828
19116	SSX2B	0.087667846	0.042670163
19117	OR2AJ1	0.058760925	0.174737361
19118	AL133475.1	0.015120702	0.727130779
19119	OR6N2	0.108895774	0.011723012
19120	RP11-290H9.2	-0.014764784	0.733306668
19121	OR13C9	-0.099469491	0.021388022
19122	OR4K5	0.019347883	0.655226805
19123	KRTAP4-4	0.11801219	0.006279833
19124	CH17-335B8.4	0.127567987	0.003118192
19125	OR5A2	0.047834293	0.269395881
19126	NPBWR2	0.157994843	0.000243546
19127	DMRTB1	0.193124394	6.83E-06
19128	RP1-309K20.6	-0.062369548	0.14968629
19129	KRTAP2-2	0.000562117	0.989650621
19130	OR56A4	-0.070626975	0.102716577
19131	FAM47C	0.072736183	0.092824222
19132	AC073107.1	0.114156145	0.008219736
19133	CSN2	-0.010117437	0.815392105
19134	PRAMEF25	0.057892065	0.181210591
19135	CNGA2	-0.022625908	0.601543933
19136	OR4M1	0.043326976	0.317174236
19137	KRT76	0.165439629	0.000120946
19138	HES3	0.125539398	0.003632168

19139	OR52A1	0.079060853	0.067659684
19140	POTEC	0.108334926	0.012165177
19141	OR5AC2	0.093200519	0.031131695
19142	KRTAP9-6	0.053656473	0.215320434
19143	AADACL3	0.095686994	0.026887295
19144	OR4S1	-0.040654355	0.347973864
19145	RP11-215A19.2	-0.013850169	0.749255378
19146	SERPINB11	0.061147016	0.157847423
19147	OR8D1	0.071698199	0.097592721
19148	CLDN17	-0.058715283	0.175073066
19149	OR4D2	0.07485141	0.083684648
19150	ZNF705D	0.012403814	0.774693527
19151	RP11-529K1.3	-0.054197254	0.21072512
19152	SSX2	0.036924813	0.39401073
19153	OR4D6	0.072182471	0.095344227
19154	LCE3C	0.085825608	0.04723676
19155	CTD-2192J16.24	-0.034368207	0.427594919
19156	XXbac-B562F10.12	0.09226127	0.032877969
19157	OR2M4	0.152742446	0.000391862
19158	OR8J1	0.087996155	0.041896736
19159	MAGEB6	0.03727711	0.389511032
19160	CLDN25	0.096168728	0.026125306
19161	RP11-1099M24.7	0.063998179	0.139317968
19162	CTD-3074O7.11	0.066077413	0.126893156
19163	CT45A5	-0.003325799	0.938825427
19164	KRTAP4-11	0.094931036	0.0281216
19165	OR5J2	0.179399237	3.00E-05
19166	XXbac-BPG32J3.19	0.08961917	0.038244907
19167	ZNF479	0.09359881	0.030415503
19168	OR10G4	-0.053295739	0.218425542
19169	OR11H2	0.215099185	5.10E-07
19170	DEFB4B	-0.023801985	0.582777881
19171	OR6Y1	0.027737066	0.522053768
19172	LCE3E	0.001280004	0.976436045
19173	KRTAP13-4	0.061582139	0.154905037
19174	OR2J2	0.040857412	0.345569324
19175	LRIT1	0.122766677	0.004458968
19176	OR52I2	0.069522592	0.108220096
19177	OR6B3	0.116108433	0.007179268
19178	FAM231A	0.152321814	0.000406811
19179	LIMS3	-0.010975746	0.800049088
19180	RP11-457D20.2	0.156249602	0.000285717
19181	SOX14	0.1654613	0.000120694
19182	PRAMEF8	-0.020992881	0.628042128
19183	GSX1	0.146870716	0.000655176
19184	TAAR2	0.083272056	0.054238435
19185	OR5K4	-0.066211001	0.126125277
19186	OR2J3	0.10191378	0.018378587
19187	OR4K13	-0.019199552	0.657701309
19188	OR9G1	0.071614047	0.097987716
19189	KRTAP10-6	0.080470223	0.062893257
19190	OR52M1	0.059175558	0.171709606
19191	KRTAP9-4	0.128570427	0.002889448
19192	RGPD5	-0.051768774	0.231924463
19193	OR9K2	0.095450795	0.027267844
19194	GOLGA6D	0.027357083	0.527772919
19195	PPP1R3A	-0.017577775	0.684995757
19196	AQP12A	-0.043660591	0.313458763

19197	HTN1	0.068795906	0.111966604
19198	OR10A7	0.038842582	0.369895813
19199	OR8B8	-0.030647595	0.479324734
19200	CT47B1	0.102538695	0.017671236
19201	GDF2	0.097972855	0.023434686
19202	TCP11X2	0.014745365	0.733644136
19203	OR2T4	-0.004021125	0.926069033
19204	OR4Q3	0.111148804	0.010086712
19205	TRIM43	0.062668537	0.147740092
19206	OR2T1	0.037853019	0.382222713
19207	CYP11B2	0.142140994	0.000977949
19208	HNRNPCL3	0.067948572	0.116463127
19209	KRTAP19-7	-0.078397221	0.070004569
19210	OR6X1	-0.007107587	0.869718141
19211	NEU2	0.05193588	0.230419046
19212	C1orf68	0.176351102	4.10E-05
19213	FTHL17	0.188950547	1.08E-05
19214	LCE2C	-0.078612117	0.069238079
19215	HIST2H3A	-0.004802792	0.911751385
19216	OR10S1	-0.021071826	0.626749686
19217	MAGED4B	0.094057424	0.029608369
19218	HTN3	0.019140129	0.658693686
19219	POM121L7	0.116445316	0.007012163
19220	AP005242.1	0.107216808	0.013090873
19221	OR6C70	-0.0163872	0.705299955
19222	TBC1D3F	0.148820541	0.000553512
19223	HMGB4	-0.0019542	0.964031505
19224	OR8U1	0.049419438	0.25382888
19225	FAM47B	0.180597328	2.64E-05
19226	TRIM77	0.088447032	0.040853871
19227	OR4A5	-0.065113655	0.132540923
19228	OR2AP1	0.058735163	0.174926785
19229	OR13C4	-0.099177415	0.021774789
19230	AC064829.1	0.090769515	0.035823183
19231	CMC4	-0.014709569	0.734266316
19232	OR5K3	-0.06879427	0.111975153
19233	GOLGA6L6	0.091200654	0.034949767
19234	OR10AG1	-0.032449652	0.453854583
19235	KRTAP10-4	0.074646752	0.084535913
19236	OR2M3	0.074491366	0.085186888
19237	OR4C46	0.022724629	0.59995828
19238	KRTAP4-12	0.144808922	0.000781321
19239	KRTAP4-8	0.094995883	0.028013843
19240	DEFB107B	0.092088785	0.033207583
19241	SERINC4	-0.010078581	0.816088414
19242	LCE2B	0.069435673	0.108662938
19243	DUX4	0.119474566	0.005659074
19244	KRTAP19-2	-0.04432443	0.306150868
19245	OR4C15	0.046487898	0.283121493
19246	OR52A5	0.073135186	0.091041586
19247	OR10G6	-0.051286353	0.236309516
19248	VN1R4	0.02253464	0.60301155
19249	OR6K2	0.081078587	0.060923112
19250	IFNA10	0.069846791	0.106580896
19251	SPANXN5	0.058754884	0.174781769
19252	OR10H5	0.105705675	0.014440408
19253	AL135842.1	0.036541373	0.398943703
19254	STATH	0.06347471	0.142588598

19255	OR9G4	0.026715258	0.537504892
19256	HSFY2	0.016158969	0.709216784
19257	CT45A3	0.095422982	0.027312958
19258	KRTAP6-1	0.13184796	0.002244086
19259	FAM47A	0.06131829	0.156684235
19260	DAZ2	-0.013580771	0.753973844
19261	TAS2R16	0.023543128	0.586884891
19262	KRTAP19-4	0.053207843	0.219186974
19263	POU4F2	0.001463267	0.973063495
19264	AC009133.22	0.011235203	0.795425886
19265	KRTAP10-9	0.089536109	0.038425018
19266	OR1D5	0.058501573	0.176651318
19267	KRTAP19-1	0.061589214	0.154857538
19268	OR8K5	0.094874829	0.028215286
19269	DEFB113	0.006846434	0.874464735
19270	FO082796.1	0.089865023	0.037715987
19271	KRTAP10-5	0.070594492	0.102875221
19272	AC025263.3	-0.012996879	0.764231914
19273	GNAT2	-0.062925311	0.146084122
19274	IFNA2	0.045820037	0.290102256
19275	SLC17A6	0.085556627	0.047936629
19276	XAGE1B	0.164910582	0.000127242
19277	OR10H4	0.034181871	0.43010599
19278	MOS	-0.003179201	0.941517007
19279	DEFB130	0.004655383	0.914449414
19280	OR4C13	0.06657686	0.124040741
19281	AC092299.1	0.027552189	0.524832397
19282	CETN1	-0.094414504	0.028992714
19283	KRTAP19-3	-0.040552999	0.349178054
19284	OR2W1	0.00417244	0.923295419
19285	GK2	0.110262753	0.010704325
19286	PRAMEF1	0.085696984	0.047570354
19287	OR4N5	0.089995906	0.037436949
19288	FAM71E2	0.133883917	0.001912567
19289	KRTAP13-3	0.022754331	0.599481571
19290	BOD1L2	0.038184916	0.378060593
19291	OR13H1	0.094158709	0.029432611
19292	OR4K15	0.045742157	0.290923749
19293	MAGEA9	0.093905586	0.029873537
19294	OR6C3	0.104506776	0.015596652
19295	OR4C12	0.073409275	0.089833016
19296	C3orf56	0.060104778	0.165066674
19297	KRTAP20-3	0.037078938	0.392038324
19298	SSX4	0.047710461	0.270638916
19299	OR5T2	0.000893358	0.9835527
19300	OR51S1	-0.036924221	0.394018323
19301	KRTAP25-1	-0.043950304	0.310255478
19302	OR8H1	0.083506785	0.053560701
19303	AMY1A	0.06746991	0.119065055
19304	OR10H3	0.126753366	0.003316071
19305	RP11-512M8.5	-0.014964435	0.729840181
19306	OR8G1	0.000647771	0.988073711
19307	OR6C4	0.038340693	0.376116714
19308	NKX1-1	0.103869215	0.016244009
19309	KRTAP20-1	0.012725174	0.769019566
19310	GAGE12J	0.058509292	0.176594128
19311	OR51H1	-0.071901838	0.096642121
19312	CTD-2278I10.6	0.113959366	0.008331734

19313	OR4P4	0.024520914	0.571442118
19314	RP11-49K24.9	-0.168485631	9.00E-05
19315	OR6C6	0.048043091	0.267308811
19316	OR2T27	0.066764287	0.122983283
19317	TEX28	0.121440949	0.004911455
19318	KRTAP4-2	0.100434323	0.020152488
19319	LCE4A	-0.006618523	0.878610815
19320	VCY	0.052648408	0.224077812
19321	TGIF2LY	0.116048607	0.007209313
19322	OR10Z1	0.10106313	0.019381103
19323	NXF2B	-0.01751918	0.685989895
19324	KRTAP10-7	0.108984253	0.011654569
19325	RP4-533D7.6	-0.027648018	0.523391184
19326	OR7A10	0.080735246	0.062028634
19327	GOLGA6L22	0.041810782	0.334421441
19328	AC013269.5	0.049524744	0.2528173
19329	RP11-58C22.1	-0.064770545	0.134597806
19330	OR2M5	0.064567546	0.135826288
19331	FSCB	0.054654905	0.206891835
19332	OR5T1	0.077939048	0.071661974
19333	PRAMEF11	0.07237553	0.094459463
19334	OR5I1	-0.053681462	0.215106513
19335	MAGEB4	0.043493393	0.315317261
19336	OR4F4	-0.030328964	0.483907817
19337	AQP12B	0.050765354	0.241110534
19338	OR8J3	0.067188786	0.120614241
19339	AP000322.53	-0.147260826	0.000633545
19340	TCEB3CL2	-0.090260978	0.03687719
19341	MAGED4	0.043929511	0.310484664
19342	OR6C1	-0.012993372	0.764293646
19343	NEUROD6	-0.019362213	0.654987934
19344	CGB1	0.043652342	0.313550276
19345	KRTAP10-8	-0.027960237	0.518709681
19346	OR4K17	-0.019693706	0.649472316
19347	OR4L1	0.067818515	0.11716565
19348	OR51L1	-0.01203277	0.781259661
19349	OR6K6	0.086836171	0.044683458
19350	CDRT15L2	0.089486919	0.03853202
19351	OR5M9	0.038883085	0.369396581
19352	RP11-603J24.9	-0.077566729	0.073032298
19353	TBC1D3I	0.109765248	0.011065609
19354	DEFB112	-0.020458368	0.636822563
19355	FERD3L	0.081215502	0.060486825
19356	OR4A16	0.118501824	0.006065497
19357	KRTAP4-6	0.132200649	0.002183138
19358	DEFB107A	0.066497673	0.124489631
19359	RP11-318A15.7	-0.002329392	0.957131926
19360	OR4A15	0.012132863	0.779486834
19361	OR2M2	0.086699959	0.045020693
19362	CTC-490E21.12	-0.023274153	0.591166539
19363	KRTAP22-1	0.066500787	0.124471957
19364	RBMY1E	0.020775652	0.631604309
19365	KRTAP4-16P	0.119428368	0.005677808
19366	KRTAP20-2	0.007532032	0.86201369
19367	OR4S2	-0.033829631	0.434876033
19368	XAGE1A	0.135085028	0.001738667
19369	SPANXA2	0.076224688	0.078150127
19370	DEFB104A	0.024486346	0.57198478

19371	OR5M1	0.127922607	0.003035455
19372	OR5T3	0.047333038	0.274451663
19373	OR5H1	0.012503592	0.772930551
19374	KRTAP4-7	0.041311963	0.340225058
19375	OR10G8	0.010290386	0.812294597
19376	SPANXN2	0.046157387	0.286561819
19377	KRTAP22-2	0.074743906	0.08413094
19378	CSHL1	0.003592372	0.933932866
19379	OR4C11	0.046400993	0.284023385
19380	CTC-512J12.6	-0.084763421	0.05005127
19381	TBC1D3C	0.123351767	0.00427153
19382	C21orf140	0.049330403	0.254686346
19383	OR14C36	0.056307394	0.193470974
19384	MBD3L2	0.096921351	0.024972104
19385	KRTAP9-8	0.095958867	0.026454937
19386	PRAMEF5	0.082357477	0.05694752
19387	OR5D14	-0.018978897	0.661389293
19388	OR5AS1	0.10117719	0.019243972
19389	AC063956.1	-0.000757291	0.986057495
19390	KRTAP19-5	0.021592157	0.618259926
19391	RP1-317E23.6	-0.045846283	0.289825757
19392	C10orf32-ASMT	0.03167114	0.464763536
19393	OR5L2	0.026970853	0.533618563
19394	SPATA31A6	0.091430158	0.034492271
19395	PRY2	0.025899155	0.55000788
19396	KRTAP9-1	0.074654773	0.084502418
19397	SPATA31A1	0.145873543	0.000713619
19398	AC006538.4	0.021727044	0.61606731
19399	RP11-125O5.2	0.137223537	0.001464493
19400	PRAMEF6	0.114477177	0.008039898
19401	LIMS3L	-0.101830638	0.018474535
19402	HNRNPCL4	0.058519522	0.17651836
19403	KRTAP9-7	0.10419561	0.015909715
19404	OR5H14	0.04090081	0.345056796
19405	RP11-599B13.6	-0.015997449	0.71199339
19406	OR10G9	0.049344336	0.254552036
19407	CTB-60B18.6	0.131174519	0.002364801
19408	OR5M3	0.01607572	0.710647408
19409	XXbac-BPG32J3.20	0.027512514	0.525429682
19410	IFNA4	-0.008279474	0.848478476
19411	AC104389.2	0.079106301	0.067501484
19412	H2AFB3	-0.002197627	0.959554676
19413	GAGE2E	0.10801646	0.012422757
19414	KRTAP9-9	0.086318371	0.04597685
19415	AC003002.4	-0.04851033	0.262678707
19416	CXorf49B	0.067595719	0.118376818
19417	PRAMEF7	-0.05160165	0.233436995
19418	OR2T12	0.01510523	0.727398877
19419	KRTAP9-3	0.041110425	0.34258804
19420	RP11-240B13.2	0.070463882	0.103515068
19421	NXF2	-0.008924804	0.836827949
19422	DCANP1	0.035882554	0.407505504
19423	OR4F17	0.019659159	0.650046247
19424	RP11-216L13.16	-0.065146103	0.132347671
19425	CTAG1A	-0.002658076	0.951090202
19426	KRTAP4-3	0.090671969	0.036023349
19427	AC010646.3	0.072037788	0.096011619
19428	ATXN3L	0.06897654	0.111025934

19429	POM121L12	-0.021610392	0.61796331
19430	TBC1D3H	-0.007532214	0.862010396
19431	DEFB106B	-0.048141869	0.266325335
19432	DMRTC1	0.092831551	0.031807987
19433	DEFB114	0.02341889	0.588860777
19434	CTD-2545G14.7	-0.017415227	0.687754909
19435	MBD3L3	0.033842363	0.434703089
19436	OR6P1	0.051813592	0.231520033
19437	RP4-539M6.19	0.029781422	0.49183852
19438	OR8H3	0.081616815	0.059222867
19439	OR5H15	-0.011883353	0.78390822
19440	ACTRT2	0.010677101	0.805379224
19441	ANKRD20A3	-0.09232759	0.032751977
19442	CDY2A	0.035689079	0.410040448
19443	SPATA31A3	0.087005977	0.044266027
19444	KRTAP4-5	0.217564325	3.74E-07
19445	SPATA31A7	0.065983795	0.127433442
19446	LCE3B	0.01986768	0.646585238
19447	DEFB103A	0.076065231	0.078777103
19448	TCEB3C	-0.083124545	0.054667984
19449	RNASE12	-0.003676953	0.932381013
19450	OR5D16	0.040664712	0.347850967
19451	OR51A4	0.024388167	0.573527324
19452	KRTAP4-9	0.003819114	0.929773299
19453	POTEB3	-0.003621374	0.93340073
19454	MAGEA2	0.090920358	0.035515513
19455	RBMY1A1	0.034789425	0.421950045
19456	RP11-463C8.4	-0.001660714	0.969430475
19457	OR5D18	0.000983534	0.981892761
19458	PRY	0.015351209	0.723140397
19459	MBD3L5	0.05488068	0.205019441
19460	CSH1	0.076183828	0.078310397
19461	OR4F5	0.075200919	0.082246901
19462	TSPY1	0.089538396	0.03842005
19463	RP11-545J16.1	0.196460252	4.69E-06
19464	USP17L4	-0.014928206	0.730468815
19465	CTC-273B12.7	0.095459073	0.027254428
19466	RP11-201K10.3	0.015216558	0.725470487
19467	CTD-2561J22.3	-0.027050158	0.532415625
19468	PRR20E	0.033594842	0.438072409
19469	OR11H12	-0.005402395	0.900787658
19470	IQCF5	0.09485108	0.028254954
19471	CTAG1B	-0.039734119	0.35900355
19472	KRTAP2-4	-0.032130683	0.458306658
19473	HAPLN4	0.081035136	0.061062115
19474	RP11-1021N1.1	-0.062823492	0.146739064
19475	RP11-108O10.8	-0.013712726	0.751661518
19476	RP11-724O16.1	-0.003163414	0.941806895
19477	CCL27	0.006802767	0.875258846
19478	OR51A2	-0.004094261	0.924728327
19479	SPANXA1	0.105276975	0.014844894
19480	RP13-512J5.1	-0.035243184	0.415918101
19481	CXorf49	0.025366115	0.558250668
19482	RP5-972B16.2	0.038942235	0.368668245
19483	HSFY1	-0.033620383	0.437724052
19484	POTED	-0.081379085	0.059968943
19485	GOLGA6L1	0.10343495	0.016698317
19486	OPN1MW	0.022450925	0.604359128

19487	RP11-51L5.7	-0.037290513	0.389340459
19488	AC073610.5	0.050813732	0.240661863
19489	TCEB3CL	-0.05591121	0.196628994
19490	OR4F29	0.105229727	0.014890077
19491	RBMY1F	-0.07240264	0.094335747
19492	KRTAP2-1	0.012117764	0.779754181
19493	PRAMEF26	0.117744835	0.006399715
19494	RP11-145E5.5	-0.003858417	0.929052474
19495	OR4M2	-0.041909628	0.333278996
19496	MAGEA2B	0.167745001	9.68E-05
19497	NT5C1B-RDH14	-0.01623486	0.707913508
19498	OR4F3	-0.001288574	0.976278312
19499	GAGE12H	0.048720472	0.260614407
19500	CFC1B	-0.014096298	0.744952625
19501	USP17L10	-0.057814821	0.181794559
19502	TRIM49D1	0.005419258	0.900479594
19503	CTD-3148I10.9	0.001537959	0.971689097
19504	AMY1C	0.112654329	0.009109586
19505	KRTAP9-2	0.008850934	0.838159821
19506	DEFB106A	-0.074906952	0.083454823
19507	MAGEA9B	-0.01852731	0.668962538
19508	NPIPA2	-0.050231968	0.246096225
19509	TP53TG3	0.040970981	0.34422909
19510	RP11-156P1.2	0.013924887	0.747948345
19511	RP11-438J1.1	0.052774006	0.222973059
19512	OR11H1	0.018934832	0.66212678
19513	WI2-3308P17.2	0.023128029	0.593498577
19514	CTD-2140B24.4	-0.002489206	0.95419397
19515	DEFA1	-0.087872991	0.042185486
19516	PRR23D1	0.007411554	0.864199288
19517	UGT2A2	-0.041317594	0.34015918
19518	DAZ3	0.035089296	0.417958071
19519	OR10R2	0.013334039	0.758303373
19520	CTD-2587H24.4	0.072204222	0.095244214
19521	AC006328.4	0.002191415	0.959668911
19522	TRIM64	0.047761991	0.270121178
19523	CTB-96E2.2	-0.02185419	0.614003645
19524	RBMY1J	0.104565056	0.015538621
19525	TSPY6P	-0.032358132	0.455129495
19526	USP17L3	0.034516493	0.425602705
19527	TSPY10	-0.050734482	0.241397157
19528	VCY1B	0.013403104	0.757090687
19529	CT45A8	0.050320791	0.245261007
19530	BPY2	-0.144688499	0.000789343
19531	GAGE13	0.014277335	0.741792834
19532	TSPY8	0.067003101	0.121646093
19533	RP11-51F16.8	0.008459778	0.845219911
19534	USP17L8	-0.05255039	0.224942677
19535	PRR23D2	0.01447726	0.738308413
19536	DEFB104B	0.002485419	0.954263582
19537	AC002310.13	-0.024489601	0.571933664
19538	CT45A9	0.006955153	0.87248815
19539	AC009491.1	-0.023384547	0.58940751
19540	TSPY3	-0.045693453	0.291438277
19541	CT45A7	-0.053882693	0.213389429
19542	CTC-326K19.6	0.001796509	0.966932193
19543	RP11-294C11.3	0.056572292	0.191380266
19544	USP17L23	0.037067894	0.39217947

19545	CPLX3	0.018930518	0.662198998
19546	LLfos-48D6.2	-0.080781783	0.061877827
19547	POTEB	0.029240557	0.499740293
19548	BPY2C	-0.023737175	0.583804888
19549	CTD-2545M3.6	0.074675498	0.084415924
19550	CT45A6	-0.037406647	0.38786439
19551	TRIM49D2	0.029424462	0.497046012
19552	RP11-293I14.2	-0.045479087	0.293710175
19553	USP17L11	0.117617734	0.006457423
19554	OR4F16	-0.038326124	0.376298259
19555	CTC-432M15.3	-0.022805241	0.598664901
19556	USP17L18	0.042384089	0.327830273
19557	TSPY4	0.044383877	0.305501989
19558	DAZ4	0.148368161	0.0005757
19559	POTEB2	-0.010377726	0.810731466
19560	TMOD4	0.007076757	0.870278255
19561	CDY1B	0.014359886	0.740353447
19562	CDY2B	-0.003618456	0.93345426
19563	USP17L15	-0.010425543	0.80987599
19564	CDY1	-0.010308204	0.811975645
19565	RSC1A1	NA	NA
19566	AL109927.1	NA	NA
19567	AL513523.2	NA	NA
19568	OR10X1	NA	NA
19569	CFAP126	NA	NA
19570	SPHAR	NA	NA
19571	OR2L8	NA	NA
19572	OR2T11	NA	NA
19573	SFT2D3	NA	NA
19574	OR5H6	NA	NA
19575	RP11-484M3.5	NA	NA
19576	TXNRD3NB	NA	NA
19577	AC074033.1	NA	NA
19578	USP17L12	NA	NA
19579	USP17L13	NA	NA
19580	USP17L17	NA	NA
19581	USP17L19	NA	NA
19582	USP17L20	NA	NA
19583	USP17L21	NA	NA
19584	USP17L22	NA	NA
19585	USP17L24	NA	NA
19586	USP17L25	NA	NA
19587	USP17L26	NA	NA
19588	USP17L5	NA	NA
19589	USP17L27	NA	NA
19590	USP17L28	NA	NA
19591	USP17L29	NA	NA
19592	USP17L30	NA	NA
19593	AC006445.1	NA	NA
19594	PIGY	NA	NA
19595	CLDN22	NA	NA
19596	CTD-2287O16.3	NA	NA
19597	DND1	NA	NA
19598	HIST1H2AK	NA	NA
19599	OR12D2	NA	NA
19600	OR10C1	NA	NA
19601	TOMM6	NA	NA
19602	RP1-139D8.6	NA	NA

19603	PGAM2	NA	NA
19604	AC079355.1	NA	NA
19605	LUZP6	NA	NA
19606	OR2F1	NA	NA
19607	DEFA1B	NA	NA
19608	AL158147.2	NA	NA
19609	MSMP	NA	NA
19610	OR2S2	NA	NA
19611	SPATA31A5	NA	NA
19612	AL161784.1	NA	NA
19613	OR1B1	NA	NA
19614	CR392000.1	NA	NA
19615	RP11-350O14.18	NA	NA
19616	AC069547.1	NA	NA
19617	OR52B4	NA	NA
19618	OR51F1	NA	NA
19619	OR52R1	NA	NA
19620	OR51G1	NA	NA
19621	OR51B2	NA	NA
19622	OR2AG1	NA	NA
19623	OR10A6	NA	NA
19624	OR4X2	NA	NA
19625	OR4X1	NA	NA
19626	OR4C16	NA	NA
19627	OR5D13	NA	NA
19628	OR5L1	NA	NA
19629	OR8K3	NA	NA
19630	OR5R1	NA	NA
19631	OR5AR1	NA	NA
19632	OR6Q1	NA	NA
19633	OR1S1	NA	NA
19634	MALAT1	NA	NA
19635	PTPRCAP	NA	NA
19636	AP001888.1	NA	NA
19637	NDUFC2-KCTD14	NA	NA
19638	FXYD6-FXYD2	NA	NA
19639	C1QTNF5	NA	NA
19640	OR8D2	NA	NA
19641	OR8B4	NA	NA
19642	HEPN1	NA	NA
19643	KCNA6	NA	NA
19644	NPFF	NA	NA
19645	C1QTNF9B-AS1	NA	NA
19646	PRR20C	NA	NA
19647	PRR20A	NA	NA
19648	PRR20B	NA	NA
19649	PRR20D	NA	NA
19650	RP11-298I3.5	NA	NA
19651	OR4N4	NA	NA
19652	AC126407.1	NA	NA
19653	RP11-296A16.1	NA	NA
19654	AC009065.4	NA	NA
19655	CEMP1	NA	NA
19656	AC009121.2	NA	NA
19657	BOLA2	NA	NA
19658	TP53TG3C	NA	NA
19659	TP53TG3B	NA	NA
19660	RP11-343C2.12	NA	NA

19661	RP11-343C2.9	NA	NA
19662	PKD1L2	NA	NA
19663	AC138028.1	NA	NA
19664	OVCA2	NA	NA
19665	C17orf100	NA	NA
19666	AC129492.1	NA	NA
19667	AC006449.2	NA	NA
19668	FAM187A	NA	NA
19669	CTD-2510F5.6	NA	NA
19670	MBD3L4	NA	NA
19671	ICAM4	NA	NA
19672	CTD-2521M24.10	NA	NA
19673	GDF1	NA	NA
19674	AC011513.3	NA	NA
19675	TNFRSF6B	NA	NA
19676	ABHD16B	NA	NA
19677	GAB4	NA	NA
19678	TSSK2	NA	NA
19679	GP1BB	NA	NA
19680	AP000349.2	NA	NA
19681	XBP1	NA	NA
19682	C22orf31	NA	NA
19683	KREMEN1	NA	NA
19684	SOX10	NA	NA
19685	AL022328.1	NA	NA
19686	GAGE12B	NA	NA
19687	GAGE12C	NA	NA
19688	GAGE12D	NA	NA
19689	GAGE12E	NA	NA
19690	GAGE12F	NA	NA
19691	GAGE12G	NA	NA
19692	AL589842.1	NA	NA
19693	CT47A8	NA	NA
19694	RP1-321E8.5	NA	NA
19695	CT47A12	NA	NA
19696	CT47A11	NA	NA
19697	CT47A10	NA	NA
19698	CT47A9	NA	NA
19699	CT47A6	NA	NA
19700	CT47A7	NA	NA
19701	CT47A5	NA	NA
19702	CT47A4	NA	NA
19703	CT47A3	NA	NA
19704	CT47A2	NA	NA
19705	CT47A1	NA	NA
19706	CT45A2	NA	NA
19707	OPN1MW2	NA	NA
19708	H2AFB2	NA	NA
19709	RBMY1B	NA	NA
19710	RBMY1D	NA	NA
19711	AC012005.4	NA	NA
19712	BPY2B	NA	NA

Table S8. 83 Genes Highly Correlated with DDX39 in ccRCC

	symbol	correlation	*p*-value
ADRM1	ADRM1	0.723464429	8.06E-88
PFDN2	PFDN2	0.701432412	2.01E-80
NFKB2	NFKB2	0.661560028	1.23E-68
TCIRG1	TCIRG1	0.660760339	2.04E-68
LAMTOR2	LAMTOR2	0.656791275	2.40E-67
RFXANK	RFXANK	0.656042361	3.81E-67
FLT3LG	FLT3LG	0.634180619	1.55E-61
C19orf66	C19orf66	0.629671102	1.96E-60
MRPL55	MRPL55	0.626602441	1.07E-59
P2RY11	P2RY11	0.626234894	1.31E-59
IRF7	IRF7	0.623409052	6.18E-59
IFFO1	IFFO1	0.620339907	3.26E-58
LIMD2	LIMD2	0.618425386	9.12E-58
SECISBP2L	SECISBP2L	-0.616038508	3.25E-57
LIME1	LIME1	0.606471257	4.79E-55
PSMC4	PSMC4	0.604821282	1.11E-54
PSMD13	PSMD13	0.602521157	3.58E-54
ENPP4	ENPP4	-0.599799836	1.41E-53
CREBL2	CREBL2	-0.598789325	2.34E-53
CLTB	CLTB	0.598316558	2.96E-53
UBXN11	UBXN11	0.595315088	1.31E-52
PSMC5	PSMC5	0.593893915	2.64E-52
PSME2	PSME2	0.593853365	2.69E-52
SNRPF	SNRPF	0.593734633	2.85E-52
TYMP	TYMP	0.582982043	5.04E-50
DENND6B	DENND6B	0.581203608	1.17E-49
PAM16	PAM16	0.580482747	1.63E-49
ACAP1	ACAP1	0.578411389	4.30E-49
RNF166	RNF166	0.573081487	5.01E-48
ISG20	ISG20	0.569191766	2.92E-47
UNC13D	UNC13D	0.563698404	3.39E-46
CYBA	CYBA	0.563623171	3.51E-46
MAN1A2	MAN1A2	-0.557491215	5.13E-45
ARHGAP4	ARHGAP4	0.555241621	1.35E-44
MFAP3	MFAP3	-0.554943302	1.54E-44
TIMM50	TIMM50	0.553848986	2.46E-44
PRKAR2A	PRKAR2A	-0.552077116	5.23E-44
ISG15	ISG15	0.551268465	7.37E-44
HIPK3	HIPK3	-0.549026179	1.90E-43
AP3B1	AP3B1	-0.543617989	1.81E-42
UQCC2	UQCC2	0.539568663	9.54E-42
IGFLR1	IGFLR1	0.538688362	1.37E-41
ZAP70	ZAP70	0.534191443	8.37E-41
MITF	MITF	-0.53291931	1.39E-40
PSMD4	PSMD4	0.531735596	2.23E-40
HCST	HCST	0.53047582	3.67E-40
PBX4	PBX4	0.530387147	3.80E-40
RELB	RELB	0.529692018	5.00E-40
KCNN4	KCNN4	0.528926123	6.76E-40
TBC1D10C	TBC1D10C	0.528213062	8.95E-40
IFI35	IFI35	0.528154482	9.16E-40
REST	REST	-0.527119572	1.37E-39
BMPR2	BMPR2	-0.526481494	1.76E-39
TMEM79	TMEM79	0.526282735	1.90E-39
TNFRSF18	TNFRSF18	0.525498599	2.58E-39
PLEKHO1	PLEKHO1	0.524710111	3.51E-39

UHMK1	UHMK1	-0.523788387	5.01E-39
GNS	GNS	-0.522561686	8.05E-39
IFI30	IFI30	0.52209826	9.62E-39
C1orf228	C1orf228	0.521360864	1.28E-38
CLOCK	CLOCK	-0.521256523	1.33E-38
ACSS3	ACSS3	-0.520358546	1.87E-38
ARID5A	ARID5A	0.51914447	2.98E-38
RPS6KA4	RPS6KA4	0.519016373	3.13E-38
STRN	STRN	-0.518010164	4.58E-38
UBR1	UBR1	-0.514698201	1.60E-37
SH2D2A	SH2D2A	0.514242843	1.90E-37
CD7	CD7	0.514055264	2.03E-37
MAP7D1	MAP7D1	0.513448525	2.55E-37
TMEM170I	TMEM170B	-0.512982848	3.04E-37
GTF2A1	GTF2A1	-0.511942471	4.47E-37
CANX	CANX	-0.511700137	4.89E-37
AMH	AMH	0.509258521	1.21E-36
PSMC3	PSMC3	0.508954123	1.35E-36
UBXN1	UBXN1	0.50833037	1.70E-36
TRAF1	TRAF1	0.507325439	2.46E-36
MEF2B	MEF2B	0.506468085	3.36E-36
LAT	LAT	0.505646859	4.53E-36
BATF	BATF	0.505167544	5.40E-36
USP12	USP12	-0.50408677	7.99E-36
MRPS21	MRPS21	0.501544749	2.00E-35
CREBRF	CREBRF	-0.50068595	2.72E-35
ARHGAP18	ARHGAP18	-0.500631447	2.77E-35

Table S9. Functions of 20 immune genes most related to DDX39 in ccRCC.

ID	Alisases	Gene Function
ADRM1	Adhesion Regulating Molecule 1	Component of the 26S proteasome, a multiprotein complex involved in the ATP-dependent degradation of ubiquitinated proteins.
PFDN2	Prefoldin Subunit 2	Among its related pathways are Metabolism of proteins and Chaperonin-mediated protein folding. Gene Ontology (GO) annotations related to this gene include unfolded protein binding and protein binding involved in protein folding.
NFKB2	Nuclear Factor Kappa B Subunit 2	This gene encodes a subunit of the transcription factor complex nuclear factor-kappa-B (NFkB). The NFkB complex is expressed in numerous cell types and functions as a central activator of genes involved in inflammation and immune function.
TCIRG1	T Cell Immune Regulator 1, ATPase H+ Transporting V0 Subunit A3	Part of the proton channel of V-ATPases (By similarity). Seems to be directly involved in T-cell activation.
LAMTOR2	Late Endosomal/Lysosomal Adaptor, MAPK And MTOR Activator 2	As part of the Ragulator complex it is involved in amino acid sensing and activation of mTORC1, a signaling complex promoting cell growth in response to growth factors, energy levels, and amino acids.
RFXANK	Regulatory Factor X Associated Ankyrin Containing Protein	Activates transcription from class II MHC promoters. Activation requires the activity of the MHC class II transactivator/CIITA.
FLT3LG	Fms Related Receptor Tyrosine Kinase 3 Ligand	FLT3LG controls the development of DCs(dendritic cells) and is particularly important for plasmacytoid DCs and CD8-positive classical DCs and their CD103-positive tissue counterparts.
C19orf66	SHFL (Shiftless Antiviral Inhibitor Of Ribosomal Frameshifting)	Inhibits programmed -1 ribosomal frameshifting of a variety of mRNAs from viruses, such as HIV1, and cellular genes, such as PEG10.
MRPL55	Mitochondrial Ribosomal Protein L55	Among its related pathways are Mitochondrial translation and Organelle biogenesis and maintenance. Gene Ontology (GO) annotations related to this gene include structural constituent of ribosome.
P2RY11	Purinergic Receptor P2Y11	Receptor for ATP and ADP coupled to G-proteins that activate both phosphatidylinositol-calcium and adenylyl cyclase second messenger systems.
IRF7	Interferon Regulatory Factor 7	Can activate distinct gene expression programs in macrophages and regulate the anti-tumor properties of primary macrophages
IFFO1	Intermediate Filament Family Orphan 1	Nuclear matrix protein involved in the immobilization of broken DNA ends and the suppression of chromosome translocation during DNA double-strand breaks (DSBs).
LIMD2	LIM Domain Containing 2	Acts as an activator of the protein-kinase ILK, thereby regulating cell motility.
SECISBP2L	SECIS Binding Protein 2 Like	Binds SECIS (Sec insertion sequence) elements present on selenocysteine (Sec) protein mRNAs, but does not promote Sec incorporation into selenoproteins in vitro.
LIME1	Lck Interacting Transmembrane Adaptor 1	Involved in BCR-mediated signaling in B-cells and TCR (T-cell antigen receptor)-mediated T-cell signaling in T-cells.
PSMC4	Proteasome 26S Subunit, ATPase 4	The proteasome participates in numerous cellular processes, including cell cycle progression, apoptosis, or DNA damage repair.

PSMD13	Proteasome 26S Subunit, Non-ATPase 13	The proteasome participates in numerous cellular processes, including cell cycle progression, apoptosis, or DNA damage repair.
ENPP4	Ectonucleotide Pyrophosphatase/Phosphodiesterase 4	Among its related pathways are Innate Immune System and Metabolism. Gene Ontology (GO) annotations related to this gene include sulfuric ester hydrolase activity and bis(5'-adenosyl)-triphosphatase activity.
CREBL2	CAMP Responsive Element Binding Protein Like 2	Probable regulator of CREB1 transcriptional activity which is involved in adipose cells differentiation.
CLTB	Clathrin Light Chain B	Clathrin is the major protein of the polyhedral coat of coated pits and vesicles.

Table S10. 14 scores of DDX39 in TIDE analysis.

Patient	No benefits	Responder	TIDE	IFNG	MSI Expr Sig	Merck18	CD274	CD8
TCGA-BP-4770-01	FALSE	FALSE	3.28	-0.91	0.15	-0.3	-0.39	0.22
TCGA-BP-5177-01	FALSE	FALSE	2.98	-0.02	0.29	-0.04	-0.52	-0.2
TCGA-B0-5095-01	FALSE	FALSE	2.82	-0.37	0.14	-0.07	-0.73	0.36
TCGA-B0-5100-01	TRUE	FALSE	2.63	-1.71	0.17	-0.97	-0.9	-1.3
TCGA-AK-3431-01	FALSE	FALSE	2.52	-0.4	0.44	-0.3	-0.74	-0.4
TCGA-B0-4696-01	TRUE	FALSE	2.42	-2.36	0.19	-1.76	-1.18	-2
TCGA-B0-5080-01	FALSE	FALSE	2.33	-0.18	0.17	0.35	-0.53	1.09
TCGA-B0-5116-01	TRUE	FALSE	2.33	-1	0.45	-0.72	-0.81	-0.7
TCGA-BP-4771-01	FALSE	FALSE	2.24	1.74	0.23	1.78	0.07	3.33
TCGA-B0-4703-01	FALSE	FALSE	2.21	0.5	0.14	0.44	0.02	0.96
TCGA-B0-4688-01	TRUE	FALSE	2.21	-1.45	0.21	-0.77	1.96	-1.4
TCGA-BP-4769-01	TRUE	FALSE	2.18	-1.05	0.48	-1.03	-0.35	-1.8
TCGA-CJ-4907-01	FALSE	FALSE	2.15	-0.29	0.24	-0.29	-0.22	-0.8
TCGA-BP-5010-01	TRUE	FALSE	2.13	-1.03	0.12	-0.41	-0.8	0.05
TCGA-BP-4804-01	FALSE	FALSE	2.07	1.13	0.29	1.16	0.03	1.87
TCGA-CJ-6033-01	FALSE	FALSE	2.05	-0.29	0.37	-0.11	-0.56	-0.2
TCGA-BP-4173-01	FALSE	FALSE	2.03	2.91	0.41	2.2	0.86	3.36
TCGA-BP-4795-01	FALSE	FALSE	2.03	-0.92	0.21	-0.31	-0.44	-0.2
TCGA-B0-4837-01	FALSE	FALSE	2.01	-0.91	0.31	-0.57	-0.75	-0.8
TCGA-CJ-4871-01	FALSE	FALSE	1.99	0.25	0.6	-0.02	-0.19	-0.1
TCGA-BP-4789-01	FALSE	FALSE	1.99	-0.94	0.42	-0.68	-0.1	-0.8
TCGA-BP-4784-01	FALSE	FALSE	1.97	-0.9	0.44	-0.65	-0.72	-1.3
TCGA-B0-5081-01	FALSE	FALSE	1.96	1.2	0.26	1.15	0.22	1.56
TCGA-CJ-4905-01	FALSE	FALSE	1.92	-0.51	0.24	-0.17	0.02	-0.3
TCGA-BP-4337-01	TRUE	FALSE	1.91	-1.05	0.29	-0.58	0.12	-0.8
TCGA-BP-4762-01	FALSE	FALSE	1.88	-0.32	0.59	0.01	-0.28	0.39
TCGA-B0-5098-01	TRUE	FALSE	1.85	-1.9	0.22	-1.4	-1.04	-1.3
TCGA-BP-4345-01	FALSE	FALSE	1.85	0.1	0.1	0.37	0.76	-0.3
TCGA-BP-4177-01	TRUE	FALSE	1.83	-1.27	0.65	-0.95	-0.46	-1.3
TCGA-B0-4843-01	TRUE	FALSE	1.82	-1.18	0.11	-0.79	-0.9	-1.4
TCGA-B8-5550-01	FALSE	FALSE	1.79	0.11	0.41	0.19	0.19	0.06
TCGA-BP-4760-01	TRUE	FALSE	1.78	-1.42	0.55	-1.03	0.33	-1.6
TCGA-A3-3308-01	FALSE	FALSE	1.78	-0.21	0.19	-0.34	0.55	-0.8
TCGA-B4-5843-01	FALSE	FALSE	1.77	-0.81	0.43	-0.78	0.17	-1.2
TCGA-AK-3426-01	FALSE	FALSE	1.77	1.38	0.18	1.63	-0.02	3.07
TCGA-DV-5568-01	FALSE	FALSE	1.76	-0.19	0.36	0.28	0.45	1.4
TCGA-CJ-5679-01	TRUE	FALSE	1.74	-1.79	0.46	-0.93	-0.94	-0.8
TCGA-BP-4346-01	FALSE	FALSE	1.74	2.25	0.52	1.74	0.65	3.18
TCGA-CZ-5469-01	TRUE	FALSE	1.72	-1.32	0.27	-0.64	-0.86	-0.6
TCGA-B0-4819-01	FALSE	FALSE	1.72	0.96	0.2	1.34	0.73	2.54
TCGA-BP-5198-01	FALSE	FALSE	1.72	-0.4	0.28	0	-0.57	0.17
TCGA-CJ-4920-01	FALSE	FALSE	1.7	-0.39	0.29	-0.42	-0.37	-1
TCGA-BP-5170-01	FALSE	FALSE	1.7	-0.75	0.52	-0.22	-0.31	-0.5
TCGA-B4-5838-01	FALSE	FALSE	1.69	-0.43	0.74	-0.37	1.04	-0.1
TCGA-A3-3349-01	TRUE	FALSE	1.68	-1.38	0.23	-0.57	-0.66	-0.1
TCGA-B0-5120-01	FALSE	FALSE	1.68	-0.65	0.53	-0.35	-0.04	-1
TCGA-CJ-5671-01	FALSE	FALSE	1.62	0.31	0.13	0.54	-0.07	0.54
TCGA-BP-4167-01	FALSE	FALSE	1.62	0.92	0.35	1.29	-0.2	1.91
TCGA-BP-4758-01	FALSE	FALSE	1.61	0.71	0.36	1	-0.03	2.36
TCGA-CJ-5677-01	FALSE	FALSE	1.57	-0.39	0.29	-0.19	-0.65	-0.8
TCGA-B0-4697-01	FALSE	FALSE	1.57	1.57	0.24	1.56	1.03	2.69
TCGA-CJ-4869-01	FALSE	FALSE	1.55	1.07	0.48	1.55	0.19	3.27
TCGA-BP-5191-01	FALSE	FALSE	1.55	1.16	0.35	0.76	-0.19	0.37
TCGA-CJ-4916-01	FALSE	FALSE	1.55	1.58	0.51	1.57	0.4	2.93
TCGA-CJ-4882-01	FALSE	FALSE	1.52	0.05	0.08	0.33	-0.64	0.84
TCGA-BP-4166-01	FALSE	FALSE	1.52	0.5	0.53	0.08	-0.28	-0.1

TCGA-MM-A563-01	FALSE	FALSE	1.51	1.07	0.35	0.78	0.36	0.74
TCGA-EU-5905-01	FALSE	FALSE	1.5	-0.52	0.28	-0.23	-0.58	0.31
TCGA-BP-4159-01	FALSE	FALSE	1.48	-0.12	0.49	-0.31	-0.71	-0.9
TCGA-BP-5199-01	FALSE	FALSE	1.46	1.46	0.25	1.34	-0.28	2.82
TCGA-B0-5092-01	FALSE	FALSE	1.45	1.48	0.35	1.61	0.21	3.16
TCGA-BP-4330-01	FALSE	FALSE	1.44	1.42	0.5	1.24	0.4	2.13
TCGA-CJ-4890-01	FALSE	FALSE	1.43	1.34	0.23	1.39	0.45	2.44
TCGA-B0-4700-01	FALSE	FALSE	1.42	1.64	0.25	1.62	0.52	2.39
TCGA-BP-4352-01	TRUE	FALSE	1.41	-1.93	0.25	-1.34	0.41	-1.8
TCGA-CJ-4901-01	FALSE	FALSE	1.41	2.32	0.4	1.56	0.71	1.97
TCGA-CJ-4887-01	FALSE	FALSE	1.4	1.12	0.65	1.18	-0.09	2.19
TCGA-BP-4763-01	FALSE	FALSE	1.39	0.11	0.73	-0.22	-0.06	-0.5
TCGA-BP-5195-01	FALSE	FALSE	1.38	-0.17	0.35	-0.09	-0.42	-0.4
TCGA-DV-5576-01	TRUE	FALSE	1.38	-1.57	0.4	-1.27	-0.56	-1.9
TCGA-B0-4714-01	FALSE	FALSE	1.37	1.61	0.42	1.07	0.62	2.05
TCGA-B0-5709-01	FALSE	FALSE	1.35	1.25	0.46	1.21	0.42	1.72
TCGA-MM-A564-01	TRUE	FALSE	1.35	-1.09	0.35	-0.74	-0.71	-1.2
TCGA-B8-5545-01	FALSE	FALSE	1.33	0.13	0.33	0.24	0.1	-0.3
TCGA-B8-A54K-01	TRUE	FALSE	1.33	-1.6	0.55	-1.29	-0.38	-1.6
TCGA-CW-5588-01	FALSE	FALSE	1.32	-0.58	0.44	-0.34	-0.53	-0.3
TCGA-A3-A8OX-01	FALSE	FALSE	1.32	0.23	0.64	0.32	0.59	0.14
TCGA-B8-5162-01	FALSE	FALSE	1.32	1.54	0.45	1.62	0.77	2.28
TCGA-B0-5693-01	FALSE	FALSE	1.31	-0.77	0.59	-0.81	0.19	-1.3
TCGA-BP-5187-01	FALSE	FALSE	1.31	0.16	0.58	-0.13	-0.18	0.57
TCGA-CZ-5988-01	FALSE	FALSE	1.3	0.17	0.51	0.12	-0.06	0.27
TCGA-BP-5000-01	FALSE	FALSE	1.3	0.39	0.28	0.44	-0.6	0.9
TCGA-BP-4765-01	FALSE	FALSE	1.3	-0.94	0.57	-0.83	-0.29	-1.7
TCGA-BP-4989-01	FALSE	FALSE	1.29	0.91	0.3	1.2	0.41	2.87
TCGA-B0-5696-01	FALSE	FALSE	1.29	-0.06	0.8	-0.01	1.18	-0.1
TCGA-A3-A6NJ-01	FALSE	FALSE	1.26	1.06	0.7	1.13	1.06	2.19
TCGA-B8-A8YJ-01	FALSE	FALSE	1.25	-0.91	0.11	-0.04	-0.27	-0.5
TCGA-B0-5812-01	FALSE	FALSE	1.25	0.54	0.5	0.18	0.04	-0.1
TCGA-B0-4707-01	TRUE	FALSE	1.24	-1.37	0.22	-0.54	-1.07	0.8
TCGA-AK-3436-01	FALSE	FALSE	1.24	-0.82	0.69	-0.51	-0.71	0
TCGA-CJ-4902-01	FALSE	FALSE	1.22	0.21	0.16	0.28	-0.38	0.19
TCGA-BP-4803-01	FALSE	FALSE	1.21	-0.37	0.51	-0.37	-0.71	-0.4
TCGA-BP-5169-01	FALSE	FALSE	1.2	-0.62	0.33	0.05	-0.72	0.44
TCGA-BP-5201-01	FALSE	FALSE	1.2	0.97	0.52	0.85	-0.02	1.98
TCGA-CJ-6031-01	FALSE	FALSE	1.19	0.55	0.42	0.75	-0.35	1.42
TCGA-A3-3316-01	FALSE	FALSE	1.19	-0.67	0.26	-0.65	-0.71	-0.9
TCGA-B0-4847-01	FALSE	FALSE	1.19	1.08	0.43	0.93	0.24	1.97
TCGA-B0-5084-01	TRUE	FALSE	1.19	-1	0.13	-0.79	-0.88	-0.5
TCGA-CW-5591-01	TRUE	FALSE	1.19	-1.41	0.45	-1.14	-0.37	-1.3
TCGA-B0-5692-01	FALSE	FALSE	1.18	1.36	0.72	1.57	0.25	3.29
TCGA-B0-4712-01	FALSE	FALSE	1.18	0.53	0.39	0.39	-0.1	0.4
TCGA-AK-3453-01	FALSE	FALSE	1.17	1.02	0.35	0.87	0.23	1.99
TCGA-B0-5097-01	FALSE	FALSE	1.16	0.17	0.18	0.05	-0.03	-0.2
TCGA-BP-4986-01	FALSE	FALSE	1.15	-0.18	0.25	-0.26	-0.21	-0.8
TCGA-DV-5567-01	TRUE	FALSE	1.15	-1.34	0.46	-1.27	-0.55	-1.6
TCGA-BP-4342-01	FALSE	FALSE	1.14	-0.49	0.31	-0.38	-0.66	-0.9
TCGA-B8-4620-01	FALSE	FALSE	1.12	-0.1	0.45	-0.31	-0.24	-1.2
TCGA-B8-5551-01	FALSE	FALSE	1.11	2.46	0.34	1.99	0.91	1.97
TCGA-CJ-4888-01	FALSE	FALSE	1.1	1.92	0.46	1.7	1.75	2.52
TCGA-B0-4694-01	FALSE	FALSE	1.1	1.69	0.76	1.39	0.11	2.08
TCGA-BP-4355-01	TRUE	FALSE	1.1	-1.17	0.17	-0.82	-0.16	-1.4
TCGA-AS-3778-01	FALSE	FALSE	1.09	-0.24	0.54	-0.29	-0.04	-0.4
TCGA-CJ-4900-01	FALSE	FALSE	1.09	1.12	0.56	1.06	-0.23	2.08
TCGA-B0-4815-01	FALSE	FALSE	1.05	1.66	0.33	1.46	0.75	2.54

TCGA-BP-4759-01	FALSE	FALSE	1.04	-0.49	0.53	-0.41	-0.52	0.16
TCGA-BP-4962-01	FALSE	FALSE	1.04	0.68	0.84	0.85	-0.05	1.58
TCGA-BP-4332-01	FALSE	FALSE	1.04	-0.76	0.44	-0.48	-0.43	-0.5
TCGA-CJ-4889-01	FALSE	FALSE	1.03	0.3	0.41	0.63	0.21	0.96
TCGA-MM-A84U-01	FALSE	FALSE	1.03	-0.65	0.32	-0.2	-0.69	-0.6
TCGA-B8-4148-01	FALSE	FALSE	1.03	1.66	0.52	1.06	0.75	2.37
TCGA-B4-5834-01	FALSE	FALSE	1.03	0.43	0.45	0.03	0.12	-0.7
TCGA-CJ-4908-01	FALSE	FALSE	1.02	-0.25	0.2	-0.05	0.01	0.2
TCGA-B0-4810-01	FALSE	FALSE	1.02	0.98	0.41	0.93	0.25	1.52
TCGA-B2-5636-01	TRUE	FALSE	1.02	-1.33	0.24	-0.84	-0.46	-1.4
TCGA-B4-5378-01	TRUE	FALSE	1.01	-1.19	0.56	-0.76	-0.14	-0.9
TCGA-DV-A4VZ-01	TRUE	FALSE	1.01	-2.3	0.37	-1.57	0.46	-1.8
TCGA-CJ-5683-01	FALSE	FALSE	1	-0.61	0.61	-0.48	-0.56	-0.3
TCGA-B2-5641-01	FALSE	FALSE	0.99	0.94	0.62	1.15	0.04	2.22
TCGA-BP-4985-01	FALSE	FALSE	0.99	-0.38	0.15	-0.58	-0.39	-1.3
TCGA-CJ-4885-01	FALSE	FALSE	0.99	-0.46	0.73	-0.43	0.06	-0.2
TCGA-BP-4961-01	FALSE	FALSE	0.99	-1.41	0.32	-0.72	-0.21	-0.9
TCGA-CZ-5468-01	FALSE	FALSE	0.98	-0.96	0.32	-0.15	0.62	-0.2
TCGA-B8-A54F-01	FALSE	FALSE	0.97	-1.09	0.57	-0.63	0.14	-0.9
TCGA-BP-5186-01	FALSE	FALSE	0.97	-0.02	0.62	-0.05	0.23	0.18
TCGA-B0-4823-01	FALSE	FALSE	0.97	0.06	0.67	0.32	-0.09	1.45
TCGA-BP-4344-01	FALSE	FALSE	0.96	-0.44	0.37	-0.47	-0.13	-1.3
TCGA-DV-5566-01	FALSE	FALSE	0.96	-0.02	0.65	-0.28	-0.18	-0.6
TCGA-BP-4781-01	FALSE	FALSE	0.95	-0.31	0.47	-0.36	-0.31	-0.9
TCGA-B0-5691-01	FALSE	FALSE	0.95	0.26	0.49	-0.03	0.13	-0.2
TCGA-CZ-4854-01	FALSE	FALSE	0.95	-0.42	0.28	-0.31	-0.37	-0.3
TCGA-3Z-A93Z-01	FALSE	FALSE	0.94	0.12	0.61	0.07	0.4	-0.4
TCGA-B8-A54J-01	FALSE	FALSE	0.93	0.24	0.43	0.34	0.48	0.91
TCGA-A3-3365-01	FALSE	FALSE	0.92	-0.06	0.54	-0.14	-0.07	-0.3
TCGA-CJ-4637-01	FALSE	FALSE	0.92	2.55	0.55	2.1	0.96	3.3
TCGA-B0-4814-01	FALSE	FALSE	0.91	-0.35	0.66	-0.39	0.57	-0.6
TCGA-B8-A7U6-01	FALSE	FALSE	0.91	-0.45	0.55	-0.52	-0.42	-0.9
TCGA-DV-5565-01	FALSE	FALSE	0.9	-0.73	0.12	-0.38	-0.3	-0.6
TCGA-CJ-4634-01	FALSE	FALSE	0.9	-0.96	0.5	-0.77	-0.17	-0.8
TCGA-B0-5106-01	FALSE	FALSE	0.89	-0.07	0.23	0.12	-0.39	0.96
TCGA-BP-4326-01	FALSE	FALSE	0.89	-0.4	0.28	-0.68	-0.26	-1.2
TCGA-BP-4974-01	FALSE	FALSE	0.88	-0.95	0.14	-0.68	-0.33	-0.7
TCGA-B0-4813-01	FALSE	FALSE	0.88	-1.44	0.33	-1.02	-0.68	-1.4
TCGA-DV-5574-01	FALSE	FALSE	0.88	0.3	0.23	0.59	0.18	1.27
TCGA-CJ-4897-01	FALSE	FALSE	0.88	-0.03	0.67	-0.1	0.18	-0.1
TCGA-CZ-5467-01	FALSE	FALSE	0.86	0.23	0.58	-0.24	-0.3	-0.8
TCGA-BP-4999-01	FALSE	FALSE	0.85	-0.57	0.36	-0.65	0.17	-1
TCGA-B0-5707-01	FALSE	FALSE	0.85	-1.64	0.61	-0.89	-0.24	-1
TCGA-CJ-4868-01	FALSE	FALSE	0.84	0.48	0.36	0.8	0.28	1.38
TCGA-CZ-5464-01	FALSE	FALSE	0.84	1.18	0.42	0.88	-0.02	1.05
TCGA-B2-4101-01	FALSE	FALSE	0.84	0.72	0.4	0.57	0.47	0.64
TCGA-CJ-4892-01	FALSE	FALSE	0.84	0.31	0.67	0.71	-0.07	2.33
TCGA-BP-4807-01	FALSE	FALSE	0.84	-0.34	0.7	-0.4	0.07	-1.1
TCGA-CZ-5466-01	FALSE	FALSE	0.84	0.52	0.65	0.68	-0.06	1.12
TCGA-B0-5695-01	FALSE	FALSE	0.83	-0.52	0.65	-0.28	0.1	-0.2
TCGA-B0-5096-01	FALSE	FALSE	0.83	-0.19	0.64	-0.15	-0.71	-0.4
TCGA-A3-3387-01	FALSE	FALSE	0.83	-0.23	0.39	-0.11	-0.34	-0.7
TCGA-CZ-4857-01	FALSE	FALSE	0.81	-0.05	0.25	-0.17	-0.47	-0.3
TCGA-A3-3347-01	FALSE	FALSE	0.81	0.19	0.18	0.2	-0.04	0.17
TCGA-A3-3362-01	FALSE	FALSE	0.81	-0.38	0.61	-0.43	-0.1	-0.3
TCGA-CZ-5461-01	FALSE	FALSE	0.8	0.84	0.52	0.2	-0.09	-0.3
TCGA-BP-5181-01	FALSE	FALSE	0.8	0.23	0.61	-0.04	0.1	-0.3
TCGA-CW-5583-01	FALSE	FALSE	0.8	-0.55	0.58	-0.46	-0.09	-0.5

TCGA-B8-5163-01	FALSE	FALSE	0.79	1.49	0.23	1.27	0.53	1.43
TCGA-DV-A4W0-05	FALSE	FALSE	0.78	-1.27	0.61	-1.1	-0.05	-1.8
TCGA-B0-5710-01	FALSE	FALSE	0.78	0.64	0.27	0.17	0.2	-0.2
TCGA-G6-A8L7-01	FALSE	FALSE	0.77	-1.75	0.4	-0.76	-0.58	-0
TCGA-B0-4833-01	FALSE	FALSE	0.77	0.13	0.47	0.45	0.09	1.59
TCGA-B0-5697-01	FALSE	FALSE	0.77	0.58	0.53	0.83	0.1	1.81
TCGA-B0-4848-01	FALSE	FALSE	0.75	0.33	0.47	0.28	-0.34	0.87
TCGA-CJ-4881-01	FALSE	FALSE	0.75	-0.45	0.16	-0.16	-0.68	0.28
TCGA-DV-5573-01	FALSE	FALSE	0.74	0.8	0.66	1.26	0.16	3.19
TCGA-B0-5400-01	FALSE	FALSE	0.74	-0.66	0.67	0.24	0.38	0.46
TCGA-BP-4975-01	FALSE	FALSE	0.73	-0.53	0.67	-0.6	-0.26	-1.1
TCGA-BP-4787-01	FALSE	FALSE	0.73	-0.41	0.29	-0.06	-0.27	-0
TCGA-A3-A8CQ-01	FALSE	FALSE	0.73	-0.34	0.42	-0.48	-0.21	-1
TCGA-B4-5836-01	FALSE	FALSE	0.73	0.42	0.63	0.22	0.33	-0.1
TCGA-BP-4777-01	FALSE	FALSE	0.72	1.08	0.79	1.19	0.51	2.43
TCGA-BP-4351-01	FALSE	FALSE	0.72	-1	0.19	-0.75	-0.7	-0.7
TCGA-BP-5196-01	FALSE	FALSE	0.71	0.62	0.22	0.77	-0.34	1.85
TCGA-B0-5694-01	FALSE	FALSE	0.71	-0.43	0.52	-0.44	-0.8	-1.3
TCGA-BP-4165-01	FALSE	FALSE	0.7	0.14	0.45	0.29	-0.18	0.45
TCGA-A3-3325-01	FALSE	FALSE	0.7	-0.69	0.44	-0.43	-0.31	-1.1
TCGA-BP-4993-01	FALSE	FALSE	0.69	-0.13	0.32	0.27	-0.18	0.84
TCGA-CW-5585-01	FALSE	FALSE	0.69	-0.77	0.76	-0.67	-0.11	-1.5
TCGA-BP-4329-01	FALSE	FALSE	0.69	0.34	0.68	-0.1	-0.09	-0.5
TCGA-CJ-4918-01	FALSE	FALSE	0.68	-0.04	0.19	0.03	-0.43	0.36
TCGA-B4-5844-01	FALSE	FALSE	0.66	-0.33	0.43	-0.54	-0.2	-1.1
TCGA-B0-5099-01	FALSE	FALSE	0.66	-1.23	0.71	-1.06	-0.24	-1.5
TCGA-BP-5176-01	FALSE	FALSE	0.66	-0.36	0.59	-0.17	-0.24	-0.5
TCGA-CZ-4856-01	FALSE	FALSE	0.66	0.28	0.69	0.45	0.09	0.8
TCGA-BP-4343-01	FALSE	FALSE	0.66	1.32	0.61	0.65	0.3	0.55
TCGA-CJ-4870-01	FALSE	FALSE	0.65	-0.96	0.66	-0.64	0.31	-1.5
TCGA-BP-4782-01	FALSE	FALSE	0.65	0.67	0.52	0.56	-0.16	0.71
TCGA-CW-6090-01	FALSE	FALSE	0.65	0.09	0.49	0.31	0.18	0.67
TCGA-A3-A8OU-01	FALSE	FALSE	0.64	-0.4	0.53	-0.19	0.17	-0.4
TCGA-BP-4964-01	FALSE	FALSE	0.64	0.38	0.65	0.04	0.24	0.3
TCGA-BP-5001-01	FALSE	FALSE	0.64	-0.48	0.36	-0.15	-0.58	0.65
TCGA-CW-6087-01	FALSE	FALSE	0.64	3.48	0.5	2.76	1.71	3.56
TCGA-CZ-4860-01	FALSE	FALSE	0.63	-0.49	0.57	-0.19	-0.5	-0.7
TCGA-CZ-4862-01	FALSE	FALSE	0.63	2.31	0.58	1.57	1.18	2.41
TCGA-CJ-4895-01	FALSE	FALSE	0.62	0.22	0.54	0.21	-0.59	0.4
TCGA-BP-4967-01	FALSE	FALSE	0.62	-0.78	0.41	-0.56	-0.61	-0.6
TCGA-B0-5690-01	FALSE	FALSE	0.61	0.04	0.18	-0.15	-0.57	-0.9
TCGA-BP-5183-01	FALSE	FALSE	0.61	0.53	0.49	0.55	-0.27	1.24
TCGA-AK-3425-01	FALSE	FALSE	0.6	1.01	0.63	0.89	-0.26	1.77
TCGA-B0-5094-01	FALSE	FALSE	0.6	-0.97	0.48	-0.57	-0.69	-0.3
TCGA-BP-5004-01	FALSE	FALSE	0.6	-0.09	0.57	-0.23	-0.4	-0.1
TCGA-B0-4827-01	FALSE	FALSE	0.6	0.76	0.52	0.64	-0.24	0.99
TCGA-BP-4349-01	FALSE	FALSE	0.59	-0.34	0.3	-0.56	-0.4	-0.8
TCGA-BP-4968-01	FALSE	FALSE	0.59	0.77	0.61	0.71	0.31	1.62
TCGA-A3-A6NI-01	FALSE	FALSE	0.58	-0.73	0.62	-0.46	-0.02	-1.6
TCGA-CJ-5684-01	FALSE	FALSE	0.58	0.59	0.64	0.62	-0.18	1.25
TCGA-AK-3445-01	FALSE	FALSE	0.58	-0.06	0.33	0.13	-0.52	0.28
TCGA-CW-5587-01	FALSE	FALSE	0.58	1.72	0.67	1.39	0.63	2.27
TCGA-CJ-5675-01	FALSE	FALSE	0.57	1.62	0.72	1.67	0.54	3.47
TCGA-DV-5575-01	FALSE	FALSE	0.57	0.76	0.54	0.54	0.64	0.82
TCGA-BP-4970-01	FALSE	FALSE	0.56	0.99	0.31	0.79	1.06	1.04
TCGA-B0-4710-01	FALSE	FALSE	0.56	-0.52	0.47	-0.31	-0.31	0.14
TCGA-CZ-5453-01	FALSE	FALSE	0.56	-0.89	0.7	-0.89	0.32	-1.7
TCGA-CJ-5689-01	FALSE	FALSE	0.54	-0.34	0.38	0.3	0	0.7

TCGA-CJ-4899-01	FALSE	FALSE	0.54	-0.64	0.27	-0.27	0.29	-0.3
TCGA-B8-5165-01	FALSE	FALSE	0.53	-0.7	0.48	-0.38	0.2	-0.5
TCGA-B0-5706-01	FALSE	FALSE	0.53	2.02	0.62	1.42	0.82	2.45
TCGA-A3-3317-01	FALSE	FALSE	0.52	0.19	0.35	0.08	-0.01	0.3
TCGA-BP-5175-01	FALSE	FALSE	0.52	-1.19	0.15	-0.2	-0.3	-0.1
TCGA-B8-5164-01	FALSE	FALSE	0.52	2.09	0.7	1.87	1.3	3.25
TCGA-B0-4691-01	FALSE	FALSE	0.51	0.05	0.66	0.37	-0.48	1.39
TCGA-CJ-5676-01	FALSE	FALSE	0.51	-0.31	0.27	0.32	-0.54	0.73
TCGA-B0-4821-01	FALSE	FALSE	0.5	-1.04	0.18	-0.25	-0.65	0.24
TCGA-BP-4162-01	FALSE	FALSE	0.5	1.11	0.69	0.97	0.91	1.83
TCGA-BP-4158-01	FALSE	FALSE	0.49	-0.41	0.62	-0.46	-0.42	-0.6
TCGA-CW-5580-01	FALSE	FALSE	0.47	-0.36	0.54	-0.52	-0.07	-1.4
TCGA-CZ-5456-01	FALSE	FALSE	0.47	-0.67	0.47	-0.06	-0.44	0.13
TCGA-CW-5589-01	FALSE	FALSE	0.47	-0.34	0.52	-0.25	-0.12	-0.9
TCGA-AK-3454-01	FALSE	FALSE	0.46	-1.17	0.06	-0.38	-1.06	0.15
TCGA-BP-4761-01	FALSE	FALSE	0.46	-0.66	0.65	0.24	-0.15	0.86
TCGA-B0-4844-01	FALSE	FALSE	0.45	0.01	0.56	-0.14	-0.48	0.25
TCGA-B8-4143-01	FALSE	FALSE	0.44	1.58	0.38	1.2	0.21	2.25
TCGA-B8-A54D-01	FALSE	FALSE	0.44	1.1	0.65	1.03	0.03	2.54
TCGA-CW-5584-01	FALSE	FALSE	0.44	0.28	0.54	-0.3	-0.34	-0.8
TCGA-A3-A6NN-01	FALSE	FALSE	0.44	-0.02	0.46	0.05	-0.05	-0.3
TCGA-CJ-4872-01	FALSE	FALSE	0.44	0.7	0.21	0.42	-0.08	0.25
TCGA-BP-4169-01	FALSE	FALSE	0.43	0.68	0.33	0.26	0.32	0.27
TCGA-BP-4983-01	FALSE	FALSE	0.43	0.95	0.62	1.61	4.98	2.61
TCGA-B0-4845-01	FALSE	FALSE	0.43	-0.96	0.25	-0.95	-0.41	-1.7
TCGA-CJ-5680-01	FALSE	FALSE	0.42	-0.66	0.75	-0.64	-0.32	-1.2
TCGA-CW-6088-01	FALSE	FALSE	0.42	-0.63	0.42	-0.58	-0.03	-1.1
TCGA-B8-5553-01	FALSE	FALSE	0.42	1.16	0.57	0.71	0.1	1.18
TCGA-CJ-4886-01	FALSE	FALSE	0.42	0.78	0.52	0.68	0.23	0.88
TCGA-BP-5194-01	FALSE	FALSE	0.42	-0.17	0.57	-0.16	0.21	-0.3
TCGA-A3-3376-01	FALSE	FALSE	0.41	-1.1	0.13	-0.42	-0.33	-0.7
TCGA-BP-4960-01	FALSE	FALSE	0.41	-0.92	0.15	-0.4	-0.85	-0.1
TCGA-G6-A8L8-01	FALSE	FALSE	0.41	-0.85	0.47	-0.73	0.03	-1.6
TCGA-CJ-4912-01	FALSE	FALSE	0.4	-1.46	0.29	-0.82	-0.87	-0.8
TCGA-B0-5712-01	FALSE	FALSE	0.4	-0.51	0.89	-0.27	0.26	0.28
TCGA-B0-4824-01	FALSE	FALSE	0.39	1.15	0.71	0.98	0.63	1.92
TCGA-BP-5192-01	FALSE	FALSE	0.39	-0.88	0.73	-0.68	-0.09	-0.8
TCGA-CW-6097-01	FALSE	FALSE	0.39	0.94	0.24	0.94	0.26	1.22
TCGA-A3-3359-01	FALSE	FALSE	0.39	0.99	0.63	0.65	0.34	1.26
TCGA-B0-4822-01	FALSE	FALSE	0.38	-1.06	0.09	-0.18	-0.81	0.52
TCGA-EU-5907-01	FALSE	FALSE	0.37	-0.25	0.82	-0.41	0.88	-1.3
TCGA-B0-4706-01	FALSE	FALSE	0.37	-0.42	0.38	-0.34	-0.27	0.56
TCGA-B0-5402-01	FALSE	FALSE	0.37	0.69	0.71	0.06	0.44	-0.9
TCGA-B0-5115-01	FALSE	FALSE	0.36	0.22	0.39	-0.06	0.52	-0.8
TCGA-CJ-6028-01	FALSE	FALSE	0.36	1.87	0.58	1.37	1.27	2.28
TCGA-B0-4841-01	FALSE	FALSE	0.35	-0.73	0.11	-0.56	-0.77	-0.2
TCGA-B0-4836-01	FALSE	FALSE	0.35	0.48	0.5	0.44	-0.36	1.23
TCGA-BP-4976-01	FALSE	FALSE	0.35	-0.81	0.81	-0.7	-0.44	-1.6
TCGA-B8-5552-01	FALSE	FALSE	0.35	-0.04	0.33	0.16	0.53	0.65
TCGA-GK-A6C7-01	FALSE	FALSE	0.34	-0.27	0.39	-0.23	-0.39	-0.3
TCGA-CZ-5459-01	FALSE	FALSE	0.34	0.59	0.66	0.2	-0.19	0.69
TCGA-CZ-5982-01	FALSE	FALSE	0.33	-0.74	0.54	-0.72	0.1	-0.9
TCGA-B0-5399-01	FALSE	FALSE	0.32	0.41	0.52	0.21	0.11	-0.1
TCGA-CZ-5465-01	FALSE	FALSE	0.31	0.1	0.7	-0.48	1.23	-1.2
TCGA-A3-3322-01	FALSE	FALSE	0.31	-1.11	0.63	-0.87	-0.07	-1.4
TCGA-CW-5590-01	FALSE	FALSE	0.31	0.77	0.44	0.33	0.13	0.11
TCGA-CJ-6030-01	FALSE	FALSE	0.3	0.75	0.4	0.82	-0.1	1.19
TCGA-BP-4327-01	FALSE	FALSE	0.3	-0.69	0.46	-0.49	0.3	-0.4

TCGA-AK-3461-01	FALSE	FALSE	0.29	0.2	0.47	-0.25	0.25	-0.5
TCGA-B0-4811-01	FALSE	FALSE	0.29	-0.71	0.18	-0.74	-0.7	-1
TCGA-CW-6093-01	FALSE	FALSE	0.29	-0.91	0.36	-0.66	-0.22	-1.3
TCGA-BP-4776-01	FALSE	FALSE	0.29	-0.46	0.44	-0.15	-0.25	-0.2
TCGA-CJ-4875-01	FALSE	FALSE	0.28	-0.45	0.44	0.34	-0.6	2.34
TCGA-B8-5546-01	FALSE	FALSE	0.27	-1.9	0.76	-1.1	-0.04	-0.8
TCGA-CJ-5681-01	FALSE	FALSE	0.27	-1.6	0.66	-1.27	-0.15	-1.7
TCGA-B0-4839-01	FALSE	FALSE	0.26	-0.11	0.47	-0.04	-0.62	-0.5
TCGA-BP-4965-01	FALSE	FALSE	0.26	-0.29	0.42	-0.28	0.32	-0.4
TCGA-CJ-5678-01	FALSE	FALSE	0.24	1.85	0.84	1.48	0.29	2.23
TCGA-BP-4982-01	FALSE	FALSE	0.24	0.06	0.41	-0.09	-0.18	-0
TCGA-AK-3434-01	FALSE	FALSE	0.24	0.38	0.53	0.23	0.09	0.21
TCGA-DV-A4W0-01	FALSE	FALSE	0.23	-0.49	0.67	-0.57	-0.39	-1
TCGA-B0-5077-01	FALSE	FALSE	0.23	-0.05	0.51	-0.15	-0.44	0.04
TCGA-6D-AA2E-01	FALSE	FALSE	0.22	-1.49	0.86	-0.61	2.46	-0.2
TCGA-BP-4161-01	FALSE	FALSE	0.22	1.38	0.58	0.85	0.64	1.36
TCGA-BP-5168-01	FALSE	FALSE	0.22	0.19	0.67	0.02	-0.32	-0
TCGA-B0-5110-01	FALSE	FALSE	0.21	-0.03	0.48	-0.11	0.27	-0.4
TCGA-A3-3346-01	FALSE	FALSE	0.21	0.57	0.77	0.34	-0.18	0.38
TCGA-BP-4987-01	FALSE	FALSE	0.2	-0.12	0.58	0.07	0.07	0.62
TCGA-CZ-4865-01	FALSE	FALSE	0.19	1.03	0.5	0.63	0.11	0.9
TCGA-B0-4818-01	FALSE	FALSE	0.19	0.92	0.7	0.5	0.46	0.52
TCGA-B0-5108-01	FALSE	FALSE	0.19	0.95	0.26	0.75	0.94	-0
TCGA-A3-3380-01	FALSE	FALSE	0.19	-0.53	0.55	-0.39	-0.34	-0.6
TCGA-CJ-4638-01	FALSE	FALSE	0.18	-1.64	0.6	-0.69	-0.71	-0.1
TCGA-CJ-4893-01	FALSE	FALSE	0.17	0.91	0.68	0.95	0.64	1.91
TCGA-A3-3358-01	FALSE	FALSE	0.16	0.74	0.71	0.52	0.15	1.31
TCGA-B0-5702-01	FALSE	FALSE	0.15	-1.62	0.25	-0.35	-0.65	-1
TCGA-CZ-4863-01	FALSE	FALSE	0.15	1.61	0.76	1.26	0.66	2.79
TCGA-CJ-5682-01	FALSE	FALSE	0.13	-0.03	0.41	0.05	-0.4	0.18
TCGA-MW-A4EC-01	FALSE	FALSE	0.13	-0.61	0.81	-0.55	0.25	-0.6
TCGA-BP-4972-01	FALSE	FALSE	0.11	0.35	0.47	0.18	0.62	-0.1
TCGA-AK-3450-01	FALSE	FALSE	0.11	0.19	0.66	-0.3	-0.12	-0.4
TCGA-A3-A6NL-01	FALSE	FALSE	0.11	0.03	0.31	0.08	-0.43	-0.4
TCGA-AK-3429-01	FALSE	FALSE	0.11	2.13	0.86	1.63	0.63	2.65
TCGA-B0-4718-01	FALSE	FALSE	0.1	0.11	0.25	0.18	0.81	0.49
TCGA-BP-5185-01	FALSE	FALSE	0.09	-1.17	0.4	-0.51	-0.34	0.25
TCGA-CJ-6027-01	FALSE	FALSE	0.09	2.06	0.78	1.57	0.8	2.05
TCGA-CZ-5987-01	FALSE	FALSE	0.09	0.51	0.75	0.23	-0.34	0.08
TCGA-BP-5009-01	FALSE	FALSE	0.08	-0.21	0.23	0.06	-0.57	1.09
TCGA-DV-5569-01	FALSE	FALSE	0.08	0.17	0.52	0.1	0.58	0.35
TCGA-CJ-4640-01	FALSE	FALSE	0.08	1.75	0.55	1.33	0.4	1.95
TCGA-AK-3460-01	FALSE	FALSE	0.08	-0.15	0.68	-0.2	-0.04	-0.1
TCGA-BP-5008-01	FALSE	FALSE	0.08	0.31	0.47	0.33	0.57	0.88
TCGA-BP-4991-01	FALSE	FALSE	0.07	-0.34	0.3	-0.55	-0.38	-1.2
TCGA-BP-5184-01	FALSE	FALSE	0.07	-0.42	0.61	-0.04	0.25	0.3
TCGA-BP-5182-01	FALSE	FALSE	0.07	0.41	0.34	0.72	0.33	0.81
TCGA-BP-4341-01	FALSE	FALSE	0.07	-1.44	0.34	-0.93	-0.01	-1.7
TCGA-B0-4693-01	FALSE	FALSE	0.05	0.49	0.74	0.49	0.64	1.24
TCGA-CZ-4858-01	FALSE	FALSE	0.05	0.81	0.48	1.02	-0.31	2.11
TCGA-G6-A8L6-01	FALSE	FALSE	0.04	-0.44	0.46	-0.08	-0.25	-0.2
TCGA-B0-5703-01	FALSE	FALSE	0.04	-0.29	0.55	-0.12	-0.57	0.52
TCGA-B0-5109-01	FALSE	FALSE	0.03	0.71	0.39	0.72	0.18	1.63
TCGA-CJ-4904-01	FALSE	FALSE	0.02	1.48	0.47	0.49	0.51	0.26
TCGA-BP-5174-01	FALSE	FALSE	0.02	-0.55	0.66	-0.37	-0.49	-0.1
TCGA-BP-4768-01	FALSE	FALSE	0.02	-1.13	0.52	-0.74	-0.46	-0.2
TCGA-EU-5904-01	FALSE	FALSE	0.02	0.05	0.49	-0.11	0.41	-0.2
TCGA-BP-4971-01	FALSE	FALSE	0.01	0.15	0.08	0.07	0.46	-0.5

TCGA-BP-4801-01	FALSE	FALSE	0.01	0.25	0.53	0.31	0.51	0.16
TCGA-BP-4998-01	FALSE	FALSE	0.01	0.51	0.26	0.26	0.36	0.44
TCGA-AK-3428-01	FALSE	FALSE	0	0.19	0.83	-0.27	0.57	-0.4
TCGA-B0-4852-01	FALSE	FALSE	0	0.2	0.61	-0.28	-0.12	-0.3
TCGA-A3-3313-01	FALSE	TRUE	0	-1.28	0.52	-1.2	1.92	-1.8
TCGA-CJ-4642-01	FALSE	TRUE	0	-2.44	0.02	-1.94	-1.26	-1.3
TCGA-CZ-5451-01	FALSE	TRUE	0	0.07	0.79	0.11	0.16	0.01
TCGA-B0-4713-01	FALSE	TRUE	-0	-0.68	0.8	-0.24	-0.73	0.18
TCGA-BP-5173-01	FALSE	TRUE	-0	0.65	0.5	0.98	0.33	1.71
TCGA-B8-5158-01	FALSE	TRUE	-0	0.63	0.44	0.35	-0.17	0.79
TCGA-A3-3335-01	FALSE	TRUE	-0	-0.73	0.91	-0.66	1.79	-0.9
TCGA-B2-3924-01.1	FALSE	TRUE	-0	2.42	0.87	1.71	1.13	2.42
TCGA-B0-5102-01	FALSE	TRUE	-0.1	0.24	0.76	0.06	0.6	0.14
TCGA-A3-3373-01	FALSE	TRUE	-0.1	0.06	0.5	-0.16	0.1	-0.8
TCGA-B4-5832-01	FALSE	TRUE	-0.1	0.06	0.71	-0.9	-0.16	-0.5
TCGA-BP-4347-01	FALSE	TRUE	-0.1	0.21	0.47	-0.08	0.47	-0.3
TCGA-CZ-4859-01	FALSE	TRUE	-0.1	-0.36	0.89	-0.46	-0.15	-0.7
TCGA-BP-4969-01	FALSE	TRUE	-0.1	-1.14	0.51	-1.01	-0.4	-1.4
TCGA-AK-3427-01	FALSE	TRUE	-0.1	-1.56	0.99	-1.56	-0.29	-1.8
TCGA-BP-5189-01	FALSE	TRUE	-0.1	0.32	0.38	0.1	-0.22	-0.5
TCGA-BP-4774-01	FALSE	TRUE	-0.1	-0.52	0.41	-0.31	-0.23	-0.2
TCGA-B0-5701-01	FALSE	TRUE	-0.1	0.69	0.59	0.3	-0.31	0.2
TCGA-A3-3382-01	FALSE	TRUE	-0.1	0.11	0.33	-0.02	-0.25	0.43
TCGA-B0-4690-01	FALSE	TRUE	-0.1	0.98	0.4	0.78	0.31	1.21
TCGA-BP-4790-01	FALSE	TRUE	-0.1	0.81	0.34	0.81	0.06	1.65
TCGA-AK-3465-01	FALSE	TRUE	-0.1	-1.17	0.87	-1.69	0.03	-1.9
TCGA-BP-5200-01	FALSE	TRUE	-0.1	0.37	0.33	0.57	0.77	1.23
TCGA-B0-4828-01	FALSE	TRUE	-0.1	-0.53	0.56	-0.49	-0.32	-0.2
TCGA-A3-3363-01	FALSE	TRUE	-0.1	-1.35	0.91	-0.85	0	-1
TCGA-B0-5083-01	FALSE	TRUE	-0.1	-0.4	0.85	-0.12	0.19	0.04
TCGA-B8-4153-01	FALSE	TRUE	-0.2	-0.58	0.49	-0.51	-0.47	-0.5
TCGA-B8-4619-01	FALSE	TRUE	-0.2	-1.45	0.85	-1.25	0.05	-1.5
TCGA-CJ-4644-01	FALSE	TRUE	-0.2	0.83	0.77	0.8	0.47	1.57
TCGA-B8-A54H-01	FALSE	TRUE	-0.2	-0.25	0.52	0.07	-0.27	1.45
TCGA-BP-4160-01	FALSE	TRUE	-0.2	1.65	0.88	1.43	0.3	3.07
TCGA-CJ-4891-01	FALSE	TRUE	-0.2	-0.49	0.4	-0.3	-0.24	-0.6
TCGA-CJ-4639-01	FALSE	TRUE	-0.2	0.85	0.68	0.76	0.56	1.69
TCGA-BP-4163-01	FALSE	TRUE	-0.2	-0.15	0.45	-0.26	-0.46	-0.4
TCGA-B0-4698-01	FALSE	TRUE	-0.2	2.15	0.64	1.71	3.07	2.45
TCGA-CZ-5458-01	FALSE	TRUE	-0.2	0.26	0.49	0.08	-0.07	0.29
TCGA-B0-4699-01	FALSE	TRUE	-0.2	0.49	0.84	0.84	2.37	1.99
TCGA-B0-5700-01	FALSE	TRUE	-0.2	-0.34	0.75	-0.23	-0.26	-0.7
TCGA-B0-4945-01	FALSE	TRUE	-0.2	-0.19	0.41	-0.32	0.24	-0.4
TCGA-BP-4353-01	FALSE	TRUE	-0.2	0.43	0.42	0.32	0.06	0.61
TCGA-AK-3456-01	FALSE	TRUE	-0.2	-0.96	0.77	-0.77	1.31	-1.3
TCGA-A3-3372-01	FALSE	TRUE	-0.2	0.58	0.25	0.1	0.15	0.03
TCGA-CZ-5457-01	FALSE	TRUE	-0.2	0.35	0.72	0.27	0.55	0.57
TCGA-AK-3451-01	FALSE	TRUE	-0.2	-0.53	0.97	0	-0.62	1.42
TCGA-CZ-5455-01	FALSE	TRUE	-0.2	2.21	0.68	1.41	1.41	2.42
TCGA-B0-4834-01	FALSE	TRUE	-0.2	-2.25	0.86	-1.46	1.1	-1.6
TCGA-B2-5633-01.1	FALSE	TRUE	-0.2	0.21	0.48	-0.02	-0.33	-0
TCGA-CJ-4876-01	FALSE	TRUE	-0.2	0.23	0.54	0.52	0.15	1.82
TCGA-T7-A92I-01	FALSE	TRUE	-0.2	-1.16	0.8	-0.62	2.76	-0.8
TCGA-CJ-4874-01	FALSE	TRUE	-0.3	1.25	0.84	0.98	0.27	2.06
TCGA-EU-5906-01	FALSE	TRUE	-0.3	-0.08	0.61	-0.18	0.19	-0.5
TCGA-BP-5202-01	FALSE	TRUE	-0.3	-0.15	0.73	-0.34	0.11	-0.8
TCGA-CZ-5984-01	FALSE	TRUE	-0.3	0.16	0.53	0.07	-0.32	-0.3
TCGA-CJ-4878-01	FALSE	TRUE	-0.3	-0.6	0.45	-0.86	0.25	-1.8

TCGA-CZ-5454-01	FALSE	TRUE	-0.3	0.28	0.7	0.4	0.37	0.02
TCGA-B0-5119-01	FALSE	TRUE	-0.3	0.05	0.75	0.27	0.42	1.08
TCGA-A3-3311-01	FALSE	TRUE	-0.3	0.47	0.57	0.26	0.06	0.44
TCGA-CZ-5470-01	FALSE	TRUE	-0.3	1.13	0.75	0.77	0.66	1.34
TCGA-B0-5713-01	FALSE	TRUE	-0.3	0.69	0.65	0.15	0.42	0.11
TCGA-BP-5178-01	FALSE	TRUE	-0.3	-0.62	0.47	-0.06	-0.62	-0.3
TCGA-CJ-4643-01	FALSE	TRUE	-0.3	0.44	0.41	0.03	0.65	-0.3
TCGA-A3-3306-01	FALSE	TRUE	-0.3	0	0.94	-0.45	-0.07	-0.7
TCGA-CZ-5985-01	FALSE	TRUE	-0.3	1.02	0.71	0.61	0.29	0.36
TCGA-B0-4842-01	FALSE	TRUE	-0.3	-0.37	0.41	0.49	-0.42	0.53
TCGA-BP-5180-01	FALSE	TRUE	-0.3	0.46	0.84	0.15	-0.41	0.36
TCGA-BP-5007-01	FALSE	TRUE	-0.3	0.19	0.45	0.09	-0.01	0.44
TCGA-BP-4799-01	FALSE	TRUE	-0.3	0.35	0.25	0.23	0.08	0.96
TCGA-CJ-5686-01	FALSE	TRUE	-0.3	0.54	0.67	0.27	0.09	0.4
TCGA-B8-5159-01	FALSE	TRUE	-0.3	0.14	0.75	0.4	0.33	1.39
TCGA-BP-4164-01	FALSE	TRUE	-0.4	-0.06	0.8	-0.36	0.47	-0.3
TCGA-B0-5117-01	FALSE	TRUE	-0.4	-1.92	0.84	-1.64	0.23	-1.9
TCGA-BP-5006-01	FALSE	TRUE	-0.4	-0.71	0.64	-0.67	-0.24	-1.2
TCGA-B0-5711-01	FALSE	TRUE	-0.4	0.46	0.58	0.28	0.43	0.13
TCGA-BP-4797-01	FALSE	TRUE	-0.4	0.49	0.67	0.42	-0.09	0.81
TCGA-BP-4338-01	FALSE	TRUE	-0.4	-0.94	0.5	-0.44	0.18	-0.9
TCGA-A3-3323-01	FALSE	TRUE	-0.4	0.28	0.34	0.08	-0.41	-0.2
TCGA-CW-5581-01	FALSE	TRUE	-0.4	0.33	0.74	0.05	0.27	-0.2
TCGA-BP-5190-01	FALSE	TRUE	-0.4	-0.71	0.74	-0.46	-0.37	-0.7
TCGA-A3-3319-01	FALSE	TRUE	-0.4	-0.26	0.52	-0.47	-0.69	-0.2
TCGA-B0-5107-01	FALSE	TRUE	-0.4	0.36	0.63	0.67	-0.48	1.74
TCGA-B2-5635-01.1	FALSE	TRUE	-0.4	0.64	0.73	0.46	0.22	1.27
TCGA-B8-4151-01	FALSE	TRUE	-0.4	0.21	0.92	-0.29	0.1	-0.9
TCGA-BP-4354-01	FALSE	TRUE	-0.4	0.74	0.34	0.7	0.7	-0.1
TCGA-BP-4174-01	FALSE	TRUE	-0.4	0.53	0.82	0.48	1.23	0.55
TCGA-DV-A4VX-01	FALSE	TRUE	-0.4	-0.71	0.71	-0.37	-0.49	0.33
TCGA-A3-3383-01	FALSE	TRUE	-0.4	0.93	0.74	0.68	-0.32	0.17
TCGA-A3-3367-01	FALSE	TRUE	-0.4	0.11	0.58	-0.24	0.49	-0.8
TCGA-A3-3385-01	FALSE	TRUE	-0.4	-1.28	0.87	-1.1	0.13	-1.6
TCGA-AS-3777-01	FALSE	TRUE	-0.4	-1.31	0.95	-1.28	-0.49	-1.4
TCGA-B2-A4SR-01	FALSE	TRUE	-0.5	0.5	0.32	0.04	0.63	0.02
TCGA-BP-4981-01	FALSE	TRUE	-0.5	-0.3	0.36	-0.1	-0.74	-0.2
TCGA-B0-4701-01	FALSE	TRUE	-0.5	0.87	0.59	0.43	0.48	0.48
TCGA-CJ-4635-01	FALSE	TRUE	-0.5	0.44	0.45	0.56	-0.29	1.78
TCGA-A3-A8OV-01	FALSE	TRUE	-0.5	-0.3	0.85	-0.37	-0.04	-1.1
TCGA-BP-4977-01	FALSE	TRUE	-0.6	-0.2	0.56	-0.33	-0.17	-0.6
TCGA-B4-5835-01	FALSE	TRUE	-0.6	-0.43	0.53	-0.22	-0.27	-0.5
TCGA-CZ-5986-01	FALSE	TRUE	-0.6	0.54	0.68	-0.13	1.16	-1.1
TCGA-B0-5113-01	FALSE	TRUE	-0.6	0.61	0.5	0.35	0.66	0.39
TCGA-B8-5549-01	FALSE	TRUE	-0.6	0.22	0.58	0.41	0.17	1.37
TCGA-B2-3923-01	FALSE	TRUE	-0.6	-1.57	0.65	-2	1.5	-2.4
TCGA-B8-4622-01	FALSE	TRUE	-0.6	0.43	0.39	0.04	-0.35	-0.5
TCGA-B0-5121-01	FALSE	TRUE	-0.6	-0.7	0.24	-0.28	-0.19	-0.1
TCGA-BP-4766-01	FALSE	TRUE	-0.6	-1.57	0.47	-1.11	-0.5	-1.6
TCGA-A3-3328-01	FALSE	TRUE	-0.6	-2.25	0.99	-1.8	0.23	-2
TCGA-A3-3307-01	FALSE	TRUE	-0.6	0.76	0.73	0.33	0.41	0.85
TCGA-A3-A8OW-01	FALSE	TRUE	-0.6	-0.3	0.46	0.1	0.26	0.54
TCGA-B0-5088-01	FALSE	TRUE	-0.6	-0.39	0.3	-0.01	-0.24	-0.1
TCGA-B0-4817-01	FALSE	TRUE	-0.7	-0.69	0.69	-0.6	-0.63	-0.9
TCGA-BP-4775-01	FALSE	TRUE	-0.7	0.09	0.65	-0.12	0.44	-0.7
TCGA-B8-4621-01	FALSE	TRUE	-0.7	-0.8	0.75	-0.48	-0.41	-0.6
TCGA-BP-4331-01	FALSE	TRUE	-0.7	0	0.58	0	-0.07	-0.2
TCGA-CJ-6032-01	FALSE	TRUE	-0.7	0.22	0.62	0.09	0.05	0.6

TCGA-CZ-4853-01	FALSE	TRUE	-0.7	0.57	0.88	-0.21	0.56	-1.2
TCGA-BP-4340-01	FALSE	TRUE	-0.7	-0.09	0.62	-0.59	-0.25	-1.2
TCGA-BP-4176-01	FALSE	TRUE	-0.8	-0.06	0.47	0.13	0.15	0.02
TCGA-BP-4994-01	FALSE	TRUE	-0.8	-0.66	0.85	-0.65	0.24	-0.4
TCGA-B8-A54I-01	FALSE	TRUE	-0.8	-0.17	0.43	0.33	0.35	-0.4
TCGA-G6-A5PC-01	FALSE	TRUE	-0.8	-1.01	0.71	0.13	-0.78	1.47
TCGA-B4-5377-01	FALSE	TRUE	-0.8	0.29	0.79	0.17	0.49	0.79
TCGA-CZ-5989-01	FALSE	TRUE	-0.8	1.14	0.83	-0.09	1.28	1.06
TCGA-AK-3447-01	FALSE	TRUE	-0.8	-1.54	1	-1.56	-0.32	-1.5
TCGA-AK-3433-01	FALSE	TRUE	-0.8	-1.33	0.94	-1.34	0.18	-1.7
TCGA-AK-3455-01	FALSE	TRUE	-0.8	1.65	0.92	1.25	-0.01	3.21
TCGA-CJ-4903-01	FALSE	TRUE	-0.8	0.33	0.48	0.41	0.23	0.82
TCGA-B8-A54E-01	FALSE	TRUE	-0.8	-0.78	0.9	-0.87	0.38	-1.1
TCGA-CJ-4873-01	FALSE	TRUE	-0.9	0.07	0.27	0.61	-0.12	0.78
TCGA-BP-4756-01	FALSE	TRUE	-0.9	-0.18	0.7	-0.31	1.29	0.02
TCGA-B2-3923-01.1	FALSE	TRUE	-0.9	-1	0.97	-1.45	0.98	-1.9
TCGA-AK-3440-01	FALSE	TRUE	-0.9	-1.45	0.98	-1.4	1.29	-1.7
TCGA-BP-4995-01	FALSE	TRUE	-0.9	-0.25	0.8	-0.3	0.34	0.08
TCGA-BP-4170-01	FALSE	TRUE	-0.9	1.25	0.72	0.65	0.28	0.8
TCGA-AK-3443-01	FALSE	TRUE	-0.9	-2.12	0.96	-1.69	1.19	-1.9
TCGA-B0-4816-01	FALSE	TRUE	-0.9	-0.18	0.33	-0.04	-0.52	-0.2
TCGA-B0-5085-01	FALSE	TRUE	-0.9	-0.96	0.38	-0.69	-0.31	-0.7
TCGA-B0-5104-01	FALSE	TRUE	-0.9	-0.48	0.63	-0.64	-0.09	-0.9
TCGA-A3-3324-01	FALSE	TRUE	-0.9	0.22	0.58	0.2	0.61	0.78
TCGA-B2-4099-01	FALSE	TRUE	-0.9	1	0.91	0.21	0.1	-0.4
TCGA-B2-5639-01	FALSE	TRUE	-0.9	0.4	0.75	0.15	0.11	-0.5
TCGA-B8-4154-01	FALSE	TRUE	-0.9	1.12	0.96	0.38	-0.01	0.71
TCGA-BP-4335-01	FALSE	TRUE	-0.9	1.07	0.4	0.43	0.34	0.97
TCGA-CZ-4866-01	FALSE	TRUE	-1	-0.6	0.85	-0.58	0.29	-0.9
TCGA-CJ-4636-01	FALSE	TRUE	-1	0.56	0.33	0.69	0.05	1.59
TCGA-B0-4849-01	FALSE	TRUE	-1	-0.06	0.41	-0.25	-0.04	-0.4
TCGA-A3-3329-01	FALSE	TRUE	-1	1.09	0.79	0.24	0.12	0.15
TCGA-BP-4334-01	FALSE	TRUE	-1	-2.16	0.8	-1.55	-0.08	-2
TCGA-AK-3458-01	FALSE	TRUE	-1.1	-0.33	0.96	-0.38	-0.59	0.35
TCGA-B8-4146-01	FALSE	TRUE	-1.1	2.02	0.96	0.33	1.83	0.48
TCGA-BP-4959-01	FALSE	TRUE	-1.1	-0.5	0.8	-0.04	-0.28	0.04
TCGA-B2-5633-01	FALSE	TRUE	-1.1	-0.94	0.03	-1.11	-0.42	-1.3
TCGA-CZ-5463-01	FALSE	TRUE	-1.1	1.05	0.84	0.43	0.54	0.32
TCGA-A3-3331-01	FALSE	TRUE	-1.2	0.58	0.57	0.18	0.15	0.07
TCGA-B0-5699-01	FALSE	TRUE	-1.2	0.47	0.63	-0.12	-0.08	-0.8
TCGA-A3-3320-01	FALSE	TRUE	-1.2	0.05	0.67	-0.22	0.24	-0.3
TCGA-B2-4098-01	FALSE	TRUE	-1.2	-0.8	0.67	-0.38	1.11	-1
TCGA-CJ-4894-01	FALSE	TRUE	-1.2	1.41	0.35	1.22	0.39	2.44
TCGA-CZ-5460-01	FALSE	TRUE	-1.3	0.35	0.7	0.62	1.33	1.39
TCGA-BP-4963-01	FALSE	TRUE	-1.3	0.16	0.46	0.03	-0.52	0
TCGA-BP-4973-01	FALSE	TRUE	-1.4	0.44	0.54	0.19	0.35	0.69
TCGA-CJ-4641-01	FALSE	TRUE	-1.4	1.48	0.64	1.34	0.62	2.11
TCGA-B0-5705-01	FALSE	TRUE	-1.4	0.74	0.78	0.51	0.63	0.91
TCGA-A3-3370-01	FALSE	TRUE	-1.4	0.91	0.57	0.86	0.72	1.72
TCGA-CZ-5462-01	FALSE	TRUE	-1.4	0.69	0.82	0.08	0.52	-0.9
TCGA-CJ-4884-01	FALSE	TRUE	-1.4	0.41	0.64	0.5	0.26	1.08
TCGA-B0-4846-01	FALSE	TRUE	-1.5	1.78	0.72	0.89	1.3	1.07
TCGA-A3-3352-01	FALSE	TRUE	-1.5	0.33	0.69	-0.15	0.24	-0.9
TCGA-BP-4798-01	FALSE	TRUE	-1.6	2.32	0.22	1.68	0.23	3.27
TCGA-BP-4992-01	FALSE	TRUE	-1.6	0.05	0.13	0.22	-0.28	1.16
TCGA-CZ-4861-01	FALSE	TRUE	-1.6	1.09	0.81	0.73	0.65	0.27
TCGA-B2-3924-01	FALSE	TRUE	-1.7	0.81	0.15	-0.1	0.77	0.25
TCGA-A3-3374-01	FALSE	TRUE	-1.7	-1.42	0.83	-0.58	1.95	-1.5

TCGA-BP-4325-01	FALSE	TRUE	-1.8	0.51	0.83	0.49	0.7	1.67
TCGA-CJ-5672-01	FALSE	TRUE	-1.8	1.33	0.48	1.41	0.26	2.62
TCGA-B2-5635-01	FALSE	TRUE	-1.9	-0.11	0.21	-0.62	-0.05	0.3
TCGA-A3-3378-01	FALSE	TRUE	-1.9	0.76	0.47	0.7	0.97	0.97
TCGA-B0-4838-01	FALSE	TRUE	-1.9	1.3	0.53	1.21	1.08	3.29
TCGA-A3-3343-01	FALSE	TRUE	-2	0.94	0.7	0.28	0.13	-0.6
TCGA-B2-4102-01	FALSE	TRUE	-2.1	0.5	0.53	0.02	0.36	-0.7
TCGA-A3-3351-01	FALSE	TRUE	-2.2	0.87	0.34	0.83	0.35	1.93
TCGA-A3-3357-01	FALSE	TRUE	-2.2	-0.11	0.82	0.34	-0.04	0.86
TCGA-B0-5698-01	FALSE	TRUE	-2.3	1.01	0.64	0.47	0.17	0.32
TCGA-CZ-5452-01	FALSE	TRUE	-2.4	1.72	0.73	1.55	0.08	2.86
TCGA-A3-3326-01	FALSE	TRUE	-2.6	1.03	0.49	0.52	0.76	-0.2
TCGA-B0-5075-01	FALSE	TRUE	-2.7	-0.16	0.8	0.31	0.05	1.03
TCGA-CZ-4864-01	FALSE	TRUE	-2.8	1.9	0.7	1.32	1	2.02
TCGA-B8-A54G-01	FALSE	TRUE	-3	1.69	0.77	1.55	-0.02	3.2

CTL.flag	Dysfunction	Exclusion	MDSC	CAF	TAM M2
FALSE	-0.17	3.28	0.17	0.31	0.02
FALSE	0.32	2.98	0.15	0.25	0.06
FALSE	0.47	2.82	0.11	0.32	0.01
FALSE	0.32	2.63	0.14	0.22	0.04
FALSE	-0.39	2.52	0.09	0.25	0.04
FALSE	-0.83	2.42	0.16	0.15	0.05
FALSE	0.92	2.33	0.08	0.26	0.01
FALSE	-0.77	2.33	0.19	0.12	0.05
TRUE	2.24	-2.1	-0.11	-0.13	-0.07
FALSE	0.59	2.21	0.11	0.27	-0.04
FALSE	-0.67	2.21	0.19	0.13	0.02
FALSE	-0.13	2.18	0.1	0.23	0.01
FALSE	0.48	2.15	0.04	0.26	0.04
FALSE	0.98	2.13	0.08	0.24	0.02
TRUE	2.07	0.21	-0.05	0.12	-0.04
FALSE	-0.22	2.05	0.14	0.18	0
TRUE	2.03	-2.06	-0.14	-0.03	-0.15
FALSE	0.65	2.03	0.05	0.21	0.05
FALSE	0.02	2.01	0.09	0.18	0.04
FALSE	-0.29	1.99	0.09	0.23	-0.02
FALSE	-0.29	1.99	0.07	0.17	0.07
FALSE	-0.3	1.97	0.12	0.13	0.05
TRUE	1.96	-0.68	-0.05	0.01	-0.06
FALSE	0.24	1.92	0.05	0.19	0.06
FALSE	0.83	1.91	0.07	0.2	0.03
FALSE	0.07	1.88	-0.01	0.22	0.07
FALSE	-1.13	1.85	0.23	0	0.04
FALSE	0.86	1.85	0.02	0.3	-0.03
FALSE	-0.43	1.83	0.11	0.11	0.05
FALSE	0.5	1.82	0.09	0.15	0.04
FALSE	0.2	1.79	0.03	0.22	0.04
FALSE	-0.32	1.78	0.09	0.16	0.03
FALSE	-0.41	1.78	0.06	0.18	0.03
FALSE	-1.81	1.77	0.09	0.12	0.06
TRUE	1.77	-0.99	-0.06	-0.03	-0.06
FALSE	0.6	1.76	0.08	0.18	0
FALSE	-0.16	1.74	0.12	0.13	0.02
TRUE	1.74	-2.73	-0.14	-0.19	-0.09
FALSE	-0.34	1.72	0.17	0.05	0.04
TRUE	1.72	-1.23	-0.07	-0.02	-0.09
FALSE	0.42	1.72	0.03	0.28	-0.04
FALSE	0.23	1.7	0.11	0.19	-0.04
FALSE	0.15	1.7	0.05	0.16	0.04
FALSE	-1.32	1.69	0.07	0.12	0.07
FALSE	0.53	1.68	0.03	0.15	0.08
FALSE	0.31	1.68	0.03	0.2	0.02
TRUE	1.62	0.83	-0.06	0.22	-0.03
TRUE	1.62	0.48	-0.06	0.15	-0.01
TRUE	1.61	-1.14	-0.01	-0.14	-0.02
FALSE	0.1	1.57	0.04	0.2	0.01
TRUE	1.57	-0.66	-0.01	-0.03	-0.07
TRUE	1.55	-1.26	-0.06	-0.09	-0.04
TRUE	1.55	-1.83	-0.09	-0.15	-0.03
TRUE	1.55	-3.22	-0.16	-0.25	-0.08
TRUE	1.52	0.36	0.02	0.08	-0.04
FALSE	-0.08	1.52	0.04	0.14	0.05

TRUE	1.51	0.46	-0.04	0.1	0.01
FALSE	0.25	1.5	0.06	0.13	0.04
FALSE	-0.26	1.48	0.07	0.11	0.05
TRUE	1.46	0.75	0.03	0.13	-0.04
TRUE	1.45	-1.04	0.02	-0.1	-0.06
TRUE	1.44	-1.94	-0.14	-0.13	-0.03
TRUE	1.43	-0.37	-0.16	0.17	-0.06
TRUE	1.42	0.77	0.03	0.17	-0.08
FALSE	-0.4	1.41	0.17	0.06	-0.02
TRUE	1.41	-0.9	-0.02	-0.02	-0.1
TRUE	1.4	-1.95	-0.1	-0.17	-0.03
FALSE	-1.2	1.39	0.07	0.13	0.01
FALSE	0.39	1.38	-0.01	0.21	0.01
FALSE	-0.36	1.38	0.11	0.06	0.03
TRUE	1.37	-2.05	-0.09	-0.18	-0.05
TRUE	1.35	0.02	-0.09	0.15	-0.05
FALSE	-0.15	1.35	0.11	0.02	0.07
FALSE	0.56	1.33	-0.02	0.21	0.01
FALSE	-0.51	1.33	0.13	0.01	0.06
FALSE	0.45	1.32	-0.01	0.18	0.04
FALSE	0.32	1.32	0.02	0.15	0.03
TRUE	1.32	-0.72	-0.14	0.08	-0.06
FALSE	-0.99	1.31	0.11	0.05	0.03
FALSE	-0.42	1.31	0.07	0.17	-0.04
FALSE	-0.47	1.3	0.1	0.12	-0.03
TRUE	1.3	0.39	0.01	0.06	0
FALSE	-0.89	1.3	0.06	0.12	0.01
TRUE	1.29	-1.5	-0.1	-0.07	-0.06
FALSE	-0.32	1.29	0.1	0.04	0.05
TRUE	1.26	-2.28	-0.1	-0.17	-0.07
FALSE	1.34	1.25	-0.04	0.24	-0.01
FALSE	-0.41	1.25	0.02	0.15	0.01
FALSE	0.45	1.24	0.12	0.06	0.02
FALSE	-0.41	1.24	0.1	0.04	0.05
TRUE	1.22	0.78	-0.03	0.15	-0.01
FALSE	-0.21	1.21	0.02	0.12	0.04
TRUE	1.2	-0.19	0	-0.02	0
TRUE	1.2	-1.19	0.01	-0.15	-0.04
TRUE	1.19	1.02	-0.05	0.2	0.01
FALSE	-0.52	1.19	-0.02	0.2	0.01
TRUE	1.19	-2.38	-0.06	-0.26	-0.05
FALSE	-0.45	1.19	0.16	-0.02	0.03
FALSE	-0.68	1.19	0.02	0.11	0.05
TRUE	1.18	-1.86	-0.03	-0.21	-0.05
FALSE	0.05	1.18	0.1	0.12	-0.05
TRUE	1.17	-1.93	-0.04	-0.21	-0.04
FALSE	0.04	1.16	0.03	0.21	-0.06
FALSE	0.35	1.15	0.01	0.18	-0.01
FALSE	-0.44	1.15	0.1	0.05	0.03
FALSE	0.28	1.14	0.06	0.1	0.02
FALSE	-0.01	1.12	0.07	0.19	-0.09
TRUE	1.11	-1.63	-0.09	-0.1	-0.07
TRUE	1.1	-1.36	-0.09	0	-0.12
TRUE	1.1	-0.49	-0.12	0.07	-0.03
FALSE	0.4	1.1	0.06	0.09	0.02
FALSE	0.26	1.09	0.03	0.08	0.05
TRUE	1.09	-1.2	-0.03	-0.07	-0.08
TRUE	1.05	-1.11	-0.02	-0.09	-0.06

FALSE	0.42	1.04	0.1	0.01	0.06
TRUE	1.04	0.27	-0.07	0.1	0.01
FALSE	-0.59	1.04	0.04	0.05	0.06
FALSE	1.25	1.03	-0.07	0.23	-0.01
FALSE	1.02	1.03	0.03	0.1	0.03
TRUE	1.03	-2.52	-0.1	-0.21	-0.08
FALSE	-0.12	1.03	0.06	0.07	0.02
FALSE	0.68	1.02	0.01	0.15	0
TRUE	1.02	1	0.08	0.11	-0.05
FALSE	0.22	1.02	0.03	0.08	0.04
FALSE	-0.37	1.01	0.08	0.06	0.01
FALSE	-0.23	1.01	0.12	0.01	0.02
FALSE	-0.13	1	0.11	0	0.03
TRUE	0.99	-1.68	-0.12	-0.11	-0.03
FALSE	-0.01	0.99	0.08	0.12	-0.05
FALSE	-1.04	0.99	0.06	0.04	0.05
FALSE	0	0.99	0	0.09	0.06
FALSE	-0.18	0.98	0.1	0.09	-0.04
FALSE	-0.04	0.97	0.03	0.11	0
FALSE	-0.15	0.97	0.02	0.1	0.02
FALSE	0.4	0.97	-0.01	0.17	-0.01
FALSE	0.13	0.96	-0.01	0.13	0.03
FALSE	-0.35	0.96	0.13	0.01	0
FALSE	-0.69	0.95	0.02	0.12	-0.01
FALSE	0.31	0.95	-0.02	0.15	0.01
FALSE	-0.16	0.95	0.08	0.05	0.01
FALSE	0.47	0.94	0.09	0.01	0.03
TRUE	0.93	-0.12	0.01	-0.03	0
FALSE	-0.54	0.92	0.02	0.1	0.02
TRUE	0.92	-1.78	-0.06	-0.11	-0.11
FALSE	-0.52	0.91	0.09	0.02	0.03
FALSE	0.08	0.91	0.1	0.06	-0.03
FALSE	0.46	0.9	0.04	0.09	0
FALSE	-0.48	0.9	-0.01	0.07	0.08
TRUE	0.89	-0.64	-0.06	0.01	-0.04
FALSE	0.04	0.89	0.09	0.12	-0.06
FALSE	0.42	0.88	0.06	0.06	0.02
FALSE	-0.09	0.88	0.1	0.01	0.02
TRUE	0.88	0.34	-0.06	0.15	-0.03
FALSE	-0.39	0.88	0.03	0.05	0.05
FALSE	-0.39	0.86	0.06	0.08	-0.02
FALSE	-0.16	0.85	0	0.09	0.03
FALSE	-0.17	0.85	0.06	0.03	0.04
TRUE	0.84	1.42	0.06	0.23	-0.07
TRUE	0.84	-0.39	-0.03	-0.01	-0.04
TRUE	0.84	-1.03	-0.1	-0.04	-0.01
TRUE	0.84	-0.51	-0.01	-0.06	-0.01
FALSE	-0.57	0.84	-0.07	0.16	0.04
TRUE	0.84	-0.4	-0.08	-0.01	0.03
FALSE	-0.31	0.83	0.08	0.01	0.03
FALSE	-0.62	0.83	0.06	0.12	-0.04
FALSE	0.11	0.83	-0.01	0.13	0
FALSE	-0.09	0.81	0.07	0.08	-0.03
FALSE	0.67	0.81	-0.02	0.18	-0.03
FALSE	-0.39	0.81	0.03	0.05	0.04
FALSE	-0.34	0.8	0	0.1	0.01
FALSE	0.04	0.8	0.05	0.04	0.03
FALSE	-0.32	0.8	0.03	0.05	0.04

TRUE	0.79	0.6	-0.01	0.17	-0.06
FALSE	-0.77	0.78	0.13	-0.04	0.02
FALSE	0.09	0.78	0.03	0.08	0.01
FALSE	0.15	0.77	0.13	-0.03	0.02
TRUE	0.77	-1.17	-0.01	-0.15	-0.01
TRUE	0.77	-0.71	-0.04	-0.04	-0.03
FALSE	-0.17	0.75	0	0.11	-0.01
FALSE	0.33	0.75	0.04	0.11	-0.03
TRUE	0.74	-1.16	-0.04	-0.11	-0.04
FALSE	0.78	0.74	-0.01	0.18	-0.05
FALSE	-0.78	0.73	-0.02	0.08	0.06
FALSE	0.43	0.73	-0.03	0.18	-0.04
FALSE	0.19	0.73	0.03	0.03	0.06
FALSE	-0.78	0.73	0.02	0.09	-0.01
TRUE	0.72	-2.45	-0.16	-0.17	-0.04
FALSE	0.34	0.72	0.07	0.03	0.02
TRUE	0.71	1.7	0.11	0.18	-0.03
FALSE	0.16	0.71	0.02	0.05	0.04
TRUE	0.7	0.01	0.04	-0.06	0.03
FALSE	0.16	0.7	0	0.02	0.09
TRUE	0.69	0.91	0.03	0.13	-0.02
FALSE	-0.36	0.69	0.05	-0.02	0.07
FALSE	-0.33	0.69	-0.01	0.1	0.03
TRUE	0.68	1.49	0	0.2	0.02
FALSE	-0.5	0.66	0.04	0.04	0.03
FALSE	-0.7	0.66	0.11	-0.03	0.02
FALSE	0.19	0.66	0.03	0.03	0.04
TRUE	0.66	-0.17	-0.03	-0.02	0.02
FALSE	-0.54	0.66	-0.04	0.13	0.01
FALSE	-0.24	0.65	0.06	-0.02	0.06
TRUE	0.65	0.04	-0.04	0.07	-0.02
FALSE	-0.26	0.65	-0.01	0.11	-0.01
FALSE	0.63	0.64	0.04	0.04	0.02
FALSE	-0.38	0.64	0.02	0.07	0.01
TRUE	0.64	-0.79	-0.01	-0.14	0.03
TRUE	0.64	-1.35	-0.06	-0.06	-0.1
FALSE	-0.84	0.63	0.08	0.05	-0.03
TRUE	0.63	-1.76	-0.11	-0.1	-0.06
FALSE	0.29	0.62	-0.02	0.17	-0.05
FALSE	-0.38	0.62	0.06	0.03	0.01
FALSE	0.82	0.61	0.03	0.03	0.03
TRUE	0.61	-1.45	-0.03	-0.19	-0.01
TRUE	0.6	0.44	-0.04	0.12	-0.01
FALSE	-0.31	0.6	0.07	0.01	0.01
FALSE	-0.81	0.6	0.03	0.05	0.02
TRUE	0.6	0.47	-0.03	0.12	-0.02
FALSE	0.29	0.59	0.04	0.03	0.03
TRUE	0.59	0.08	0.04	-0.03	0
FALSE	0.31	0.58	0.05	0	0.03
TRUE	0.58	-0.49	-0.01	-0.1	0.03
FALSE	0.34	0.58	0.03	0.05	0
TRUE	0.58	-2.18	-0.17	-0.12	-0.05
TRUE	0.57	-2.17	-0.09	-0.21	-0.05
FALSE	0.3	0.57	0.06	0.09	-0.07
TRUE	0.56	0.12	-0.08	0.14	-0.04
FALSE	0.32	0.56	0.07	0.01	0
FALSE	-0.92	0.56	0.07	-0.03	0.04
TRUE	0.54	1.29	-0.02	0.23	-0.01

FALSE	0.41	0.54	-0.05	0.12	0.02
FALSE	-0.09	0.53	-0.04	0.07	0.04
TRUE	0.53	-0.46	0.01	0.02	-0.1
FALSE	0.07	0.52	-0.01	0.07	0.01
FALSE	0.78	0.52	0.06	0.02	0.01
TRUE	0.52	-2.98	-0.19	-0.18	-0.09
TRUE	0.51	0.88	0.09	0.07	-0.04
FALSE	1.24	0.51	0	0.08	-0.01
TRUE	0.5	1.03	0.15	0.03	-0.03
TRUE	0.5	-2.31	-0.12	-0.13	-0.11
FALSE	-0.63	0.49	0.05	-0.05	0.07
FALSE	-1.04	0.47	0.01	0.07	0
FALSE	0.15	0.47	0.01	0.08	-0.02
FALSE	0.36	0.47	-0.04	0.04	0.06
FALSE	1.15	0.46	0.05	0.02	0.01
FALSE	0.77	0.46	0.09	0	-0.01
FALSE	0.43	0.45	0.07	0.04	-0.05
TRUE	0.44	-0.62	0.06	-0.06	-0.09
TRUE	0.44	-2.42	-0.12	-0.23	-0.02
FALSE	0.08	0.44	-0.02	0.05	0.03
FALSE	0.85	0.44	0	0.01	0.06
TRUE	0.44	1.75	0.06	0.2	0.01
FALSE	0.7	0.43	0	0.16	-0.09
TRUE	0.43	-0.32	0.04	-0.02	-0.07
FALSE	-0.24	0.43	0.09	-0.08	0.06
FALSE	-0.46	0.42	0.02	0.01	0.04
FALSE	-0.6	0.42	0	0.02	0.05
TRUE	0.42	-1.19	-0.09	-0.05	-0.04
TRUE	0.42	0.54	-0.03	0.11	0.01
FALSE	-0.38	0.42	-0.02	0.04	0.05
FALSE	0.88	0.41	-0.05	0.07	0.05
FALSE	1.23	0.41	0.02	0.08	-0.03
FALSE	-0.21	0.41	0.09	-0.09	0.05
FALSE	0.29	0.4	0.06	-0.01	0.01
FALSE	-0.94	0.4	0.09	-0.07	0.04
TRUE	0.39	-2.14	-0.1	-0.21	-0.03
FALSE	-0.43	0.39	0.06	-0.05	0.05
TRUE	0.39	0.47	-0.01	0.1	-0.03
TRUE	0.39	-1.94	-0.12	-0.16	-0.03
FALSE	1.26	0.38	0.04	0.01	0.01
FALSE	-0.83	0.37	0.05	0.02	-0.01
TRUE	0.37	1.68	0.15	0.09	0.02
FALSE	-0.37	0.37	0	0.04	0.02
FALSE	-0.11	0.36	0.01	0.06	-0.01
TRUE	0.36	-1.31	-0.09	-0.03	-0.08
FALSE	0.79	0.35	0.04	0	0.02
TRUE	0.35	1.65	0.09	0.18	-0.01
FALSE	-0.84	0.35	0.03	-0.05	0.07
FALSE	0.87	0.35	-0.1	0.15	0
FALSE	0.5	0.34	0.03	0	0.01
FALSE	0.09	0.34	0.1	-0.04	-0.02
FALSE	-1.1	0.33	0.04	0	0.01
FALSE	0.54	0.32	0	0.01	0.03
FALSE	-1.07	0.31	0.09	-0.03	-0.01
FALSE	-0.24	0.31	-0.01	0	0.06
TRUE	0.31	0.72	0.05	0.06	0.01
TRUE	0.3	-1.32	-0.13	-0.05	-0.03
FALSE	0.62	0.3	0.05	-0.01	0.01

FALSE	-0.1	0.29	0.02	0.07	-0.05
FALSE	0.4	0.29	0.1	-0.08	0.03
FALSE	-0.65	0.29	0.03	-0.01	0.02
FALSE	0.91	0.29	0.06	-0.01	0
TRUE	0.28	-0.64	0.03	-0.16	0.04
FALSE	-0.86	0.27	0.01	-0.04	0.07
FALSE	-0.76	0.27	0.01	-0.04	0.08
FALSE	0.83	0.26	0.03	-0.02	0.04
FALSE	-0.76	0.26	0	0	0.04
TRUE	0.24	-1.64	-0.06	-0.18	-0.02
FALSE	0.26	0.24	-0.07	0.1	0.01
TRUE	0.24	0.72	0.05	0.02	0.03
FALSE	-0.75	0.23	0.05	0.02	-0.04
FALSE	0.39	0.23	0.05	-0.02	0.01
FALSE	-0.12	0.22	-0.03	0.02	0.04
TRUE	0.22	-2.28	-0.15	-0.14	-0.06
FALSE	-0.26	0.22	0.02	-0.02	0.03
FALSE	-0.25	0.21	-0.03	0.06	0
FALSE	-0.1	0.21	0.08	0.02	-0.07
TRUE	0.2	-0.38	-0.02	-0.09	0.05
TRUE	0.19	0.81	0	0.14	-0.02
TRUE	0.19	-1.23	-0.03	-0.14	-0.03
FALSE	1.15	0.19	-0.09	0.23	-0.11
FALSE	0.21	0.19	-0.02	-0.03	0.08
FALSE	-0.03	0.18	0.08	-0.07	0.02
TRUE	0.17	-0.96	-0.06	-0.09	0
TRUE	0.16	-1.75	-0.12	-0.12	-0.03
FALSE	1.09	0.15	0.01	-0.05	0.06
TRUE	0.15	-1.74	-0.08	-0.14	-0.05
TRUE	0.13	2.4	0.1	0.24	0.03
FALSE	-0.41	0.13	0.06	-0.11	0.05
FALSE	0.39	0.11	-0.16	0.18	0
FALSE	-1.07	0.11	0.01	-0.02	0.02
FALSE	1.12	0.11	0.01	0	0.01
TRUE	0.11	-1.12	-0.01	-0.16	-0.01
FALSE	0.59	0.1	0.06	0	-0.04
FALSE	0.9	0.09	0.05	-0.08	0.06
TRUE	0.09	-0.67	0.02	-0.06	-0.08
FALSE	0.03	0.09	0.06	-0.02	-0.04
FALSE	0.9	0.08	-0.01	0.03	-0.01
TRUE	0.08	-0.05	0.04	0.02	-0.08
TRUE	0.08	-0.67	-0.06	-0.03	-0.02
FALSE	-0.63	0.08	0	-0.02	0.03
FALSE	0.62	0.08	-0.04	0.13	-0.08
FALSE	-0.13	0.07	-0.02	0.02	0
TRUE	0.07	1.23	-0.02	0.13	0.07
FALSE	1.27	0.07	-0.17	0.19	0
FALSE	0.16	0.07	0.03	-0.07	0.04
TRUE	0.05	-2.26	-0.08	-0.18	-0.09
TRUE	0.05	0.84	0.12	0.04	-0.03
FALSE	0.62	0.04	0.04	-0.07	0.03
TRUE	0.04	0.61	0.08	-0.01	0.01
TRUE	0.03	1.07	0.18	0	-0.03
TRUE	0.02	-0.11	-0.02	0.03	-0.03
FALSE	-0.21	0.02	0.03	-0.06	0.04
FALSE	-0.65	0.02	0.04	-0.06	0.02
FALSE	0.06	0.02	-0.01	0.05	-0.03
FALSE	1.39	0.01	-0.08	0.15	-0.05

FALSE	0.94	0.01	-0.17	0.17	0.01
FALSE	0.86	0.01	-0.08	0.11	-0.02
FALSE	-1.12	0	0.03	0.01	-0.04
FALSE	-1.37	0	0.06	-0.05	-0.02
FALSE	-1.23	0	0.01	-0.05	0.05
FALSE	0.52	0	0.03	-0.05	0.04
TRUE	0	-0.85	-0.02	-0.14	0.02
FALSE	-0.19	-0.01	0.03	-0.03	0
FALSE	0.12	-0.01	0.05	-0.07	0.02
FALSE	0.02	-0.02	0.01	0.02	-0.02
FALSE	-0.69	-0.04	0.04	-0.03	-0.03
TRUE	-0.04	-2.55	-0.12	-0.24	-0.05
FALSE	-0.32	-0.06	0.05	-0.06	0.01
FALSE	-0.53	-0.06	-0.07	0.06	0.01
FALSE	-1.76	-0.06	-0.03	0.03	0
FALSE	-0.14	-0.06	0.04	-0.06	0.01
FALSE	-1.15	-0.07	0.04	-0.1	0.04
FALSE	-0.12	-0.08	0.04	-0.1	0.05
FALSE	-1.04	-0.08	0.04	-0.13	0.07
FALSE	0.16	-0.08	-0.09	0.12	-0.03
FALSE	0.46	-0.09	0.03	-0.03	-0.02
FALSE	-0.64	-0.1	0.01	-0.05	0.01
FALSE	-0.54	-0.1	0.01	0	-0.02
TRUE	-0.11	0.44	0.05	0.11	-0.09
FALSE	1.11	-0.12	-0.13	0.14	-0.03
FALSE	-0.57	-0.13	0.01	-0.08	0.05
FALSE	0.87	-0.13	-0.06	0.1	-0.05
FALSE	-0.06	-0.14	0.09	-0.1	-0.01
FALSE	-0.85	-0.14	-0.02	-0.08	0.07
FALSE	0.22	-0.14	-0.09	0.04	0.04
FALSE	0.19	-0.15	0.03	-0.07	0.02
FALSE	-0.74	-0.15	0	-0.06	0.05
TRUE	-0.16	-0.44	-0.05	-0.05	0.02
FALSE	0.2	-0.16	0.02	-0.1	0.05
TRUE	-0.17	-1.09	-0.09	-0.03	-0.06
FALSE	0.5	-0.17	-0.04	0.11	-0.09
TRUE	-0.17	-1	-0.03	-0.13	0
FALSE	-0.29	-0.18	-0.05	0.02	0
TRUE	-0.18	0.67	0.18	-0.04	-0.04
FALSE	0.39	-0.18	-0.02	-0.05	0.03
TRUE	-0.19	0.35	0.06	0.03	-0.04
FALSE	0.62	-0.19	0.04	-0.07	0
FALSE	-0.53	-0.19	0.01	-0.01	-0.03
TRUE	-0.2	-0.28	-0.07	0.02	0.01
FALSE	-1.02	-0.2	-0.07	-0.01	0.05
FALSE	0.2	-0.21	0	0	-0.02
FALSE	-0.42	-0.22	-0.02	-0.05	0.04
FALSE	-0.75	-0.22	0	-0.09	0.06
TRUE	-0.23	-1.93	-0.12	-0.1	-0.08
FALSE	-0.86	-0.23	0.01	-0.08	0.05
FALSE	-0.76	-0.23	-0.06	-0.02	0.04
TRUE	-0.23	-0.16	0.08	-0.09	-0.02
FALSE	-0.73	-0.23	-0.05	-0.03	0.04
TRUE	-0.25	-1.29	-0.06	-0.13	-0.02
FALSE	-0.47	-0.25	-0.05	0.01	0
FALSE	-0.42	-0.25	-0.02	-0.02	0
FALSE	0.11	-0.25	0.01	-0.05	0
FALSE	-0.89	-0.26	0.06	-0.07	-0.03

TRUE	-0.26	-1.37	-0.14	-0.04	-0.02
TRUE	-0.26	0.16	0.02	-0.05	0.04
FALSE	-0.58	-0.27	-0.06	0.02	-0.01
TRUE	-0.27	-1	-0.02	-0.13	0
TRUE	-0.28	-0.6	0.01	-0.09	-0.01
FALSE	0.92	-0.29	-0.01	0	-0.02
FALSE	0.18	-0.3	-0.08	0.04	0
FALSE	-1.5	-0.3	0.05	-0.13	0.04
TRUE	-0.3	-1.9	-0.14	-0.1	-0.05
FALSE	1.22	-0.3	-0.05	0.03	-0.02
FALSE	0.2	-0.31	0.03	-0.07	-0.01
FALSE	0.32	-0.32	-0.01	-0.05	0
TRUE	-0.33	0.32	0	0.11	-0.05
TRUE	-0.33	-0.7	-0.07	-0.01	-0.03
FALSE	-0.54	-0.34	-0.01	-0.06	0.02
FALSE	-1.31	-0.35	0.04	-0.09	0
FALSE	-0.15	-0.35	0.01	-0.1	0.05
FALSE	-0.16	-0.35	-0.03	-0.04	0.01
TRUE	-0.35	0.23	-0.03	0.03	0.03
FALSE	0.06	-0.35	-0.08	0.04	-0.01
FALSE	-0.15	-0.37	-0.01	-0.04	-0.01
FALSE	0.58	-0.37	-0.08	0.02	0.01
FALSE	-0.28	-0.37	0.01	-0.08	0.02
FALSE	-0.24	-0.37	0	-0.09	0.05
FALSE	-0.12	-0.38	0.03	-0.05	-0.04
FALSE	0.1	-0.38	0.08	-0.15	0
TRUE	-0.39	-1.58	-0.1	-0.12	-0.03
FALSE	-1.27	-0.39	0.02	-0.12	0.03
FALSE	0.18	-0.41	-0.06	0.09	-0.1
FALSE	-0.89	-0.41	-0.01	-0.04	-0.02
FALSE	-0.33	-0.42	0.05	-0.09	-0.02
FALSE	0.74	-0.42	0.02	-0.14	0.04
FALSE	-0.58	-0.42	-0.03	-0.07	0.04
FALSE	-1.38	-0.44	0.02	-0.15	0.07
FALSE	-0.27	-0.44	0	-0.09	0.03
FALSE	0.29	-0.51	-0.03	-0.07	0.03
FALSE	0.81	-0.52	0.03	-0.08	-0.02
FALSE	0.04	-0.53	0.05	-0.06	-0.08
FALSE	0.48	-0.54	-0.03	-0.03	-0.02
FALSE	-0.22	-0.54	0	-0.14	0.04
FALSE	0	-0.55	-0.03	-0.03	-0.02
FALSE	-0.78	-0.55	0	-0.1	0.01
FALSE	-0.92	-0.55	-0.01	-0.05	-0.02
FALSE	0.12	-0.55	-0.05	0.01	-0.04
FALSE	0.21	-0.56	-0.03	-0.02	-0.04
FALSE	-1.11	-0.57	-0.05	-0.04	0.02
FALSE	0.28	-0.58	-0.09	-0.01	0.01
FALSE	0.95	-0.59	-0.02	-0.08	0.02
FALSE	-0.33	-0.61	-0.05	-0.08	0.03
FALSE	-0.88	-0.61	0.02	-0.15	0.05
TRUE	-0.61	-1.48	-0.09	-0.14	-0.01
FALSE	0.41	-0.63	-0.01	-0.14	0.04
FALSE	1.48	-0.64	-0.11	0.02	0
FALSE	0.15	-0.65	0	-0.03	-0.06
FALSE	-0.12	-0.67	0	-0.1	0
FALSE	-0.38	-0.71	-0.07	-0.06	0.03
FALSE	0.5	-0.71	-0.07	-0.07	0.02
FALSE	-0.51	-0.72	-0.02	-0.06	-0.03

FALSE	-0.2	-0.72	0.01	-0.14	0.02
FALSE	-0.81	-0.74	-0.01	-0.1	-0.01
FALSE	-0.19	-0.75	-0.12	0.06	-0.05
FALSE	-0.41	-0.75	-0.03	-0.08	0.01
FALSE	1.3	-0.75	-0.03	-0.07	-0.01
FALSE	0.38	-0.76	0.02	-0.15	0.01
FALSE	-0.29	-0.77	-0.05	-0.05	-0.03
FALSE	-1.65	-0.79	-0.05	-0.06	0.01
FALSE	-0.7	-0.79	-0.01	-0.16	0.06
FALSE	-0.63	-0.8	-0.02	-0.14	0.05
TRUE	-0.8	-1.36	-0.03	-0.17	-0.02
FALSE	0.09	-0.81	-0.08	-0.04	-0.01
FALSE	-0.98	-0.81	-0.06	-0.11	0.05
FALSE	1.25	-0.85	-0.06	-0.04	-0.02
FALSE	-0.45	-0.85	-0.05	-0.11	0.03
FALSE	-0.87	-0.85	-0.03	-0.13	0.04
FALSE	-0.69	-0.85	0	-0.13	0
FALSE	-0.42	-0.85	-0.04	-0.08	0
FALSE	0.38	-0.87	-0.08	-0.01	-0.05
FALSE	-0.97	-0.87	-0.01	-0.15	0.04
FALSE	0.72	-0.87	-0.1	-0.02	-0.01
FALSE	0.88	-0.89	0	-0.13	0.01
FALSE	-0.61	-0.9	-0.05	-0.04	-0.04
FALSE	-0.16	-0.9	-0.06	-0.07	0
FALSE	-0.51	-0.9	-0.03	-0.14	0.01
FALSE	-0.23	-0.9	-0.05	-0.09	0
TRUE	-0.91	-0.5	0	-0.12	0.04
FALSE	0.54	-0.92	-0.02	-0.06	-0.07
FALSE	-1.09	-0.95	-0.07	-0.1	0.02
FALSE	0.8	-0.96	-0.1	-0.01	-0.03
FALSE	0.08	-0.98	-0.02	-0.09	-0.05
FALSE	-0.38	-1	-0.06	-0.1	0
FALSE	0.3	-1.02	-0.01	-0.16	0.02
TRUE	-1.05	0.25	0.07	-0.1	0.06
FALSE	-1.98	-1.06	-0.04	-0.09	-0.03
FALSE	0.17	-1.07	-0.14	-0.06	0.04
FALSE	-0.38	-1.08	-0.12	-0.05	0.01
FALSE	-1.27	-1.12	-0.01	-0.16	0
FALSE	0.05	-1.2	-0.1	-0.06	-0.02
FALSE	-0.3	-1.22	-0.03	-0.15	0
FALSE	-0.61	-1.23	-0.08	-0.09	-0.02
FALSE	-0.34	-1.23	-0.08	-0.1	0
FALSE	0.77	-1.24	-0.03	-0.15	-0.02
FALSE	0.61	-1.28	-0.02	-0.14	-0.04
FALSE	0.73	-1.34	-0.11	-0.09	-0.01
FALSE	0.11	-1.35	-0.06	-0.12	-0.02
FALSE	0.84	-1.35	-0.11	-0.04	-0.05
FALSE	-0.08	-1.35	-0.05	-0.16	0
FALSE	0.74	-1.36	-0.11	-0.06	-0.04
FALSE	-0.55	-1.39	-0.07	-0.08	-0.06
FALSE	0.11	-1.43	-0.08	-0.14	0
FALSE	-0.28	-1.48	-0.08	-0.03	-0.11
FALSE	-0.25	-1.48	-0.07	-0.19	0.02
FALSE	1.37	-1.59	-0.11	-0.03	-0.11
FALSE	1.3	-1.63	-0.03	-0.18	-0.03
FALSE	-0.32	-1.63	-0.07	-0.07	-0.12
FALSE	-0.5	-1.65	-0.09	-0.13	-0.03
FALSE	0.6	-1.74	-0.16	-0.09	0

FALSE	-0.51	-1.75	-0.06	-0.2	-0.01
FALSE	1.77	-1.79	-0.14	-0.12	-0.01
FALSE	-0.74	-1.85	-0.12	-0.13	-0.02
FALSE	0.46	-1.87	-0.16	-0.04	-0.08
FALSE	0.6	-1.93	-0.05	-0.17	-0.08
FALSE	0.1	-2.01	-0.12	-0.17	-0.02
FALSE	0.22	-2.1	-0.16	-0.17	0
FALSE	0.99	-2.15	-0.14	-0.11	-0.08
FALSE	-0.36	-2.21	-0.12	-0.2	-0.02
FALSE	0.28	-2.34	-0.12	-0.14	-0.1
FALSE	0.95	-2.43	-0.11	-0.24	-0.03
FALSE	1	-2.64	-0.15	-0.23	-0.02
FALSE	0.15	-2.7	-0.19	-0.17	-0.05
FALSE	0.51	-2.8	-0.15	-0.2	-0.09
FALSE	0.81	-3.01	-0.15	-0.26	-0.05
